# Genomic Signatures After Five Generations of Intensive Selective Breeding: Runs of Homozygosity and Genetic Diversity in Representative Domestic and Wild Populations of Turbot (*Scophthalmus maximus*)

**DOI:** 10.3389/fgene.2020.00296

**Published:** 2020-04-03

**Authors:** Oscar Aramburu, Francisco Ceballos, Adrián Casanova, Alan Le Moan, Jakob Hemmer-Hansen, Dorte Bekkevold, Carmen Bouza, Paulino Martínez

**Affiliations:** ^1^Department of Zoology, Genetics and Physical Anthropology, Faculty of Veterinary, Universidade de Santiago de Compostela, Lugo, Spain; ^2^Instituto de Acuicultura, Universidade de Santiago de Compostela, Santiago de Compostela, Spain; ^3^Sydney Brenner Institute for Molecular Bioscience, Faculty of Health Sciences, University of the Witwatersrand, Johannesburg, Johannesburg, South Africa; ^4^National Institute of Aquatic Resources, Technical University of Denmark, Silkeborg, Denmark

**Keywords:** turbot, SNP panels, runs of homozygosity, genetic diversity, selective sweep

## Abstract

Massive genotyping of single nucleotide polymorphisms (SNP) has opened opportunities for analyzing the way in which selection shapes genomes. Artificial or natural selection usually leaves genomic signatures associated with selective sweeps around the responsible locus. Strong selective sweeps are most often identified either by lower genetic diversity than the genomic average and/or islands of runs of homozygosity (ROHi). Here, we conducted an analysis of selective sweeps in turbot (*Scophthalmus maximus*) using two SNP datasets from a Northeastern Atlantic population (36 individuals) and a domestic broodstock (46 individuals). Twenty-six families (∼ 40 offspring per family) from this broodstock and three SNP datasets applying differing filtering criteria were used to adjust ROH calling parameters. The best-fitted genomic inbreeding estimate (F_ROH_) was obtained by the sum of ROH longer than 1 Mb, called using a 21,615 SNP panel, a sliding window of 37 SNPs and one heterozygous SNP per window allowed. These parameters were used to obtain the ROHi distribution in the domestic and wild populations (49 and 0 ROHi, respectively). Regions with higher and lower genetic diversity within each population were obtained using sliding windows of 37 SNPs. Furthermore, those regions were mapped in the turbot genome against previously reported genetic markers associated with QTL (Quantitative Trait Loci) and outlier loci for domestic or natural selection to identify putative selective sweeps. Out of the 319 and 278 windows surpassing the suggestive pooled heterozygosity thresholds (ZHp) in the wild and domestic population, respectively, 78 and 54 were retained under more restrictive ZHp criteria. A total of 116 suggestive windows (representing 19 genomic regions) were linked to either QTL for production traits, or outliers for divergent or balancing selection. Twenty-four of them (representing 3 genomic regions) were retained under stricter ZHp thresholds. Eleven QTL/outlier markers were exclusively found in suggestive regions of the domestic broodstock, 7 in the wild population and one in both populations; one (broodstock) and two (wild) of those were found in significant regions retained under more restrictive ZHp criteria in the broodstock and the wild population, respectively. Genome mining and functional enrichment within regions associated with selective sweeps disclosed relevant genes and pathways related to aquaculture target traits, including growth and immune-related pathways, metabolism and response to hypoxia, which showcases how this genome atlas of genetic diversity can be a valuable resource to look for candidate genes related to natural or artificial selection in turbot populations.

## Introduction

Artificial selection has been accomplished by humans to domesticate species with desirable properties, in order to reach more profitable phenotypes. Despite its differences with natural selection, both fit to the same general principle: selective pressure is indirectly applied on specific genomic regions where genes controlling relevant traits are found, thus modifying the allelic frequencies in the target population (Brito et al., 2017). The main difference between them is that domestication involves the relaxation of the selective pressure applied on fitness traits relevant for survival in wild populations, while the pressure on traits relevant for production is intensified (Sun et al., 2014).

A strong selective pressure leads to an increase in the frequencies of favorable allelic variants and, consequently, to a decrease of genetic variation in the gene/s under selection. Moreover, due to the physical association of that locus with nearby loci (genetic linkage), genetic variants of loci in the vicinity are also suffering a decrease of genetic variability through hitch-hiking effects, thus being deviated from the expected proportions under neutrality (Sun et al., 2014). Accordingly, a pattern of linkage disequilibrium (LD) will emerge around the locus targeted by selection. This train of “dragging” events is known as a selective sweep and leaves behind detectable signatures at the genome level (Smith and Haigh, 1974; Qanbari et al., 2012).

In most studies, only strong selective sweeps (where only one allele is targeted by selection) have been described (Johansson et al., 2010; Rubin et al., 2010, 2012; Ramey et al., 2013). Soft selective sweeps (where more than one allele is targeted by selection) are much harder to detect due to their resistance to heterozygosity decrease (Sun et al., 2014), so they are underrepresented in scientific studies (Messer and Petrov, 2013). Since strong selective sweeps determine the increase in the frequency of a specific allele (in some cases leading to complete fixation), its detection is much easier than in soft selective sweeps. Therefore, they are useful for the detection of candidate genes responsible for important traits related to domestication and selection (Smith and Haigh, 1974; Sun et al., 2014). However, care must be taken since candidate regions subject to strong selective sweeps might also represent false positives due to genetic drift (through effects of effective size, founder effect, bottlenecks) or inbreeding (Purfield et al., 2017).

There are several methods to identify selective sweeps through a genomic screening using large numbers of markers, such as SNP (Single Nucleotide Polymorphism) loci. Two main approaches have been particularly employed to identify signatures of selection (Bonhomme et al., 2015). One of them explores the distribution of genetic diversity across the genome using indexes such as pooled heterozygosity, which has been applied in different livestock species (Rubin et al., 2010, 2012; Qanbari et al., 2012; Sun et al., 2014; Zwane et al., 2019). On the other hand, the analysis of ROH (Runs of Homozygosity), among its many applications, has frequently been used to study genomic signatures of selection (Metzger et al., 2015). A strong selective pressure will reduce genetic diversity on the targeted locus and neighbor loci. The newly formed haplotypes could give rise to ROHs among the individuals of the population experiencing the selective pressure. ROH analysis represents an informative methodology to address demographic studies (Kirin et al., 2010; Ceballos et al., 2018b) and to map recessive mutations in complex diseases (Nalls et al., 2009). In some domestic animals, it has been used to estimate genetic diversity across the genome (Metzger et al., 2015).

Furthermore, genome-wide ROH analysis is a useful tool to identify genomic signatures of inbreeding. Inbreeding reduces genetic diversity within individuals, leading to the appearance of long ROH genome stretches. The longer the ROH, the more recently the consanguineous event has occurred, since recombination would have had fewer opportunities to break up those ROH (Purfield et al., 2017). ROH analysis has provided more precise inbreeding estimates than methods based on pedigrees (Ferenčaković et al., 2017). However, care must be taken since not all ROHs can be attributable to Identity by Descent (IBD). Short ROH segments can occur by chance (Identity by State; IBS) (Marras et al., 2014) due to the uneven recombination frequency across the genome. This fact determines the existence of common haplotypes among non-related ancestors as a consequence of genetic drift or the evolutionary history of the population under study (Purfield et al., 2017). Genome-wide ROH analysis has also facilitated the study of inbreeding in natural populations in evolutionary and conservation studies (Kardos et al., 2016).

Selective sweeps have been detected in various domestic species such as goat (Brito et al., 2017), cattle (Gutiérrez-Gil et al., 2015), sheep (Moradi et al., 2012), chicken (Rubin et al., 2010; Qanbari et al., 2012), pig (Rubin et al., 2012), and dog (Quilez et al., 2011). However, to our knowledge, studies of selective sweeps related to fish domestication and its impact are scarce, since most breeding programs are relatively new (half a century). Recently, the availability of increasing genomic resources and tools has allowed the tracking of selection events in some fish species such as stickleback (Cano et al., 2006; Jones et al., 2012), Atlantic salmon (Vasemägi et al., 2012; Barson et al., 2015) and catfish (Sun et al., 2014).

Turbot breeding programs started in the 1990s and are currently in the 5th generation of selection (Martínez et al., 2016). Domestic turbot is of Atlantic origin in the three European companies with selective breeding programs. Wild Atlantic turbot populations show genetic homogeneity and are considered as a nearly panmictic unit (Vilas et al., 2015; do Prado et al., 2018b). The turbot genome has recently been assembled (Figueras et al., 2016) and anchored to a high-density genetic map (Maroso et al., 2018). Linkage maps (Bouza et al., 2007, 2012; Martínez et al., 2008; Hermida et al., 2013) have been used in this species to localize QTL related to the main traits for selective breeding programs, namely sex determination (Martínez et al., 2009), growth (Sánchez-Molano et al., 2011; Rodríguez-Ramilo et al., 2014) and resistance to various pathogens (Rodríguez-Ramilo et al., 2011, 2013, 2014), as a step toward marker assisted selection (Sciara et al., 2018). Furthermore, studies have also provided outlier markers and candidate genomic regions related to adaptive variation in wild (do Prado et al., 2018b) and domestic turbot (do Prado et al., 2018a). Recent advances in genotyping by sequencing and whole-genome resequencing have provided a large number of SNP loci covering the 567-Mb turbot genome (Maroso et al., 2018). These resources bring up new opportunities for genome scanning in order to study genetic diversity and selection signatures in turbot at even higher marker density than that reported in other mammalian livestock (Brito et al., 2017).

The objectives of this study are: (i) to conduct a genome-wide characterization of ROH and genetic diversity in wild and domestic turbot using a large number of SNP loci; obtaining firstly meaningful computational conditions to accurately call ROH and estimate the genomic inbreeding coefficient (F_ROH_), and secondly the ROH distribution and F_ROH_ in the wild and domestic turbot samples; (ii) to co-localize previously reported QTL and outlier markers associated with productive traits and adaptive variability with genomic regions of high and low genetic diversity suggestive of selection in wild and domestic turbot; and (iii) to explore gene functions and pathways associated with these regions through preliminary genome mining and functional annotation analyses.

## Materials and Methods

### Ethics Statement

The animal experimental procedures carried out in this study were approved and conducted in strict accordance with the Directive 2010/63/EU of the European Parliament and of the Council of 22 September 2010 on the protection of animals used for scientific purposes.

### Domestic Individuals

When the study was performed, the four active European hatcheries were founded with individuals from the Atlantic, where populations are genetically rather homogeneous (*F*_ST_ = 0.0024; do Prado et al., 2018b). The broodstock of CETGA (Aquaculture Cluster of Galicia, Ribeira, Spain), constituted by a mix of these four hatcheries, provided a good representation of domestic turbot (do Prado et al., 2018a), and it was used to analyze both the ROHi (Runs of Homozygosity islands) and the distribution of genetic diversity in our study. Despite different breeding strategies, the same traits have been targeted in the turbot breeding programs, i.e., growth-related traits and resistance to major infectious diseases affecting turbot production (Sánchez-Molano et al., 2011; Rodríguez-Ramilo et al., 2014), suggesting that processes of convergent selection may have shaped the genome of the different turbot broodstock. We analyzed a random sample of the CETGA broodstock consisting of 46 breeders, which were additionally used for the foundation of 44 families (average of 39.5 individuals and range of 36–45 individuals per family) in the framework of the FISHBOOST project (EU 613611; Anacleto et al., 2019; Saura et al., 2019). Twenty-six of these 44 families were used in this study in order to determine the best parameters for ROH calling.

The raw genotyping data for our study was taken from Maroso et al. (2018), which followed a 2b-RAD protocol for SNP genotyping. We considered three different SNP panels for genotyping the CETGA sample and the families analyzed. These panels derived from the same bioinformatic pipeline but used more relaxed or stricter quality control (QC) procedures (Maroso et al., 2018). The panels consisted of 25,511, 21,615 (Supplementary Table 1) and 18,198 SNP, at an approximate density of 1 SNP per 22, 26, and 31 kb, respectively, across the turbot genome (567 Mb; Figueras et al., 2016; Maroso et al., 2018). The performance of these three panels was compared in order to select the most adequate SNP panel for ROH calling.

### Wild Individuals

The wild population consisted of 36 individuals from the Major Fishing Area 27-4b (Atlantic NE, North Sea). Previous reports suggested that this is a panmictic population not introgressed from hatchery turbot releases that were carried out in the past in the North Sea (do Prado et al., 2018a). Environmental factors and neutral forces shaping the genome of this population have been reported by do Prado et al. (2018b).

DNA was extracted using the DNeasy blood tissue kit following provider protocol (Qiagen), while library preparation was carried out following a modified version of the ddRAD paired-end protocol from Poland and Rife (2012). *Pst1* and *Msp1* restriction enzymes were used for targeting RAD-tags followed by an additional agarose gel selection between 350 and 500 bp. After a PCR amplification phase (12 cycles), the library was purified with AMPure beads. The quality was controlled on a bioanalyzer using the high sensitivity DNA reagent (Agilent Technologies). The final library was then sequenced on one lane of Illumina HiSeq4000 at the BGI sequencing platform.

The pipeline for SNP calling, filtering and genotyping developed for domestic turbot by Maroso et al. (2018) was adapted for the wild population data as follows. Raw reads were head-trimmed to 85 bp using Trimmomatic (Bolger et al., 2014), except for the forward sequences on the second library: in order to address differences in library preparation, those reads needed to be head-trimmed first to 87 bp and later tail-trimmed to 85 bp using Stacks’ *process_radtags* pipeline (Catchen et al., 2013). *Process_radtags* was also used to filter raw reads according to sequence quality, using a 9 bp sliding window and allowing a minimum score of 20 (-w 0.1 -s 20).

Filtered reads were mapped against the latest version of the turbot reference genome (ASM318616v1; Figueras et al., 2016). Bowtie1.1.2 (Langmead et al., 2009) was used despite the existence of a more advanced version (Bowtie2; Langmead and Salzberg, 2012) since control measures are more straightforward in the former version, and the only drawback (processing speed) was negligible for our dataset. The following parameters were set: (i) reads were only considered when aligned to a single site in the reference genome (-m 1); (ii) the mismatch-threshold allowed up to 3 for 85 bp fragments (-v 3); (iii) overlapping was avoided by setting the minimum insert size to 170 bp (-I 170, 85 bp + 85 bp); and (iv) the maximum insert size was set to 500 bp (-X 500).

Mapped reads were processed with the Stacks2.2 *gstacks* module (Catchen et al., 2013). Default parameters were set, except for a stricter alpha threshold for genotype-calling (–gt-alpha 0.01) and without soft-clipping of the 5′ and 3′ ends (–max-clipped 0). Stacks’ *populations* module (Catchen et al., 2013) was used for further filtering and parameters were set so that only SNPs genotyped in > 56% of the individuals (-r 0.56; 20 individuals) were considered; no additional MAF filters (–min_maf 0) were added.

Using VCFtools (Danecek et al., 2011) we considered only those genotypes with a minimum read depth of 8 reads (–minDP 8). Due to the low read representation in some loci after this filtering step, the 20-individuals-filter was applied again, as well as a filter for monomorphic SNPs. Finally, in those cases where a RAD-tag contained more than one SNP, only the closest one to the 5′ end was retained, according to the filtering pipeline used in the domestic broodstock (Maroso et al., 2018).

### Offspring’s Inbreeding Coefficient Estimation From Domestic Parent’s Identity by Descent

Identity by descent estimates for each couple in the 26 domestic families analyzed were calculated using PLINK’s –genome flag (Purcell et al., 2007). PLINK employs a hidden Markov model (HMM) to infer underlying IBD in chromosomal segments based on observed IBS. PLINK’s HMM Z0, Z1, Z2 provide similar estimates of Cotterman coefficients of relatedness k0, k1, k2. Kinship coefficients of the parents (equal to the inbreeding coefficient of their offspring) were obtained by dividing IBD estimates by half (θ_IBD_). As some deviations between the genomic inbreeding coefficient calculated from ROH (F_ROH_) and the θ_IBD_ were expected, families were grouped by their θ_IBD_ status in five classes: unrelated (0 < θ_IBD_ < 0.0076), second cousin (0.0076 < θ_IBD_ < 0.038), first cousin (0.038 < θ_IBD_ < 0.0937), half-sibling (0.0937 < θ_IBD_ < 0.1872) and full-sibling relatives (θ_IBD_ > 0.1872).

### Obtaining Meaningful PLINK Conditions

The observational approach implemented by PLINK v1.9 was used to call ROHs. The simplicity of this approach allows efficient execution on data from different array platforms or sequencing technologies (Howrigan et al., 2011; Joshi et al., 2015; Ceballos et al., 2018a, b). Tests on simulated and real data showed that the approach used by PLINK outperformed its competitors in reliably detecting ROH.

Different PLINK conditions were used to obtain ROH from the three QC datasets. In order to search for the most meaningful conditions for accurate ROH calling, the minimum number of SNPs that a ROH is required to have (–homozyg-snp), the required minimum density to consider a ROH (–homozyg-density) and the number of SNPs that the sliding window must have (-homozyg-window-snp), ROHs were set up to change from 24 to 40 SNPs for each iteration. Moreover, the number of heterozygous SNPs allowed in a window (–homozyg-window-het) was set up to 0 (no heterozygous SNP allowed) and 1 (one heterozygous SNP allowed to account for genotyping errors) for each SNP-per-window iteration. The other parameters were constant for all iterations (–homozyg-kb 200; –homozyg-gap 1000; –homozyg-window-missing 5; –homozyg-windows-threshold 0.05).

### Obtaining the Genomic Inbreeding Coefficient (F_ROH_)

F_ROH_ was calculated for every PLINK condition tested as the total sum of ROH divided by the total length of the genome, under the lack of sex chromosome heteromorphism in turbot (Taboada et al., 2014), as follows:

$$F_{R\,O\,H}=\frac{\sum l\,e\,n_{R\,O\,H}}{l\,e\,n_{g\,e\,n\,o\,m\,e}}$$

Different ROH length cut-offs were used to obtain F_ROH_: 1, 2, 4, 8 and 10 Mb. In order to find the best cut-off and PLINK conditions, parental offspring F_ROH_ coefficients were regressed against the known F_IBD_.

### Genomic Distribution of ROH

Genomic regions where ROH are particularly prevalent (ROH islands: ROHi) were obtained for each family and for both populations analyzed, the broodstock and the wild ones. In order to search for ROH islands a sliding window of 50 kb was used. In every 50 kb genomic window the number of individuals in ROH was obtained. To know if a specific genomic window had a significant enrichment of ROH across the population a binomial test with *P* < 2 × 10^–5^ with Bonferroni correction for 640 windows was applied.

### Genetic Diversity and Selective Sweep Analysis

The selective sweep screening was performed using the SNP datasets for both the broodstock (21,615 SNP panel, see section “Results and Discussion”) and the wild populations. Both datasets were analyzed using a 37 SNP sliding window, previously set up as the best fitted for ROH-calling (see Results and Discussion), and 1 SNP sliding at a time. For each window, pooled heterozygosity (Hp) (Rubin et al., 2010, 2012), along with the ZHp score, were computed to estimate the patterns of genetic diversity across the turbot genome as follows;

$$H_{P}=\frac{2\,\sum n_{M\,A\,J}\,\sum n_{M\,I\,N}}{{\left(\sum n_{M\,A\,J}+\sum n_{M\,I\,N}\right)}^{2}}$$

$$Z\,H_{P}=\frac{\left(H_{P}-\mu \,H_{P}\right)}{\sigma \,H_{P}}$$

Windows with ZHp < −3 or ZHp > 3 were retained as strict regions with significant lower- or higher-than-average genetic diversity (Low-GD; High-GD), respectively. Given that identification of extreme diversity regions of heterogeneous physical length may be too restrictive (Stainton et al., 2016), less strict ZHp thresholds (≤ −2.5 and ≥ 2.5) were also evaluated to identify suggestive regions, considering the unequal genome distribution of the SNP panels. The retained windows were contrasted to the genome positions of QTL markers for the main productive traits (growth, sex determination, resistance to *Aeromonas salmonicida*, *Philasterides dicentrarchi* and VHSV) and outlier loci related to adaptive variation previously reported in domestic and wild turbot (Martínez et al., 2009; Rodríguez-Ramilo et al., 2011, 2013, 2014; Sánchez-Molano et al., 2011; do Prado et al., 2018a, b; Sciara et al., 2018), in order to identity putative selective sweeps as considered in other livestock species (Stainton et al., 2016; Zwane et al., 2019).

### Gene Clustering and Functional Analysis

To identify the underlying biological functions of the candidate selective sweeps, the boundaries of selected candidate regions were used to retrieve gene lists from Ensembl-BioMart using the updated version of the turbot genome assembly (Maroso et al., 2018). The *in silico* analysis was conducted using the Functional Annotation Clustering tool implemented in DAVID (Huang da et al., 2009a, b) considering GO-term (Molecular Function 3 and 4; Biological Process 3 and 4), KEGG-pathway and UP_KEYWORDS annotation categories, as well as functional enrichment analyses (*P* < 0.05). The analysis was performed on overlapping regions between ROH islands (ROHi) and/or low-high genetic diversity regions taking as reference functional QTL markers and/or outlier loci.

## Results and Discussion

### SNP Genotyping Data

In this study, broad panels of SNPs derived from genotyping by sequencing protocols were genotyped (Supplementary Tables 3, 4), using the most recent turbot genome assembly (Maroso et al., 2018), and were applied to identify selective sweeps in wild and domestic turbot populations. This was specifically addressed by analyzing the distribution of ROH (runs of homozygosity) and by classifying low- versus high-GD (genetic diversity) regions across the turbot genome, an approach that has been frequently carried out in terrestrial livestock species, but scarcely to date in fish (Cano et al., 2006; Vasemägi et al., 2012; Sun et al., 2014).

Despite using SNP panels coming from two different genotyping by sequencing techniques, the final marker density was very similar for both the domestic and wild populations, and appropriate for the purpose of the study. Furthermore, an effort was done to homogenize genotyping in both populations by accommodating the filtering and genotyping pipeline to both datasets. A total of 21,615 SNP loci (Supplementary Table 1) were considered for the analysis of the domestic population after checking the performance of different stringency filters to accommodate the data to meaningful PLINK conditions (see below). Regarding the wild population, a total of 126,159,372 raw paired reads were produced, thus representing 3,504,427 raw paired reads on average for the 36 individuals (range: 1,021,421–7,790,680). Approximately 90.4% of these reads were retained after quality filtering through Stacks’ *process_radtags* pipeline. Reference mapping was conducted through the alignment of sequence reads with the current turbot genome assembly (Maroso et al., 2018) using Bowtie 1.1.2, keeping approximately 58.3% of the reads. The average number of reads per sample was 2,049,832.31 (range: 643,736–4,654,385).

After Stacks’ *populations* filter, 45,906 RAD-tags were retained for analysis in the wild population. Using VCF tools the coverage and 20-individuals filters were applied rendering a total of 28,790 RAD-tags (69,504 SNPs). However, only a total of 28,790 SNP loci were considered, since only one SNP was retained per RAD-tag. After deleting monomorphic SNPs (generated after the coverage filter), a final set of 25,681 SNP loci was obtained (Supplementary Table 2), which represents an average density of 1 SNP per 22 kb in the turbot genome (567 Mb).

The SNP densities handled for the small 567 Mb genome of turbot (Figueras et al., 2016; Maroso et al., 2018) were within the range in similar studies in other fish and vertebrates, representing even higher resolution than that reported in the larger mammal genomes (Marras et al., 2014; Brito et al., 2017).

### Obtaining Meaningful PLINK Conditions

Three different SNP panels were tested in 26 domestic families derived from the domestic parental broodstock in order to determine the best ROH calling parameters to fit estimation of kinship coefficients between breeders. The relationship between the minimum number of SNPs to call a ROH and the total ROH length retrieved supports that PLINK finds more ROH with datasets derived from more relaxed QC criteria and when considering fewer SNPs per sliding window by having more SNPs (Supplementary Figure 1). Also, the total sum of ROH changed dramatically by allowing 1 heterozygote SNP per PLINK’s sliding window.

In order to assess the outcome of allowing 1 or none heterozygous SNP per PLINK’s sliding window, the mean total length of ROH per family and kinship degree was plotted considering eight ROH size classes (Supplementary Figure 2). For each dataset, it is possible to see how by allowing 1 heterozygous SNP per sliding window a larger proportion of long ROH (>12 Mb) is systematically found in those families classified as full-siblings or half-siblings crosses by the IBD analysis. By not allowing any heterozygous SNP, these long ROH are broken, and thus, the number of medium-sized ROH is substantially increased (Ceballos et al., 2018a). The golden standard procedure to call for ROH in humans and livestock (Howrigan et al., 2011; Ferenčaković et al., 2017; Ceballos et al., 2018a) allows 1 or more heterozygous SNP, depending on the sequencing technology and marker density, in order to cope with calling errors and missing data that can mistakenly break ROH, thus increasing the power of detecting IBD.

Furthermore, it is shown that there are small differences between the strict (18,198 SNP) and the medium (21,615 SNP) QC criteria when 1 heterozygous SNP per sliding window is allowed, while large differences are detected with the very relaxed QC criterion (25,511 SNP; Supplementary Figure 2). Relaxed QC criteria enables SNP calling errors, since more relaxed QC procedures allow SNP calling errors associated with paralogous sequences or presence of null alleles (Maroso et al., 2018), capable of generating medium size ROHs. All in all, the use of the 21,615 SNP dataset through a sliding window of 37 SNP and allowing 1 heterozygous SNP seems to be the best fitting parameters for ROH analysis in our case.

### Obtaining Accurate Estimates of the Genomic Inbreeding Coefficient (F_ROH_)

An exploratory analysis was addressed to assess the effect of the different ROH size thresholds (ROH > 1 Mb, > 2 Mb, > 4 Mb, > 8 Mb, and > 10 Mb) on F_ROH_ estimation fitting to IBD (Supplementary Figure 3). Considering the three QC SNP dataset and 1 or 0 heterozygous SNP, the best fitted outcome was achieved when ROH longer than 1 Mb are considered θ_IBD_ (Table 1 and Supplementary Figure 3). Furthermore, Table 1 supports that the best conditions for ROH-calling and accurate F_ROH_ coefficient fitted to IBD are achieved when a medium QC criterion (21,615 SNP), 1 heterozygote SNP allowed per window and ROH longer than 1 Mb are applied. The size of a ROH is influenced by different factors such as recombination or linkage disequilibrium (Peripolli et al., 2016), although it has been found to be approximately correlated with its age: longer ROH will be inherited from recent ancestors, while shorter ROH will be from distant ancestors, as ROHs are broken down by recombination across generations (McQuillan et al., 2008; Marras et al., 2014). In this sense, it has been observed in other species (mammal livestock and humans) that very recent consanguinity, like that produced in an incestuous mating, generates very long ROH, longer than 8 Mb (Ceballos et al., 2018a; Goszczynski et al., 2018). The coefficient of variation (CV) is strikingly smaller when comparing intercepts and slopes of different QC procedures with 1 heterozygous SNP allowed per window. These CV are also smaller when using 1 Mb as ROH size threshold in comparison to longer cut-offs.

**TABLE 1:** Regression of the kinship coefficient (θ_IBD_) and the genomic inbreeding estimated through ROH (F_ROH_).

ROH category		18,198 SNP panel		21,615 SNP panel		25,511 SNP panel		CV (%)	
		0 Het	1 Het	0 Het	1 Het	0 Het	1 Het	0 Het	1 Het
ROH > 1 Mb	Intercept	0.011	0.011	0.008	0.011	0.008	0.012	19.25	5.09
	Slope	0.81	0.853	0.701	0.859	0.692	0.887	8.94	2.09
	R^2^	0.762	0.76	0.76	0.78	0.784	0.78		
ROH > 2 Mb	Intercept	0.008	0.012	0.004	0.008	0.004	0.009	43.30	21.53
	Slope	0.77	0.82	0.587	0.83	0.481	0.829	23.86	0.67
	R^2^	0.75	0.75	0.73	0.77	0.735	0.77		
ROH > 4 Mb	Intercept	0.004	0.08	0.001	0.003	0.004	0.005	57.74	149.63
	Slope	0.695	0.743	0.35	0.74	0.187	0.664	63.16	6.26
	R^2^	0.71	0.72	0.6	0.73	0.472	0.714		
ROH > 10 Mb	Intercept	0.0009	0.004	0.01	0.002	0.019	0.01	90.80	78.06
	Slope	0.388	0.48	0.02	0.427	0.001	0.25	160.02	31.23
	R^2^	0.52	0.55	0.35	0.42	0.01	0.424		

*Regression was obtained for three different QC datasets (strict: 18,198 SNP, medium: 22,615 and relaxed: 25,511), allowing none or 1 heterozygous SNP (het) per ROH and different ROH sizes cut-off (1, 2, 4 and 10 Mb). Coefficients of variation (CV, in %) are given in the last two columns.*

**FIGURE 1:**
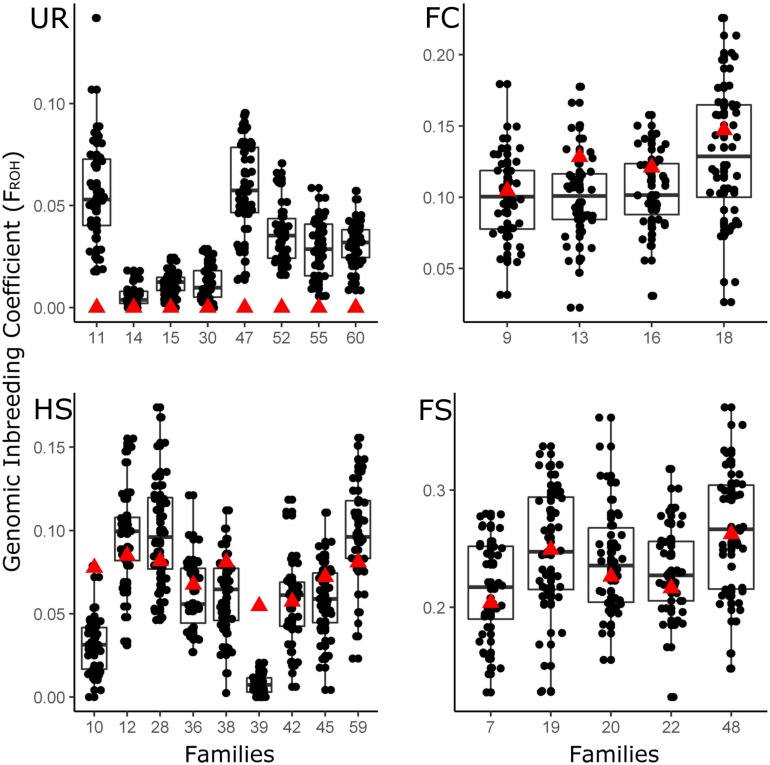
Genomic inbreeding estimated through ROH (F_ROH_). F_ROH_ (*y*-axis) was obtained using a 1 Mb ROH cut-off for each family. Family codes are presented in *x*-axis and grouped by their parental kinship coefficients θ_IBD_ as follows: UR; unrelated (0.0076 < θ_IBD_), FC; first cousin (0.038 < θ_IBD_ < 0.0937), HS; half-sibling (0.0937 < θ_IBD_ < 0.1872) and FS; full-sibling (θ_IBD_ > 0.1872). Red triangles represent the θ_IBD_ of each family’s parents.

However, there is not a perfect correlation between θ_IBD_ and F_ROH_, the coefficient of variation of F_ROH_ being higher when considering non-inbred families (0 < θ_IBD_ < 0.0076; Supplementary Table 5). Moreover, when comparing the F_ROH_ and the θ_IBD_ interval (considering the medium QC criterion and 1 Het), non-inbred families showed higher differences compared with more inbred ones. Thus, when non-inbred families are considered (Figure 1), the θ_IBD_ estimates of each family’s parents show extremely higher differences with the median of the F_ROH_ box-plot than in more inbred families (Figure 1). The discrepancies found among non-inbred families might be related to the misclassification of some parents as fully unrelated, when a certain degree of ancestral consanguinity could occur in the domestic broodstock (do Prado et al., 2018a). Even with these differences, it is possible to conclude that the F_ROH_ (for ROH > 1 Mb) of the offspring correlates the best (*R*^2^ =0.78) with their parent’s θ_IBD_ when using the medium QC criterion, a PLINK’s sliding window of 37 SNP and 1 heterozygous SNP allowed.

### Genomic Distribution of ROH and ROHi

**FIGURE 2:**
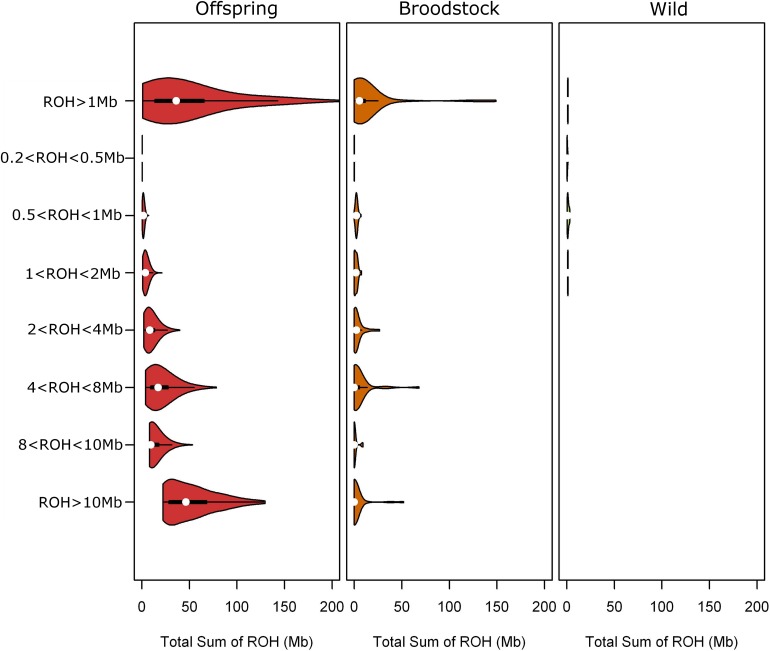
Runs of homozygosity sum distribution for each ROH size category in the domestic and wild populations. ROH < 1 Mb were not considered for F_ROH_ estimation.

Large differences were found in ROH distribution between the wild and domestic turbot populations either considering the parental broodstock or additionally including all offspring. This is in agreement with the presence of inbred breeders in the broodstock according to kinship estimates (Figure 2). In the wild population, nearly no ROH were found among the size categories considered and, in fact, ROH longer than 2 Mb were not found at all (Figure 2). As expected, due to low ROH-identification, ROHi were not detected in the wild population. It would be interesting to explore smaller ROH segments (<1 Mb), to complement ZHp analysis in this population. Ideally this should be carried out using the same SNP panel in both the wild and farm populations and if possible, a higher density SNP panel, which is not the case for our study. These are the expected results in a large panmictic population such as Atlantic turbot (do Prado et al., 2018b). Higher marker density would be needed to identify shorter ROH that are IBD, ideally through whole-genome re-sequencing (Marras et al., 2014).

In the broodstock population, an important number of ROH were identified, especially with sizes below 1 Mb (which were not considered for F_ROH_ estimation), followed by 4 < ROH < 8 Mb (Figure 2). An atypical amount of medium size-ROH (4−8 Mb) was detected across turbot families, particularly in full-siblings. This observation makes sense if one considers the recent history of turbot aquaculture. Assuming that ROH length correlates with the number of generations elapsed since the common ancestor (Howrigan et al., 2011; Marras et al., 2014), the over-representation of 4−8 Mb ROH in turbot would indicate ∼7−4 generations assuming the 0.6 Mb/cM specifically reported for turbot (Bouza et al., 2012; Maroso et al., 2018). Accordingly, these genomic signals might be related to the duration of turbot breeding programs (five generations of selection; initiated 15 years ago), but also to previous domestication phases (going back up to 10 generations ago; 30 years; Martínez et al., 2016).

**FIGURE 3:**
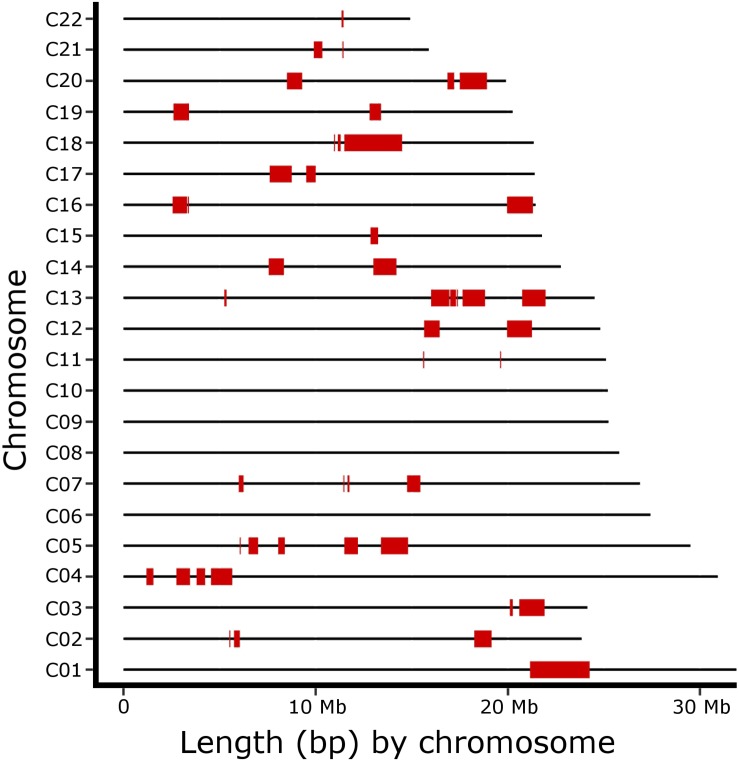
Runs of homozygosity islands genome distribution in the broodstock population. Each ROH island (ROHi) is represented by red segments across turbot (*S*. *maximus*) chromosomes.

Furthermore, 49 ROHi were identified in the broodstock population (Figure 3 and Supplementary Table 6), distributed across all chromosomes excluding C06, C08, C09 and C10. ROH sizes ranged from 0.05 (several chromosomes) to 3.1 (C01) Mb, being 0.65 Mb on average. The longest ROHi were found in C01 (3.1 Mb), C18 (3 Mb), C20 and C05 (1,4 Mb both). When chromosome length was taken into account, ROH percentage coverage ranged from 0.17 to 14.05 %; the longest ROH were found in C18 (14.05 %), C01 (9.72 %), C20 (7.03 %), and C16 (6.29 %) (Supplementary Table 6).

The genomic distribution of ROHi might be useful to pinpoint candidate genomic regions under selection, as a preliminary step to search for candidate genes related to productive traits using genome mining (Ku et al., 2011; Goszczynski et al., 2018). The uneven distribution of ROHi observed across the turbot genome supports this idea and clustering of small to large ROHi were detected at particular regions of chromosomes C01, C03, C04, C13, C18, among others. In some cases, various consecutive ROH islands appeared interrupted by small genome stretches (e.g., on chromosome C13), which could suggest interleaved homozygous haplotype deficiency regions associated with deleterious/lethal haplotypes (Pausch et al., 2015). A significant occurrence of lethal recessive alleles was previously reported by Martínez et al. (2008) using diploid gynogenetic families. In the broodstock population, ROH can be further used to investigate genome signatures of selection, contributing to dissect the genetic architecture of quantitative traits and to map genes of interest for breeders. In turbot, the differences observed in ROHi-distribution could point to genomic regions closely linked to genes mainly under selection for growth rate; as these regions tend to exhibit ROHi and lower genetic diversity than the average genome (Mastrangelo et al., 2017). The ROHi distribution might also be associated with the recombination rate landscape across the genome, since ROH hotspots might be associated with genomic regions with low recombination rate. By contrast, ROH are scarcely expected in genomic regions associated with higher fitness (Mastrangelo et al., 2017), such as those harboring genes involved in immune robustness, which have been reported to exhibit high genetic diversity in wild populations (e.g., major histocompatibility complex; Worley et al., 2010; Sutton et al., 2011).

### Genetic Diversity and Selective Sweeps

Genome-wide genetic variation within and between populations is the raw material for evolutionary change, selective breeding and sustainable management of genetic resources (Akagi et al., 2016; Brito et al., 2017). The genomic screening in our study rendered 20,823 and 24,889 37-SNP-wide windows for the domestic broodstock and the wild population, respectively. Average Hp was higher in the domestic broodstock (0.27; range: 0.14–0.38) than in the wild population (0.19; range; 0.07–0.31). This is a rather unexpected outcome considering the processes of genetic drift associated with domestic populations, that likely has to do with the different RADseq-derived SNP panels used in both populations. The fraction of loci with rare alleles (MAF (minimum allele frequency) < 0.05) was much lower in the broodstock than in the wild SNP panels (29.62 vs. 53.46%, respectively; Supplementary Figure 4), thus strongly lowering heterozygosity estimates in the wild population. Despite limitations due to the ascertainment bias expected for different SNP panels, the loss of rare alleles in the broodstock sample would agree with genetic drift processes associated with founder effects and breeding practices on the finite turbot broodstock (Luikart et al., 1998; do Prado et al., 2018a; Maroso et al., 2018).

**FIGURE 4:**
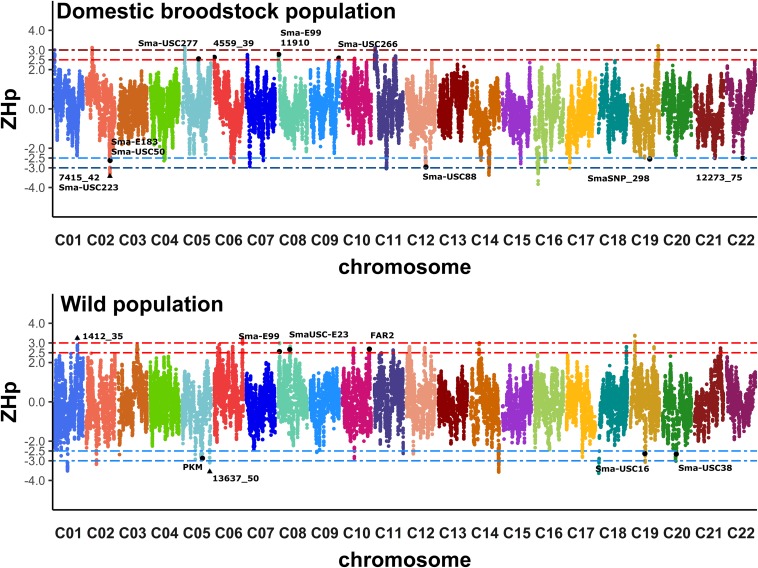
Genome-wide distribution of Z-transformed pooled heterozygosity (ZHp) in the broodstock and wild populations. ZHp estimates were obtained using 37-SNP sliding windows (colored dots) across turbot chromosomes. ZHp thresholds are represented as dotted horizontal lines (ZHp > 2.5/3.0, red; ZHp < –2.5/3.0; blue). Relevant markers included inside strict low and/or high genetic diversity regions are displayed as black triangles; those inside suggestive low and/or high genetic diversity regions are displayed as black dots.

The less strict ZHp criteria (ZHp ≥ 2.5 and ZHp ≤ −2.5) represented about 1.28% (319 windows) and 1.34% (278 windows) of the total sum of windows in the wild and broodstock populations, respectively (Supplementary Figure 5). Windows surpassing the ZHp threshold of 2.5 were cataloged as suggestive high-genetic diversity (high-GD) regions, whereas those with ZHp below −2.5 were considered as suggestive low-GD regions. Both suggestive high-GD and low-GD regions were unevenly distributed across the turbot genome: as such, some chromosomes did not harbor any retained windows (chromosomes C03 and C20 in the broodstock; chromosomes C04, C07, C13, C15, C16 and C22 in wild turbot; Figure 4 and Supplementary Figure 6).

More restrictive ZHp criteria (ZHp ≥ 3.0 and ZHp ≤ −3.0) represented about 0.31% (78 windows) and 0.26% (54 windows) of the total number of windows in the wild and broodstock populations, respectively (Supplementary Figure 5). Windows surpassing the ZHp threshold of 3.0 were cataloged as significant high-GD regions, whereas those with ZHp below −3.0 were considered as significant low-GD regions. Both significant high-GD and low-GD regions were unevenly distributed across chromosomes in the turbot genome in the broodstock (C01, C02, C05, C11, C12, C14, C16, C17, and C19) and in the wild populations (C01, C02, C05, C06, C14, C18, C19, and C20).

Genomic regions in the vicinity of loci subjected to directional selection showed specific signatures that facilitate the identification of selective sweeps (Sun et al., 2014; Brito et al., 2017; Zwane et al., 2019). Conversely, high genetic diversity has been reported in genomic regions associated with fitness, such as those harboring loci involved in immune robustness (Mastrangelo et al., 2017). The genome-wide scanning of the turbot genome revealed regions with higher and lower genetic diversity (GD) than the average across the genome. Forty five and 71 suggestive (less strict ZHp threshold) windows in the wild and broodstock populations, respectively, matched to 8 (chromosomes C01, C05, C08, C10, C19, and C20) and 12 (chromosomes C02, C05, C06, C08, C09, C12, C19, and C22) respective genomic regions including functional markers (QTL and/or adaptive outlier loci). One marker in chromosome C08 (Sma-E99) was found in a high-GD region in both the domestic and wild populations (Figure 4). These data targeted a total of 19 candidate genomic regions with low-GD and high-GD, suggestive to be under selection, which harbor QTL-associated markers for productive traits related to breeding programs, and/or outlier loci related to adaptive variation in wild turbot (Figure 4 and Table 2). Among the selected significant windows under strict ZHp criteria, 13 wild and 11 broodstock windows matched to two (chromosomes C01 and C05) and one (chromosome C02) genomic regions including functional markers. These data targeted three candidate genomic regions showing low- and high-GD that harbored QTL-associated markers for productive traits related to breeding programs and/or outlier loci related to adaptive variation, thus likely to be under selective pressure (Figure 4 and Table 2).

**TABLE 2:** List of QTL-associated markers and outliers located in suggestive high-GD and low-GD regions for the broodstock and wild populations.

Population	Category	Chrom	ID	Position (bp)	Associated traits/Selection signatures
Broodstock	High-GD	C05	Sma-USC277^[5]^	16721340	Resistance and Survival time to *Philasterides dicentrarchi*; Resistance to *Aeromonas salmonicida*
		C06	4559_39^[2]^	1109461	Genetic divergence between farm (origin ORI1) and wild turbot
		C08	11910^[1]^	833346	Balancing selection; Global outlier
		C08	Sma-E99^[1,10]^	1047506	Divergent selection
		C09	Sma-USC266^[8]^	23265541	Growth
	Low-GD	C02	**Sma-USC223^[7,8]^**	18889875	Growth (Body weight and Body length)
		C02	Sma-USC50^[7,8]^	19267847	Growth (Body weight and Body length)
		C02	Sma-E183^[8]^	19293723	Growth (Body weight)
		C02	**7415_42^[1]^**	18430391	Balancing selection; Divergence Baltic Sea and Black Sea vs. Atlantic turbot
		C12	Sma-USC88^[6]^	16323329	Survival time to VHSV
		C19	SmaSNP_298^[1,10]^	13228742	Divergent selection
		C22	12273_75^[2]^	8140606	Genetic divergence between farm (origin ORI1) and wild turbot
Wild	High-GD	C01	**1412_35^[2]^**	25668281	Genetic divergence between farm (origin ORI2) and wild turbot
		C08	Sma-E99^[1,10]^	1047506	Divergent selection
		C08	SmaUSC-E23^[4,5]^	9946986	Resistance and Survival time to *Philasterides dicentrarchi*; Resistance to *Aeromonas salmonicida*
		C10	FAR2^[3]^	23144173	Positional gene marker; growth QTL
	Low-GD	C05	**13637_50^[2]^**	27533562	Genetic divergence between farm (origin ORI1) and wild turbot
		C05	PKM^[3]^	19976976	Positional gene marker; growth QTL
		C19	Sma-USC16^[8]^	9911085	Growth (Body weight, Body length and condition factor)
		C20	Sma-USC38^[1,5,9]^	10007284	Stabilizing selection; Resistance and Survival time to *Philasterides dicentrarchi*

*Information about the marker ID, genetic diversity (GD) region they are found in (High-GD, ZHp > 2.5; low-GD, ZHp < −2.5), position in the chromosome and traits they are associated with are presented. Markers in bold were found in selected high-/low-GD regions (ZHp > 3.0; ZHp < −3.0). Sma-E99 (underlined) was detected in both populations in a high-GD region. [1] do Prado et al. (2018a); [2] do Prado et al. (2018b); [3] Robledo et al. (2016); [4] Rodríguez-Ramilo et al. (2011); [5] Rodríguez-Ramilo et al. (2013); [6] Rodríguez-Ramilo et al. (2014); [7] Sánchez-Molano et al. (2011); [8] Sciara et al. (2018); [9] Vilas et al. (2010); [10] Vilas et al. (2015).*

### Integrative Genome Atlas of Genetic Diversity and ROHi

An integrative genome atlas of GD in the broodstock, including the ROHi and low/high-GD regions detected, was developed in this study. Different genomic stretches were highlighted due to the presence of low-/high-GD, ROHi and/or QTL/adaptive-associated markers. Another atlas for low-/high-GD regions in the wild turbot population was also obtained, although no ROHi were found. Both genomic atlases were anchored to the turbot genome sequence (Maroso et al., 2018) and are shown in Supplementary Figure 7. These data represent useful landmarks to identify, prioritize and investigate putative signatures of selection in the species as a basis for further gene-specific research. In this sense, the application of genome mining and functional enrichment analyses can be useful for uncovering of candidate genes and pathways underlying suggestive genomic regions subject to selection.

In the broodstock population, a significant low-GD region in C02 was associated with a ROHi and a growth-related QTL marker (Sma-USC223; Sánchez-Molano et al., 2011; Sciara et al., 2018). This region was expanded under a less restrictive ZHp threshold and harbored other closely linked growth-related QTL markers (Sma-USC50 and Sma-E183). These three markers were tightly linked to important growth-related genes, such as *fgf6* and *lep*, and their association with growth was further validated in families from different turbot breeding programs (Sciara et al., 2018). Moreover, another locus linked to balancing selection between Baltic and Black Sea population, consistent with parallel adaptation to salinity (7415_42, do Prado et al., 2018b), was also identified within this significant low-GD region at C02. Some suggestive low-GD regions detected under a less restrictive ZHp threshold were also pinpointed by the positional mapping of functional markers in the broodstock population. Among them, a suggestive low-GD region overlapping with a ROHi and a QTL-associated marker for *P. dicentrarichii* resistance in turbot (Sma-USC38) was identified on C20. Functional enrichment in immune-related genes, such as *tfe3*, *mst1r*, *myb* and *fos* was previously documented (Rodríguez-Ramilo et al., 2013; Figueras et al., 2016). Another interesting and previously unexplored region with ROHi and low-GD stretches was detected in the broodstock population. This region, located in chromosome C07 (Supplementary Figure 7), was functionally enriched in terms and pathways associated with the membrane attack complex, complement activation and regulation, and leukocyte/lymphocyte-mediated immunity. Two particularly relevant immune-related genes were mined in this region: *c8a* and *c8b*; coding for the complement component C8 alpha and beta chains, respectively. C8 is a key component of the complement cascade which participates in the formation of the Membrane Attack Complex and directly interacts with pathogens as part of innate immune pathways, thus, likely under pathogen-induced selective pressure (Webb et al., 2015). Finally, one suggestive high-GD region associated with an adaptive-marker, which had been poorly studied to date, was mined in the turbot genome to explore underlying candidate genes and pathways. This high-GD region is located at C06 in the broodstock population (Supplementary Figure 7) and linked to a domestication-associated marker in this species (4559_39; do Prado et al., 2018a). The region showed functional enrichment suggesting a role in response to hypoxia (e.g., *ahcy* and *angpt4* genes linked to methylation regulation and angiogenesis, respectively) and phosphate compound metabolic processes (e.g., *alpl* involved in bone mineralization and linked to skeletal defects). Hypoxia tolerance, when interacting with water temperature and salinity, has a major impact on fish physiology, including growth and immune response in aquaculture conditions (Lund et al., 2017), and has been associated with selection signatures in the channel catfish (Sun et al., 2014). Genome mining of this region also revealed other immune-related genes, such as *ifsf21a*, which belongs to a family of membrane receptors, and *il17rd*, which encodes a component of the interleukin receptor 17 and associated with immune response against bacterial pathogens in fish mucosal tissues (Wang et al., 2014; Ding et al., 2016).

In the wild population, the two detected regions under strict ZHp criteria included relevant functional markers that were associated with genetic differentiation between farmed (two origins, ORI1 and ORI2) and wild turbot populations (do Prado et al., 2018a). One marker (1412_35; ORI2) was identified in a high-GD region at chromosome C01: This region harbors some key genes associated with the TCA cycle, such as *sdha* and *mdh2*. *Sdha* encodes a major catalytic subunit of succinate-ubiquinone oxidoreductase and has been previously suggested as a gene under long-term balancing selection/heterosis in humans (Baysal et al., 2007). *Mdh2* catalyses the reversible oxidation of malate to oxaloacetate and has been described in *Drosophila* as an example of balancing selection (Peng et al., 1991). The other marker (13637_50; ORI1) was identified in a low-GD region at chromosome C05, harboring genes like *stard5, trappc3* and *txlng* involved in intracellular traffic and regulation of cellular cycle, the latter one (taxilin gamma) being identified as a target of natural selection in human and Great Apes (Zhao et al., 2019). An interesting suggestive high-GD region at chromosome C08 was identified in the wild population under a less restrictive ZHp threshold (Figueras et al., 2016). This region harbors a relevant functional marker (SmaUSC-E23) closely linked to several immune-related genes and was associated with resistance to major pathogen threats for turbot production (*Aeromonas salmonicida* and *Philasterides dicentrarichii*; Rodríguez-Ramilo et al., 2011, 2013; Martínez et al., 2016).

## Conclusion

Two SNP datasets with similar marker density from a domestic and a wild turbot population were used to characterize the genome-wide distribution of runs of homozygosity (ROH) and genetic diversity (GD). After fitting ROH-calling parameters, large differences were found in ROH distribution between the broodstock and wild turbot populations, bringing insights into the history of domestication and selection in this species. The study provides useful information to estimate genomic inbreeding and identify selective sweeps in turbot. Genomic signatures of selection were identified by checking the overlapping distribution of ROHi and extreme genetic diversity regions across the genome, along with the presence of functional markers associated with QTL or adaptive variability in turbot. Genome mining and functional analysis on some selected regions provided candidate genes and pathways potentially explaining the action of selection in turbot populations. The atlases of genome diversity in broodstock and wild turbot populations in this study represent useful information for implementing further genomic research and breeding applications in turbot.

## Data Availability Statement

All datasets generated for this study are included in the Supplementary Material.

## Ethics Statement

The animal experimental procedures carried out in this study were approved and conducted in strict accordance with the Directive 2010/63/EU of the European Parliament and of the Council of 22 September 2010 on the protection of animals used for scientific purposes.

## Author Contributions

OA participated in data collection, data analysis and interpretation, and drafting the manucript. FC participated in data analysis and interpretation. AC participated in data collection, data analysis and interpretation. AL, JH-H, and DB participated in data collection and critical revision of the manucript. CB and PM participated in the conception, drafting and critical revision of the manucript.

## Conflict of Interest

The authors declare that the research was conducted in the absence of any commercial or financial relationships that could be construed as a potential conflict of interest.

## References

[B1] Akagi T. Hanada T. Yaegaki H. Gradziel T. M. Tao R. (2016). Genome-wide view of genetic diversity reveals paths of selection and cultivar differentiation in peach domestication. *DNA Res.* 23 271–282. 10.1093/dnares/dsw014 27085183 PMC4909313

[B2] Anacleto O. Cabaleiro S. Villanueva B. Saura M. Houston R. D. Woolliams J. A. (2019). Genetic differences in host infectivity affect disease spread and survival in epidemics. *Sci. Rep.* 9:4924. 10.1038/s41598-019-40567-w 30894567 PMC6426847

[B3] Barson N. J. Aykanat T. Hindar K. Baranski M. Bolstad G. H. Fiske P. (2015). Sex-dependent dominance at a single locus maintains variation in age at maturity in salmon. *Nature* 528 405–408. 10.1038/nature16062 26536110

[B4] Baysal B. E. Lawrence E. C. Ferrell R. E. (2007). Sequence variation in human succinate dehydrogenase genes: evidence for long-term balancing selection on SDHA. *BMC Biol.* 5:12. 10.1186/1741-7007-5-12 17376234 PMC1852088

[B5] Bolger A. M. Lohse M. Usadel B. (2014). Trimmomatic: a flexible trimmer for Illumina sequence data. *Bioinformatics* 30 2114–2120. 10.1093/bioinformatics/btu170 24695404 PMC4103590

[B6] Bonhomme M. Boitard S. San Clemente H. Dumas B. Young N. Jacquet C. (2015). Genomic signature of selective sweeps illuminates adaptation of *Medicago truncatula* to root-associated microorganisms. *Mol. Biol. Evol.* 32 2097–2110. 10.1093/molbev/msv092 25901015 PMC4833077

[B7] Bouza C. Hermida M. Pardo B. G. Fernández C. Fortes G. G. Castro J. (2007). A microsatellite genetic map of the turbot (*Scophthalmus maximus*). *Genetics* 177 2457–2467. 10.1534/genetics.107.075416 18073440 PMC2219478

[B8] Bouza C. Hermida M. Pardo B. G. Vera M. Fernández C. de la Herrán R. (2012). An Expressed Sequence Tag (EST)-enriched genetic map of turbot (S. maximus): a useful framework for comparative genomics across model and farmed teleosts. *BMC Genet.* 13:54. 10.1186/1471-2156-13-54 22747677 PMC3464660

[B9] Brito L. F. Kijas J. W. Ventura R. V. Sargolzaei M. Porto-Neto L. R. Cánovas A. (2017). Genetic diversity and signatures of selection in various goat breeds revealed by genome-wide SNP markers. *BMC Genomics* 18:229. 10.1186/s12864-017-3610-0 28288562 PMC5348779

[B10] Cano J. M. Matsuba C. Mäkinen H. Merilä J. (2006). The utility of QTL-Linked markers to detect selective sweeps in natural populations – a case study of the EDA gene and a linked marker in threespine stickleback. *Mol. Ecol.* 15 4613–4621. 10.1111/j.1365-294X.2006.03099.x 17107487

[B11] Catchen J. Hohenlohe P. A. Bassham S. Amores A. Cresko W. A. (2013). Stacks: an analysis tool set for population genomics. *Mol. Ecol.* 22 3124–3140. 10.1111/mec.12354 23701397 PMC3936987

[B12] Ceballos F. C. Hazelhurst S. Ramsay M. (2018a). Assessing runs of Homozygosity: a comparison of SNP Array and whole genome sequence low coverage data. *BMC Genom.* 19:106. 10.1186/s12864-018-4489-0 29378520 PMC5789638

[B13] Ceballos F. C. Joshi P. K. Clark D. W. Ramsay M. Wilson J. F. (2018b). Runs of homozygosity: windows into population history and trait architecture. *Nat. Rev. Genet.* 19 220–234. 10.1038/nrg.2017.109 29335644

[B14] Danecek P. Auton A. Abecasis G. Albers C. A. Banks E. DePristo M. A. (2011). The variant call format and VCFtools. *Bioinformatics* 27 2156–2158. 10.1093/bioinformatics/btr330 21653522 PMC3137218

[B15] Ding Y. Ai C. Mu Y. Ao J. Chen X. (2016). Molecular characterization and evolution analysis of five interleukin-17 receptor genes in large yellow croaker *Larimichthys crocea*. *Fish Shellfish Immunol.* 58 332–339. 10.1016/j.fsi.2016.09.017 27633682

[B16] do Prado F. D. Vera M. Hermida M. Bouza C. Maes G. E. Volckaert F. A. M. (2018a). Tracing the genetic impact of farmed turbot *Scophthalmus maximus* on wild populations. *Aquac. Environ. Interact.* 10 447–463. 10.3354/aei00282

[B17] do Prado F. D. Vera M. Hermida M. Bouza C. Pardo B. G. Vilas R. (2018b). Parallel evolution and adaptation to environmental factors in a marine flatfish: implications for fisheries and aquaculture management of the turbot (*Scophthalmus maximus*). *Evol. Appl.* 11 1322–1341. 10.1111/eva.12628 30151043 PMC6099829

[B18] Ferenčaković M. Sölkner J. Kapš M. Curik I. (2017). Genome-wide mapping and estimation of inbreeding depression of semen quality traits in a cattle population. *J. Dairy Sci.* 100 4721–4730. 10.3168/jds.2016-12164 28434751

[B19] Figueras A. Robledo D. Corvelo A. Hermida M. Pereiro P. Rubiolo J. A. (2016). Whole genome sequencing of turbot (*Scophthalmus maximus*; *Pleuronectiformes*): a fish adapted to demersal life. *DNA Res.* 23 181–192. 10.1093/dnares/dsw007 26951068 PMC4909306

[B20] Goszczynski D. Molina A. Terán E. Morales-Durand H. Ross P. Cheng H. (2018). Runs of homozygosity in a selected cattle population with extremely inbred bulls: descriptive and functional analyses revealed highly variable patterns. *PLoS One* 13:e0200069. 10.1371/journal.pone.0200069 29985951 PMC6037354

[B21] Gutiérrez-Gil B. Arranz J. J. Wiener P. (2015). An interpretive review of selective sweep studies in *Bos taurus* cattle populations: identification of unique and shared selection signals across breeds. *Front. Genet.* 6:167. 10.3389/fgene.2015.00167 26029239 PMC4429627

[B22] Hermida M. Bouza C. Fernández C. Sciara A. A. Rodríguez-Ramilo S. T. Fernández J. (2013). Compilation of mapping resources in turbot (*Scophthalmus maximus*): a new integrated consensus genetic map. *Aquaculture* 414-415 19–25. 10.1016/j.aquaculture.2013.07.040

[B23] Howrigan D. P. Simonson M. A. Keller M. C. (2011). Detecting autozygosity through runs of homozygosity: a comparison of three autozygosity detection algorithms. *BMC Genomics* 12:460. 10.1186/1471-2164-12-460 21943305 PMC3188534

[B24] Huang da W. Sherman B. T. Lempicki R. A. (2009a). Bioinformatics enrichment tools: paths toward the comprehensive functional analysis of large gene lists. *Nucleic Acids Res.* 37 1–13. 10.1093/nar/gkn923 19033363 PMC2615629

[B25] Huang da W. Sherman B. T. Lempicki R. A. (2009b). Systematic and integrative analysis of large gene lists using DAVID bioinformatics resources. *Nat. Protoc.* 4 44–57. 10.1038/nprot.2008.211 19131956

[B26] Johansson A. M. Pettersson M. E. Siegel P. B. Carlborg O. (2010). Genome-wide effects of long-term divergent selection. *PLoS Genet.* 6:e1001188. 10.1371/journal.pgen.1001188 21079680 PMC2973821

[B27] Jones F. C. Grabherr M. G. Chan Y. F. Russell P. Mauceli E. Johnson J. (2012). The genomic basis of adaptive evolution in threespine sticklebacks. *Nature* 484 55–61. 10.1038/nature10944 22481358 PMC3322419

[B28] Joshi P. K. Esko T. Mattsson H. Eklund N. Gandin I. Nutile T. (2015). Directional dominance on stature and cognition in diverse human populations. *Nature* 532 459–462. 10.1038/nature14618 26131930 PMC4516141

[B29] Kardos M. Taylor H. R. Ellegren H. Luikart G. Allendorf F. W. (2016). Genomic advances the study of inbreeding depression in the wild. *Evol. Appl.* 9 1205–1218. 10.1111/eva12414 27877200 PMC5108213

[B30] Kirin M. McQuillan R. Franklin C. S. Campbell H. McKeigue P. M. Wilson J. F. (2010). Genomic runs of homozygosity record population history and consanguinity. *PLoS One* 5:e13996. 10.1371/journal.pone.0013996 21085596 PMC2981575

[B31] Ku C. S. Naidoo N. Teo S. M. Pawitan Y. (2011). Regions of homozygosity and their impact on complex diseases and traits. *Hum. Genet.* 129 1–15. 10.1007/s00439-010-0920-6 21104274

[B32] Langmead B. Salzberg S. L. (2012). Fast gapped-read alignment with Bowtie 2. *Nat. Methods* 9 357–359. 10.1038/nmeth.1923 22388286 PMC3322381

[B33] Langmead B. Trapnell C. Pop M. Salzberg S. L. (2009). Ultrafast and memory-efficient alignment of short DNA sequences to the human genome. *Genome Biol.* 10:R25. 10.1186/gb-2009-10-3-r25 19261174 PMC2690996

[B34] Luikart G. Allendorf F. W. Cornuet J. M. Sherwin W. B. (1998). Distortion of allele frequency distributions provides a test for recent population bottlenecks. *J. Hered.* 89 238–247. 10.1093/jhered/89.3.238 9656466

[B35] Lund M. Krudtaa Dahle M. Timmerhaus G. Alarcon M. Powell M. Aspehaug V. (2017). Hypoxia tolerance and responses to hypoxic stress during heart and skeletal muscle inflammation in Atlantic salmon (*Salmo salar*). *PLoS One* 12:e0181109. 10.1371/journal.pone.0181109 28700748 PMC5507449

[B36] Maroso F. Hermida M. Millán A. Blanco A. Saura M. Fernández A. (2018). Highly dense linkage maps from 31 full-sibling families of turbot (*Scophthalmus maximus*) provide insights into recombination patterns and chromosome rearrangements throughout a newly refined genome assembly. *DNA Res.* 25 439–450. 10.1093/dnares/dsy015 29897548 PMC6105115

[B37] Marras G. Gaspa G. Sorbolini S. Dimauro C. Ajmone-Marsan P. Valentini A. (2014). Analysis of runs of homozygosity and their relationship with inbreeding in five cattle breeds farmed in Italy. *Anim. Genet.* 46 110–121. 10.1111/age.12259 25530322

[B38] Martínez P. Bouza C. Hermida M. Fernández J. Toro M. A. Vera M. (2009). Identification of the major sex-determining region of turbot (*Scophthalmus maximus*). *Genet. Soc. Am.* 183 1443–1452. 10.1534/genetics.109.107979 19786621 PMC2787431

[B39] Martínez P. Hermida M. Pardo B. G. Fernández C. Castro J. Cal R. M. (2008). Centromere-linkage in the turbot (*Scophthalmus maximus*) throught half-tetrad analysis in diploid meiogynogenetics. *Aquaculture* 280 81–88. 10.1016/j.aquaculture.2008.05.011

[B40] Martínez P. Robledo D. Rodríguez-Ramilo S. T. Hermida M. Taboada X. Pereiro P. (2016). Turbot (*Scophthalmus maximus*) genomic resources: application for boosting aquaculture production. *Genomics Aquac.* 2016 131–163. 10.1016/B978-0-12-801418-9.00006-8

[B41] Mastrangelo S. Tolone M. Sardina M. T. Sottile G. Sutera A. M. Di Gerlando R. (2017). Genome-wide scan for runs of homozygosity identifies potential candidate genes associated with local adaptation in Valle del Belice sheep. *Genet. Select. Evol.* 49:84. 10.1186/s12711-017-0360-z 29137622 PMC5684758

[B42] McQuillan R. Leutenegger A. L. Abdel-Rahman R. Franklin C. S. Pericic M. Barac-Lauc L. (2008). Runs of homozygosity in European populations. *Am. J. Hum. Genet.* 83 359–372. 10.1016/j.ajhg.2008.08.007 18760389 PMC2556426

[B43] Messer P. W. Petrov D. A. (2013). Population genomics of rapid adaptation by soft selective sweeps. *Trends Ecol. Evol.* 28 659–669. 10.1016/j.tree.2013.08.003 24075201 PMC3834262

[B44] Metzger J. Karwath M. Tonda R. Beltran S. Águeda L. Gut M. (2015). Runs of homozygosity reveal signatures of positive selection for reproduction traits in breed and non-breed horses. *BMC Genomics* 16:764. 10.1186/s12864-015-1977-3 26452642 PMC4600213

[B45] Moradi M. H. Nejati-Javaremi A. Moradi-Shahrbabak M. Dodds K. G. McEwan J. C. (2012). Genomic scan of selective sweeps in thin and fat tail sheep breeds for identifying of candidate regions associated with fat deposition. *BMC Genet.* 13:10. 10.1186/1471-2156-13-10 22364287 PMC3351017

[B46] Nalls M. A. Guerreiro R. J. Simon-Sánchez J. Bras J. T. Traynor B. J. Gibbs J. R. (2009). Extended tracts of homozygosity identify novel candidate genes associated with late onset Alzheimer’s disease. *Neurogenetics* 10 183–190. 10.1007/s10048-009-0182-4 19271249 PMC2908484

[B47] Pausch H. Schwarzenbacher H. Burgstaller J. Flisikowski K. Wurmser C. Jansen S. (2015). Homozygous haplotype deficiency reveals deleterious mutations compromising reproductive and rearing success in cattle. *BMC Genomics* 16:312. 10.1186/s12864-015-1483-7 25927203 PMC4403906

[B48] Peng T. X. Moya A. Ayala F. J. (1991). Two modes of balancing selection in *Drosophila melanogaster*: overcompensation and overdominance. *Genetics* 128 381–391. 1906418 10.1093/genetics/128.2.381 PMC1204475

[B49] Peripolli E. Munari D. P. Silva M. V. G. B. Lima A. L. F. Irgang R. Baldi F. (2016). Runs of homozygosity: current knowledge and applications in livestock. *Anim. Genet.* 48 255–271. 10.1111/age.12526 27910110

[B50] Poland J. A. Rife T. (2012). Genotyping-by-sequencing for plant breeding and genetics. *Plant Genome J.* 5 92–102. 10.3835/plantgenome2012.05.0005

[B51] Purcell S. Neale B. Todd-Brown K. Thomas L. Ferreira M. A. Bender D. (2007). PLINK: a tool set for whole-genome association and population-based linkage analyses. *Am. J. Hum. Genet.* 81 559–575. 10.1086/519795 17701901 PMC1950838

[B52] Purfield D. C. McParland S. Wall E. Berry D. P. (2017). The distribution of runs of homozygosity and selection signatures in six commercial meat sheep breeds. *PLoS One* 12:e0176780. 10.1371/journal.pone.0176780 28463982 PMC5413029

[B53] Qanbari S. Strom T. M. Haberer G. Weigend S. Gheyas A. A. Turner F. (2012). A high resolution genome-wide scan for significant selective sweeps: an application to pooled sequence data in laying chickens. *PLoS One* 7:e49525. 10.1371/journal.pone.0049525 23209582 PMC3510216

[B54] Quilez J. Short A. D. Martínez V. Kennedy L. J. Ollier W. Sánchez A. (2011). A selective sweep of >8 Mb on chromosome 26 in the Boxer genome. *BMC Genomics* 12:339. 10.1186/1471-2164-12-339 21722374 PMC3152542

[B55] Ramey H. R. Decker J. E. McKay S. D. Rolf M. M. Schnabel R. D. Taylor J. F. (2013). Detection of selective sweeps in cattle using genome-wide SNP data. *BMC Genomics* 14:382. 10.1186/1471-2164-14-382 23758707 PMC3681554

[B56] Robledo D. Fernández C. Hermida M. Sciara A. Álvarez-Dios J. A. Cabaleiro S. (2016). Integrative transcriptome, genome and quantitative trait loci resources identify single nucleotide polymorphisms in candidate genes for growth traits in turbot. *Int. J. Mol. Sci.* 17:243. 10.3390/ijms17020243 26901189 PMC4783974

[B57] Rodríguez-Ramilo S. T. de la Herrán R. Ruiz-Rejón C. Hermida M. Fernández C. Pereiro P. (2014). Identification of quantitative trait *Loci* associated with resistance to viral haemorrhagic septicaemia (VHS) in turbot (*Scophthalmus maximus*): a comparison between bacterium, parasite and virus diseases. *Mar. Biotechnol.* 16 265–276. 10.1007/s10126-013-9544-x 24078233

[B58] Rodríguez-Ramilo S. T. Fernández J. Toro M. A. Bouza C. Hermida M. Fernández C. (2013). Uncovering QTL for resistance and survival time to *Philasterides dicentrarchi* in turbot (*Scophthalmus maximus*). *Anim. Genet.* 44 149–157. 10.1111/j.1365-2052.2012.02385.x 22690723

[B59] Rodríguez-Ramilo S. T. Toro M. A. Bouza C. Hermida M. Pardo B. G. Cabaleiro S. (2011). QTL detection for *Aeromonas salmonicida* resistance related traits in turbot (*Scophthalmus maximus*). *BMC Genomics* 12:541. 10.1186/1471-2164-12-541 22047500 PMC3216323

[B60] Rubin C. J. Megens H. J. Martinez-Barrio A. Maqbool K. Sayyab S. Schwochow D. (2012). Strong signatures of selection in the domestic pig genome. *PNAS* 109 19529–19536. 10.1073/pnas.1217149109 23151514 PMC3511700

[B61] Rubin C. J. Zody M. C. Eriksson J. Meadows J. R. Sherwood E. Webster M. T. (2010). Whole-genome resequencing reveals *Loci* under selection during chicken domestication. *Nature* 464 587–591. 10.1038/nature08832 20220755

[B62] Sánchez-Molano E. Cerna A. Toro M. A. Bouza C. Hermida M. Pardo B. G. (2011). Detection of growth-related QTL in turbot (*Scophthalmus maximus*). *BMC Genomics* 12:473. 10.1186/1471-2164-12-473 21958071 PMC3195100

[B63] Saura M. Carabaño M. J. Fernández A. Cabaleiro S. Doeschl-Wilson A. B. Anacleto O. (2019). Disentangling genetic variation for resistance and endurance to scuticociliatosis in turbot using pedigree and genomic information. *Front. Genet.* 10:539 10.3389/fgene.2019.00539 PMC6565924 31231428

[B64] Sciara A. A. Rodríguez-Ramilo S. T. Hermida M. Gómez-Tato A. Fernández J. Bouza C. (2018). Validation of growth-related quantitative trait loci markers in turbot (*Scophthalmus maximus*) families as a step toward marker assisted selection. *Aquaculture* 495 602–610. 10.1016/j.aquaculture.2018.06.010

[B65] Smith J. M. Haigh J. (1974). The hitch-hiking effect of a favorable gene. *Genet. Res.* 23 23–35. 10.1017/s0016672300014634 4407212

[B66] Stainton J. J. Charlesworth B. Haley C. S. Kranis A. Watson K. Wiener P. (2016). Use of high-density SNP data to identify patterns of diversity and signatures of selection in broiler chickens. *J. Anim. Breed. Genet.* 134 87–97. 10.1111/jbg.12228 27349343 PMC5363361

[B67] Sun L. Liu S. Wang R. Jiang Y. Zhang Y. Zhang J. (2014). Identification and analysis of genome-wide SNPs provide insight into signatures of selection and domestication in channel catfish (*Ictalurus punctatus*). *PLoS One* 9:e109666. 10.1371/journal.pone.0109666 25313648 PMC4196944

[B68] Sutton J. T. Nakagawa S. Robertson B. C. Jamieson I. G. (2011). Disentangling the roles of natural selection and genetic drift in shaping variation at MHC immunity genes. *Mol. Ecol.* 20 4408–4420. 10.1111/j.1365-294X.2011.05292.x 21981032

[B69] Taboada X. Hermida M. Pardo B. G. Vera M. Piferrer F. Viñas A. (2014). Fine mapping and evolution of the major sex determining region in turbot (*Scophthalmus maximus*). *G3 Gens Genomes Genet.* 4 1871–1880. 10.1534/g3.114.012328 25106948 PMC4199694

[B70] Vasemägi A. Nilsson J. McGinnity P. Cross T. O’Reilly P. Glebe B. (2012). Screen for Footprings Of Selection During Domestication/Captive Breeding Of Atlantic Salmon. *Comp. Funct. Genom.* 2012:628204. 10.1155/2012/628204 23326209 PMC3544263

[B71] Vilas R. Bouza C. Vera M. Millán A. Martínez P. (2010). Variation in anonymous and EST-microsatellites suggests adaptive population divergence in turbot. *Mar. Ecol. Prog. Ser.* 420 231–239. 10.3354/meps08874

[B72] Vilas R. Vandamme S. G. Vera M. Bouza C. Maes G. E. Volckaert F. A. (2015). A genome scan for candidate genes involved in the adaptation of turbot (*Scophthalmus maximus*). *Mar. Genom.* 23 77–86. 10.1016/j.margen.2015.04.011 25959584

[B73] Wang X. Li C. Thongda W. Luo Y. Bech B. Peatman E. (2014). Characterization and mucosal responses of interleukin 17 family ligand and receptor genes in channel catfish *Ictalurus punctatus*. *Fish Shellfish Immunol.* 38 47–55. 10.1016/j.fsi.2014.02.020 24602926

[B74] Webb A. E. Gerek Z. N. Morgan C. C. Walsh T. A. Loscher C. E. Edwards S. V. (2015). Adaptive evolution as a predictor of species-specific innate immune response. *Mol. Biol. Evol.* 32 1717–1729. 10.1093/molbev/msv051 25758009 PMC4476151

[B75] Worley K. Collet J. Spurgin L. G. Cornwallis C. Pizzari T. Richardson D. S. (2010). MHC heterozygosity and survival in red junglefowl. *Mol. Ecol.* 19 3064–3075. 10.1111/j.1365-294X.2010.04724.x 20618904

[B76] Zhao S. Zhang T. Liu Q. Wu H. Su B. Shi P. (2019). Identifying lineage-specific targets of natural selection by a bayesian analysis of genomic polymorphisms and divergence from multiple species. *Mol. Biol. Evol.* 36 1302–1315. 10.1093/molbev/msz046 30840083

[B77] Zwane A. A. Schnabel R. D. Hoff J. Choudhury A. Makgahlela M. L. Maiwashe A. (2019). Genome-wide SNP discovery in indigenous cattle breeds of South Africa. *Front. Genet.* 10:273. 10.3389/fgene.2019.00273 30988672 PMC6452414

