# Supplementary material for: Genomic Signatures After Five Generations of Intensive Selective Breeding: Runs of Homozygosity and Genetic Diversity in Representative Domestic and Wild Populations of Turbot (Scophthalmus maximus)
Source: Front Genet. 2020 Apr 3;11:296. doi: 10.3389/fgene.2020.00296 (PMC7169425; doi:10.3389/fgene.2020.00296)
Supplement: Supplementary file 3 [file Table_3.docx]

Supplementary Table 3. Genotyping of 21.615 SNP loci in turbot broodstock (46 individuals) using Genepop format (http://genepop.curtin.edu.au/). SNP genotypes are indicated as follows: 01 = A, 02 = C, 03 = G and 04 = T.

154721,154725,154740,154746,154773,154783,154784,154786,146344,146189,146284,146325,146338,146339,146340,146351,146357,146379,146382,146383,146386,146393,146395,146404,146417,146422,146430,146440,146446,146466,146475,146485,146499,146544,146553,146130,146131,146140,146144,146148,146176,146206,146214,146219,146221,146233,146234,146238,146241,146250,146255,146266,146289,146292,146309,146310,146313,146314,146316,146321,158218,158213,158202,158194,158183,158174,158234,165487,136952,136944,136925,136917,136916,136892,136875,136867,136858,136840,136832,136830,136806,136797,136794,136793,136790,136789,136783,136782,136777,136766,136749,136747,136740,136730,136721,136719,136712,136699,136684,136681,137350,137341,137339,137317,137307,137295,137290,137287,137284,137278,137272,137270,137268,137260,137250,137246,137245,137240,137237,137236,137225,137222,137208,137206,137203,137200,137191,137190,137180,137175,137161,137153,137145,137127,137120,137118,137115,137110,137108,137099,137098,137092,137086,137085,137077,137073,137057,137055,137036,137030,137029,137024,137022,158691,158690,158688,158686,158674,158668,158663,158661,158657,158715,158677,164006,164005,164015,157076,157125,157153,157160,157165,156996,157020,157022,157026,157038,157051,157065,157073,157080,157081,157095,157110,157111,157120,157136,157139,157143,152453,152433,152423,152418,152411,152406,152403,152400,152392,152377,152371,152346,152342,152338,152327,152325,152274,152272,152270,152268,152266,152264,152257,152253,152250,152240,152238,152211,152203,152196,152328,152190,159512,159522,159463,159475,159495,159501,159507,159510,157872,157869,157868,157861,157851,157845,157832,157828,157826,157823,157818,157812,157802,157780,157899,163816,165113,165674,156881,156986,156989,156991,156869,156878,156879,156880,156892,156904,156905,156911,156920,156926,156927,156931,156932,156945,156946,156949,156954,156955,156965,165134,158400,158394,158393,158385,158379,158359,158354,158345,158424,158422,158415,158378,158373,158344,160131,160146,156398,156393,156391,156382,156375,156368,156367,156366,156361,156358,156355,156349,156326,156316,156415,156399,156364,156407,140439,140432,140418,140394,140391,140377,140375,140369,140360,140357,140348,140342,140339,140326,140316,140308,140299,140295,140282,140246,140227,140215,140212,140211,140203,140199,140195,140183,140160,140146,140145,140144,140441,140371,140297,140434,156616,156669,156562,156592,156666,156672,156674,156527,156530,156536,156538,156542,156544,156551,156556,156561,156567,156571,156576,156580,156583,156585,156594,156598,156604,156612,156625,156633,156637,156642,156653,156656,156662,164559,149867,149865,149853,149847,149841,149839,149820,149809,149796,149791,149789,149788,149784,149779,149757,149732,149705,149695,149679,149855,149767,149760,149719,148994,148978,148963,148930,161321,161255,161269,161310,163625,163628,139795,139806,139836,139840,139853,139857,139859,139862,139899,139910,139920,139922,139955,139957,139961,139963,139980,140003,140006,140014,140022,140040,140052,140055,140064,140109,140108,140113,140127,151764,151753,151738,151701,151697,151690,151766,151673,101852,101859,101862,101863,101880,101884,101886,101899,101901,101918,101920,101932,101954,101968,101972,101977,101978,101985,101990,102002,102011,147150,147072,147050,147025,147003,147001,146986,146950,146947,146945,147184,147178,146944,127306,127299,127283,127260,127253,127233,127221,127207,127185,127164,127157,127155,127141,127137,127136,127129,127120,127110,127102,127101,127096,127091,127082,127076,127073,127070,127068,127055,127041,127640,127638,127632,127608,127590,127589,127564,127562,127559,127553,127552,127547,127545,127502,127478,127473,127438,127428,127426,127424,127416,127397,127395,127394,127389,127381,127373,127368,127367,127353,127307,127179,96348,96355,96386,96388,96411,96412,96438,96458,96462,96468,96471,96490,96492,96493,166012,25780,25775,25770,25760,25756,25752,25748,25743,25742,25738,25732,25730,25717,25709,25708,25699,25697,25695,25679,25675,25672,25639,25616,25607,25605,25603,25564,25560,25555,25546,25545,25532,25521,25515,25480,25471,25462,25450,25446,25439,25435,25421,25415,25405,25391,25373,25367,25356,25354,25331,29133,29131,29130,29129,29126,29118,29110,29095,29082,29078,29075,29073,29069,29058,29056,29044,29038,29029,29016,29012,29011,29010,29009,29000,28999,28994,28991,28987,28985,28973,28954,28936,28932,28917,28912,28907,28893,28892,28875,28862,28843,28820,28799,28797,28792,28780,28775,28772,28768,28755,28753,28725,28701,28692,28691,28688,28684,28679,28666,28652,28650,28646,28629,28621,28605,28601,28595,28585,28578,28576,28575,28568,28561,28558,28555,28542,28538,28534,28529,28526,28503,28496,28495,28481,28461,28457,28434,28410,28370,28348,28346,28339,28338,28333,28322,28319,28316,28315,28309,28304,28302,28290,28280,28278,28249,28230,28229,28222,28219,28206,28195,28194,28185,28183,28175,28173,28159,28156,28155,28145,28138,28123,28122,28118,28115,28100,28085,28074,28073,28072,28067,28065,28062,28052,28046,28039,28033,28012,27988,27985,27984,27977,27970,27962,27954,27944,27937,27904,27903,27888,27878,27874,27864,27857,27851,27838,27834,27812,27800,27768,27758,27754,27739,27720,27712,27687,27680,27659,27650,27649,27648,27642,27611,27607,27596,27593,27583,27537,27534,27533,27531,27526,27524,27520,27510,27503,27497,27492,27491,27485,27461,27431,27407,27397,27384,27373,27346,27313,27309,27308,27303,27300,27295,27248,27246,27243,27240,27239,27235,27229,27225,27205,27193,27185,27173,27170,27158,27125,27105,27104,27094,27086,27084,27080,27066,27064,27061,27060,27055,27051,27046,27032,27025,27017,27013,27005,26989,26979,26969,26965,26953,26949,26925,26898,26876,26873,26872,26871,26865,26860,26859,26846,26844,26840,26831,26779,26778,26774,26749,26746,26720,26683,26682,26681,26668,26649,26636,26635,26611,26138,26448,26532,27072,27203,27410,28210,28217,25928,25983,25986,26131,26295,26433,26514,26537,26670,26689,26705,26728,26756,26823,26824,26861,26863,26966,26993,27160,27197,27286,27367,27439,27656,27660,27696,27767,27805,27825,27899,27935,28019,28106,28356,28388,28436,28676,28677,28707,28825,28919,28960,28998,29028,29048,29049,29050,25485,25505,25541,25544,25635,25636,25638,25642,25648,25649,25658,25719,25747,25793,25807,25821,25826,25827,25834,25835,25841,25846,25849,25851,25864,25865,25871,25875,25898,25903,25906,25920,25922,25940,25943,25945,25959,25963,25974,25975,26018,26033,26046,26057,26070,26071,26089,26090,26091,26094,26097,26098,26102,26108,26109,26123,26135,26144,26151,26167,26171,26174,26179,26185,26201,26207,26215,26219,26224,26231,26255,26278,26293,26303,26304,26333,26335,26343,26346,26351,26355,26375,26376,26379,26382,26389,26390,26401,26403,26422,26423,26442,26460,26464,26467,26475,26482,26493,26494,26525,26531,26536,26538,26540,26549,26561,26565,26568,26569,26570,26574,26576,26580,26581,26582,26592,26603,26606,166325,165684,165240,142996,142990,142988,142976,142971,142968,142965,142964,142960,142945,142940,142926,142923,142917,142916,142910,142893,142892,142886,142885,142875,142871,142869,142860,142859,142857,142827,142826,142825,142824,142817,142813,142803,142791,142785,142754,142742,142739,143042,142781,142776,165131,120385,120373,120366,120325,120320,120310,120303,120291,120290,120281,120280,120267,120226,120224,120216,120204,120201,120193,120185,120171,120170,120163,120152,120147,120143,120134,120131,120125,120100,120094,120091,120666,120645,120628,120589,120566,120562,120559,120557,120556,120545,120543,120538,120537,120536,120535,120527,120503,120496,120495,120490,120489,120486,120482,120474,120471,120458,120457,120455,120453,120441,120425,120403,120400,120394,120356,120352,120342,120211,120210,120110,120615,120504,120407,120107,165749,148065,148130,148133,147953,147955,147958,147960,147968,147970,147976,147981,147985,147986,148004,148029,148061,148060,148064,148089,148107,148108,148112,165700,124428,124385,124386,124383,124379,124378,124371,124370,124369,124350,124345,124344,124319,124309,124299,124298,124292,124291,124285,124281,124279,124278,124275,124271,124269,124255,124250,124711,124710,124703,124700,124699,124684,124670,124665,124660,124648,124647,124638,124636,124601,124600,124593,124582,124578,124558,124557,124547,124543,124541,124539,124527,124509,124501,124499,124487,124457,124268,124265,124635,124486,124297,124646,164309,164277,164279,164282,157513,157510,157504,157502,157498,157493,157495,157494,157491,157490,157487,157482,157470,157548,157546,163732,163730,163729,163726,138469,138447,138489,138492,138493,138498,138504,138538,138560,138561,138567,138574,138583,138593,138596,138599,138610,138614,138615,132231,132227,132223,132218,132214,132198,132421,132413,132404,132399,132376,132373,132350,132348,132343,132336,132326,132323,132307,132280,132279,132275,132269,132258,132236,132219,132334,132259,109061,109197,109324,109355,108920,108932,108960,109007,109108,109137,109138,109140,109185,109194,109198,109212,109215,109219,109227,109228,109235,161833,161849,161851,149325,149409,149410,149437,149276,149277,149310,149363,149366,149389,149421,149427,165915,156677,156682,156690,156697,156699,156709,156733,156739,156742,122313,122307,122298,122296,122290,122283,122274,122273,122270,122269,122256,122255,122249,122247,122243,122236,122231,122227,122223,122218,122165,122162,122157,122131,122114,122112,122092,122088,122078,122071,122065,122062,122059,122057,122049,122048,122043,122042,122037,122036,122035,122034,122593,122581,122573,122569,122567,122566,122550,122546,122538,122527,122526,122524,122514,122503,122500,122496,122493,122459,122440,122433,122421,122413,122409,122395,122394,122373,122371,122370,122365,122360,122357,122351,122347,122323,122321,122107,122064,122555,125001,125302,124870,124968,124990,125000,125013,125020,125095,125101,125102,125118,125128,125136,125152,125189,125210,125238,125242,125244,125257,125273,125276,125298,125301,125316,125341,125344,124726,124752,124756,124781,124796,124800,124815,124817,124829,124833,124835,124842,124854,124915,124938,124959,135668,136069,136062,136048,136043,136041,136035,135994,135984,135968,135964,135919,135870,135860,135855,135853,135850,135845,135832,135781,135780,135776,135773,135761,135760,135747,135744,135725,135721,136056,136049,136013,135895,135880,135741,127679,127879,127944,127946,127993,127997,128007,128019,128025,128034,128062,128064,128066,128091,128115,128117,128118,128125,128145,128154,128159,128160,127642,127645,127649,127655,127659,127661,127683,127695,127710,127711,127712,127718,127733,127735,127738,127741,127746,127751,127757,127767,127780,127804,127813,127816,127817,127827,127839,127846,127851,127862,127874,127876,127884,127893,127896,127898,164387,164385,154964,154957,154934,154894,154870,154970,159209,159156,159148,159127,165835,99152,98327,98331,98593,98606,98610,98633,98679,99072,99092,99093,99095,99099,99102,99105,99120,99122,99124,99128,99138,99140,99141,99143,99150,99183,99192,99203,99207,99211,99215,99218,99223,99225,99230,99231,99237,99239,99240,99247,99249,97985,97997,98000,98003,98004,98007,98012,98021,98031,98054,98056,98063,98080,98084,98092,98094,98105,98114,98115,98119,98129,98133,98135,98154,98155,98156,98161,98164,98169,98179,98183,98192,98193,98194,98209,98215,98226,98227,98238,98256,98272,98280,98297,98300,98305,98309,98315,98317,98320,98321,98350,98351,98375,98378,98380,98392,98394,98400,98404,98410,98415,98416,98441,98444,98450,98460,98470,98477,98494,98499,98512,98513,98535,98537,98542,98545,98556,98562,98565,98581,98587,98590,98612,98613,98621,98628,98634,98635,98644,98647,98650,98653,98654,98657,98658,98660,98668,98675,98677,98697,98702,98704,98722,98724,98727,98747,98750,98756,98765,98768,98779,98808,98812,98840,98843,98846,98847,98853,98878,98881,98883,98892,98897,98905,98908,98912,98915,98916,98926,98930,98949,98950,98964,98978,98979,98980,98990,98991,98999,99005,99007,99013,99022,99032,99033,99046,99047,99048,99060,99062,99064,93886,92983,93060,93238,93301,93317,93347,93452,93474,93517,93569,93636,93646,93753,93761,93762,93790,93791,93801,93803,93804,93811,93815,93816,93819,93841,93856,93860,93867,93870,93880,93890,93894,93907,93916,93920,93928,93930,92613,92614,92636,92640,92648,92650,92662,92664,92665,92672,92673,92684,92693,92694,92697,92723,92724,92732,92747,92754,92765,92766,92768,92775,92776,92779,92793,92801,92810,92818,92823,92829,92837,92838,92840,92845,92846,92855,92865,92867,92870,92872,92876,92878,92881,92884,92885,92907,92908,92910,92912,92916,92925,92928,92931,92955,92966,92987,93015,93021,93039,93047,93051,93058,93062,93070,93073,93091,93092,93098,93104,93108,93133,93136,93138,93141,93151,93161,93169,93170,93185,93193,93198,93200,93218,93222,93224,93225,93227,93229,93237,93242,93243,93245,93249,93257,93270,93275,93279,93281,93284,93289,93302,93306,93307,93308,93312,93326,93335,93341,93349,93353,93361,93370,93377,93388,93391,93393,93425,93427,93428,93429,93436,93446,93460,93467,93478,93487,93488,93512,93513,93519,93521,93529,93530,93536,93543,93549,93560,93570,93573,93580,93584,93621,93630,93660,93663,93669,93671,93677,93683,93698,93700,93715,93720,93734,93736,93739,93743,93748,154673,154687,154567,154576,154584,154600,154601,154606,154608,154622,154623,154629,154647,154649,154661,154663,154664,154678,109256,109261,109273,109290,109315,109320,109332,109334,109340,109347,109348,109359,109370,109371,109374,109375,109381,109382,109383,109385,109387,109400,109401,109402,109406,109407,109408,108697,108701,108707,108708,108710,108711,108718,108723,108731,108737,108747,108749,108753,108757,108764,108765,108768,108774,108777,108781,108791,108801,108806,108810,108811,108821,108822,108823,108824,108831,108832,108834,108845,108847,108856,108869,108872,108883,108891,108895,108900,108904,108914,108922,108925,108930,108945,108948,108954,108955,108985,109008,109011,109013,109024,109026,109029,109034,109035,109059,109062,109064,109069,109076,109077,109095,109101,109103,109121,109133,109136,109141,109142,109148,109149,109156,109158,109165,161708,161713,144072,144052,144049,144045,144037,144032,144026,144017,144002,143991,143990,143961,143959,143945,143936,143935,143928,143919,143916,143908,144077,143932,158014,158010,157980,158035,158030,165778,158760,158755,158748,158745,158744,158732,158778,158768,164484,161234,161246,164784,161738,161747,161745,162812,154244,154161,154160,154189,154194,154199,162368,162369,162352,165163,162942,162934,162916,162929,164835,165721,103477,103469,103465,103416,103392,103384,103334,103309,103303,103297,103285,103283,103271,103269,103268,103267,103266,103263,103254,103238,103221,103187,103169,103168,103164,103154,103136,103135,103133,103130,103095,103083,103069,103059,103054,103036,103021,103010,102990,102988,102960,102928,102915,102907,102906,102896,102892,102882,102866,102860,102859,102848,102847,102843,102841,102835,102825,102824,102816,102801,102800,102789,102786,102779,102776,102771,102750,102740,102735,102732,102731,102715,102713,102707,102705,102704,102693,102689,102685,102684,102680,102675,102671,102658,102641,102631,102615,102603,102598,102597,102591,102574,102569,102568,102562,102560,103711,103681,103679,103677,103667,103659,103653,103642,103634,103622,103620,103597,103592,103586,103584,103581,103579,103577,103568,103567,103549,103548,103533,103524,103522,103520,103519,103516,103515,103504,103496,103493,103484,103479,103478,103430,103329,103206,103173,102957,102922,102837,102765,102749,102638,102629,103686,103644,103629,165321,165278,81651,81629,81625,81618,81610,81607,81602,81595,81594,81591,81583,81581,81578,81577,81569,81567,81566,81565,81563,81562,81550,81546,81533,81525,81505,81482,81466,81452,81449,81448,81435,81424,81417,81406,81402,81395,81393,81391,81379,81377,81366,81359,81352,81351,81349,81346,81343,81336,81329,81322,81314,81308,81304,81303,81297,81289,81276,81261,81234,81230,81226,81224,81205,81183,81178,81171,81166,81153,81143,81142,81138,81130,81120,81118,81112,81109,81103,81077,81074,81064,81063,81054,81049,81047,81044,81026,81018,80996,80994,80986,80981,80977,80966,80958,80957,80950,80949,80945,80938,80935,80927,80925,80923,80918,80903,80897,80895,80878,80862,80859,80846,80843,80836,80832,80829,80828,80807,80806,80796,80793,80786,80784,80778,80759,80748,80733,80727,80705,80700,80699,80698,80695,80672,80667,80661,80651,80641,80639,80633,80622,80619,80615,80612,80603,80595,80579,80572,80567,80566,80557,80540,80527,80526,80484,80450,80447,80428,80423,80410,80408,80401,80400,80388,80387,80379,80365,80358,80347,80337,80315,80298,80297,80270,80262,80260,80253,80247,80225,80220,80207,81861,81855,81849,81843,81830,81828,81813,81810,81808,81791,81784,81774,81763,81756,81754,81753,81748,81747,81737,81730,81720,81706,81682,81674,81673,81672,81667,81657,81628,81627,81590,81500,81491,81279,81225,81197,81096,80951,80940,80758,80735,80533,80525,80510,80464,80463,81833,81772,164781,164775,162875,162886,164625,164627,126109,126105,126101,126100,126090,126088,126060,126056,126045,126031,126022,125999,125998,125980,125971,125935,125923,125895,126477,126473,126457,126453,126447,126445,126433,126414,126413,126410,126405,126403,126402,126382,126381,126372,126368,126364,126358,126353,126349,126342,126335,126315,126313,126292,126289,126279,126270,126249,126242,126235,126211,126192,126187,126181,126173,126159,126132,126117,126021,125965,125932,126272,126212,164853,165142,165144,163605,163573,163574,163576,163582,163590,163592,163596,163557,164945,75414,75384,75383,75376,75351,75350,75347,75345,75330,75322,75313,75311,75298,75269,75258,75257,75252,75245,75239,75238,75237,75216,75192,75190,75186,75182,75175,75161,75159,75152,75145,75137,75127,75125,75117,75114,75100,75097,75081,75075,75068,75054,75048,75023,75019,75015,74997,74992,74981,74971,74969,74956,74934,74915,74911,74906,74900,74891,74887,74885,74882,74878,74873,74868,74865,74845,74835,74822,74808,74805,74802,74794,74787,74785,74778,74776,74741,74738,74723,74713,74712,74711,74706,74681,74680,74674,74666,74665,74663,74661,74653,74632,74627,74623,74622,74614,74606,74604,74596,74574,74571,74528,74519,74515,74508,74507,74505,74499,74497,74492,74487,74480,74478,74465,74454,74443,74426,74420,74417,74397,74390,74385,74376,74340,74320,74315,74305,74297,74292,74282,74276,74257,74235,74230,74226,74225,74205,74189,74163,74162,74157,74146,74132,74129,74122,74120,74119,74113,74107,74105,74098,74094,74074,74070,74056,74046,74016,73994,73985,73979,73975,73964,73949,73945,73922,73915,73914,73909,73899,73896,73881,73876,73875,73869,73853,75585,75580,75550,75548,75542,75539,75536,75522,75506,75505,75493,75490,75484,75474,75473,75466,75458,75455,75454,75451,75422,75421,75353,75337,75283,75213,75111,75073,74916,74800,74649,74568,74537,74524,74355,74317,74278,74250,74240,74188,74180,74093,74062,74896,74306,74837,159758,159751,159750,159742,159774,159772,113951,113937,113903,113899,113898,113871,113867,113848,113846,113837,113830,113823,113796,113787,113780,113764,113763,113754,113747,113732,113728,113719,113706,113693,113689,113687,113683,113681,113679,113675,113673,113659,113652,113648,113613,113612,113594,113590,113584,113582,113574,113565,113559,113558,113556,113554,113536,113523,113520,113516,114257,114254,114219,114213,114200,114199,114189,114182,114179,114166,114163,114162,114134,114127,114125,114105,114102,114098,114091,114082,114057,114043,114041,114036,114005,113999,113984,113981,113980,113961,113875,113755,113692,114075,114074,113998,113690,165476,85830,85833,84906,84934,84939,84947,84964,85052,85237,85244,85371,85413,85421,85505,85520,85527,85568,85602,85625,85639,85687,85737,85739,85761,85771,85774,85795,85798,85804,85806,85811,85812,85814,85820,85824,85839,85840,85841,85854,85887,85891,85897,85916,85917,85923,85925,85927,85929,85933,85934,85960,85964,85976,85987,85988,85993,86000,86002,86005,86007,84804,84811,84826,84829,84832,84841,84845,84847,84850,84879,84892,84907,84908,84914,84916,84919,84928,84931,84935,84942,84949,84951,84960,84971,84972,84973,84976,84979,84981,84982,84988,84989,84991,85013,85023,85024,85037,85038,85051,85053,85072,85073,85082,85084,85087,85088,85090,85095,85106,85111,85113,85126,85139,85143,85146,85147,85148,85159,85161,85167,85171,85181,85202,85211,85221,85225,85277,85298,85325,85334,85337,85338,85339,85358,85366,85373,85374,85375,85385,85391,85395,85403,85438,85443,85447,85459,85464,85465,85468,85471,85478,85481,85484,85486,85490,85491,85494,85500,85508,85513,85518,85529,85531,85543,85549,85581,85592,85593,85601,85611,85623,85632,85641,85644,85646,85656,85676,85680,85689,85715,85717,85724,85746,85750,85776,85780,85783,166181,165576,165573,162344,160017,160048,160037,160493,160494,160474,160482,160483,155355,155350,155349,155348,155336,155360,162379,162378,162386,163493,165628,162998,162556,162552,162550,164401,164400,164397,163926,166036,160640,160639,160653,160652,160650,160648,160655,154804,154805,154808,154809,154815,154821,154834,154840,154842,154845,159453,159457,159462,159419,159421,159424,159425,159442,159447,159449,161432,161442,161425,165495,119911,119954,119964,119987,120066,119682,119685,119712,119784,119813,119851,119875,119880,119881,119883,119886,119888,119894,119896,119899,119903,119907,119914,119918,119934,119936,119943,119944,119946,119965,119978,119983,119988,119996,120004,120007,120012,120013,120016,120019,120020,120030,120045,120072,120073,120074,120078,120081,120085,120086,119645,119648,119653,119656,119662,119666,119670,119686,119687,119688,119697,119705,119717,119726,119727,119731,119739,119756,119788,119789,119791,119840,119869,45015,45010,45009,45007,45006,45001,44989,44986,44983,44978,44964,44946,44934,44926,44924,44914,44901,44900,44894,44891,44878,44869,44862,44858,44855,44838,44833,44832,44812,44804,44793,44791,44783,44782,44779,44774,44768,44761,44758,44754,44747,44734,44712,44709,44697,44692,44689,44685,44679,44676,44675,44661,44652,44645,44639,44629,44621,44599,44594,44592,44586,44585,44564,44562,44556,44551,44547,44537,44535,44524,44518,44516,44511,44498,44496,44489,44484,44483,44474,44471,44470,44467,44461,44457,44456,44452,44448,44435,44414,44408,44401,44394,44374,44371,44367,44361,48342,148921,148919,148917,148916,148912,148902,148900,148891,148874,148867,148850,148849,148828,148813,148809,148801,148778,148774,148768,148835,135350,135327,135313,135645,135618,135601,135597,135571,135556,135552,135540,135538,135536,135535,135529,135519,135518,135507,135501,135467,135459,135431,135419,135404,135403,135399,135398,135397,135396,135383,135378,135374,135357,135355,135582,123893,123888,123873,123867,123866,123859,123850,123847,123845,123822,123804,123788,123786,123785,123780,123772,123770,123769,123768,123763,123740,123729,123728,123727,123709,123698,123688,123687,123681,123680,123679,123658,123655,123652,123651,123629,123628,124248,124227,124219,124207,124202,124196,124194,124186,124183,124177,124166,124157,124150,124146,124145,124138,124134,124126,124121,124115,124074,124067,124056,124052,124045,124002,123991,123985,123975,123973,123972,123965,123959,123957,123954,123951,123940,123932,123931,123926,123925,123923,123922,123920,123919,123916,123911,123907,123902,123900,123640,124234,124049,123842,53814,53810,53801,53796,53793,53790,53788,53779,53770,53768,53729,53726,53724,53702,53687,53678,53675,53671,53663,53657,53654,53649,53641,53632,53625,53624,53622,53615,53559,53554,53551,53546,53540,53534,53532,53511,53509,53500,53497,53493,53474,53463,53442,53439,53436,53421,53419,53417,53412,53402,53400,53394,53381,53369,53364,53361,53348,53346,53333,53327,53316,53306,53291,53285,53282,53279,53271,53267,53262,53256,53255,53253,53249,53232,53231,53215,53207,53202,53192,53186,53182,53176,53173,53163,53158,53154,53139,53129,53128,53114,53113,53109,53107,53101,53097,53090,53089,53080,53076,53067,53059,53052,53044,53031,53010,53004,53003,52990,52987,52981,52980,52971,52965,52964,52952,52949,52924,52911,52910,52887,52879,52876,52866,52865,52862,52857,52851,52848,52839,52827,52819,52817,52809,52802,52795,52787,52772,52771,52769,52767,52746,52716,52714,52709,52702,52692,52684,52681,52673,52671,52670,52667,52663,52627,52626,52618,52608,52583,52556,52553,52546,52543,52532,52529,52527,52526,52520,52519,52512,52481,52474,52463,52436,52435,52426,52423,52414,52401,52399,52398,52394,52377,52369,52340,52330,52317,52307,52295,52277,52274,52272,52266,52259,52251,52229,52224,52195,52189,52153,52144,52143,52135,52112,52105,52087,52086,52083,52053,52045,52042,52013,52011,52002,52001,52000,51993,51985,51955,51950,51946,51934,51924,51913,51896,51893,51892,51877,51872,51868,51862,51857,51833,51824,51821,51820,51817,51812,51809,51807,51805,51785,51784,51779,51774,51772,51763,51754,51745,51742,51739,51734,51732,51724,51715,51697,51693,51692,51681,51669,51668,51647,51646,51640,51639,51623,51576,51570,51559,51554,51551,51535,51523,51516,51510,51505,51504,51498,51493,51489,51484,51481,51473,51471,51470,51458,51456,51410,51396,51389,51388,51369,51358,51357,51354,51344,51342,51339,51335,51334,51333,51319,51318,51307,51306,51300,51297,51274,51270,51253,51252,51234,51232,51226,51218,51215,51213,51212,51204,51195,51187,51180,51160,51154,51153,51147,51142,51138,51114,51093,51089,51081,51061,51055,51047,51044,51028,51021,51010,51009,51008,51004,51002,50998,50993,50992,50991,50986,50981,50977,50965,50960,50936,50929,53842,53830,53695,53668,53635,53629,53471,53332,53320,53150,53146,53140,52908,52790,52706,52688,52602,52598,52592,52468,52454,52200,52089,52075,52018,51973,51859,51834,51701,51517,51497,51450,51208,51206,51171,51141,51134,51080,51069,51035,50982,50924,53860,53524,53203,52325,51083,163697,163702,163706,164571,164572,163243,163241,163219,163216,163188,163259,163257,163285,163278,116856,116853,116852,116851,116842,116838,116828,116814,116795,116790,116768,116740,116721,116708,116705,116704,116681,116667,116665,116645,116640,116622,116586,116582,116570,116569,116563,116551,116549,116529,116526,116479,116446,117127,117126,117125,117124,117121,117120,117119,117104,117100,117095,117091,117085,117064,117063,117058,117055,117042,117024,116996,116971,116962,116961,116954,116943,116938,116937,116935,116923,116919,116898,116886,116885,116882,116876,116866,116864,116819,116758,116724,116703,116683,116631,116605,116562,116557,116541,117123,116925,144916,144911,144907,144905,144898,144870,144865,144783,144770,144757,144753,144743,97812,97861,96814,96966,97016,97073,97078,97090,97114,97123,97328,97329,97646,97678,97803,97815,97830,97841,97851,97853,97870,97877,97901,97903,97913,97914,97919,97931,97935,97936,97937,97938,97940,97944,97953,97964,96729,96735,96739,96750,96756,96757,96761,96768,96774,96776,96801,96807,96808,96813,96819,96827,96831,96834,96844,96847,96852,96854,96855,96857,96882,96883,96912,96922,96924,96927,96929,96935,96948,96960,96961,96965,96969,96989,97008,97009,97019,97021,97022,97032,97035,97049,97051,97053,97054,97055,97057,97060,97064,97071,97080,97084,97085,97087,97096,97108,97139,97145,97147,97163,97170,97171,97182,97183,97186,97196,97198,97199,97200,97207,97209,97211,97212,97218,97226,97229,97231,97232,97235,97237,97239,97248,97249,97250,97252,97257,97258,97266,97269,97279,97287,97293,97299,97313,97325,97343,97344,97351,97368,97372,97386,97389,97403,97404,97419,97420,97429,97440,97446,97447,97456,97464,97470,97477,97483,97491,97492,97506,97507,97511,97515,97527,97538,97546,97548,97549,97568,97579,97581,97602,97610,97612,97622,97627,97632,97634,97638,97655,97658,97663,97667,97673,97674,97687,97700,97704,97708,97715,97718,97728,97736,97741,97743,97745,97757,97759,97762,97763,97764,97768,159919,159916,159909,159908,159877,159874,159943,159941,159940,159937,159934,159925,159928,114833,114841,114863,114870,114878,114882,114888,114902,114906,114299,114307,114315,114322,114328,114335,114348,114354,114359,114364,114368,114389,114402,114427,114428,114429,114430,114431,161378,161375,161368,161366,161352,161351,161349,161343,161334,161403,161392,161391,161383,161379,158333,158253,158268,158273,158274,158277,158283,158294,158302,134716,134723,134759,134761,134764,134780,134783,134790,134814,134817,134825,134830,134834,134835,134860,134884,134885,134894,134525,134533,134552,141768,141519,141667,141787,141800,141836,141941,141942,141944,141957,141963,141966,141980,141982,141988,141996,142003,142017,142019,142023,142026,142027,142039,142059,142078,142080,142088,142102,142105,142112,142114,142120,142135,142151,142155,142162,142171,142181,142185,142194,142200,142204,142227,142229,142233,141491,141497,141500,141505,141514,141524,141525,141528,141532,141540,141556,141558,141559,141562,141561,141565,141566,141570,141571,141576,141579,141586,141590,141595,141608,141622,141638,141643,141660,141662,141681,141706,141712,141728,141729,141731,141735,141748,141750,141775,141782,141817,141819,141832,141835,141839,141866,141868,141874,141897,141902,141905,141916,141917,141927,161640,161623,161622,161607,161576,161569,161567,161663,161633,145643,145654,145743,145899,145541,145611,145631,145641,145649,145651,145660,145665,145677,145697,145699,145700,145722,145742,145747,145750,145762,145774,145781,145788,145800,145804,145807,165395,151940,151943,151874,151951,151956,151796,151800,151811,151832,151873,151891,151892,151896,151917,151928,151933,163720,162981,162985,162748,162747,165546,153069,153085,153086,153090,153101,153103,153108,153117,153118,157282,157293,157291,157290,157286,165860,156033,156075,161719,161724,161727,161728,161733,153882,153879,153861,153860,153849,153844,153843,153875,163962,163958,165230,163469,155267,155254,155247,155237,155231,155217,155287,155275,152556,152549,152542,152541,152540,152527,152526,152523,152512,152494,152490,152487,152486,152482,152480,152475,152560,164638,165818,165820,83149,83048,83079,83080,83100,83102,82070,82206,82324,82332,82337,82469,82493,82687,82891,82909,82992,83061,83064,83065,83066,83067,83074,83076,83077,83081,83084,83085,83087,83089,83095,83098,83119,83120,83123,83125,83141,83142,83143,83146,83163,83165,83181,81872,81877,81878,81880,81881,81883,81902,81905,81906,81910,81918,81922,81934,81944,81965,81967,81972,81974,81982,81994,81995,82008,82023,82037,82044,82048,82058,82064,82071,82094,82095,82102,82123,82146,82156,82165,82167,82168,82180,82182,82195,82196,82204,82219,82221,82227,82234,82235,82239,82241,82242,82243,82245,82255,82259,82272,82288,82290,82302,82307,82309,82312,82317,82322,82327,82329,82335,82344,82346,82353,82355,82362,82367,82374,82388,82394,82417,82429,82436,82447,82471,82474,82475,82494,82498,82501,82505,82507,82518,82519,82520,82525,82526,82546,82549,82555,82580,82591,82603,82614,82642,82660,82686,82691,82695,82701,82712,82733,82737,82758,82763,82768,82777,82786,82816,82818,82833,82834,82837,82849,82857,82858,82878,82879,82884,82897,82912,82922,82923,82935,82944,82949,82972,82974,82982,82984,82985,82986,82989,83011,83013,83019,83023,83037,164923,48322,48310,48302,48271,48270,48261,48259,48247,48225,48224,48215,48195,48187,48184,48170,48164,48161,48160,48141,48116,48115,48113,48098,48089,48079,48066,48059,48051,48037,48005,48003,47992,47988,47985,47984,47945,47944,47933,47932,47921,47918,47901,47884,47878,47851,47846,47844,47813,47803,47792,47782,47773,47772,47768,47766,47763,47744,47743,47709,47702,47683,47674,47662,47653,47639,47628,47625,47624,47617,47595,47566,47561,47511,47508,47499,47482,47460,47421,47384,47369,47347,47346,47343,47341,47336,47323,47318,47312,47294,47293,47285,47274,47269,47267,47257,47248,47243,47231,47226,47218,47213,47174,47162,47159,47147,47143,47130,47116,47108,47098,47092,47090,47089,47073,47061,47024,47022,47011,47007,47001,46981,46979,46962,46954,46952,46950,46939,46935,46917,46910,46886,46879,46873,46871,46867,46866,46860,46858,46849,46844,46841,46821,46814,46811,46808,46805,46784,46746,46739,46735,46727,46714,46709,46705,46700,46690,46674,46657,46654,46650,46627,46619,46598,46585,46584,46574,46567,46548,46542,46539,46533,46530,46518,46509,46504,46492,46464,46425,46407,46400,46373,46370,46365,46342,46321,46309,46302,46298,46290,46287,46274,46256,46255,46249,46246,46237,46232,46208,46196,46150,46136,46129,46096,46093,46088,46078,46072,46054,46050,46042,46034,46024,46021,46017,45977,45975,45967,45966,45959,45958,45941,45940,45939,45926,45923,45917,45910,45903,45898,45886,45885,45867,45841,45830,45821,45796,45794,45791,45783,45764,45763,45754,45713,45708,45689,45686,45678,45642,45641,45631,45626,45622,45616,45601,45584,45562,45561,45558,45550,45548,45534,45529,45528,45496,45488,45474,45470,45465,45460,45440,45436,45431,45420,45404,45397,45367,45365,45352,45348,45333,45317,45308,45301,45297,45276,45263,45259,45255,45252,45251,45232,45228,45227,45221,45215,45197,45194,45182,45179,45177,45176,45175,45168,45166,45164,45159,45141,45135,45129,45127,45125,45118,45107,45097,45088,45080,45078,45073,45069,45065,45057,45046,45040,45027,45018,44945,44871,44857,44834,44699,44595,44487,44370,48312,48207,48206,47750,47738,47363,47084,47072,46943,46933,46797,46793,46758,46713,46691,46616,46436,46323,46211,46204,45924,45871,45823,45766,45735,45690,45679,45674,45671,45600,45383,45337,45256,45241,48262,47849,46929,46432,45239,45071,45271,165708,164979,164819,86113,86121,86202,86249,86568,86696,86718,86719,86789,87044,87126,87155,87219,87366,87458,87474,87482,87512,87513,87721,87734,87736,87820,87960,88034,88038,88043,88097,88105,88120,88122,88141,88147,88150,88162,88164,88168,86009,86013,86018,86024,86040,86047,86052,86060,86063,86065,86068,86069,86071,86083,86086,86106,86108,86116,86120,86124,86152,86154,86155,86160,86171,86174,86177,86196,86200,86205,86213,86216,86217,86225,86230,86235,86255,86263,86267,86274,86281,86282,86295,86299,86304,86305,86308,86309,86310,86313,86317,86322,86326,86336,86344,86368,86375,86389,86390,86428,86441,86445,86459,86463,86472,86477,86480,86497,86501,86502,86506,86508,86525,86527,86537,86544,86558,86560,86562,86565,86566,86576,86593,86598,86605,86608,86625,86636,86682,86694,86707,86716,86723,86733,86738,86744,86754,86761,86764,86773,86776,86785,86801,86803,86808,86812,86813,86814,86815,86817,86820,86824,86833,86834,86836,86841,86862,86867,86888,86889,86890,86903,86904,86908,86909,86925,86952,86968,86984,86991,86992,86994,86995,87000,87003,87004,87005,87011,87017,87035,87043,87062,87085,87086,87098,87107,87115,87117,87132,87146,87151,87166,87170,87190,87203,87218,87221,87223,87225,87230,87238,87245,87253,87262,87303,87310,87321,87325,87331,87336,87337,87338,87340,87344,87360,87362,87369,87370,87380,87389,87419,87436,87437,87448,87468,87469,87497,87507,87508,87509,87510,87521,87525,87526,87527,87539,87544,87550,87561,87569,87571,87572,87573,87574,87581,87584,87585,87601,87619,87622,87632,87645,87653,87655,87663,87664,87677,87683,87687,87688,87694,87699,87700,87706,87715,87732,87752,87763,87770,87781,87788,87800,87803,87807,87810,87816,87818,87833,87857,87858,87865,87866,87868,87872,87878,87881,87887,87891,87894,87906,87922,87923,87928,87932,87941,87953,87959,87964,87967,87972,87974,87977,87990,87994,87996,132531,132528,132521,132517,132506,132496,132495,132492,132486,132485,132467,132453,132451,132450,132443,132432,132430,132906,132896,132887,132885,132864,132862,132845,132833,132824,132818,132797,132790,132789,132786,132783,132768,132750,132741,132716,132713,132703,132670,132661,132636,132632,132627,132600,132596,132584,132582,132570,132567,132563,132477,132861,132695,132807,110154,110210,110269,109477,109653,109681,109729,109817,109836,109890,109995,109996,109998,110003,110010,110020,110036,110044,110049,110052,110055,110066,110083,110089,110093,110101,110110,110119,110120,110124,110131,110135,110164,110166,110177,110195,110208,110226,110237,110243,110257,110262,110268,110271,110283,110289,110291,110296,110303,110320,110323,109410,109415,109448,109455,109457,109459,109474,109485,109487,109489,109493,109503,109506,109509,109514,109546,109552,109555,109558,109567,109598,109604,109609,109615,109617,109631,109636,109646,109663,109678,109683,109689,109692,109694,109713,109732,109736,109750,109762,109763,109776,109782,109793,109794,109796,109802,109825,109837,109854,109855,109872,109877,109887,109896,109927,109933,109988,139946,139992,152159,152166,152072,152115,148528,148533,148536,148540,148550,148565,148581,148622,148623,148626,148627,148629,148652,148653,164166,143500,143497,143492,143489,143466,143448,143417,143415,143413,143399,143368,143363,143361,143353,143347,143333,143317,143288,143280,143278,143270,143248,143245,143242,143230,143343,143310,165092,166117,146112,146102,146097,146091,146085,146084,146077,146070,146068,146067,146061,146059,146054,146037,146023,146021,146015,146012,146009,145998,145997,145995,145966,145959,146114,162303,162304,162312,162315,162320,138749,138812,138626,138674,138676,138679,138690,138691,138702,138717,138720,138722,138727,138735,138745,138755,138779,138781,138787,138789,138796,138798,138813,138630,138636,138637,138640,138649,138652,138662,138663,138670,138672,162643,162649,162654,157562,157566,157568,157570,157573,157574,157577,157579,165990,160111,160108,160107,160086,160127,146916,146909,146904,146885,146878,146873,146862,146848,146836,146835,146833,146820,146818,146815,146814,146793,146791,146788,146937,146861,146790,163453,163458,141292,141295,141329,141339,141345,141355,141394,141463,141474,141297,141302,141310,141316,141319,141326,141337,141341,141354,141358,141362,141363,141384,141389,141407,141432,141433,141443,141455,141456,141457,141466,141467,141472,141478,133979,133974,133981,134012,134062,133860,133909,133919,133926,133930,133932,133933,133941,133942,133956,134000,134015,134018,134021,134024,134030,134047,134067,134074,134075,134077,134081,134082,134109,134110,134112,134118,134133,134136,134140,134141,133817,133834,133835,133848,133849,133874,133876,133896,133897,133901,133904,114450,114451,114457,114469,114475,114482,114485,114486,114494,114505,114516,114522,114528,114545,114550,114556,114564,114586,114588,114592,114598,114600,114613,114623,114631,114639,114648,114650,114652,114655,114656,114657,114663,163086,163092,162597,162603,100873,101417,101451,100598,100620,100686,100694,100697,100990,100995,101185,101228,101328,101361,101385,101405,101423,101445,101446,101454,101473,101496,101513,101514,101516,101519,101523,101530,101542,101545,101560,101573,101574,101583,101587,101588,101598,101605,101608,101609,101611,100478,100518,100535,100548,100549,100569,100586,100590,100599,100601,100616,100619,100630,100635,100642,100655,208677,100672,100689,100693,100699,100700,100702,100720,100731,100747,100756,100759,100767,100771,100772,100779,100786,100799,100812,100819,100843,100854,100860,100862,100867,100877,100890,100899,100907,100910,100941,100947,100977,100980,100981,100988,100992,100998,101000,101029,101032,101034,101048,101055,101066,101070,101084,101086,101092,101102,101118,101119,101121,101130,101132,101135,101147,101151,101155,101157,101164,101177,101178,101186,101192,101193,101202,101212,101221,101227,101247,101251,101259,101261,101268,101270,101272,101296,101303,101322,101326,101337,101341,101359,117924,118013,118024,118025,118077,118303,118487,118662,118702,118715,118720,118744,118751,118756,118761,118772,118777,118794,118801,118809,118810,118811,118821,118839,118843,118845,118851,118852,118854,118859,118893,118895,118896,118898,118902,118916,118918,118930,118934,118944,118954,118965,118975,118977,118979,118983,117879,117883,117885,117893,117900,117911,117915,117928,117931,117932,117938,117950,117954,117959,117972,117973,117980,117983,118019,118027,118034,118035,118067,118096,118103,118146,118150,118169,118189,118191,118195,118217,118236,118286,118297,118317,118385,118393,118395,118399,118407,118419,118423,118429,118433,118473,118479,118486,118488,118491,118494,118523,118529,118536,118550,118575,118586,118601,118617,118653,118665,118674,118676,118693,118695,118699,118712,118716,118740,118742,38371,38359,38356,38351,38344,38336,38333,38329,38327,38322,38310,38296,38260,38252,38239,38235,38232,38223,38219,38194,38182,38180,38163,38161,38149,38135,38122,38086,38082,38080,38073,38069,38058,38046,38039,38027,38014,38012,38011,38004,38001,37998,37977,37964,37936,37922,37918,37910,37909,37879,37874,37857,37853,37848,37832,37830,37826,37823,37777,37770,37769,37762,37741,37735,37725,37722,37721,37704,37702,37670,37667,37664,37645,37609,37606,37602,37591,37581,37577,37576,37575,37563,37562,37561,37554,37553,37537,37531,37526,37506,37502,37485,37479,37477,37470,37466,37464,37449,37434,37416,37411,37382,37367,37363,37362,37361,37357,37355,37346,37335,37312,37306,37290,37287,37279,37263,37261,37257,37250,37245,37239,37237,37231,37226,37213,37208,37204,37201,37191,37188,37163,37158,37149,37148,37146,37135,37129,37122,37081,37079,37059,37054,37045,37031,37023,36992,36991,36990,36981,36964,36962,36932,36923,36914,36911,36905,36888,36883,36869,36861,36857,36849,36840,36839,36827,36826,36825,36810,36795,36757,36742,36726,36702,36697,36694,36654,36652,36645,36632,36622,36609,36600,36599,36597,36587,36579,36560,36547,36512,36505,36502,36500,36498,36495,36492,36484,36482,36478,36473,36466,36449,36448,36433,36429,36427,36421,36417,36408,36397,36395,36385,36381,36368,36366,36357,36351,36348,36344,36343,36339,36336,36330,36329,36328,36327,36325,36323,36295,36285,36277,36273,36271,36268,36267,36264,36250,36236,36234,36231,36224,36220,36218,36215,36189,36183,36179,36169,36152,36142,36131,36125,36116,36106,36072,36067,36026,36013,36006,35973,35964,35959,35954,35953,35942,35940,35936,35928,35911,35904,35901,35898,35894,35891,35887,35880,35873,35862,35860,35853,35836,35834,35806,35803,35799,35795,35783,35777,35763,35762,35761,35753,35749,35733,35708,35703,35689,35686,35676,35670,35649,35641,35632,35628,35624,35622,35617,35608,35583,35577,35573,35559,35550,35549,35539,35535,35531,35522,35518,35512,35511,35510,35508,35501,35483,35482,35481,35466,35460,35454,35447,35404,35403,35396,35391,35381,35352,38419,38413,38412,38402,38398,38396,38315,38293,38191,38170,38098,38067,38013,37889,37888,37859,37680,37652,37578,37558,37520,37447,37436,37427,37281,37269,37160,37103,37097,37094,37061,36998,36966,36863,36834,36763,36734,36723,36551,35932,35930,35913,35534,35420,35971,165124,163874,157962,157969,157909,157915,157916,157922,157924,157930,157936,134234,134231,134216,134201,134193,134182,134175,134173,134145,134504,134501,134498,134497,134486,134485,134483,134477,134472,134471,134469,134464,134445,134444,134439,134437,134435,134414,134409,134399,134390,134380,134378,134377,134368,134349,134343,134335,134326,134309,134300,134288,134278,134272,134254,134247,134181,134155,134284,164937,159860,159869,159842,164342,164598,113425,113430,113187,113197,113217,113243,113251,113278,113282,113310,113351,113367,113386,113409,113411,113415,113431,113434,113436,113448,113451,113452,113463,113474,113475,113476,113477,113478,113480,113482,113489,113494,112846,112854,112864,112880,112881,112883,112884,112894,112897,112902,112905,112913,112926,112934,112936,112953,112954,112957,112981,112982,112988,112989,112991,112995,113005,113012,113019,113055,113061,113064,113065,113068,113073,113075,113091,113098,113099,113115,113119,113121,113122,113133,113135,113144,113151,113152,113161,113166,164058,164051,164050,164048,145408,145275,145297,145343,145365,145371,145373,145383,145386,145423,145427,164900,164806,165067,163941,165255,162780,162783,166276,159256,159236,159231,159229,159260,143703,143693,160881,160875,160906,160904,160901,160899,160884,163780,163776,164267,164270,164271,159299,159297,159290,159288,159286,159284,159274,159319,159306,159277,159272,165687,164036,164025,163752,154529,154524,154522,154518,154504,154502,154500,154499,154497,154483,154473,154469,154464,154456,154455,154445,154444,154428,154416,154406,154404,154401,154394,154390,154381,154561,154550,154549,154534,154435,143221,143219,143208,143196,143194,143190,143181,143174,143170,143166,143165,143164,143153,143148,143145,143135,143134,143133,143128,143126,143118,143109,143106,143100,143096,143083,143077,143075,143052,143222,143204,143124,143064,143080,165762,165511,163911,163920,165413,166245,166261,165503,3205,3201,3200,3177,3176,3161,3143,3135,3125,3121,3114,3091,3084,3080,3079,3068,3058,3048,3044,3035,3024,3019,3016,3012,2999,2984,2979,2978,2973,2969,2962,2956,2926,2921,2917,2906,2901,2887,2862,2849,2838,2828,2818,2815,2805,2787,2786,2782,2777,2764,2763,2760,2753,2751,2745,2735,2734,2723,2721,2718,2713,2703,2699,2692,2680,2673,2669,2658,2648,2646,2635,2634,2627,2620,2619,2618,2610,2609,2607,2601,2575,2573,2571,2569,2566,2559,2547,2537,2522,2521,2513,2501,2480,2478,2456,2454,2453,2447,2444,2441,2430,2428,2418,2415,2410,2408,2407,2399,2390,2377,2361,2360,2355,2352,2344,2341,2335,2334,2323,2321,2320,2315,2314,2313,2309,2303,2287,2284,2282,2275,2274,2273,2270,2260,2253,2243,2237,2209,2206,2205,2203,2202,2199,2183,2171,2157,2152,2144,2143,2142,2141,2138,2130,2129,2128,2127,2126,2120,2097,2096,2081,2077,2075,2067,2047,2042,2041,2040,2039,2034,2032,2024,2020,2005,2004,2001,2000,1996,1990,1986,1983,1980,1978,1976,1962,1960,1946,1944,1940,1936,1924,1918,1916,1912,1902,1898,1894,1888,1873,1872,1867,1866,1836,1835,1814,1808,1787,1786,1774,1764,1751,1740,1715,1707,1703,1698,1663,1652,1647,1639,1621,1615,1593,1589,1579,1573,1567,1549,1539,1521,1511,1503,1501,1472,1468,1466,1461,1447,1443,1438,1429,1419,1412,1406,1397,1396,1394,1392,1389,1371,1355,1344,1335,1329,1322,1312,1284,1267,1263,1231,1230,1225,1220,1194,1185,1168,1159,1155,1152,1144,1142,1134,1115,1102,1092,1066,1061,1052,1040,1039,1025,1022,1003,1000,998,997,992,989,981,967,961,956,952,927,916,910,907,901,886,882,880,871,865,836,828,815,811,805,799,783,778,762,760,756,746,744,721,690,688,680,673,663,648,645,644,624,622,610,596,595,584,582,578,577,576,566,563,562,545,535,531,529,527,502,498,489,486,466,456,455,444,438,428,427,424,417,405,397,396,390,388,365,364,315,286,284,280,271,263,252,233,222,219,216,211,210,193,185,155,154,145,140,139,138,135,133,129,127,107,94,89,87,85,74,70,54,50,41,32,15,7,6048,6038,6037,6035,6031,6020,6010,6004,5969,5957,5946,5937,5934,5930,5921,5910,5906,5903,5895,5890,5871,5849,5829,5821,5815,5810,5800,5793,5782,5773,5771,5770,5768,5763,5750,5744,5739,5731,5730,5729,5728,5711,5695,5693,5672,5661,5646,5641,5630,5629,5627,5624,5614,5612,5609,5604,5591,5590,5567,5554,5553,5551,5549,5540,5538,5531,5526,5525,5524,5515,5513,5493,5483,5468,5451,5440,5406,5402,5387,5369,5352,5350,5296,5294,5293,5291,5286,5281,5259,5243,5242,5241,5240,5230,5227,5223,5220,5210,5174,5170,5165,5160,5151,5150,5143,5132,5130,5117,5114,5110,5105,5104,5092,5088,5085,5077,5064,5045,5041,5026,5025,5020,5013,5007,5006,4999,4998,4997,4985,4982,4975,4969,4964,4963,4961,4948,4934,4933,4932,4910,4906,4905,4904,4901,4893,4892,4889,4871,4862,4841,4838,4831,4826,4816,4801,4796,4783,4777,4759,4754,4749,4736,4737,4730,4718,4710,4709,4705,4703,4702,4698,4695,4694,4693,4689,4685,4680,4673,4671,4663,4659,4653,4651,4629,4605,4604,4591,4576,4569,4559,4543,4541,4536,4519,4513,4497,4494,4489,4486,4484,4479,4476,4474,4473,4470,4461,4455,4445,4442,4426,4422,4418,4395,4368,4364,4338,4334,4323,4316,4298,4295,4294,4284,4280,4263,4227,4225,158631,158620,158608,158600,158593,158655,158642,128337,128331,128326,128325,128323,128322,128315,128306,128303,128274,128271,128253,128231,128220,128205,128182,128179,128634,128623,128607,128600,128593,128586,128572,128558,128557,128544,128534,128529,128520,128519,128510,128509,128503,128499,128497,128491,128487,128485,128483,128468,128458,128449,128417,128416,128409,128407,128405,128397,128383,128380,128372,128353,128347,128341,128339,128332,128298,128622,128613,128612,128611,128535,128501,145173,145266,145057,145062,145070,145079,145082,145088,145090,145091,145094,145110,145111,145114,145117,145118,145123,145129,145140,145144,145181,145182,145186,145194,145197,145198,145208,145212,145214,145219,145220,145221,145238,145245,130145,130120,130119,130102,130101,130076,130075,130070,130068,130066,130038,130034,130018,130014,130007,129998,129995,129987,129978,129968,129966,129956,129951,129945,129937,129936,129935,130641,130638,130632,130607,84731,84736,83214,83215,83231,83265,83291,83474,83532,83556,83678,83681,84002,84055,84158,84466,84528,84533,84598,84671,84673,84679,84683,84696,84697,84702,84704,84708,84719,84722,84728,84729,84738,84767,84778,84780,84784,84791,84792,84795,84797,83193,83194,83199,83203,83204,83209,83210,83226,83230,83234,83246,83247,83254,83258,83261,83287,83294,83295,83320,83324,83336,83338,83348,83349,83350,83354,83374,83376,83381,83382,83384,83389,83398,83407,83412,83416,83427,83429,83433,83434,83450,83462,83463,83482,83487,83495,83496,83512,83513,83515,83518,83519,83539,83540,83542,83565,83568,83569,83572,83578,83588,83597,83606,83609,83623,83659,83665,83673,83675,83696,83697,83703,83709,83712,83724,83725,83728,83734,83740,83744,83751,83755,83756,83762,83767,83775,83776,83777,83779,83787,83805,83812,83835,83846,83848,83858,83861,83864,83869,83874,83875,83882,83885,83886,83888,83890,83891,83903,83909,83910,83913,83916,83917,83921,83922,83925,83926,83931,83932,83949,83950,83951,83969,83977,83983,83996,84004,84006,84019,84022,84027,84028,84036,84045,84046,84051,84052,84053,84056,84060,84062,84067,84070,84078,84079,84082,84083,84093,84104,84106,84107,84111,84119,84120,84123,84135,84137,84142,84152,84153,84164,84167,84169,84180,84191,84197,84202,84206,84211,84212,84220,84222,84233,84234,84235,84236,84249,84259,84261,84269,84286,84296,84299,226529,84301,84304,84308,84309,84311,84314,84316,84319,84321,84324,84334,84340,84343,84346,84349,84360,84367,84371,84376,84377,84390,84393,84423,84428,84431,84435,84439,84443,84449,84464,84484,84490,84502,84512,84516,84520,84521,84536,84546,84548,84554,84555,84556,84557,84563,84567,84573,84580,84599,84618,84625,84628,84630,84631,84637,84646,149873,149877,149881,149890,157681,157670,157669,166050,150106,149941,149975,149977,149982,149994,150005,150006,150040,150048,150050,150084,160806,160802,160834,160830,160828,160818,160829,166193,160051,160078,160075,166135,160514,160526,153960,153916,153969,153967,163167,163155,150578,150576,150560,150558,150556,150554,150541,150535,150533,150520,150511,150506,150504,150494,150490,150596,150593,150592,150540,150499,150481,166148,139777,139774,139767,139763,139740,139735,139731,139727,139723,139708,139707,139704,139694,139693,139690,139688,139665,139659,139655,139654,139642,139637,139631,139622,139620,139615,139606,139602,139601,139596,139585,139573,139572,139569,139560,139753,139751,139743,139658,139652,139581,160662,67417,68033,68452,68486,66291,66445,66460,66480,66573,66707,67106,67107,67216,67258,67412,67433,67553,67830,67843,67897,67934,68147,68169,68191,68263,68319,68353,68433,68434,68469,68483,66287,66310,66323,66324,66326,66330,66334,66337,66338,66350,66351,66353,66355,66357,66364,66366,66404,66411,66412,66418,66419,66426,66434,66440,66442,66444,66474,66501,66502,66514,66521,66525,66530,66535,66538,66560,66561,66562,66563,66566,66576,66581,66592,66594,66609,66615,66618,66623,66635,66642,66649,66659,66660,66664,66679,66683,66694,66718,66730,66731,66736,66755,66769,66772,66777,66780,66781,66784,66794,66797,66805,66813,66833,66837,66840,66857,66863,66865,66876,66899,66903,66908,66916,66924,66929,66930,66942,66945,66950,66955,66961,66963,66970,66978,66979,66986,66989,66991,67000,67011,67013,67020,67022,67027,67028,67038,67050,67053,67055,67070,67076,67079,67088,67098,67109,67111,67117,67124,67130,67137,67149,67151,67153,67164,67178,67197,67211,67220,67228,67245,67249,67251,67253,67271,67272,67277,67278,67328,67329,67333,67334,67335,67338,67342,67345,67375,67378,67379,67395,67407,67424,67425,67436,67441,67442,67443,67447,67456,67471,67472,67492,67496,67506,67507,67517,67518,67519,67523,67548,67559,177583,67567,67580,67614,67618,67636,67664,67667,67668,67682,67703,67713,67723,67728,67732,67744,67751,67754,67756,67757,67759,67771,67778,67781,67795,67797,67802,67805,67808,67826,67836,67838,67840,67844,67857,67858,67864,67872,67889,67891,67902,67923,67930,67939,67942,67953,67966,67969,67976,67983,67985,67986,67990,68000,68009,68010,68012,68017,68024,68026,68034,68057,68076,68088,68090,68092,68098,68100,68125,68127,68129,68136,68141,68143,68149,68151,68157,68162,68171,68180,68183,68207,68249,68250,68268,68283,68308,68321,68326,68329,68333,68339,68352,68356,68370,68391,68392,68399,68403,68411,50785,50781,50729,50714,50696,50695,50659,50649,50648,50618,50611,50610,50601,50591,50588,50576,50574,50566,50550,50545,50536,50515,50464,50448,50439,50420,50417,50409,50407,50402,50396,50386,50385,50347,50328,50322,50319,50318,50305,50302,50301,50299,50276,50267,50263,50242,50233,50227,50208,50186,50174,50147,50144,50121,50120,50098,50096,50090,50081,50068,50050,50044,50041,50032,50008,50000,49995,49984,49978,49971,49965,49962,49961,49941,49936,49931,49914,49909,49903,49878,49876,49872,49858,49847,49846,49830,49814,49811,49782,49780,49779,49775,49774,49771,49763,49754,49753,49752,49744,49732,49731,49726,49712,49704,49693,49690,49683,49677,49673,49671,49669,49666,49651,49634,49620,49606,49604,49592,49587,49581,49579,49576,49567,49554,49545,49541,49533,49532,49518,49488,49484,49482,49475,49473,49469,49466,49445,49435,49418,49404,49399,49394,49375,49346,49336,49332,49304,49290,49287,49266,49265,49262,49254,49245,49238,49237,49227,49219,49214,49206,49203,49196,49191,49190,49178,49168,49159,49136,49127,49123,49121,49117,49114,49113,49102,49094,49078,49060,49053,49044,49032,49031,49024,49013,49009,48999,48987,48980,48964,48962,48955,48953,48952,48943,48930,48904,48893,48891,48890,48876,48867,48857,48842,48822,48804,48803,48799,48798,48795,48787,48777,48767,48766,48762,48755,48749,48748,48732,48725,48724,48723,48680,48679,48677,48665,48657,48651,48647,48646,48639,48628,48625,48623,48608,48606,48603,48599,48597,48593,48592,48553,48538,48535,48525,48508,48500,48482,48473,48460,48459,48457,48454,48453,48445,48441,48438,48436,48431,48430,48429,48428,48425,48422,48421,48418,48403,48401,48399,48384,48371,48355,48352,50914,50909,50897,50896,50890,50889,50879,50873,50862,50831,50830,50828,50822,50533,50427,50039,50038,50037,50036,49985,49958,49719,49474,49384,49381,49361,49164,48944,48927,166305,13536,13530,13528,13517,13514,13512,13509,13507,13494,13489,13487,13484,13481,13470,13467,13447,13430,13427,13424,13417,13404,13399,13397,13395,13378,13374,13368,13365,13356,13348,13338,13335,13307,13300,13287,13281,13277,13265,13255,13253,13246,13241,13240,13232,13231,13222,13221,13207,13206,13201,13193,13180,13173,13169,13166,13154,13138,13137,13130,13127,13105,13082,13035,13034,13008,12993,12983,12982,12946,12945,12943,12932,12930,12928,12921,12914,12908,12891,12881,12873,12871,12868,12863,12854,12852,12848,12847,12840,12836,12835,12830,12823,12819,12817,12813,12798,12797,12795,12794,12788,12768,12766,12758,12748,12744,12741,12740,12736,12730,12717,12710,12707,12685,12679,12677,12675,12660,12656,12652,12634,12629,12620,12617,12602,12600,12598,12586,12585,12569,12565,12558,12554,12551,12549,12540,12539,12538,12537,12534,12525,12520,12518,12515,12513,12511,12502,12486,12480,12475,12470,12465,12439,12437,12436,12430,12415,12414,12404,12391,12388,12385,12369,12367,12366,12341,12339,12325,12313,12309,12298,12288,12285,12282,12275,166238,153497,153473,153470,153469,153460,153454,153452,153449,153448,138882,138876,138873,138870,138869,139164,139139,139138,139116,139110,139102,139078,139061,139045,139044,139042,139041,139035,139025,139015,138999,138976,138975,138969,138968,138965,138964,138961,138951,138931,138930,138924,138907,139029,164913,112628,112623,112614,112598,112586,112571,112569,112557,112544,112532,112531,112529,112528,112514,112508,112503,112502,112501,112500,112498,112487,112482,112468,112465,112464,112458,112457,112449,112443,112439,112428,112426,112423,112394,112385,112376,112370,112362,112348,112346,112345,112342,112328,112303,112298,112286,112266,112265,112237,112230,112215,112214,112210,112209,112196,112192,112182,112180,112173,112164,112163,112161,112160,112159,112147,112146,112143,112142,112139,112138,112133,112122,112118,112117,112107,112105,112104,112103,112096,112084,112079,112069,112060,112058,112056,112044,112043,112040,112034,112022,112021,112020,112012,112010,112009,112003,111998,111989,111983,111964,111961,111955,111954,111948,111943,111933,111914,111897,111889,111888,111858,111846,111842,111841,111830,111802,112823,112814,112808,112801,112793,112765,112746,112691,112678,112677,112674,112673,112665,112656,112648,112436,112415,112347,112150,112076,111886,112086,112049,151032,151017,151005,151003,150988,150973,150960,150958,150957,150941,150935,150934,150932,150928,150919,150902,150875,150872,150867,150868,150864,150861,150860,150845,150833,150831,150776,150773,150741,150740,151048,150952,104523,104583,103754,103803,103877,103948,103961,104237,104402,104411,104414,104424,104442,104445,104448,104454,104457,104460,104465,104477,104480,104488,104489,104491,104497,104501,104503,183995,104506,104516,104522,104534,104567,104575,104580,104588,104593,104601,104610,104614,104615,104616,104618,104619,104623,104628,104631,104644,104645,104652,104655,104656,103724,103731,103732,103734,103735,103738,103745,103751,103752,103768,103769,103773,103774,103777,103801,103805,103808,103815,103820,103824,103827,103829,103830,103833,103837,103860,103867,103878,103889,103892,103918,103924,103933,103941,103945,103950,103982,103986,103988,103999,104001,104007,104021,104022,104032,104035,104050,104052,104059,104060,104066,104078,104088,104101,104105,104138,104143,104144,104150,104156,104160,104163,104164,104186,104191,104193,104195,104202,104204,104208,104212,104217,104219,104221,104224,104229,104259,104262,104267,104274,104280,104282,104290,104301,104308,104310,104327,104329,104333,104337,104362,104391,104400,104403,143881,143866,143865,143863,143828,143825,143816,143813,143808,143801,143769,143727,165446,151212,151217,151222,151242,151246,151261,151267,151271,151274,151324,151331,151335,151341,151375,151382,151394,62053,62045,62018,61995,61959,61931,61924,61923,61852,61838,61828,61818,61811,61803,61777,61769,61753,61728,61712,61706,61702,61701,61685,61683,61660,61658,61657,61633,61605,61599,61597,61585,61580,61568,61519,61500,61494,61487,61486,61477,61450,61445,61439,61418,61416,61409,61408,61393,61387,61377,61367,61366,61361,61354,61341,61328,61300,61234,61233,61217,61215,61214,61203,61199,61181,61180,61172,61159,61147,61142,61130,61110,61097,61093,61090,61081,61073,61067,61059,61056,61054,61049,61045,61034,61031,61021,61000,60992,60976,60974,60964,60963,60959,60952,60935,60934,60923,60912,60910,60907,60884,60881,60872,60829,60821,60807,60806,60802,60797,60790,60779,60778,60770,60761,60724,60711,60698,60695,60685,60672,60666,60651,60650,60647,60623,60622,60618,60612,60604,60599,60575,60563,60534,60522,60519,60515,60506,60502,60484,60479,60468,60465,60454,60440,60439,60437,60435,60431,60426,60424,60403,60401,60391,60387,60383,60366,60356,60349,60342,60337,60335,60332,60331,60322,60316,60309,60282,60280,60269,60260,60243,60241,60216,60204,60195,60194,60175,60173,60166,60163,60161,60156,60154,60133,60132,60121,60115,60104,60103,60090,60089,60066,60065,60057,60049,60048,60047,60032,60017,59994,59993,59990,59988,59973,59957,59916,59906,59905,59903,59874,59863,59848,59847,59815,59808,59806,62153,62150,62146,62124,62120,62116,62113,62100,62089,62068,62036,62030,61882,61871,61813,61688,61662,61590,61478,61457,61438,61263,61229,61219,61165,60795,60763,60629,60537,60450,60233,60230,60072,59932,59883,59836,59804,62143,60045,150357,150311,150354,131821,131828,131829,131869,131874,131881,131903,131906,131927,131929,131938,131049,131054,131062,131076,131096,131110,131121,131122,131139,131168,131172,131174,131210,131223,131237,131238,131246,131259,131327,131330,131354,131366,131370,131393,131422,131433,131457,131459,131460,131471,131476,131489,131514,131515,131519,131520,131529,131566,131580,131581,131606,131607,131614,131623,131631,131634,131640,131656,131662,131666,131673,131682,131686,131689,131707,131708,131717,131726,131727,165899,62309,62677,63362,63795,64059,64083,64101,62313,62317,62382,62436,62563,62566,62601,62768,62874,62903,62965,63090,63122,63269,63366,63411,63427,63455,63456,63476,63529,63536,63556,63640,63898,63986,64010,64016,64021,64034,64039,64044,64050,64076,64094,62173,62178,62191,62201,62215,62217,62226,62237,62248,62255,62258,62272,62277,62278,62281,62289,62291,62292,62306,62308,62320,62337,62339,62351,62354,62358,62362,62378,62386,62390,62392,62396,62424,62426,62433,62435,62438,62451,62450,62453,62467,62468,62475,62476,62497,62504,62514,62516,62528,62530,62539,62541,62564,62577,62584,62591,62593,62639,62641,62649,62669,62694,62695,62697,62701,62710,62726,62730,62735,62740,62764,62765,62771,62780,62782,62792,62795,62797,62799,62802,62818,62821,62823,62826,62830,62842,62848,62858,62865,62873,62878,62894,62897,62921,62922,62923,62945,62949,62988,62991,62998,63000,63008,63012,63019,63020,63022,63026,63027,63029,63030,63033,63036,63038,63039,63044,63069,63071,63095,63098,63102,63111,63113,63123,63127,63129,63135,63141,63153,63155,63159,63163,63169,63170,63176,63177,63178,63180,63189,63191,63208,63229,63230,63233,63234,63236,63240,63256,63275,63284,63296,63307,63308,63313,63327,63332,63353,63354,63382,63390,63391,63416,63419,63422,63426,63430,63433,63435,63438,63441,63442,63449,63451,63459,63469,63471,63475,63481,63487,63488,63490,63491,63495,63499,63511,63517,63518,63528,63532,63534,63537,63545,63549,63557,63564,63567,63578,63588,63592,63595,63597,63605,63609,63611,63625,63627,63628,63629,63633,63638,63642,63647,63662,63664,63668,63675,63677,63686,63693,63698,63701,63703,63716,63717,63720,63723,63749,63754,63756,63760,63775,63789,63804,63810,63814,63823,63827,63836,63841,63842,63849,63856,63860,63863,63865,63872,63885,63891,63895,63901,63908,63911,63923,63925,63928,63936,63939,63945,63948,63956,165680,165764,156212,156169,156180,156233,156133,156134,156141,156149,156148,156151,156152,156153,156154,156157,156162,156164,156168,156170,156181,156185,156190,156199,156201,156203,156206,156274,156265,156252,156237,156236,156310,156301,156290,156287,159563,165711,116051,116278,116406,116070,116112,116113,116212,116270,116276,116287,116290,116292,116298,116329,116339,116347,116354,116363,116370,116372,116373,116376,116379,116383,116384,116397,116411,116414,116416,116418,116049,116059,116069,116073,116078,116091,116117,116123,116126,116135,116137,116139,116151,116154,116161,116165,116168,116169,116176,116178,116187,116192,116207,116209,116215,116217,116222,116233,116235,116239,116249,116266,162088,162330,163736,153183,153161,153139,153125,153205,165513,157225,157208,157205,157197,157192,157180,157173,157243,157242,157239,157219,157200,162699,162697,146763,146745,146716,146711,146704,146685,146682,146681,146663,146660,146659,146646,146643,146638,146635,146633,146614,146610,146609,146608,146598,146596,146581,146594,164209,122850,122836,122826,122814,122810,122803,122800,122799,122796,122795,122793,122783,122779,122778,122766,122759,122755,122745,122741,122738,122722,122720,122710,122700,122691,122678,122677,122656,122654,122639,122634,122618,122606,122603,123093,123086,123083,123073,123055,123050,123046,123045,123037,123033,123032,123029,123028,123027,123019,123014,122998,122993,122989,122985,122977,122975,122969,122958,122957,122934,122930,122913,122901,122893,122860,122809,123057,122995,122933,150253,150136,150146,150157,150159,150160,150165,150171,150175,150182,130391,130368,130357,130347,130338,130331,130320,130312,130310,130304,130303,130273,130250,130245,130236,130233,130216,130201,130197,130196,130195,130191,130185,130178,147347,147331,147318,147302,147299,147295,147293,147290,147253,147244,147243,147240,147226,147221,147210,147357,147354,147310,147351,123593,123418,123426,123431,123349,123357,123366,123385,123388,123392,123396,123408,123420,123424,123425,123435,123445,123457,123476,123485,123490,123495,123497,123504,123509,123526,123531,123538,123542,123543,123556,123559,123564,123572,123586,123098,123108,123109,123133,123152,123158,123159,123187,123202,123205,123206,123219,123225,123235,123238,123254,123280,123281,123284,123307,123313,123326,123333,155804,155772,155767,155752,155727,155814,141241,141280,141004,141010,141011,141028,141043,141044,141065,141076,141078,141106,141112,141120,141130,141132,141133,141139,141143,141170,141173,141175,141184,141186,141194,141201,141207,141218,141226,141228,141233,141240,141242,141246,141252,141255,141261,141264,141266,141274,155683,155661,155676,155690,155693,155700,155582,155583,155584,155585,155591,155602,155605,155615,155617,155665,155668,155671,134584,134620,134727,134863,134577,134578,134592,134596,134598,134608,134617,134618,134659,134677,134679,134680,134683,134687,134691,134692,134697,134700,134701,134713,54319,54022,56433,56581,56978,53905,54127,54132,54141,54150,54175,54283,54436,54604,54659,54666,54941,54949,54981,55041,55131,55163,55192,55240,55318,55373,55382,55408,55592,55664,55773,55823,55850,55873,55915,55917,55985,56025,56031,56179,56258,56397,56615,56617,56636,56774,56825,56858,56903,56963,56966,57041,57048,57050,53899,53906,53907,53908,53913,53917,53920,53923,53924,53934,53948,53951,53955,53966,53968,53971,53985,53988,54011,54020,54024,54034,54038,54050,54054,54071,54076,54077,54081,54105,54107,54112,54128,54129,54130,54131,54137,54149,54153,54160,54180,54181,54186,54187,54192,54208,54213,54218,54230,54234,54239,54245,54250,54253,54254,54255,54256,54257,54293,54301,54311,54318,54332,54334,54347,54348,54358,54372,54374,54378,54388,54399,54400,54405,54442,54447,54456,54458,54480,54489,54499,54501,54527,54537,54538,54556,54563,54570,54590,54601,54608,54612,54635,54652,54669,54685,54690,54703,54706,54718,54720,54721,54732,54740,54748,54765,54770,54771,54779,54787,54788,54826,54830,54839,54843,54861,54875,54878,54884,54890,54895,54909,54911,54917,54922,54927,54930,54935,54950,54952,54959,54961,54978,55003,55022,55030,55044,55062,55066,55068,55072,55081,55100,55103,55113,55123,55132,55139,55142,55146,55151,55154,55194,55200,55204,55230,55232,55233,55244,55246,55258,55295,55329,55338,55348,55353,55379,55383,55402,55413,55420,55430,55443,55450,55454,55457,55476,55480,55481,55484,55499,55532,55535,55537,55543,55546,55548,55553,55556,55557,55558,55561,55562,55570,55590,55595,55605,55662,55671,55705,55711,55716,55719,55730,55760,55788,55794,55801,55803,55807,55812,55833,55838,55853,55860,55864,55865,55871,55894,55898,55904,55916,55930,55935,55940,55951,55962,55963,55967,55974,55981,55982,55998,56017,56033,56036,56045,56054,56067,56072,56082,56095,56106,56115,56150,56168,56206,56214,56216,56219,56237,56244,56280,56282,56295,56296,56297,56303,56308,56334,56379,56382,56384,56386,56399,56405,56414,56420,56425,56430,56438,56440,56441,56456,56463,56470,56475,56485,56488,56493,56511,56518,56527,56551,56564,56570,56609,56611,56614,56623,56629,56630,56659,56663,56664,56672,56687,56688,56701,56722,56727,56728,56729,56740,56758,56764,56773,56777,56791,56793,56813,56816,56818,56819,56824,56828,56831,56832,56837,56842,56872,56876,56895,56900,56915,56921,56922,56939,56962,56964,56992,56994,56995,56998,57001,57010,57016,57025,164956,126773,126870,126888,126920,126712,126737,126744,126753,126758,126760,126777,126784,126788,126792,126800,126803,126810,126815,126820,126826,126829,126840,126842,126845,126848,126854,126857,126873,126880,126903,126923,126927,126935,126937,126943,126954,126977,126985,126987,126989,126996,127016,127023,126521,126534,126536,126539,126542,126543,126549,126559,126570,126582,126597,126601,126604,126605,126613,126617,126627,126628,126630,126632,126637,126652,126655,126657,126667,126674,126677,126691,126694,126699,126702,121129,121042,121043,121044,121076,120855,120883,120964,120968,120985,121001,121010,121017,121023,121028,121032,121039,121045,121054,121065,121066,121077,121082,121118,121124,121126,121128,121134,121144,121146,121149,121150,121168,121178,121187,121188,121191,121192,121201,121209,121213,121215,121220,121227,121230,121232,121236,121237,121244,121251,121254,120690,120692,120698,120704,120723,120750,120751,120772,120812,120815,120818,120819,120822,120826,120831,120837,120842,120850,120851,120863,120867,120868,120872,120877,120881,120882,120895,120897,120899,120908,120916,120917,120924,120925,120927,120949,120957,165739,159961,160013,159954,159958,159973,159974,159978,159984,159989,165812,165300,137647,137642,137639,137636,137618,137608,137595,137590,137580,137574,137572,137567,137566,137533,137527,137525,137508,137503,137497,137480,137473,137470,137469,137468,137452,137435,137432,137429,137422,137398,137396,137384,137379,137367,137366,137365,137362,137359,137804,137801,137799,137784,137779,137769,137768,137762,137757,137756,137754,137749,137747,137743,137741,137740,137733,137731,137729,137727,137725,137720,137715,137712,137709,137698,137695,137689,137680,137667,137664,137659,137537,137518,137395,137753,137705,137699,137660,149085,149061,149062,149075,149084,163807,163809,148741,148722,148706,148702,148696,148695,148685,148673,164878,165212,163332,155095,155202,155206,155087,155088,155101,155103,155111,155113,155121,155125,155129,155154,155155,155174,155181,159012,159016,159018,159062,159077,159003,159019,159028,159031,159034,159044,160262,160269,160243,154344,154342,154304,154298,154265,154372,154357,160940,159692,159687,159680,159669,159713,159706,153410,153409,153393,153387,153375,153371,153512,153511,153374,142457,142449,142446,142444,142432,142429,142423,142413,142406,142383,190570,142368,142364,142349,142344,142305,142304,142301,142298,142296,142288,142287,142279,142275,142253,165416,162138,69867,68503,68520,68548,68556,68672,68880,68937,68949,68966,69001,69061,69113,69135,69236,69480,69490,69502,69526,69555,69623,69624,69698,69734,69765,69830,70055,70057,70059,70064,70070,70072,70078,70086,70088,70093,70094,70101,70122,70124,70128,70134,70135,70136,70141,70147,70150,70160,70168,70169,70176,70177,70178,70182,70181,70185,70192,70204,70206,70209,70215,70218,70220,70222,68504,68508,68509,68511,68529,68534,68542,68552,68557,68570,68572,68576,68581,68582,68583,68588,68590,68598,68611,68614,68617,68618,68620,68635,68637,68639,68652,68681,68691,68695,68703,68709,68716,68718,68725,68734,68746,68757,68761,68764,68765,68773,68786,68790,68791,68818,68821,68827,68839,68845,68846,68848,68859,68865,68869,68883,68887,68893,68903,68905,68908,68920,68926,68933,68938,68942,68956,68957,68963,68969,68984,68985,69002,69009,69023,69025,69031,69033,69034,69051,69057,69066,69076,69080,69095,69101,69106,69115,69136,69138,69153,69165,69168,69181,69195,69213,69214,69229,69240,69261,69277,69286,69303,69314,69324,69327,69339,69342,69350,69357,69360,69372,69384,69389,69402,69415,69416,69423,69442,69443,69451,69465,69469,69492,69498,69499,69523,69525,69531,69544,69565,69573,69576,69577,69582,69584,69585,69592,69597,69604,69607,69612,69619,69622,69627,69633,69636,69641,69645,69646,69649,69650,69658,69664,69675,69697,69702,69710,69711,69714,69729,69730,69749,69753,69758,69761,69764,69775,69778,69779,69784,69798,69802,69804,69808,69814,69827,69838,69843,69848,69851,69855,69871,69886,69889,69898,69904,69905,69908,69909,69919,69920,69925,69934,69945,69955,69956,69959,69962,69968,69969,69975,69976,69982,69983,69989,69990,70000,70006,70009,70020,70028,70031,70038,70041,162238,162225,162226,162211,147439,147470,147563,147569,147573,147374,147379,147382,147384,147390,147393,147402,147414,147415,147421,147425,147432,147441,147452,147454,147455,147468,147474,147502,147506,147515,147519,147530,147539,147546,147553,147558,147560,147561,147562,18349,18448,19833,19834,19961,19975,16994,17250,17304,17898,17949,18046,18107,18177,18504,18519,18612,18638,18640,18648,18989,19112,19151,19172,19472,19490,19496,19516,19747,19890,19902,19903,19925,19939,19971,20040,20056,20058,20102,20186,20202,20396,20450,20498,20634,20733,20786,20928,21014,21033,21154,21171,16949,16950,17096,17214,17220,17228,17255,17269,17360,17677,17799,17809,17812,17821,17835,17837,17838,17863,17864,17872,17878,17882,17893,17911,17920,17928,17933,17936,17946,17952,17959,17992,18001,18016,18025,18030,18045,18054,18079,18080,18087,18101,18104,18106,18116,18135,18165,18183,18209,18217,18218,18220,18239,18241,18247,18249,18255,18267,18269,18271,18278,18279,18280,18285,18286,18287,18288,18310,18315,18316,18321,18325,18331,18338,18346,18351,18366,18377,18379,18394,18399,18400,18405,18415,18422,18435,18449,18459,18467,18477,18482,18485,18491,18496,18522,18523,18526,18528,18530,18532,18565,18570,18583,18598,18618,18627,18632,18633,18655,18657,18663,18668,18719,18728,18733,18759,18765,18785,18803,18813,18815,18841,18842,18883,18889,18890,18901,18912,18925,18930,18935,18944,18945,18953,18959,18970,18972,18991,18995,18996,19000,19003,19011,19025,19035,19043,19049,19051,19067,19085,19098,19110,19135,19141,19145,19148,19162,19182,19184,19190,19222,19229,19237,19243,19269,19271,19282,19285,19289,19298,19299,19304,19308,19312,19329,19335,19339,19348,19352,19357,19372,19374,19377,19382,19386,19387,19395,19401,19407,19409,19417,19422,19423,19437,19440,19442,19444,19464,19477,19494,19503,19507,19524,19536,19540,19543,19544,19549,19552,19564,19566,19571,19590,19595,19615,19632,19652,19671,19678,19680,19687,19699,19708,19730,19744,19754,19777,19778,19798,19806,19812,19818,19819,19820,19821,19828,19831,19872,19876,19879,19889,19897,19915,19931,19934,19943,19944,19954,19958,19965,19985,19999,20007,20009,20024,20034,20039,20055,20066,20072,20077,20093,20101,20103,20104,20157,20159,20164,20168,20201,20206,20222,20225,20226,20234,20235,20238,20241,20244,20260,20267,20268,20277,20288,20289,20324,20340,20347,20348,20350,20356,20362,20376,20378,20421,20426,20444,20473,20480,20493,20503,20509,20515,20517,20518,20521,20524,20533,20541,20542,20545,20547,20561,20565,20572,20588,20593,20611,20616,20618,20619,20635,20644,20645,20651,20652,20654,20664,20676,20685,20696,20700,20715,20726,20730,20734,20744,20745,20758,20763,20768,20782,20784,20789,20793,20808,20809,20815,20817,20824,20825,20828,20833,20862,20871,20874,20883,20884,20885,20888,20891,20896,20907,20910,20913,20915,20926,20933,20936,20950,20959,20964,20968,20978,20987,20990,20994,20996,20997,20999,21008,21013,21015,21018,21034,21043,21063,21069,21071,21073,21075,21080,21090,21091,21093,21094,21102,21119,21123,21127,21132,21134,21140,21142,21149,21165,21167,21179,21180,21186,21189,21192,21199,21202,16933,16948,16951,16952,16963,16971,16976,16984,16991,17006,17018,17023,17033,17035,17036,17053,17075,17078,17083,17095,17105,17110,17112,17113,17114,17116,17142,17147,17155,17166,17178,17183,17186,17188,17191,17194,17204,17217,17224,17252,17264,17274,17278,17279,17281,17299,17305,17306,17313,17317,17322,17329,17333,17347,17351,17353,17376,17378,17388,17403,17416,17419,17425,17433,17443,17446,17480,17483,17485,17486,17487,17494,17509,17511,17514,17526,17531,17545,17548,17553,17562,17566,17569,17582,17586,17593,17598,17605,17606,17609,17612,17626,17642,17651,17661,17662,17672,17686,17687,17688,17694,17711,17713,17716,17726,17727,17742,17753,17756,17757,17766,17798,160697,160715,160716,160723,160698,160708,160709,150463,150459,150452,150445,150443,150440,150436,150421,150412,150409,150398,150390,150386,150384,150469,150437,150382,165927,151974,151977,151979,151985,151998,152008,152012,152021,152023,152026,152029,152032,152040,165544,150725,150603,150606,150616,150617,150625,150635,150636,150643,150645,150660,150663,150666,150682,150685,150707,150711,150712,150717,150722,150731,164915,147950,147921,147914,147911,147905,147902,147901,147896,147895,147890,147884,147877,147874,147867,147855,147830,147825,147772,147767,147756,147750,147845,165751,164377,160448,160440,160432,160458,128778,128727,128761,128776,128780,128789,128790,128805,128806,128810,128827,128833,128834,128838,128841,128842,128849,128851,188198,128864,128865,128882,128886,128640,128645,128675,128699,128710,128713,128716,128721,128725,128726,128736,128754,159725,159721,159727,152686,152655,152639,152637,152632,152616,152614,152586,152685,152696,129655,129654,129653,129649,129645,129643,129625,129620,129618,129617,129605,129590,129589,129585,129578,129573,129553,129552,129549,129548,129539,129537,129532,129526,129925,129922,129909,129907,129906,129905,129903,129901,129895,129893,129884,129882,129877,129876,129870,129866,129853,129852,129835,129815,129814,129812,129809,129808,129801,129799,129793,129788,129784,129782,129776,129772,129768,129749,129748,129747,129703,129669,129663,129639,129632,129600,129920,129698,129623,129609,151648,151617,151613,151602,151601,151598,151594,151572,151569,151560,151557,151661,166110,165957,22130,22118,22114,22113,22107,22103,22095,22091,22074,22046,22041,22028,22026,22022,22021,22013,22006,21998,21992,21991,21990,21985,21984,21979,21961,21954,21950,21946,21928,21927,21918,21917,21915,21906,21905,21898,21854,21851,21843,21841,21840,21831,21827,21816,21809,21806,21797,21785,21782,21781,21778,21774,21766,21757,21752,21747,21741,21740,21733,21729,21728,21722,21711,21708,21702,21691,21674,21672,21670,21664,21654,21653,21625,21619,21614,21612,21600,21594,21589,21585,21581,21573,21572,21571,21565,21563,21548,21545,21543,21541,21538,21534,21528,21516,21507,21501,21494,21492,21491,21471,21468,21467,21461,21457,21456,21442,21440,21436,21435,21422,21419,21414,21413,21390,21383,21382,21376,21368,21365,21364,21362,21361,21360,21345,21332,21329,21325,21317,21316,21306,21303,21258,21256,21242,21241,21233,21224,21218,25323,25313,25300,25297,25282,25279,25262,25260,25258,25250,25230,25228,25224,25223,25220,25203,25198,25196,25195,25184,25168,25139,25137,25117,25110,25098,25093,25084,25079,25072,25059,25042,25041,25033,25028,25023,25020,25017,25016,25013,25009,25001,24997,24995,24989,24975,24971,24962,24959,24940,24933,24923,24920,24915,24905,24903,24888,24887,24877,24867,24851,24822,24821,24815,24811,24791,24790,24783,24773,24771,24762,24754,24708,24707,24705,24702,24680,24679,24676,24671,24658,24627,24623,24620,24608,24595,24593,24586,24578,24574,24570,24557,24555,24554,24553,24543,24533,24528,24525,24517,24512,24507,24494,24456,24455,24441,24437,24435,24432,24427,24425,24421,24395,24393,24386,24379,24377,24374,24366,24358,24349,24339,24338,24330,24314,24312,24310,24308,24293,24284,24279,24275,24264,24263,24250,24246,24244,24232,24224,24216,24185,24171,24162,24135,24130,24115,24113,24090,24085,24082,24071,24056,24051,24047,24046,24041,24031,24027,24018,24013,23989,23987,23978,23976,23975,23955,23951,23948,23938,23933,23931,23926,23921,23912,23909,23905,23902,23900,23883,23867,23865,23862,23857,23856,23851,23813,23787,23780,23778,23767,23766,23743,23732,23726,23721,23715,23668,23664,23662,23633,23628,23623,23618,23605,23592,23586,23583,23582,23566,23560,23538,23532,23527,23514,23507,23461,23457,23450,23436,23412,23411,23400,23398,23379,23367,23350,23349,23345,23336,23331,23320,23318,23315,23313,23303,23298,23291,23275,23274,23263,23235,23220,23215,23211,23200,23195,23182,23173,23172,23169,23168,23166,23160,23159,23155,23141,23129,23125,23112,23097,23096,23074,23071,23039,23025,23021,23011,22997,22982,22980,22979,22964,22957,22933,22931,22930,22909,22897,22851,22830,22807,22790,22782,22780,22774,22765,22756,22746,22743,22742,22729,22719,22713,22704,22701,22699,22689,22679,22649,22640,22637,22621,22610,22600,22584,22571,22565,22560,22559,22556,22554,22543,22527,22519,22501,22493,22472,22471,22468,22461,22443,22435,22430,22429,22419,22401,22380,22371,22370,22369,22367,22363,22347,22339,22337,22330,22326,22315,22314,22308,22289,22275,22263,22260,22254,22242,22241,22240,22239,22238,22217,22203,22194,22191,22182,22181,22169,22164,22159,22147,22140,22138,22133,21947,21849,21813,21707,21590,21542,21527,21476,21385,21377,21290,21278,25307,25249,25236,25102,25049,24979,24970,24893,24862,24789,24711,24662,24606,24273,24235,24195,24108,24011,23854,23842,23679,23672,23667,23494,23489,23462,23335,23283,23251,23111,23018,23016,22904,22873,22695,22670,22562,22541,22531,22529,22378,22354,22322,22313,22294,22280,22278,22198,22137,24032,23639,22961,22959,22259,166279,164726,117506,117498,117496,117490,117476,117473,117470,117469,117451,117437,117413,117412,117402,117399,117396,117395,117391,117378,117355,117351,117348,117342,117339,117338,117325,117319,117318,117315,117311,117288,117282,117274,117266,117253,117238,117224,117210,117207,117206,117203,117185,117183,117166,117162,117160,117157,117145,117141,117135,117849,117845,117844,117833,117827,117825,117803,117791,117784,117776,117775,117760,117744,117740,117731,117717,117695,117693,117679,117674,117672,117669,117663,117658,117642,117640,117639,117637,117631,117625,117623,117622,117617,117616,117597,117593,117582,117575,117574,117571,117556,117552,117550,117532,117524,117523,117455,117310,117196,117628,164653,162070,162075,162076,158485,90504,90495,90481,90467,90458,90457,90456,90450,90435,90432,90425,90416,90408,90407,90405,90399,90372,90364,90363,90359,90349,90345,90343,90323,90320,90308,90304,90297,90295,90278,90261,90255,90247,90244,90230,90226,90219,90211,90209,90206,90197,90187,90177,90164,90163,90158,90156,90154,90153,90125,90117,90114,90108,90103,90093,90084,90079,90073,90072,90061,90053,90045,90026,90022,90020,90018,90016,90001,89994,89993,89985,89977,89952,89942,89941,89934,89931,89926,89923,89916,89911,89908,89907,89891,89887,89882,89881,89876,89842,89836,89826,89823,89811,89805,89802,89801,89796,89793,89789,89757,89750,89737,89728,89724,89708,89707,89704,89699,89694,89691,89679,89666,89664,89663,89655,89648,89638,89637,89634,89621,89618,89614,89611,89609,89588,89565,89554,89552,89546,89541,89528,89523,89518,89517,89513,89512,89511,89501,89494,89489,89482,89480,89478,89471,89467,89448,89444,89420,89410,89401,89398,89397,89394,89383,89371,89364,89355,89352,90737,90726,90724,90720,90713,90702,90696,90695,90692,90680,90677,90673,90671,90668,90667,90663,90661,90658,90653,90648,90646,90642,90636,90635,90631,90618,90603,90594,90590,90575,90568,90564,90551,90531,90395,90369,90225,90221,90214,90031,89862,89860,89821,89786,89684,89682,89597,90559,90423,129255,129387,129416,129450,129194,129257,129260,129270,129307,129313,129314,129331,129333,148492,148502,148438,148436,148465,148484,165634,165913,154136,154087,154103,154104,154132,131633,131685,131773,131781,131810,131086,131315,131369,131405,131481,131539,131552,131629,131725,131742,131747,131758,131759,131771,131772,131787,131790,131800,149191,149189,149182,149173,149166,149164,149163,149162,149155,149144,149133,149130,149123,149121,149118,149117,149115,149101,149098,149147,149202,30961,29918,30384,30524,29257,29314,29326,29430,29432,29620,29746,29751,29775,29894,29898,30047,30145,30161,30377,30596,30863,30864,30900,30949,31233,31236,31235,31323,31417,31427,31524,31628,31901,32051,32120,32135,32244,32249,32321,29160,29168,29169,29171,29190,29209,29214,29218,29225,29231,29237,29240,29242,29246,29253,29255,29268,29276,29279,29283,29285,29286,29288,29289,29291,29293,29295,29306,29307,29310,29312,29320,29325,29328,29336,29339,29341,29356,29358,29361,29362,29364,29368,29382,29385,29390,29397,29398,29405,29408,29415,29436,29443,29448,29451,29466,29478,29486,29495,29504,29505,29507,29531,29532,29533,29534,29536,29540,29542,29543,29550,29556,29566,29568,29569,29570,29579,29582,29594,29603,29609,29615,29617,29623,29627,29634,29639,29649,29654,29655,29661,29665,29667,29672,29677,29678,29685,29686,29690,29697,29698,29701,29703,29704,29705,29711,29715,29738,29741,29749,29765,29768,29769,29772,29784,29788,29789,29791,29795,29798,29801,29810,29813,29827,29830,29832,29834,29849,29853,29871,29872,29875,29889,29901,29909,29911,29916,29920,29930,29937,29943,29947,29959,29970,29979,29983,29995,30001,30004,30011,30013,30014,30025,30041,30079,30093,30097,30098,30112,30123,30125,30133,30134,30139,30146,30149,30162,30164,30165,30171,30175,30181,30183,30194,30205,30208,30217,30224,30228,73653,73638,73626,73619,73618,73592,73590,73587,73583,73577,73574,73561,73559,73556,73548,73543,73537,73530,73526,73519,73513,73512,73499,73496,73495,73494,73492,73485,73453,73435,73422,73421,73415,73413,73409,73389,73373,73372,73362,73360,73347,73340,73332,73328,73321,73318,73302,73292,73291,73278,73277,73276,73274,73273,73269,73267,73256,73254,73244,73230,73216,73214,73203,73188,73187,73181,73180,73168,73165,73163,73139,73122,73057,73054,73052,73041,73032,73016,73011,73008,73007,73006,73005,72981,72971,72959,72958,72953,72944,72939,72918,72915,72908,72882,72867,72856,72850,72802,72799,72798,72791,72785,72765,72733,72723,72705,72704,72688,72686,72685,72680,72676,72667,72658,72650,72649,72639,72629,72607,72600,72598,72593,72566,72559,72534,72525,72508,72507,72490,72482,72462,72449,72443,72442,72435,72434,72414,72406,72398,72397,72378,72375,72366,72357,72356,72348,72300,72299,72275,72271,72268,72267,72264,72247,72234,72232,72230,72225,72212,72206,72200,72187,72186,72184,72179,72177,72176,72171,72164,72147,72146,72145,72124,72123,72114,72105,72104,72101,72040,73844,73837,73810,73807,73794,73787,73780,73770,73769,73759,73754,73752,73750,73749,73739,73736,73729,73727,73725,73723,73722,73718,73713,73710,73703,73698,73694,73693,73690,73686,73685,73681,73679,73675,73673,73672,73610,73609,73586,73568,73488,73483,73458,73455,73351,73220,73209,73178,73118,73094,72735,72589,72532,72370,72320,72314,72185,72180,72140,73776,72854,73667,165202,137817,137819,137822,137835,137846,137861,137864,137865,137868,137878,137879,137891,137892,137912,137920,137932,137938,137939,137942,137943,137948,137950,137956,137959,137962,137965,137983,137989,137995,138013,138018,138023,138053,138061,138079,138088,138090,138105,138106,138123,138128,137808,164073,164070,164068,164063,119303,119301,119290,119270,119243,119233,119221,119217,119211,119198,119195,119178,119176,119158,119150,119128,119099,119097,119074,119065,119052,119044,119034,119004,119643,119634,119628,119611,119565,119549,119528,119511,119497,119496,119476,119470,119449,119442,119427,119417,119416,119390,119373,119367,119349,119347,119334,119314,119231,119542,119489,30245,30255,30264,30265,30266,30267,30298,30304,30310,30320,30322,30325,30351,30356,30371,30378,30387,30393,30395,30397,30415,30431,30445,30448,30456,30462,30463,30473,30475,30480,30484,30499,30507,30514,30517,30531,30535,30557,30562,30570,30572,30587,30597,30598,30611,30626,30629,30657,30674,30677,30702,30704,30711,30733,30740,30746,30751,30778,30783,30786,30788,30789,30794,30796,30800,30809,30832,30833,30846,30853,30859,30869,30905,30907,30912,30918,30924,30943,30944,30973,30977,30978,30983,30987,30988,30992,30999,31000,31001,31013,31014,31018,31024,31046,31049,31052,31055,31073,31076,31079,31108,31115,31117,31121,31125,31129,31149,31160,31164,31165,31166,31172,31175,31194,31199,31205,31222,31227,31252,31257,31267,31274,31276,31278,31291,31298,31305,31328,31334,31346,31349,31355,31361,31363,31364,31377,31379,31384,31392,31397,31410,31411,31413,31419,31432,31451,31452,31458,31459,31472,31480,31489,31496,31500,31536,31554,31582,31595,31599,31601,31610,31612,31616,31633,31657,31667,31673,31675,31682,31686,31693,31745,31750,31754,31760,31762,31767,31773,31777,31782,31783,31785,31786,31788,31807,31808,31812,31820,31827,31829,31839,31853,31862,31868,31870,31883,31892,31914,31925,31926,31927,31931,31939,31940,31942,31970,31972,31975,31983,31987,32001,32003,32004,32009,32010,32013,32016,32036,32038,32057,32060,32092,32099,32104,32111,32115,32124,32130,32131,32138,32139,32147,32149,32150,32152,32174,32182,32183,32184,32215,32219,32221,32223,32224,32225,32235,32245,32257,32265,32266,32269,32274,32276,32283,32303,32304,32317,32318,32331,29140,29141,29146,29147,165881,153305,153345,153347,153221,153237,153268,153285,153288,153325,153334,125612,125602,125568,125559,125557,125542,125531,125527,125523,125515,125509,125504,125502,125478,125467,125466,125464,125460,125439,125438,125421,125412,125406,125404,125402,125378,125368,125355,125354,125887,125874,125868,125854,125853,125840,125829,125825,125819,125813,125784,125782,125780,125758,125755,125751,125743,125728,125721,125720,125715,125707,125698,125686,125661,125658,125654,125651,125623,125598,125431,125401,125381,125359,125826,139245,139246,139424,139451,139488,139515,139225,139229,139232,139263,139264,139273,139274,139288,139290,139300,139306,139327,139328,139376,139381,139397,139411,139414,139419,139428,139434,139436,139472,139473,139480,139502,139510,139513,139521,139547,139177,154062,154065,153984,154006,154020,154032,154033,154052,163076,163060,163072,164020,164299,164295,164296,161198,161206,161214,161217,161181,114814,114734,114725,114683,114314,114798,114750,163399,163400,163403,160946,160960,160972,111789,111383,111612,111617,111258,111278,111469,111591,111598,111616,111628,111656,111662,111669,111670,111683,111699,111704,111706,111723,111751,111755,111759,111765,111767,111771,111227,111231,111234,111245,111246,111248,111251,111253,111257,111273,111283,111307,111312,111319,111321,111332,111333,111356,111368,111370,111375,111384,111387,111394,111398,111403,111415,111418,111420,111432,111458,111475,111480,111488,111497,111507,111510,111513,111533,111540,111576,111587,166306,137014,137013,136993,136991,136966,136961,136893,137300,136954,136767,165223,7737,7736,7735,7729,7720,7718,7712,7708,7641,7603,7578,7551,7549,7535,7534,7530,7527,7519,7511,7502,7500,7488,7485,7484,7474,7473,7471,7467,7428,7424,7418,7404,7397,7395,7393,7390,7387,7385,7380,7361,7345,7341,7340,7333,7330,7327,7321,7318,7306,7298,7291,7289,7281,7276,7275,7274,7272,7271,7270,7260,7246,7239,7238,7237,7223,7216,7215,7212,7208,7206,7184,7173,7169,7165,7163,7160,7158,7151,7145,7135,7128,7126,167537,7122,7119,7076,7073,7070,7066,7061,7049,7045,7043,7008,7005,7000,6994,6993,6959,6944,6939,6931,6926,6923,6922,6919,6913,6910,6909,6907,6906,6904,6900,6894,6893,6886,6876,6868,6863,6859,6856,6840,6839,6819,6818,6813,6811,6806,6804,6803,6800,6782,6777,6774,6763,6762,6758,6755,6738,6725,6721,6717,6716,6712,6703,6699,6692,6689,6673,6672,6638,6623,6598,6583,6580,6574,6571,6526,6522,6519,6512,6508,6507,6504,6501,6498,6489,6462,6436,6433,6419,6406,6397,6382,6354,6352,6344,6315,6314,6308,6307,6270,6266,6261,6253,6240,6232,6231,6230,6214,6212,6201,6198,6197,6189,6172,6169,6164,6162,6156,6154,6152,6149,6148,6140,6134,6108,6107,6106,6102,6092,6057,6056,6052,11076,11055,11052,11043,11036,11021,11016,11009,11000,10985,10981,10980,10971,10969,10968,10955,10921,10918,10915,10913,10909,10894,10873,10872,10869,10858,10851,10849,10835,10820,10804,10803,10801,10799,10795,10787,10782,10775,10771,10767,10761,10753,10737,10734,10728,10723,10713,10698,10687,10653,10652,10648,10645,10626,10621,10615,10614,10613,10611,10610,10600,10583,10572,10562,10551,10550,10546,10541,10534,10527,10523,10522,10519,10515,10504,10499,10495,10477,10476,10475,10466,10465,10443,10440,10431,10408,10404,10394,10386,10368,10364,10362,10355,10352,10349,10340,10338,10315,10313,10309,10306,10303,10302,10298,10295,10290,10286,10273,10270,10262,10259,10243,10232,10215,10201,10197,10194,10190,10188,10186,10184,10175,10171,10154,168021,10148,10143,10142,10132,10120,10119,10112,10105,10102,10096,10085,10081,10077,10060,10040,10027,10024,10023,10020,10018,10011,10009,9983,9981,9967,9960,9958,9957,9954,9938,9907,9906,9900,9893,9888,9885,9882,9875,9873,9870,9858,9856,9830,9829,9823,9809,9808,9802,9801,9797,9785,9778,9770,9743,9738,9719,9715,9697,9690,9689,9686,9657,9648,9647,9615,9604,9603,9589,9574,9571,9548,9528,9513,9511,9467,9454,9449,9444,9420,9417,9400,9396,9395,9383,9381,9373,9370,9365,9351,9350,9330,9317,9316,9298,9295,9293,9245,9240,9236,9234,9223,9220,9217,9203,9191,9180,9177,9162,9159,9157,9131,9125,9099,9091,9086,9073,9067,9066,9059,9039,9038,9033,9028,9027,9017,9011,8997,8990,8979,8978,8957,8950,8941,8928,8918,8917,8911,8909,8900,8887,8886,8881,8869,8852,8840,8820,8801,8800,8795,8793,8779,8773,8762,8761,8747,8746,8744,8739,8737,8725,8715,8681,8679,8650,8637,8633,8618,8605,8582,8574,8571,8565,8558,8555,8553,8546,8540,8539,8532,8524,8506,8503,8486,8474,8468,8453,8452,8449,8444,8440,8437,8434,8422,8412,8410,8397,8373,8363,8340,8320,8308,8299,8281,8264,8251,8238,8214,8194,8187,8179,8173,8171,8164,8153,8137,8135,8131,8127,8122,8107,8100,8094,8093,8091,8089,8085,8065,8059,8048,8046,8041,8039,8034,8033,8024,8006,7991,7985,7965,7963,7961,7954,7933,7929,7908,7905,7902,7892,7891,7890,7889,7886,7881,7879,7870,7852,7845,7836,7835,7831,7830,7827,7824,7817,7795,7792,7789,7785,7784,7779,7778,7774,7768,7763,7730,7724,7661,7654,7570,7496,7382,7365,7351,7269,7129,7055,7013,6948,6768,6767,6766,6761,6760,6745,6701,6637,6606,6591,6557,6434,6342,6309,6130,6116,6073,11072,10975,10974,10867,10707,10702,10668,10655,10595,10451,10305,10263,10260,10207,9887,9853,9850,9746,9670,9656,9412,9255,9226,8694,8580,8550,8309,8283,8282,8268,8205,8193,8162,8132,8114,8106,8037,8013,7859,7855,7819,11002,10711,10507,8498,8458,7958,7910,9337,165699,165591,165484,115237,115344,115346,115360,115414,114978,114980,115021,115110,115157,115204,115220,115221,115222,115232,115246,115260,115276,115283,115286,115290,115316,115322,115326,115330,115337,115338,115352,115384,115388,115389,115395,115398,115400,115416,115418,115419,115423,115426,115428,115431,115433,115439,115445,114931,114944,114946,114963,114967,114971,114973,114989,114992,114994,115009,115014,115023,115025,115037,115038,115046,115053,115060,115071,115078,115086,115105,115113,115118,115126,115138,115170,115174,115183,115190,115193,115194,158140,158138,158101,158097,158087,158083,158082,158074,158066,158064,158063,158060,158053,158046,158090,165481,145022,144927,144930,144942,144947,144958,144972,144974,144987,145009,145017,145037,162498,156854,162024,162012,159786,159812,159347,159329,164196,163514,163537,163523,163515,163327,163326,163325,163321,152818,152814,152801,152787,152774,152763,152755,152736,152722,152750,142711,142706,142690,142685,142669,142654,142647,142644,142640,142613,142597,142568,142566,142554,142552,142539,142534,142511,142508,142487,142469,142730,160400,160398,160426,160421,160411,160346,160345,160419,144322,144320,144313,144309,144298,144292,144281,144274,144273,144264,144242,144222,144221,144196,144194,144190,144173,144162,144161,144145,144139,144135,144122,144093,144301,144214,164642,164691,164692,132995,132984,132974,132941,132940,132923,133338,133329,133325,133286,133267,133266,133249,133245,133227,133220,133212,133201,133195,133132,133114,133109,133093,133091,133080,133067,133054,133037,133033,133015,132994,133019,132960,91943,92546,91697,92013,92424,92466,92513,90788,90820,90884,91057,91085,91167,91291,91365,91366,91426,91465,91494,91672,91798,92172,92218,92274,92328,92332,92346,92347,92353,92355,92426,92434,92438,92445,92454,92458,92467,92478,92481,92491,92492,92503,92512,92515,92525,92547,92550,92554,92564,92590,92599,92601,92603,90764,90766,90767,90782,90794,90807,90825,90828,90835,90838,90844,90854,90860,90869,90876,90882,90883,90885,90900,90902,90906,90912,90915,90917,90927,90932,90935,90938,90941,90961,90973,90995,91005,91010,91016,91031,91033,91034,91037,91040,91061,91063,91068,91076,91083,91101,91107,91114,91125,91127,91131,91132,91134,91135,91170,91198,91202,91215,91216,91221,91226,91229,91240,91246,91263,91267,91274,91284,91298,91300,91304,91306,91321,91339,91345,91356,91362,91372,91386,91394,91396,91397,91403,91406,91416,91417,91421,91441,91446,91449,91454,91462,91469,91488,91493,91499,91506,91529,91530,91545,91554,91564,91601,91605,91609,91631,91638,91639,91641,91646,91657,91658,91668,91669,91686,91687,91690,91691,91692,91693,91700,91701,91709,91728,91759,91761,91762,91768,91773,91775,91782,91790,91791,91795,91808,91813,91817,91828,91831,91836,91840,91841,91844,91850,91853,91868,91873,91882,91886,91892,91900,91910,91911,91930,91947,91953,91987,92005,92031,92039,92041,92043,92045,92049,92054,92062,92073,92075,92080,92085,92087,92092,92099,92102,92108,92113,92114,92123,92132,92138,92159,92174,92176,92188,92212,92219,92229,92231,92237,92247,92284,92285,92295,92301,92306,92325,92330,92336,92339,92342,92350,92371,92376,92382,92383,92393,92394,92395,92403,110901,110893,110876,110850,110844,110840,110838,110834,110833,110821,110818,110807,110789,110787,110780,110756,110753,110751,110727,110720,110707,110703,110683,110680,110669,110667,110657,110653,110649,110648,110627,110618,110607,110575,110570,110555,110550,110549,110547,110526,110511,110507,110502,110500,110495,110482,110469,110447,110437,110433,110432,110420,110406,110391,110386,110378,110377,110374,110370,110363,110355,110350,110349,110347,110330,110326,110325,111202,111201,111200,111180,111179,111172,111168,111164,111150,111121,111117,111097,111071,111057,111055,111049,111032,111020,111016,111012,111009,111006,111002,111001,110998,110993,110991,110983,110981,110977,110967,110960,110957,110944,110935,110932,110921,110917,110804,110613,110582,110413,111139,110514,164472,164477,34808,34798,34793,34784,34779,34763,34760,34758,34742,34739,34738,34731,34726,34724,34707,34700,34693,34690,34687,34665,34632,34619,34610,34598,34597,34592,34584,34574,34568,34565,34519,34503,34496,34495,34481,34473,34470,34469,34465,34455,34439,34433,34431,34424,34423,34419,34418,34413,34407,34406,34403,34396,34393,34382,34380,34379,34373,34369,34359,34350,34349,34341,34331,34330,34327,34321,34304,34298,34288,34276,34270,34238,34236,34233,34232,34228,34220,34215,34214,34213,34212,34203,34202,34188,34187,34185,34177,34141,34137,34136,34105,34092,34091,34082,34079,34068,34060,34059,34051,34050,34048,34046,34045,34027,34022,33996,33972,33970,33950,33947,33940,33939,33933,33931,33923,33922,33915,33908,33906,33880,33877,33865,33860,33847,33835,33824,33799,33786,33782,33769,33765,33764,33757,33734,33723,33722,33715,33713,33707,33693,33662,33660,33657,33653,33648,33643,33633,33617,33602,33592,33590,33584,33579,33576,33572,33570,33559,33557,33556,33551,33538,33532,33524,33522,33521,33506,33502,33501,33499,33497,33487,33484,33480,33459,33444,33433,33432,33429,33414,33403,33402,33400,33361,33352,33351,33346,33344,33340,33339,33313,33249,33240,33237,33229,33226,33215,33214,33213,33185,33183,33176,33156,33150,33133,33124,33120,33110,33109,33108,33096,33080,33078,33070,33063,33058,33048,33038,33027,33026,33023,33021,33014,33008,33007,32999,32989,32976,32972,32971,32968,32956,32939,32935,32925,32918,32917,32913,32906,32902,32896,32894,32881,32871,32862,32861,32860,32850,32844,32831,32819,32818,32792,32790,32782,32780,32779,32778,32777,32775,32757,32755,32751,32747,32737,32734,32720,32717,32702,32694,32674,32667,32665,32663,32646,32644,32636,32635,32634,32630,32625,32624,32611,32610,32607,32602,32600,32595,32587,32578,32574,32570,32560,32555,32537,32534,32530,32525,32524,32522,32513,32508,32497,32492,32471,32466,32454,32447,32443,32441,32434,32427,32420,32402,32394,32388,32383,32382,32367,32365,32353,35347,35340,35336,35335,35332,35330,35327,35286,35242,35223,35198,35148,35036,34904,34818,34814,34733,34662,34637,34538,34525,34522,34475,34302,34174,33984,33954,33928,33899,33898,33843,33798,33708,33516,33486,33443,33418,33412,33373,33248,33184,33135,33125,33077,32984,32786,32727,32666,32569,32546,32540,34928,34617,34452,33753,33652,32621,165372,165235,164198,140465,140626,140600,140596,140591,140575,140573,140563,140559,140558,140557,140555,140553,140542,140518,140512,140503,140487,140486,140483,140479,140474,140622,140601,140578,166272,147719,147718,147712,147708,147705,147685,147684,147677,147671,147667,147662,147659,147657,147655,147652,147649,147644,147636,147631,147625,147621,147598,147595,147724,156078,156062,156041,158894,158914,166087,121395,121381,121380,121370,121369,121364,121358,121351,121349,121348,121332,121320,121316,121312,121305,121285,121282,121279,121274,121263,121529,121508,121507,121505,121502,121485,121458,121446,121432,121404,121525,121513,121497,121498,121425,121398,78840,179753,80171,80192,78684,78729,78923,78941,79186,79313,79596,79692,79753,79819,79837,80009,80046,80059,80061,80065,80070,80077,80078,80079,80086,80089,80101,80104,80105,80132,80133,80134,80137,80139,80152,80179,80186,80190,80191,80193,78697,78702,78712,78732,78735,78739,78748,78751,78754,78759,78761,78763,78764,78766,78767,78774,78780,78781,78791,78796,78797,78799,78804,78807,78811,78816,78818,78819,78822,78829,78830,78844,78850,78855,78869,78871,78878,78879,78890,78893,78900,78915,78916,78917,78918,78919,78921,78925,78926,78927,78933,78955,78958,78967,78974,78975,78979,78982,78986,78990,78993,78994,78995,79009,79014,79023,79029,79031,79033,79039,79042,79067,79072,79074,79085,79089,79090,79096,79098,79101,79111,79120,79127,79128,79137,79147,79157,79159,79162,79165,79168,79176,79179,79189,79195,79203,79210,79224,79227,79246,79248,79261,79265,79267,79269,79272,79280,79286,79292,79297,79306,79308,79309,79311,79315,79322,79341,79346,79348,79353,79363,79364,79366,79375,79400,79410,79415,79417,79420,79425,79436,79438,79443,79458,79460,79465,79477,79483,79489,79493,79508,79509,79513,79515,79517,79523,79539,79541,79543,79544,79545,79546,79548,79559,79563,79564,79576,79604,79612,79644,79650,79652,79659,79673,79683,79684,79686,79700,79712,79719,79722,79724,79744,79758,79769,79774,79780,79783,79801,79803,79807,79811,79816,79821,79831,79839,79841,79867,79870,79880,79884,79889,79897,79899,79912,79913,79921,79924,79926,79929,79932,79933,79940,79951,79952,79964,79969,79979,79994,80012,80018,80027,80036,80042,80043,96319,96316,96311,96286,96281,96274,96266,96264,96254,96252,96248,96246,96239,96236,96235,96232,96229,96225,96222,96203,96202,96185,96174,96159,96146,96145,96143,96141,96128,96098,96090,96086,96077,96060,96056,96047,96046,96029,96026,96022,96021,96005,95997,95991,95990,95979,95977,95976,95969,95963,95948,95946,95939,95922,95916,95914,95912,95900,95896,95894,95890,95879,95862,95851,95845,95827,95826,95823,95807,95799,95779,95777,95773,95758,95741,95730,95728,95726,95725,95724,95721,95710,95701,95698,95692,95686,95681,95676,95666,95661,95639,95637,95628,95626,95622,95619,95603,95592,95583,95563,95553,95543,95542,95541,95534,95523,95521,95505,95500,95490,95482,95480,95476,95472,95452,95446,95445,95441,95435,95418,95409,95395,95388,95385,95355,95354,96713,96711,96699,96692,96688,96676,96670,96645,96644,96643,96642,96640,96638,96625,96599,96580,96576,96574,96568,96551,96545,96536,96523,96515,96508,96501,96449,96448,96434,96433,96400,96371,96364,96333,96119,96117,96063,96003,95847,95770,95752,95495,95479,95456,95438,96550,65161,64305,64446,65195,65261,65264,66123,66233,64122,64153,64375,64379,64514,64689,64692,64726,64814,64837,64844,64901,65006,65011,65154,65248,65325,65440,65615,65761,65798,65807,65809,65985,66052,66068,66069,66144,66154,66165,66166,66193,66216,66220,66234,66246,66248,66250,66256,66262,66266,66271,66282,64110,64136,64139,64159,64163,64164,64165,64166,64177,64185,64193,64211,64225,64227,64231,64232,64244,64256,64264,64275,64278,64284,64288,64291,64293,64307,64310,64318,64327,64329,64352,64362,64372,64382,64383,64386,64392,64407,64411,64412,64419,64434,64436,64438,64441,64444,64447,64448,64468,64470,64471,64472,64473,64478,64479,64483,64486,64512,64522,64539,64556,64563,64564,64576,64577,64583,64618,64628,64631,64632,64634,64640,64641,64644,64649,64657,64660,64679,64682,64695,64710,64718,64729,64740,64753,64755,64772,64776,64790,64808,64818,64834,64841,64845,64852,64867,64874,64887,64896,64902,64911,64920,64921,64939,64943,64953,64964,64965,64973,64982,64989,64992,65001,65018,65024,65035,65038,65040,65052,65059,65061,65069,65078,65080,65106,65111,65120,65121,65122,65126,65142,65146,65157,65160,65164,65166,65175,65185,65186,65190,65197,65198,65200,65209,65210,65223,65228,65231,65268,65269,65271,65281,65284,65293,65294,65296,65309,65314,65324,65326,65328,65345,65346,65358,65364,65377,65389,65393,65404,65410,65417,65426,65427,65429,65430,65431,65439,65445,65447,65450,65461,65470,65472,65473,65477,65481,65491,65503,65504,65512,65514,65519,65522,65537,65540,65543,65544,65545,65568,65572,65574,65575,65578,65584,65587,65590,65599,65622,65624,65639,65642,65651,65653,65659,65671,65672,65678,65680,65710,65709,65733,65736,65746,65752,65769,65777,65816,65833,65852,65858,65876,65886,65891,65915,65923,65929,65935,65936,65943,65951,65954,65955,65956,65963,65965,65967,65989,65990,65995,65996,66000,66003,66004,66009,66017,66034,66035,66036,66037,66049,66056,66071,66077,66078,66083,66086,66135,163866,136379,136527,136543,136639,136652,136671,136098,136143,136207,136232,136233,136269,136272,136337,136389,136399,136423,136426,136439,136441,136446,136456,136461,136475,136482,136494,136529,136542,136568,136572,136576,136585,136588,136594,136596,136597,136624,136628,136630,136646,136649,136658,136093,136103,136110,136194,136214,136220,136228,136242,136279,136300,164426,164427,164428,165000,164928,164930,164933,163614,163433,163441,163406,163408,165834,115789,115810,115820,115834,115848,115857,115861,115869,115880,115897,115908,115913,115914,115916,115931,115937,115940,115956,115959,115961,115973,115989,115991,116005,116006,116041,115455,115463,115474,115480,115486,115503,115528,115531,115533,115539,115544,115549,115551,115552,115568,115571,115606,115609,115610,115626,115652,115653,115662,115670,115671,115674,115675,115678,115684,115693,115695,115697,115698,115700,115701,115703,115712,115713,115714,115716,115717,115722,115730,115733,115738,115740,115743,115744,164334,166143,160309,160322,160321,160311,160303,160299,160314,164170,162743,161140,161148,164710,164713,161504,161494,161499,160191,160189,160182,164880,166018,161941,164708,162845,162839,89103,89087,89082,89077,89073,89070,89063,89054,89041,89034,89020,89006,89002,88993,88988,88986,88985,88983,88980,88979,88957,88952,88950,88945,88943,88931,88927,88925,88918,88903,88902,88895,88894,88893,88892,88885,88881,88880,88871,88864,88862,88861,88859,88849,88846,88835,88823,88809,88801,88792,88786,88779,88775,88769,88766,88760,88750,88744,88734,88724,88720,88707,88699,88695,88681,88676,88671,88659,88658,88647,88645,88641,88637,88631,88625,88615,88606,88601,88575,88572,88568,88565,88559,88552,88548,88546,88539,88533,88519,88515,88496,88491,88488,88480,88472,88469,88452,88451,88449,88440,88415,88407,88405,88398,88397,88387,88380,88374,88373,88369,88367,88361,88360,88358,88357,88356,88354,88348,88344,88343,88337,88335,88334,88331,88312,88309,88306,88299,88295,88290,88271,88270,88261,88254,88247,88234,88230,88226,88224,88210,88209,88208,88206,88202,88201,88182,88181,89347,89336,89328,89325,89316,89313,89290,89288,89285,89283,89279,89272,89267,89260,89257,89252,89249,89246,89237,89225,89216,89212,89201,89200,89190,89189,89188,89187,89173,89162,89152,89145,89137,89067,89035,89003,88966,88961,88848,88713,88665,88640,88503,88450,88448,88444,88410,88400,88342,88314,88304,89348,89175,88281,88173,166267,104890,105372,105433,105543,104743,104766,104789,104856,105067,105086,105112,105255,105258,105262,105328,105330,105333,105336,105342,105345,105354,105355,105356,105364,105368,105370,105388,105389,105395,105399,105411,105413,105417,105418,105423,105430,105432,105442,105462,105467,105470,105473,105475,105482,105483,105484,105486,105488,105496,105500,105501,105502,105505,105506,105517,105568,105572,105580,105595,104686,104693,104695,104702,104705,104710,104711,104718,104723,104729,104737,104745,104749,104762,104778,104785,104788,104790,104799,104806,104807,104826,104836,104847,104849,104850,104853,104868,104892,104909,104913,104918,104920,104955,104957,104966,104968,105000,105003,105010,105012,105013,105019,105028,105030,105047,105080,105087,105092,105093,105123,105134,105142,105155,105157,105158,105160,105183,105186,105188,105201,105204,105212,105217,105223,105229,105230,105251,105256,105276,105282,105287,105288,105289,105299,105301,105313,77201,77198,77185,77178,77177,77176,77158,77155,77145,77125,77119,77114,77110,77107,77097,77083,77080,77061,77053,77048,77033,77031,77030,77027,77023,77005,77002,76997,76996,76960,76957,76954,76946,76937,76931,76927,76921,76915,76913,76901,76897,76884,76883,76864,76861,76858,76828,76810,76806,76805,76804,76793,76784,76758,76757,76738,76736,76732,76718,76714,76704,76694,76690,76681,76671,76670,76668,76667,76666,76661,76658,76657,76656,76649,76645,76642,76639,76637,76627,76625,76620,76614,76612,76604,76596,76568,76566,76565,76564,76563,76543,76535,76519,76518,76507,76503,76501,76495,76494,76489,76479,76473,76466,76458,76444,76438,76421,76415,76394,76389,76385,76384,76378,76373,76362,76354,76330,76327,76326,76323,76322,76314,76312,76309,76302,76285,76280,76277,76272,76269,76268,76265,76255,76240,76239,76227,76224,76202,76173,76147,76146,76129,76121,76083,76071,76055,76052,76030,76025,76013,76011,76008,75989,75988,75985,75982,75977,75971,75970,75968,75965,75902,75895,75887,75859,75855,75847,75839,75833,75825,75821,75805,75802,75795,75787,75784,75770,75769,75768,75763,75751,75747,75741,75740,75737,75724,75696,75689,75675,75653,75654,75638,75636,75605,75601,75596,77446,77445,77444,77427,77398,77376,77375,77349,77340,77336,77326,77321,77309,77307,77304,77293,77282,77279,77268,77261,77258,77256,77242,77228,77220,77210,77188,77165,77151,77084,77042,77024,76788,76740,76634,76502,76443,76416,76346,76250,76205,76168,76077,76042,75999,75868,77244,77237,76743,94180,94815,95266,95277,94108,94173,94223,94231,94516,94580,94649,94689,94693,94698,94966,95032,95075,95086,95087,95113,95118,95120,95121,95169,95171,95177,95183,95186,95191,95211,95213,95223,95225,95226,95233,95235,95245,95253,95260,95263,95273,95290,95297,95299,95301,95305,95313,95320,95329,95333,95344,95346,93937,93944,93956,93960,93964,93982,93999,94026,94027,94034,94045,94047,94048,94053,94074,94075,94085,94087,94090,94095,94096,94102,94152,94154,94177,94178,94182,94196,94206,94215,94222,94225,94234,94246,94262,94263,94264,94266,94271,94273,94279,94288,94297,94300,94319,94331,94345,94358,94372,94373,94378,94379,94380,94390,94394,94395,94396,94400,94402,94410,94426,94431,94443,94446,94458,94464,94465,94468,94471,94484,94485,94492,94496,94511,94515,94522,94527,94530,94538,94540,94546,94549,94561,94565,94569,94583,94584,94589,94594,94597,94598,94602,94609,94612,94625,94636,94635,94643,94644,94655,94668,94673,94714,94725,94727,94734,94738,94746,94757,94758,94765,94770,94772,94780,94782,94786,94790,94791,94793,94798,94801,94803,94818,94859,94868,94875,94885,94888,94913,94928,94943,94950,94956,94958,94961,94965,94974,94976,94998,95017,95021,95023,95026,95031,95036,95038,95054,95059,95070,95082,151162,151158,151150,151120,151116,151109,151107,151059,151056,151054,151050,151188,144404,144382,144380,144371,144369,144366,144363,144357,144698,144689,144668,144666,144661,144655,144620,144603,144583,144574,144560,144547,144539,144521,144510,144487,144485,144459,144446,144441,144440,144439,161453,161449,161448,161443,161476,161471,161469,161466,102392,102390,102381,102326,102295,102285,102278,102237,102202,102187,102164,102145,102143,102134,102127,102125,102124,102120,102117,102116,102107,102085,102084,102083,102080,102079,102078,102074,102052,102022,102021,151529,151520,151516,151513,151510,151509,151488,151482,151479,151465,151449,151448,151420,151546,151469,151440,151545,155555,155524,155507,155501,155488,155487,155479,155466,155457,155455,155454,155446,155441,155437,155428,155380,155378,155373,155365,155498,155463,165705,162558,162574,156497,156487,156476,156465,156446,156483,156443,133463,133459,133348,133785,149651,149646,149645,149640,149591,149578,149553,149489,149460,149665,163971,143530,143685,143705,143509,143520,143524,143541,143542,143543,143546,143553,143563,143578,143589,143596,143617,143627,143664,143677,155920,155980,156026,155844,155850,155854,155856,155858,155859,155878,155882,155895,155909,155919,155924,155939,155944,155945,155960,156005,156016,159569,159578,159597,159603,160756,160742,160738,160731,160799,160778,130753,130747,130744,130741,130739,130725,130724,130719,130716,130710,130700,130695,130694,130683,130666,130656,130649,130648,131029,131005,130994,130990,130981,130974,130973,130966,130963,130951,130942,130938,130935,130934,130927,130925,130903,130898,130896,130882,130859,130856,130844,130836,130824,130823,130813,130802,130780,130777,130776,130769,150243,150240,165510,42183,42659,44258,44288,44305,44314,44339,41518,41573,41641,41643,41662,41680,41712,41726,41749,41793,41815,41866,41978,41994,42014,42274,42532,42645,42700,42705,42811,42890,43178,43288,43383,43407,43524,43525,43588,43627,43632,43756,43761,43847,43856,43904,43990,44004,44104,44249,44259,44264,44273,44275,44277,44300,44306,44322,44323,44331,44330,44334,41520,41525,41528,41531,41537,41543,41555,41568,41569,41571,41575,41592,41594,41614,41626,41683,41689,41692,41697,41706,41736,41739,41769,41771,41781,41782,41787,41791,41806,41810,41813,41836,41842,41854,41872,41885,41890,41910,41927,41935,41973,41984,41990,41997,42011,42017,42018,42019,42032,42043,42049,42051,42055,42064,42068,42069,42071,42080,42084,42097,42099,42102,42108,42114,42117,42119,42134,42137,42139,42145,42149,42160,42162,42175,42179,42184,42187,42190,42193,42196,42197,42209,42212,42216,42225,42226,42271,42272,42275,42276,42298,42300,42330,42331,42332,42333,42336,42346,42349,42363,42365,42367,42385,42398,42400,42410,42422,42423,42437,42439,42443,42451,42455,42465,42487,42499,42503,42513,42541,42554,42561,42562,42563,42564,42569,42574,42585,42586,42587,42594,42595,42603,42604,42620,42621,42626,42639,42672,42685,42689,42695,42696,42707,42719,42720,42732,42735,42754,42759,42762,42778,42779,42780,42785,42793,42801,42802,42803,42810,42835,42837,42842,42846,42848,42851,42858,42869,42873,42880,42881,42891,42910,42912,42920,42921,42922,42923,42940,42945,42951,42957,42962,42966,42968,42969,42982,42988,42990,42991,43017,43019,43029,43031,43032,43038,43045,43059,43068,43072,43079,43083,43085,43086,43099,43107,43125,43148,43152,43154,43155,43164,43171,43173,43177,43187,43199,43204,43223,43225,43241,43258,43264,43265,43292,43293,43318,43323,43332,43336,43363,43369,43377,43379,43412,43436,43441,43456,43457,43459,43462,43480,43495,43501,43508,43515,43518,43521,43529,43537,43538,43561,43565,43569,43573,43578,43589,43614,43621,43623,43625,43640,43668,43684,43690,43691,43692,43699,43700,43714,43716,43725,43733,43743,43751,43755,43757,43788,43794,43807,43813,43819,43831,43841,43845,43853,43910,43928,43945,43960,43968,43971,43978,43996,43999,44009,44019,44030,44039,44042,44055,44093,44097,44110,44124,44129,44131,44136,44138,44142,44145,44158,44162,44185,44195,44203,44209,44210,44215,44218,44225,71856,71853,71830,71827,71826,71825,71814,71806,71798,71781,71779,71773,71772,71763,71762,71761,71754,71732,71724,71723,71721,71691,71690,71685,71671,71667,71662,71660,71648,71613,71612,71602,71600,71596,71555,71547,71517,71485,71479,71476,71475,71455,71451,71447,71446,71443,71431,71427,71414,71408,71404,71395,71393,71382,71360,71355,71343,71341,71332,71313,71308,71301,71299,71293,71280,71253,71244,71232,71223,71217,71216,71204,71201,71199,71197,71191,71190,71185,71182,71180,71177,71172,71160,71152,71139,71110,71080,71074,71055,71030,70996,70976,70968,70962,70945,70944,70942,70936,70929,70923,70912,70896,70868,70861,70835,70833,70817,70805,70804,70796,70792,70783,70777,70754,70742,70740,70739,70733,70731,70697,70695,70681,70675,70650,70629,70625,70624,70619,70613,70611,70599,70586,70582,70580,70579,70572,70557,70548,70521,70520,70511,70491,70470,70467,70464,70456,70452,70422,70414,70411,70398,70396,70393,70392,70389,70381,70375,70365,70364,70347,70343,70328,70327,70314,70297,70287,70263,70258,70253,70243,70237,70234,70232,70230,70228,72013,72012,72010,72005,72004,72000,71990,71987,71986,71985,71978,71969,71966,71962,71955,71941,71940,71939,71937,71935,71933,71929,71924,71923,71917,71909,71906,71903,71901,71900,71898,71889,71880,71874,71872,71870,71726,71709,71642,71608,71592,71493,71484,71471,71437,71154,71127,71111,71101,71037,71016,70930,70877,70849,70755,70737,70724,70717,70687,70588,70499,70480,70391,70290,70277,71980,71958,71953,71741,165549,165548,108548,108543,108537,108532,108524,108511,108507,108505,108501,108498,108491,108485,108474,108470,108468,108465,108458,108454,108451,108450,108441,108440,108437,108429,108426,108421,108411,108407,108405,108393,108390,108372,108368,108365,108358,108357,108354,108349,108344,108342,108313,108310,108309,108308,108307,108306,108298,108296,108282,108281,108270,108250,108249,108245,108241,108240,108229,108227,108222,108217,108215,108205,108199,108192,108182,108180,108178,108177,108163,108155,108146,108130,108128,108118,108116,108115,108107,108106,108098,108095,108081,108080,108684,108681,108663,108661,108658,108656,108653,108651,108647,108644,108642,108635,108633,108630,108627,108619,108613,108602,108599,108590,108568,108565,108559,108555,108528,108473,108435,108100,108657,108650,108579,108242,165489,163798,157655,157649,157648,157632,157625,157621,157659,165904,157411,157383,157381,165759,145839,145853,145854,145862,145864,145884,145895,145904,145920,145934,145473,145504,145512,145525,145537,145547,145550,145566,153685,153539,153551,153552,153570,153585,153599,153604,153612,153616,153653,153659,153667,153669,153670,153697,153711,153720,153725,153727,153746,153753,153754,153759,153770,153792,153798,153801,153806,153808,153811,145463,145455,145454,153062,152842,152846,152849,152862,152864,152871,152873,152878,152890,152894,152895,152901,152902,152913,152915,152918,152921,152923,152937,152940,152975,152982,152989,152992,153004,153010,153012,153016,153018,153025,153027,153030,153032,153033,153034,153038,153045,153046,153051,153058,153059,153060,164633,4291,5091,5322,5563,5748,881,1571,2402,2526,3212,3220,3367,3431,3594,3649,3694,3770,3773,3990,4030,4044,4266,4701,4712,4729,4807,4919,5060,5071,5118,5311,5351,5357,5495,5500,5503,5537,5700,5721,5751,5786,5787,5801,5846,12,181,320,349,506,600,675,733,759,807,1174,1268,1382,1415,1547,1551,1638,1738,1749,1798,1810,1937,2188,2268,2289,2389,2392,2533,2621,2689,2697,2701,2877,2985,3056,3140,3165,3180,3206,3209,3223,3233,3237,3240,3243,3244,3248,3259,3264,3265,3273,3274,3282,3297,3313,3315,3316,3334,3351,3352,3354,3356,3359,3361,3365,3383,3389,3414,3418,3429,3430,3433,3434,3441,3446,3487,3489,3513,3514,3525,3538,3559,3584,3585,3596,3605,3622,3672,3678,3680,3743,3747,3753,3761,3767,3768,3775,3778,3817,3820,3821,3827,3842,3846,3850,3851,3856,3860,3870,3877,3878,3880,3887,3899,3906,3911,3931,3941,3942,3953,3966,3978,3981,3986,4001,4005,4023,4032,4033,4046,4069,4090,4097,4107,4110,4127,4128,4129,4152,4159,4160,4181,4187,59708,59674,59649,59644,59642,59632,59623,59621,59620,59610,59608,59604,59593,59576,59573,59562,59543,59542,59536,59531,59525,59524,59523,59517,59515,59511,59510,59497,59495,59481,59480,59475,59472,59463,59436,59433,59431,59426,59404,59380,59378,59369,59353,59351,59348,59337,59319,59310,59282,59280,59270,59263,59258,59240,59232,59202,59201,59172,59171,59165,59156,59148,59146,59124,59122,59110,59105,59093,59092,59091,59089,59083,59067,59055,59039,59037,59033,59031,59030,59023,59015,59010,58982,58981,58977,58971,58954,58945,58933,58920,58914,58906,58889,58878,58861,58859,58853,58851,58844,58836,58826,58820,58816,58815,58783,58761,58739,58716,58709,58708,58701,58700,58699,58696,58694,58685,58684,58665,58646,58632,58616,58610,58606,58605,58598,58579,58569,58557,58550,58542,58541,58537,58533,58531,58524,58494,58486,58473,58462,58453,58431,58430,58422,58421,58417,58416,58414,58412,58410,58407,58398,58381,58366,58344,58338,58328,58323,58320,58315,58311,58308,58275,58274,58263,58254,58236,58230,58224,58223,58207,58206,58196,58195,58186,58179,58153,58149,58146,58144,58125,58122,58119,58111,58093,58076,58073,58070,58044,58043,58031,58030,58025,58024,58018,58012,58010,57985,57984,57980,57977,57975,57967,57966,57964,57959,57952,57947,57944,57941,57915,57898,57893,57887,57880,57879,57851,57846,57841,57839,57814,57810,57807,57793,57791,57790,57787,57780,57778,57777,57775,57770,57764,57759,57758,57749,57748,57739,57738,57716,57713,57707,57698,57685,57684,57661,57657,57648,57632,57630,57606,57601,57600,57599,57585,57583,57581,57578,57577,57573,57570,57555,57552,57514,57499,57495,57477,57468,57462,57460,57455,57448,57434,57433,57424,57419,57409,57400,57374,57365,57363,57354,57344,57337,57327,57321,57314,57311,57302,57289,57284,57280,57279,57277,57199,57193,57179,57163,57158,57157,57148,57141,57137,57129,57119,57116,57114,57113,57111,57100,57096,57095,57091,59794,59790,59777,59754,59733,59730,59717,59711,59597,59584,59216,176092,58912,58682,58673,58669,58647,58552,58539,58479,58437,58394,58392,58377,58298,58237,58172,57736,57703,57571,57558,57527,57497,165953,35319,35299,35279,35267,35258,35235,35233,35203,35202,35201,35199,35197,35193,35188,35183,35173,35172,35157,35156,35133,35125,35123,35116,35109,35098,35097,35089,35065,35057,35053,35034,35027,34996,34985,34982,34981,34972,34967,34966,34949,34947,34943,34937,34922,34911,34906,34903,34898,34896,34882,34877,34866,34865,34859,34835,34833,34826,34820,34819,121766,121745,121740,121737,121736,121735,121734,121731,121725,121721,121713,121689,121681,121679,121677,121669,121667,121664,121657,121654,121651,121647,121642,121641,121635,121622,121614,121613,121607,121590,121580,121563,121556,122022,122021,121997,121991,121990,121981,121980,121979,121974,121973,121965,121961,121956,121941,121940,121938,121934,121919,121918,121911,121907,121904,121903,121895,121890,121886,121875,121865,121848,121844,121825,121820,121812,121810,121808,121806,121803,121798,121793,121791,121790,121781,121696,121620,122015,122008,121926,121816,121709,121972,165662,164108,162394,162396,162398,162401,101847,101842,101837,101831,101830,101825,101820,101816,101810,101805,101802,101800,101794,101792,101783,101782,101779,101766,101758,101752,101744,101741,101739,101731,101729,101699,101687,101681,101678,101660,101652,101650,101648,101645,101640,101639,101635,101631,101621,101617,102554,102538,102533,102528,102520,102516,102514,102509,102500,102497,102493,102492,102476,102473,102459,102456,102455,102453,102441,102438,102435,102431,102414,102410,102405,102400,102259,102257,102061,101937,101688,102547,102518,164661,164663,164681,162409,162422,158566,158573,164147,161799,161784,161774,161767,161829,161821,161820,161818,161808,161764,161816,162280,162250,162252,162262,162264,162152,162151,162140,162139,162177,130027,130168,130226,130301,130314,130409,130433,130451,130476,130489,130494,130501,130506,130512,130526,130561,130581,161045,161058,161535,161536,161545,161551,161553,161527,161529,161530,148315,148244,148248,148276,148292,148303,148359,148362,148154,148162,148164,148165,148167,148168,148176,148177,148183,148187,148193,148202,148211,148216,148221,148225,148231,148259,148266,148267,148279,148310,148317,148327,148330,148349,148352,148353,158857,158853,158848,158832,158827,158810,158796,158790,158785,158889,158882,158861,158850,158844,133442,133413,133411,133410,133388,133369,133364,133356,133346,133802,133801,133790,189046,133782,133767,133760,133758,133754,133751,133745,133704,133689,133686,189025,133678,133667,133662,133657,133651,133650,133648,133629,133612,133607,133558,133557,133555,133553,133550,133544,133538,133530,133520,133513,133495,133486,133479,133471,164960,164962,163670,163683,163686,107910,107887,107878,107877,107875,107865,107856,107855,107846,107842,107834,107831,107826,107824,107804,107787,107781,107772,107767,107761,107759,107753,107742,107740,107737,107729,107720,107718,107713,107703,107699,107688,107687,107685,107675,107660,107658,107656,107647,107643,107640,107635,107631,107625,107623,107598,107592,107589,107582,107567,107557,107547,107544,107510,107505,107504,107496,107494,107488,107487,107479,107478,107475,107453,107446,107440,107439,107436,107417,107411,107404,107399,107392,107390,107388,184451,107386,107363,107361,107359,107358,107352,107342,107336,107333,107324,107322,107319,107316,107310,107306,107295,107292,107288,107272,107242,107240,107229,107221,107220,107211,107203,107202,107196,107189,107164,107142,107131,107116,107114,107097,107062,107042,107040,107039,107035,107028,107021,107012,107001,107000,106998,106989,106986,106964,106963,106957,106941,106939,106938,106934,106929,106922,106920,106918,106917,106902,106896,106894,106871,106848,106845,106844,106825,106813,106808,106798,106790,106783,106781,106774,106765,106759,106752,106751,106748,106744,106739,106727,106690,106669,106656,106652,106641,106624,106622,106581,106572,106571,106562,106560,106557,106554,106536,106518,106504,106461,106458,106456,106451,106435,106409,106406,106402,106365,106346,106345,106332,106331,106326,106309,106304,106303,106302,106301,106297,106290,106278,106277,106271,106239,106226,106221,106206,106204,106191,106190,106179,106172,106171,106163,106162,106157,106156,106143,106128,106124,106123,106121,106112,106107,106105,106104,106097,106083,106074,106073,106057,106042,106037,106032,106020,106011,106003,105968,105962,105957,105949,105947,105940,105936,105932,105922,105915,105903,105900,105895,105888,105886,105885,105883,105881,105871,105869,105861,105859,105852,105843,105826,105819,105816,105807,105803,105799,105776,105775,105768,105759,105728,105725,105717,105715,105705,105703,105674,105673,105669,105663,105661,105655,105652,105648,105645,105640,105639,105635,105628,108054,108050,108043,108036,108027,108007,108006,108004,107992,107982,107976,107963,107961,107943,107938,107935,107932,107930,107832,107814,107736,107682,107454,107428,107355,107180,107166,107015,106882,106829,106824,106817,106792,106743,106599,106588,106568,106447,106372,106369,106344,106284,106262,106247,106212,106200,106114,105981,105870,105818,105788,105730,105707,105700,105610,108051,107980,107952,107937,107425,106683,163957,159399,159400,159412,159353,159354,159364,159365,159368,159380,159383,159390,100184,100181,100180,100179,100166,100164,100156,100143,100117,100104,100098,100082,100061,100047,100042,100039,100028,100016,100015,100011,99991,99977,99975,99968,99955,99950,99948,99942,99936,99925,99923,99922,99913,99903,99887,99871,99866,99854,99850,99849,99847,99845,99842,99825,99824,99812,99810,99809,99803,99800,99795,99785,99773,99771,99766,99753,99742,99726,99720,99717,99711,99708,99707,99706,99694,99690,99688,99679,99667,99655,99652,99641,99638,99621,99616,99605,99602,99584,99582,99554,99549,99541,99539,99529,99527,99519,99502,183143,99500,99485,99482,99474,99473,99464,99454,99449,99447,99430,99429,99423,99421,99405,99402,99399,99397,99396,99393,99385,99381,99370,99366,99359,99358,99353,99352,99345,99342,99327,99309,99308,99292,99286,99274,99272,99264,100446,100444,100418,100386,100381,100380,100373,100353,100346,100343,100340,100322,100319,100316,100296,100289,100271,100237,100235,100230,100229,100220,100213,100211,100207,100190,100189,100130,100128,100014,99999,99998,99896,99702,99586,99389,100414,100400,100302,100282,100248,99721,100299,165779,78587,78582,78578,78577,78576,78573,78567,78555,78552,78546,78540,78523,78517,78513,78509,78507,78500,78489,78488,78484,78480,78479,78475,78474,78467,78450,78442,78441,78433,78427,78416,78404,78403,78401,78398,78394,78380,78374,78372,78370,78354,78351,78350,78343,78330,78321,78318,78303,78283,78281,78259,78256,78233,78231,78229,78218,78215,78204,78196,78194,78190,78186,78177,78173,78170,78169,78159,78155,78153,78145,78143,78138,78124,78123,78120,78117,78111,78104,78094,78086,78085,78084,78069,78060,78059,78057,78047,78045,78042,78038,78024,78016,78010,78005,78002,77998,77994,77990,77986,77983,77982,77975,77972,77971,77967,77962,77952,77949,77940,77929,77928,77922,77921,77920,77912,77905,77892,77886,77884,77883,77878,77872,77852,77846,77840,77831,77813,77796,77786,77784,77778,77776,77765,77764,77759,77747,77744,77743,77735,77724,77709,77707,77706,77704,77701,77698,77694,77691,77676,77675,77672,77658,77655,77650,77643,77639,77625,77613,77600,77596,77589,77575,77574,77572,77571,77569,77563,77554,77553,77551,77542,77528,77523,77516,77515,77514,77510,77504,77495,77494,77475,77474,77449,78683,78680,78678,78671,78665,78663,78657,78652,78649,78643,78637,78635,78631,78624,78615,78610,78597,78447,78377,78289,78067,78004,77918,77896,77652,77634,77623,77532,77496,77455,78669,165373,159654,159650,159644,159657,162536,138429,138422,138414,138406,138405,138383,138350,138341,138336,138333,138332,138320,138305,138304,138303,138301,138285,138280,138277,138274,138260,138259,138253,138235,138219,138205,138204,138190,138183,138166,166125,166124,166127,12248,12245,12243,12241,12240,12235,12234,12230,12227,12224,12216,12198,12192,12190,12188,12177,12159,12154,12146,12124,12121,12119,12092,12091,12069,12067,12065,12063,12062,12056,12044,12042,12041,12040,12025,12011,12006,12003,11998,11996,11985,11980,11978,11977,11974,11970,11954,11936,11923,11916,11909,11903,11899,11892,11868,11845,11841,11840,11839,11837,11825,11820,11819,11818,11792,11789,11781,11780,11777,11774,11759,11745,11734,11733,11724,11715,11705,11701,11673,11672,11660,11658,11627,11620,11615,11604,11595,11586,11584,11575,11573,11560,11552,11544,11532,11529,11524,11520,11515,11470,11465,11462,11452,11451,11443,11432,11424,11407,11401,11384,11372,11370,11367,11361,11358,11352,11347,11344,11333,11328,11323,11318,11294,11291,11286,11278,11273,11270,11268,11267,11265,11256,11253,11249,11242,11239,11232,11229,11225,11220,11211,11210,11207,11201,11200,11199,11191,11188,11186,11176,11172,11171,11160,11158,11153,11147,11142,11129,11120,11116,11109,11103,11095,11092,11090,11088,11085,16888,16882,16879,16875,16871,16868,16867,16864,16861,16856,16853,16847,16836,16835,16829,16828,16827,16825,16817,16805,16794,16767,16765,16748,16742,16741,16734,16730,16729,16728,16727,16716,16713,16700,16686,16677,16663,16642,16606,16599,16571,16557,16542,16532,16531,16522,16513,16497,16491,16483,16471,16470,16462,16461,16460,16427,16419,16418,16415,16413,16404,16399,16397,16391,16382,16376,16370,16366,16365,16357,16351,16345,16341,16336,16317,16311,16294,16279,16277,16249,16244,16235,16233,16223,16221,16210,16209,16208,16185,16169,16163,16150,16147,16144,16141,16134,16124,16093,16089,16077,16063,16061,16058,16057,16054,16053,16048,16047,16041,16039,16005,15997,15996,15975,15969,15959,15948,15943,15938,15933,15931,15921,15918,15908,15903,15900,15897,15894,15888,15860,15854,15851,15834,15825,15824,15822,15821,15820,15813,15810,15805,15789,15762,15746,15744,15743,15740,15736,15735,15727,15720,15719,15713,15701,15697,15687,15668,15664,15659,15637,15631,15629,15628,15627,15624,15621,15608,15607,15603,15589,15581,15578,15572,15567,15558,15551,15539,15532,15530,15509,15493,15480,15477,15471,15457,15452,15442,15438,15432,15425,15423,15417,15416,15387,15373,15363,15319,15316,15307,15294,15290,15287,15283,15277,15266,15259,15230,15227,15220,15216,15205,15185,15173,15151,15146,15140,15134,15133,15096,15080,15074,15043,15033,15023,15010,15002,14999,14979,14953,14951,14950,14944,14941,14935,14910,14908,14897,14896,14895,14887,14879,14878,14867,14857,14851,14845,14838,14828,14827,14808,14804,14803,14795,14789,14787,14784,14782,14777,14774,14765,14763,14761,14748,14747,14745,14743,14728,14722,14719,14717,14699,14697,14691,14684,14681,14675,14673,14666,14661,14654,14651,14646,14632,14623,14621,14618,14615,14610,14608,14606,14600,14597,14594,14592,14591,14584,14583,14581,14578,14576,14569,14568,14558,14557,14556,14551,14527,14526,14525,14524,14523,14521,14518,14513,14512,14487,14475,14460,14459,14448,14446,14445,14434,14430,14428,14426,14424,14415,14414,14413,14407,14406,14402,14395,14394,14391,14389,14388,14385,14380,14375,14361,14358,14355,14353,14347,14341,14323,14319,14308,14294,14293,14284,14282,14281,14278,14265,14241,14223,14208,14174,14171,14166,14165,14161,14160,14152,14148,14147,14140,14131,14126,14102,14087,14072,14069,14066,14064,14062,14050,14047,14043,14024,14023,14021,14020,14005,14004,14003,13997,13987,13982,13976,13967,13966,13959,13957,13944,13940,13935,13924,13912,13907,13904,13897,13894,13888,13871,13869,13859,13852,13838,13835,13827,13823,13816,13814,13803,13793,13783,13775,13767,13764,13763,13749,13739,13720,13714,13709,13697,13693,13676,13662,13653,13635,13631,13627,13624,13621,13617,13607,13604,13585,13583,13582,13575,13573,13570,13559,13529,13508,13483,13475,13409,13383,13366,13306,13305,13303,13296,13161,13160,13101,13044,12933,12926,12882,12628,12507,12311,12263,12107,12057,12007,11971,11940,11858,11857,11856,11794,11499,11493,11442,11402,11381,11379,11350,11233,11196,11167,11139,11093,16914,16885,16778,16774,16731,16565,16551,16526,16171,16158,15999,15988,15880,15871,15807,15792,15776,15775,15759,15682,15666,15534,15420,15326,15279,15247,15183,15021,14964,14921,14916,14848,14720,14716,14553,14531,14500,14497,14403,14318,14244,14135,14120,14089,13873,13795,13761,13673,13600,13561,11562,11208,16509,15685,15620,14011,13878,14571,12182,165833,165537,165217,155074,155068,155071,155072,154983,155014,155015,155024,155026,155042,155043,155048,155059,155065,158543,158544,165304,57403,57225,57186,57104,58992,57614,57307,149242,149224,149211,149268,160915,160931,160927,161681,161689,161688,161671,161673,166265,132015,132009,132002,132001,131990,131976,131964,131954,131952,131945,131943,132180,132175,132174,132172,132162,132143,132139,132138,132132,132126,132090,132068,165051,165986,165772,41366,41476,38627,38826,38877,38880,39120,39193,39203,39330,39377,39691,39723,39760,39766,39957,40069,40357,40393,40561,40835,40975,41083,41477,41488,41490,38425,38433,38437,38439,38440,38441,38446,38457,38463,38471,38472,38496,38514,38518,38551,38557,38560,38563,38564,38570,38592,38597,38609,38613,38621,38622,38624,38628,38631,38640,38641,38661,38666,38668,38675,38687,38691,38711,38713,38719,38732,38735,38756,38762,38764,38766,38783,38802,38806,38812,38817,38827,38850,38851,38860,38870,38882,38883,38884,38891,38901,38904,38934,38935,38940,38947,38953,38955,38957,38959,38964,38981,38994,39003,39011,39014,39018,39024,39033,39042,39056,39061,39064,39076,39084,39089,39091,39101,39104,39107,39109,39115,39122,39126,39134,39144,39153,39155,39157,39164,39165,39175,39178,39201,39215,39220,39231,39237,39252,39255,39265,39270,39274,39275,39285,39288,39293,39308,39323,39324,39333,39334,39352,39357,39363,39364,39365,39366,39381,39390,39396,39422,39425,39430,39439,39454,39476,39483,39490,39492,39493,39495,39516,39526,39544,39547,39550,39551,39555,39563,39571,39575,39583,39589,39608,39627,39631,39633,39636,39645,39651,39653,39664,39670,39678,39679,39687,39688,39693,39701,39705,39710,39714,39717,39733,39735,39755,39759,39793,39795,39801,39847,39849,39850,39851,39852,39853,39862,39870,39877,39889,39892,39903,39910,39911,39920,39922,39930,39933,39946,39955,39961,39972,39984,39999,40001,40008,40009,40013,40021,40028,40029,40048,40055,40056,40082,40118,40133,40137,40147,40151,40152,40157,40158,40161,40164,40174,40185,40187,40191,40192,40193,40202,40203,40208,40209,40212,40219,40228,40241,40267,40291,40294,40296,40303,40304,40323,40324,40325,40328,40331,40341,40346,40349,40363,40380,40383,40402,40419,40423,40432,40438,40447,40454,40459,40468,40471,40479,40483,40492,40497,40502,40504,40511,40535,40540,40550,40558,40559,40614,40617,40629,40633,40640,40660,40715,40728,40736,40737,40739,40741,40742,40753,40757,40761,40776,40784,40785,40791,40794,40796,40797,40798,40806,40807,40814,40822,40823,40840,40845,40853,40856,40877,40878,40883,40885,40886,40887,40892,40893,40894,40911,40915,40939,40940,40952,40954,40957,40968,40982,40984,40985,40992,41002,41008,41010,41014,41024,41039,41041,41043,41044,41048,41058,41061,41064,41070,41071,41076,41079,41080,41084,41113,41118,41123,41136,41138,41158,41161,41177,41180,41187,41189,41195,41200,41203,41211,41230,41236,41240,41245,41256,41257,41264,41265,41269,41277,41278,41281,41293,41325,41327,41330,41332,41337,41343,41348,41354,41357,41371,41392,41393,41398,41411,41414,41415,41418,41428,41441,41446,41453,41459,41468,41472,165248,129239,129237,129235,129210,129209,129207,129206,129205,129191,129177,129164,129158,129147,129141,129139,129130,129118,129111,129089,129081,129069,129063,129055,129046,129042,129031,128991,128986,128974,128972,128969,128960,128954,128947,128944,128938,128933,128932,128923,128913,128906,128896,128893,129521,129514,129501,129491,129489,129484,129475,129473,129471,129469,129464,129460,129442,129438,129424,129409,129407,129404,129400,129379,129361,129359,129355,129350,129347,129340,163306,165775,140982,140967,140966,140965,140961,140945,140940,140938,140931,140928,140912,140907,140901,140895,140892,140874,140858,140855,140850,140847,140834,140831,140821,140814,140802,140792,140776,140775,140768,140746,140727,140710,140690,140676,140652,140643,140636,140635,140630,140919,140844,140765,140756,140696,140678,135277,135038,135130,135257,134969,134972,134973,134985,134988,134990,135002,135010,135024,135030,135034,135044,135052,135061,135075,135077,135081,135083,135104,135106,135114,135121,135128,135145,135147,135150,135152,135157,135160,135165,135174,135176,135181,135186,135196,135204,135205,135214,135221,135223,135233,135235,135238,135240,135247,135264,135267,135279,135282,135286,135287,135294,134930,134934,134951,134963,134966,157338,157334,157319,157315,157367,157364,157357,158957,158983,158988,158936,158968

Pop

FB-X-005, 0303 0202 0202 0204 0303 0204 0102 0404 0404 0202 0404 0103 0104 0101 0404 0204 0102 0204 0101 0404 0404 0103 0202 0202 0202 0203 0103 0101 0303 0204 0303 0303 0303 0204 0204 0202 0101 0202 0204 0103 0202 0202 0204 0404 0202 0202 0202 0202 0102 0202 0103 0202 0103 0101 0303 0404 0303 0303 0203 0204 0202 0303 0204 0202 0103 0202 0303 0204 0101 0204 0203 0404 0101 0404 0104 0303 0404 0103 0304 0102 0404 0202 0303 0103 0404 0103 0303 0204 0404 0303 0101 0202 0204 0204 0103 0102 0101 0303 0303 0202 0101 0202 0202 0202 0000 0303 0103 0101 0103 0303 0202 0404 0303 0202 0202 0404 0101 0202 0404 0101 0101 0103 0104 0000 0103 0203 0303 0404 0103 0101 0202 0204 0202 0303 0204 0404 0304 0202 0202 0102 0404 0101 0202 0303 0102 0303 0303 0104 0303 0102 0101 0404 0303 0202 0303 0303 0404 0303 0304 0202 0202 0103 0101 0404 0000 0203 0101 0202 0101 0101 0204 0101 0204 0303 0104 0101 0303 0303 0202 0303 0304 0304 0104 0103 0304 0202 0104 0303 0303 0202 0202 0202 0404 0303 0202 0303 0404 0202 0101 0303 0304 0404 0202 0202 0202 0202 0202 0202 0101 0101 0303 0202 0303 0204 0104 0202 0203 0101 0303 0103 0303 0404 0404 0202 0304 0404 0303 0203 0101 0202 0303 0101 0204 0204 0303 0303 0101 0303 0104 0404 0303 0102 0101 0303 0303 0404 0101 0404 0103 0102 0202 0204 0101 0101 0303 0404 0303 0101 0404 0404 0103 0102 0303 0204 0204 0101 0103 0204 0202 0104 0202 0303 0204 0104 0101 0104 0203 0303 0202 0404 0101 0303 0404 0202 0303 0204 0404 0204 0304 0303 0202 0202 0202 0303 0101 0204 0303 0101 0404 0101 0101 0204 0202 0203 0202 0303 0202 0303 0202 0303 0103 0204 0202 0202 0203 0404 0404 0202 0404 0204 0303 0101 0103 0404 0202 0204 0303 0404 0303 0304 0404 0202 0404 0404 0304 0202 0204 0303 0103 0203 0202 0303 0303 0404 0202 0101 0203 0101 0404 0303 0303 0202 0404 0404 0202 0104 0202 0101 0202 0101 0101 0203 0404 0202 0404 0202 0303 0404 0303 0204 0104 0202 0404 0303 0202 0102 0303 0303 0203 0303 0404 0102 0103 0303 0303 0202 0204 0303 0101 0202 0202 0202 0101 0103 0204 0103 0103 0303 0101 0404 0404 0303 0103 0303 0304 0303 0404 0303 0303 0202 0101 0404 0202 0102 0101 0104 0103 0303 0303 0000 0303 0101 0404 0303 0303 0303 0101 0104 0303 0103 0303 0204 0101 0204 0104 0202 0404 0101 0101 0204 0202 0202 0404 0202 0103 0303 0303 0202 0303 0404 0303 0000 0202 0304 0304 0303 0202 0202 0204 0202 0303 0202 0404 0202 0103 0404 0204 0101 0101 0304 0303 0202 0304 0404 0103 0303 0304 0404 0202 0204 0000 0304 0102 0101 0202 0103 0202 0202 0101 0202 0303 0103 0103 0404 0204 0303 0102 0101 0202 0204 0202 0101 0104 0101 0101 0303 0202 0303 0303 0101 0102 0103 0303 0202 0102 0103 0204 0101 0304 0104 0303 0203 0303 0404 0303 0202 0202 0101 0404 0303 0101 0101 0102 0101 0404 0102 0103 0103 0202 0202 0204 0303 0404 0101 0303 0101 0303 0202 0202 0202 0000 0204 0303 0202 0101 0303 0104 0101 0303 0404 0103 0404 0202 0404 0202 0202 0303 0203 0404 0303 0404 0303 0303 0103 0101 0404 0304 0204 0404 0204 0203 0103 0204 0204 0303 0101 0303 0303 0303 0404 0204 0101 0404 0303 0204 0303 0303 0102 0404 0101 0404 0202 0404 0303 0101 0404 0204 0404 0102 0202 0202 0404 0101 0202 0103 0101 0304 0202 0101 0101 0404 0101 0303 0202 0404 0101 0101 0202 0202 0101 0303 0102 0101 0404 0303 0101 0404 0202 0202 0202 0303 0404 0202 0202 0303 0303 0303 0102 0303 0303 0404 0202 0101 0303 0303 0101 0303 0404 0404 0101 0303 0202 0202 0103 0404 0103 0404 0303 0202 0404 0304 0404 0202 0202 0202 0101 0404 0303 0101 0101 0404 0303 0303 0404 0303 0204 0404 0101 0303 0103 0303 0101 0202 0101 0101 0303 0303 0101 0202 0304 0204 0304 0404 0203 0303 0303 0202 0404 0404 0202 0202 0404 0101 0404 0101 0202 0101 0202 0203 0103 0204 0101 0303 0404 0103 0404 0101 0202 0101 0202 0202 0101 0202 0103 0202 0303 0103 0202 0202 0303 0303 0303 0103 0404 0303 0202 0404 0202 0101 0103 0202 0202 0404 0202 0101 0202 0404 0202 0404 0404 0103 0303 0303 0404 0303 0303 0101 0203 0303 0101 0101 0303 0404 0202 0101 0404 0303 0304 0101 0202 0101 0101 0202 0202 0404 0303 0404 0404 0404 0404 0101 0101 0202 0404 0202 0404 0204 0103 0303 0303 0303 0202 0404 0202 0202 0103 0101 0101 0202 0404 0203 0101 0303 0202 0303 0103 0303 0202 0202 0103 0101 0404 0103 0404 0202 0203 0404 0202 0104 0101 0303 0203 0303 0101 0303 0104 0101 0101 0303 0103 0304 0404 0303 0101 0404 0101 0202 0202 0204 0101 0404 0202 0202 0202 0104 0103 0202 0202 0303 0101 0101 0404 0103 0204 0404 0101 0404 0304 0303 0102 0303 0404 0103 0303 0101 0204 0101 0303 0202 0404 0404 0202 0404 0303 0000 0102 0202 0404 0202 0203 0102 0101 0202 0303 0203 0103 0204 0203 0404 0202 0303 0202 0404 0202 0303 0104 0204 0404 0202 0202 0203 0404 0103 0303 0103 0404 0303 0202 0202 0104 0101 0202 0303 0303 0303 0303 0404 0303 0202 0202 0202 0202 0101 0404 0303 0404 0202 0104 0104 0404 0404 0202 0202 0103 0304 0102 0203 0303 0202 0404 0202 0104 0202 0204 0101 0202 0204 0303 0303 0103 0404 0204 0101 0303 0202 0103 0202 0202 0202 0101 0202 0404 0303 0303 0101 0404 0103 0303 0303 0202 0303 0101 0202 0101 0303 0101 0303 0303 0204 0204 0303 0303 0202 0103 0101 0304 0101 0202 0204 0101 0303 0202 0202 0104 0101 0303 0202 0203 0102 0404 0204 0204 0204 0303 0303 0101 0202 0204 0202 0101 0304 0202 0103 0303 0404 0404 0404 0303 0404 0202 0204 0404 0202 0202 0304 0101 0404 0202 0303 0103 0101 0103 0103 0404 0303 0101 0202 0202 0101 0303 0101 0202 0204 0303 0103 0101 0204 0103 0202 0101 0404 0202 0404 0202 0304 0204 0303 0303 0404 0101 0204 0303 0204 0404 0303 0101 0203 0303 0103 0404 0204 0404 0101 0303 0303 0102 0202 0304 0303 0103 0404 0202 0202 0303 0204 0202 0102 0203 0304 0101 0204 0202 0303 0404 0204 0303 0404 0101 0303 0303 0102 0101 0303 0104 0303 0202 0103 0103 0103 0304 0404 0204 0103 0204 0303 0202 0101 0104 0104 0303 0204 0303 0101 0404 0103 0101 0103 0101 0101 0202 0101 0202 0203 0101 0101 0101 0303 0101 0202 0303 0404 0303 0304 0202 0202 0303 0404 0404 0303 0204 0304 0202 0204 0203 0204 0202 0203 0103 0404 0204 0202 0303 0204 0202 0202 0303 0303 0101 0404 0303 0404 0202 0303 0202 0204 0204 0303 0204 0103 0101 0202 0203 0404 0404 0104 0304 0102 0404 0303 0404 0202 0103 0202 0101 0303 0101 0101 0101 0202 0101 0404 0202 0103 0404 0204 0204 0101 0202 0104 0101 0404 0204 0204 0202 0203 0202 0202 0202 0303 0202 0303 0303 0303 0202 0303 0303 0103 0101 0101 0303 0202 0303 0204 0202 0101 0303 0202 0102 0203 0202 0303 0303 0202 0202 0303 0303 0404 0203 0204 0303 0303 0202 0404 0000 0202 0303 0202 0304 0404 0101 0203 0101 0202 0303 0104 0203 0204 0303 0204 0104 0303 0303 0103 0101 0303 0303 0303 0103 0303 0204 0204 0303 0202 0102 0103 0404 0202 0204 0204 0104 0204 0202 0103 0304 0404 0203 0202 0104 0101 0404 0303 0404 0202 0101 0202 0303 0404 0303 0404 0303 0303 0202 0102 0204 0303 0101 0303 0204 0303 0303 0303 0303 0303 0103 0102 0304 0404 0303 0202 0404 0204 0102 0304 0303 0202 0104 0404 0404 0203 0202 0303 0104 0103 0404 0404 0101 0104 0303 0202 0303 0103 0404 0101 0202 0202 0202 0303 0404 0303 0202 0303 0101 0404 0101 0202 0303 0204 0303 0303 0101 0104 0101 0404 0303 0202 0103 0404 0303 0202 0404 0202 0303 0202 0404 0202 0103 0404 0102 0101 0101 0101 0303 0304 0304 0404 0304 0101 0104 0101 0304 0102 0404 0204 0101 0204 0103 0303 0202 0204 0202 0303 0202 0303 0101 0102 0404 0303 0101 0101 0101 0303 0103 0404 0204 0404 0404 0203 0202 0304 0103 0101 0303 0404 0303 0404 0404 0202 0303 0102 0204 0204 0104 0202 0202 0303 0104 0303 0103 0404 0303 0202 0202 0303 0404 0203 0404 0202 0404 0101 0103 0303 0303 0303 0303 0303 0203 0404 0303 0101 0104 0303 0102 0204 0202 0404 0101 0303 0204 0102 0303 0404 0303 0404 0202 0203 0303 0202 0202 0303 0102 0103 0103 0204 0103 0404 0303 0204 0303 0202 0303 0103 0103 0204 0404 0404 0103 0202 0202 0104 0202 0203 0303 0104 0202 0203 0102 0104 0202 0404 0303 0204 0303 0303 0304 0303 0303 0404 0303 0404 0202 0103 0202 0404 0101 0304 0204 0102 0404 0101 0404 0303 0101 0404 0000 0404 0303 0202 0303 0104 0103 0404 0404 0404 0101 0202 0303 0303 0404 0304 0303 0404 0202 0202 0303 0202 0202 0101 0103 0303 0404 0202 0404 0203 0303 0202 0202 0303 0101 0303 0204 0202 0101 0101 0101 0202 0202 0404 0303 0202 0303 0101 0202 0404 0202 0101 0202 0202 0404 0303 0404 0202 0101 0303 0303 0202 0303 0202 0404 0103 0102 0204 0101 0202 0103 0303 0202 0303 0404 0102 0303 0204 0404 0101 0104 0104 0202 0202 0404 0204 0202 0303 0202 0202 0101 0303 0404 0202 0404 0101 0202 0303 0202 0303 0104 0104 0303 0103 0303 0204 0101 0202 0303 0204 0101 0203 0404 0103 0104 0303 0404 0101 0202 0204 0204 0101 0104 0303 0404 0303 0204 0202 0303 0404 0303 0304 0202 0102 0404 0303 0202 0303 0202 0202 0303 0304 0303 0204 0202 0202 0303 0102 0404 0304 0103 0103 0103 0203 0101 0104 0202 0101 0101 0103 0303 0202 0304 0202 0103 0101 0103 0101 0202 0303 0101 0102 0103 0304 0303 0303 0203 0104 0104 0202 0303 0102 0101 0202 0202 0202 0404 0303 0303 0303 0303 0202 0404 0202 0303 0404 0101 0202 0404 0202 0202 0303 0104 0404 0404 0204 0303 0303 0202 0404 0202 0202 0204 0202 0404 0101 0202 0104 0303 0103 0303 0101 0103 0202 0303 0202 0303 0202 0203 0202 0202 0404 0102 0102 0202 0101 0202 0202 0404 0202 0304 0202 0303 0204 0101 0404 0303 0204 0103 0103 0101 0303 0202 0202 0202 0404 0202 0202 0101 0303 0101 0104 0404 0303 0303 0303 0404 0204 0103 0202 0104 0404 0404 0103 0204 0103 0303 0000 0303 0303 0101 0303 0104 0202 0101 0101 0104 0303 0103 0303 0404 0204 0303 0202 0202 0204 0303 0101 0202 0203 0303 0202 0303 0204 0304 0404 0303 0303 0204 0303 0202 0303 0303 0101 0303 0404 0304 0404 0204 0303 0404 0404 0101 0303 0202 0101 0202 0303 0202 0404 0103 0104 0404 0204 0303 0304 0101 0404 0204 0303 0103 0202 0104 0303 0101 0104 0303 0202 0101 0101 0202 0103 0202 0404 0101 0304 0101 0303 0404 0303 0404 0303 0101 0404 0404 0202 0404 0303 0202 0202 0304 0202 0303 0303 0303 0101 0404 0404 0303 0101 0303 0101 0204 0404 0101 0303 0101 0303 0101 0101 0204 0202 0102 0303 0104 0303 0202 0303 0303 0303 0303 0303 0404 0404 0202 0303 0202 0103 0404 0102 0404 0202 0303 0404 0202 0303 0303 0101 0101 0404 0202 0404 0101 0202 0404 0102 0202 0101 0304 0104 0202 0202 0104 0404 0101 0404 0202 0404 0101 0101 0101 0303 0202 0202 0202 0103 0103 0303 0202 0103 0204 0104 0202 0101 0404 0304 0103 0404 0404 0303 0303 0303 0101 0202 0202 0202 0202 0404 0202 0303 0101 0101 0204 0404 0202 0202 0303 0202 0103 0303 0202 0303 0204 0203 0102 0103 0202 0202 0202 0202 0202 0404 0303 0101 0404 0202 0102 0303 0101 0101 0303 0202 0303 0303 0303 0202 0303 0101 0303 0101 0303 0303 0202 0202 0202 0404 0303 0303 0202 0202 0404 0303 0404 0202 0103 0303 0101 0404 0404 0202 0303 0204 0103 0202 0101 0303 0101 0303 0303 0202 0202 0203 0404 0404 0101 0303 0202 0103 0303 0101 0303 0404 0103 0404 0303 0202 0304 0101 0404 0404 0103 0101 0203 0303 0202 0304 0303 0404 0202 0202 0404 0103 0202 0202 0303 0202 0203 0103 0202 0101 0404 0204 0103 0101 0404 0303 0404 0101 0303 0202 0303 0103 0101 0303 0202 0202 0101 0104 0204 0103 0303 0103 0102 0202 0101 0104 0202 0202 0404 0204 0104 0103 0303 0404 0204 0202 0203 0204 0204 0202 0101 0104 0101 0303 0104 0101 0101 0204 0202 0103 0404 0202 0204 0202 0204 0303 0404 0104 0404 0204 0404 0303 0404 0404 0101 0101 0203 0303 0000 0204 0202 0304 0000 0101 0102 0303 0404 0103 0303 0202 0101 0203 0204 0404 0404 0404 0101 0203 0202 0303 0404 0202 0204 0304 0303 0101 0101 0404 0101 0202 0203 0102 0103 0404 0103 0103 0101 0202 0103 0104 0103 0202 0203 0101 0101 0202 0202 0404 0303 0101 0404 0101 0101 0202 0303 0303 0202 0303 0000 0303 0204 0204 0202 0102 0103 0204 0303 0202 0404 0404 0303 0101 0202 0104 0202 0204 0404 0202 0103 0204 0101 0404 0104 0404 0304 0404 0103 0104 0202 0202 0101 0202 0103 0404 0103 0101 0303 0204 0202 0203 0303 0303 0404 0202 0102 0303 0202 0203 0404 0303 0404 0202 0101 0202 0404 0303 0101 0101 0303 0303 0202 0202 0303 0202 0303 0303 0202 0104 0404 0103 0303 0202 0303 0101 0203 0202 0202 0204 0202 0202 0303 0202 0103 0303 0303 0202 0202 0202 0202 0404 0103 0204 0202 0303 0303 0101 0104 0101 0303 0202 0202 0103 0404 0202 0203 0202 0404 0102 0202 0404 0203 0101 0101 0404 0404 0303 0404 0304 0202 0303 0102 0303 0202 0101 0303 0101 0202 0404 0303 0104 0202 0404 0303 0303 0404 0304 0202 0303 0104 0202 0202 0202 0103 0303 0202 0303 0303 0101 0303 0303 0101 0104 0101 0204 0304 0303 0101 0202 0404 0202 0303 0202 0404 0303 0202 0203 0204 0103 0204 0404 0103 0204 0202 0101 0202 0404 0202 0303 0102 0404 0303 0303 0304 0203 0202 0404 0303 0202 0101 0303 0202 0101 0303 0204 0303 0104 0204 0303 0204 0202 0101 0404 0303 0404 0204 0102 0404 0404 0304 0202 0104 0202 0103 0101 0303 0204 0101 0104 0104 0104 0101 0202 0202 0303 0404 0303 0101 0103 0304 0101 0303 0404 0202 0202 0303 0101 0202 0101 0404 0102 0101 0303 0101 0202 0103 0000 0202 0101 0101 0303 0101 0104 0102 0202 0202 0404 0404 0104 0202 0103 0303 0303 0202 0304 0202 0202 0303 0102 0202 0101 0304 0104 0303 0303 0202 0404 0303 0404 0202 0202 0102 0404 0101 0303 0104 0202 0404 0404 0404 0303 0303 0101 0103 0102 0303 0303 0404 0303 0101 0103 0103 0303 0404 0103 0101 0404 0304 0404 0101 0101 0101 0404 0103 0404 0404 0204 0304 0203 0103 0104 0103 0303 0204 0104 0404 0404 0202 0202 0101 0304 0101 0303 0204 0204 0204 0404 0101 0103 0204 0303 0404 0202 0202 0304 0303 0303 0303 0404 0404 0101 0303 0202 0303 0103 0204 0202 0303 0101 0104 0101 0101 0404 0404 0101 0202 0404 0303 0303 0303 0303 0404 0404 0202 0303 0304 0202 0303 0202 0202 0304 0202 0204 0202 0202 0101 0303 0101 0101 0404 0202 0101 0404 0303 0101 0101 0204 0104 0203 0303 0101 0303 0404 0303 0103 0202 0404 0101 0404 0303 0101 0202 0101 0202 0202 0404 0303 0202 0202 0104 0202 0404 0202 0202 0202 0101 0303 0103 0404 0303 0202 0202 0102 0404 0102 0202 0202 0303 0404 0303 0202 0202 0204 0303 0404 0303 0303 0404 0202 0303 0303 0101 0102 0202 0104 0204 0101 0204 0103 0303 0102 0202 0202 0404 0102 0303 0404 0303 0303 0303 0101 0303 0202 0303 0303 0404 0404 0303 0202 0103 0104 0303 0303 0202 0204 0202 0204 0203 0404 0404 0303 0104 0000 0202 0204 0404 0204 0303 0101 0202 0202 0303 0303 0202 0303 0304 0203 0202 0404 0404 0303 0202 0303 0104 0204 0303 0404 0303 0303 0404 0404 0304 0404 0202 0102 0103 0203 0101 0103 0303 0303 0303 0303 0202 0103 0202 0202 0101 0103 0104 0101 0404 0303 0104 0103 0202 0303 0303 0303 0303 0404 0202 0404 0202 0304 0404 0104 0101 0202 0303 0202 0104 0102 0202 0404 0203 0101 0303 0202 0404 0303 0204 0202 0404 0204 0202 0404 0202 0202 0404 0303 0104 0404 0101 0303 0202 0202 0304 0303 0103 0202 0202 0202 0404 0303 0103 0404 0101 0304 0303 0101 0204 0202 0102 0202 0404 0404 0202 0304 0404 0102 0404 0404 0204 0203 0202 0303 0202 0303 0101 0204 0404 0304 0204 0303 0101 0303 0404 0404 0404 0204 0303 0103 0202 0103 0104 0102 0101 0103 0101 0103 0101 0204 0404 0404 0101 0101 0101 0303 0404 0404 0404 0101 0204 0202 0202 0303 0404 0404 0104 0304 0101 0304 0404 0102 0202 0303 0202 0303 0202 0404 0404 0202 0204 0202 0101 0404 0204 0404 0202 0202 0202 0103 0104 0103 0101 0101 0203 0303 0202 0304 0404 0202 0101 0202 0204 0303 0101 0303 0404 0303 0202 0202 0404 0202 0202 0101 0202 0202 0202 0101 0303 0204 0202 0303 0102 0202 0303 0303 0202 0202 0202 0102 0102 0202 0303 0303 0204 0202 0303 0303 0101 0203 0404 0103 0204 0202 0103 0303 0202 0303 0101 0202 0303 0404 0103 0303 0204 0404 0101 0202 0404 0303 0303 0202 0101 0303 0101 0101 0404 0101 0303 0303 0303 0202 0202 0202 0303 0404 0101 0202 0202 0303 0202 0202 0101 0101 0303 0303 0202 0202 0101 0202 0101 0202 0204 0203 0404 0103 0303 0404 0304 0101 0303 0202 0102 0202 0404 0303 0204 0102 0202 0303 0303 0303 0404 0101 0303 0303 0202 0303 0103 0404 0303 0203 0404 0202 0202 0203 0202 0102 0404 0303 0202 0303 0404 0101 0303 0202 0304 0404 0101 0303 0303 0404 0404 0103 0303 0102 0202 0303 0303 0303 0404 0204 0103 0404 0303 0303 0304 0304 0404 0202 0104 0303 0104 0202 0101 0203 0101 0303 0304 0404 0303 0202 0103 0303 0303 0204 0404 0404 0202 0102 0202 0303 0202 0303 0102 0404 0303 0303 0303 0103 0303 0202 0303 0303 0102 0204 0103 0202 0303 0103 0303 0102 0303 0101 0202 0404 0404 0204 0202 0304 0202 0103 0101 0404 0303 0101 0203 0404 0404 0202 0303 0103 0202 0202 0104 0202 0202 0303 0101 0204 0303 0104 0101 0404 0303 0104 0101 0303 0303 0103 0303 0404 0104 0304 0203 0204 0101 0101 0202 0101 0304 0202 0303 0103 0103 0303 0303 0103 0303 0303 0202 0103 0101 0404 0101 0303 0202 0202 0303 0204 0303 0303 0103 0303 0103 0202 0404 0202 0204 0404 0203 0303 0204 0404 0404 0404 0202 0404 0101 0303 0303 0204 0101 0304 0202 0404 0303 0202 0303 0404 0303 0204 0101 0204 0101 0202 0202 0404 0101 0204 0101 0202 0404 0303 0303 0202 0203 0101 0103 0404 0303 0202 0404 0202 0303 0202 0101 0404 0303 0204 0203 0101 0303 0303 0404 0204 0404 0202 0202 0303 0102 0204 0303 0202 0404 0101 0101 0202 0404 0304 0202 0202 0404 0104 0404 0101 0303 0101 0303 0202 0303 0202 0303 0101 0202 0202 0101 0303 0101 0202 0303 0202 0303 0303 0101 0202 0101 0103 0303 0101 0102 0101 0404 0202 0202 0202 0202 0104 0303 0204 0103 0103 0202 0404 0404 0202 0103 0404 0103 0202 0101 0304 0404 0102 0103 0202 0303 0202 0404 0103 0404 0404 0404 0103 0103 0404 0101 0303 0303 0303 0303 0202 0103 0404 0404 0202 0304 0304 0204 0202 0202 0304 0304 0204 0204 0202 0101 0303 0303 0103 0204 0303 0303 0303 0204 0204 0303 0101 0404 0204 0404 0204 0101 0202 0202 0202 0202 0204 0202 0404 0303 0404 0202 0303 0204 0101 0104 0204 0303 0303 0103 0103 0204 0404 0202 0303 0303 0204 0202 0203 0404 0103 0202 0103 0203 0404 0202 0404 0101 0202 0303 0202 0104 0202 0304 0202 0404 0303 0101 0404 0404 0104 0202 0202 0104 0303 0202 0202 0404 0103 0303 0202 0303 0104 0303 0304 0202 0202 0103 0104 0202 0303 0202 0404 0303 0303 0303 0202 0404 0101 0304 0404 0102 0101 0102 0303 0101 0203 0303 0101 0204 0303 0101 0303 0202 0202 0303 0104 0404 0303 0202 0303 0103 0303 0202 0104 0202 0303 0404 0101 0101 0303 0202 0102 0101 0101 0103 0202 0000 0303 0202 0303 0204 0303 0303 0101 0202 0303 0303 0303 0204 0103 0202 0304 0104 0202 0404 0101 0303 0202 0103 0303 0101 0204 0101 0404 0404 0202 0203 0404 0202 0404 0202 0303 0202 0204 0303 0102 0303 0202 0203 0404 0101 0303 0101 0101 0101 0303 0404 0202 0204 0404 0204 0303 0303 0101 0303 0303 0303 0202 0404 0101 0303 0404 0101 0303 0202 0404 0304 0404 0303 0202 0103 0101 0103 0303 0204 0303 0204 0101 0202 0101 0202 0303 0303 0404 0304 0101 0102 0203 0303 0101 0202 0404 0303 0202 0303 0202 0202 0404 0404 0303 0404 0404 0303 0303 0404 0202 0404 0303 0404 0204 0101 0203 0103 0303 0101 0102 0204 0104 0202 0101 0202 0404 0202 0303 0103 0404 0101 0303 0404 0203 0202 0404 0103 0204 0202 0303 0304 0404 0303 0404 0102 0202 0202 0303 0404 0303 0103 0101 0103 0303 0204 0204 0303 0404 0202 0303 0202 0202 0101 0404 0303 0303 0104 0204 0203 0404 0404 0103 0204 0404 0404 0202 0202 0102 0204 0202 0202 0303 0101 0101 0303 0404 0202 0203 0404 0101 0202 0202 0202 0303 0103 0303 0303 0202 0404 0204 0101 0303 0202 0103 0404 0404 0101 0103 0303 0101 0303 0303 0303 0303 0303 0103 0304 0204 0404 0202 0404 0103 0104 0303 0303 0102 0304 0202 0202 0202 0303 0202 0202 0103 0202 0202 0101 0202 0203 0101 0102 0404 0303 0303 0303 0404 0101 0304 0202 0202 0202 0101 0303 0303 0303 0304 0404 0204 0404 0101 0101 0303 0303 0202 0202 0103 0404 0102 0304 0101 0303 0101 0204 0404 0104 0404 0101 0103 0104 0304 0202 0303 0102 0404 0404 0202 0101 0101 0202 0303 0404 0303 0101 0204 0202 0404 0202 0303 0303 0202 0101 0303 0103 0404 0404 0404 0202 0303 0202 0303 0303 0102 0101 0303 0303 0103 0404 0404 0202 0303 0303 0303 0404 0204 0202 0303 0101 0303 0103 0204 0202 0101 0101 0103 0101 0303 0404 0101 0404 0303 0101 0101 0404 0404 0303 0101 0404 0303 0404 0202 0202 0303 0404 0202 0202 0404 0303 0303 0204 0404 0303 0404 0202 0101 0101 0101 0103 0204 0202 0303 0202 0103 0202 0303 0304 0103 0103 0303 0303 0303 0404 0204 0101 0101 0303 0101 0202 0101 0101 0101 0303 0104 0101 0202 0404 0304 0404 0303 0103 0202 0404 0204 0303 0303 0202 0303 0204 0404 0202 0202 0101 0103 0102 0303 0202 0101 0101 0202 0303 0202 0303 0404 0204 0303 0404 0103 0303 0103 0101 0303 0202 0101 0101 0101 0101 0204 0101 0204 0101 0101 0202 0101 0202 0303 0202 0404 0203 0104 0404 0101 0202 0204 0202 0303 0303 0304 0303 0203 0304 0101 0404 0303 0202 0404 0304 0204 0103 0202 0303 0101 0404 0101 0103 0404 0404 0404 0204 0101 0101 0404 0303 0304 0101 0304 0103 0303 0101 0101 0303 0202 0303 0303 0404 0404 0404 0303 0303 0303 0103 0202 0202 0303 0404 0404 0202 0103 0101 0303 0303 0202 0204 0204 0303 0101 0204 0303 0303 0303 0202 0204 0101 0202 0303 0202 0101 0202 0202 0303 0303 0303 0404 0202 0202 0103 0303 0404 0204 0303 0101 0202 0404 0202 0204 0404 0202 0104 0103 0202 0404 0204 0204 0202 0303 0101 0303 0202 0404 0103 0101 0102 0101 0202 0203 0204 0202 0404 0404 0204 0101 0101 0303 0303 0101 0303 0101 0404 0404 0303 0303 0101 0103 0103 0303 0202 0303 0404 0101 0103 0404 0202 0303 0202 0303 0303 0303 0303 0404 0404 0404 0303 0404 0202 0404 0404 0202 0202 0303 0202 0101 0404 0101 0202 0202 0202 0101 0303 0101 0404 0103 0102 0101 0101 0202 0303 0202 0303 0303 0103 0202 0202 0404 0204 0204 0303 0404 0202 0000 0303 0202 0404 0101 0104 0303 0202 0101 0202 0404 0202 0203 0303 0202 0404 0303 0303 0204 0101 0303 0202 0202 0104 0102 0303 0203 0404 0202 0103 0303 0303 0404 0202 0101 0303 0202 0102 0101 0303 0203 0103 0202 0404 0404 0303 0202 0202 0202 0303 0101 0202 0202 0103 0203 0202 0303 0303 0303 0404 0103 0303 0304 0404 0103 0303 0303 0102 0202 0203 0303 0303 0101 0101 0303 0103 0303 0101 0304 0104 0303 0404 0404 0404 0303 0101 0404 0202 0202 0202 0404 0404 0101 0303 0101 0202 0204 0202 0104 0103 0000 0404 0303 0404 0202 0202 0303 0202 0303 0202 0202 0303 0303 0104 0404 0101 0202 0404 0103 0202 0404 0202 0404 0103 0303 0404 0404 0202 0204 0304 0303 0203 0204 0202 0202 0204 0103 0303 0304 0303 0303 0202 0304 0102 0101 0103 0103 0202 0202 0204 0303 0103 0202 0204 0204 0101 0202 0101 0101 0103 0101 0101 0202 0202 0204 0202 0404 0304 0203 0101 0203 0204 0303 0303 0303 0202 0304 0202 0101 0303 0202 0304 0101 0303 0101 0202 0303 0303 0404 0202 0202 0404 0202 0404 0101 0202 0103 0304 0104 0404 0202 0101 0204 0202 0101 0404 0303 0101 0202 0303 0202 0202 0103 0101 0202 0303 0303 0204 0304 0303 0303 0202 0404 0303 0202 0404 0404 0202 0204 0404 0404 0404 0202 0101 0404 0303 0303 0303 0303 0101 0404 0204 0104 0303 0404 0203 0000 0304 0101 0303 0204 0202 0303 0203 0303 0202 0101 0101 0202 0101 0101 0203 0303 0103 0103 0303 0202 0102 0404 0103 0303 0404 0202 0104 0404 0202 0404 0101 0203 0101 0202 0101 0101 0404 0202 0102 0303 0103 0202 0103 0404 0202 0202 0303 0203 0102 0303 0303 0404 0404 0101 0101 0101 0202 0202 0303 0204 0303 0104 0204 0304 0202 0202 0404 0202 0202 0203 0104 0202 0303 0303 0304 0204 0202 0103 0202 0204 0203 0303 0303 0303 0101 0103 0303 0104 0202 0202 0303 0202 0303 0304 0404 0101 0101 0101 0404 0202 0303 0102 0204 0101 0104 0103 0404 0101 0304 0203 0202 0202 0204 0102 0404 0102 0102 0404 0202 0103 0303 0303 0202 0303 0203 0103 0203 0303 0202 0101 0202 0202 0104 0202 0404 0103 0101 0204 0204 0204 0202 0202 0202 0202 0404 0202 0404 0303 0303 0101 0101 0303 0404 0303 0303 0202 0202 0404 0101 0202 0103 0303 0101 0102 0101 0202 0303 0101 0303 0303 0404 0104 0101 0303 0101 0104 0104 0303 0202 0303 0204 0101 0204 0202 0304 0303 0101 0202 0204 0102 0304 0103 0203 0303 0303 0202 0202 0204 0103 0103 0303 0303 0202 0303 0303 0203 0101 0202 0104 0202 0102 0404 0404 0101 0303 0303 0304 0203 0204 0303 0404 0202 0203 0101 0103 0101 0404 0103 0103 0101 0303 0303 0202 0202 0303 0202 0204 0303 0303 0404 0404 0202 0202 0204 0204 0101 0303 0202 0303 0202 0404 0104 0303 0101 0103 0103 0103 0404 0204 0303 0303 0404 0303 0303 0101 0103 0102 0404 0103 0202 0103 0303 0303 0104 0303 0103 0103 0303 0404 0303 0101 0303 0104 0101 0104 0204 0204 0303 0202 0101 0404 0101 0404 0204 0202 0404 0303 0202 0101 0303 0103 0202 0404 0202 0404 0303 0404 0404 0202 0101 0303 0404 0202 0101 0102 0404 0202 0102 0303 0101 0104 0202 0202 0103 0202 0202 0404 0101 0303 0202 0404 0204 0202 0404 0101 0303 0404 0303 0102 0000 0202 0101 0203 0303 0101 0404 0202 0101 0101 0404 0103 0304 0303 0202 0404 0303 0203 0303 0202 0101 0202 0303 0404 0103 0303 0202 0202 0101 0303 0101 0103 0202 0101 0404 0404 0202 0303 0202 0202 0303 0202 0204 0303 0204 0202 0101 0303 0101 0103 0303 0304 0404 0303 0103 0202 0404 0101 0303 0303 0303 0202 0202 0202 0202 0404 0304 0303 0103 0204 0303 0202 0202 0101 0101 0303 0202 0204 0202 0303 0204 0404 0303 0303 0303 0202 0303 0303 0104 0204 0103 0404 0304 0204 0303 0204 0303 0303 0101 0303 0101 0404 0303 0202 0202 0404 0101 0303 0303 0303 0103 0101 0203 0101 0101 0404 0202 0202 0404 0101 0303 0404 0202 0101 0101 0101 0303 0102 0404 0202 0101 0101 0203 0202 0103 0101 0404 0303 0101 0202 0303 0101 0202 0202 0202 0202 0101 0404 0202 0102 0303 0101 0404 0103 0203 0202 0404 0404 0103 0202 0303 0303 0202 0101 0203 0303 0101 0404 0303 0303 0303 0103 0404 0103 0203 0303 0303 0202 0202 0204 0202 0404 0204 0404 0303 0203 0303 0104 0202 0101 0101 0202 0101 0303 0202 0202 0101 0404 0202 0202 0202 0101 0303 0202 0303 0303 0404 0101 0202 0202 0202 0202 0404 0303 0303 0404 0404 0404 0404 0101 0404 0202 0303 0202 0101 0202 0101 0303 0103 0101 0303 0304 0104 0101 0303 0202 0101 0303 0202 0101 0202 0303 0304 0101 0204 0404 0304 0202 0404 0204 0303 0204 0202 0204 0404 0202 0102 0101 0303 0303 0202 0404 0202 0104 0303 0101 0103 0202 0202 0101 0303 0303 0101 0404 0303 0104 0101 0404 0202 0404 0104 0202 0101 0202 0202 0101 0404 0404 0404 0303 0103 0303 0303 0404 0103 0204 0103 0101 0404 0303 0202 0101 0202 0303 0202 0101 0104 0104 0404 0204 0303 0404 0303 0303 0303 0202 0304 0303 0303 0404 0203 0104 0202 0303 0202 0303 0202 0202 0202 0404 0104 0103 0101 0103 0104 0303 0102 0101 0104 0204 0101 0303 0101 0303 0101 0303 0202 0404 0303 0303 0202 0303 0101 0202 0303 0204 0104 0303 0101 0404 0404 0404 0202 0304 0202 0303 0202 0101 0303 0404 0103 0202 0304 0303 0202 0102 0204 0404 0103 0103 0104 0202 0101 0303 0404 0203 0102 0303 0303 0303 0204 0303 0103 0101 0304 0303 0404 0404 0203 0202 0303 0404 0202 0303 0303 0101 0303 0303 0204 0103 0303 0202 0303 0303 0404 0404 0303 0101 0202 0103 0303 0202 0203 0101 0204 0103 0303 0103 0102 0303 0203 0202 0303 0202 0202 0404 0103 0202 0404 0102 0102 0404 0101 0103 0101 0202 0404 0303 0202 0202 0404 0202 0102 0101 0101 0204 0303 0202 0104 0101 0101 0404 0202 0404 0104 0101 0101 0203 0202 0404 0404 0202 0404 0204 0303 0303 0202 0304 0303 0404 0102 0404 0101 0404 0202 0303 0202 0303 0303 0103 0102 0202 0202 0101 0204 0202 0404 0103 0202 0304 0404 0303 0202 0404 0204 0202 0303 0203 0404 0303 0102 0303 0303 0303 0202 0202 0404 0202 0404 0202 0204 0204 0102 0204 0202 0204 0304 0104 0303 0101 0202 0202 0204 0103 0303 0303 0304 0103 0303 0303 0202 0103 0101 0404 0404 0303 0404 0204 0303 0404 0101 0202 0404 0304 0101 0303 0101 0303 0101 0104 0404 0303 0404 0303 0404 0303 0101 0202 0303 0202 0101 0404 0204 0202 0303 0101 0202 0202 0404 0202 0104 0303 0303 0303 0202 0202 0101 0101 0303 0204 0103 0303 0404 0303 0303 0404 0204 0202 0202 0202 0202 0204 0303 0103 0101 0202 0303 0404 0102 0101 0303 0103 0101 0101 0101 0303 0404 0202 0101 0101 0101 0101 0202 0303 0202 0303 0103 0304 0101 0202 0303 0103 0303 0104 0303 0101 0303 0101 0202 0101 0303 0404 0404 0303 0202 0404 0102 0303 0103 0101 0303 0303 0202 0203 0404 0104 0204 0303 0404 0202 0101 0101 0101 0303 0303 0404 0204 0101 0202 0104 0404 0202 0303 0204 0303 0303 0101 0303 0404 0303 0202 0101 0303 0103 0202 0104 0404 0202 0404 0203 0202 0303 0404 0303 0303 0101 0202 0103 0303 0404 0304 0304 0303 0303 0102 0303 0103 0103 0101 0303 0404 0303 0204 0303 0202 0404 0303 0303 0101 0101 0303 0202 0303 0101 0103 0203 0303 0102 0404 0404 0204 0303 0303 0101 0303 0101 0304 0404 0303 0202 0204 0204 0103 0303 0202 0101 0104 0404 0202 0404 0202 0101 0202 0101 0103 0202 0101 0103 0404 0103 0204 0102 0101 0303 0304 0103 0104 0303 0303 0303 0101 0202 0304 0204 0404 0202 0303 0202 0202 0303 0202 0103 0202 0204 0101 0104 0404 0404 0304 0202 0202 0203 0303 0404 0204 0303 0101 0102 0203 0101 0103 0303 0202 0103 0101 0101 0204 0303 0303 0204 0204 0102 0202 0404 0000 0303 0102 0202 0204 0102 0202 0304 0303 0202 0203 0101 0204 0303 0304 0303 0303 0101 0202 0202 0103 0404 0303 0104 0303 0202 0303 0202 0304 0202 0204 0404 0103 0303 0202 0204 0102 0202 0101 0303 0303 0101 0202 0204 0103 0202 0202 0202 0103 0202 0202 0202 0202 0204 0203 0303 0202 0101 0101 0204 0404 0404 0303 0303 0304 0202 0202 0303 0202 0101 0204 0101 0204 0303 0000 0104 0101 0101 0404 0303 0202 0404 0202 0303 0304 0304 0303 0101 0103 0104 0202 0202 0102 0101 0303 0303 0202 0404 0102 0303 0303 0202 0104 0103 0101 0103 0103 0304 0103 0101 0303 0404 0304 0202 0101 0404 0303 0404 0404 0202 0202 0101 0404 0303 0404 0101 0202 0202 0101 0101 0304 0404 0202 0101 0404 0202 0101 0101 0204 0204 0303 0202 0101 0102 0102 0404 0101 0104 0404 0303 0104 0303 0303 0101 0202 0404 0303 0303 0101 0404 0202 0103 0304 0303 0101 0202 0101 0204 0303 0303 0103 0303 0303 0101 0203 0203 0303 0102 0101 0304 0101 0202 0304 0203 0101 0404 0203 0303 0204 0202 0404 0202 0101 0404 0104 0103 0202 0404 0101 0404 0303 0202 0204 0202 0101 0202 0204 0101 0202 0204 0202 0202 0101 0303 0101 0204 0101 0202 0303 0202 0103 0204 0202 0202 0202 0101 0202 0101 0101 0101 0103 0202 0304 0204 0103 0304 0204 0202 0102 0202 0101 0303 0104 0202 0303 0204 0202 0102 0102 0203 0101 0202 0101 0103 0202 0101 0304 0404 0101 0104 0202 0303 0202 0404 0101 0303 0202 0303 0303 0303 0404 0303 0104 0304 0303 0103 0204 0404 0202 0303 0303 0303 0303 0101 0202 0404 0303 0204 0202 0303 0202 0101 0404 0304 0303 0103 0202 0101 0303 0202 0101 0303 0404 0303 0101 0203 0202 0404 0202 0202 0101 0202 0102 0202 0102 0103 0404 0303 0404 0101 0303 0404 0202 0000 0103 0202 0104 0102 0202 0103 0102 0304 0101 0404 0103 0404 0303 0303 0101 0404 0202 0303 0103 0202 0204 0104 0204 0202 0303 0202 0101 0202 0303 0204 0404 0203 0103 0202 0303 0204 0404 0404 0404 0103 0204 0101 0102 0101 0101 0404 0204 0204 0303 0404 0202 0404 0404 0304 0303 0101 0103 0202 0202 0303 0303 0000 0204 0404 0102 0404 0101 0303 0303 0202 0202 0203 0303 0202 0102 0303 0104 0303 0101 0304 0404 0202 0303 0202 0202 0202 0303 0303 0303 0202 0103 0303 0304 0202 0103 0303 0303 0404 0202 0303 0103 0404 0303 0204 0303 0101 0204 0204 0202 0303 0404 0103 0404 0103 0101 0404 0404 0101 0404 0303 0101 0103 0101 0203 0101 0102 0404 0404 0404 0303 0404 0101 0304 0303 0404 0304 0103 0303 0303 0202 0303 0304 0103 0202 0303 0202 0103 0303 0404 0303 0202 0303 0102 0303 0404 0202 0204 0202 0303 0103 0103 0303 0204 0304 0404 0404 0202 0303 0303 0101 0404 0303 0202 0202 0101 0404 0101 0204 0103 0303 0304 0202 0101 0202 0202 0303 0204 0404 0303 0404 0204 0404 0303 0303 0202 0104 0202 0303 0102 0204 0404 0202 0104 0303 0303 0202 0404 0101 0202 0404 0404 0303 0202 0102 0202 0101 0303 0101 0202 0404 0202 0202 0202 0304 0202 0103 0204 0303 0303 0202 0202 0404 0303 0202 0202 0202 0203 0101 0102 0303 0303 0103 0304 0103 0404 0204 0404 0103 0303 0101 0204 0303 0202 0103 0303 0404 0101 0303 0103 0303 0303 0404 0404 0202 0203 0203 0104 0404 0101 0303 0303 0303 0104 0303 0404 0303 0202 0202 0204 0202 0202 0202 0303 0304 0101 0404 0202 0404 0101 0104 0303 0404 0103 0303 0404 0103 0103 0104 0202 0303 0303 0101 0303 0204 0204 0404 0303 0202 0204 0303 0202 0204 0202 0103 0103 0404 0101 0303 0101 0202 0303 0102 0104 0404 0203 0104 0202 0103 0303 0202 0202 0104 0404 0303 0101 0202 0303 0404 0202 0202 0303 0303 0102 0303 0404 0202 0101 0404 0204 0202 0303 0202 0202 0404 0303 0101 0303 0303 0202 0202 0202 0404 0303 0303 0303 0204 0103 0203 0101 0202 0303 0202 0202 0101 0303 0102 0202 0303 0303 0303 0303 0404 0404 0404 0202 0304 0202 0202 0404 0404 0404 0202 0303 0103 0303 0202 0304 0204 0404 0303 0303 0202 0303 0303 0202 0101 0303 0303 0303 0202 0204 0103 0303 0202 0303 0404 0101 0202 0202 0102 0303 0303 0303 0202 0204 0101 0101 0203 0203 0202 0204 0404 0303 0204 0202 0202 0204 0103 0404 0104 0102 0303 0101 0303 0404 0202 0104 0204 0303 0303 0202 0202 0000 0202 0101 0404 0303 0303 0303 0204 0202 0101 0103 0404 0304 0103 0303 0101 0202 0104 0202 0404 0404 0303 0102 0303 0303 0304 0303 0303 0202 0101 0303 0000 0101 0202 0202 0204 0202 0202 0304 0202 0101 0104 0404 0103 0303 0101 0304 0404 0303 0202 0202 0303 0203 0101 0202 0404 0202 0203 0101 0303 0404 0303 0303 0202 0303 0404 0204 0101 0103 0404 0202 0304 0202 0103 0202 0202 0101 0202 0202 0404 0202 0202 0204 0204 0204 0101 0303 0303 0101 0204 0102 0303 0202 0404 0202 0202 0101 0404 0101 0101 0103 0102 0101 0102 0102 0101 0303 0204 0404 0404 0404 0204 0101 0202 0101 0104 0101 0204 0204 0303 0303 0101 0202 0202 0404 0303 0303 0104 0404 0303 0404 0203 0203 0303 0303 0101 0101 0203 0404 0404 0103 0404 0101 0101 0204 0404 0202 0303 0101 0404 0202 0404 0303 0202 0202 0303 0101 0304 0101 0101 0404 0101 0404 0101 0204 0303 0404 0102 0204 0202 0101 0101 0404 0404 0103 0303 0202 0303 0103 0101 0101 0202 0303 0202 0204 0202 0101 0101 0202 0303 0101 0404 0102 0204 0103 0303 0202 0101 0204 0104 0204 0202 0404 0204 0303 0204 0303 0202 0303 0303 0303 0303 0102 0204 0303 0101 0303 0303 0404 0101 0101 0304 0404 0304 0303 0303 0202 0101 0103 0101 0204 0204 0101 0303 0103 0303 0303 0202 0103 0202 0303 0101 0303 0202 0202 0304 0404 0202 0202 0101 0101 0101 0303 0404 0303 0204 0404 0404 0101 0303 0203 0304 0204 0303 0303 0303 0303 0204 0303 0202 0103 0303 0303 0204 0303 0202 0204 0202 0103 0104 0101 0103 0202 0404 0202 0303 0303 0204 0101 0103 0303 0304 0103 0203 0101 0101 0104 0204 0103 0101 0404 0303 0202 0303 0404 0304 0103 0202 0303 0303 0204 0101 0404 0202 0304 0404 0404 0101 0404 0204 0202 0103 0303 0104 0101 0303 0101 0303 0303 0303 0202 0102 0303 0303 0101 0000 0102 0202 0303 0103 0202 0204 0202 0404 0404 0202 0303 0101 0404 0303 0103 0303 0103 0101 0303 0202 0202 0103 0204 0303 0101 0103 0204 0404 0103 0303 0101 0303 0303 0103 0303 0303 0404 0202 0404 0203 0101 0202 0303 0404 0101 0303 0202 0303 0202 0303 0404 0103 0404 0202 0303 0101 0404 0204 0304 0303 0202 0101 0202 0101 0203 0404 0101 0303 0101 0303 0404 0103 0101 0202 0303 0404 0202 0101 0404 0101 0202 0303 0303 0303 0303 0303 0101 0303 0202 0101 0102 0202 0101 0303 0103 0202 0202 0101 0101 0104 0404 0303 0303 0404 0202 0303 0303 0202 0203 0202 0203 0202 0204 0404 0404 0202 0202 0204 0202 0303 0202 0101 0202 0101 0101 0202 0101 0202 0101 0404 0101 0303 0204 0101 0102 0103 0204 0101 0303 0303 0303 0202 0101 0102 0101 0303 0304 0303 0404 0404 0202 0303 0303 0101 0204 0103 0303 0103 0303 0104 0101 0202 0202 0303 0101 0202 0101 0204 0204 0203 0101 0104 0202 0303 0104 0204 0304 0202 0101 0101 0101 0202 0303 0404 0404 0102 0202 0303 0101 0101 0303 0103 0104 0102 0303 0303 0202 0101 0101 0101 0202 0103 0404 0404 0204 0101 0101 0000 0203 0101 0303 0204 0404 0303 0303 0202 0304 0104 0404 0203 0103 0303 0101 0101 0203 0404 0304 0404 0103 0202 0202 0104 0101 0404 0101 0304 0104 0303 0104 0404 0202 0202 0404 0101 0404 0404 0404 0204 0104 0103 0404 0404 0202 0202 0404 0101 0104 0104 0202 0204 0303 0104 0202 0202 0101 0104 0101 0303 0202 0404 0303 0404 0303 0202 0404 0303 0202 0103 0404 0404 0303 0104 0404 0203 0103 0202 0303 0102 0303 0202 0204 0101 0303 0404 0101 0101 0202 0102 0303 0204 0303 0202 0303 0303 0202 0101 0303 0202 0202 0102 0103 0102 0303 0202 0204 0303 0304 0202 0103 0404 0303 0303 0103 0303 0404 0202 0303 0303 0303 0104 0202 0303 0103 0202 0204 0303 0101 0101 0202 0203 0204 0202 0202 0202 0303 0204 0204 0303 0404 0101 0404 0303 0303 0202 0202 0303 0202 0204 0404 0101 0304 0404 0103 0102 0101 0204 0101 0304 0404 0404 0104 0101 0101 0303 0102 0202 0101 0202 0102 0102 0404 0203 0101 0101 0101 0101 0203 0102 0404 0304 0102 0303 0404 0103 0202 0202 0202 0202 0303 0404 0103 0202 0404 0202 0103 0101 0103 0202 0303 0101 0303 0202 0103 0103 0202 0404 0101 0101 0101 0101 0102 0303 0404 0102 0102 0202 0303 0101 0303 0202 0204 0103 0101 0104 0202 0202 0204 0303 0303 0101 0304 0202 0303 0202 0303 0101 0303 0101 0101 0303 0204 0103 0404 0404 0101 0102 0404 0202 0101 0304 0304 0101 0404 0303 0202 0202 0303 0202 0303 0202 0103 0303 0202 0404 0204 0103 0204 0303 0303 0202 0303 0101 0202 0404 0102 0404 0404 0202 0404 0202 0102 0404 0303 0303 0101 0202 0204 0101 0303 0202 0202 0202 0101 0404 0101 0202 0101 0404 0202 0103 0102 0204 0101 0101 0204 0404 0202 0202 0204 0303 0303 0101 0102 0202 0101 0404 0404 0404 0304 0202 0303 0404 0104 0303 0101 0404 0102 0202 0202 0102 0202 0404 0202 0103 0104 0202 0404 0404 0303 0202 0404 0101 0202 0101 0202 0303 0204 0204 0404 0304 0101 0101 0104 0101 0202 0303 0303 0202 0404 0101 0303 0303 0202 0101 0101 0204 0203 0101 0104 0102 0303 0204 0303 0303 0303 0303 0103 0304 0204 0303 0102 0101 0202 0103 0204 0204 0202 0101 0303 0202 0303 0202 0404 0404 0102 0204 0303 0202 0303 0202 0202 0202 0202 0202 0404 0101 0202 0202 0103 0101 0204 0202 0104 0202 0101 0303 0303 0101 0103 0104 0202 0303 0303 0202 0404 0303 0303 0303 0101 0303 0404 0202 0204 0101 0204 0202 0303 0102 0202 0303 0303 0404 0204 0202 0101 0202 0303 0202 0303 0103 0303 0202 0202 0102 0303 0204 0303 0101 0101 0104 0204 0103 0103 0204 0204 0202 0103 0202 0303 0303 0202 0202 0303 0101 0204 0103 0204 0303 0404 0303 0204 0303 0202 0404 0101 0202 0303 0204 0101 0303 0303 0304 0101 0204 0204 0101 0202 0303 0404 0303 0101 0101 0101 0202 0104 0101 0101 0303 0303 0404 0303 0104 0101 0202 0404 0202 0101 0304 0101 0303 0101 0404 0404 0103 0101 0404 0303 0304 0303 0104 0104 0303 0202 0104 0303 0204 0101 0303 0404 0404 0303 0303 0303 0101 0304 0303 0101 0303 0303 0202 0404 0404 0101 0404 0303 0303 0102 0103 0101 0303 0102 0101 0202 0202 0404 0101 0303 0202 0202 0203 0202 0303 0404 0101 0202 0303 0404 0404 0202 0202 0404 0203 0104 0101 0101 0404 0202 0303 0202 0303 0101 0204 0204 0101 0303 0202 0303 0104 0101 0303 0404 0404 0103 0304 0101 0101 0202 0404 0303 0303 0303 0404 0202 0103 0204 0303 0404 0404 0101 0203 0101 0303 0303 0202 0101 0202 0303 0303 0202 0203 0103 0303 0101 0202 0404 0303 0304 0304 0204 0104 0101 0303 0202 0101 0102 0404 0202 0104 0000 0202 0204 0101 0404 0303 0103 0304 0404 0303 0303 0202 0202 0202 0303 0101 0101 0303 0303 0102 0101 0303 0101 0202 0303 0303 0102 0102 0202 0202 0303 0404 0204 0202 0303 0103 0404 0103 0404 0404 0303 0404 0101 0202 0103 0101 0404 0000 0202 0303 0203 0303 0404 0103 0101 0202 0303 0404 0202 0303 0202 0204 0101 0204 0202 0202 0404 0303 0202 0304 0204 0303 0203 0404 0304 0202 0202 0202 0202 0303 0102 0303 0101 0202 0304 0102 0303 0202 0103 0202 0202 0404 0103 0202 0303 0104 0404 0303 0202 0101 0303 0404 0303 0202 0202 0202 0102 0303 0102 0202 0304 0202 0304 0103 0303 0303 0404 0104 0203 0303 0102 0404 0101 0202 0202 0202 0103 0202 0303 0103 0101 0404 0202 0204 0101 0303 0304 0202 0404 0104 0303 0101 0204 0204 0103 0303 0103 0202 0404 0303 0202 0203 0404 0101 0202 0101 0103 0102 0103 0304 0202 0303 0101 0303 0202 0101 0202 0202 0202 0204 0202 0404 0303 0303 0404 0101 0204 0303 0404 0101 0101 0101 0103 0404 0404 0101 0102 0404 0204 0202 0101 0101 0202 0102 0204 0104 0000 0102 0101 0202 0303 0101 0103 0103 0202 0203 0202 0404 0202 0404 0202 0303 0404 0102 0202 0404 0303 0103 0101 0204 0303 0304 0404 0202 0404 0202 0303 0404 0202 0303 0102 0103 0202 0404 0404 0404 0101 0101 0202 0404 0404 0404 0103 0101 0303 0204 0202 0202 0404 0104 0404 0303 0103 0204 0103 0203 0303 0101 0202 0101 0103 0304 0000 0204 0202 0103 0103 0202 0102 0202 0303 0404 0202 0202 0202 0103 0303 0303 0101 0101 0202 0303 0101 0204 0202 0202 0202 0404 0202 0101 0202 0204 0304 0204 0202 0404 0104 0101 0204 0104 0303 0202 0202 0404 0103 0203 0103 0204 0303 0404 0204 0202 0202 0203 0303 0102 0204 0303 0202 0404 0102 0202 0101 0204 0404 0202 0102 0101 0103 0404 0101 0101 0101 0101 0303 0303 0204 0204 0202 0404 0104 0303 0303 0202 0101 0204 0404 0303 0202 0103 0404 0303 0404 0203 0203 0101 0202 0204 0303 0303 0303 0104 0303 0101 0404 0404 0101 0202 0404 0404 0101 0303 0103 0103 0404 0304 0303 0101 0000 0303 0303 0303 0204 0404 0204 0303 0104 0103 0404 0202 0202 0404 0204 0101 0202 0104 0303 0303 0404 0202 0103 0404 0404 0101 0101 0404 0303 0404 0404 0202 0404 0102 0202 0202 0202 0303 0202 0101 0404 0204 0404 0101 0303 0304 0202 0101 0304 0404 0404 0101 0103 0202 0101 0404 0102 0102 0303 0103 0101 0404 0303 0204 0404 0204 0204 0202 0202 0204 0404 0303 0202 0102 0303 0202 0303 0303 0202 0101 0203 0303 0303 0103 0104 0103 0404 0304 0404 0102 0101 0204 0303 0101 0101 0303 0102 0204 0202 0101 0204 0404 0204 0101 0103 0202 0102 0101 0303 0202 0404 0202 0303 0103 0202 0404 0202 0202 0303 0101 0202 0202 0202 0303 0303 0404 0101 0204 0303 0204 0102 0202 0202 0101 0303 0202 0404 0104 0202 0202 0303 0204 0103 0303 0404 0102 0404 0202 0304 0202 0404 0404 0203 0204 0303 0202 0304 0404 0404 0202 0103 0404 0202 0104 0104 0202 0202 0103 0202 0101 0104 0303 0101 0404 0101 0202 0101 0204 0404 0202 0000 0303 0101 0303 0303 0103 0202 0101 0202 0303 0404 0404 0404 0103 0303 0101 0101 0204 0202 0303 0303 0101 0202 0303 0202 0101 0103 0303 0303 0303 0101 0101 0303 0202 0101 0404 0202 0101 0101 0303 0404 0101 0404 0203 0404 0202 0404 0202 0202 0303 0204 0404 0101 0303 0404 0303 0101 0101 0202 0202 0303 0202 0104 0103 0103 0303 0104 0203 0303 0204 0204 0202 0303 0303 0101 0202 0101 0303 0303 0404 0101 0303 0103 0103 0202 0204 0202 0102 0101 0101 0404 0303 0404 0203 0404 0202 0303 0202 0404 0202 0303 0303 0303 0304 0101 0202 0101 0404 0101 0303 0202 0103 0404 0202 0101 0204 0303 0404 0303 0303 0101 0303 0104 0303 0404 0204 0304 0204 0204 0202 0202 0101 0303 0202 0202 0303 0202 0101 0303 0303 0303 0202 0202 0203 0303 0103 0304 0404 0303 0203 0303 0202 0404 0103 0202 0303 0103 0202 0303 0303 0202 0104 0101 0204 0303 0203 0404 0104 0101 0303 0202 0202 0101 0202 0404 0202 0103 0202 0101 0202 0404 0404 0303 0204 0303 0102 0303 0202 0101 0304 0202 0404 0202 0103 0303 0404 0404 0303 0101 0104 0303 0404 0404 0202 0202 0303 0104 0202 0203 0204 0202 0303 0103 0101 0103 0101 0101 0204 0202 0103 0404 0404 0303 0304 0304 0404 0404 0102 0202 0202 0202 0303 0202 0303 0204 0101 0202 0202 0404 0102 0202 0202 0102 0303 0202 0404 0101 0202 0204 0303 0404 0101 0204 0202 0202 0303 0101 0303 0204 0101 0202 0202 0104 0204 0101 0303 0303 0203 0404 0101 0204 0303 0303 0101 0101 0203 0303 0103 0202 0303 0404 0404 0202 0202 0204 0103 0202 0202 0202 0202 0103 0102 0404 0303 0202 0404 0404 0101 0202 0303 0101 0203 0303 0404 0102 0203 0202 0404 0202 0202 0102 0101 0103 0202 0103 0204 0104 0303 0303 0202 0404 0101 0202 0202 0102 0101 0203 0303 0202 0202 0101 0204 0303 0203 0204 0102 0101 0404 0101 0404 0404 0101 0404 0204 0202 0303 0202 0204 0104 0304 0404 0203 0104 0101 0204 0303 0101 0303 0404 0303 0202 0303 0104 0204 0404 0202 0202 0104 0103 0204 0104 0304 0000 0202 0202 0204 0404 0303 0204 0303 0404 0202 0303 0304 0202 0202 0303 0101 0304 0102 0303 0202 0202 0303 0202 0303 0303 0303 0103 0303 0404 0304 0204 0303 0203 0204 0104 0303 0204 0404 0202 0303 0202 0303 0104 0303 0202 0101 0101 0303 0101 0203 0404 0102 0303 0404 0202 0101 0303 0202 0104 0204 0303 0103 0404 0202 0303 0303 0303 0304 0203 0103 0404 0202 0303 0202 0202 0303 0404 0101 0303 0303 0303 0303 0202 0404 0101 0303 0101 0404 0404 0101 0404 0303 0102 0104 0204 0303 0303 0101 0404 0304 0404 0404 0101 0303 0303 0404 0102 0204 0303 0303 0101 0102 0202 0103 0202 0202 0202 0101 0202 0303 0204 0304 0303 0202 0204 0404 0204 0104 0303 0204 0101 0303 0101 0202 0101 0102 0303 0303 0202 0104 0204 0103 0304 0404 0303 0102 0304 0304 0404 0101 0202 0202 0202 0204 0303 0101 0202 0304 0202 0103 0202 0303 0203 0104 0303 0303 0101 0202 0101 0303 0104 0103 0203 0404 0303 0202 0202 0202 0303 0304 0103 0303 0101 0101 0304 0404 0202 0202 0202 0204 0101 0204 0103 0303 0202 0204 0404 0103 0202 0202 0101 0103 0404 0101 0203 0303 0202 0203 0404 0404 0101 0102 0304 0202 0303 0202 0404 0104 0103 0303 0404 0404 0303 0202 0404 0303 0304 0202 0304 0103 0204 0404 0202 0404 0102 0404 0202 0303 0204 0203 0204 0303 0203 0204 0304 0204 0404 0103 0101 0103 0304 0202 0204 0404 0404 0103 0204 0303 0202 0202 0202 0404 0101 0204 0404 0104 0404 0101 0202 0202 0303 0404 0202 0404 0103 0303 0204 0303 0104 0303 0202 0303 0101 0204 0404 0103 0103 0202 0303 0202 0404 0102 0204 0404 0303 0101 0101 0202 0404 0303 0202 0303 0303 0202 0101 0404 0303 0202 0202 0103 0202 0203 0101 0101 0404 0202 0404 0303 0101 0303 0202 0303 0103 0202 0202 0303 0303 0102 0202 0204 0202 0303 0202 0202 0202 0204 0101 0204 0101 0404 0102 0101 0404 0202 0102 0303 0202 0303 0303 0202 0101 0101 0404 0303 0303 0101 0202 0303 0204 0202 0101 0404 0202 0204 0202 0101 0404 0303 0202 0303 0202 0202 0404 0303 0101 0202 0303 0202 0404 0103 0202 0102 0202 0101 0202 0101 0102 0404 0404 0204 0101 0103 0202 0303 0303 0404 0404 0304 0101 0103 0303 0101 0102 0303 0303 0303 0103 0304 0202 0101 0202 0303 0101 0101 0104 0404 0102 0101 0202 0103 0404 0204 0404 0202 0202 0202 0101 0303 0303 0404 0202 0404 0303 0303 0202 0101 0303 0303 0103 0303 0103 0101 0404 0202 0204 0202 0202 0000 0404 0202 0103 0103 0303 0101 0404 0101 0303 0202 0303 0303 0303 0102 0304 0404 0101 0404 0304 0204 0204 0101 0303 0202 0404 0303 0202 0101 0203 0202 0202 0404 0101 0304 0202 0101 0303 0202 0202 0202 0204 0303 0404 0203 0204 0101 0404 0103 0101 0303 0202 0202 0404 0202 0104 0303 0304 0202 0103 0202 0404 0303 0202 0103 0103 0303 0303 0204 0202 0101 0103 0204 0303 0202 0303 0303 0204 0303 0101 0202 0202 0202 0202 0102 0303 0303 0203 0404 0202 0404 0101 0303 0101 0303 0303 0202 0202 0102 0101 0203 0101 0204 0101 0404 0203 0303 0303 0202 0202 0204 0103 0103 0204 0303 0303 0404 0404 0202 0303 0204 0404 0101 0104 0104 0202 0203 0202 0202 0202 0101 0202 0202 0202 0303 0203 0303 0103 0303 0103 0202 0404 0304 0404 0203 0202 0303 0204 0303 0404 0202 0202 0404 0303 0202 0303 0101 0202 0202 0404 0303 0101 0404 0404 0202 0202 0101 0202 0202 0404 0303 0202 0101 0101 0303 0104 0204 0102 0101 0304 0404 0204 0101 0303 0202 0102 0202 0303 0000 0204 0102 0303 0404 0101 0204 0101 0202 0303 0101 0203 0303 0202 0101 0404 0303 0202 0303 0202 0304 0104 0103 0101 0102 0202 0303 0101 0303 0202 0303 0202 0202 0202 0304 0204 0404 0101 0303 0101 0303 0303 0101 0303 0404 0404 0303 0202 0303 0202 0101 0404 0303 0101 0104 0404 0404 0202 0202 0202 0303 0303 0303 0203 0303 0202 0304 0404 0202 0303 0303 0202 0101 0404 0304 0101 0204 0000 0202 0202 0203 0101 0101 0303 0303 0202 0101 0203 0101 0404 0202 0202 0304 0101 0404 0202 0202 0303 0304 0103 0404 0102 0202 0202 0202 0303 0404 0101 0202 0303 0404 0101 0101 0404 0104 0103 0101 0202 0303 0202 0103 0104 0104 0303 0202 0101 0101 0204 0303 0202 0202 0202 0404 0404 0304 0202 0404 0202 0404 0101 0204 0101 0404 0202 0202 0202 0303 0303 0202 0202 0303 0202 0202 0404 0303 0303 0102 0101 0303 0104 0101 0404 0101 0101 0202 0000 0303 0204 0303 0202 0104 0404 0303 0304 0404 0101 0202 0204 0202 0303 0404 0202 0103 0101 0303 0404 0404 0303 0202 0202 0304 0303 0202 0101 0103 0103 0404 0404 0303 0404 0404 0103 0202 0204 0204 0103 0303 0404 0202 0203 0104 0303 0204 0202 0202 0204 0404 0303 0404 0101 0303 0204 0202 0303 0204 0202 0202 0202 0303 0103 0303 0303 0404 0404 0304 0303 0303 0102 0303 0103 0303 0101 0303 0101 0103 0103 0202 0304 0104 0404 0102 0404 0202 0204 0101 0303 0202 0303 0104 0103 0404 0303 0202 0303 0404 0303 0202 0303 0202 0202 0101 0101 0204 0404 0404 0103 0204 0101 0103 0103 0103 0103 0104 0104 0204 0202 0303 0101 0101 0404 0404 0304 0303 0203 0303 0303 0303 0204 0303 0101 0303 0404 0104 0202 0303 0103 0303 0202 0103 0204 0202 0101 0304 0101 0303 0303 0202 0203 0103 0102 0303 0202 0202 0101 0404 0102 0303 0404 0202 0304 0204 0303 0101 0103 0202 0303 0103 0202 0202 0102 0101 0101 0202 0202 0104 0304 0101 0101 0103 0303 0103 0101 0303 0404 0404 0404 0104 0404 0202 0202 0404 0202 0103 0101 0303 0101 0404 0101 0404 0103 0404 0103 0303 0204 0101 0404 0101 0202 0102 0303 0202 0404 0404 0103 0202 0303 0103 0303 0000 0202 0303 0202 0203 0101 0303 0102 0102 0101 0303 0202 0202 0202 0102 0204 0202 0202 0303 0303 0404 0101 0202 0202 0303 0404 0202 0204 0204 0204 0303 0202 0303 0101 0303 0404 0202 0304 0303 0101 0101 0303 0303 0202 0202 0303 0404 0404 0101 0104 0404 0103 0204 0202 0101 0102 0000 0303 0303 0404 0101 0101 0404 0103 0202 0202 0101 0101 0103 0101 0202 0101 0202 0103 0203 0303 0404 0303 0101 0202 0404 0404 0202 0404 0202 0304 0404 0404 0404 0303 0104 0203 0202 0202 0202 0404 0202 0303 0303 0202 0303 0303 0101 0202 0101 0202 0404 0101 0404 0202 0303 0101 0303 0102 0404 0103 0202 0204 0202 0202 0103 0303 0202 0101 0101 0303 0103 0303 0101 0101 0303 0404 0202 0102 0202 0101 0202 0102 0103 0203 0101 0101 0303 0204 0101 0404 0303 0202 0101 0102 0101 0202 0303 0404 0303 0202 0203 0303 0202 0303 0303 0103 0303 0404 0204 0303 0404 0103 0202 0303 0101 0303 0202 0101 0303 0101 0404 0202 0303 0303 0404 0202 0103 0304 0303 0304 0404 0303 0101 0404 0204 0103 0303 0101 0303 0404 0202 0304 0404 0101 0202 0404 0104 0101 0103 0103 0101 0202 0303 0304 0103 0404 0101 0101 0303 0303 0101 0303 0104 0103 0101 0404 0202 0103 0303 0303 0104 0303 0202 0303 0101 0304 0404 0404 0103 0303 0202 0202 0101 0203 0101 0103 0303 0104 0404 0303 0101 0101 0303 0101 0202 0203 0103 0204 0101 0202 0101 0202 0202 0202 0304 0204 0303 0102 0101 0204 0204 0303 0202 0203 0303 0202 0103 0303 0404 0303 0101 0202 0304 0204 0103 0404 0101 0103 0101 0303 0404 0204 0101 0202 0104 0202 0303 0101 0204 0102 0303 0202 0202 0101 0404 0101 0202 0103 0204 0202 0202 0404 0101 0202 0101 0103 0103 0202 0303 0202 0204 0304 0202 0104 0103 0204 0203 0303 0202 0204 0101 0303 0202 0104 0202 0202 0404 0101 0104 0101 0204 0303 0202 0204 0303 0204 0303 0202 0404 0203 0404 0101 0101 0404 0202 0202 0303 0303 0202 0202 0101 0303 0202 0204 0103 0303 0204 0303 0404 0203 0404 0202 0303 0204 0303 0304 0204 0103 0203 0404 0303 0101 0202 0101 0202 0303 0101 0304 0303 0303 0202 0204 0202 0404 0101 0202 0404 0303 0303 0101 0103 0404 0303 0101 0203 0303 0202 0202 0103 0404 0202 0101 0303 0204 0103 0303 0304 0202 0404 0202 0303 0204 0103 0102 0101 0203 0303 0102 0103 0303 0404 0101 0204 0101 0304 0101 0404 0303 0101 0303 0204 0404 0404 0303 0303 0303 0404 0303 0101 0202 0202 0101 0303 0204 0303 0103 0101 0202 0102 0103 0202 0404 0303 0202 0204 0204 0303 0202 0404 0404 0202 0303 0202 0303 0101 0303 0404 0303 0404 0202 0303 0204 0304 0202 0101 0202 0404 0202 0202 0404 0101 0103 0304 0303 0202 0101 0101 0103 0404 0101 0404 0104 0102 0303 0404 0204 0203 0404 0204 0303 0404 0202 0104 0102 0104 0404 0202 0404 0103 0104 0404 0101 0404 0101 0303 0204 0304 0204 0104 0101 0202 0101 0101 0303 0101 0101 0202 0202 0202 0404 0102 0303 0304 0202 0303 0203 0404 0303 0202 0202 0404 0102 0303 0303 0303 0202 0404 0404 0404 0303 0103 0404 0304 0203 0202 0202 0202 0202 0303 0304 0404 0102 0101 0103 0000 0303 0202 0101 0202 0101 0101 0204 0101 0103 0101 0404 0303 0303 0404 0202 0204 0101 0202 0404 0303 0404 0202 0304 0103 0404 0204 0202 0204 0102 0202 0303 0303 0404 0101 0202 0202 0404 0202 0303 0103 0103 0303 0303 0101 0303 0102 0304 0303 0204 0204 0303 0101 0404 0304 0204 0202 0404 0404 0303 0303 0101 0202 0000 0101 0404 0101 0202 0303 0202 0404 0101 0204 0303 0404 0303 0203 0202 0101 0103 0101 0303 0303 0204 0103 0303 0304 0202 0202 0202 0303 0202 0102 0103 0202 0202 0103 0303 0203 0104 0404 0101 0202 0404 0101 0304 0204 0303 0303 0303 0404 0102 0204 0404 0204 0203 0202 0303 0204 0404 0202 0202 0101 0404 0103 0303 0303 0203 0303 0104 0404 0202 0101 0202 0101 0304 0101 0204 0202 0204 0202 0304 0404 0303 0102 0303 0103 0303 0202 0204 0303 0204 0202 0102 0303 0101 0404 0202 0204 0303 0303 0202 0303 0101 0404 0404 0202 0202 0101 0202 0303 0303 0103 0202 0202 0204 0404 0104 0303 0203 0303 0104 0303 0203 0101 0101 0404 0303 0101 0202 0101 0202 0303 0101 0202 0404 0304 0204 0103 0202 0202 0103 0404 0202 0204 0202 0202 0103 0101 0303 0404 0103 0202 0404 0303 0404 0204 0404 0404 0303 0202 0202 0304 0103 0202 0404 0103 0204 0202 0303 0404 0202 0101 0404 0404 0103 0204 0202 0202 0202 0303 0202 0404 0303 0202 0202 0202 0404 0103 0101 0101 0103 0102 0101 0204 0404 0404 0303 0101 0202 0204 0303 0202 0404 0304 0101 0101 0202 0204 0101 0101 0101 0202 0203 0101 0101 0202 0303 0102 0202 0103 0404 0103 0202 0303 0202 0202 0101 0303 0404 0103 0101 0101 0404 0303 0101 0404 0404 0303 0203 0404 0104 0202 0101 0104 0303 0404 0101 0404 0202 0303 0103 0104 0404 0404 0204 0101 0404 0101 0303 0303 0203 0303 0101 0204 0303 0404 0101 0303 0303 0202 0202 0202 0204 0404 0303 0304 0303 0404 0404 0202 0101 0303 0303 0304 0303 0101 0303 0102 0202 0303 0303 0404 0303 0202 0404 0303 0101 0101 0103 0404 0101 0104 0203 0303 0303 0404 0404 0101 0101 0304 0104 0303 0202 0404 0202 0202 0103 0303 0203 0101 0404 0202 0404 0303 0303 0404 0202 0102 0202 0204 0404 0202 0404 0101 0404 0000 0104 0101 0101 0404 0101 0303 0303 0202 0404 0404 0303 0404 0303 0404 0404 0202 0102 0404 0202 0101 0303 0303 0303 0101 0303 0303 0303 0404 0303 0204 0202 0404 0303 0404 0304 0103 0104 0101 0202 0104 0303 0404 0404 0303 0101 0303 0404 0303 0303 0303 0303 0202 0101 0103 0204 0202 0202 0202 0404 0204 0202 0202 0204 0303 0204 0101 0303 0404 0203 0202 0202 0303 0103 0404 0202 0101 0303 0202 0404 0303 0202 0303 0202 0101 0303 0404 0103 0101 0303 0204 0404 0303 0104 0101 0103 0101 0204 0103 0101 0303 0101 0104 0202 0303 0303 0101 0102 0202 0304 0203 0103 0303 0101 0303 0202 0104 0103 0303 0204 0304 0404 0101 0103 0304 0303 0202 0303 0103 0303 0202 0304 0202 0103 0101 0204 0202 0303 0103 0204 0202 0101 0104 0202 0202 0204 0303 0204 0202 0202 0404 0203 0404 0203 0303 0202 0203 0101 0101 0303 0404 0202 0303 0404 0102 0101 0303 0103 0104 0202 0204 0202 0204 0202 0404 0103 0303 0203 0104 0202 0202 0202 0404 0404 0303 0202 0303 0204 0204 0203 0202 0202 0303 0101 0202 0202 0101 0303 0404 0103 0101 0404 0104 0204 0202 0204 0204 0102 0102 0404 0202 0103 0404 0404 0404 0102 0202 0303 0303 0202 0404 0202 0303 0404 0101 0404 0303 0303 0204 0303 0103 0303 0303 0404 0304 0303 0404 0104 0203 0101 0404 0202 0104 0404 0202 0102 0303 0303 0202 0303 0304 0104 0404 0202 0404 0303 0102 0202 0303 0202 0101 0103 0202 0404 0102 0202 0204 0202 0404 0202 0204 0101 0202 0101 0303 0303 0101 0303 0303 0102 0204 0303 0202 0303 0202 0103 0304 0404 0202 0303 0203 0104 0303 0303 0101 0404 0404 0101 0202 0404 0303 0303 0204 0204 0303 0303 0204 0204 0204 0303 0204 0101 0101 0303 0202 0203 0204 0304 0404 0304 0204 0101 0101 0104 0202 0101 0404 0102 0103 0303 0203 0202 0101 0101 0404 0102 0203 0101 0202 0104 0104 0202 0202 0104 0303 0103 0204 0101 0101 0303 0303 0101 0404 0204 0102 0103 0103 0202 0303 0204 0303 0101 0303 0404 0203 0303 0202 0303 0101 0202 0101 0404 0101 0101 0204 0101 0103 0202 0000 0102 0103 0303 0103 0202 0404 0404 0404 0303 0101 0404 0303 0404 0303 0103 0404 0202 0202 0404 0303 0202 0304 0404 0103 0202 0202 0404 0101 0101 0202 0303 0103 0101 0202 0101 0202 0303 0303 0404 0202 0103 0202 0101 0202 0202 0104 0204 0303 0303 0404 0404 0303 0303 0202 0202 0303 0102 0101 0202 0303 0303 0202 0102 0304 0202 0202 0303 0101 0404 0202 0202 0404 0202 0202 0303 0303 0404 0303 0203 0204 0202 0202 0101 0202 0304 0202 0404 0404 0202 0303 0303 0204 0303 0103 0202 0101 0303 0204 0303 0303 0404 0202 0404 0102 0202 0202 0101 0303 0303 0304 0304 0104 0303 0101 0404 0304 0303 0103 0103 0103 0101 0303 0404 0101 0404 0101 0303 0101 0101 0103 0101 0404 0101 0303 0303 0202 0203 0103 0202 0303 0303 0204 0303 0203 0404 0303 0101 0303 0202 0202 0303 0101 0303 0101 0404 0303 0102 0101 0101 0304 0303 0203 0303 0202 0202 0202 0303 0202 0303 0404 0203 0202 0103 0000 0101 0000 0101 0104 0202 0202 0303 0202 0101 0202 0202 0404 0101 0103 0303 0101 0203 0303 0203 0101 0101 0101 0101 0202 0202 0303 0404 0202 0102 0404 0404 0303 0303 0404 0101 0103 0303 0202 0204 0202 0303 0204 0103 0101 0103 0101 0202 0202 0202 0404 0303 0202 0202 0101 0203 0103 0404 0404 0303 0103 0204 0303 0404 0202 0303 0103 0102 0303 0101 0102 0303 0202 0202 0202 0404 0101 0202 0303 0303 0303 0303 0101 0303 0404 0102 0303 0202 0303 0103 0103 0303 0203 0202 0101 0404 0204 0101 0103 0202 0104 0202 0404 0202 0202 0404 0404 0303 0103 0101 0304 0303 0104 0202 0204 0101 0202 0102 0303 0303 0204 0404 0404 0303 0204 0202 0103 0202 0404 0203 0304 0204 0404 0204 0101 0204 0404 0101 0303 0303 0202 0404 0404 0303 0101 0104 0303 0404 0101 0101 0404 0404 0303 0303 0404 0303 0101 0404 0303 0404 0202 0202 0101 0103 0404 0202 0202 0303 0303 0103 0102 0202 0101 0202 0202 0303 0101 0101 0101 0404 0303 0303 0303 0303 0303 0101 0000 0304 0303 0404 0404 0304 0303 0202 0101 0202 0304 0202 0404 0202 0303 0303 0303 0104 0303 0404 0303 0202 0303 0103 0404 0303 0103 0303 0101 0303 0202 0101 0404 0101 0303 0104 0304 0304 0303 0404 0202 0303 0303 0303 0103 0202 0202 0203 0303 0101 0202 0101 0202 0101 0404 0404 0303 0404 0303 0202 0303 0303 0202 0202 0303 0404 0101 0202 0404 0404 0303 0103 0102 0303 0202 0404 0202 0101 0202 0303 0202 0204 0103 0204 0404 0204 0304 0303 0101 0202 0202 0202 0202 0202 0404 0303 0303 0303 0101 0303 0202 0404 0103 0303 0101 0203 0203 0101 0204 0202 0202 0104 0303 0404 0101 0303 0204 0103 0102 0204 0202 0202 0404 0202 0104 0101 0203 0101 0101 0101 0303 0202 0102 0204 0203 0202 0103 0101 0303 0103 0303 0102 0203 0202 0202 0303 0103 0404 0204 0102 0303 0303 0202 0000 0202 0404 0303 0303 0202 0404 0101 0204 0303 0303 0101 0404 0202 0101 0202 0404 0103 0104 0101 0404 0404 0104 0202 0303 0204 0104 0303 0202 0404 0101 0304 0104 0103 0202 0303 0101 0101 0303 0103 0104 0104 0203 0303 0101 0202 0103 0202 0404 0202 0204 0203 0304 0101 0101 0101 0303 0204 0202 0404 0303 0202 0303 0404 0303 0103 0404 0103 0304 0101 0101 0304 0304 0101 0204 0404 0303 0303 0303 0404 0202 0202 0101 0204 0202 0304 0404 0101 0202 0202 0404 0101 0404 0101 0202 0404 0202 0303 0404 0102 0303 0101 0404 0202 0404 0404 0101 0404 0101 0202 0303 0303 0202 0202 0203 0404 0404 0404 0103 0404 0102 0404 0303 0404 0101 0203 0303 0101 0303 0101 0204 0101 0303 0202 0304 0303 0303 0202 0204 0103 0303 0202 0103 0202 0101 0103 0101 0202 0101 0104 0304 0202 0303 0204 0204 0404 0101 0303 0202 0103 0303 0303 0101 0101 0102 0404 0202 0202 0101 0303 0101 0202 0102 0202 0303 0404 0103 0404 0304 0202 0202 0202 0101 0303 0101 0203 0303 0404 0303 0101 0404 0404 0202 0202 0103 0202 0102 0101 0304 0102 0203 0103 0404 0404 0101 0101 0202 0404 0202 0303 0202 0303 0404 0102 0303 0101 0103 0101 0303 0202 0404 0304 0103 0404 0103 0202 0102 0303 0202 0204 0101 0404 0304 0303 0303 0101 0202 0101 0303 0204 0104 0103 0404 0303 0303 0101 0203 0303 0103 0303 0303 0303 0101 0303 0103 0404 0204 0103 0303 0304 0204 0303 0304 0303 0202 0303 0404 0303 0303 0303 0202 0303 0303 0101 0202 0203 0101 0303 0101 0202 0404 0202 0101 0204 0304 0101 0204 0104 0101 0303 0303 0303 0202 0303 0202 0204 0303 0203 0202 0404 0202 0101 0303 0202 0102 0202 0404 0202 0103 0303 0204 0101 0000 0104 0202 0303 0103 0000 0101 0101 0202 0103 0101 0203 0204 0202 0202 0101 0404 0101 0104 0101 0404 0101 0404 0103 0101 0202 0303 0404 0204 0404 0202 0103 0202 0202 0102 0303 0404 0204 0203 0202 0304 0204 0404 0202 0102 0202 0202 0404 0202 0303 0202 0204 0202 0303 0101 0303 0202 0202 0304 0303 0103 0204 0101 0202 0202 0404 0404 0404 0103 0404 0303 0101 0202 0101 0102 0204 0202 0404 0202 0303 0404 0102 0103 0202 0103 0104 0202 0303 0204 0202 0202 0303 0104 0303 0404 0303 0202 0102 0202 0404 0204 0101 0202 0101 0404 0202 0202 0101 0303 0103 0104 0101 0404 0303 0404 0404 0303 0101 0303 0104 0103 0202 0204 0103 0103 0204 0102 0101 0204 0103 0101 0303 0202 0204 0404 0303 0000 0204 0404 0202 0303 0204 0303 0203 0202 0404 0202 0101 0101 0303 0202 0101 0101 0202 0203 0303 0303 0303 0101 0202 0202 0102 0304 0103 0303 0103 0204 0303 0404 0204 0203 0202 0204 0303 0404 0101 0101 0101 0103 0404 0303 0304 0202 0101 0404 0101 0303 0303 0103 0304 0404 0303 0101 0202 0303 0000 0404 0101 0204 0404 0202 0303 0103 0104 0404 0304 0204 0101 0303 0303 0104 0404 0203 0204 0103 0404 0101 0404 0104 0203 0404 0303 0101 0404 0404 0304 0103 0202 0404 0202 0303 0202 0404 0102 0103 0404 0303 0202 0303 0202 0303 0202 0404 0103 0202 0104 0304 0304 0303 0104 0203 0103 0202 0204 0404 0303 0101 0303 0204 0103 0104 0202 0204 0303 0202 0303 0204 0303 0101 0101 0303 0404 0101 0404 0303 0202 0101 0303 0303 0101 0404 0101 0404 0303 0404 0204 0203 0204 0103 0304 0202 0204 0101 0101 0101 0101 0303 0101 0101 0404 0303 0202 0304 0303 0202 0202 0203 0202 0104 0202 0303 0202 0303 0101 0103 0303 0303 0404 0103 0204 0202 0303 0203 0303 0102 0303 0103 0303 0202 0304 0101 0404 0202 0101 0103 0202 0404 0101 0103 0101 0103 0303 0303 0404 0404 0303 0404 0103 0101 0204 0203 0101 0103 0202 0404 0103 0303 0204 0303 0202 0304 0101 0303 0202 0303 0101 0204 0204 0104 0104 0304 0101 0101 0101 0204 0303 0204 0404 0202 0101 0202 0404 0303 0103 0404 0101 0202 0202 0202 0103 0202 0304 0303 0202 0102 0202 0101 0103 0104 0303 0303 0103 0202 0404 0202 0303 0404 0404 0204 0404 0204 0101 0103 0101 0303 0203 0303 0202 0202 0404 0202 0101 0101 0202 0202 0103 0303 0404 0202 0202 0101 0303 0103 0303 0404 0404 0204 0303 0104 0404 0303 0101 0104 0202 0404 0203 0204 0404 0202 0404 0104 0204 0101 0202 0102 0303 0303 0404 0303 0404 0404 0202 0303 0202 0304 0303 0303 0202 0101 0203 0101 0101 0101 0204 0404 0202 0000 0103 0404 0204 0204 0303 0303 0202 0101 0101 0203 0303 0303 0202 0303 0101 0101 0204 0101 0202 0202 0101 0404 0102 0104 0202 0101 0202 0202 0304 0404 0303 0404 0000 0404 0304 0101 0101 0101 0202 0404 0202 0101 0303 0204 0101 0404 0104 0202 0102 0101 0203 0404 0303 0303 0204 0102 0404 0202 0202 0303 0303 0303 0404 0202 0102 0303 0303 0303 0303 0103 0103 0101 0404 0303 0101 0304 0101 0404 0202 0101 0204 0104 0204 0202 0404 0303 0202 0202 0303 0202 0202 0303 0404 0303 0303 0103 0103 0103 0102 0203 0303 0404 0204 0404 0404 0404 0202 0202 0102 0303 0101 0404 0404 0101 0102 0202 0303 0303 0204 0303 0404 0303 0102 0202 0204 0101 0404 0202 0404 0204 0202 0303 0202 0203 0202 0101 0204 0101 0101 0303 0101 0303 0202 0204 0404 0101 0000 0404 0303 0404 0203 0101 0404 0101 0303 0404 0101 0404 0404 0303 0104 0102 0104 0304 0104 0203 0202 0404 0101 0202 0303 0202 0303 0101 0104 0304 0203 0303 0303 0404 0102 0303 0202 0202 0103 0304 0103 0304 0202 0204 0101 0202 0101 0303 0104 0203 0203 0303 0404 0102 0202 0101 0202 0404 0303 0303 0202 0303 0202 0202 0203 0103 0203 0404 0404 0404 0101 0303 0202 0103 0101 0303 0101 0104 0202 0202 0202 0304 0303 0101 0202 0404 0202 0101 0102 0303 0404 0101 0303 0404 0303 0404 0404 0102 0303 0104 0404 0202 0101 0303 0202 0204 0404 0103 0303 0404 0202 0303 0404 0303 0202 0101 0204 0104 0303 0202 0202 0202 0101 0101 0303 0101 0202 0303 0303 0204 0104 0202 0303 0101 0202 0101 0202 0303 0304 0404 0204 0104 0404 0202 0103 0101 0202 0404 0103 0000 0204 0102 0202 0303 0404 0102 0404 0303 0202 0303 0202 0101 0303 0104 0202 0303 0202 0202 0303 0202 0303 0404 0202 0303 0304 0404 0304 0103 0404 0101 0303 0101 0303 0404 0101 0202 0404 0101 0304 0303 0303 0404 0103 0202 0303 0204 0404 0204 0102 0101 0101 0404 0101 0404 0202 0202 0404 0303 0304 0404 0101 0104 0202 0101 0101 0202 0303 0202 0202 0103 0104 0202 0202 0103 0101 0202 0404 0202 0104 0202 0303 0104 0404 0204 0202 0404 0303 0104 0303 0404 0303 0203 0104 0303 0202 0101 0404 0303 0101 0303 0202 0202 0101 0303 0303 0103 0202 0202 0103 0303 0204 0103 0202 0202 0204 0303 0404 0202 0202 0204 0202 0101 0103 0202 0204 0303 0103 0202 0303 0202 0404 0101 0202 0202 0404 0202 0102 0303 0404 0203 0203 0303 0303 0000 0303 0103 0202 0303 0202 0101 0202 0101 0101 0202 0202 0202 0404 0101 0303 0101 0303 0204 0204 0404 0101 0404 0203 0202 0404 0101 0303 0303 0102 0202 0204 0202 0202 0202 0303 0404 0101 0404 0404 0202 0204 0303 0404 0204 0101 0304 0202 0102 0404 0103 0303 0203 0303 0204 0303 0202 0304 0202 0102 0204 0101 0202 0303 0404 0101 0202 0204 0303 0202 0202 0101 0404 0303 0303 0101 0404 0303 0204 0202 0303 0102 0303 0303 0303 0202 0202 0404 0101 0404 0104 0101 0404 0202 0101 0101 0202 0404 0103 0204 0404 0101 0202 0204 0202 0303 0103 0303 0101 0103 0101 0204 0303 0101 0101 0303 0101 0204 0204 0303 0104 0102 0202 0303 0204 0303 0202 0303 0202 0202 0202 0404 0204 0204 0303 0103 0101 0202 0202 0202 0204 0303 0202 0202 0104 0303 0103 0202 0202 0103 0303 0304 0202 0303 0304 0103 0101 0101 0303 0101 0103 0202 0204 0303 0304 0202 0303 0404 0404 0101 0103 0404 0101 0204 0404 0303 0101 0202 0303 0203 0202 0303 0202 0202 0404 0404 0101 0101 0101 0303 0000 0101 0103 0103 0202 0202 0404 0304 0101 0101 0303 0101 0101 0202 0101 0202 0204 0203 0404 0202 0404 0202 0101 0404 0202 0404 0304 0404 0303 0202 0101 0303 0404 0404 0103 0101 0202 0102 0303 0303 0102 0204 0202 0303 0404 0303 0202 0303 0202 0204 0303 0202 0204 0101 0202 0303 0303 0404 0202 0404 0101 0404 0303 0404 0103 0404 0101 0101 0203 0102 0204 0101 0303 0204 0304 0304 0202 0202 0404 0202 0104 0103 0303 0202 0202 0303 0303 0104 0202 0104 0202 0304 0303 0202 0202 0303 0101 0202 0101 0303 0101 0202 0303 0303 0203 0101 0202 0202 0104 0202 0202 0303 0404 0303 0304 0303 0103 0303 0203 0101 0103 0202 0202 0101 0202 0303 0103 0404 0303 0103 0204 0204 0202 0303 0404 0101 0303 0103 0304 0303 0101 0103 0103 0202 0101 0304 0404 0101 0101 0202 0204 0101 0204 0303 0404 0203 0204 0202 0303 0101 0101 0204 0404 0404 0101 0101 0404 0202 0204 0404 0102 0304 0202 0404 0304 0404 0404 0101 0104 0202 0102 0304 0104 0102 0103 0202 0101 0101 0202 0404 0404 0101 0204 0203 0303 0303 0202 0202 0303 0303 0404 0203 0101 0204 0303 0202 0303 0404 0203 0202 0303 0203 0204 0404 0103 0101 0404 0202 0202 0202 0203 0202 0304 0303 0404 0303 0101 0101 0303 0404 0101 0101 0303 0202 0303 0303 0303 0202 0101 0204 0303 0404 0303 0202 0303 0303 0203 0303 0404 0101 0404 0303 0404 0103 0202 0303 0404 0102 0203 0303 0101 0404 0204 0101 0202 0303 0202 0202 0303 0202 0102 0202 0103 0101 0202 0303 0202 0102 0202 0202 0104 0101 0203 0204 0303 0102 0202 0203 0202 0204 0204 0303 0404 0202 0202 0404 0103 0101 0303 0303 0101 0101 0101 0102 0101 0204 0303 0101 0202 0101 0303 0202 0101 0104 0204 0204 0303 0404 0103 0204 0303 0303 0404 0202 0202 0202 0303 0101 0404 0202 0202 0202 0404 0202 0202 0404 0101 0102 0404 0404 0202 0404 0202 0104 0303 0303 0101 0204 0204 0303 0304 0101 0202 0202 0404 0101 0202 0303 0303 0101 0303 0404 0104 0103 0103 0303 0103 0202 0101 0303 0102 0404 0303 0404 0303 0303 0303 0202 0404 0303 0101 0101 0202 0303 0202 0202 0303 0202 0303 0202 0104 0104 0202 0202 0303 0101 0103 0101 0303 0404 0104 0202 0404 0203 0303 0101 0303 0103 0101 0202 0303 0404 0202 0204 0202 0202 0101 0204 0303 0303 0101 0101 0101 0202 0404 0404 0202 0303 0404 0102 0103 0202 0304 0204 0303 0303 0101 0000 0404 0101 0404 0202 0104 0303 0202 0204 0304 0202 0101 0101 0404 0202 0101 0303 0303 0203 0101 0204 0101 0103 0303 0303 0303 0303 0202 0404 0202 0404 0404 0303 0303 0101 0202 0404 0203 0202 0404 0202 0404 0104 0202 0202 0303 0304 0304 0204 0103 0203 0103 0404 0303 0202 0303 0303 0202 0303 0404 0101 0101 0101 0303 0303 0101 0404 0202 0202 0404 0404 0303 0202 0101 0101 0104 0202 0103 0202 0404 0103 0303 0303 0303 0202 0101 0202 0202 0303 0101 0404 0204 0202 0303 0404 0101 0101 0303 0404 0202 0303 0204 0202 0202 0204 0103 0303 0404 0404 0202 0404 0101 0404 0203 0101 0303 0101 0303 0404 0101 0404 0304 0404 0101 0303 0404 0404 0404 0303 0203 0303 0303 0000 0101 0404 0303 0304 0103 0101 0303 0202 0404 0404 0101 0202 0202 0303 0404 0202 0303 0202 0101 0103 0203 0102 0404 0303 0303 0303 0101 0404 0101 0204 0204 0404 0101 0101 0404 0404 0204 0103 0404 0303 0101 0101 0104 0101 0102 0404 0204 0204 0204 0404 0202 0204 0303 0404 0404 0101 0204 0304 0202 0303 0202 0101 0202 0404 0104 0102 0102 0303 0104 0303 0404 0303 0303 0102 0303 0202 0204 0202 0202 0202 0202 0202 0101 0101 0101 0101 0303 0101 0404 0103 0101 0204 0303 0103 0303 0101 0104 0202 0102 0202 0101 0303 0202 0101 0202 0202 0303 0204 0101 0101 0202 0101 0204 0404 0404 0101 0303 0303 0101 0204 0202 0204 0404 0303 0404 0202 0103 0104 0103 0202 0101 0404 0203 0202 0203 0303 0101 0101 0104 0202 0104 0303 0103 0202 0202 0202 0202 0304 0203 0202 0204 0202 0404 0101 0404 0103 0303 0303 0303 0204 0101 0103 0202 0103 0101 0101 0101 0104 0304 0303 0202 0404 0202 0202 0404 0202 0303 0404 0101 0304 0103 0101 0204 0202 0304 0101 0102 0404 0404 0204 0202 0303 0104 0202 0304 0204 0101 0202 0202 0202 0101 0202 0303 0204 0203 0303 0303 0404 0202 0101 0101 0303 0102 0202 0303 0101 0101 0202 0202 0202 0303 0204 0204 0101 0303 0303 0101 0404 0303 0204 0303 0204 0204 0203 0204 0203 0303 0103 0202 0404 0204 0102 0101 0203 0303 0202 0101 0404 0101 0202 0101 0101 0101 0202 0303 0303 0203 0203 0103 0202 0404 0103 0101 0404 0101 0101 0202 0102 0404 0101 0101 0102 0101 0304 0103 0101 0404 0303 0202 0404 0202 0303 0303 0104 0404 0303 0303 0202 0304 0303 0303 0404 0103 0101 0202 0303 0101 0303 0303 0303 0202 0404 0303 0202 0404 0303 0202 0101 0303 0204 0202 0404 0101 0103 0404 0303 0102 0303 0303 0303 0101 0202 0202 0102 0404 0203 0204 0102 0303 0404 0202 0101 0303 0103 0102 0202 0303 0202 0202 0104 0000 0202 0404 0303 0203 0303 0303 0101 0103 0103 0303 0304 0404 0304 0303 0204 0102 0202 0202 0202 0101 0303 0203 0204 0304 0103 0202 0303 0303 0304 0101 0202 0103 0404 0202 0303 0404 0404 0303 0204 0101 0202 0202 0103 0303 0101 0303 0104 0202 0404 0101 0404 0303 0303 0101 0204 0303 0204 0202 0103 0303 0204 0204 0202 0204 0103 0104 0103 0103 0202 0104 0103 0202 0204 0103 0104 0101 0404 0104 0303 0204 0304 0202 0303 0202 0204 0101 0202 0303 0202 0404 0303 0303 0204 0303 0204 0304 0404 0303 0203 0404 0102 0101 0303 0101 0404 0202 0101 0404 0202 0203 0202 0303 0303 0404 0204 0203 0303 0204 0103 0404 0202 0103 0103 0404 0303 0303 0101 0404 0103 0103 0303 0303 0202 0303 0204 0202 0204 0202 0101 0202 0104 0101 0202 0303 0303 0103 0202 0303 0303 0404 0103 0303 0202 0203 0404 0104 0404 0303 0304 0303 0101 0204 0202 0101 0202 0304 0303 0101 0203 0202 0202 0101 0202 0101 0404 0202 0104 0404 0303 0202 0303 0303 0202 0204 0304 0303 0102 0404 0303 0203 0404 0202 0404 0204 0102 0202 0404 0202 0000 0101 0303 0202 0202 0404 0404 0202 0101 0202 0101 0303 0103 0103 0202 0404 0404 0303 0303 0203 0202 0103 0304 0404 0203 0404 0404 0202 0103 0101 0204 0202 0303 0404 0202 0202 0303 0202 0404 0303 0203 0202 0303 0101 0101 0104 0202 0303 0102 0303 0101 0202 0304 0303 0101 0303 0304 0303 0303 0101 0204 0202 0204 0101 0202 0204 0103 0101 0204 0101 0202 0202 0404 0303 0303 0102 0404 0101 0303 0404 0202 0303 0204 0101 0102 0202 0101 0404 0202 0404 0101 0303 0404 0404 0203 0202 0404 0202 0303 0101 0202 0101 0303 0303 0303 0101 0101 0303 0202 0202 0202 0101 0303 0404 0204 0203 0304 0404 0103 0303 0202 0202 0202 0304 0202 0202 0304 0204 0202 0404 0404 0101 0404 0303 0303 0203 0404 0101 0203 0101 0103 0202 0404 0303 0101 0204 0104 0204 0202 0204 0203 0303 0101 0103 0101 0202 0303 0202 0202 0202 0202 0304 0202 0204 0204 0203 0303 0303 0303 0204 0303 0000 0303 0103 0202 0404 0404 0101 0404 0202 0202 0103 0203 0202 0203 0101 0101 0303 0202 0303 0101 0203 0204 0303 0303 0404 0404 0303 0204 0202 0303 0101 0404 0202 0304 0303 0104 0404 0204 0202 0303 0101 0203 0303 0202 0203 0404 0303 0303 0303 0304 0204 0103 0104 0404 0202 0104 0202 0103 0202 0303 0303 0404 0303 0303 0103 0104 0101 0202 0204 0404 0202 0303 0103 0303 0103 0404 0303 0303 0303 0303 0101 0404 0404 0202 0303 0104 0404 0101 0204 0202 0404 0202 0103 0202 0303 0303 0101 0404 0102 0103 0303 0202 0101 0104 0303 0101 0303 0202 0303 0103 0101 0303 0303 0202 0202 0404 0303 0101 0202 0404 0101 0303 0404 0202 0101 0303 0303 0304 0101 0102 0101 0404 0202 0202 0202 0303 0202 0303 0303 0303 0103 0203 0303 0101 0404 0303 0104 0202 0202 0303 0202 0202 0202 0303 0303 0303 0104 0202 0303 0404 0101 0101 0202 0103 0202 0303 0404 0304 0404 0202 0103 0202 0102 0103 0303 0304 0202 0101 0404 0101 0303 0202 0303 0404 0303 0101 0104 0101 0203 0101 0304 0204 0404 0202 0404 0303 0404 0101 0204 0303 0304 0404 0202 0202 0202 0303 0202 0204 0303 0303 0202 0303 0203 0303 0101 0404 0202 0202 0304 0202 0202 0202 0101 0404 0101 0404 0303 0204 0202 0104 0404 0303 0101 0303 0101 0102 0404 0101 0404 0202 0303 0202 0303 0404 0101 0202 0101 0303 0202 0101 0202 0404 0101 0404 0104 0202 0000 0101 0101 0404 0203 0404 0101 0202 0304 0303 0303 0404 0204 0101 0204 0303 0404 0404 0304 0104 0204 0202 0204 0303 0101 0101 0303 0204 0103 0101 0204 0202 0404 0103 0204 0203 0303 0101 0202 0404 0102 0103 0303 0202 0204 0101 0202 0404 0101 0104 0101 0102 0101 0404 0303 0202 0204 0101 0103 0303 0202 0101 0102 0101 0202 0104 0303 0404 0404 0202 0303 0303 0303 0101 0202 0202 0202 0204 0101 0102 0103 0101 0303 0202 0304 0202 0202 0103 0202 0404 0202 0303 0404 0404 0103 0303 0202 0304 0404 0404 0404 0303 0104 0102 0202 0202 0202 0303 0203 0202 0303 0101 0101 0202 0101 0103 0204 0404 0404 0202 0103 0303 0202 0202 0102 0304 0202 0404 0303 0404 0103 0202 0202 0303 0303 0303 0202 0303 0404 0404 0202 0101 0101 0404 0102 0101 0204 0303 0404 0204 0101 0101 0104 0202 0202 0303 0404 0202 0303 0202 0404 0103 0404 0101 0202 0304 0101 0303 0203 0303 0102 0303 0404 0303 0203 0304 0202 0204 0202 0101 0103 0404 0303 0303 0204 0404 0303 0103 0303 0304 0303 0104 0101 0303 0303 0204 0204 0104 0202 0303 0404 0404 0103 0303 0103 0303 0303 0202 0103 0303 0404 0303 0101 0202 0101 0404 0303 0101 0304 0404 0203 0203 0303 0303 0404 0102 0202 0202 0101 0101 0103 0202 0204 0103 0404 0303 0404 0202 0303 0404 0303 0303 0404 0101 0101 0303 0204 0101 0202 0303 0204 0303 0304 0404 0102 0303 0204 0202 0303 0303 0303 0202 0202 0101 0404 0203 0404 0101 0202 0103 0303 0101 0101 0103 0303 0202 0202 0304 0202 0104 0404 0104 0303 0303 0303 0303 0202 0101 0303 0102 0202 0202 0404 0303 0103 0303 0202 0203 0404 0202 0202 0404 0202 0303 0204 0202 0103 0303 0404 0404 0202 0104 0202 0101 0303 0104 0303 0202 0101 0103 0202 0202 0404 0202 0102 0204 0101 0101 0204 0101 0404 0102 0103 0304 0303 0202 0202 0202 0404 0303 0202 0103 0203 0303 0101 0404 0101 0204 0303 0101 0404 0101 0303 0404 0202 0202 0303 0101 0202 0101 0303 0404 0202 0103 0202 0101 0202 0101 0101 0303 0202 0202 0404 0202 0202 0202 0202 0104 0102 0202 0204 0103 0303 0404 0202 0404 0202 0103 0404 0104 0101 0202 0202 0101 0202 0303 0103 0202 0303 0202 0103 0404 0303 0404 0404 0202 0101 0103 0202 0101 0304 0303 0303 0303 0303 0404 0103 0101 0303 0104 0104 0103 0101 0303 0303 0204 0101 0404 0202 0101 0303 0202 0101 0303 0202 0202 0203 0303 0103 0103 0404 0303 0303 0101 0203 0101 0202 0404 0101 0303 0101 0404 0303 0101 0101 0404 0202 0404 0103 0204 0203 0101 0103 0202 0303 0102 0202 0203 0102 0101 0303 0101 0202 0203 0404 0202 0102 0101 0404 0101 0103 0202 0404 0303 0203 0303 0203 0101 0202 0304 0303 0404 0303 0303 0202 0202 0204 0303 0101 0404 0303 0204 0101 0404 0101 0103 0204 0000 0303 0101 0202 0204 0202 0404 0102 0303 0101 0101 0404 0103 0202 0303 0202 0404 0104 0101 0103 0104 0102 0303 0303 0404 0202 0303 0101 0103 0202 0101 0404 0303 0104 0204 0303 0202 0202 0203 0404 0303 0103 0101 0404 0304 0303 0202 0303 0101 0404 0303 0303 0101 0404 0404 0404 0303 0404 0304 0303 0102 0102 0303 0101 0101 0101 0202 0202 0303 0101 0404 0101 0103 0303 0204 0204 0102 0202 0101 0404 0202 0102 0404 0303 0202 0202 0202 0203 0204 0202 0101 0202 0101 0102 0404 0304 0303 0303 0202 0303 0404 0303 0404 0101 0103 0101 0202 0202 0101 0304 0202 0303 0303 0202 0101 0000 0303 0101 0404 0102 0404 0204 0000 0303 0203 0203 0102 0202 0303 0102 0103 0101 0303 0202 0303 0303 0303 0204 0303 0303 0202 0102 0404 0202 0204 0101 0404 0303 0404 0202 0404 0204 0202 0103 0202 0404 0303 0404 0303 0202 0303 0303 0202 0101 0303 0303 0202 0204 0404 0101 0303 0204 0303 0103 0404 0202 0303 0203 0303 0202 0203 0102 0101 0102 0404 0000 0303 0303 0202 0202 0104 0303 0104 0304 0204 0204 0102 0204 0103 0101 0202 0202 0101 0202 0404 0303 0204 0101 0404 0103 0303 0303 0101 0101 0101 0303 0404 0202 0101 0303 0202 0102 0202 0101 0204 0303 0101 0203 0202 0101 0202 0303 0000 0202 0303 0204 0204 0202 0303 0303 0202 0202 0101 0404 0303 0404 0303 0101 0202 0202 0202 0303 0404 0404 0404 0404 0204 0303 0202 0101 0303 0202 0202 0101 0101 0202 0303 0101 0204 0304 0202 0303 0404 0404 0404 0202 0103 0303 0303 0303 0404 0103 0204 0202 0404 0103 0204 0404 0202 0303 0303 0404 0404 0101 0303 0202 0204 0303 0101 0404 0101 0101 0202 0103 0303 0404 0303 0202 0404 0101 0101 0303 0202 0202 0202 0303 0202 0304 0103 0101 0102 0203 0202 0202 0101 0104 0303 0202 0204 0101 0303 0101 0404 0304 0104 0303 0404 0101 0303 0404 0304 0404 0204 0101 0202 0202 0404 0202 0104 0404 0303 0204 0303 0103 0202 0303 0404 0304 0404 0202 0202 0303 0202 0202 0204 0202 0204 0404 0404 0103 0304 0203 0303 0202 0101 0202 0404 0202 0303 0202 0204 0103 0202 0303 0303 0101 0103 0404 0202 0404 0303 0202 0303 0204 0101 0102 0404 0102 0202 0303 0204 0404 0202 0103 0303 0303 0203 0202 0303 0103 0202 0202 0102 0404 0303 0203 0404 0202 0101 0303 0303 0404 0204 0404 0202 0404 0101 0000 0303 0202 0303 0204 0101 0101 0202 0303 0202 0202 0303 0202 0202 0203 0101 0303 0303 0304 0202 0101 0103 0202 0202 0101 0303 0202 0202 0204 0104 0204 0202 0101 0202 0202 0101 0103 0303 0404 0202 0103 0101 0202 0202 0303 0103 0103 0202 0202 0101 0204 0404 0000 0404 0104 0101 0202 0204 0303 0102 0202 0202 0202 0202 0404 0101 0103 0404 0101 0303 0103 0303 0202 0101 0303 0204 0101 0202 0404 0303 0303 0202 0303 0202 0303 0204 0202 0101 0303 0303 0202 0404 0303 0303 0404 0303 0103 0204 0303 0101 0202 0303 0202 0303 0303 0203 0000 0404 0202 0104 0202 0204 0104 0202 0304 0303 0101 0204 0101 0101 0101 0101 0103 0204 0404 0101 0303 0404 0303 0101 0202 0303 0202 0202 0101 0104 0202 0101 0203 0303 0303 0303 0202 0204 0303 0303 0202 0204 0404 0101 0304 0404 0303 0101 0102 0404 0404 0103 0404 0404 0104 0101 0303 0304 0404 0101 0303 0404 0202 0202 0101 0101 0101 0202 0202 0202 0202 0303 0303 0404 0404 0202 0304 0101 0404 0101 0303 0303 0202 0303 0103 0304 0404 0202 0101 0102 0303 0303 0303 0202 0101 0102 0404 0202 0203 0404 0101 0303 0303 0101 0303 0202 0101 0304 0303 0303 0202 0202 0202 0303 0102 0202 0404 0203 0202 0103 0303 0104 0404 0303 0303 0404 0202 0303 0202 0203 0101 0204 0104 0204 0303 0202 0101 0202 0404 0101 0101 0303 0404 0101 0303 0203 0303 0404 0404 0103 0202 0202 0102 0204 0101 0202 0303 0202 0202 0303 0101 0101 0101 0303 0404 0204 0101 0103 0101 0304 0303 0303 0303 0304 0303 0303 0204 0404 0304 0404 0303 0202 0101 0303 0101 0101 0204 0404 0204 0202 0303 0303 0303 0202 0202 0404 0202 0202 0404 0103 0404 0202 0202 0202 0303 0303 0202 0101 0404 0303 0404 0404 0204 0404 0202 0303 0202 0103 0303 0202 0203 0204 0404 0303 0303 0101 0404 0303 0202 0202 0101 0303 0404 0204 0303 0101 0303 0303 0103 0303 0204 0202 0203 0303 0303 0102 0404 0303 0303 0203 0404 0101 0303 0404 0202 0204 0202 0202 0104 0202 0202 0202 0202 0101 0102 0104 0101 0103 0404 0202 0404 0303 0202 0103 0202 0203 0202 0303 0204 0303 0303 0304 0303 0303 0202 0303 0303 0303 0103 0202 0303 0303 0202 0101 0104 0202 0103 0303 0101 0202 0303 0202 0404 0303 0103 0202 0202 0303 0104 0303 0102 0202 0303 0303 0202 0202 0202 0202 0404 0101 0101 0202 0202 0304 0304 0202 0101 0202 0101 0303 0303 0101 0303 0404 0000 0303 0101 0101 0303 0303 0202 0202 0204 0303 0000 0102 0202 0202 0304 0303 0000 0303 0303 0202 0404 0404 0202 0202 0202 0303 0303 0103 0101 0202 0101 0101 0101 0103 0303 0202 0202 0101 0103 0204 0202 0101 0103 0202 0103 0202 0303 0202 0104 0303 0204 0101 0404 0202 0202 0202 0000 0303 0101 0101 0204 0202 0202 0303 0202 0202 0202 0303 0303 0203 0404 0202 0303 0202 0202 0202 0404 0101 0101 0103 0303 0202 0404 0303 0303 0202 0101 0204 0404 0202 0303 0101 0303 0404 0203 0101 0202 0101 0404 0404 0404 0101 0203 0101 0404 0202 0303 0202 0404 0101 0202 0101 0202 0202 0303 0404 0303 0101 0303 0303 0404 0202 0404 0103 0101 0103 0101 0202 0202 0204 0202 0203 0303 0404 0000 0103 0303 0303 0404 0202 0202 0404 0202 0203 0103 0104 0404 0303 0202 0203 0202 0404 0202 0303 0303 0404 0203 0204 0303 0204 0202 0203 0303 0102 0304 0101 0202 0404 0303 0202 0303 0202 0204 0103 0303 0104 0101 0404 0303 0303 0303 0404 0303 0202 0101 0104 0103 0202 0304 0404 0103 0203 0202 0304 0303 0404 0404 0202 0101 0204 0101 0303 0103 0204 0101 0404 0101 0303 0101 0204 0204 0303 0103 0303 0101 0303 0101 0104 0404 0304 0303 0101 0303 0103 0204 0101 0102 0203 0202 0104 0202 0303 0203 0303 0404 0303 0404 0404 0101 0202 0303 0202 0303 0404 0101 0304 0203 0202 0404 0303 0203 0303 0204 0202 0101 0204 0404 0102 0204 0202 0303 0303 0204 0404 0404 0204 0101 0202 0101 0202 0404 0103 0303 0404 0303 0202 0202 0202 0303 0404 0303 0404 0303 0303 0202 0202 0303 0303 0404 0404 0204 0202 0203 0202 0304 0303 0404 0303 0404 0101 0202 0303 0404 0303 0202 0202 0204 0101 0202 0202 0303 0204 0104 0101 0202 0101 0101 0303 0303 0202 0202 0202 0202 0204 0303 0304 0303 0202 0404 0000 0404 0202 0202 0202 0303 0101 0101 0104 0303 0202 0303 0101 0101 0101 0303 0101 0202 0404 0101 0304 0101 0404 0303 0404 0303 0303 0304 0303 0103 0303 0202 0101 0202 0202 0204 0202 0303 0404 0404 0103 0102 0404 0101 0303 0202 0404 0404 0204 0101 0103 0404 0202 0202 0404 0104 0404 0202 0404 0101 0404 0303 0204 0203 0103 0103 0404 0101 0303 0103 0101 0303 0101 0202 0202 0102 0101 0204 0102 0202 0202 0104 0204 0101 0303 0203 0202 0202 0203 0303 0303 0101 0404 0103 0102 0303 0202 0103 0202 0404 0303 0202 0101 0404 0303 0404 0103 0202 0303 0101 0303 0404 0202 0202 0202 0101 0202 0202 0202 0101 0303 0103 0101 0204 0103 0202 0202 0303 0104 0202 0303 0303 0404 0103 0101 0000 0102 0104 0204 0404 0202 0101 0202 0202 0202 0303 0202 0202 0404 0202 0303 0404 0303 0103 0202 0404 0104 0101 0204 0102 0103 0303 0404 0304 0404 0303 0303 0101 0303 0103 0202 0404 0303 0404 0202 0104 0104 0404 0104 0404 0303 0303 0202 0304 0404 0103 0101 0101 0202 0303 0404 0103 0101 0303 0101 0404 0404 0303 0102 0102 0202 0204 0202 0103 0101 0404 0303 0202 0104 0202 0303 0303 0303 0303 0303 0404 0103 0304 0104 0204 0101 0101 0303 0404 0202 0101 0102 0303 0404 0101 0203 0101 0303 0303 0303 0202 0202 0404 0102 0202 0303 0202 0203 0303 0404 0404 0104 0404 0303 0202 0303 0404 0303 0202 0404 0404 0404 0101 0202 0101 0303 0303 0103 0303 0202 0404 0103 0202 0203 0303 0103 0303 0203 0202 0303 0101 0303 0101 0101 0404 0202 0202 0303 0204 0202 0101 0303 0304 0203 0202 0101 0101 0103 0404 0103 0303 0303 0102 0303 0404 0202 0101 0103 0404 0202 0303 0101 0204 0404 0202 0103 0404 0303 0303 0404 0202 0202 0101 0202 0102 0104 0202 0202 0202 0303 0101 0202 0404 0404 0102 0204 0102 0101 0204 0103 0303 0202 0303 0202 0202 0404 0103 0404 0202 0103 0404 0103 0101 0404 0101 0101 0104 0204 0103 0102 0101 0303 0303 0203 0202 0103 0101 0404 0404 0303 0204 0404 0404 0202 0204 0404 0304 0202 0303 0102 0103 0303 0303 0104 0202 0103 0101 0101 0404 0303 0303 0404 0303 0202 0404 0103 0101 0202 0103 0303 0303 0101 0404 0303 0404 0202 0303 0103 0303 0101 0203 0101 0102 0202 0303 0404 0102 0103 0304 0101 0303 0404 0202 0303 0101 0303 0202 0102 0303 0303 0404 0404 0404 0204 0404 0101 0202 0404 0404 0101 0101 0303 0303 0303 0404 0202 0303 0202 0304 0203 0204 0202 0101 0202 0304 0202 0303 0303 0202 0404 0103 0202 0202 0204 0204 0202 0303 0303 0404 0202 0303 0404 0202 0101 0404 0103 0404 0101 0101 0102 0203 0202 0303 0202 0303 0202 0404 0202 0303 0101 0303 0202 0404 0101 0404 0303 0103 0204 0104 0101 0101 0404 0102 0102 0103 0202 0303 0303 0101 0103 0202 0202 0303 0203 0203 0103 0404 0204 0101 0303 0101 0101 0202 0101 0202 0102 0202 0101 0204 0102 0404 0304 0204 0303 0101 0404 0303 0303 0304 0101 0303 0404 0202 0404 0303 0202 0101 0404 0202 0202 0202 0202 0303 0303 0101 0103 0303 0103 0303 0103 0103 0303 0202 0404 0202 0404 0101 0303 0404 0101 0202 0203 0303 0101 0202 0303 0202 0202 0101 0202 0404 0303 0203 0101 0404 0202 0202 0103 0303 0303 0202 0204 0202 0303 0304 0202 0303 0103 0202 0404 0303 0202 0202 0303 0104 0102 0204 0204 0204 0101 0101 0304 0102 0102 0202 0303 0202 0404 0202 0204 0204 0202 0202 0103 0303 0404 0303 0202 0204 0103 0202 0202 0103 0303 0404 0303 0404 0303 0303 0204 0303 0101 0202 0202 0303 0204 0404 0204 0303 0101 0101 0101 0303 0303 0204 0104 0404 0101 0303 0303 0303 0101 0104 0202 0303 0101 0204 0102 0404 0202 0102 0202 0304 0204 0104 0101 0202 0203 0303 0202 0101 0303 0303 0202 0303 0103 0404 0404 0404 0202 0202 0101 0101 0404 0101 0303 0303 0101 0303 0103 0202 0104 0404 0101 0404 0303 0204 0101 0303 0101 0103 0404 0404 0202 0102 0101 0303 0404 0303 0101 0202 0202 0103 0303 0202 0101 0101 0404 0202 0303 0103 0404 0203 0303 0202 0101 0203 0202 0404 0101 0202 0102 0202 0202 0303 0404 0404 0303 0101 0103 0202 0202 0104 0102 0303 0204 0303 0202 0101 0202 0303 0101 0203 0404 0202 0303 0202 0202 0204 0404 0202 0101 0103 0103 0303 0303 0404 0203 0202 0204 0304 0101 0202 0303 0101 0203 0303 0101 0202 0202 0404 0303 0204 0404 0104 0202 0303 0202 0204 0101 0104 0404 0404 0303 0202 0202 0104 0103 0101 0303 0202 0101 0202 0202 0202 0102 0404 0303 0101 0202 0202 0203 0204 0404 0303 0303 0404 0000 0404 0102 0202 0303 0303 0303 0204 0202 0102 0404 0202 0303 0304 0202 0101 0101 0303 0303 0303 0102 0204 0202 0101 0101 0404 0101 0202 0202 0202 0404 0303 0303 0103 0404 0202 0202 0303 0404 0204 0202 0304 0204 0202 0404 0202 0203 0202 0202 0202 0303 0101 0404 0103 0202 0101 0404 0204 0202 0204 0101 0303 0101 0101 0202 0101 0304 0404 0101 0202 0103 0303 0204 0303 0304 0303 0101 0203 0202 0303 0303 0304 0202 0202 0303 0303 0404 0202 0303 0204 0204 0303 0202 0103 0303 0202 0103 0404 0204 0404 0101 0304 0101 0202 0404 0204 0101 0102 0303 0404 0101 0404 0404 0202 0204 0202 0404 0202 0404 0101 0202 0202 0303 0404 0204 0203 0404 0404 0202 0404 0303 0204 0404 0404 0202 0103 0404 0202 0303 0101 0303 0404 0303 0203 0101 0303 0202 0404 0101 0101 0202 0404 0202 0101 0404 0202 0202 0303 0202 0101 0202 0204 0303 0303 0102 0101 0404 0202 0303 0202 0202 0103 0102 0103 0103 0404 0202 0304 0103 0101 0202 0303 0404 0101 0202 0202 0303 0202 0202 0303 0303 0101 0101 0202 0202 0101 0203 0204 0303 0202 0404 0303 0203 0101 0404 0102 0101 0303 0202 0303 0404 0101 0202 0303 0404 0204 0304 0303 0101 0404 0303 0202 0101 0202 0101 0202 0404 0202 0404 0204 0103 0404 0404 0404 0101 0303 0203 0203 0303 0303 0404 0303 0304 0202 0404 0101 0303 0404 0202 0203 0303 0104 0404 0202 0303 0304 0202 0102 0404 0303 0303 0303 0202 0101 0404 0303 0204 0204 0101 0102 0204 0202 0203 0103 0303 0303 0202 0101 0304 0304 0303 0101 0202 0101 0404 0104 0103 0101 0103 0404 0101 0101 0202 0404 0202 0404 0303 0202 0303 0404 0103 0202 0303 0204 0101 0404 0404 0404 0303 0101 0202 0303 0203 0202 0202 0102 0404 0303 0404 0304 0202 0404 0303 0104 0404 0303 0202 0101 0303 0303 0202 0404 0202 0202 0304 0203 0404 0404 0103 0103 0202 0103 0104 0404 0101 0101 0203 0103 0303 0101 0404 0104 0101 0101 0204 0404 0202 0202 0203 0202 0304 0404 0303 0202 0202 0303 0202 0404 0304 0202 0204 0101 0303 0101 0404 0303 0404 0404 0103 0202 0404 0101 0303 0303 0103 0304 0303 0202 0101 0103 0202 0202 0303 0204 0303 0303 0303 0202 0101 0202 0202 0303 0103 0404 0202 0202 0202 0404 0101 0104 0404 0104 0204 0203 0404 0304 0202 0202 0202 0101 0101 0102 0101 0104 0101 0303 0404 0404 0101 0203 0204 0204 0101 0404 0103 0404 0101 0101 0101 0303 0404 0303 0304 0404 0101 0404 0304 0103 0103 0202 0101 0404 0204 0204 0202 0202 0202 0101 0303 0303 0202 0303 0304 0202 0202 0202 0303 0404 0101 0202 0101 0303 0404 0103 0103 0303 0101 0202 0103 0404 0202 0101 0303 0204 0404 0101 0101 0203 0202 0102 0104 0103 0404 0303 0404 0404 0202 0204 0404 0202 0202 0103 0202 0303 0204 0102 0202 0202 0303 0104 0103 0404 0202 0204 0303 0303 0202 0202 0202 0203 0303 0102 0303 0404 0203 0202 0404 0404 0303 0404 0204 0202 0202 0104 0103 0202 0101 0101 0202 0303 0102 0103 0101 0303 0202 0204 0202 0202 0101 0101 0202 0204 0204 0101 0303 0202 0202 0202 0101 0303 0404 0202 0101 0303 0404 0101 0202 0204 0204 0104 0101 0101 0202 0404 0101 0202 0204 0303 0202 0303 0202 0303 0203 0204 0204 0304 0303 0202 0101 0202 0104 0101 0103 0303 0404 0204 0104 0203 0202 0304 0101 0204 0202 0303 0101 0101 0000 0103 0303 0303 0404 0202 0303 0303 0202 0202 0202 0404 0202 0303 0202 0104 0202 0202 0303 0204 0202 0101 0404 0103 0103 0404 0404 0103 0101 0304 0101 0303 0202 0204 0303 0303 0204 0202 0202 0104 0104 0404 0101 0202 0202 0204 0404 0204 0101 0104 0103 0204 0303 0103 0204 0202 0202 0103 0104 0103 0303 0303 0101 0202 0202 0101 0202 0103 0202 0103 0103 0203 0404 0303 0103 0303 0404 0103 0101 0404 0101 0103 0103 0202 0404 0103 0101 0202 0404 0101 0104 0204 0104 0303 0101 0101 0101 0202 0202 0101 0202 0202 0404 0303 0202 0303 0202 0101 0202 0404 0202 0102 0204 0202 0202 0204 0404 0404 0202 0202 0202 0204 0404 0404 0404 0303 0204 0202 0204 0202 0303 0101 0104 0404 0303 0102 0102 0203 0104 0203 0103 0202 0404 0303 0101 0303 0101 0303 0101 0102 0204 0202 0404 0204 0101 0202 0204 0303 0101 0202 0202 0404 0202 0204 0204 0104 0202 0303 0103 0102 0102 0404 0202 0303 0303 0303 0101 0404 0202 0104 0204 0304 0202 0202 0304 0104 0203 0101 0202 0101 0102 0303 0204 0304 0103 0102 0101 0204 0303 0102 0202 0303 0202 0202 0202 0202 0404 0202 0303 0103 0202 0202 0404 0404 0104 0202 0101 0303 0303 0404 0202 0202 0204 0101 0404 0202 0304 0404 0202 0204 0303 0101 0202 0404 0303 0404 0304 0404 0303 0202 0202 0404 0104 0101 0103 0303 0204 0103 0303 0202 0101 0202 0101 0000 0103 0204 0404 0404 0101 0304 0101 0303 0404 0103 0202 0104 0202 0101 0103 0101 0404 0404 0104 0102 0202 0404 0103 0404 0202 0404 0203 0202 0404 0303 0204 0204 0104 0404 0404 0102 0202 0202 0404 0204 0204 0404 0202 0203 0303 0304 0303 0303 0303 0202 0101 0303 0202 0404 0102 0202 0303 0304 0404 0202 0404 0103 0303 0202 0204 0303 0202 0303 0103 0101 0303 0303 0202 0303 0204 0102 0404 0303 0304 0103 0303 0101 0202 0303 0303 0101 0404 0303 0101 0104 0204 0202 0202 0202 0000 0202 0303 0204 0101 0103 0000 0202 0202 0303 0303 0202 0202 0204 0101 0104 0404 0101 0404 0101 0303 0303 0404 0102 0101 0202 0101 0204 0101 0404 0404 0101 0202 0202 0303 0202 0202 0202 0303 0204 0303 0204 0101 0303 0101 0303 0304 0101 0303 0103 0404 0202 0404 0303 0303 0103 0101 0404 0404 0404 0202 0202 0204 0104 0202 0204 0404 0202 0101 0303 0303 0304 0303 0404 0404 0303 0202 0303 0202 0303 0303 0304 0303 0202 0303 0202 0101 0103 0303 0204 0102 0101 0202 0202 0303 0303 0304 0303 0303 0103 0204 0303 0101 0404 0101 0101 0202 0101 0202 0304 0101 0303 0101 0103 0102 0101 0204 0104 0202 0104 0202 0101 0303 0404 0101 0404 0104 0202 0103 0303 0202 0303 0303 0303 0202 0103 0404 0101 0303 0404 0203 0204 0103 0303 0204 0202 0204 0101 0202 0204 0101 0102 0104 0101 0204 0303 0202 0101 0204 0303 0203 0104 0103 0303 0202 0303 0303 0303 0202 0202 0103 0303 0303 0202 0101 0303 0303 0101 0202 0203 0102 0303 0404 0103 0103 0303 0303 0101 0101 0404 0303 0101 0202 0404 0202 0202 0303 0202 0101 0404 0404 0101 0303 0303 0204 0202 0202 0303 0303 0303 0101 0204 0204 0102 0101 0404 0303 0202 0103 0404 0202 0303 0303 0303 0101 0102 0103 0202 0202 0204 0303 0202 0304 0101 0103 0101 0202 0404 0303 0204 0204 0104 0101 0404 0103 0202 0101 0203 0202 0303 0103 0303 0303 0404 0101 0204 0203 0303 0103 0303 0303 0303 0101 0101 0303 0102 0202 0203 0101 0101 0204 0202 0303 0303 0404 0101 0101 0202 0204 0202 0204 0303 0202 0101 0303 0404 0303 0103 0204 0202 0404 0202 0303 0303 0303 0103 0202 0103 0404 0204 0404 0202 0202 0202 0303 0202 0404 0404 0304 0202 0303 0101 0202 0303 0303 0202 0303 0204 0303 0202 0404 0303 0404 0101 0303 0303 0203 0204 0103 0103 0103 0303 0404 0303 0202 0202 0404 0204 0101 0202 0404 0101 0404 0104 0202 0102 0204 0101 0204 0303 0202 0404 0202 0202 0203 0303 0304 0304 0102 0103 0204 0101 0102 0303 0103 0303 0304 0101 0404 0202 0404 0404 0303 0303 0204 0203 0202 0103 0104 0101 0202 0303 0101 0404 0404 0101 0303 0304 0204 0404 0204 0202 0203 0103 0303 0101 0203 0404 0303 0404 0303 0404 0202 0104 0202 0103 0404 0203 0404 0303 0101 0202 0202 0204 0202 0102 0202 0202 0303 0303 0101 0404 0101 0303 0303 0101 0303 0101 0303 0103 0404 0101 0000 0202 0202 0202 0202 0303 0404 0102 0204 0202 0204 0303 0103 0204 0103 0202 0404 0404 0404 0202 0404 0404 0203 0204 0202 0303 0202 0304 0202 0303 0303 0103 0104 0303 0204 0202 0204 0202 0202 0202 0303 0303 0203 0303 0303 0202 0303 0303 0101 0202 0101 0303 0303 0303 0303 0404 0202 0000 0202 0202 0204 0404 0101 0104 0202 0101 0404 0303 0303 0303 0303 0304 0103 0404 0303 0303 0101 0103 0404 0303 0404 0202 0104 0202 0303 0202 0204 0303 0202 0202 0202 0203 0404 0303 0303 0202 0202 0103 0303 0202 0303 0202 0303 0304 0204 0202 0103 0204 0204 0303 0303 0404 0204 0101 0303 0404 0101 0404 0304 0202 0202 0303 0404 0101 0303 0104 0102 0101 0303 0303 0404 0202 0204 0404 0404 0303 0303 0101 0303 0303 0303 0101 0203 0303 0303 0404 0203 0202 0303 0202 0204 0202 0303 0202 0101 0202 0404 0303 0101 0303 0202 0203 0304 0404 0101 0202 0202 0303 0103 0303 0202 0202 0404 0303 0303 0101 0303 0202 0303 0404 0202 0202 0303 0204 0303 0101 0303 0303 0303 0404 0103 0202 0303 0103 0303 0202 0103 0303 0204 0202 0404 0104 0303 0303 0203 0103 0202 0202 0304 0202 0104 0204 0303 0404 0101 0303 0202 0101 0303 0303 0202 0303 0204 0104 0204 0101 0404 0101 0202 0404 0303 0101 0303 0202 0404 0404 0204 0204 0202 0104 0202 0404 0303 0202 0202 0202 0101 0102 0404 0103 0303 0303 0404 0202 0202 0404 0101 0303 0202 0202 0202 0101 0404 0202 0304 0404 0202 0102 0303 0202 0101 0202 0202 0404 0101 0102 0103 0204 0404 0101 0101 0303 0404 0404 0202 0102 0202 0303 0202 0202 0404 0303 0303 0103 0303 0202 0303 0304 0303 0404 0303 0303 0404 0202 0303 0202 0202 0303 0202 0303 0303 0404 0101 0204 0303 0202 0202 0202 0102 0101 0102 0204 0303 0303 0202 0101 0404 0303 0202 0404 0204 0303 0101 0404 0204 0103 0202 0303 0202 0404 0404 0404 0103 0303 0104 0404 0202 0303 0102 0303 0103 0303 0303 0404 0303 0303 0404 0404 0303 0202 0101 0404 0101 0101 0103 0101 0202 0101 0101 0303 0202 0202 0303 0202 0204 0101 0101 0101 0101 0404 0203 0204 0202 0303 0303 0404 0202 0404 0101 0303 0202 0102 0303 0202 0204 0204 0404 0104 0202 0202 0101 0304 0304 0404 0202 0102 0101 0303 0404 0303 0404 0203 0303 0303 0101 0404 0102 0103 0404 0202 0303 0303 0404 0103 0204 0202 0103 0104 0202 0202 0104 0404 0202 0202 0202 0101 0101 0303 0303 0303 0303 0101 0404 0303 0303 0202 0202 0404 0303 0404 0404 0303 0202 0204 0101 0404 0202 0303 0303 0101 0101 0303 0303 0000 0404 0303 0303 0202 0404 0204 0202 0102 0202 0101 0101 0303 0304 0204 0203 0404 0303 0303 0101 0404 0303 0103 0303 0103 0404 0204 0304 0202 0101 0203 0101 0304 0304 0404 0303 0303 0203 0202 0103 0203 0204 0303 0404 0101 0303 0101 0103 0202 0202 0101 0202 0202 0202 0304 0104 0304 0203 0102 0204 0101 0202 0204 0303 0202 0103 0202 0202 0102 0202 0404 0101 0202 0202 0303 0404 0202 0103 0103 0303 0303 0303 0404 0303 0101 0202 0303 0204 0103 0202 0404 0104 0202 0303 0203 0204 0101 0204 0404 0104 0404 0404 0203 0404 0101 0103 0303 0303 0104 0303 0303 0404 0202 0404 0101 0303 0102 0404 0204 0204 0303 0103 0101 0303 0104 0202 0202 0204 0303 0303 0303 0303 0202 0101 0202 0104 0303 0303 0202 0303 0303 0303 0104 0102 0101 0101 0101 0404 0204 0404 0103 0101 0101 0202 0404 0204 0202 0103 0202 0303 0303 0202 0202 0101 0204 0404 0202 0202 0303 0303 0303 0404 0102 0204 0303 0304 0101 0404 0303 0103 0203 0202 0101 0103 0404 0303 0102 0202 0202 0202 0404 0101 0303 0303 0303 0404 0404 0204 0202 0202 0404 0304 0101 0101 0404 0304 0202 0202 0404 0101 0101 0303 0303 0303 0101 0404 0202 0101 0204 0101 0101 0103 0101 0203 0104 0202 0101 0102 0202 0303 0101 0303 0101 0103 0304 0404 0204 0202 0303 0404 0303 0202 0101 0204 0103 0102 0202 0102 0101 0404 0304 0101 0404 0303 0102 0202 0202 0103 0104 0303 0102 0202 0102 0404 0102 0202 0303 0101 0404 0101 0404 0202 0101 0101 0303 0303 0102 0303 0303 0202 0303 0203 0202 0303 0303 0202 0303 0303 0303 0303 0204 0202 0303 0102 0204 0304 0303 0202 0202 0204 0303 0304 0202 0202 0101 0303 0102 0202 0303 0202 0202 0202 0101 0202 0303 0202 0103 0303 0202 0303 0404 0303 0101 0404 0404 0101 0303 0304 0202 0101 0303 0102 0204 0101 0103 0204 0101 0202 0304 0101 0303 0202 0102 0303 0104 0404 0202 0101 0202 0404 0303 0404 0303 0204 0101 0303 0101 0404 0404 0000 0204 0304 0303 0102 0304 0101 0204 0303 0101 0303 0303 0303 0101 0404 0104 0303 0404 0202 0202 0103 0303 0101 0101 0202 0202 0101 0404 0204 0103 0404 0101 0202 0404 0101 0101 0202 0101 0101 0404 0303 0103 0303 0202 0404 0202 0202 0303 0101 0303 0303 0303 0202 0304 0103 0101 0101 0202 0303 0203 0202 0101 0202 0202 0203 0202 0202 0202 0202 0303 0103 0202 0303 0101 0404 0404 0102 0202 0204 0101 0303 0202 0303 0103 0102 0404 0303 0202 0303 0202 0101 0303 0404 0404 0303 0202 0404 0202 0102 0202 0404 0303 0103 0104 0202 0204 0101 0303 0101 0204 0204 0303 0404 0202 0204 0404 0303 0202 0102 0103 0101 0404 0303 0202 0303 0202 0202 0103 0303 0303 0101 0204 0202 0404 0202 0202 0303 0104 0404 0404 0101 0404 0204 0101 0202 0304 0404 0203 0303 0104 0202 0104 0103 0202 0303 0204 0404 0304 0101 0102 0202 0303 0303 0101 0101 0202 0202 0103 0103 0404 0103 0202 0102 0101 0101 0102 0202 0202 0101 0103 0204 0101 0303 0303 0404 0303 0101 0101 0404 0304 0104 0303 0304 0202 0101 0303 0202 0202 0204 0102 0404 0404 0101 0202 0204 0202 0204 0204 0103 0204 0404 0204 0202 0303 0204 0404 0204 0404 0303 0303 0103 0101 0404 0203 0103 0303 0104 0404 0202 0404 0202 0202 0101 0202 0204 0303 0101 0101 0303 0303 0202 0304 0101 0101 0202 0304 0202 0202 0104 0404 0303 0404 0303 0202 0404 0303 0304 0102 0404 0404 0202 0202 0303 0101 0104 0000 0404 0404 0404 0404 0303 0303 0303 0303 0202 0202 0202 0202 0103 0103 0404 0000 0204 0404 0202 0303 0202 0202 0202 0304 0101 0203 0000 0103 0204 0202 0204 0303 0101 0202 0303 0404 0101 0101 0202 0303 0202 0202 0304 0202 0404 0202 0404 0101 0303 0303 0303 0104 0202 0102 0404 0202 0104 0303 0404 0101 0104 0404 0303 0303 0404 0404 0303 0204 0203 0204 0304 0202 0204 0404 0404 0303 0203 0104 0303 0104 0404 0101 0202 0404 0202 0202 0404 0202 0101 0303 0204 0101 0303 0202 0103 0404 0000 0303 0203 0102 0303 0404 0303 0202 0304 0202 0303 0000 0404 0102 0104 0103 0404 0103 0101 0104 0101 0101 0101 0202 0204 0101 0103 0303 0303 0202 0103 0101 0202 0404 0102 0404 0204 0101 0404 0101 0101 0202 0101 0101 0202 0101 0103 0103 0303 0101 0203 0202 0404 0303 0404 0202 0303 0202 0202 0202 0404 0101 0303 0103 0101 0303 0202 0104 0101 0102 0303 0101 0204 0202 0101 0303 0404 0202 0103 0103 0202 0404 0103 0103 0303 0202 0303 0202 0101 0202 0202 0103 0101 0303 0204 0202 0303 0101 0203 0404 0204 0204 0303 0303 0204 0404 0101 0404 0303 0202 0103 0303 0202 0101 0404 0202 0000 0404 0203 0101 0202 0102 0101 0202 0101 0103 0303 0404 0404 0303 0202 0303 0101 0103 0404 0202 0101 0202 0303 0404 0103 0204 0304 0101 0303 0102 0103 0204 0203 0104 0101 0104 0202 0204 0304 0303 0101 0102 0303 0101 0000 0103 0202 0202 0303 0103 0103 0103 0102 0303 0101 0104 0202 0404 0101 0404 0103 0404 0102 0202 0404 0202 0103 0101 0303 0404 0202 0101 0202 0101 0102 0204 0303 0102 0101 0104 0101 0303 0303 0104 0102 0304 0101 0303 0202 0203 0101 0202 0404 0404 0101 0303 0303 0204 0202 0103 0303 0202 0202 0202 0404 0404 0104 0404 0303 0102 0204 0202 0202 0303 0303 0303 0101 0101 0101 0101 0101 0404 0204 0202 0304 0303 0102 0303 0000 0404 0203 0303 0303 0101 0303 0404 0103 0203 0202 0404 0202 0404 0202 0101 0404 0404 0202 0202 0101 0202 0304 0102 0101 0202 0404 0202 0204 0103 0303 0101 0202 0303 0404 0101 0103 0404 0304 0303 0404 0303 0202 0303 0103 0303 0303 0202 0202 0103 0202 0101 0303 0303 0104 0202 0101 0101 0204 0404 0202 0101 0101 0303 0202 0101 0303 0202 0202 0303 0101 0202 0202 0204 0303 0303 0202 0303 0202 0102 0202 0204 0404 0101 0202 0101 0202 0101 0202 0202 0101 0204 0204 0103 0202 0303 0101 0202 0303 0404 0303 0404 0404 0404 0202 0101 0303 0204 0303 0103 0103 0303 0204 0404 0202 0202 0204 0202 0102 0202 0101 0404 0404 0202 0203 0202 0303 0202 0404 0202 0202 0204 0103 0404 0204 0404 0303 0404 0303 0202 0101 0404 0102 0404 0202 0303 0404 0202 0303 0101 0404 0101 0404 0303 0101 0102 0204 0104 0404 0404 0404 0404 0303 0303 0203 0101 0204 0101 0101 0404 0102 0404 0303 0303 0202 0102 0204 0303 0101 0202 0202 0202 0103 0202 0303 0303 0103 0202 0303 0303 0202 0104 0303 0204 0204 0202 0202 0101 0103 0404 0204 0303 0101 0204 0102 0203 0404 0202 0404 0303 0202 0101 0304 0101 0202 0303 0404 0204 0404 0103 0304 0304 0202 0303 0101 0303 0101 0203 0103 0404 0303 0202 0202 0104 0202 0303 0204 0101 0404 0404 0202 0303 0104 0101 0000 0101 0303 0303 0404 0102 0101 0303 0202 0202 0404 0102 0102 0101 0101 0404 0303 0101 0101 0202 0303 0404 0204 0303 0101 0404 0203 0101 0303 0103 0404 0103 0203 0303 0202 0204 0103 0303 0303 0202 0202 0404 0101 0303 0404 0103 0202 0104 0204 0303 0404 0104 0101 0303 0303 0202 0404 0204 0202 0202 0101 0303 0202 0202 0202 0404 0303 0303 0103 0303 0303 0303 0101 0303 0404 0404 0202 0303 0202 0204 0202 0303 0204 0202 0304 0202 0101 0203 0101 0202 0202 0404 0303 0303 0101 0202 0303 0304 0404 0303 0202 0103 0404 0404 0202 0303 0303 0203 0102 0202 0202 0204 0101 0103 0103 0303 0303 0101 0404 0303 0202 0102 0104 0202 0000 0103 0202 0101 0404 0101 0204 0101 0202 0101 0202 0202 0303 0202 0303 0103 0103 0104 0303 0303 0304 0101 0101 0303 0404 0202 0102 0202 0101 0202 0303 0101 0404 0204 0101 0303 0303 0303 0101 0202 0104 0303 0202 0101 0404 0303 0303 0202 0103 0303 0404 0303 0303 0303 0101 0404 0101 0202 0404 0103 0202 0202 0101 0202 0204 0202 0202 0103 0101 0203 0202 0202 0303 0101 0101 0404 0404 0202 0404 0103 0202 0202 0202 0202 0101 0103 0303 0303 0303 0404 0202 0404 0101 0103 0202 0304 0103 0404 0101 0303 0303 0303 0203 0202 0404 0103 0303 0101 0101 0202 0204 0202 0303 0202 0204 0103 0202 0404 0303 0000 0101 0102 0202 0303 0101 0404 0303 0202 0404 0202 0303 0303 0303 0103 0303 0103 0204 0101 0202 0303 0101 0303 0404 0202 0202 0103 0303 0303 0404 0102 0202 0303 0303 0303 0202 0204 0303 0103 0202 0202 0202 0202 0101 0303 0101 0303 0101 0202 0103 0103 0203 0303 0203 0202 0404 0202 0404 0404 0202 0404 0303 0404 0303 0204 0104 0202 0103 0203 0202 0304 0404 0303 0104 0103 0104 0404 0103 0101 0303 0202 0303 0101 0103 0102 0102 0202 0204 0102 0103 0303 0202 0404 0303 0101 0101 0303 0303 0303 0303 0303 0303 0101 0303 0203 0101 0103 0202 0203 0202 0404 0101 0204 0103 0303 0303 0101 0304 0103 0304 0104 0104 0103 0303 0304 0202 0404 0404 0202 0303 0303 0101 0202 0303 0204 0202 0303 0101 0202 0303 0303 0101 0102 0404 0202 0303 0101 0101 0203 0104 0204 0303 0404 0404 0102 0303 0303 0202 0202 0101 0103 0202 0202 0404 0303 0303 0404 0202 0101 0303 0103 0202 0404 0101 0104 0102 0203 0104 0202 0203 0202 0404 0104 0404 0101 0204 0304 0102 0103 0303 0303 0202 0203 0203 0202 0303 0202 0303 0404 0303 0303 0103 0202 0101 0101 0202 0303 0202 0103 0101 0202 0101 0204 0102 0202 0204 0204 0303 0404 0000 0404 0202 0404 0404 0103 0202 0204 0202 0102 0103 0202 0103 0104 0202 0404 0202 0101 0304 0101 0304 0103 0303 0104 0104 0303 0202 0404 0404 0202 0101 0104 0202 0101 0202 0202 0303 0103 0404 0204 0404 0303 0404 0303 0204 0303 0304 0101 0303 0202 0101 0304 0404 0101 0202 0303 0404 0303 0303 0202 0202 0204 0404 0303 0103 0102 0303 0303 0303 0404 0202 0202 0202 0203 0404 0101 0202 0103 0203 0203 0000 0303 0404 0404 0202 0303 0101 0404 0404 0303 0204 0101 0101 0202 0404 0101 0303 0204 0303 0202 0202 0303 0202 0202 0303 0202 0303 0303 0101 0202 0103 0101 0104 0404 0204 0202 0404 0203 0202 0103 0104 0202 0404 0404 0404 0101 0404 0103 0404 0104 0101 0303 0202 0202 0101 0101 0101 0202 0101 0404 0204 0202 0103 0101 0204 0303 0404 0101 0303 0303 0204 0101 0303 0202 0404 0202 0404 0202 0204 0202 0101 0204 0202 0103 0202 0404 0203 0303 0101 0101 0404 0103 0404 0303 0103 0202 0202 0203 0202 0101 0303 0404 0303 0404 0202 0303 0303 0404 0102 0203 0203 0203 0202 0304 0404 0202 0303 0303 0303 0104 0103 0303 0404 0202 0304 0404 0104 0303 0404 0404 0303 0202 0101 0101 0101 0303 0202 0303 0202 0303 0303 0104 0404 0304 0104 0404 0103 0202 0404 0404 0304 0101 0404 0203 0103 0202 0204 0202 0303 0202 0101 0303 0303 0104 0202 0101 0101 0202 0303 0303 0202 0101 0103 0202 0304 0202 0202 0103 0202 0202 0202 0204 0104 0404 0202 0303 0103 0304 0103 0404 0101 0404 0404 0202 0303 0202 0101 0404 0202 0202 0204 0204 0202 0202 0101 0104 0202 0202 0101 0204 0404 0404 0203 0202 0303 0304 0202 0202 0101 0202 0101 0404 0303 0303 0104 0202 0404 0303 0202 0101 0404 0101 0303 0404 0404 0101 0101 0101 0103 0101 0202 0304 0204 0101 0203 0202 0303 0202 0304 0303 0204 0404 0202 0202 0202 0101 0404 0202 0203 0202 0202 0202 0202 0204 0202 0404 0304 0404 0404 0202 0404 0103 0101 0303 0204 0202 0303 0404 0303 0204 0202 0202 0202 0202 0303 0303 0404 0303 0101 0101 0101 0303 0101 0101 0204 0101 0202 0204 0101 0101 0202 0101 0303 0202 0303 0202 0404 0303 0202 0404 0202 0202 0202 0404 0101 0101 0204 0303 0404 0202 0103 0404 0303 0104 0303 0103 0303 0204 0404 0103 0404 0404 0103 0202 0202 0101 0104 0204 0404 0303 0303 0202 0101 0204 0202 0202 0303 0101 0404 0303 0303 0103 0202 0404 0404 0404 0202 0404 0101 0101 0103 0202 0202 0202 0303 0103 0103 0101 0303 0404 0103 0202 0303 0103 0101 0404 0202 0303 0404 0101 0303 0303 0303 0204 0303 0404 0101 0202 0404 0202 0404 0101 0101 0304 0303 0202 0303 0303 0202 0303 0202 0103 0204 0404 0404 0202 0303 0202 0203 0104 0204 0101 0303 0202 0404 0202 0404 0202 0404 0303 0404 0103 0202 0202 0404 0303 0103 0204 0303 0104 0000 0303 0404 0202 0102 0202 0303 0303 0101 0303 0202 0202 0204 0101 0103 0204 0404 0303 0103 0202 0303 0202 0102 0304 0404 0404 0203 0202 0101 0303 0303 0202 0303 0303 0202 0304 0202 0404 0101 0103 0202 0101 0202 0204 0404 0303 0000 0101 0303 0304 0303 0104 0202 0202 0304 0303 0202 0303 0202 0404 0202 0202 0204 0101 0202 0202 0202 0303 0303 0303 0303 0101 0303 0303 0303 0303 0404 0404 0303 0303 0104 0101 0204 0202 0202 0202 0204 0204 0202 0202 0404 0304 0000 0204 0204 0404 0103 0103 0101 0103 0303 0404 0101 0202 0303 0404 0202 0204 0101 0101 0101 0303 0303 0303 0304 0101 0102 0303 0104 0203 0103 0202 0404 0104 0202 0101 0303 0101 0103 0404 0303 0404 0101 0303 0404 0103 0303 0202 0404 0204 0202 0303 0404 0203 0101 0101 0303 0103 0202 0101 0103 0303 0303 0204 0303 0303 0101 0404 0404 0204 0303 0303 0303 0303 0202 0203 0202 0303 0303 0204 0101 0101 0303 0303 0202 0303 0303 0202 0202 0303 0202 0202 0204 0101 0103 0103 0204 0202 0202 0303 0101 0104 0303 0303 0204 0103 0202 0102 0202 0303 0102 0202 0103 0104 0101 0103 0104 0202 0202 0104 0204 0303 0303 0303 0202 0203 0303 0303 0202 0404 0404 0101 0404 0404 0303 0204 0303 0202 0202 0303 0303 0203 0103 0303 0303 0204 0202 0202 0202 0202 0404 0101 0204 0202 0303 0404 0202 0404 0303 0303 0204 0404 0303 0103 0202 0303 0202 0203 0304 0103 0404 0101 0404 0103 0202 0404 0101 0202 0202 0101 0204 0303 0101 0303 0202 0303 0404 0303 0303 0104 0303 0101 0404 0303 0104 0101 0404 0103 0303 0101 0404 0303 0202 0103 0303 0203 0404 0202 0404 0202 0204 0202 0101 0303 0303 0102 0404 0101 0102 0101 0404 0202 0303 0303 0304 0303 0303 0404 0303 0202 0202 0204 0303 0103 0404 0101 0103 0101 0103 0303 0101 0101 0202 0404 0303 0204 0202 0101 0202 0303 0303 0101 0303 0304 0202 0303 0404 0202 0303 0202 0202 0202 0101 0202 0103 0202 0102 0204 0202 0303 0102 0103 0404 0101 0202 0303 0404 0404 0102 0103 0202 0304 0404 0303 0404 0202 0202 0103 0101 0303 0101 0202 0303 0202 0104 0202 0304 0202 0303 0103 0102 0404 0101 0101 0202 0203 0204 0404 0202 0404 0204 0304 0303 0304 0303 0102 0303 0404 0303 0202 0203 0101 0203 0101 0203 0204 0102 0103 0303 0202 0102 0404 0103 0303 0101 0303 0303 0303 0404 0404 0202 0202 0202 0202 0202 0101 0202 0101 0202 0202 0202 0304 0303 0404 0404 0202 0404 0303 0202 0303 0203 0404 0303 0202 0404 0101 0404 0202 0202 0202 0303 0204 0303 0204 0103 0101 0103 0303 0101 0202 0404 0202 0202 0202 0404 0101 0404 0101 0404 0101 0404 0303 0202 0404 0101 0202 0303 0103 0202 0303 0404 0103 0202 0101 0404 0101 0202 0101 0202 0304 0103 0404 0404 0404 0101 0404 0103 0303 0202 0204 0404 0404 0101 0404 0101 0404 0303 0202 0303 0303 0101 0204 0202 0404 0304 0303 0204 0303 0102 0101 0404 0101 0404 0404 0103 0303 0103 0303 0303 0202 0103 0101 0202 0101 0204 0404 0101 0404 0203 0101 0303 0202 0101 0202 0101 0204 0303 0303 0202 0404 0203 0404 0303 0404 0202 0202 0101 0404 0404 0101 0404 0303 0303 0404 0101 0101 0202 0102 0202 0101 0101 0204 0304 0303 0404 0202 0202 0204 0404 0103 0202 0101 0101 0103 0104 0103 0202 0104 0101 0202 0202 0303 0204 0303 0202 0103 0404 0404 0303 0103 0103 0202 0202 0303 0204 0202 0101 0304 0103 0404 0303 0101 0404 0204 0103 0202 0202 0204 0404 0202 0202 0103 0404 0303 0202 0202 0202 0303 0101 0304 0103 0404 0303 0304 0104 0101 0101 0204 0204 0104 0202 0303 0404 0202 0303 0202 0202 0102 0304 0103 0303 0404 0101 0101 0202 0102 0101 0303 0101 0404 0303 0303 0203 0303 0202 0202 0303 0101 0404 0204 0204 0404 0101 0202 0303 0104 0203 0204 0103 0101 0202 0104 0304 0304 0202 0303 0202 0204 0202 0404 0303 0204 0404 0101 0303 0204 0102 0101 0404 0202 0303 0202 0102 0303 0404 0304 0102 0202 0304 0202 0202 0203 0304 0303 0103 0203 0303 0202 0102 0303 0303 0304 0303 0303 0103 0304 0101 0202 0101 0101 0101 0204 0404 0202 0303 0101 0104 0103 0104 0204 0202 0304 0104 0303 0101 0104 0404 0202 0202 0204 0204 0204 0103 0404 0303 0303 0101

FB-X-007, 0303 0202 0202 0202 0303 0204 0101 0404 0404 0202 0204 0103 0104 0103 0304 0404 0102 0204 0104 0404 0404 0303 0202 0204 0102 0303 0303 0101 0303 0204 0304 0303 0203 0202 0202 0202 0101 0202 0204 0101 0202 0303 0404 0404 0204 0204 0202 0202 0101 0202 0303 0202 0103 0103 0303 0404 0303 0303 0303 0404 0202 0203 0204 0202 0101 0202 0303 0202 0101 0204 0202 0404 0101 0404 0104 0103 0304 0101 0303 0202 0404 0204 0303 0303 0404 0103 0303 0202 0404 0303 0101 0204 0202 0202 0103 0101 0103 0303 0202 0202 0101 0204 0204 0202 0303 0103 0303 0101 0303 0303 0102 0404 0303 0203 0202 0404 0101 0202 0404 0101 0101 0303 0101 0404 0303 0203 0303 0404 0303 0101 0202 0204 0202 0303 0202 0404 0404 0202 0202 0202 0404 0101 0202 0303 0102 0303 0303 0101 0303 0102 0101 0404 0304 0202 0303 0303 0000 0303 0304 0204 0202 0103 0101 0204 0103 0203 0101 0204 0101 0103 0204 0101 0204 0303 0404 0101 0303 0103 0202 0303 0304 0303 0104 0103 0303 0202 0104 0303 0303 0202 0202 0202 0304 0303 0202 0303 0101 0202 0101 0103 0304 0404 0202 0202 0204 0202 0202 0202 0101 0101 0103 0202 0303 0204 0404 0202 0203 0101 0303 0103 0303 0104 0204 0202 0304 0404 0303 0203 0101 0102 0303 0104 0404 0204 0304 0103 0101 0303 0104 0404 0303 0102 0103 0303 0103 0204 0101 0404 0103 0102 0202 0204 0101 0101 0103 0404 0303 0101 0404 0404 0101 0102 0303 0204 0204 0103 0303 0204 0202 0104 0202 0303 0204 0104 0101 0104 0202 0303 0202 0404 0101 0303 0101 0404 0404 0204 0304 0202 0404 0303 0204 0204 0202 0303 0101 0202 0103 0101 0404 0101 0303 0204 0202 0203 0202 0304 0202 0303 0202 0303 0303 0202 0202 0202 0203 0304 0404 0202 0404 0202 0303 0101 0303 0404 0202 0204 0303 0204 0303 0304 0404 0202 0404 0404 0304 0202 0204 0103 0103 0203 0202 0303 0303 0204 0202 0103 0203 0101 0404 0303 0303 0202 0404 0404 0202 0404 0102 0102 0202 0101 0101 0203 0404 0202 0404 0202 0303 0304 0203 0202 0104 0202 0404 0303 0202 0102 0303 0303 0203 0303 0404 0202 0303 0303 0304 0202 0204 0303 0101 0202 0202 0204 0101 0303 0202 0101 0303 0303 0103 0104 0404 0303 0303 0303 0303 0303 0204 0303 0303 0202 0103 0104 0202 0101 0101 0101 0103 0303 0103 0104 0303 0101 0404 0303 0303 0303 0303 0101 0103 0103 0303 0202 0101 0000 0104 0204 0404 0101 0101 0204 0202 0204 0404 0204 0303 0203 0303 0204 0103 0404 0303 0202 0202 0304 0404 0303 0202 0202 0204 0202 0303 0202 0404 0204 0103 0404 0202 0404 0103 0304 0303 0203 0304 0404 0101 0304 0303 0104 0102 0204 0303 0304 0101 0103 0203 0101 0204 0202 0103 0202 0303 0101 0303 0104 0204 0303 0202 0101 0202 0204 0202 0101 0104 0101 0103 0101 0102 0203 0103 0202 0101 0303 0303 0204 0202 0303 0404 0101 0404 0101 0303 0303 0303 0404 0303 0202 0202 0101 0404 0103 0101 0104 0102 0104 0404 0102 0103 0101 0202 0202 0202 0303 0204 0101 0103 0103 0303 0202 0202 0202 0101 0202 0404 0202 0101 0303 0101 0101 0303 0204 0303 0404 0202 0404 0203 0202 0303 0202 0104 0303 0404 0303 0103 0103 0103 0304 0404 0202 0404 0204 0303 0103 0204 0204 0103 0101 0203 0304 0303 0404 0202 0101 0304 0304 0202 0103 0303 0101 0404 0101 0304 0202 0404 0303 0103 0404 0202 0202 0101 0202 0202 0404 0101 0202 0303 0101 0303 0202 0101 0102 0404 0102 0303 0204 0202 0404 0101 0203 0404 0103 0303 0102 0101 0404 0303 0101 0101 0202 0203 0204 0303 0303 0102 0202 0303 0303 0000 0202 0303 0303 0404 0202 0202 0103 0101 0101 0303 0204 0404 0101 0303 0202 0202 0101 0404 0103 0404 0304 0202 0404 0304 0404 0202 0203 0202 0101 0404 0303 0102 0202 0404 0303 0103 0404 0303 0202 0204 0101 0303 0101 0203 0101 0202 0103 0101 0303 0303 0101 0202 0303 0202 0404 0101 0202 0303 0303 0102 0202 0404 0202 0203 0204 0101 0404 0101 0102 0101 0101 0203 0101 0202 0101 0303 0404 0303 0404 0101 0000 0103 0202 0202 0101 0204 0101 0202 0103 0103 0202 0202 0303 0303 0303 0103 0202 0101 0202 0404 0203 0102 0101 0202 0202 0404 0202 0101 0202 0404 0202 0404 0404 0101 0303 0303 0404 0103 0303 0101 0203 0303 0103 0101 0303 0104 0202 0101 0404 0303 0304 0101 0202 0101 0101 0102 0202 0404 0303 0404 0204 0404 0404 0101 0101 0202 0404 0202 0404 0202 0101 0203 0303 0202 0404 0404 0102 0202 0101 0101 0101 0202 0204 0203 0101 0303 0202 0303 0101 0303 0202 0202 0303 0101 0404 0103 0404 0202 0303 0104 0203 0404 0103 0303 0202 0303 0101 0303 0104 0101 0101 0103 0303 0304 0202 0303 0103 0404 0101 0202 0202 0204 0101 0404 0202 0202 0202 0404 0103 0202 0202 0303 0101 0103 0104 0101 0404 0404 0102 0404 0304 0404 0101 0303 0404 0103 0303 0101 0202 0101 0303 0202 0404 0303 0202 0303 0303 0404 0101 0202 0204 0202 0202 0101 0101 0202 0303 0303 0303 0202 0203 0204 0102 0303 0202 0404 0102 0101 0404 0404 0404 0202 0202 0202 0404 0101 0303 0303 0404 0303 0202 0202 0404 0101 0204 0303 0303 0303 0103 0404 0303 0202 0202 0202 0204 0101 0404 0103 0404 0202 0101 0104 0404 0404 0102 0202 0103 0404 0202 0303 0303 0202 0404 0202 0101 0204 0202 0101 0202 0404 0103 0103 0303 0404 0404 0103 0303 0202 0303 0202 0202 0202 0101 0202 0404 0303 0303 0101 0404 0303 0303 0404 0202 0303 0104 0202 0104 0303 0103 0303 0103 0202 0204 0303 0303 0202 0101 0101 0404 0101 0202 0202 0101 0303 0202 0202 0104 0101 0103 0202 0203 0202 0204 0202 0202 0404 0303 0303 0101 0202 0202 0202 0101 0304 0202 0103 0303 0404 0104 0404 0303 0404 0202 0202 0303 0202 0202 0304 0101 0303 0202 0303 0103 0303 0303 0101 0404 0303 0101 0202 0202 0103 0101 0101 0202 0404 0202 0303 0303 0202 0303 0204 0101 0404 0202 0000 0202 0404 0404 0303 0103 0104 0101 0404 0303 0202 0404 0304 0101 0203 0303 0101 0101 0202 0204 0102 0103 0203 0202 0202 0304 0303 0103 0404 0204 0202 0303 0204 0202 0102 0203 0304 0101 0202 0202 0303 0304 0202 0303 0404 0101 0303 0303 0202 0101 0303 0404 0303 0202 0303 0303 0101 0304 0404 0202 0303 0204 0103 0202 0101 0104 0404 0303 0204 0303 0104 0204 0303 0103 0303 0101 0103 0202 0101 0202 0303 0104 0101 0103 0203 0102 0202 0303 0404 0303 0303 0202 0202 0103 0404 0404 0303 0404 0303 0202 0202 0203 0202 0202 0202 0103 0101 0202 0204 0103 0204 0202 0202 0303 0303 0101 0204 0303 0404 0202 0303 0202 0404 0202 0303 0404 0303 0101 0202 0202 0404 0404 0404 0304 0102 0404 0303 0404 0404 0303 0202 0104 0101 0101 0101 0103 0202 0101 0404 0202 0103 0404 0204 0404 0101 0202 0104 0101 0404 0202 0204 0202 0303 0204 0404 0204 0303 0202 0103 0303 0303 0203 0103 0103 0303 0103 0103 0303 0204 0303 0404 0102 0101 0103 0204 0202 0202 0204 0303 0303 0202 0204 0303 0303 0404 0202 0202 0303 0303 0202 0404 0303 0202 0303 0202 0304 0404 0101 0203 0101 0204 0103 0104 0203 0204 0303 0204 0101 0303 0103 0103 0101 0303 0303 0203 0303 0303 0202 0202 0303 0202 0202 0303 0404 0202 0204 0204 0104 0202 0202 0000 0404 0404 0202 0102 0101 0101 0304 0303 0204 0204 0101 0202 0303 0404 0303 0204 0303 0203 0202 0101 0204 0303 0104 0103 0204 0303 0303 0303 0303 0303 0303 0102 0404 0404 0303 0202 0404 0204 0202 0404 0101 0303 0101 0404 0104 0202 0202 0303 0104 0103 0104 0404 0102 0101 0303 0202 0303 0101 0404 0103 0202 0202 0204 0303 0404 0303 0202 0303 0101 0404 0404 0202 0303 0202 0103 0303 0101 0404 0101 0404 0103 0202 0103 0204 0101 0202 0204 0404 0103 0202 0404 0202 0101 0303 0102 0303 0103 0303 0203 0303 0404 0404 0404 0101 0404 0101 0404 0101 0404 0202 0101 0204 0103 0203 0204 0202 0102 0303 0202 0303 0101 0101 0304 0303 0101 0101 0101 0303 0303 0404 0404 0404 0404 0202 0202 0304 0101 0101 0303 0404 0103 0404 0404 0202 0303 0102 0204 0404 0101 0102 0202 0303 0101 0303 0103 0404 0303 0102 0202 0303 0204 0202 0404 0202 0404 0103 0103 0303 0303 0103 0303 0303 0203 0404 0304 0101 0101 0103 0202 0204 0102 0000 0101 0303 0204 0000 0303 0404 0303 0404 0102 0203 0103 0202 0202 0303 0202 0303 0303 0204 0303 0404 0304 0204 0304 0202 0203 0303 0103 0204 0204 0404 0103 0202 0202 0104 0202 0202 0303 0104 0204 0203 0102 0104 0202 0204 0303 0202 0303 0303 0304 0303 0303 0404 0103 0204 0202 0000 0202 0404 0101 0404 0204 0102 0404 0102 0404 0303 0101 0404 0404 0404 0303 0202 0303 0101 0303 0204 0101 0104 0101 0204 0303 0303 0404 0304 0304 0404 0202 0203 0103 0202 0202 0101 0103 0103 0204 0202 0404 0202 0303 0202 0204 0303 0104 0303 0404 0202 0101 0101 0101 0202 0202 0404 0303 0202 0303 0101 0202 0404 0202 0101 0202 0202 0404 0303 0404 0202 0101 0303 0303 0202 0303 0202 0404 0303 0102 0202 0101 0202 0303 0103 0202 0303 0404 0102 0303 0202 0404 0103 0104 0104 0202 0202 0304 0202 0202 0103 0202 0202 0101 0303 0404 0202 0404 0101 0202 0103 0202 0303 0101 0404 0303 0101 0303 0204 0101 0202 0303 0404 0101 0203 0404 0303 0101 0303 0404 0103 0202 0404 0202 0101 0404 0303 0304 0303 0404 0102 0303 0204 0303 0404 0202 0101 0404 0303 0102 0103 0204 0102 0303 0304 0303 0202 0202 0202 0303 0102 0404 0404 0103 0103 0303 0203 0101 0404 0202 0303 0101 0103 0303 0202 0303 0204 0101 0101 0103 0101 0202 0304 0101 0102 0303 0404 0303 0303 0203 0104 0101 0202 0303 0102 0101 0204 0202 0202 0404 0303 0303 0303 0103 0202 0304 0202 0303 0304 0101 0202 0404 0102 0202 0303 0104 0404 0404 0404 0303 0303 0202 0404 0204 0202 0204 0202 0404 0101 0202 0101 0303 0103 0303 0101 0103 0202 0303 0202 0303 0202 0203 0202 0202 0404 0101 0102 0202 0101 0204 0202 0304 0202 0404 0202 0303 0404 0101 0404 0303 0204 0103 0103 0101 0303 0202 0202 0204 0404 0202 0202 0101 0303 0101 0404 0404 0303 0303 0303 0404 0204 0103 0202 0104 0404 0404 0103 0204 0103 0304 0101 0303 0303 0101 0303 0104 0202 0101 0101 0404 0303 0103 0303 0404 0404 0303 0202 0204 0204 0303 0101 0202 0203 0303 0202 0303 0204 0304 0104 0303 0103 0204 0303 0102 0303 0303 0101 0304 0304 0304 0404 0202 0303 0404 0404 0104 0303 0202 0101 0202 0303 0202 0204 0303 0404 0404 0204 0303 0303 0101 0404 0404 0303 0101 0202 0101 0303 0000 0104 0303 0202 0101 0101 0202 0103 0202 0404 0103 0303 0101 0303 0404 0303 0404 0303 0101 0404 0404 0202 0404 0303 0202 0202 0304 0202 0303 0303 0303 0101 0404 0404 0303 0101 0303 0101 0204 0404 0101 0303 0101 0303 0101 0101 0204 0202 0102 0303 0104 0303 0202 0303 0303 0303 0303 0303 0404 0404 0202 0303 0202 0103 0404 0202 0404 0202 0303 0404 0202 0303 0303 0101 0101 0404 0202 0404 0101 0202 0404 0102 0202 0101 0304 0104 0202 0202 0104 0404 0101 0404 0202 0404 0101 0101 0101 0303 0202 0202 0202 0103 0103 0303 0202 0103 0204 0104 0202 0101 0404 0304 0103 0404 0404 0303 0303 0303 0101 0202 0202 0202 0202 0404 0202 0303 0101 0101 0204 0404 0202 0202 0303 0202 0103 0303 0202 0303 0204 0203 0102 0103 0202 0202 0202 0202 0202 0404 0303 0101 0404 0202 0102 0303 0101 0101 0303 0202 0303 0303 0303 0202 0303 0101 0303 0101 0303 0303 0202 0202 0202 0404 0303 0303 0202 0202 0404 0303 0404 0202 0103 0303 0101 0404 0404 0202 0303 0204 0103 0202 0101 0303 0101 0303 0303 0202 0202 0203 0404 0404 0101 0303 0202 0103 0303 0101 0303 0404 0103 0404 0303 0202 0304 0101 0404 0404 0103 0101 0203 0303 0202 0304 0303 0404 0202 0202 0404 0103 0202 0202 0303 0202 0203 0103 0202 0101 0404 0204 0103 0101 0404 0303 0404 0101 0303 0202 0303 0103 0101 0303 0202 0202 0101 0104 0204 0103 0303 0103 0102 0202 0101 0104 0202 0202 0404 0202 0101 0303 0303 0404 0204 0202 0303 0404 0204 0204 0103 0104 0101 0303 0104 0101 0101 0204 0202 0101 0404 0202 0204 0202 0202 0303 0404 0404 0404 0404 0404 0303 0404 0404 0101 0101 0303 0303 0303 0204 0202 0304 0101 0101 0101 0303 0404 0103 0303 0202 0101 0303 0204 0404 0304 0000 0101 0203 0202 0303 0404 0102 0204 0304 0303 0101 0101 0204 0101 0202 0203 0202 0103 0404 0101 0303 0101 0202 0103 0404 0103 0202 0203 0103 0101 0202 0202 0404 0303 0101 0404 0101 0101 0202 0303 0303 0202 0303 0203 0303 0202 0202 0202 0202 0101 0204 0303 0202 0404 0404 0103 0101 0202 0404 0202 0204 0104 0202 0103 0404 0101 0404 0101 0204 0303 0404 0101 0404 0202 0202 0101 0202 0101 0404 0103 0101 0303 0204 0202 0202 0304 0303 0404 0202 0101 0303 0202 0202 0204 0303 0104 0202 0101 0202 0204 0303 0101 0101 0303 0303 0202 0204 0303 0202 0303 0303 0202 0104 0404 0303 0303 0202 0304 0104 0202 0102 0202 0202 0202 0202 0303 0202 0103 0303 0303 0202 0202 0202 0202 0404 0303 0204 0102 0303 0303 0101 0101 0101 0303 0202 0202 0303 0404 0202 0303 0202 0204 0102 0202 0404 0202 0103 0104 0404 0404 0303 0204 0304 0202 0203 0102 0303 0204 0101 0303 0101 0204 0404 0304 0104 0202 0404 0303 0303 0404 0304 0202 0303 0104 0202 0202 0202 0103 0303 0202 0303 0303 0101 0303 0203 0101 0404 0101 0204 0304 0303 0103 0202 0404 0202 0303 0202 0404 0303 0202 0202 0204 0103 0404 0304 0103 0202 0202 0101 0202 0404 0202 0303 0102 0404 0303 0103 0304 0203 0202 0404 0203 0202 0101 0103 0202 0101 0303 0202 0303 0101 0202 0103 0202 0204 0101 0404 0303 0404 0202 0202 0404 0104 0404 0202 0101 0204 0303 0101 0303 0202 0101 0104 0404 0101 0102 0202 0202 0303 0404 0303 0101 0303 0303 0104 0304 0404 0202 0202 0304 0104 0202 0103 0404 0102 0101 0303 0103 0202 0101 0404 0202 0101 0103 0303 0101 0104 0102 0202 0202 0404 0204 0101 0202 0303 0303 0303 0202 0304 0102 0202 0303 0102 0202 0101 0304 0104 0303 0303 0202 0404 0303 0404 0202 0202 0202 0404 0101 0203 0104 0202 0404 0404 0404 0303 0303 0101 0303 0102 0303 0303 0404 0103 0101 0103 0103 0303 0404 0303 0101 0304 0304 0404 0101 0103 0101 0404 0103 0104 0404 0202 0404 0303 0101 0404 0101 0304 0204 0404 0404 0404 0202 0102 0101 0303 0101 0303 0404 0204 0204 0404 0101 0303 0202 0304 0404 0202 0202 0303 0303 0303 0303 0404 0404 0101 0203 0202 0303 0303 0204 0202 0303 0101 0104 0101 0101 0404 0104 0104 0202 0104 0303 0303 0303 0303 0404 0404 0202 0303 0304 0202 0303 0102 0202 0303 0202 0202 0102 0202 0101 0303 0102 0103 0404 0204 0101 0404 0303 0103 0101 0204 0104 0203 0303 0101 0303 0204 0303 0103 0202 0404 0101 0404 0303 0101 0202 0101 0204 0202 0204 0203 0202 0202 0101 0202 0404 0202 0202 0202 0101 0303 0101 0404 0303 0202 0202 0101 0404 0202 0102 0202 0303 0404 0303 0204 0202 0204 0303 0404 0304 0303 0104 0102 0103 0103 0101 0101 0203 0101 0202 0101 0204 0303 0103 0102 0202 0202 0404 0101 0303 0404 0303 0303 0303 0103 0304 0202 0303 0303 0204 0404 0303 0202 0101 0104 0303 0303 0202 0204 0202 0404 0203 0404 0404 0303 0404 0101 0202 0204 0404 0202 0303 0101 0202 0202 0203 0303 0202 0303 0303 0203 0202 0404 0404 0303 0202 0303 0404 0204 0303 0404 0303 0103 0304 0404 0303 0404 0202 0202 0101 0203 0101 0103 0103 0303 0303 0303 0202 0101 0202 0202 0102 0303 0104 0101 0404 0303 0104 0103 0202 0103 0303 0103 0303 0404 0202 0404 0202 0304 0404 0101 0101 0202 0303 0202 0104 0102 0202 0404 0202 0102 0203 0202 0404 0303 0404 0202 0404 0404 0202 0404 0202 0202 0404 0103 0104 0000 0101 0303 0102 0204 0404 0303 0103 0204 0204 0202 0204 0303 0101 0404 0103 0404 0304 0101 0202 0202 0101 0202 0303 0404 0202 0404 0104 0102 0404 0404 0404 0303 0202 0303 0202 0303 0101 0204 0404 0303 0404 0303 0101 0303 0404 0404 0204 0202 0303 0101 0202 0101 0104 0102 0101 0101 0101 0101 0101 0204 0404 0404 0101 0101 0103 0303 0404 0404 0404 0101 0404 0202 0202 0304 0303 0304 0404 0303 0101 0304 0404 0102 0202 0303 0203 0103 0303 0101 0404 0202 0204 0202 0101 0404 0202 0404 0204 0204 0202 0303 0404 0303 0101 0101 0203 0304 0204 0303 0404 0202 0102 0202 0404 0303 0101 0303 0404 0303 0204 0204 0404 0202 0202 0101 0203 0204 0202 0101 0203 0202 0202 0303 0202 0203 0303 0103 0202 0202 0202 0202 0202 0202 0303 0303 0202 0202 0303 0303 0101 0303 0404 0101 0404 0202 0103 0303 0202 0303 0303 0102 0103 0404 0101 0303 0204 0404 0102 0202 0404 0304 0203 0202 0101 0303 0303 0102 0404 0103 0303 0303 0304 0202 0202 0203 0303 0303 0101 0202 0202 0303 0404 0204 0101 0101 0103 0303 0202 0202 0101 0202 0102 0202 0204 0203 0204 0303 0103 0404 0303 0404 0304 0202 0101 0202 0404 0303 0202 0202 0204 0103 0303 0303 0000 0404 0303 0303 0203 0303 0303 0104 0303 0303 0304 0202 0202 0303 0202 0102 0104 0303 0202 0303 0404 0102 0303 0202 0303 0404 0103 0303 0303 0404 0404 0101 0303 0202 0204 0303 0203 0304 0303 0202 0101 0404 0404 0101 0304 0303 0204 0203 0101 0303 0404 0204 0101 0203 0101 0303 0303 0404 0303 0204 0103 0103 0303 0202 0404 0404 0202 0202 0202 0103 0202 0303 0102 0404 0303 0303 0303 0103 0303 0202 0303 0303 0102 0204 0103 0202 0303 0303 0303 0102 0303 0101 0202 0204 0404 0204 0202 0304 0202 0103 0104 0104 0303 0101 0203 0304 0404 0202 0303 0103 0202 0202 0101 0202 0102 0303 0101 0404 0303 0104 0101 0404 0303 0104 0101 0303 0303 0103 0103 0404 0404 0304 0203 0204 0102 0101 0202 0103 0304 0202 0303 0101 0303 0303 0103 0101 0303 0303 0202 0303 0101 0104 0101 0303 0202 0102 0303 0204 0303 0303 0103 0303 0103 0202 0000 0202 0204 0404 0303 0303 0202 0404 0404 0404 0202 0404 0102 0303 0303 0204 0101 0304 0203 0404 0303 0000 0303 0404 0203 0202 0101 0404 0101 0202 0202 0404 0101 0202 0101 0202 0404 0303 0303 0202 0202 0101 0101 0404 0303 0202 0404 0202 0303 0204 0101 0404 0303 0204 0202 0102 0303 0303 0404 0202 0404 0202 0202 0303 0101 0404 0303 0202 0404 0101 0101 0204 0404 0404 0204 0202 0404 0104 0404 0101 0103 0101 0101 0202 0303 0202 0303 0101 0202 0202 0101 0203 0101 0202 0303 0202 0303 0303 0101 0202 0101 0303 0303 0101 0101 0101 0104 0202 0202 0202 0202 0404 0303 0202 0103 0303 0202 0404 0404 0202 0103 0404 0103 0102 0101 0404 0404 0202 0103 0202 0303 0102 0404 0303 0404 0404 0404 0103 0103 0404 0101 0303 0103 0303 0303 0202 0103 0104 0204 0202 0404 0304 0204 0202 0204 0303 0304 0204 0204 0202 0101 0303 0303 0103 0204 0303 0303 0303 0204 0204 0303 0101 0404 0204 0404 0204 0101 0202 0202 0202 0202 0204 0202 0404 0303 0404 0202 0303 0204 0101 0104 0204 0303 0303 0103 0103 0204 0404 0202 0303 0303 0204 0202 0203 0404 0103 0202 0103 0203 0404 0202 0404 0101 0202 0303 0202 0101 0204 0303 0202 0404 0303 0101 0404 0404 0104 0202 0202 0404 0303 0202 0202 0404 0303 0303 0202 0303 0104 0303 0304 0202 0203 0101 0104 0202 0203 0202 0404 0303 0303 0303 0204 0204 0101 0404 0404 0202 0101 0202 0303 0101 0203 0303 0101 0202 0303 0101 0303 0202 0202 0103 0104 0404 0303 0202 0103 0103 0103 0204 0101 0102 0303 0404 0101 0101 0303 0102 0102 0101 0101 0101 0202 0204 0303 0202 0103 0202 0103 0303 0103 0202 0303 0303 0303 0204 0103 0102 0404 0404 0202 0404 0101 0303 0202 0303 0303 0101 0204 0101 0404 0404 0204 0303 0404 0202 0404 0202 0303 0202 0404 0303 0202 0303 0202 0202 0204 0101 0303 0101 0101 0101 0303 0404 0204 0404 0404 0202 0303 0303 0101 0303 0303 0303 0202 0404 0102 0303 0404 0101 0303 0202 0404 0404 0404 0303 0202 0103 0101 0103 0303 0204 0303 0204 0000 0202 0101 0203 0303 0303 0404 0304 0101 0102 0203 0303 0101 0202 0404 0303 0204 0303 0202 0204 0404 0404 0103 0104 0404 0303 0303 0404 0202 0404 0303 0404 0204 0101 0203 0103 0303 0101 0102 0202 0404 0202 0101 0202 0404 0102 0303 0103 0404 0101 0303 0404 0202 0202 0404 0103 0204 0202 0304 0304 0404 0303 0404 0102 0202 0202 0303 0404 0203 0103 0101 0103 0303 0404 0404 0303 0304 0202 0303 0202 0202 0101 0404 0303 0303 0101 0204 0303 0404 0404 0303 0204 0404 0404 0202 0202 0101 0204 0202 0202 0303 0103 0104 0303 0404 0202 0202 0104 0101 0202 0202 0202 0303 0103 0303 0304 0202 0404 0404 0101 0303 0202 0101 0404 0304 0101 0103 0303 0101 0404 0303 0303 0203 0103 0101 0304 0204 0204 0102 0104 0303 0101 0303 0303 0102 0303 0202 0202 0202 0303 0202 0202 0303 0202 0202 0103 0202 0202 0101 0202 0404 0303 0303 0303 0404 0101 0404 0204 0202 0202 0101 0303 0303 0304 0303 0404 0202 0404 0103 0101 0303 0303 0202 0202 0303 0204 0202 0303 0101 0304 0101 0204 0404 0404 0404 0101 0103 0104 0303 0202 0203 0102 0304 0404 0202 0101 0101 0202 0303 0404 0303 0101 0202 0203 0404 0202 0303 0303 0202 0101 0303 0101 0404 0404 0204 0202 0303 0202 0303 0303 0202 0101 0303 0303 0101 0404 0204 0202 0303 0303 0303 0404 0204 0102 0303 0103 0303 0103 0202 0202 0101 0103 0303 0303 0303 0404 0103 0404 0303 0101 0101 0404 0404 0303 0101 0404 0303 0204 0202 0202 0303 0404 0203 0202 0404 0303 0303 0404 0104 0303 0404 0204 0101 0101 0103 0303 0202 0202 0303 0102 0303 0202 0303 0304 0101 0303 0303 0303 0303 0404 0404 0101 0101 0303 0101 0204 0101 0101 0103 0303 0104 0101 0202 0404 0303 0404 0303 0103 0202 0404 0202 0303 0303 0204 0304 0202 0404 0202 0202 0101 0303 0101 0303 0202 0103 0101 0202 0303 0204 0101 0404 0204 0303 0204 0103 0303 0101 0202 0303 0202 0101 0104 0101 0101 0204 0101 0404 0101 0103 0202 0101 0202 0303 0202 0404 0203 0104 0404 0101 0202 0204 0202 0303 0203 0304 0303 0202 0404 0101 0204 0303 0202 0404 0404 0202 0303 0202 0303 0101 0404 0102 0103 0404 0404 0404 0204 0102 0101 0404 0303 0404 0103 0304 0101 0303 0101 0101 0303 0202 0303 0303 0404 0404 0404 0303 0303 0303 0101 0204 0202 0303 0404 0304 0204 0303 0104 0103 0103 0203 0204 0404 0303 0101 0202 0303 0303 0303 0202 0000 0102 0202 0103 0202 0103 0202 0202 0303 0303 0303 0000 0202 0202 0103 0303 0404 0202 0303 0101 0202 0404 0202 0404 0404 0202 0104 0103 0202 0404 0204 0204 0202 0303 0103 0303 0203 0404 0103 0101 0202 0101 0202 0203 0204 0000 0404 0404 0204 0101 0101 0303 0303 0101 0303 0101 0204 0404 0303 0303 0101 0101 0303 0303 0202 0303 0404 0101 0303 0204 0202 0303 0102 0303 0303 0303 0303 0404 0404 0404 0303 0404 0202 0404 0404 0202 0202 0303 0202 0101 0404 0103 0202 0202 0202 0101 0303 0101 0404 0303 0101 0103 0101 0202 0303 0203 0303 0303 0103 0202 0202 0404 0204 0204 0303 0404 0202 0202 0303 0202 0404 0101 0104 0303 0202 0101 0202 0404 0202 0203 0303 0202 0404 0303 0303 0204 0101 0303 0202 0202 0104 0102 0303 0203 0404 0202 0103 0303 0303 0404 0202 0101 0303 0202 0102 0101 0303 0203 0103 0202 0404 0404 0303 0202 0202 0202 0303 0101 0202 0202 0103 0000 0202 0303 0303 0303 0404 0103 0303 0304 0404 0103 0303 0303 0102 0202 0203 0303 0303 0101 0102 0303 0103 0303 0101 0304 0104 0303 0404 0404 0404 0303 0101 0404 0202 0202 0202 0404 0404 0101 0303 0101 0202 0204 0202 0104 0103 0204 0404 0303 0404 0202 0202 0303 0202 0303 0203 0202 0303 0303 0104 0404 0101 0202 0404 0103 0202 0404 0202 0404 0103 0303 0404 0404 0202 0204 0304 0303 0203 0204 0202 0202 0204 0103 0303 0304 0303 0303 0202 0304 0102 0101 0103 0103 0202 0202 0204 0303 0103 0202 0204 0204 0101 0202 0101 0101 0103 0101 0101 0202 0202 0204 0202 0404 0304 0203 0101 0203 0204 0303 0103 0303 0202 0304 0202 0101 0303 0202 0304 0101 0303 0101 0202 0303 0303 0404 0202 0202 0404 0202 0404 0101 0202 0103 0304 0104 0404 0202 0101 0204 0202 0101 0404 0303 0101 0202 0303 0202 0202 0103 0000 0202 0303 0303 0204 0304 0303 0303 0202 0404 0303 0202 0404 0404 0202 0204 0404 0404 0404 0202 0101 0404 0303 0303 0303 0303 0101 0404 0204 0104 0303 0404 0203 0103 0304 0101 0303 0204 0202 0303 0202 0303 0202 0101 0101 0202 0101 0101 0203 0303 0103 0103 0303 0202 0102 0404 0103 0303 0404 0202 0104 0404 0202 0404 0101 0203 0101 0202 0101 0101 0404 0202 0102 0303 0103 0202 0103 0404 0202 0202 0303 0203 0102 0303 0303 0404 0404 0102 0101 0101 0204 0202 0303 0202 0303 0404 0404 0304 0204 0202 0404 0202 0203 0202 0104 0202 0303 0103 0303 0204 0102 0101 0202 0204 0203 0103 0304 0303 0101 0101 0303 0404 0202 0202 0303 0204 0304 0303 0404 0103 0103 0101 0404 0202 0203 0202 0204 0101 0404 0303 0404 0101 0303 0203 0202 0404 0404 0101 0404 0202 0102 0404 0202 0101 0303 0303 0204 0303 0303 0303 0203 0303 0202 0101 0204 0202 0104 0202 0202 0103 0103 0404 0202 0404 0202 0204 0202 0202 0404 0202 0204 0303 0303 0101 0101 0303 0404 0304 0303 0102 0202 0204 0104 0204 0303 0303 0101 0102 0101 0202 0303 0102 0103 0303 0404 0104 0102 0303 0101 0104 0404 0103 0202 0303 0204 0101 0404 0202 0303 0103 0104 0202 0204 0102 0304 0103 0203 0303 0303 0202 0202 0204 0303 0103 0303 0303 0202 0303 0203 0203 0101 0202 0104 0202 0102 0404 0404 0101 0303 0303 0304 0203 0204 0303 0404 0202 0203 0101 0103 0101 0404 0103 0103 0101 0303 0303 0202 0202 0303 0202 0204 0303 0303 0404 0404 0202 0202 0204 0204 0101 0303 0202 0000 0202 0404 0104 0303 0101 0103 0103 0103 0404 0204 0303 0303 0404 0303 0303 0101 0103 0102 0404 0103 0202 0103 0303 0303 0104 0303 0103 0103 0303 0104 0303 0101 0303 0404 0101 0104 0204 0204 0103 0202 0101 0404 0101 0404 0204 0202 0404 0303 0202 0101 0303 0103 0202 0404 0202 0104 0103 0104 0404 0202 0101 0303 0404 0202 0101 0202 0204 0202 0202 0303 0101 0104 0202 0204 0103 0202 0202 0404 0101 0303 0202 0404 0204 0202 0404 0103 0303 0404 0303 0101 0101 0204 0104 0203 0303 0101 0404 0202 0101 0101 0304 0103 0303 0303 0204 0404 0303 0303 0203 0202 0101 0204 0303 0404 0101 0303 0202 0204 0101 0303 0101 0103 0102 0101 0404 0104 0202 0303 0202 0204 0303 0202 0204 0303 0202 0202 0101 0303 0101 0103 0303 0304 0404 0303 0103 0202 0404 0102 0103 0303 0303 0202 0202 0202 0203 0404 0304 0303 0103 0204 0303 0202 0204 0101 0101 0303 0202 0202 0202 0303 0404 0404 0303 0303 0303 0202 0303 0304 0404 0204 0101 0304 0304 0404 0303 0204 0303 0303 0101 0303 0101 0404 0303 0202 0102 0404 0103 0303 0303 0303 0303 0103 0203 0101 0101 0404 0202 0202 0404 0101 0304 0404 0204 0101 0101 0103 0303 0102 0404 0202 0101 0101 0303 0202 0101 0101 0404 0103 0101 0202 0303 0101 0204 0202 0202 0202 0101 0404 0202 0202 0303 0101 0404 0303 0203 0202 0404 0404 0103 0202 0303 0303 0202 0101 0203 0303 0101 0404 0103 0103 0303 0103 0404 0303 0202 0303 0303 0202 0202 0404 0204 0404 0202 0404 0303 0203 0303 0101 0202 0101 0101 0202 0101 0103 0202 0202 0103 0404 0202 0202 0202 0103 0103 0202 0303 0303 0404 0101 0203 0202 0203 0202 0404 0303 0103 0404 0404 0404 0202 0101 0404 0102 0303 0202 0101 0202 0101 0303 0103 0101 0303 0304 0104 0102 0303 0202 0101 0303 0202 0103 0202 0103 0404 0101 0202 0404 0304 0202 0404 0204 0303 0204 0202 0204 0404 0202 0102 0101 0303 0303 0202 0304 0204 0104 0303 0101 0303 0202 0202 0101 0303 0303 0101 0404 0103 0104 0101 0404 0202 0204 0104 0202 0101 0203 0203 0101 0304 0404 0404 0303 0103 0303 0303 0304 0101 0204 0303 0103 0404 0303 0202 0101 0202 0303 0204 0101 0104 0404 0404 0204 0103 0404 0303 0303 0303 0202 0304 0103 0303 0404 0203 0101 0204 0303 0202 0303 0202 0204 0202 0204 0104 0103 0101 0303 0104 0303 0102 0101 0104 0204 0101 0303 0101 0303 0101 0303 0202 0404 0303 0303 0204 0303 0101 0202 0303 0202 0104 0303 0101 0404 0404 0404 0202 0304 0202 0000 0204 0104 0203 0404 0000 0204 0304 0303 0202 0102 0204 0404 0103 0103 0104 0202 0101 0303 0404 0203 0102 0303 0303 0303 0204 0303 0103 0101 0304 0303 0404 0404 0303 0202 0303 0404 0202 0303 0303 0101 0303 0303 0204 0103 0303 0202 0303 0303 0404 0404 0303 0101 0202 0103 0303 0202 0203 0101 0204 0103 0303 0103 0102 0303 0203 0202 0303 0202 0202 0404 0103 0202 0404 0102 0101 0404 0101 0103 0101 0202 0404 0303 0202 0202 0404 0202 0102 0101 0101 0204 0303 0202 0104 0101 0101 0404 0202 0404 0104 0101 0101 0203 0202 0404 0404 0202 0404 0204 0303 0303 0202 0304 0303 0404 0102 0404 0101 0404 0202 0303 0202 0303 0303 0103 0102 0202 0202 0101 0204 0202 0404 0103 0202 0304 0404 0303 0202 0404 0204 0202 0303 0203 0404 0303 0102 0303 0303 0303 0202 0202 0404 0202 0404 0202 0204 0204 0102 0204 0202 0204 0304 0104 0303 0101 0202 0202 0204 0103 0303 0303 0304 0103 0303 0303 0202 0103 0101 0404 0404 0303 0404 0204 0303 0404 0101 0202 0404 0304 0101 0303 0101 0303 0101 0104 0404 0303 0404 0303 0404 0303 0101 0202 0303 0202 0101 0404 0202 0202 0303 0101 0202 0202 0404 0202 0104 0303 0303 0303 0202 0202 0101 0101 0303 0204 0103 0303 0404 0303 0303 0404 0204 0202 0202 0202 0202 0204 0303 0103 0101 0202 0303 0404 0102 0101 0303 0103 0101 0101 0101 0303 0404 0202 0101 0101 0101 0101 0202 0303 0202 0303 0103 0304 0101 0202 0303 0103 0303 0104 0303 0101 0303 0101 0202 0101 0303 0404 0404 0303 0202 0404 0102 0303 0103 0101 0303 0303 0202 0203 0404 0104 0204 0303 0404 0202 0101 0101 0101 0303 0303 0404 0204 0101 0202 0104 0404 0202 0303 0204 0303 0303 0101 0303 0404 0303 0202 0101 0303 0303 0202 0104 0404 0202 0404 0203 0202 0303 0404 0303 0303 0101 0202 0103 0303 0404 0304 0304 0303 0303 0102 0303 0103 0103 0101 0303 0404 0303 0204 0303 0202 0404 0303 0303 0101 0101 0303 0202 0303 0101 0103 0203 0303 0102 0404 0404 0204 0303 0303 0101 0303 0101 0304 0404 0303 0202 0204 0204 0103 0303 0202 0101 0104 0404 0202 0404 0202 0101 0202 0101 0103 0202 0101 0103 0404 0103 0204 0102 0101 0303 0304 0103 0104 0303 0303 0303 0101 0202 0304 0204 0404 0202 0303 0202 0202 0303 0202 0103 0202 0204 0101 0104 0404 0404 0304 0202 0202 0203 0303 0404 0204 0303 0101 0102 0203 0101 0103 0303 0202 0103 0101 0101 0204 0303 0303 0204 0204 0102 0202 0404 0104 0303 0102 0202 0204 0102 0202 0304 0303 0202 0203 0101 0204 0303 0304 0303 0303 0101 0202 0202 0103 0404 0303 0104 0303 0202 0303 0202 0304 0202 0204 0404 0103 0303 0202 0204 0102 0202 0101 0303 0303 0101 0202 0204 0303 0202 0102 0202 0103 0102 0202 0202 0204 0204 0303 0203 0202 0101 0101 0404 0404 0404 0303 0203 0404 0204 0202 0303 0202 0103 0204 0101 0202 0303 0304 0104 0103 0101 0404 0303 0202 0404 0204 0303 0304 0304 0303 0103 0103 0404 0202 0202 0102 0101 0303 0303 0202 0404 0102 0303 0103 0204 0104 0103 0101 0103 0103 0304 0103 0101 0304 0404 0304 0202 0101 0104 0103 0404 0404 0202 0202 0101 0104 0303 0404 0101 0202 0202 0101 0101 0404 0104 0202 0102 0404 0202 0101 0101 0202 0204 0303 0204 0101 0102 0101 0304 0101 0104 0204 0303 0104 0303 0303 0102 0202 0404 0303 0303 0101 0104 0202 0103 0303 0103 0103 0202 0101 0204 0303 0303 0303 0303 0303 0101 0303 0203 0303 0202 0101 0303 0103 0202 0404 0203 0101 0404 0203 0103 0204 0202 0404 0202 0101 0404 0104 0303 0202 0404 0103 0104 0303 0202 0204 0202 0101 0204 0204 0101 0202 0204 0202 0202 0101 0303 0101 0204 0101 0202 0303 0102 0303 0204 0202 0202 0202 0101 0202 0101 0101 0101 0103 0202 0304 0204 0103 0304 0204 0202 0102 0202 0101 0303 0104 0202 0303 0204 0202 0102 0102 0203 0101 0202 0101 0103 0202 0101 0304 0404 0101 0104 0202 0303 0202 0404 0101 0303 0202 0303 0303 0303 0404 0303 0104 0304 0303 0103 0204 0404 0202 0303 0303 0303 0303 0101 0202 0404 0303 0204 0202 0303 0202 0101 0404 0304 0303 0103 0202 0101 0303 0202 0101 0303 0304 0303 0103 0202 0204 0404 0202 0202 0101 0202 0102 0202 0102 0101 0404 0303 0404 0101 0303 0404 0202 0000 0303 0202 0404 0202 0202 0103 0202 0303 0101 0104 0303 0404 0304 0303 0101 0404 0202 0303 0103 0204 0204 0404 0204 0202 0303 0202 0101 0202 0303 0204 0404 0203 0103 0202 0303 0204 0404 0404 0404 0103 0204 0101 0102 0101 0101 0404 0204 0204 0304 0404 0202 0404 0404 0304 0303 0101 0103 0202 0202 0303 0303 0204 0204 0404 0102 0404 0101 0303 0303 0202 0202 0203 0303 0202 0102 0303 0104 0303 0101 0304 0404 0202 0303 0202 0202 0202 0303 0303 0303 0202 0103 0303 0304 0202 0103 0303 0303 0404 0202 0303 0103 0404 0303 0204 0303 0101 0204 0204 0202 0303 0404 0103 0404 0103 0101 0404 0404 0101 0404 0303 0101 0103 0101 0203 0101 0102 0404 0404 0404 0303 0404 0101 0304 0303 0404 0304 0103 0303 0303 0202 0303 0304 0103 0202 0303 0202 0103 0103 0404 0303 0202 0303 0102 0303 0404 0202 0204 0202 0303 0303 0101 0303 0202 0304 0104 0404 0202 0303 0303 0101 0404 0303 0202 0202 0101 0404 0101 0204 0303 0303 0304 0202 0101 0202 0202 0303 0204 0404 0303 0104 0404 0404 0304 0304 0202 0104 0202 0303 0102 0202 0404 0202 0101 0303 0303 0202 0404 0101 0202 0404 0404 0303 0202 0102 0202 0101 0303 0101 0202 0404 0202 0202 0202 0304 0202 0103 0204 0303 0303 0202 0202 0404 0303 0202 0202 0202 0203 0101 0102 0303 0303 0103 0304 0103 0104 0204 0404 0303 0303 0101 0204 0303 0202 0103 0303 0404 0101 0303 0103 0303 0303 0404 0404 0202 0203 0203 0104 0404 0101 0303 0303 0303 0104 0303 0404 0303 0202 0202 0204 0202 0202 0202 0303 0304 0101 0404 0202 0404 0101 0104 0303 0404 0103 0303 0404 0103 0103 0104 0202 0303 0303 0101 0303 0204 0204 0404 0303 0202 0204 0303 0202 0204 0202 0103 0103 0404 0101 0303 0101 0202 0303 0102 0104 0404 0202 0104 0202 0103 0303 0202 0202 0104 0404 0303 0101 0202 0303 0404 0202 0202 0303 0303 0102 0303 0404 0202 0101 0404 0204 0202 0303 0202 0202 0404 0303 0101 0303 0303 0202 0202 0202 0404 0303 0303 0303 0204 0103 0203 0101 0202 0303 0202 0202 0101 0303 0102 0202 0303 0303 0303 0303 0404 0404 0404 0202 0304 0202 0202 0404 0404 0404 0202 0303 0103 0303 0202 0304 0204 0404 0303 0303 0202 0303 0303 0202 0101 0303 0303 0303 0202 0204 0103 0303 0202 0303 0404 0101 0202 0202 0102 0303 0303 0303 0202 0204 0101 0101 0203 0203 0202 0204 0404 0303 0204 0202 0202 0000 0103 0404 0104 0102 0303 0101 0303 0404 0202 0104 0204 0303 0303 0202 0202 0202 0202 0101 0404 0303 0303 0303 0204 0202 0101 0103 0404 0304 0103 0303 0101 0202 0104 0202 0404 0404 0303 0102 0303 0303 0304 0303 0303 0202 0101 0303 0303 0101 0202 0202 0204 0202 0202 0304 0202 0101 0104 0404 0101 0303 0102 0404 0304 0303 0202 0202 0304 0303 0101 0202 0404 0202 0303 0101 0303 0404 0303 0303 0202 0303 0404 0404 0101 0303 0404 0202 0303 0202 0103 0202 0203 0101 0204 0202 0404 0202 0202 0204 0202 0404 0101 0303 0303 0101 0204 0202 0304 0202 0404 0202 0204 0101 0404 0101 0101 0103 0102 0101 0102 0102 0101 0303 0204 0404 0404 0404 0202 0101 0202 0101 0104 0101 0202 0204 0303 0303 0101 0202 0202 0104 0303 0303 0104 0404 0303 0404 0202 0203 0303 0303 0101 0101 0202 0204 0404 0103 0104 0101 0101 0404 0404 0202 0303 0101 0404 0202 0404 0303 0202 0203 0303 0101 0304 0101 0101 0404 0101 0404 0101 0204 0303 0404 0102 0204 0202 0101 0104 0404 0404 0103 0303 0202 0303 0303 0101 0103 0202 0103 0202 0202 0202 0101 0101 0202 0103 0101 0404 0102 0202 0103 0303 0202 0101 0404 0404 0204 0204 0404 0404 0103 0204 0303 0202 0103 0303 0303 0303 0102 0204 0303 0101 0304 0303 0404 0104 0104 0304 0404 0304 0303 0303 0202 0101 0103 0101 0204 0202 0101 0303 0101 0303 0304 0202 0303 0204 0303 0101 0303 0202 0204 0304 0404 0204 0202 0101 0103 0101 0103 0404 0303 0204 0304 0404 0101 0303 0203 0304 0000 0303 0303 0303 0303 0204 0103 0202 0103 0303 0303 0204 0303 0202 0404 0203 0101 0104 0102 0303 0202 0404 0202 0303 0303 0204 0101 0103 0303 0303 0303 0203 0102 0104 0104 0202 0103 0101 0404 0303 0202 0303 0204 0404 0101 0202 0303 0303 0404 0101 0404 0203 0304 0204 0404 0104 0404 0204 0202 0101 0303 0404 0101 0303 0102 0303 0303 0303 0202 0102 0303 0103 0101 0101 0102 0202 0103 0303 0202 0204 0202 0404 0404 0202 0304 0103 0404 0303 0103 0203 0103 0103 0303 0202 0202 0303 0204 0103 0102 0303 0404 0304 0303 0303 0101 0303 0303 0103 0303 0303 0404 0202 0404 0303 0101 0202 0303 0404 0101 0103 0202 0303 0202 0303 0404 0101 0404 0202 0303 0101 0404 0202 0304 0303 0204 0101 0202 0101 0203 0404 0101 0303 0101 0303 0404 0103 0102 0202 0303 0404 0202 0101 0404 0101 0202 0303 0303 0303 0303 0303 0101 0303 0202 0103 0101 0202 0101 0303 0103 0202 0202 0101 0101 0104 0404 0303 0303 0404 0202 0303 0303 0202 0203 0202 0203 0202 0204 0404 0404 0202 0202 0204 0202 0303 0202 0101 0202 0101 0101 0202 0101 0202 0102 0404 0101 0303 0204 0101 0102 0103 0204 0101 0303 0303 0303 0202 0101 0102 0101 0303 0304 0303 0404 0404 0202 0303 0303 0101 0204 0103 0303 0103 0303 0104 0101 0202 0202 0303 0101 0202 0101 0204 0204 0203 0101 0104 0202 0303 0104 0204 0304 0202 0101 0101 0101 0202 0303 0404 0404 0102 0202 0303 0101 0101 0303 0103 0104 0102 0303 0303 0202 0101 0101 0101 0202 0103 0404 0404 0204 0101 0101 0101 0203 0101 0303 0202 0404 0303 0303 0202 0304 0104 0404 0203 0103 0303 0101 0101 0203 0404 0304 0404 0103 0202 0202 0104 0101 0404 0101 0304 0104 0303 0104 0404 0202 0202 0404 0101 0404 0404 0404 0204 0104 0103 0404 0404 0000 0202 0404 0101 0104 0104 0202 0204 0303 0104 0202 0202 0101 0104 0101 0303 0202 0404 0303 0404 0303 0202 0404 0303 0202 0103 0404 0404 0303 0104 0404 0203 0103 0202 0303 0101 0303 0202 0204 0101 0303 0404 0101 0101 0202 0102 0303 0204 0303 0202 0303 0303 0202 0101 0303 0202 0202 0102 0103 0102 0303 0202 0204 0303 0304 0202 0103 0404 0303 0303 0103 0303 0404 0202 0303 0303 0303 0104 0202 0303 0103 0202 0204 0303 0101 0101 0202 0203 0204 0202 0202 0202 0303 0204 0204 0303 0404 0101 0404 0303 0303 0202 0202 0303 0202 0204 0404 0101 0304 0404 0103 0102 0101 0204 0101 0304 0404 0404 0104 0101 0101 0303 0102 0202 0101 0202 0102 0102 0404 0203 0101 0101 0101 0101 0203 0102 0404 0304 0102 0303 0404 0103 0202 0202 0202 0202 0303 0404 0103 0202 0404 0202 0103 0101 0103 0202 0303 0101 0303 0202 0103 0103 0202 0404 0101 0101 0101 0101 0102 0303 0404 0102 0102 0202 0303 0101 0303 0202 0204 0103 0101 0104 0202 0202 0204 0303 0303 0101 0304 0202 0303 0202 0303 0101 0303 0101 0101 0303 0204 0103 0404 0404 0101 0102 0404 0202 0101 0304 0304 0101 0404 0303 0202 0202 0303 0202 0303 0202 0103 0303 0202 0404 0204 0103 0204 0303 0303 0202 0303 0101 0202 0404 0102 0404 0404 0202 0404 0202 0102 0404 0303 0303 0101 0202 0204 0101 0303 0202 0202 0202 0101 0404 0101 0202 0101 0404 0202 0103 0102 0204 0101 0101 0204 0404 0202 0202 0204 0303 0303 0101 0102 0202 0101 0404 0404 0404 0304 0202 0303 0404 0104 0303 0101 0404 0102 0202 0202 0102 0202 0404 0202 0103 0104 0202 0404 0404 0303 0202 0404 0101 0202 0101 0202 0303 0204 0204 0404 0304 0101 0101 0104 0101 0202 0303 0303 0202 0404 0101 0303 0303 0202 0101 0101 0204 0203 0101 0104 0102 0303 0204 0303 0303 0303 0303 0103 0304 0204 0303 0102 0101 0202 0103 0204 0204 0202 0101 0303 0202 0303 0202 0404 0404 0102 0204 0303 0202 0303 0202 0202 0202 0202 0202 0404 0101 0202 0202 0000 0101 0204 0202 0101 0202 0101 0303 0303 0101 0103 0104 0202 0303 0303 0202 0404 0303 0303 0303 0101 0303 0404 0202 0204 0101 0204 0202 0303 0102 0202 0303 0303 0404 0204 0202 0101 0202 0303 0202 0303 0103 0303 0202 0202 0102 0303 0204 0303 0104 0101 0104 0204 0303 0103 0204 0204 0202 0103 0202 0303 0303 0202 0202 0303 0101 0204 0103 0204 0303 0404 0303 0204 0303 0202 0404 0101 0202 0303 0204 0101 0303 0303 0304 0101 0204 0204 0101 0202 0303 0404 0303 0101 0101 0101 0202 0104 0101 0101 0303 0303 0404 0303 0104 0101 0202 0404 0202 0101 0304 0101 0303 0101 0404 0404 0103 0101 0404 0303 0304 0303 0104 0104 0303 0202 0104 0303 0204 0101 0303 0404 0404 0303 0303 0303 0101 0304 0303 0101 0303 0303 0202 0404 0404 0101 0404 0303 0303 0202 0101 0101 0303 0202 0101 0202 0202 0404 0101 0303 0202 0202 0203 0202 0303 0404 0101 0202 0303 0404 0404 0202 0202 0404 0203 0104 0101 0101 0404 0202 0303 0202 0103 0101 0202 0202 0101 0303 0202 0303 0101 0101 0303 0104 0404 0103 0304 0101 0101 0202 0404 0103 0404 0303 0404 0202 0303 0202 0303 0104 0404 0103 0303 0103 0304 0303 0202 0104 0202 0303 0303 0202 0203 0101 0303 0101 0202 0404 0303 0303 0304 0204 0404 0101 0303 0202 0000 0202 0204 0202 0104 0204 0202 0404 0101 0204 0303 0103 0304 0404 0303 0303 0202 0202 0202 0103 0101 0101 0303 0304 0202 0104 0303 0101 0202 0303 0303 0101 0202 0202 0202 0304 0404 0204 0202 0303 0101 0404 0103 0204 0404 0303 0404 0103 0202 0103 0101 0404 0103 0202 0303 0202 0303 0404 0101 0103 0202 0303 0404 0202 0303 0202 0404 0102 0202 0202 0202 0404 0303 0202 0304 0204 0303 0203 0404 0304 0202 0202 0203 0202 0303 0202 0303 0101 0102 0304 0102 0303 0202 0103 0202 0202 0404 0303 0202 0303 0101 0404 0303 0202 0102 0303 0404 0103 0102 0202 0202 0102 0203 0102 0202 0303 0202 0303 0103 0304 0303 0404 0101 0303 0303 0102 0204 0101 0202 0202 0202 0101 0202 0303 0303 0103 0404 0202 0204 0101 0303 0304 0202 0404 0104 0303 0101 0404 0204 0103 0303 0103 0202 0404 0303 0202 0303 0404 0101 0202 0101 0303 0101 0101 0303 0202 0303 0101 0303 0202 0101 0202 0202 0202 0202 0204 0404 0303 0303 0204 0101 0204 0303 0404 0101 0101 0103 0101 0204 0404 0101 0202 0404 0404 0202 0101 0101 0202 0202 0204 0104 0000 0102 0101 0202 0103 0101 0103 0103 0202 0202 0202 0404 0202 0404 0202 0303 0404 0102 0202 0404 0303 0103 0101 0204 0303 0303 0404 0202 0404 0202 0303 0104 0202 0303 0102 0303 0202 0404 0404 0404 0101 0103 0202 0404 0104 0104 0303 0103 0303 0202 0202 0202 0404 0104 0404 0304 0103 0204 0303 0202 0303 0101 0202 0101 0103 0303 0404 0404 0204 0103 0103 0202 0102 0202 0303 0404 0202 0202 0202 0103 0303 0303 0101 0101 0202 0303 0101 0204 0202 0202 0202 0404 0202 0101 0202 0204 0304 0204 0202 0404 0104 0101 0204 0104 0303 0202 0202 0404 0103 0203 0103 0204 0303 0404 0204 0202 0202 0203 0303 0102 0204 0303 0202 0404 0102 0202 0101 0204 0404 0202 0102 0101 0103 0404 0101 0101 0101 0101 0303 0303 0204 0204 0202 0404 0104 0303 0303 0202 0101 0204 0404 0303 0202 0103 0404 0303 0404 0203 0203 0101 0202 0204 0303 0303 0303 0104 0303 0101 0404 0404 0101 0202 0404 0404 0101 0303 0103 0103 0404 0304 0303 0101 0202 0303 0303 0303 0204 0404 0204 0303 0104 0103 0404 0202 0202 0404 0204 0101 0202 0104 0303 0303 0404 0202 0103 0404 0404 0101 0101 0404 0303 0404 0404 0202 0404 0102 0202 0202 0202 0303 0202 0101 0404 0202 0304 0101 0303 0303 0204 0101 0404 0404 0104 0101 0103 0202 0103 0404 0102 0102 0303 0103 0101 0204 0303 0204 0304 0204 0204 0202 0202 0204 0404 0303 0202 0101 0303 0202 0303 0103 0202 0101 0303 0303 0103 0103 0104 0101 0204 0304 0404 0202 0101 0204 0303 0101 0101 0303 0101 0202 0202 0101 0204 0404 0204 0103 0103 0203 0102 0101 0103 0202 0404 0202 0303 0103 0204 0404 0202 0202 0103 0101 0202 0202 0202 0303 0103 0404 0101 0202 0303 0202 0102 0202 0202 0101 0303 0202 0404 0104 0202 0202 0303 0204 0303 0303 0404 0101 0404 0204 0303 0202 0404 0204 0303 0404 0303 0102 0304 0404 0404 0202 0101 0104 0202 0104 0404 0202 0204 0101 0202 0101 0104 0303 0101 0404 0101 0202 0101 0204 0404 0202 0404 0303 0101 0303 0303 0103 0102 0101 0202 0303 0404 0404 0404 0103 0303 0303 0101 0404 0202 0103 0303 0104 0202 0303 0204 0101 0103 0303 0303 0303 0101 0102 0303 0202 0101 0204 0203 0101 0103 0303 0404 0101 0304 0303 0404 0202 0204 0202 0202 0303 0404 0404 0101 0303 0204 0303 0101 0101 0203 0204 0303 0202 0101 0103 0103 0303 0404 0303 0303 0202 0202 0202 0303 0303 0101 0203 0101 0303 0303 0204 0101 0303 0103 0103 0202 0204 0202 0202 0101 0101 0404 0303 0404 0303 0404 0203 0303 0202 0404 0204 0303 0303 0303 0304 0101 0202 0101 0404 0101 0303 0202 0103 0404 0202 0101 0404 0303 0204 0303 0303 0101 0303 0404 0303 0404 0204 0304 0204 0202 0202 0202 0101 0303 0202 0202 0303 0202 0102 0303 0303 0303 0202 0202 0303 0303 0103 0303 0404 0303 0202 0303 0202 0404 0101 0202 0303 0101 0202 0303 0303 0202 0404 0101 0204 0303 0203 0404 0101 0103 0303 0202 0202 0101 0202 0404 0202 0103 0202 0101 0202 0104 0404 0303 0202 0303 0102 0303 0202 0104 0304 0202 0204 0202 0101 0303 0404 0404 0303 0101 0101 0303 0404 0404 0202 0202 0303 0104 0202 0303 0202 0202 0303 0303 0101 0103 0101 0101 0204 0202 0103 0404 0404 0303 0303 0304 0404 0204 0202 0204 0202 0202 0303 0202 0303 0202 0101 0202 0202 0404 0102 0202 0202 0102 0303 0202 0204 0101 0202 0204 0303 0404 0101 0204 0202 0202 0303 0101 0303 0204 0101 0202 0202 0104 0204 0101 0303 0103 0303 0404 0101 0204 0303 0303 0101 0101 0203 0303 0103 0202 0303 0304 0404 0202 0204 0204 0103 0202 0202 0202 0202 0103 0102 0404 0303 0202 0404 0204 0101 0202 0303 0104 0202 0303 0404 0102 0303 0202 0404 0202 0202 0202 0103 0103 0204 0101 0202 0404 0303 0303 0202 0404 0101 0202 0202 0102 0101 0303 0303 0203 0202 0101 0202 0303 0202 0202 0102 0101 0304 0101 0404 0404 0103 0404 0202 0202 0303 0202 0204 0104 0304 0404 0203 0101 0101 0204 0303 0101 0203 0404 0103 0202 0303 0101 0204 0404 0202 0102 0404 0103 0202 0104 0304 0101 0102 0202 0204 0404 0303 0204 0303 0404 0202 0303 0404 0202 0202 0303 0102 0304 0102 0303 0202 0203 0304 0202 0303 0303 0303 0103 0303 0304 0303 0204 0303 0203 0404 0101 0303 0204 0101 0202 0303 0202 0303 0404 0303 0202 0101 0101 0303 0101 0202 0404 0102 0103 0204 0202 0101 0303 0202 0104 0202 0103 0103 0404 0202 0303 0303 0103 0404 0202 0303 0404 0202 0103 0202 0202 0303 0204 0101 0303 0303 0103 0303 0202 0404 0101 0303 0101 0404 0404 0101 0404 0303 0102 0104 0204 0304 0303 0101 0404 0404 0404 0404 0101 0303 0303 0404 0101 0404 0103 0303 0101 0101 0202 0103 0202 0202 0202 0103 0202 0304 0204 0303 0303 0202 0202 0404 0204 0104 0303 0404 0101 0303 0101 0202 0101 0102 0303 0303 0102 0104 0202 0303 0303 0404 0303 0102 0303 0304 0204 0101 0202 0203 0202 0204 0303 0101 0202 0303 0202 0103 0202 0304 0303 0101 0303 0303 0101 0204 0101 0304 0101 0303 0303 0204 0303 0202 0202 0202 0303 0304 0303 0303 0101 0101 0000 0404 0202 0202 0202 0204 0101 0202 0303 0303 0202 0204 0204 0103 0202 0202 0101 0303 0404 0101 0203 0303 0202 0303 0404 0404 0101 0202 0404 0202 0303 0203 0404 0101 0101 0303 0404 0404 0303 0202 0404 0303 0304 0204 0303 0101 0404 0404 0202 0304 0102 0404 0202 0303 0404 0303 0204 0303 0203 0202 0303 0404 0404 0101 0101 0103 0304 0202 0202 0404 0404 0303 0204 0303 0204 0204 0102 0404 0101 0204 0404 0104 0404 0101 0204 0202 0303 0204 0202 0404 0101 0203 0204 0303 0404 0303 0202 0303 0101 0204 0404 0303 0103 0202 0303 0204 0404 0102 0202 0204 0303 0104 0101 0202 0404 0303 0202 0303 0303 0202 0102 0404 0303 0202 0202 0101 0202 0203 0101 0101 0404 0202 0404 0303 0101 0303 0202 0303 0101 0202 0202 0303 0303 0202 0202 0204 0202 0303 0202 0203 0202 0202 0101 0404 0101 0204 0101 0102 0204 0202 0202 0303 0202 0303 0303 0202 0101 0101 0404 0303 0303 0101 0202 0303 0204 0202 0101 0104 0202 0404 0202 0101 0404 0303 0202 0303 0204 0202 0304 0303 0101 0202 0303 0202 0404 0103 0202 0102 0202 0103 0202 0101 0101 0404 0404 0204 0101 0303 0202 0303 0303 0404 0204 0304 0102 0103 0303 0101 0102 0303 0303 0303 0103 0304 0202 0101 0202 0103 0103 0101 0104 0404 0102 0101 0202 0103 0404 0202 0404 0102 0202 0203 0101 0303 0303 0404 0202 0404 0303 0303 0202 0101 0303 0303 0103 0303 0101 0101 0404 0202 0404 0202 0202 0303 0404 0202 0303 0303 0303 0101 0404 0101 0303 0202 0303 0303 0303 0102 0303 0404 0101 0404 0404 0404 0000 0102 0303 0202 0404 0303 0202 0101 0203 0202 0202 0404 0101 0304 0202 0101 0303 0203 0102 0202 0204 0304 0404 0203 0204 0101 0204 0103 0103 0303 0204 0202 0404 0202 0404 0303 0303 0202 0101 0202 0404 0303 0202 0103 0103 0303 0303 0404 0202 0101 0101 0204 0303 0202 0303 0303 0204 0303 0101 0202 0202 0202 0202 0102 0303 0303 0203 0404 0202 0404 0101 0303 0101 0303 0103 0202 0202 0202 0101 0203 0101 0204 0101 0404 0303 0303 0103 0202 0202 0204 0103 0303 0204 0304 0303 0404 0204 0202 0203 0204 0404 0101 0104 0101 0202 0303 0202 0202 0202 0101 0202 0202 0202 0303 0203 0103 0103 0303 0103 0202 0204 0303 0404 0303 0202 0303 0202 0304 0404 0202 0202 0204 0304 0202 0303 0101 0202 0202 0404 0303 0103 0404 0000 0202 0202 0101 0102 0202 0204 0304 0202 0101 0101 0103 0101 0204 0202 0101 0304 0404 0202 0101 0304 0202 0202 0202 0303 0103 0404 0101 0303 0404 0101 0204 0101 0202 0103 0101 0203 0303 0202 0101 0404 0303 0202 0303 0202 0404 0404 0103 0101 0101 0202 0303 0101 0303 0202 0103 0202 0202 0202 0303 0204 0404 0104 0303 0101 0303 0103 0101 0303 0404 0404 0303 0204 0103 0202 0103 0104 0303 0103 0101 0404 0000 0202 0202 0202 0303 0303 0303 0202 0303 0202 0304 0404 0202 0303 0303 0203 0101 0404 0303 0101 0204 0104 0202 0202 0203 0101 0101 0303 0303 0202 0101 0203 0101 0404 0202 0202 0304 0101 0404 0202 0202 0303 0304 0103 0404 0102 0202 0202 0202 0303 0404 0101 0202 0303 0404 0101 0101 0404 0404 0103 0101 0202 0303 0202 0103 0104 0104 0303 0202 0101 0101 0204 0303 0202 0202 0202 0404 0404 0304 0202 0404 0202 0404 0101 0204 0101 0404 0202 0202 0202 0303 0303 0202 0202 0303 0202 0202 0404 0303 0303 0102 0101 0303 0104 0101 0404 0101 0101 0202 0303 0303 0204 0303 0202 0104 0404 0303 0304 0404 0101 0202 0204 0202 0303 0404 0202 0103 0101 0203 0404 0404 0303 0202 0202 0304 0303 0202 0101 0103 0103 0404 0404 0303 0404 0404 0103 0202 0204 0204 0103 0303 0404 0202 0203 0104 0303 0204 0202 0202 0202 0404 0303 0404 0101 0303 0204 0202 0303 0204 0202 0202 0202 0303 0103 0303 0303 0404 0404 0304 0303 0303 0102 0303 0103 0303 0101 0303 0101 0103 0103 0202 0304 0104 0404 0102 0404 0202 0204 0101 0303 0202 0303 0104 0103 0404 0303 0202 0303 0404 0303 0202 0303 0202 0202 0101 0101 0204 0304 0204 0103 0204 0101 0103 0303 0103 0303 0404 0404 0202 0102 0103 0101 0101 0104 0404 0303 0303 0303 0303 0103 0203 0204 0303 0101 0303 0404 0104 0202 0303 0103 0303 0202 0103 0404 0202 0103 0304 0101 0303 0103 0202 0202 0101 0102 0303 0202 0202 0104 0304 0202 0303 0404 0202 0304 0204 0303 0101 0101 0202 0303 0101 0204 0202 0102 0101 0101 0202 0202 0404 0303 0101 0101 0303 0303 0103 0101 0303 0404 0304 0404 0104 0404 0202 0202 0404 0203 0303 0101 0303 0102 0404 0101 0104 0103 0404 0303 0303 0204 0101 0404 0404 0202 0102 0103 0202 0404 0404 0303 0202 0303 0101 0303 0404 0202 0303 0202 0203 0101 0303 0202 0102 0101 0303 0202 0202 0202 0102 0204 0202 0102 0303 0303 0404 0101 0202 0202 0303 0204 0203 0204 0202 0404 0303 0202 0103 0101 0303 0404 0204 0303 0304 0101 0101 0303 0103 0204 0202 0303 0404 0304 0101 0101 0404 0101 0404 0203 0101 0102 0202 0303 0303 0404 0101 0101 0404 0303 0202 0202 0101 0101 0303 0101 0202 0101 0202 0103 0203 0303 0404 0303 0101 0202 0404 0204 0202 0404 0202 0304 0404 0404 0404 0103 0404 0202 0204 0202 0202 0304 0202 0303 0303 0202 0303 0303 0101 0202 0101 0202 0404 0101 0404 0202 0303 0101 0303 0101 0204 0303 0202 0202 0202 0203 0103 0303 0202 0101 0101 0303 0103 0303 0101 0101 0303 0404 0204 0202 0204 0101 0202 0101 0103 0303 0103 0101 0303 0204 0101 0404 0103 0202 0101 0101 0101 0202 0303 0404 0103 0202 0203 0304 0202 0303 0303 0103 0303 0104 0204 0303 0404 0303 0202 0303 0101 0303 0202 0101 0303 0101 0404 0202 0103 0303 0404 0202 0103 0404 0303 0303 0404 0303 0101 0404 0204 0103 0303 0101 0303 0404 0202 0404 0204 0101 0202 0404 0404 0101 0103 0103 0101 0202 0303 0303 0103 0404 0104 0104 0303 0103 0101 0000 0104 0103 0101 0404 0202 0101 0303 0303 0104 0303 0202 0303 0101 0304 0404 0404 0303 0303 0202 0202 0101 0202 0101 0303 0103 0104 0404 0303 0101 0101 0303 0104 0202 0203 0303 0202 0104 0202 0101 0202 0204 0203 0404 0204 0303 0102 0101 0404 0202 0303 0203 0202 0303 0102 0103 0303 0404 0303 0101 0202 0303 0204 0103 0404 0101 0103 0101 0303 0204 0000 0101 0102 0104 0202 0303 0101 0204 0101 0303 0202 0203 0104 0404 0103 0202 0303 0202 0203 0202 0404 0101 0204 0101 0101 0101 0202 0103 0202 0204 0304 0202 0101 0101 0404 0203 0303 0202 0202 0101 0103 0102 0104 0202 0202 0404 0101 0104 0101 0204 0303 0203 0204 0303 0204 0303 0202 0404 0203 0404 0103 0101 0404 0202 0202 0303 0303 0202 0202 0101 0103 0202 0404 0303 0303 0202 0103 0404 0303 0404 0202 0303 0204 0303 0304 0204 0101 0303 0404 0303 0101 0204 0101 0202 0303 0101 0404 0303 0303 0203 0404 0202 0000 0101 0202 0404 0303 0303 0101 0101 0404 0303 0103 0203 0303 0202 0202 0103 0304 0202 0101 0303 0202 0101 0303 0303 0202 0404 0202 0303 0202 0103 0202 0101 0202 0303 0102 0103 0303 0104 0103 0204 0103 0303 0101 0404 0303 0103 0303 0404 0404 0404 0303 0303 0303 0204 0303 0101 0202 0202 0101 0303 0202 0303 0101 0104 0202 0102 0103 0202 0404 0303 0204 0202 0000 0303 0202 0204 0104 0204 0303 0204 0303 0101 0303 0304 0303 0404 0202 0303 0204 0304 0202 0104 0202 0404 0204 0204 0404 0101 0103 0304 0303 0202 0103 0101 0103 0404 0101 0404 0104 0102 0303 0404 0202 0202 0404 0202 0303 0404 0202 0404 0102 0404 0404 0203 0404 0103 0404 0404 0104 0404 0103 0303 0404 0303 0204 0104 0101 0202 0101 0101 0303 0101 0101 0202 0202 0202 0404 0101 0303 0303 0202 0303 0202 0404 0103 0204 0202 0304 0202 0103 0303 0103 0202 0404 0404 0404 0303 0103 0404 0304 0203 0202 0202 0202 0204 0103 0303 0404 0102 0101 0303 0103 0303 0202 0101 0202 0101 0101 0202 0104 0103 0101 0204 0303 0303 0404 0202 0204 0101 0202 0404 0303 0404 0102 0304 0103 0404 0204 0202 0204 0102 0202 0303 0303 0404 0101 0203 0204 0404 0202 0303 0101 0101 0303 0303 0101 0303 0102 0304 0303 0204 0204 0303 0101 0404 0304 0404 0202 0404 0404 0303 0303 0101 0202 0102 0101 0404 0101 0202 0303 0102 0404 0101 0404 0103 0404 0303 0303 0202 0101 0103 0101 0303 0303 0404 0103 0303 0303 0202 0203 0202 0303 0202 0202 0103 0202 0202 0101 0304 0303 0104 0404 0101 0202 0404 0101 0304 0204 0303 0303 0303 0404 0102 0204 0404 0204 0202 0204 0303 0404 0404 0202 0202 0101 0404 0103 0303 0303 0203 0303 0101 0404 0202 0104 0202 0101 0304 0101 0204 0202 0202 0202 0303 0204 0303 0102 0303 0103 0303 0202 0404 0304 0202 0202 0202 0303 0101 0204 0204 0204 0103 0303 0202 0303 0101 0404 0404 0202 0202 0101 0202 0303 0303 0303 0202 0102 0404 0404 0104 0303 0203 0303 0104 0303 0203 0101 0102 0404 0303 0101 0202 0103 0202 0303 0101 0202 0404 0304 0202 0103 0202 0202 0303 0104 0202 0404 0202 0202 0303 0101 0303 0404 0103 0202 0404 0303 0404 0202 0404 0404 0303 0204 0202 0304 0103 0202 0404 0101 0204 0202 0103 0404 0202 0101 0404 0404 0103 0202 0202 0202 0202 0303 0202 0404 0203 0202 0202 0204 0104 0303 0101 0101 0103 0102 0101 0404 0104 0404 0303 0101 0202 0204 0303 0202 0404 0304 0101 0101 0202 0404 0101 0101 0101 0202 0202 0101 0101 0202 0303 0102 0204 0101 0404 0303 0202 0303 0202 0204 0103 0303 0304 0103 0101 0104 0404 0303 0101 0404 0404 0303 0303 0404 0101 0202 0101 0404 0303 0404 0104 0404 0204 0303 0103 0404 0304 0304 0202 0101 0404 0101 0203 0303 0202 0303 0101 0202 0303 0404 0101 0303 0303 0202 0202 0202 0204 0404 0303 0304 0303 0404 0404 0202 0101 0303 0303 0304 0303 0101 0303 0102 0202 0303 0303 0404 0303 0202 0404 0303 0101 0101 0103 0404 0101 0104 0203 0303 0303 0404 0404 0101 0101 0304 0104 0303 0202 0404 0202 0202 0103 0303 0203 0101 0404 0202 0000 0000 0303 0404 0204 0102 0202 0204 0404 0202 0404 0101 0404 0202 0104 0101 0101 0404 0101 0303 0303 0202 0404 0404 0303 0404 0303 0404 0404 0202 0102 0404 0202 0101 0303 0303 0303 0101 0303 0303 0303 0404 0303 0204 0202 0404 0303 0404 0304 0103 0104 0101 0202 0104 0303 0404 0404 0303 0101 0303 0404 0303 0303 0303 0303 0202 0101 0103 0204 0202 0202 0202 0404 0204 0202 0202 0204 0303 0204 0101 0303 0404 0203 0202 0202 0303 0103 0404 0202 0101 0303 0202 0404 0303 0202 0303 0202 0101 0303 0404 0103 0101 0303 0204 0404 0203 0104 0101 0103 0101 0204 0103 0101 0303 0101 0104 0202 0303 0303 0101 0102 0202 0304 0203 0103 0303 0101 0303 0202 0104 0103 0303 0204 0304 0404 0101 0103 0304 0303 0202 0303 0103 0303 0202 0304 0202 0103 0101 0204 0202 0303 0103 0204 0202 0101 0104 0202 0202 0204 0303 0204 0202 0202 0404 0203 0404 0203 0303 0202 0203 0101 0101 0303 0404 0202 0303 0104 0202 0103 0303 0103 0104 0202 0204 0202 0204 0202 0404 0303 0303 0202 0101 0202 0203 0202 0404 0304 0303 0202 0304 0204 0404 0203 0202 0202 0303 0103 0202 0202 0101 0103 0404 0103 0103 0404 0101 0404 0202 0404 0204 0102 0202 0204 0204 0103 0404 0404 0104 0202 0202 0303 0303 0202 0204 0202 0103 0204 0101 0404 0303 0203 0202 0303 0103 0303 0303 0404 0304 0303 0404 0104 0203 0101 0304 0202 0404 0404 0202 0102 0303 0303 0202 0303 0000 0104 0404 0202 0404 0303 0102 0202 0303 0202 0101 0103 0202 0404 0101 0202 0204 0202 0404 0202 0204 0101 0202 0101 0303 0303 0101 0103 0303 0101 0202 0103 0202 0303 0202 0303 0404 0404 0202 0203 0202 0101 0303 0304 0101 0304 0404 0102 0202 0404 0303 0303 0204 0204 0303 0303 0204 0404 0204 0303 0204 0101 0101 0303 0202 0203 0204 0303 0404 0304 0204 0103 0104 0104 0204 0101 0404 0102 0103 0303 0203 0202 0101 0101 0404 0202 0203 0101 0202 0101 0404 0202 0202 0404 0203 0101 0404 0101 0101 0303 0303 0101 0404 0202 0202 0101 0303 0204 0303 0204 0304 0102 0303 0404 0202 0103 0202 0303 0101 0202 0103 0404 0103 0101 0204 0101 0103 0102 0202 0202 0103 0303 0101 0204 0304 0104 0204 0304 0101 0304 0303 0104 0303 0303 0404 0202 0203 0204 0303 0202 0303 0404 0103 0202 0202 0404 0101 0101 0204 0303 0101 0101 0202 0102 0202 0303 0303 0404 0204 0103 0202 0101 0202 0102 0101 0204 0303 0203 0404 0404 0303 0303 0202 0202 0303 0101 0103 0202 0103 0303 0202 0102 0303 0202 0202 0101 0102 0404 0204 0102 0404 0202 0203 0303 0303 0404 0303 0203 0204 0202 0202 0103 0202 0404 0202 0404 0404 0202 0303 0303 0404 0303 0103 0202 0102 0404 0202 0103 0303 0404 0202 0404 0101 0202 0202 0101 0303 0303 0303 0304 0101 0303 0102 0304 0303 0303 0103 0101 0303 0101 0303 0303 0104 0404 0101 0103 0103 0101 0303 0101 0404 0101 0303 0303 0202 0203 0303 0202 0303 0103 0204 0303 0202 0204 0304 0101 0303 0202 0204 0303 0101 0103 0101 0404 0303 0202 0101 0104 0404 0303 0202 0103 0202 0202 0202 0303 0203 0103 0404 0202 0404 0303 0303 0101 0303 0101 0404 0202 0202 0303 0202 0101 0202 0202 0404 0101 0303 0103 0103 0000 0303 0303 0101 0101 0103 0101 0202 0202 0303 0202 0202 0202 0404 0204 0303 0203 0404 0101 0103 0304 0202 0202 0202 0303 0202 0103 0303 0303 0101 0202 0202 0101 0204 0303 0202 0204 0103 0203 0303 0204 0204 0303 0303 0404 0303 0304 0102 0304 0103 0202 0303 0101 0102 0303 0202 0202 0204 0404 0104 0202 0303 0303 0303 0103 0101 0303 0404 0101 0103 0202 0303 0303 0103 0103 0303 0202 0101 0404 0204 0101 0101 0204 0101 0202 0404 0202 0202 0404 0404 0303 0101 0101 0404 0203 0404 0102 0204 0101 0202 0202 0103 0303 0404 0404 0404 0303 0404 0202 0303 0204 0204 0203 0404 0204 0404 0204 0101 0204 0404 0102 0303 0103 0204 0104 0204 0103 0101 0404 0303 0202 0101 0104 0404 0404 0303 0303 0204 0103 0101 0303 0303 0204 0204 0202 0101 0103 0404 0202 0202 0303 0203 0303 0202 0202 0101 0202 0202 0103 0102 0101 0104 0404 0303 0303 0303 0303 0303 0101 0303 0304 0303 0304 0404 0303 0303 0202 0101 0202 0303 0202 0404 0202 0303 0103 0303 0404 0303 0404 0303 0202 0303 0303 0404 0103 0303 0203 0101 0303 0202 0101 0404 0101 0303 0404 0404 0404 0303 0404 0202 0303 0303 0101 0303 0202 0202 0203 0303 0101 0202 0101 0203 0102 0404 0404 0103 0404 0303 0202 0000 0303 0202 0202 0303 0404 0202 0202 0404 0101 0202 0101 0202 0303 0202 0204 0202 0101 0202 0303 0202 0202 0101 0404 0304 0202 0303 0303 0101 0202 0202 0202 0404 0202 0404 0303 0103 0303 0103 0103 0202 0104 0303 0303 0101 0203 0303 0101 0202 0204 0202 0104 0303 0404 0303 0103 0404 0103 0101 0202 0202 0202 0204 0202 0101 0101 0303 0101 0101 0202 0103 0202 0102 0204 0202 0202 0103 0101 0303 0303 0103 0102 0202 0202 0204 0303 0303 0404 0204 0102 0303 0303 0204 0303 0202 0204 0404 0303 0202 0404 0104 0202 0303 0303 0101 0404 0204 0101 0204 0202 0303 0104 0103 0304 0202 0104 0202 0303 0204 0104 0303 0202 0204 0101 0303 0404 0103 0202 0303 0101 0101 0203 0103 0104 0104 0303 0303 0101 0202 0103 0202 0404 0202 0202 0303 0303 0101 0101 0101 0303 0202 0202 0404 0303 0204 0103 0404 0303 0103 0404 0303 0304 0101 0101 0304 0404 0101 0204 0404 0404 0303 0202 0404 0202 0204 0103 0404 0202 0304 0404 0101 0202 0204 0404 0101 0404 0101 0202 0404 0202 0303 0204 0101 0303 0101 0404 0204 0204 0104 0101 0404 0101 0202 0103 0303 0202 0202 0203 0104 0404 0404 0103 0404 0102 0404 0303 0404 0101 0203 0303 0101 0303 0101 0202 0101 0303 0202 0304 0303 0103 0202 0204 0303 0303 0202 0303 0202 0103 0103 0103 0202 0101 0104 0304 0203 0303 0404 0404 0404 0101 0303 0204 0303 0303 0303 0104 0000 0102 0304 0202 0404 0101 0103 0101 0202 0202 0102 0303 0104 0101 0404 0303 0202 0202 0202 0102 0103 0303 0202 0303 0104 0103 0101 0104 0204 0203 0204 0303 0202 0202 0103 0404 0101 0202 0303 0204 0404 0104 0103 0202 0202 0202 0303 0202 0303 0404 0102 0303 0101 0103 0102 0101 0202 0404 0304 0103 0404 0303 0202 0101 0303 0202 0204 0103 0404 0303 0303 0303 0102 0202 0101 0103 0404 0104 0303 0202 0303 0303 0101 0202 0404 0103 0303 0303 0303 0104 0303 0103 0204 0202 0303 0101 0304 0202 0303 0304 0303 0202 0103 0404 0303 0303 0303 0303 0103 0303 0101 0202 0203 0103 0303 0101 0202 0404 0202 0101 0404 0303 0101 0404 0404 0101 0303 0303 0303 0202 0303 0202 0404 0303 0202 0202 0404 0202 0101 0103 0202 0102 0202 0404 0202 0103 0303 0404 0101 0404 0404 0202 0303 0103 0101 0101 0101 0202 0103 0101 0202 0202 0202 0202 0103 0404 0101 0404 0101 0404 0101 0404 0303 0102 0202 0303 0404 0202 0404 0202 0101 0202 0202 0102 0303 0404 0204 0203 0202 0404 0204 0404 0202 0202 0404 0202 0104 0000 0303 0203 0202 0204 0304 0303 0303 0204 0202 0304 0303 0103 0204 0101 0202 0202 0104 0404 0404 0303 0404 0304 0101 0202 0103 0202 0202 0202 0204 0202 0303 0404 0102 0303 0202 0103 0101 0202 0303 0204 0202 0101 0303 0101 0303 0104 0103 0202 0101 0204 0204 0202 0104 0202 0101 0404 0202 0202 0101 0103 0303 0101 0101 0404 0303 0404 0404 0303 0303 0303 0404 0101 0202 0204 0303 0101 0202 0101 0103 0204 0303 0104 0303 0202 0202 0404 0303 0202 0204 0202 0202 0303 0404 0103 0203 0202 0404 0202 0101 0103 0303 0202 0101 0101 0202 0303 0303 0203 0303 0101 0202 0202 0202 0304 0303 0303 0103 0202 0103 0204 0202 0203 0202 0404 0303 0404 0101 0103 0102 0103 0404 0303 0404 0203 0303 0104 0103 0304 0303 0303 0404 0104 0303 0101 0202 0304 0101 0404 0101 0404 0404 0203 0103 0103 0104 0404 0404 0202 0101 0303 0303 0101 0404 0203 0202 0303 0304 0103 0303 0101 0202 0404 0303 0202 0404 0404 0404 0103 0102 0404 0202 0203 0202 0204 0101 0103 0404 0303 0202 0303 0202 0303 0202 0000 0101 0202 0101 0404 0304 0303 0404 0202 0303 0202 0404 0404 0103 0101 0303 0202 0303 0404 0202 0202 0303 0202 0303 0202 0303 0101 0101 0203 0404 0101 0204 0103 0202 0101 0304 0303 0103 0404 0101 0204 0103 0202 0404 0202 0204 0103 0404 0204 0202 0103 0101 0101 0101 0303 0101 0101 0404 0303 0202 0303 0303 0203 0203 0303 0202 0404 0102 0303 0202 0303 0101 0103 0103 0303 0404 0103 0204 0204 0303 0303 0304 0202 0303 0103 0303 0202 0304 0101 0404 0202 0102 0103 0202 0404 0101 0000 0101 0103 0303 0303 0404 0404 0303 0404 0101 0101 0202 0202 0303 0303 0204 0404 0103 0303 0202 0203 0202 0404 0101 0103 0202 0103 0103 0204 0404 0404 0404 0404 0103 0101 0101 0204 0303 0204 0404 0204 0102 0202 0404 0303 0103 0404 0101 0202 0202 0202 0101 0204 0303 0303 0202 0102 0202 0103 0103 0104 0303 0303 0303 0202 0404 0202 0303 0404 0404 0202 0404 0404 0101 0303 0101 0303 0202 0303 0202 0202 0404 0202 0101 0101 0204 0202 0101 0303 0404 0202 0202 0101 0303 0103 0303 0404 0204 0202 0303 0104 0404 0103 0101 0104 0202 0404 0203 0204 0404 0202 0404 0104 0204 0103 0204 0101 0103 0303 0104 0303 0304 0404 0202 0303 0202 0304 0203 0303 0202 0101 0203 0101 0101 0101 0204 0404 0204 0404 0101 0404 0202 0204 0303 0303 0202 0101 0101 0202 0303 0303 0202 0303 0101 0101 0204 0101 0202 0204 0101 0104 0102 0404 0202 0103 0202 0204 0303 0404 0303 0404 0101 0404 0304 0101 0101 0101 0202 0204 0102 0101 0303 0202 0101 0404 0104 0203 0101 0101 0303 0404 0303 0303 0204 0102 0404 0202 0202 0303 0303 0303 0404 0202 0101 0303 0303 0303 0303 0303 0103 0101 0404 0303 0101 0304 0101 0404 0202 0101 0404 0101 0204 0202 0404 0303 0202 0202 0303 0202 0202 0303 0404 0303 0203 0103 0103 0303 0202 0202 0303 0404 0404 0404 0404 0404 0202 0202 0101 0303 0101 0404 0404 0101 0101 0202 0303 0103 0202 0303 0404 0303 0102 0102 0404 0101 0404 0202 0404 0204 0202 0303 0202 0303 0202 0101 0204 0101 0101 0303 0101 0303 0202 0202 0404 0101 0303 0404 0303 0404 0202 0101 0404 0101 0303 0404 0101 0404 0404 0303 0404 0102 0104 0303 0101 0202 0102 0404 0101 0102 0303 0202 0303 0103 0104 0404 0203 0303 0303 0404 0102 0303 0202 0202 0303 0304 0103 0303 0202 0202 0101 0202 0104 0303 0404 0303 0203 0303 0404 0102 0202 0101 0202 0404 0103 0303 0102 0303 0202 0202 0202 0303 0303 0404 0404 0404 0101 0304 0204 0303 0101 0303 0101 0101 0202 0202 0202 0304 0203 0101 0202 0404 0202 0101 0202 0303 0404 0101 0303 0104 0103 0404 0404 0101 0303 0404 0204 0202 0101 0203 0202 0404 0404 0303 0303 0404 0202 0303 0404 0303 0203 0101 0404 0104 0303 0202 0202 0202 0101 0101 0303 0103 0202 0103 0303 0204 0104 0202 0303 0101 0202 0101 0202 0303 0303 0404 0204 0104 0404 0202 0101 0102 0202 0404 0303 0304 0204 0102 0202 0303 0204 0102 0404 0303 0202 0303 0203 0101 0303 0104 0202 0303 0204 0202 0303 0102 0303 0404 0202 0303 0304 0404 0304 0103 0404 0104 0303 0101 0303 0404 0101 0202 0404 0101 0304 0303 0303 0404 0103 0202 0303 0204 0404 0204 0202 0101 0101 0404 0101 0404 0202 0202 0404 0303 0303 0404 0101 0404 0202 0101 0101 0202 0103 0202 0202 0303 0101 0202 0204 0103 0104 0202 0404 0202 0104 0202 0000 0101 0404 0202 0204 0404 0303 0104 0103 0304 0303 0303 0104 0303 0202 0104 0304 0303 0101 0203 0204 0202 0101 0303 0203 0303 0202 0102 0303 0103 0204 0103 0202 0202 0204 0304 0404 0202 0202 0204 0202 0101 0303 0202 0202 0303 0103 0202 0303 0203 0404 0101 0202 0202 0404 0202 0102 0303 0404 0303 0303 0103 0303 0304 0303 0103 0202 0303 0202 0101 0202 0104 0101 0202 0202 0202 0404 0101 0303 0101 0303 0204 0204 0404 0101 0104 0303 0202 0404 0101 0303 0303 0202 0202 0204 0203 0202 0202 0303 0204 0101 0304 0404 0204 0204 0303 0104 0204 0101 0404 0203 0102 0404 0303 0303 0202 0203 0202 0303 0202 0303 0202 0102 0202 0101 0202 0203 0404 0101 0202 0404 0303 0202 0202 0101 0304 0303 0303 0303 0404 0303 0202 0204 0303 0102 0303 0303 0303 0202 0202 0404 0101 0404 0404 0101 0404 0202 0101 0101 0202 0204 0101 0404 0404 0101 0202 0202 0203 0303 0303 0303 0101 0101 0101 0204 0303 0101 0101 0303 0101 0204 0204 0303 0404 0101 0102 0303 0204 0103 0202 0303 0202 0102 0202 0104 0204 0204 0303 0103 0101 0202 0202 0202 0404 0303 0202 0202 0404 0303 0103 0202 0202 0103 0303 0303 0202 0303 0304 0103 0101 0101 0303 0101 0103 0202 0204 0303 0303 0202 0303 0304 0404 0101 0101 0404 0101 0202 0104 0303 0101 0202 0303 0202 0202 0103 0202 0202 0404 0104 0101 0101 0101 0303 0101 0101 0303 0101 0202 0202 0404 0304 0102 0102 0203 0101 0101 0202 0101 0202 0204 0203 0404 0202 0204 0202 0101 0404 0202 0404 0303 0404 0303 0202 0103 0303 0404 0404 0103 0101 0202 0102 0303 0303 0102 0204 0202 0303 0404 0303 0202 0303 0202 0204 0304 0202 0204 0101 0204 0303 0303 0404 0202 0404 0101 0204 0303 0404 0303 0404 0101 0101 0203 0101 0204 0103 0303 0204 0404 0303 0202 0102 0404 0102 0404 0103 0303 0202 0102 0103 0103 0101 0202 0404 0202 0404 0303 0202 0202 0303 0101 0202 0101 0303 0104 0202 0303 0303 0203 0101 0202 0202 0104 0202 0202 0303 0404 0303 0304 0303 0103 0303 0203 0101 0103 0202 0202 0101 0202 0303 0103 0404 0303 0103 0204 0204 0202 0303 0104 0101 0303 0103 0304 0303 0101 0103 0103 0202 0101 0304 0404 0101 0101 0202 0204 0101 0204 0303 0404 0203 0204 0202 0303 0101 0101 0204 0404 0404 0101 0101 0404 0202 0204 0404 0102 0304 0202 0404 0304 0404 0404 0101 0404 0202 0102 0304 0104 0102 0103 0202 0101 0101 0202 0404 0404 0101 0204 0203 0303 0303 0202 0202 0303 0303 0404 0203 0101 0204 0303 0202 0303 0404 0203 0202 0303 0203 0204 0404 0103 0101 0404 0202 0202 0202 0203 0202 0304 0303 0404 0303 0101 0101 0303 0404 0101 0101 0303 0202 0303 0303 0303 0202 0101 0204 0303 0404 0303 0202 0303 0303 0203 0303 0404 0101 0404 0303 0404 0103 0202 0303 0404 0102 0203 0303 0101 0404 0204 0101 0202 0303 0202 0202 0303 0202 0102 0202 0103 0101 0202 0303 0202 0102 0202 0202 0104 0101 0203 0204 0303 0102 0202 0203 0202 0204 0204 0303 0404 0202 0202 0404 0303 0101 0303 0303 0101 0101 0101 0102 0101 0204 0303 0101 0202 0101 0303 0202 0101 0104 0204 0204 0303 0404 0103 0204 0303 0303 0404 0202 0202 0202 0303 0101 0404 0202 0202 0202 0404 0202 0202 0404 0101 0102 0404 0404 0202 0404 0202 0104 0304 0303 0101 0204 0204 0303 0304 0101 0202 0202 0404 0101 0202 0303 0303 0101 0303 0404 0104 0103 0103 0303 0103 0202 0101 0303 0102 0404 0303 0404 0303 0303 0303 0202 0404 0303 0101 0101 0202 0303 0202 0202 0303 0202 0303 0202 0104 0104 0202 0202 0303 0101 0103 0101 0303 0404 0104 0202 0404 0203 0303 0101 0303 0103 0101 0202 0303 0404 0202 0204 0202 0202 0101 0204 0303 0303 0101 0101 0101 0202 0404 0404 0202 0303 0404 0102 0103 0202 0304 0204 0303 0303 0000 0404 0404 0101 0404 0202 0104 0303 0202 0204 0304 0202 0101 0101 0404 0202 0101 0303 0303 0203 0101 0204 0101 0000 0303 0303 0303 0303 0202 0404 0202 0404 0404 0303 0303 0101 0202 0404 0203 0202 0404 0202 0404 0104 0202 0202 0303 0304 0304 0204 0103 0203 0103 0404 0303 0202 0303 0303 0202 0303 0404 0101 0101 0101 0303 0303 0101 0404 0202 0202 0104 0404 0303 0202 0101 0101 0104 0202 0103 0202 0404 0103 0303 0303 0303 0202 0101 0202 0202 0303 0101 0404 0204 0202 0303 0404 0101 0101 0303 0404 0202 0303 0204 0202 0202 0204 0103 0303 0404 0404 0202 0404 0101 0404 0203 0101 0303 0101 0303 0404 0101 0404 0304 0404 0101 0303 0404 0404 0404 0303 0203 0303 0303 0404 0101 0404 0303 0304 0103 0101 0303 0202 0404 0404 0101 0202 0202 0303 0404 0202 0303 0202 0101 0103 0203 0102 0404 0303 0303 0303 0101 0404 0101 0204 0204 0404 0101 0101 0404 0404 0204 0103 0404 0303 0101 0101 0104 0101 0102 0404 0204 0204 0204 0404 0202 0204 0303 0404 0404 0101 0204 0304 0202 0303 0202 0101 0202 0404 0104 0102 0102 0303 0104 0303 0404 0303 0303 0102 0303 0202 0204 0202 0202 0202 0202 0202 0101 0101 0101 0101 0303 0101 0404 0103 0101 0204 0303 0103 0303 0101 0104 0202 0102 0202 0101 0303 0202 0101 0202 0202 0303 0000 0101 0101 0202 0101 0204 0404 0404 0101 0303 0303 0101 0204 0202 0204 0404 0303 0404 0202 0103 0104 0103 0202 0101 0404 0203 0202 0203 0303 0101 0101 0104 0202 0104 0303 0103 0202 0202 0202 0202 0304 0203 0202 0204 0202 0404 0101 0000 0103 0303 0303 0303 0204 0101 0103 0202 0103 0101 0101 0101 0104 0304 0303 0202 0404 0202 0202 0404 0202 0303 0104 0101 0304 0103 0101 0204 0202 0304 0101 0102 0404 0404 0204 0202 0303 0104 0202 0304 0204 0101 0202 0202 0202 0101 0202 0303 0204 0203 0303 0303 0404 0202 0101 0101 0303 0102 0202 0303 0101 0101 0202 0202 0202 0303 0204 0204 0101 0303 0303 0101 0404 0303 0204 0303 0204 0204 0203 0204 0203 0303 0103 0202 0404 0204 0202 0101 0203 0303 0202 0101 0404 0101 0202 0101 0101 0101 0202 0303 0303 0203 0202 0101 0202 0304 0103 0101 0404 0103 0101 0202 0202 0204 0103 0102 0102 0101 0304 0103 0101 0404 0303 0202 0404 0202 0303 0303 0104 0404 0303 0303 0202 0404 0303 0303 0104 0303 0101 0202 0303 0101 0103 0103 0303 0202 0404 0303 0202 0404 0303 0202 0101 0303 0202 0202 0404 0101 0101 0404 0103 0101 0303 0303 0303 0101 0202 0202 0202 0104 0303 0202 0202 0303 0404 0202 0101 0303 0303 0102 0202 0303 0202 0202 0404 0303 0202 0404 0303 0202 0303 0203 0101 0101 0103 0303 0304 0404 0304 0303 0204 0102 0202 0102 0202 0101 0103 0203 0404 0304 0303 0202 0303 0303 0304 0000 0202 0103 0404 0202 0303 0404 0404 0303 0204 0102 0202 0202 0101 0303 0101 0303 0101 0202 0404 0101 0404 0303 0303 0101 0202 0203 0204 0202 0103 0303 0404 0404 0202 0204 0103 0104 0103 0101 0202 0000 0103 0202 0204 0103 0101 0101 0404 0404 0303 0204 0304 0202 0303 0202 0204 0101 0202 0303 0204 0404 0303 0303 0202 0303 0204 0304 0304 0303 0203 0404 0202 0101 0303 0104 0404 0202 0104 0404 0102 0202 0202 0103 0303 0404 0204 0203 0303 0204 0303 0404 0202 0303 0101 0404 0303 0303 0101 0204 0103 0103 0303 0303 0202 0304 0204 0202 0204 0202 0101 0202 0104 0101 0202 0303 0303 0303 0202 0303 0303 0404 0101 0304 0202 0303 0404 0404 0404 0303 0304 0103 0101 0204 0202 0101 0202 0404 0303 0101 0202 0202 0202 0101 0202 0101 0404 0102 0404 0404 0303 0202 0303 0303 0202 0204 0304 0303 0202 0404 0103 0203 0404 0202 0404 0202 0202 0202 0404 0102 0101 0103 0303 0202 0202 0404 0404 0204 0101 0202 0101 0303 0103 0103 0204 0404 0404 0303 0303 0203 0404 0101 0303 0303 0303 0202 0404 0202 0101 0101 0204 0202 0202 0304 0204 0202 0303 0202 0404 0303 0202 0202 0303 0101 0103 0104 0202 0103 0202 0303 0103 0202 0303 0303 0101 0303 0304 0303 0303 0101 0202 0202 0404 0101 0202 0202 0103 0104 0204 0101 0204 0202 0202 0303 0303 0102 0404 0103 0303 0404 0202 0303 0204 0101 0101 0202 0101 0304 0204 0404 0000 0303 0404 0404 0303 0202 0404 0202 0103 0101 0202 0104 0101 0103 0303 0101 0101 0303 0202 0202 0202 0101 0303 0404 0202 0203 0304 0404 0303 0303 0202 0102 0203 0304 0202 0202 0000 0202 0202 0204 0404 0101 0204 0303 0202 0203 0404 0101 0202 0101 0303 0202 0404 0303 0101 0000 0104 0202 0202 0204 0203 0303 0101 0103 0101 0202 0303 0202 0202 0202 0202 0304 0202 0202 0202 0202 0303 0304 0303 0202 0303 0303 0203 0303 0204 0404 0404 0101 0404 0202 0203 0103 0303 0204 0203 0101 0103 0303 0204 0303 0101 0203 0404 0303 0303 0404 0304 0303 0204 0202 0303 0101 0404 0202 0303 0303 0101 0404 0204 0202 0303 0101 0203 0303 0202 0303 0404 0203 0303 0303 0000 0404 0303 0104 0404 0204 0404 0202 0103 0202 0303 0103 0404 0303 0304 0103 0104 0101 0202 0204 0404 0202 0303 0101 0303 0303 0404 0303 0303 0103 0303 0102 0304 0404 0202 0303 0101 0404 0101 0204 0204 0304 0202 0303 0202 0303 0103 0101 0404 0202 0101 0303 0202 0101 0404 0103 0101 0303 0202 0303 0101 0101 0303 0303 0204 0202 0304 0101 0101 0204 0104 0102 0303 0404 0202 0103 0303 0303 0304 0101 0202 0101 0404 0404 0202 0202 0303 0202 0303 0303 0303 0103 0202 0103 0101 0404 0303 0101 0202 0202 0303 0202 0202 0202 0101 0303 0304 0104 0202 0303 0404 0303 0404 0204 0303 0404 0303 0304 0303 0404 0202 0103 0202 0102 0101 0303 0303 0202 0101 0404 0101 0303 0202 0103 0404 0303 0101 0101 0101 0202 0101 0303 0202 0404 0202 0404 0303 0404 0101 0202 0303 0304 0404 0202 0202 0204 0103 0202 0202 0303 0303 0202 0303 0303 0303 0101 0404 0202 0202 0303 0202 0202 0204 0101 0404 0101 0404 0303 0202 0202 0404 0404 0303 0101 0303 0101 0202 0404 0104 0404 0202 0303 0202 0303 0404 0102 0202 0101 0303 0202 0101 0202 0404 0101 0404 0101 0202 0202 0101 0101 0404 0202 0404 0101 0202 0303 0303 0103 0404 0202 0101 0204 0303 0404 0404 0304 0104 0204 0202 0204 0303 0101 0101 0303 0204 0103 0101 0204 0202 0404 0103 0204 0303 0303 0101 0202 0404 0102 0103 0303 0202 0204 0101 0202 0404 0101 0104 0101 0102 0101 0404 0303 0202 0204 0101 0103 0303 0202 0101 0102 0101 0202 0104 0303 0404 0404 0202 0303 0303 0303 0101 0202 0202 0202 0204 0101 0102 0103 0101 0303 0202 0304 0202 0202 0103 0202 0404 0202 0303 0404 0404 0103 0303 0202 0304 0404 0404 0404 0303 0104 0102 0202 0202 0202 0303 0203 0202 0303 0101 0101 0202 0101 0103 0204 0404 0404 0202 0103 0303 0202 0202 0102 0304 0202 0404 0303 0404 0103 0202 0202 0303 0303 0303 0202 0303 0404 0404 0202 0101 0101 0404 0102 0101 0204 0303 0404 0204 0104 0000 0104 0202 0202 0303 0404 0202 0303 0202 0404 0103 0404 0101 0202 0304 0101 0303 0203 0303 0102 0303 0404 0303 0203 0304 0202 0204 0202 0101 0103 0404 0303 0303 0204 0404 0303 0103 0303 0304 0303 0104 0101 0303 0303 0204 0204 0104 0202 0303 0404 0404 0103 0303 0103 0303 0303 0202 0103 0303 0404 0303 0101 0202 0101 0404 0303 0101 0304 0404 0203 0203 0303 0303 0404 0102 0202 0202 0101 0101 0103 0202 0204 0103 0404 0303 0404 0202 0303 0204 0303 0303 0404 0101 0101 0303 0204 0101 0202 0303 0204 0303 0304 0404 0102 0303 0204 0202 0303 0303 0303 0202 0202 0101 0404 0203 0404 0101 0202 0103 0303 0101 0101 0103 0303 0202 0202 0304 0202 0104 0404 0104 0303 0303 0303 0303 0202 0101 0303 0102 0202 0202 0404 0303 0103 0303 0202 0203 0404 0202 0202 0404 0202 0303 0204 0202 0103 0303 0404 0404 0202 0104 0202 0101 0303 0104 0303 0202 0101 0103 0202 0202 0404 0202 0102 0204 0101 0101 0204 0101 0404 0102 0103 0304 0303 0202 0202 0202 0404 0303 0202 0103 0203 0303 0101 0404 0101 0204 0303 0101 0404 0101 0303 0404 0202 0202 0303 0101 0202 0101 0303 0404 0202 0103 0202 0101 0202 0101 0101 0303 0202 0202 0404 0202 0202 0202 0202 0104 0102 0202 0204 0103 0303 0404 0202 0404 0202 0103 0404 0104 0101 0202 0202 0101 0202 0303 0103 0202 0303 0204 0103 0404 0303 0404 0404 0202 0101 0103 0202 0101 0304 0303 0303 0303 0303 0404 0103 0101 0303 0104 0104 0103 0101 0303 0303 0204 0101 0404 0202 0101 0303 0202 0101 0303 0202 0202 0203 0303 0103 0103 0404 0303 0303 0101 0203 0101 0202 0404 0101 0303 0101 0404 0303 0101 0101 0404 0202 0404 0103 0204 0203 0101 0000 0202 0303 0102 0202 0203 0102 0101 0303 0101 0202 0203 0404 0202 0202 0101 0404 0102 0103 0202 0404 0303 0203 0303 0303 0101 0202 0304 0203 0404 0303 0303 0202 0202 0202 0303 0103 0104 0303 0202 0101 0404 0101 0103 0204 0204 0303 0101 0202 0204 0202 0404 0102 0303 0103 0101 0404 0303 0202 0303 0202 0204 0404 0101 0101 0101 0101 0303 0303 0104 0202 0303 0101 0103 0202 0101 0304 0303 0104 0204 0303 0202 0202 0203 0404 0303 0303 0101 0404 0303 0303 0202 0303 0101 0404 0303 0303 0101 0304 0404 0304 0303 0404 0304 0303 0102 0102 0303 0101 0101 0101 0202 0204 0303 0101 0404 0101 0103 0203 0204 0204 0102 0202 0101 0204 0202 0202 0404 0103 0202 0202 0204 0203 0202 0204 0101 0202 0101 0102 0404 0404 0103 0303 0202 0303 0404 0303 0404 0101 0103 0101 0202 0202 0103 0304 0202 0303 0303 0202 0101 0101 0303 0101 0404 0101 0204 0204 0204 0303 0203 0202 0202 0202 0303 0102 0103 0101 0303 0202 0303 0303 0303 0202 0303 0103 0202 0101 0404 0202 0204 0101 0404 0303 0204 0202 0404 0204 0202 0103 0202 0404 0303 0404 0303 0202 0203 0303 0202 0101 0303 0303 0202 0204 0404 0101 0303 0404 0303 0101 0404 0202 0303 0203 0303 0203 0303 0202 0101 0101 0404 0404 0303 0303 0202 0204 0404 0303 0101 0304 0202 0204 0202 0202 0103 0101 0202 0202 0101 0202 0404 0303 0404 0101 0404 0103 0303 0303 0101 0101 0101 0303 0404 0202 0101 0303 0203 0102 0204 0101 0204 0303 0101 0203 0202 0101 0202 0303 0204 0204 0303 0202 0204 0204 0303 0303 0202 0202 0101 0304 0303 0404 0303 0101 0202 0202 0202 0303 0404 0404 0404 0404 0404 0303 0204 0103 0303 0202 0202 0103 0101 0202 0303 0101 0204 0303 0202 0304 0404 0404 0404 0204 0303 0303 0303 0303 0404 0103 0204 0202 0404 0303 0202 0404 0404 0303 0303 0204 0304 0102 0303 0202 0202 0303 0101 0404 0104 0101 0202 0303 0303 0404 0303 0202 0404 0101 0101 0303 0202 0202 0202 0303 0202 0303 0303 0101 0102 0203 0202 0202 0101 0104 0303 0202 0204 0101 0303 0101 0404 0404 0104 0303 0404 0101 0303 0404 0304 0104 0204 0101 0202 0202 0404 0202 0101 0204 0303 0204 0303 0103 0202 0203 0404 0303 0204 0202 0202 0103 0202 0202 0204 0202 0204 0404 0404 0303 0304 0203 0303 0202 0101 0202 0000 0202 0303 0202 0404 0303 0202 0303 0303 0101 0303 0404 0202 0204 0303 0202 0303 0204 0101 0102 0404 0102 0102 0103 0404 0404 0202 0103 0303 0303 0303 0203 0304 0101 0202 0202 0101 0404 0303 0203 0404 0203 0101 0303 0303 0404 0204 0404 0202 0404 0101 0303 0103 0202 0303 0204 0101 0101 0202 0303 0202 0202 0303 0202 0202 0202 0103 0303 0303 0303 0202 0101 0303 0202 0202 0101 0203 0202 0202 0204 0104 0204 0202 0101 0202 0202 0101 0103 0303 0404 0202 0103 0101 0202 0204 0303 0103 0103 0202 0202 0101 0404 0404 0101 0104 0101 0101 0202 0204 0303 0102 0000 0102 0202 0202 0404 0101 0103 0404 0101 0303 0000 0303 0202 0101 0303 0204 0101 0202 0404 0303 0303 0202 0303 0202 0103 0202 0202 0103 0103 0103 0202 0404 0303 0303 0404 0303 0303 0202 0303 0103 0204 0304 0202 0303 0303 0303 0101 0104 0202 0104 0204 0204 0104 0202 0304 0303 0101 0204 0101 0101 0101 0101 0101 0204 0404 0000 0103 0404 0303 0101 0202 0303 0203 0202 0101 0104 0202 0101 0203 0303 0303 0303 0202 0204 0103 0303 0202 0204 0404 0101 0303 0404 0303 0101 0101 0404 0404 0103 0104 0404 0404 0101 0303 0303 0304 0101 0303 0404 0202 0202 0101 0101 0101 0202 0202 0202 0202 0303 0303 0404 0404 0202 0404 0101 0404 0104 0303 0303 0203 0303 0303 0303 0404 0203 0101 0102 0303 0303 0303 0204 0101 0102 0404 0202 0303 0404 0101 0303 0303 0104 0303 0202 0101 0303 0303 0303 0202 0102 0202 0303 0101 0202 0404 0303 0202 0103 0303 0101 0404 0103 0303 0204 0202 0303 0204 0203 0101 0202 0101 0202 0303 0102 0101 0202 0204 0102 0101 0103 0204 0101 0303 0303 0303 0204 0404 0103 0202 0202 0202 0202 0101 0202 0303 0202 0202 0303 0102 0101 0101 0303 0404 0204 0101 0103 0101 0303 0303 0303 0303 0304 0203 0303 0202 0404 0303 0404 0303 0202 0101 0303 0103 0101 0202 0404 0202 0204 0303 0203 0303 0202 0202 0404 0203 0202 0404 0101 0404 0202 0202 0202 0303 0303 0202 0101 0404 0303 0404 0404 0204 0404 0202 0303 0202 0101 0303 0202 0303 0202 0404 0303 0303 0101 0404 0303 0202 0202 0101 0203 0404 0204 0303 0103 0303 0303 0101 0303 0404 0202 0303 0303 0303 0102 0404 0303 0303 0203 0404 0101 0303 0404 0202 0204 0202 0102 0404 0202 0202 0204 0204 0101 0102 0104 0101 0303 0404 0202 0404 0303 0202 0103 0202 0202 0202 0103 0204 0304 0103 0304 0303 0203 0102 0303 0303 0103 0103 0101 0303 0303 0102 0101 0101 0202 0103 0303 0303 0204 0303 0202 0303 0203 0303 0102 0202 0303 0101 0303 0102 0204 0303 0103 0202 0202 0202 0204 0404 0101 0101 0202 0202 0304 0404 0204 0101 0202 0101 0303 0303 0101 0304 0404 0202 0303 0101 0101 0303 0103 0202 0202 0204 0304 0204 0202 0204 0202 0304 0303 0101 0303 0303 0202 0204 0204 0202 0202 0204 0303 0203 0103 0101 0202 0101 0101 0101 0103 0303 0102 0202 0101 0103 0202 0202 0101 0303 0202 0101 0204 0303 0102 0404 0303 0204 0101 0404 0202 0202 0202 0101 0203 0101 0101 0204 0202 0202 0303 0202 0202 0202 0303 0303 0203 0404 0202 0303 0102 0202 0202 0404 0101 0101 0303 0303 0202 0404 0303 0303 0204 0101 0404 0404 0202 0303 0101 0303 0404 0303 0104 0202 0101 0404 0104 0404 0101 0202 0101 0404 0202 0303 0202 0404 0101 0202 0101 0204 0202 0303 0404 0303 0101 0303 0303 0404 0202 0304 0303 0101 0101 0101 0204 0202 0204 0202 0202 0303 0304 0101 0101 0303 0303 0404 0202 0202 0404 0202 0202 0303 0104 0404 0303 0202 0303 0204 0404 0203 0303 0303 0404 0303 0204 0303 0202 0202 0203 0103 0202 0303 0101 0202 0104 0303 0202 0303 0202 0204 0103 0103 0404 0101 0404 0303 0303 0303 0404 0303 0202 0101 0104 0303 0202 0404 0404 0103 0303 0202 0404 0303 0404 0204 0202 0101 0204 0101 0303 0101 0204 0101 0404 0101 0303 0101 0404 0204 0303 0303 0303 0101 0103 0102 0404 0404 0304 0303 0101 0304 0103 0204 0101 0101 0203 0204 0404 0202 0303 0202 0303 0304 0303 0404 0404 0101 0202 0303 0203 0303 0404 0101 0304 0203 0202 0204 0303 0202 0303 0204 0203 0101 0404 0404 0102 0204 0202 0303 0303 0404 0404 0404 0202 0101 0202 0101 0202 0404 0101 0303 0404 0303 0202 0202 0202 0303 0404 0303 0404 0303 0303 0202 0202 0303 0304 0404 0404 0204 0202 0203 0202 0303 0303 0204 0303 0404 0101 0202 0303 0404 0303 0202 0202 0204 0101 0202 0202 0303 0202 0101 0101 0204 0101 0101 0103 0303 0102 0202 0202 0202 0204 0303 0303 0303 0202 0404 0101 0404 0202 0202 0202 0303 0103 0101 0104 0303 0202 0303 0101 0101 0101 0303 0101 0202 0404 0101 0404 0000 0404 0303 0404 0303 0303 0303 0303 0103 0303 0202 0101 0202 0202 0204 0202 0303 0404 0404 0103 0102 0404 0102 0303 0202 0404 0404 0204 0101 0303 0404 0202 0202 0404 0404 0404 0202 0404 0101 0204 0303 0202 0203 0101 0103 0204 0102 0303 0103 0101 0303 0101 0202 0202 0102 0101 0404 0102 0202 0102 0104 0202 0101 0303 0303 0202 0202 0203 0303 0303 0103 0404 0101 0202 0303 0202 0303 0203 0404 0303 0202 0101 0204 0101 0204 0303 0204 0303 0101 0303 0404 0202 0000 0202 0101 0202 0202 0202 0101 0303 0103 0101 0202 0101 0202 0202 0103 0404 0204 0103 0000 0101 0303 0101 0101 0102 0404 0202 0404 0204 0101 0404 0202 0202 0303 0202 0202 0404 0303 0303 0204 0303 0101 0202 0304 0104 0101 0202 0102 0103 0103 0104 0303 0104 0303 0304 0101 0303 0103 0202 0404 0103 0404 0202 0104 0104 0404 0104 0404 0103 0404 0404 0303 0404 0101 0101 0101 0102 0303 0404 0303 0303 0303 0303 0404 0204 0303 0202 0101 0204 0204 0204 0303 0103 0204 0303 0204 0404 0101 0303 0303 0303 0303 0101 0104 0101 0304 0104 0202 0104 0202 0303 0404 0202 0101 0202 0303 0404 0101 0202 0101 0303 0303 0303 0203 0202 0204 0102 0202 0303 0202 0303 0303 0204 0404 0101 0404 0303 0303 0101 0204 0303 0202 0404 0404 0404 0101 0202 0104 0303 0103 0303 0202 0202 0404 0101 0202 0202 0303 0103 0303 0303 0204 0103 0101 0303 0101 0101 0404 0102 0202 0303 0202 0204 0101 0101 0404 0203 0202 0101 0101 0303 0404 0103 0303 0303 0202 0303 0104 0204 0103 0101 0404 0202 0103 0404 0204 0404 0202 0103 0404 0203 0202 0404 0202 0202 0101 0203 0202 0101 0202 0102 0202 0303 0101 0404 0104 0204 0202 0204 0102 0101 0202 0303 0303 0202 0303 0204 0202 0404 0103 0204 0204 0303 0404 0303 0101 0404 0404 0101 0101 0404 0101 0202 0103 0303 0304 0202 0202 0101 0303 0304 0404 0303 0202 0404 0204 0202 0204 0404 0303 0204 0303 0101 0303 0303 0303 0104 0102 0303 0101 0102 0204 0303 0303 0404 0303 0202 0404 0303 0103 0102 0303 0303 0101 0101 0404 0303 0404 0202 0303 0101 0103 0101 0303 0101 0202 0202 0103 0404 0101 0101 0404 0101 0103 0204 0202 0303 0101 0202 0202 0102 0303 0303 0404 0204 0404 0204 0304 0101 0204 0404 0204 0101 0101 0303 0103 0303 0404 0202 0103 0202 0303 0203 0202 0204 0104 0202 0303 0101 0304 0303 0202 0404 0101 0202 0204 0204 0204 0202 0203 0203 0404 0202 0303 0404 0204 0102 0404 0103 0404 0101 0103 0202 0303 0202 0303 0102 0303 0203 0404 0102 0101 0101 0303 0102 0304 0101 0404 0303 0303 0404 0404 0104 0103 0202 0102 0102 0103 0204 0103 0103 0102 0101 0202 0202 0202 0202 0203 0103 0404 0202 0202 0103 0303 0102 0202 0104 0202 0101 0202 0101 0202 0202 0104 0303 0202 0103 0101 0404 0303 0303 0404 0101 0303 0204 0202 0404 0303 0202 0101 0404 0202 0202 0202 0102 0303 0303 0101 0101 0303 0101 0303 0103 0103 0303 0202 0404 0102 0204 0101 0303 0404 0101 0202 0202 0303 0101 0202 0303 0202 0202 0102 0202 0404 0103 0303 0101 0404 0202 0202 0101 0303 0303 0202 0204 0202 0303 0303 0202 0303 0103 0102 0104 0303 0202 0202 0303 0101 0102 0404 0202 0404 0101 0101 0404 0202 0101 0202 0303 0204 0404 0202 0404 0204 0204 0202 0303 0303 0404 0303 0202 0204 0103 0202 0202 0103 0303 0404 0303 0404 0303 0303 0204 0303 0101 0202 0202 0303 0204 0404 0204 0303 0101 0101 0101 0303 0303 0204 0104 0404 0101 0303 0303 0303 0103 0404 0202 0303 0101 0204 0101 0404 0202 0101 0202 0303 0204 0104 0101 0202 0203 0303 0202 0101 0303 0303 0202 0303 0103 0404 0204 0404 0202 0202 0102 0101 0404 0101 0103 0303 0101 0303 0103 0202 0104 0404 0101 0404 0103 0204 0103 0303 0101 0103 0404 0404 0202 0101 0101 0303 0404 0303 0101 0203 0202 0103 0303 0202 0101 0101 0404 0202 0303 0103 0204 0203 0303 0202 0101 0303 0202 0404 0101 0202 0101 0202 0202 0303 0404 0404 0303 0101 0103 0202 0202 0104 0102 0303 0204 0303 0202 0101 0202 0303 0101 0203 0104 0202 0303 0202 0102 0204 0204 0204 0103 0103 0103 0303 0303 0404 0202 0202 0202 0404 0101 0202 0103 0101 0303 0303 0103 0202 0202 0404 0303 0204 0404 0101 0202 0303 0202 0202 0104 0104 0104 0104 0303 0202 0202 0104 0303 0101 0303 0202 0104 0202 0202 0202 0202 0404 0303 0101 0202 0202 0203 0204 0404 0103 0103 0404 0202 0404 0102 0202 0303 0303 0303 0204 0202 0102 0204 0202 0203 0304 0202 0101 0101 0303 0303 0303 0102 0204 0202 0101 0101 0404 0101 0202 0202 0202 0404 0303 0303 0103 0404 0202 0204 0303 0104 0202 0204 0404 0202 0202 0404 0202 0203 0204 0202 0202 0303 0101 0404 0103 0203 0101 0404 0204 0202 0404 0101 0303 0101 0101 0202 0101 0304 0404 0101 0202 0101 0203 0204 0303 0404 0303 0101 0203 0202 0303 0303 0304 0202 0202 0303 0303 0404 0202 0303 0202 0204 0303 0204 0103 0303 0202 0303 0404 0404 0404 0101 0304 0101 0202 0404 0204 0101 0101 0303 0404 0101 0404 0404 0202 0202 0202 0204 0202 0404 0101 0102 0204 0303 0404 0202 0303 0404 0404 0202 0404 0303 0404 0404 0404 0202 0101 0404 0202 0303 0101 0303 0404 0303 0203 0101 0303 0202 0404 0101 0104 0202 0304 0202 0101 0404 0202 0202 0303 0102 0101 0202 0202 0303 0303 0102 0101 0404 0202 0103 0202 0202 0101 0102 0103 0303 0404 0202 0304 0303 0101 0202 0303 0404 0101 0204 0204 0103 0202 0202 0303 0303 0101 0101 0202 0202 0101 0202 0404 0303 0202 0404 0303 0203 0103 0404 0101 0101 0303 0202 0304 0404 0101 0202 0303 0104 0204 0304 0303 0101 0404 0303 0202 0103 0202 0101 0202 0404 0204 0404 0202 0103 0104 0404 0104 0101 0303 0203 0303 0303 0303 0404 0303 0304 0202 0404 0101 0203 0404 0202 0202 0303 0104 0404 0204 0303 0303 0204 0102 0404 0103 0103 0303 0202 0101 0404 0303 0204 0204 0104 0102 0204 0202 0203 0103 0303 0103 0202 0101 0304 0304 0303 0101 0202 0101 0404 0104 0103 0101 0101 0404 0101 0101 0202 0404 0202 0404 0303 0202 0303 0404 0103 0202 0303 0202 0101 0404 0404 0404 0303 0101 0202 0303 0303 0202 0202 0101 0404 0303 0404 0303 0202 0404 0303 0101 0000 0303 0202 0101 0303 0303 0202 0404 0202 0202 0404 0303 0204 0404 0103 0303 0203 0101 0101 0404 0101 0101 0303 0303 0303 0101 0404 0104 0101 0101 0404 0304 0202 0202 0202 0202 0303 0304 0303 0102 0202 0303 0202 0404 0303 0202 0404 0101 0303 0101 0404 0303 0404 0404 0303 0202 0204 0104 0303 0303 0103 0304 0303 0204 0101 0303 0202 0202 0103 0204 0303 0303 0203 0202 0101 0202 0202 0303 0103 0404 0202 0202 0202 0204 0103 0104 0404 0104 0204 0203 0404 0303 0202 0202 0202 0101 0103 0102 0101 0101 0101 0303 0404 0104 0101 0203 0204 0204 0103 0404 0101 0104 0101 0101 0101 0103 0304 0303 0303 0404 0101 0404 0304 0303 0303 0202 0101 0404 0404 0202 0203 0202 0102 0101 0303 0103 0202 0303 0303 0202 0202 0102 0101 0202 0101 0202 0101 0303 0202 0303 0103 0203 0101 0202 0103 0404 0202 0101 0303 0202 0204 0103 0103 0203 0202 0202 0104 0103 0404 0303 0404 0404 0203 0204 0404 0202 0202 0103 0202 0303 0204 0202 0102 0204 0304 0104 0103 0404 0202 0204 0303 0303 0202 0202 0202 0203 0303 0102 0304 0404 0202 0202 0404 0404 0303 0404 0202 0202 0204 0404 0101 0202 0101 0101 0202 0303 0102 0103 0101 0303 0202 0204 0202 0202 0102 0101 0202 0202 0404 0101 0303 0202 0202 0202 0101 0303 0104 0202 0101 0303 0304 0103 0202 0404 0202 0404 0101 0104 0202 0404 0101 0202 0204 0303 0204 0303 0202 0303 0203 0202 0404 0303 0303 0102 0102 0202 0104 0101 0101 0303 0404 0202 0104 0303 0204 0303 0101 0202 0202 0303 0101 0103 0101 0103 0303 0303 0404 0102 0103 0303 0202 0202 0202 0404 0204 0303 0203 0104 0202 0102 0303 0204 0202 0101 0204 0303 0101 0404 0404 0103 0101 0304 0101 0303 0202 0204 0303 0304 0202 0202 0204 0404 0101 0404 0101 0202 0202 0204 0404 0204 0101 0101 0103 0204 0303 0101 0404 0202 0202 0103 0104 0103 0303 0303 0101 0102 0202 0101 0202 0303 0202 0101 0101 0303 0104 0303 0101 0303 0404 0303 0101 0404 0101 0103 0303 0202 0404 0103 0101 0102 0204 0101 0104 0204 0104 0303 0101 0103 0101 0202 0204 0101 0202 0204 0404 0303 0202 0303 0202 0101 0102 0104 0204 0102 0204 0202 0202 0204 0404 0404 0202 0202 0202 0202 0404 0404 0404 0103 0204 0202 0404 0202 0303 0101 0104 0404 0303 0102 0202 0303 0404 0203 0103 0202 0000 0303 0101 0303 0101 0103 0000 0102 0204 0202 0404 0204 0101 0202 0202 0303 0101 0202 0202 0404 0202 0204 0202 0404 0202 0303 0103 0102 0202 0204 0204 0103 0303 0303 0101 0404 0202 0104 0204 0304 0202 0202 0304 0104 0203 0101 0202 0101 0202 0303 0204 0404 0101 0101 0101 0204 0103 0101 0202 0303 0202 0204 0202 0202 0404 0202 0303 0303 0202 0202 0404 0404 0104 0202 0102 0103 0304 0404 0202 0202 0204 0104 0404 0202 0304 0404 0202 0404 0303 0101 0202 0404 0303 0204 0304 0404 0303 0204 0202 0404 0104 0104 0303 0303 0404 0103 0103 0204 0101 0202 0101 0404 0103 0204 0404 0404 0101 0404 0101 0303 0404 0103 0202 0104 0102 0104 0101 0101 0304 0404 0104 0101 0202 0404 0103 0404 0202 0404 0203 0202 0204 0103 0204 0404 0101 0404 0104 0102 0202 0202 0404 0202 0204 0404 0202 0203 0303 0304 0303 0303 0303 0202 0101 0303 0202 0404 0102 0202 0303 0304 0404 0202 0404 0103 0303 0202 0204 0303 0202 0303 0103 0101 0303 0303 0202 0303 0204 0102 0404 0303 0304 0103 0303 0101 0202 0303 0303 0101 0404 0103 0101 0104 0204 0202 0202 0202 0202 0202 0303 0204 0101 0103 0102 0202 0202 0303 0303 0202 0202 0204 0101 0104 0404 0101 0404 0101 0303 0303 0404 0102 0101 0202 0101 0204 0101 0404 0404 0101 0202 0202 0303 0202 0202 0202 0303 0204 0303 0204 0101 0303 0101 0303 0304 0101 0303 0103 0404 0202 0404 0303 0303 0103 0101 0404 0404 0404 0202 0202 0204 0104 0202 0204 0404 0202 0101 0303 0303 0304 0303 0104 0404 0303 0202 0303 0202 0303 0303 0304 0303 0202 0303 0202 0101 0103 0303 0204 0102 0101 0202 0202 0303 0303 0304 0303 0303 0103 0204 0303 0101 0404 0101 0101 0202 0101 0202 0304 0101 0303 0101 0103 0102 0101 0204 0104 0202 0104 0202 0101 0303 0404 0101 0404 0104 0202 0103 0303 0202 0303 0303 0303 0202 0103 0404 0101 0303 0404 0203 0204 0103 0303 0204 0202 0204 0101 0202 0204 0101 0102 0104 0101 0204 0303 0202 0101 0204 0303 0203 0104 0103 0303 0202 0303 0303 0303 0202 0202 0103 0303 0303 0202 0101 0303 0303 0101 0202 0203 0102 0303 0404 0103 0103 0303 0303 0101 0101 0404 0303 0101 0202 0404 0202 0202 0303 0202 0101 0404 0404 0101 0303 0303 0204 0202 0202 0303 0303 0303 0101 0204 0204 0102 0101 0404 0303 0202 0103 0404 0202 0303 0303 0303 0101 0102 0103 0202 0202 0204 0303 0202 0304 0101 0103 0101 0202 0404 0303 0204 0204 0104 0101 0404 0103 0202 0101 0203 0202 0303 0103 0303 0303 0404 0101 0204 0203 0303 0103 0303 0303 0303 0101 0101 0303 0102 0202 0203 0101 0101 0204 0202 0303 0303 0404 0101 0101 0202 0204 0202 0204 0303 0202 0101 0303 0404 0303 0103 0204 0202 0404 0202 0303 0303 0303 0103 0202 0103 0404 0204 0404 0202 0202 0202 0303 0202 0404 0404 0304 0202 0303 0101 0202 0303 0303 0202 0303 0204 0303 0202 0404 0303 0404 0101 0303 0303 0203 0204 0103 0103 0103 0303 0404 0303 0202 0202 0404 0204 0101 0202 0404 0101 0404 0104 0202 0102 0404 0101 0204 0303 0202 0404 0202 0202 0203 0303 0304 0304 0102 0103 0202 0101 0102 0303 0103 0303 0304 0101 0404 0202 0404 0404 0303 0303 0204 0203 0202 0103 0404 0101 0202 0303 0101 0404 0404 0101 0303 0304 0204 0404 0204 0202 0203 0103 0303 0101 0203 0404 0303 0404 0303 0404 0202 0104 0202 0103 0404 0202 0404 0303 0101 0202 0202 0204 0202 0102 0202 0202 0303 0303 0101 0404 0101 0303 0303 0101 0303 0101 0303 0103 0404 0101 0101 0202 0202 0202 0202 0303 0404 0102 0204 0202 0204 0303 0303 0204 0103 0202 0404 0404 0404 0202 0404 0404 0203 0204 0202 0303 0202 0304 0202 0303 0303 0103 0104 0303 0204 0202 0204 0202 0202 0202 0303 0303 0203 0303 0303 0202 0303 0303 0101 0202 0101 0303 0303 0303 0303 0404 0202 0104 0202 0202 0204 0404 0101 0104 0202 0101 0404 0303 0303 0303 0303 0304 0103 0404 0303 0303 0101 0103 0404 0303 0404 0202 0104 0202 0303 0202 0202 0303 0202 0202 0202 0203 0404 0303 0303 0202 0202 0103 0303 0202 0303 0202 0303 0304 0204 0202 0303 0204 0204 0303 0303 0404 0202 0101 0303 0404 0101 0404 0303 0202 0202 0303 0104 0101 0203 0101 0102 0103 0303 0303 0404 0202 0204 0404 0404 0303 0303 0101 0303 0303 0303 0101 0202 0304 0303 0404 0303 0202 0303 0202 0204 0202 0303 0202 0101 0202 0404 0303 0102 0303 0204 0202 0303 0304 0101 0202 0102 0303 0103 0303 0202 0202 0204 0303 0303 0104 0303 0202 0303 0404 0202 0202 0303 0204 0303 0101 0303 0303 0303 0404 0103 0102 0303 0103 0303 0202 0303 0203 0404 0202 0404 0104 0303 0303 0203 0103 0204 0204 0404 0202 0404 0202 0303 0404 0101 0303 0202 0101 0303 0303 0202 0303 0204 0104 0204 0101 0404 0101 0202 0404 0303 0101 0303 0202 0404 0404 0204 0204 0202 0104 0202 0404 0303 0202 0202 0202 0101 0102 0404 0103 0303 0303 0404 0202 0202 0404 0101 0303 0202 0202 0202 0101 0404 0202 0304 0404 0202 0102 0303 0202 0101 0202 0202 0404 0101 0102 0103 0204 0404 0101 0101 0303 0404 0404 0202 0102 0202 0303 0202 0202 0404 0303 0303 0103 0303 0202 0303 0304 0303 0404 0303 0303 0404 0202 0303 0202 0202 0303 0202 0303 0303 0404 0101 0204 0303 0202 0202 0202 0102 0101 0102 0204 0303 0303 0202 0101 0404 0303 0202 0404 0204 0303 0101 0404 0204 0103 0202 0303 0202 0404 0404 0404 0103 0303 0104 0404 0202 0303 0102 0303 0103 0303 0303 0404 0303 0303 0404 0404 0103 0202 0101 0404 0101 0101 0103 0101 0202 0101 0101 0303 0202 0202 0303 0202 0204 0101 0101 0101 0101 0404 0203 0204 0202 0303 0303 0404 0202 0404 0101 0303 0202 0102 0303 0202 0204 0204 0404 0104 0202 0202 0101 0304 0304 0404 0202 0102 0101 0303 0404 0303 0404 0203 0303 0303 0101 0404 0102 0103 0104 0202 0303 0303 0404 0103 0204 0202 0103 0104 0202 0202 0104 0404 0202 0202 0202 0101 0101 0303 0303 0303 0303 0101 0404 0303 0303 0202 0202 0404 0303 0404 0404 0303 0202 0204 0101 0404 0202 0303 0303 0101 0101 0303 0303 0000 0404 0303 0303 0202 0404 0204 0202 0102 0202 0101 0101 0303 0304 0204 0203 0404 0303 0303 0101 0404 0303 0103 0303 0103 0404 0204 0304 0202 0101 0203 0101 0304 0304 0404 0303 0303 0203 0202 0103 0203 0202 0303 0404 0101 0303 0101 0103 0202 0202 0101 0202 0202 0202 0304 0404 0304 0203 0102 0204 0101 0202 0204 0303 0202 0103 0202 0202 0102 0202 0404 0101 0202 0202 0303 0404 0202 0103 0103 0303 0303 0303 0404 0303 0101 0202 0303 0204 0103 0202 0404 0104 0202 0303 0203 0204 0101 0204 0404 0104 0404 0404 0203 0404 0101 0103 0303 0303 0104 0303 0303 0404 0202 0000 0101 0303 0102 0404 0204 0204 0303 0103 0101 0303 0404 0202 0202 0204 0303 0303 0303 0303 0202 0101 0202 0104 0303 0303 0202 0303 0303 0303 0104 0102 0101 0101 0101 0404 0204 0404 0103 0101 0101 0202 0404 0202 0202 0103 0202 0303 0303 0202 0202 0101 0204 0404 0202 0202 0303 0303 0303 0404 0102 0204 0303 0304 0101 0404 0303 0103 0203 0202 0101 0103 0404 0303 0102 0202 0202 0202 0404 0101 0303 0303 0303 0404 0404 0204 0202 0202 0000 0304 0101 0101 0404 0404 0202 0202 0404 0103 0101 0303 0103 0103 0101 0404 0202 0101 0204 0101 0101 0101 0102 0303 0104 0202 0101 0102 0204 0303 0101 0303 0101 0103 0303 0404 0202 0202 0303 0204 0303 0202 0101 0204 0103 0102 0202 0102 0101 0404 0304 0103 0404 0303 0102 0202 0202 0303 0101 0303 0102 0204 0102 0404 0102 0202 0303 0101 0204 0101 0404 0202 0000 0101 0303 0303 0101 0303 0303 0202 0303 0203 0202 0303 0303 0202 0303 0303 0303 0303 0202 0202 0303 0102 0202 0304 0303 0202 0202 0204 0303 0404 0202 0202 0101 0303 0102 0202 0303 0202 0202 0202 0101 0202 0303 0202 0101 0103 0202 0303 0404 0303 0101 0404 0204 0101 0303 0303 0202 0101 0303 0202 0202 0101 0103 0204 0101 0404 0304 0101 0303 0202 0102 0303 0404 0404 0202 0103 0202 0404 0303 0404 0303 0204 0101 0103 0101 0404 0404 0000 0404 0304 0303 0102 0304 0101 0204 0303 0104 0303 0103 0303 0101 0404 0101 0303 0404 0202 0202 0303 0303 0101 0101 0202 0202 0000 0404 0204 0103 0404 0101 0202 0404 0101 0101 0202 0104 0101 0404 0303 0101 0303 0202 0404 0202 0102 0103 0101 0303 0303 0303 0202 0303 0101 0103 0101 0202 0103 0203 0202 0101 0202 0202 0303 0202 0202 0202 0202 0303 0103 0202 0303 0103 0404 0404 0101 0202 0404 0101 0303 0202 0303 0303 0102 0204 0303 0202 0303 0202 0101 0103 0404 0404 0303 0202 0204 0202 0102 0204 0304 0303 0103 0404 0202 0204 0104 0203 0101 0404 0204 0303 0204 0202 0202 0404 0303 0202 0202 0101 0101 0404 0303 0202 0303 0202 0303 0103 0303 0103 0101 0404 0202 0104 0202 0202 0303 0104 0404 0404 0101 0404 0404 0101 0203 0304 0404 0202 0303 0104 0202 0101 0303 0204 0303 0404 0404 0304 0102 0102 0202 0303 0303 0101 0101 0202 0202 0103 0103 0404 0103 0202 0102 0101 0000 0102 0204 0202 0103 0103 0204 0102 0303 0304 0204 0103 0101 0101 0304 0304 0404 0303 0404 0202 0101 0303 0202 0202 0204 0102 0404 0404 0102 0202 0204 0202 0204 0204 0103 0202 0404 0202 0202 0303 0204 0404 0204 0404 0303 0303 0103 0101 0404 0203 0103 0303 0404 0404 0202 0404 0102 0202 0101 0202 0404 0303 0103 0101 0303 0303 0202 0404 0102 0101 0202 0404 0202 0202 0404 0404 0303 0404 0000 0202 0202 0303 0404 0101 0000 0404 0204 0203 0303 0102 0404 0101 0404 0404 0404 0404 0303 0303 0303 0202 0202 0204 0202 0202 0303 0303 0303 0303 0404 0404 0202 0103 0204 0202 0202 0303 0101 0202 0203 0303 0204 0202 0202 0303 0101 0102 0103 0404 0101 0104 0202 0303 0204 0202 0304 0202 0404 0203 0404 0101 0303 0303 0304 0101 0102 0102 0204 0202 0404 0303 0404 0101 0404 0404 0303 0303 0404 0404 0101 0204 0203 0204 0303 0202 0202 0202 0404 0103 0202 0404 0303 0101 0204 0101 0202 0404 0404 0204 0404 0202 0101 0303 0404 0404 0303 0102 0303 0101 0101 0103 0203 0202 0303 0204 0303 0202 0304 0404 0303 0000 0104 0102 0404 0103 0303 0303 0101 0101 0101 0101 0101 0202 0404 0102 0101 0303 0303 0202 0303 0101 0202 0404 0202 0404 0202 0101 0404 0104 0104 0202 0101 0104 0202 0101 0303 0303 0303 0101 0303 0102 0404 0303 0404 0204 0303 0204 0202 0102 0404 0103 0303 0303 0103 0303 0202 0104 0101 0202 0303 0101 0202 0202 0101 0103 0404 0202 0303 0303 0204 0404 0303 0101 0303 0202 0303 0202 0101 0202 0102 0103 0101 0103 0202 0202 0303 0101 0203 0404 0204 0204 0303 0303 0202 0404 0101 0404 0303 0202 0103 0103 0202 0103 0104 0202 0404 0404 0203 0102 0202 0102 0101 0202 0101 0303 0303 0404 0404 0303 0202 0303 0101 0303 0404 0202 0101 0202 0303 0404 0103 0204 0304 0103 0303 0102 0103 0202 0303 0104 0101 0104 0202 0204 0303 0303 0101 0102 0303 0102 0404 0303 0202 0202 0303 0103 0103 0103 0102 0103 0101 0104 0202 0404 0102 0404 0103 0404 0102 0102 0404 0202 0101 0101 0303 0404 0202 0101 0102 0101 0102 0204 0303 0102 0101 0104 0104 0303 0303 0104 0202 0304 0101 0303 0202 0303 0103 0204 0104 0404 0101 0103 0303 0202 0202 0303 0303 0202 0202 0102 0104 0404 0104 0404 0303 0202 0204 0202 0202 0000 0303 0303 0101 0101 0101 0101 0101 0404 0204 0102 0303 0303 0102 0303 0101 0404 0203 0303 0303 0303 0203 0204 0103 0303 0202 0404 0202 0404 0202 0101 0404 0404 0202 0202 0101 0202 0404 0101 0101 0202 0404 0202 0204 0103 0303 0101 0202 0303 0404 0101 0303 0404 0303 0303 0404 0303 0204 0303 0303 0303 0303 0202 0202 0303 0202 0101 0303 0303 0101 0202 0101 0101 0202 0404 0202 0101 0101 0303 0202 0101 0303 0202 0202 0303 0101 0202 0202 0202 0103 0303 0202 0203 0202 0202 0202 0202 0404 0101 0202 0101 0202 0101 0202 0202 0101 0202 0404 0303 0204 0303 0101 0202 0303 0404 0303 0404 0404 0404 0202 0101 0303 0202 0303 0103 0103 0303 0404 0404 0202 0204 0204 0202 0101 0203 0101 0404 0404 0202 0203 0202 0303 0202 0204 0202 0202 0202 0103 0104 0204 0404 0303 0204 0303 0202 0101 0404 0202 0404 0203 0303 0204 0204 0303 0101 0204 0101 0404 0303 0103 0102 0204 0101 0404 0404 0404 0404 0303 0303 0203 0101 0204 0000 0101 0404 0202 0404 0303 0303 0204 0202 0204 0203 0101 0202 0102 0102 0101 0202 0303 0303 0303 0204 0303 0303 0202 0104 0303 0202 0404 0202 0202 0101 0101 0104 0204 0303 0101 0204 0102 0303 0404 0202 0404 0303 0202 0101 0303 0101 0202 0303 0404 0202 0404 0103 0304 0303 0202 0303 0101 0303 0101 0202 0101 0404 0303 0202 0202 0104 0202 0303 0202 0101 0404 0404 0202 0103 0404 0101 0404 0101 0303 0303 0404 0202 0101 0303 0202 0202 0404 0101 0102 0102 0101 0104 0303 0101 0101 0202 0303 0404 0202 0304 0101 0404 0202 0101 0303 0103 0404 0103 0203 0303 0204 0204 0103 0103 0303 0202 0202 0404 0101 0303 0404 0103 0202 0104 0204 0303 0404 0104 0101 0303 0303 0202 0404 0204 0202 0202 0101 0303 0202 0202 0202 0404 0303 0303 0103 0303 0303 0303 0101 0303 0404 0404 0202 0303 0202 0204 0202 0303 0204 0202 0304 0202 0101 0203 0101 0202 0202 0404 0303 0303 0101 0202 0303 0304 0404 0303 0202 0103 0404 0404 0202 0303 0303 0203 0102 0202 0202 0204 0101 0103 0103 0303 0303 0101 0404 0103 0202 0102 0104 0202 0101 0103 0202 0101 0404 0101 0204 0101 0202 0101 0202 0202 0303 0202 0303 0103 0103 0104 0303 0303 0304 0101 0101 0303 0404 0202 0102 0202 0101 0202 0303 0101 0404 0204 0101 0303 0303 0303 0101 0202 0404 0303 0202 0101 0404 0303 0303 0202 0103 0303 0404 0303 0303 0303 0101 0404 0101 0202 0404 0103 0202 0202 0101 0202 0204 0202 0202 0103 0101 0203 0202 0202 0303 0101 0101 0404 0404 0202 0404 0103 0202 0202 0202 0202 0101 0103 0303 0303 0303 0404 0202 0404 0101 0103 0202 0304 0103 0404 0101 0303 0303 0303 0203 0202 0404 0103 0303 0101 0101 0202 0204 0202 0303 0202 0204 0103 0202 0404 0303 0103 0101 0102 0202 0303 0101 0404 0303 0202 0404 0202 0303 0303 0303 0103 0303 0103 0204 0101 0202 0303 0101 0303 0404 0202 0202 0103 0303 0303 0404 0102 0202 0303 0303 0303 0202 0204 0303 0103 0202 0202 0202 0202 0101 0303 0101 0303 0101 0202 0103 0103 0203 0303 0203 0202 0404 0202 0404 0404 0202 0404 0303 0404 0303 0404 0104 0204 0103 0202 0202 0304 0204 0203 0404 0303 0104 0104 0303 0101 0303 0202 0303 0101 0303 0101 0102 0202 0202 0102 0101 0303 0202 0404 0304 0104 0101 0303 0303 0303 0303 0303 0303 0102 0303 0303 0303 0303 0204 0203 0202 0204 0104 0404 0303 0303 0303 0404 0304 0103 0303 0404 0101 0101 0303 0304 0204 0404 0204 0202 0303 0303 0101 0202 0304 0404 0202 0303 0102 0202 0303 0303 0000 0202 0404 0204 0303 0101 0101 0203 0101 0202 0304 0404 0000 0102 0303 0303 0202 0202 0104 0303 0202 0202 0404 0103 0303 0404 0202 0101 0303 0101 0102 0404 0101 0101 0202 0303 0101 0202 0202 0202 0404 0404 0404 0103 0404 0304 0202 0303 0303 0303 0202 0203 0202 0202 0303 0202 0303 0404 0303 0303 0101 0202 0104 0101 0202 0303 0303 0101 0103 0203 0103 0204 0101 0102 0202 0204 0303 0404 0101 0404 0102 0404 0404 0103 0202 0202 0204 0202 0103 0202 0303 0104 0202 0404 0202 0102 0304 0101 0303 0303 0203 0404 0000 0303 0202 0304 0204 0102 0101 0104 0202 0101 0204 0202 0203 0303 0404 0404 0404 0103 0404 0303 0404 0303 0303 0104 0303 0202 0101 0304 0404 0101 0202 0303 0204 0303 0103 0202 0202 0404 0204 0303 0101 0202 0303 0303 0303 0304 0204 0101 0202 0203 0404 0101 0202 0303 0303 0203 0303 0203 0204 0202 0404 0103 0101 0404 0404 0303 0202 0101 0103 0204 0404 0101 0303 0204 0303 0202 0202 0303 0202 0202 0303 0202 0303 0303 0101 0202 0303 0101 0404 0404 0202 0202 0204 0202 0202 0101 0101 0204 0404 0204 0404 0101 0404 0101 0404 0104 0103 0203 0204 0202 0102 0101 0101 0202 0101 0204 0204 0202 0303 0303 0204 0303 0304 0104 0000 0303 0202 0104 0303 0204 0404 0202 0104 0202 0404 0202 0101 0202 0202 0103 0202 0404 0000 0303 0101 0101 0404 0101 0404 0101 0303 0202 0204 0202 0202 0103 0303 0404 0103 0104 0202 0303 0103 0204 0202 0303 0203 0202 0202 0303 0404 0202 0303 0303 0303 0104 0101 0303 0404 0203 0304 0404 0101 0303 0404 0404 0303 0202 0101 0101 0101 0303 0102 0303 0202 0303 0303 0404 0404 0303 0104 0404 0103 0202 0104 0404 0404 0101 0404 0202 0303 0102 0204 0202 0203 0202 0101 0303 0101 0404 0102 0101 0101 0202 0203 0103 0404 0104 0101 0202 0303 0202 0202 0303 0202 0202 0202 0202 0101 0304 0202 0303 0303 0304 0303 0204 0102 0404 0404 0202 0303 0204 0101 0104 0202 0203 0204 0000 0202 0202 0101 0101 0204 0000 0101 0204 0404 0404 0202 0202 0303 0404 0000 0202 0101 0202 0101 0000 0303 0303 0104 0202 0404 0303 0202 0104 0104 0102 0303 0304 0404 0101 0102 0101 0303 0101 0202 0303 0204 0101 0303 0202 0103 0202 0304 0103 0202 0304 0202 0202 0202 0101 0104 0102 0203 0202 0202 0202 0204 0404 0204 0404 0404 0404 0204 0202 0204 0303 0303 0303 0404 0202 0303 0404 0304 0404 0203 0202 0204 0202 0303 0103 0304 0203 0101 0101 0101 0103 0101 0101 0202 0101 0204 0204 0103 0101 0202 0101 0303 0202 0303 0202 0404 0303 0102 0404 0202 0204 0102 0404 0104 0101 0404 0303 0404 0202 0303 0404 0303 0104 0303 0101 0303 0202 0404 0103 0404 0404 0303 0202 0101 0101 0404 0404 0404 0303 0303 0204 0101 0202 0202 0202 0303 0101 0101 0303 0303 0101 0101 0404 0204 0304 0202 0104 0101 0101 0303 0202 0202 0202 0103 0303 0303 0101 0303 0404 0103 0202 0303 0101 0103 0104 0202 0303 0404 0101 0303 0103 0303 0202 0303 0404 0101 0202 0204 0202 0404 0101 0101 0303 0303 0202 0303 0103 0202 0303 0203 0101 0404 0404 0204 0202 0103 0204 0303 0101 0202 0101 0303 0202 0404 0202 0202 0202 0202 0303 0404 0303 0202 0202 0404 0303 0303 0202 0303 0104 0404 0303 0404 0202 0102 0102 0303 0303 0104 0303 0202 0202 0204 0303 0101 0404 0404 0303 0000 0202 0303 0202 0101 0304 0404 0404 0000 0202 0104 0303 0203 0202 0303 0303 0202 0304 0202 0404 0101 0103 0202 0101 0202 0204 0404 0303 0103 0303 0303 0304 0303 0404 0202 0202 0304 0303 0202 0303 0202 0404 0202 0202 0204 0103 0202 0202 0102 0303 0303 0303 0303 0102 0303 0303 0303 0303 0104 0404 0303 0303 0104 0101 0404 0202 0202 0202 0404 0202 0202 0204 0404 0303 0101 0404 0404 0404 0103 0303 0101 0103 0303 0404 0101 0404 0303 0202 0202 0204 0101 0101 0101 0103 0303 0303 0404 0101 0202 0303 0104 0303 0101 0202 0404 0104 0202 0101 0303 0103 0103 0404 0303 0404 0101 0103 0104 0303 0303 0204 0404 0404 0202 0303 0404 0203 0101 0101 0304 0303 0202 0101 0303 0303 0303 0404 0303 0103 0101 0104 0204 0204 0303 0103 0103 0303 0202 0202 0202 0303 0303 0204 0103 0103 0303 0303 0202 0303 0303 0204 0202 0303 0202 0202 0204 0101 0101 0103 0202 0202 0202 0303 0101 0101 0303 0303 0202 0103 0204 0202 0202 0103 0102 0202 0303 0104 0101 0103 0104 0202 0204 0104 0204 0303 0303 0103 0202 0202 0303 0303 0202 0404 0104 0101 0104 0404 0303 0204 0303 0202 0202 0303 0103 0203 0103 0303 0303 0202 0202 0202 0202 0202 0404 0101 0204 0202 0303 0404 0202 0404 0303 0303 0204 0404 0303 0103 0202 0304 0202 0303 0303 0303 0304 0101 0404 0103 0202 0404 0104 0202 0202 0103 0204 0303 0101 0303 0202 0303 0404 0103 0303 0404 0303 0103 0404 0304 0101 0101 0404 0303 0303 0101 0404 0303 0202 0103 0303 0202 0404 0202 0404 0202 0202 0202 0101 0303 0303 0202 0404 0101 0202 0101 0404 0202 0303 0303 0304 0203 0303 0404 0303 0202 0202 0204 0303 0103 0404 0101 0303 0101 0303 0303 0101 0104 0202 0404 0303 0204 0202 0101 0202 0303 0303 0101 0303 0304 0202 0203 0404 0202 0303 0202 0202 0202 0101 0202 0303 0202 0102 0204 0202 0303 0202 0303 0404 0101 0202 0303 0204 0404 0102 0103 0202 0304 0404 0303 0404 0202 0202 0103 0101 0303 0103 0202 0303 0204 0404 0202 0304 0202 0304 0303 0202 0404 0101 0101 0202 0203 0204 0404 0202 0404 0204 0304 0303 0404 0303 0101 0103 0404 0303 0102 0203 0104 0203 0101 0203 0204 0202 0303 0303 0202 0102 0404 0303 0303 0101 0303 0303 0203 0404 0404 0202 0202 0202 0202 0202 0104 0202 0101 0202 0202 0202 0404 0303 0404 0404 0202 0404 0303 0202 0303 0303 0404 0303 0204 0204 0101 0404 0202 0202 0202 0303 0204 0303 0404 0103 0104 0303 0303 0101 0202 0404 0202 0202 0102 0404 0101 0404 0101 0404 0101 0404 0103 0202 0304 0101 0202 0303 0103 0202 0303 0404 0101 0202 0103 0404 0101 0202 0101 0202 0404 0103 0404 0104 0404 0101 0204 0303 0303 0202 0404 0404 0404 0101 0404 0101 0204 0303 0202 0103 0303 0104 0204 0202 0204 0304 0303 0204 0303 0102 0101 0404 0101 0404 0404 0101 0303 0103 0103 0303 0202 0103 0101 0202 0101 0202 0404 0101 0404 0203 0101 0203 0202 0101 0202 0103 0204 0303 0303 0202 0404 0203 0404 0103 0404 0202 0202 0101 0404 0204 0102 0404 0303 0303 0404 0102 0101 0202 0102 0202 0103 0101 0404 0303 0103 0404 0202 0303 0202 0404 0103 0202 0101 0101 0101 0104 0101 0102 0104 0101 0202 0202 0303 0202 0303 0202 0103 0404 0404 0303 0103 0303 0202 0204 0303 0404 0202 0101 0304 0303 0404 0303 0101 0204 0202 0303 0202 0202 0204 0204 0202 0202 0103 0104 0303 0202 0202 0202 0303 0101 0304 0101 0404 0303 0304 0104 0102 0101 0404 0204 0404 0202 0303 0104 0203 0303 0202 0202 0101 0304 0101 0000 0404 0101 0103 0202 0202 0101 0303 0101 0204 0303 0103 0202 0303 0202 0202 0303 0404 0204 0202 0204 0404 0101 0202 0103 0101 0203 0202 0303 0101 0202 0101 0404 0304 0202 0303 0202 0202 0202 0404 0103 0202 0404 0101 0303 0404 0101 0101 0202 0202 0103 0102 0101 0303 0204 0303 0102 0000 0304 0202 0202 0202 0303 0101 0103 0303 0303 0102 0102 0303 0304 0404 0404 0303 0303 0303 0103 0202 0101 0101 0101 0202 0404 0204 0303 0101 0101 0103 0404 0404 0202 0303 0404 0303 0101 0000 0404 0203 0202 0202 0202 0204 0103 0204 0304 0303 0103
[truncated: 4,755,781 more chars]
