# Supplementary material for: Genomic Signatures After Five Generations of Intensive Selective Breeding: Runs of Homozygosity and Genetic Diversity in Representative Domestic and Wild Populations of Turbot (Scophthalmus maximus)
Source: Front Genet. 2020 Apr 3;11:296. doi: 10.3389/fgene.2020.00296 (PMC7169425; doi:10.3389/fgene.2020.00296)
Supplement: Supplementary file 4 [file Table_4.docx]

Supplementary table 4. Genotyping of 25.681 SNP loci in wild turbot (36 individuals) using Genepop format (http://genepop.curtin.edu.au/). SNP genotypes are indicated as follows: 01 = A, 02 = C, 03 = G and 04 = T.

1_50253, 1_50525, 1_66367, 1_85037, 1_96907, 1_126882, 1_134273, 1_150956, 1_152573, 1_172861, 1_178489, 1_178846, 1_187323, 1_211001, 1_212734, 1_232896, 1_239638, 1_293507, 1_293832, 1_315772, 1_321098, 1_330795, 1_346598, 1_356823, 1_372671, 1_412833, 1_418537, 1_430602, 1_431077, 1_434701, 1_448034, 1_453875, 1_459561, 1_462984, 1_468020, 1_470128, 1_495409, 1_496380, 1_501139, 1_516517, 1_520007, 1_525599, 1_557303, 1_563938, 1_572984, 1_588807, 1_597610, 1_632769, 1_633406, 1_635675, 1_636168, 1_636512, 1_661843, 1_680153, 1_686905, 1_698334, 1_699268, 1_700390, 1_732137, 1_769102, 1_775193, 1_811451, 1_833458, 1_833933, 1_839994, 1_840802, 1_846753, 1_848252, 1_848630, 1_850366, 1_933069, 1_949777, 1_973892, 1_984511, 1_990025, 1_990089, 1_995069, 1_1011793, 1_1020367, 1_1041708, 1_1045800, 1_1059693, 1_1080861, 1_1085278, 1_1089562, 1_1139764, 1_1147995, 1_1179011, 1_1182761, 1_1184890, 1_1218345, 1_1234020, 1_1237098, 1_1241119, 1_1241352, 1_1245013, 1_1256608, 1_1259334, 1_1290293, 1_1293517, 1_1296590, 1_1299751, 1_1306546, 1_1315922, 1_1322179, 1_1333004, 1_1334300, 1_1339031, 1_1343780, 1_1352314, 1_1361106, 1_1367016, 1_1374790, 1_1379477, 1_1385743, 1_1385967, 1_1401696, 1_1408743, 1_1409793, 1_1450781, 1_1457483, 1_1494993, 1_1495975, 1_1507018, 1_1511540, 1_1526344, 1_1555232, 1_1563639, 1_1564070, 1_1564148, 1_1575059, 1_1576269, 1_1576295, 1_1579880, 1_1588744, 1_1617341, 1_1618029, 1_1651870, 1_1652912, 1_1653693, 1_1659852, 1_1660232, 1_1668220, 1_1685352, 1_1686950, 1_1693798, 1_1703603, 1_1717776, 1_1751632, 1_1779667, 1_1799437, 1_1802044, 1_1826792, 1_1834764, 1_1835174, 1_1846866, 1_1852335, 1_1856128, 1_1857883, 1_1864861, 1_1892477, 1_1901477, 1_1929310, 1_1930196, 1_1932029, 1_1942637, 1_1976449, 1_1980550, 1_1985286, 1_2004772, 1_2020111, 1_2025283, 1_2063631, 1_2087226, 1_2090068, 1_2094085, 1_2103451, 1_2120786, 1_2121285, 1_2123140, 1_2127285, 1_2148605, 1_2152056, 1_2160875, 1_2174377, 1_2175157, 1_2179839, 1_2183595, 1_2185106, 1_2205701, 1_2220374, 1_2221151, 1_2232998, 1_2241212, 1_2256817, 1_2260500, 1_2263403, 1_2268833, 1_2269487, 1_2273926, 1_2290762, 1_2293215, 1_2295134, 1_2295805, 1_2299392, 1_2306240, 1_2320345, 1_2321264, 1_2325115, 1_2326713, 1_2334071, 1_2351462, 1_2351605, 1_2361731, 1_2367695, 1_2380284, 1_2383854, 1_2390839, 1_2400407, 1_2441703, 1_2454214, 1_2460062, 1_2472780, 1_2480553, 1_2482118, 1_2491181, 1_2491705, 1_2492234, 1_2504373, 1_2562254, 1_2563997, 1_2585250, 1_2588747, 1_2599491, 1_2601403, 1_2604085, 1_2605312, 1_2607615, 1_2609026, 1_2623889, 1_2625837, 1_2636450, 1_2658141, 1_2665034, 1_2667161, 1_2672349, 1_2677924, 1_2705629, 1_2712797, 1_2715493, 1_2715980, 1_2717703, 1_2717788, 1_2742493, 1_2748704, 1_2768103, 1_2784748, 1_2794879, 1_2795113, 1_2796400, 1_2800580, 1_2806108, 1_2809389, 1_2814965, 1_2843399, 1_2849878, 1_2851469, 1_2854673, 1_2909630, 1_2912497, 1_2915891, 1_2933229, 1_2966220, 1_2974721, 1_2987231, 1_2995067, 1_3007514, 1_3015889, 1_3044839, 1_3045756, 1_3045852, 1_3047358, 1_3051992, 1_3052170, 1_3067234, 1_3078053, 1_3088684, 1_3090240, 1_3095365, 1_3163842, 1_3190028, 1_3206369, 1_3241468, 1_3241789, 1_3251404, 1_3266006, 1_3291503, 1_3338002, 1_3385797, 1_3387066, 1_3389483, 1_3402715, 1_3403461, 1_3422312, 1_3437501, 1_3445863, 1_3447243, 1_3479779, 1_3500311, 1_3517564, 1_3530687, 1_3535520, 1_3541575, 1_3565972, 1_3566925, 1_3567361, 1_3567842, 1_3584644, 1_3600239, 1_3602192, 1_3620151, 1_3641319, 1_3645926, 1_3650248, 1_3661679, 1_3712392, 1_3727408, 1_3756310, 1_3776741, 1_3777010, 1_3780952, 1_3806157, 1_3812694, 1_3813144, 1_3831024, 1_3853399, 1_3853619, 1_3858346, 1_3863160, 1_3863540, 1_3879143, 1_3884023, 1_3896017, 1_3898354, 1_3899485, 1_3944957, 1_3965879, 1_3999277, 1_4096475, 1_4103400, 1_4103641, 1_4128511, 1_4182323, 1_4205802, 1_4225224, 1_4244324, 1_4274500, 1_4280165, 1_4305343, 1_4333851, 1_4339866, 1_4347877, 1_4366870, 1_4368962, 1_4400566, 1_4428726, 1_4450609, 1_4460136, 1_4468890, 1_4555586, 1_4565989, 1_4594713, 1_4618411, 1_4641113, 1_4672652, 1_4703924, 1_4715286, 1_4725874, 1_4729738, 1_4737953, 1_4796089, 1_4847203, 1_4852789, 1_4855177, 1_4871080, 1_4877895, 1_4885148, 1_4888526, 1_4893922, 1_4901783, 1_4913909, 1_4918852, 1_4933249, 1_4971425, 1_4974430, 1_4982604, 1_4984042, 1_5013692, 1_5035687, 1_5060632, 1_5072460, 1_5072764, 1_5097857, 1_5107917, 1_5151814, 1_5158537, 1_5171124, 1_5173461, 1_5179620, 1_5202697, 1_5202875, 1_5217269, 1_5220979, 1_5228773, 1_5235320, 1_5238754, 1_5245545, 1_5256619, 1_5266768, 1_5293411, 1_5304294, 1_5304423, 1_5327135, 1_5330490, 1_5346667, 1_5361147, 1_5369033, 1_5374637, 1_5384424, 1_5398105, 1_5410084, 1_5419229, 1_5419932, 1_5422856, 1_5452640, 1_5456765, 1_5481929, 1_5482152, 1_5486821, 1_5487363, 1_5501517, 1_5521954, 1_5530493, 1_5538981, 1_5539224, 1_5545218, 1_5561298, 1_5569424, 1_5608252, 1_5610638, 1_5619663, 1_5648671, 1_5672472, 1_5683841, 1_5685908, 1_5695022, 1_5699145, 1_5703407, 1_5709174, 1_5710411, 1_5715582, 1_5722817, 1_5757740, 1_5791982, 1_5819127, 1_5823630, 1_5871704, 1_5875296, 1_5893011, 1_5894972, 1_5949791, 1_6049359, 1_6053852, 1_6064909, 1_6080305, 1_6087183, 1_6097707, 1_6140851, 1_6151915, 1_6177898, 1_6195111, 1_6209616, 1_6219234, 1_6286936, 1_6296463, 1_6367497, 1_6376390, 1_6388092, 1_6416342, 1_6420400, 1_6435459, 1_6481428, 1_6498179, 1_6524229, 1_6525965, 1_6543626, 1_6567457, 1_6571969, 1_6573069, 1_6587618, 1_6588165, 1_6601290, 1_6608138, 1_6611434, 1_6626909, 1_6678283, 1_6683923, 1_6687541, 1_6695117, 1_6709206, 1_6711283, 1_6743250, 1_6828099, 1_6834315, 1_6840408, 1_6842044, 1_6862113, 1_6865675, 1_6869656, 1_6877460, 1_6878486, 1_6893677, 1_6895374, 1_6901606, 1_6911320, 1_6916715, 1_6928155, 1_6958251, 1_6973150, 1_6983463, 1_6987390, 1_7001614, 1_7017077, 1_7017302, 1_7018221, 1_7046371, 1_7048322, 1_7053834, 1_7058315, 1_7060471, 1_7191023, 1_7213545, 1_7225064, 1_7253183, 1_7254824, 1_7316321, 1_7350609, 1_7379420, 1_7389769, 1_7405572, 1_7417644, 1_7433375, 1_7434087, 1_7437585, 1_7443675, 1_7475036, 1_7486904, 1_7502145, 1_7524135, 1_7527927, 1_7555618, 1_7560443, 1_7565182, 1_7581039, 1_7586666, 1_7632048, 1_7632270, 1_7634128, 1_7640004, 1_7661961, 1_7714808, 1_7717411, 1_7746701, 1_7765264, 1_7767034, 1_7771938, 1_7793714, 1_7813314, 1_7829807, 1_7850022, 1_7874515, 1_7903177, 1_7910151, 1_7952681, 1_7960225, 1_7997323, 1_8004304, 1_8084618, 1_8151707, 1_8163911, 1_8166566, 1_8171138, 1_8215399, 1_8233445, 1_8234487, 1_8266566, 1_8276847, 1_8303977, 1_8317725, 1_8334592, 1_8335596, 1_8362401, 1_8392480, 1_8423659, 1_8445201, 1_8460884, 1_8469021, 1_8505604, 1_8553981, 1_8595436, 1_8599540, 1_8599983, 1_8611682, 1_8649100, 1_8681290, 1_8708131, 1_8722328, 1_8722893, 1_8725760, 1_8735262, 1_8741180, 1_8751787, 1_8762485, 1_8769782, 1_8771265, 1_8782484, 1_8786561, 1_8787177, 1_8787491, 1_8787805, 1_8813840, 1_8830791, 1_8832368, 1_8891502, 1_8905246, 1_8906794, 1_8917775, 1_8957606, 1_8978303, 1_9031221, 1_9034748, 1_9039434, 1_9042234, 1_9092832, 1_9102217, 1_9110918, 1_9139841, 1_9185046, 1_9186273, 1_9233034, 1_9233352, 1_9246652, 1_9250328, 1_9254660, 1_9260447, 1_9285290, 1_9297359, 1_9312079, 1_9343800, 1_9345125, 1_9353860, 1_9356412, 1_9378132, 1_9394706, 1_9414486, 1_9417008, 1_9487934, 1_9509930, 1_9517181, 1_9524104, 1_9531342, 1_9536324, 1_9567357, 1_9569448, 1_9572676, 1_9595995, 1_9599800, 1_9608927, 1_9622120, 1_9627476, 1_9649192, 1_9655817, 1_9657642, 1_9663667, 1_9695158, 1_9731143, 1_9766348, 1_9776219, 1_9776419, 1_9802225, 1_9874234, 1_9933879, 1_9959524, 1_9966045, 1_10009988, 1_10110821, 1_10137896, 1_10178528, 1_10187828, 1_10195686, 1_10227412, 1_10268920, 1_10310860, 1_10476774, 1_10515206, 1_10554644, 1_10633346, 1_10645239, 1_10650463, 1_10670474, 1_10683273, 1_10687181, 1_10688108, 1_10693865, 1_10844212, 1_10875822, 1_10879702, 1_10901235, 1_10968031, 1_10986300, 1_10988360, 1_11026196, 1_11036907, 1_11042662, 1_11056360, 1_11074104, 1_11085618, 1_11099043, 1_11155854, 1_11171759, 1_11219391, 1_11317552, 1_11324761, 1_11337399, 1_11341738, 1_11347475, 1_11360161, 1_11366526, 1_11434204, 1_11444419, 1_11460730, 1_11464385, 1_11466216, 1_11497491, 1_11530967, 1_11549888, 1_11573233, 1_11630715, 1_11635353, 1_11658079, 1_11690376, 1_11745214, 1_11755158, 1_11803609, 1_11817952, 1_11835047, 1_11847939, 1_11864758, 1_11872865, 1_11940456, 1_11961367, 1_11986549, 1_12006770, 1_12010633, 1_12030373, 1_12031815, 1_12059315, 1_12063372, 1_12074042, 1_12076813, 1_12092847, 1_12093462, 1_12100623, 1_12115316, 1_12126290, 1_12139045, 1_12151853, 1_12152019, 1_12189095, 1_12272006, 1_12281537, 1_12346125, 1_12404526, 1_12413076, 1_12433455, 1_12436553, 1_12459675, 1_12490148, 1_12533081, 1_12589344, 1_12600828, 1_12605693, 1_12622183, 1_12638598, 1_12657823, 1_12673136, 1_12725464, 1_12811449, 1_12848091, 1_12876184, 1_12886538, 1_12931770, 1_12934744, 1_12943835, 1_12957899, 1_12986368, 1_13000595, 1_13010329, 1_13020118, 1_13022445, 1_13039116, 1_13103341, 1_13143205, 1_13148469, 1_13161080, 1_13163631, 1_13176271, 1_13201511, 1_13301886, 1_13324608, 1_13337721, 1_13339997, 1_13341949, 1_13372898, 1_13403746, 1_13415544, 1_13428114, 1_13428399, 1_13450857, 1_13457708, 1_13519485, 1_13545098, 1_13558308, 1_13578370, 1_13604956, 1_13616013, 1_13646889, 1_13697566, 1_13709103, 1_13720545, 1_13760488, 1_13767593, 1_13886914, 1_13895964, 1_13926698, 1_13992339, 1_14005870, 1_14033449, 1_14048656, 1_14054761, 1_14072212, 1_14086015, 1_14093785, 1_14130190, 1_14136297, 1_14152207, 1_14176612, 1_14186538, 1_14213181, 1_14214647, 1_14234504, 1_14278357, 1_14341989, 1_14347893, 1_14351715, 1_14353457, 1_14356347, 1_14371123, 1_14413137, 1_14433194, 1_14445073, 1_14507463, 1_14549474, 1_14558015, 1_14558363, 1_14640252, 1_14649147, 1_14651047, 1_14658975, 1_14712666, 1_14739638, 1_14739909, 1_14744938, 1_14910365, 1_14952584, 1_14974037, 1_14977306, 1_14985036, 1_14985415, 1_14997847, 1_15030064, 1_15045314, 1_15068994, 1_15078862, 1_15079267, 1_15079643, 1_15084720, 1_15086338, 1_15087402, 1_15095683, 1_15115459, 1_15126904, 1_15132809, 1_15181146, 1_15196831, 1_15201648, 1_15214600, 1_15262019, 1_15284228, 1_15288535, 1_15301746, 1_15313657, 1_15352383, 1_15380492, 1_15392884, 1_15398183, 1_15451742, 1_15464453, 1_15478341, 1_15494508, 1_15540222, 1_15568648, 1_15589416, 1_15633574, 1_15664127, 1_15732834, 1_15742951, 1_15745870, 1_15752553, 1_15753034, 1_15794431, 1_15814859, 1_15815335, 1_15821964, 1_15859729, 1_15897801, 1_15901584, 1_15903271, 1_15908751, 1_15928844, 1_16000021, 1_16013597, 1_16015014, 1_16021236, 1_16042355, 1_16104707, 1_16144047, 1_16146462, 1_16164605, 1_16167095, 1_16187836, 1_16199307, 1_16200424, 1_16200715, 1_16206841, 1_16215083, 1_16238682, 1_16256855, 1_16277253, 1_16299282, 1_16301816, 1_16305220, 1_16316020, 1_16328673, 1_16328908, 1_16337764, 1_16341926, 1_16345963, 1_16406676, 1_16471313, 1_16492020, 1_16494814, 1_16538678, 1_16543733, 1_16579441, 1_16581705, 1_16594822, 1_16599550, 1_16649689, 1_16705517, 1_16711703, 1_16712906, 1_16736931, 1_16762969, 1_16766397, 1_16771448, 1_16789055, 1_16852107, 1_16854070, 1_16893241, 1_16909925, 1_16942894, 1_16964824, 1_17010513, 1_17037421, 1_17042248, 1_17125690, 1_17144404, 1_17158410, 1_17173725, 1_17217765, 1_17221452, 1_17243110, 1_17248562, 1_17272905, 1_17381900, 1_17431282, 1_17507264, 1_17528539, 1_17541882, 1_17580513, 1_17604141, 1_17614516, 1_17661418, 1_17665871, 1_17783309, 1_17800828, 1_17865133, 1_17912451, 1_17915946, 1_17925849, 1_17953090, 1_17974699, 1_17997250, 1_18009957, 1_18018590, 1_18045811, 1_18068131, 1_18070967, 1_18085779, 1_18091120, 1_18215891, 1_18227110, 1_18278849, 1_18282623, 1_18340353, 1_18344004, 1_18346961, 1_18347235, 1_18355778, 1_18359548, 1_18400294, 1_18423572, 1_18427221, 1_18450762, 1_18452300, 1_18458954, 1_18529546, 1_18557547, 1_18557724, 1_18571600, 1_18615590, 1_18676313, 1_18682178, 1_18712516, 1_18721907, 1_18727478, 1_18756088, 1_18789404, 1_18828694, 1_18839781, 1_18840760, 1_18856665, 1_18860981, 1_18884667, 1_18893376, 1_18922777, 1_19010528, 1_19047510, 1_19135120, 1_19135197, 1_19168018, 1_19171970, 1_19175821, 1_19176413, 1_19188025, 1_19215713, 1_19246269, 1_19271862, 1_19299605, 1_19307784, 1_19318791, 1_19429924, 1_19530666, 1_19664261, 1_19675298, 1_19819568, 1_19823753, 1_19843578, 1_19848168, 1_19857126, 1_19869504, 1_19922332, 1_19936685, 1_19967926, 1_19971437, 1_20002267, 1_20057889, 1_20075185, 1_20082532, 1_20114983, 1_20117407, 1_20164060, 1_20218936, 1_20252715, 1_20253218, 1_20270360, 1_20282815, 1_20401445, 1_20413635, 1_20468795, 1_20538805, 1_20540082, 1_20546895, 1_20547033, 1_20550218, 1_20600623, 1_20648360, 1_20651168, 1_20706724, 1_20735651, 1_20777059, 1_20811558, 1_20856663, 1_20859122, 1_20866812, 1_20879896, 1_20912195, 1_20916274, 1_20919653, 1_20921867, 1_20935413, 1_20936603, 1_20959146, 1_20984831, 1_20985544, 1_21041680, 1_21054047, 1_21055291, 1_21069958, 1_21084287, 1_21123461, 1_21125927, 1_21128768, 1_21162978, 1_21183610, 1_21186738, 1_21193442, 1_21203269, 1_21217458, 1_21232199, 1_21282759, 1_21287022, 1_21289032, 1_21303686, 1_21329805, 1_21333259, 1_21405143, 1_21448980, 1_21555878, 1_21598288, 1_21625048, 1_21679457, 1_21681946, 1_21725996, 1_21750868, 1_21754452, 1_21770177, 1_21773400, 1_21805602, 1_21845950, 1_21848280, 1_21860674, 1_21873524, 1_21873921, 1_21889635, 1_21946913, 1_21957666, 1_21972003, 1_21981163, 1_21993900, 1_22059645, 1_22103303, 1_22153872, 1_22183619, 1_22183908, 1_22205477, 1_22226761, 1_22239795, 1_22256449, 1_22333984, 1_22342900, 1_22348019, 1_22383602, 1_22387377, 1_22388306, 1_22400312, 1_22434073, 1_22440227, 1_22473126, 1_22495176, 1_22543058, 1_22548563, 1_22565174, 1_22566473, 1_22586460, 1_22588724, 1_22600462, 1_22614577, 1_22626719, 1_22634352, 1_22656724, 1_22672450, 1_22677912, 1_22706400, 1_22727540, 1_22731771, 1_22736539, 1_22740642, 1_22762072, 1_22764965, 1_22856647, 1_22863656, 1_22970019, 1_22991932, 1_23003256, 1_23021881, 1_23048221, 1_23060516, 1_23083436, 1_23184985, 1_23189381, 1_23191702, 1_23198084, 1_23214969, 1_23262471, 1_23300904, 1_23311945, 1_23321471, 1_23356046, 1_23418769, 1_23450569, 1_23459427, 1_23467301, 1_23478329, 1_23526025, 1_23530256, 1_23536249, 1_23538160, 1_23572008, 1_23616927, 1_23667972, 1_23722556, 1_23755866, 1_23794537, 1_23796932, 1_23799153, 1_23799825, 1_23948070, 1_24031621, 1_24050899, 1_24061186, 1_24061373, 1_24143017, 1_24188055, 1_24200273, 1_24228507, 1_24236094, 1_24238678, 1_24290912, 1_24329029, 1_24364345, 1_24402583, 1_24406873, 1_24450096, 1_24505144, 1_24524993, 1_24541513, 1_24546872, 1_24547943, 1_24549048, 1_24566680, 1_24569756, 1_24600014, 1_24617165, 1_24648734, 1_24674300, 1_24694197, 1_24718908, 1_24770415, 1_24775464, 1_24809095, 1_24811604, 1_24824353, 1_24832357, 1_24845499, 1_24848127, 1_24930411, 1_24935775, 1_25002571, 1_25003663, 1_25011189, 1_25015881, 1_25038850, 1_25044429, 1_25162676, 1_25208757, 1_25220896, 1_25248622, 1_25252309, 1_25257182, 1_25264748, 1_25358003, 1_25433318, 1_25443699, 1_25445524, 1_25469761, 1_25493506, 1_25533789, 1_25550082, 1_25628807, 1_25651634, 1_25663724, 1_25754264, 1_25893901, 1_26037407, 1_26057607, 1_26137259, 1_26153863, 1_26203530, 1_26216368, 1_26233428, 1_26246252, 1_26249636, 1_26294660, 1_26324238, 1_26328386, 1_26347893, 1_26352943, 1_26366423, 1_26377821, 1_26395839, 1_26419983, 1_26430306, 1_26457726, 1_26463738, 1_26496166, 1_26510789, 1_26520367, 1_26540091, 1_26574489, 1_26627714, 1_26636923, 1_26674600, 1_26677047, 1_26677426, 1_26681263, 1_26681763, 1_26688454, 1_26748247, 1_26751327, 1_26792120, 1_26807473, 1_26809409, 1_26826132, 1_26832094, 1_26848670, 1_26862595, 1_26875413, 1_26879486, 1_26939988, 1_26943891, 1_26945067, 1_26954259, 1_26956521, 1_26976554, 1_27002239, 1_27008166, 1_27014996, 1_27030965, 1_27054742, 1_27109346, 1_27131263, 1_27206251, 1_27206439, 1_27213286, 1_27251407, 1_27251692, 1_27317422, 1_27336630, 1_27337357, 1_27342406, 1_27343465, 1_27356998, 1_27364950, 1_27370687, 1_27403627, 1_27411634, 1_27412524, 1_27413238, 1_27416682, 1_27420287, 1_27461141, 1_27493509, 1_27507310, 1_27513828, 1_27516024, 1_27536731, 1_27542070, 1_27544322, 1_27591821, 1_27596197, 1_27614366, 1_27652136, 1_27729629, 1_27733961, 1_27750053, 1_27756435, 1_27777879, 1_27782718, 1_27807374, 1_27827089, 1_27868365, 1_27883871, 1_27884798, 1_27896658, 1_27964487, 1_27996777, 1_28013090, 1_28072817, 1_28073071, 1_28075188, 1_28083775, 1_28090050, 1_28095411, 1_28108666, 1_28131167, 1_28133991, 1_28134519, 1_28143624, 1_28157781, 1_28158099, 1_28175335, 1_28187985, 1_28216958, 1_28223561, 1_28261500, 1_28262062, 1_28276387, 1_28350785, 1_28353712, 1_28385346, 1_28420835, 1_28428147, 1_28436330, 1_28460555, 1_28466550, 1_28493165, 1_28538370, 1_28555696, 1_28557994, 1_28570042, 1_28577183, 1_28583982, 1_28603316, 1_28613361, 1_28615905, 1_28655521, 1_28659436, 1_28695570, 1_28709998, 1_28716846, 1_28733690, 1_28757350, 1_28767439, 1_28799073, 1_28832986, 1_28835257, 1_28836511, 1_28842407, 1_28856164, 1_28874863, 1_28903152, 1_28904233, 1_28912269, 1_28929137, 1_28941441, 1_28941936, 1_28945125, 1_28949922, 1_28970860, 1_28978869, 1_28981743, 1_28982586, 1_28982890, 1_28995060, 1_29056952, 1_29064186, 1_29072448, 1_29077318, 1_29078383, 1_29078942, 1_29098785, 1_29118108, 1_29146531, 1_29147081, 1_29173030, 1_29173154, 1_29173397, 1_29182133, 1_29182556, 1_29188158, 1_29198191, 1_29205955, 1_29211889, 1_29219017, 1_29225227, 1_29225830, 1_29233828, 1_29273063, 1_29278087, 1_29294972, 1_29305937, 1_29310817, 1_29311618, 1_29317589, 1_29318962, 1_29355185, 1_29356583, 1_29363210, 1_29401138, 1_29419796, 1_29433139, 1_29436322, 1_29443235, 1_29470867, 1_29472767, 1_29481959, 1_29486309, 1_29526958, 1_29538249, 1_29539826, 1_29544122, 1_29547934, 1_29559838, 1_29567577, 1_29584859, 1_29600281, 1_29602078, 1_29612149, 1_29626757, 1_29637557, 1_29645473, 1_29647251, 1_29686902, 1_29696492, 1_29697103, 1_29715434, 1_29718338, 1_29726170, 1_29735953, 1_29743831, 1_29758971, 1_29759362, 1_29761689, 1_29765420, 1_29766125, 1_29766321, 1_29776482, 1_29803394, 1_29821999, 1_29855800, 1_29871542, 1_29882646, 1_29893116, 1_29895586, 1_29900466, 1_29918556, 1_29933837, 1_29936315, 1_29960400, 1_29967720, 1_29971516, 1_29992280, 1_30018505, 1_30036791, 1_30045494, 1_30049856, 1_30053341, 1_30054626, 1_30061402, 1_30170000, 1_30209554, 1_30279248, 1_30282526, 1_30331661, 1_30333718, 1_30347504, 1_30383358, 1_30384829, 1_30489414, 1_30502995, 1_30525463, 1_30527504, 1_30527884, 1_30530996, 1_30539179, 1_30610656, 1_30615918, 1_30618194, 1_30644859, 1_30645086, 1_30656802, 1_30659491, 1_30667528, 1_30765179, 1_30792008, 1_30797125, 1_30804636, 1_30813312, 1_30873040, 1_30873489, 1_30874001, 1_30874137, 1_30881695, 1_30888021, 1_30907852, 1_30924764, 1_30936197, 1_30939364, 1_30956305, 1_30962772, 1_30981544, 1_30982052, 1_30997233, 1_31029847, 1_31050333, 1_31058562, 1_31058856, 1_31062951, 1_31089763, 1_31090545, 1_31095759, 1_31110876, 1_31111085, 1_31127774, 1_31160453, 1_31160603, 1_31172632, 1_31173116, 1_31179372, 1_31179710, 1_31182850, 1_31260030, 1_31270018, 1_31303773, 1_31311282, 1_31319783, 1_31348644, 1_31361381, 1_31366316, 1_31387900, 1_31441811, 1_31445256, 1_31478446, 1_31491280, 1_31492552, 1_31533411, 1_31608073, 1_31609657, 1_31622710, 1_31660812, 1_31691729, 1_31717893, 1_31724350, 1_31754408, 1_31758565, 1_31770255, 1_31795441, 1_31800650, 1_31806605, 1_31827478, 1_31827860, 1_31842012, 1_31872689, 1_31875238, 1_31879947, 1_31897851, 2_751, 2_12892, 2_15072, 2_19394, 2_26350, 2_47827, 2_58111, 2_58966, 2_119011, 2_133348, 2_154120, 2_161158, 2_184148, 2_214995, 2_216992, 2_232546, 2_247952, 2_248156, 2_278993, 2_279995, 2_280813, 2_294256, 2_313001, 2_353624, 2_367590, 2_375787, 2_375877, 2_401994, 2_403686, 2_414419, 2_414899, 2_418208, 2_450486, 2_465816, 2_481943, 2_486258, 2_489581, 2_509196, 2_525481, 2_549207, 2_550049, 2_550696, 2_556625, 2_565799, 2_566232, 2_574708, 2_612749, 2_645708, 2_650519, 2_661909, 2_667815, 2_700721, 2_710832, 2_731413, 2_742399, 2_755057, 2_756161, 2_768276, 2_768426, 2_770116, 2_817538, 2_817957, 2_829888, 2_852918, 2_867687, 2_887048, 2_903389, 2_968675, 2_968960, 2_993319, 2_995403, 2_1013019, 2_1014316, 2_1028899, 2_1030119, 2_1036388, 2_1064175, 2_1069584, 2_1073460, 2_1090877, 2_1092918, 2_1096032, 2_1118945, 2_1122980, 2_1150625, 2_1151278, 2_1155452, 2_1166534, 2_1176529, 2_1187599, 2_1200303, 2_1203131, 2_1214258, 2_1221879, 2_1280889, 2_1286038, 2_1289585, 2_1290186, 2_1292032, 2_1297935, 2_1299645, 2_1313770, 2_1346220, 2_1354764, 2_1356042, 2_1408145, 2_1415015, 2_1420815, 2_1421939, 2_1427322, 2_1430977, 2_1440254, 2_1440828, 2_1464374, 2_1465842, 2_1493693, 2_1494210, 2_1504590, 2_1538009, 2_1543082, 2_1565049, 2_1584281, 2_1639334, 2_1644789, 2_1646691, 2_1665866, 2_1691313, 2_1716719, 2_1739704, 2_1748516, 2_1758075, 2_1784263, 2_1784876, 2_1789807, 2_1806832, 2_1811306, 2_1817105, 2_1826486, 2_1834063, 2_1834363, 2_1860675, 2_1860978, 2_1941769, 2_1955562, 2_2003381, 2_2004291, 2_2023217, 2_2038195, 2_2040607, 2_2063574, 2_2075113, 2_2083889, 2_2097991, 2_2120354, 2_2146410, 2_2153279, 2_2154182, 2_2179258, 2_2185684, 2_2196405, 2_2200340, 2_2212017, 2_2233403, 2_2276380, 2_2278789, 2_2286033, 2_2293775, 2_2310929, 2_2317239, 2_2330395, 2_2346815, 2_2364247, 2_2379772, 2_2395125, 2_2399607, 2_2413620, 2_2428148, 2_2447355, 2_2452479, 2_2456067, 2_2477405, 2_2504100, 2_2510542, 2_2511444, 2_2512178, 2_2517636, 2_2534961, 2_2552524, 2_2562623, 2_2563584, 2_2578103, 2_2584180, 2_2601603, 2_2618813, 2_2621947, 2_2643212, 2_2646295, 2_2650938, 2_2655872, 2_2666115, 2_2669338, 2_2672203, 2_2674843, 2_2684899, 2_2691335, 2_2695327, 2_2700132, 2_2716132, 2_2723664, 2_2742056, 2_2742796, 2_2747516, 2_2790893, 2_2793827, 2_2797914, 2_2833726, 2_2848862, 2_2855672, 2_2869868, 2_2870540, 2_2881753, 2_2884645, 2_2904563, 2_2912588, 2_2920349, 2_2925971, 2_2926487, 2_2937679, 2_2938176, 2_2961072, 2_2963834, 2_2975782, 2_2981215, 2_2981973, 2_2988667, 2_3002234, 2_3026608, 2_3035362, 2_3040400, 2_3046230, 2_3072710, 2_3072825, 2_3076893, 2_3106171, 2_3110773, 2_3119741, 2_3120775, 2_3121468, 2_3129910, 2_3135322, 2_3139975, 2_3142584, 2_3143270, 2_3161987, 2_3219439, 2_3219561, 2_3238426, 2_3241715, 2_3266000, 2_3301014, 2_3325821, 2_3326214, 2_3352780, 2_3363961, 2_3375181, 2_3375705, 2_3380142, 2_3383597, 2_3393016, 2_3417384, 2_3418757, 2_3435661, 2_3446632, 2_3475225, 2_3494604, 2_3497453, 2_3498932, 2_3514448, 2_3514655, 2_3523463, 2_3530183, 2_3574989, 2_3616959, 2_3664906, 2_3698793, 2_3711942, 2_3716020, 2_3716872, 2_3720273, 2_3731601, 2_3764087, 2_3776136, 2_3780880, 2_3781189, 2_3781686, 2_3783187, 2_3786351, 2_3786711, 2_3790878, 2_3801902, 2_3817079, 2_3818883, 2_3845957, 2_3859627, 2_3910514, 2_3912528, 2_3920529, 2_3953576, 2_3956470, 2_3970892, 2_4007662, 2_4032486, 2_4063646, 2_4065379, 2_4070317, 2_4078448, 2_4083644, 2_4117295, 2_4130562, 2_4160807, 2_4166637, 2_4202553, 2_4203195, 2_4218109, 2_4221609, 2_4243135, 2_4249716, 2_4258752, 2_4271585, 2_4290640, 2_4316734, 2_4325088, 2_4340752, 2_4348051, 2_4350043, 2_4350947, 2_4367692, 2_4382162, 2_4432518, 2_4443523, 2_4443726, 2_4448790, 2_4466005, 2_4466306, 2_4481556, 2_4497906, 2_4533863, 2_4539658, 2_4549845, 2_4564853, 2_4567654, 2_4581823, 2_4587383, 2_4598213, 2_4608823, 2_4623985, 2_4625985, 2_4626829, 2_4643931, 2_4659354, 2_4679504, 2_4679860, 2_4688161, 2_4689337, 2_4708109, 2_4710634, 2_4720662, 2_4737868, 2_4738007, 2_4750538, 2_4763019, 2_4829832, 2_4892463, 2_4900008, 2_4924676, 2_4931313, 2_4941806, 2_5023258, 2_5036738, 2_5052527, 2_5081588, 2_5097005, 2_5113713, 2_5116492, 2_5117719, 2_5134079, 2_5142762, 2_5158907, 2_5163406, 2_5183773, 2_5234182, 2_5234962, 2_5235156, 2_5245536, 2_5245837, 2_5271050, 2_5272865, 2_5301329, 2_5301617, 2_5302875, 2_5303366, 2_5319993, 2_5335533, 2_5336150, 2_5350929, 2_5377472, 2_5385525, 2_5395879, 2_5405130, 2_5415398, 2_5453845, 2_5491077, 2_5499682, 2_5500207, 2_5535402, 2_5550794, 2_5578041, 2_5587907, 2_5588031, 2_5592532, 2_5605238, 2_5606354, 2_5635113, 2_5652242, 2_5661024, 2_5680424, 2_5712085, 2_5718577, 2_5749317, 2_5750393, 2_5757372, 2_5757744, 2_5761040, 2_5774815, 2_5778131, 2_5787983, 2_5801380, 2_5802178, 2_5831339, 2_5874562, 2_5923123, 2_5938635, 2_5950227, 2_5955271, 2_6014119, 2_6076584, 2_6134511, 2_6138313, 2_6164190, 2_6173365, 2_6229321, 2_6256023, 2_6289221, 2_6371584, 2_6441734, 2_6475664, 2_6498151, 2_6536583, 2_6678577, 2_6715931, 2_6742307, 2_6756666, 2_6761699, 2_6773400, 2_6786683, 2_6813776, 2_6995133, 2_7005166, 2_7007087, 2_7014730, 2_7077909, 2_7090833, 2_7118498, 2_7137556, 2_7137578, 2_7316923, 2_7319690, 2_7377418, 2_7403572, 2_7419030, 2_7428625, 2_7454483, 2_7597244, 2_7614583, 2_7628494, 2_7628748, 2_7645707, 2_7697086, 2_7706027, 2_7740815, 2_7750935, 2_7767189, 2_7982822, 2_7998243, 2_8022343, 2_8049883, 2_8061127, 2_8068333, 2_8088979, 2_8153151, 2_8184377, 2_8187641, 2_8198424, 2_8208978, 2_8299101, 2_8396246, 2_8398043, 2_8404228, 2_8404709, 2_8426589, 2_8462380, 2_8468668, 2_8475936, 2_8584806, 2_8603323, 2_8658236, 2_8675221, 2_8694139, 2_8695971, 2_8750321, 2_8755091, 2_8769313, 2_8781390, 2_8810141, 2_8862497, 2_8888919, 2_8892349, 2_8894810, 2_8897805, 2_8900043, 2_8918911, 2_8938469, 2_9005302, 2_9005607, 2_9024643, 2_9064858, 2_9066711, 2_9071200, 2_9099872, 2_9125622, 2_9129146, 2_9132488, 2_9209602, 2_9219349, 2_9235969, 2_9251980, 2_9252650, 2_9257786, 2_9291729, 2_9309854, 2_9379505, 2_9385509, 2_9398672, 2_9416794, 2_9443907, 2_9511658, 2_9523005, 2_9535326, 2_9537809, 2_9538164, 2_9540207, 2_9542295, 2_9590553, 2_9616513, 2_9628934, 2_9647759, 2_9682474, 2_9711043, 2_9711292, 2_9712688, 2_9725836, 2_9776684, 2_9783551, 2_9799113, 2_9813596, 2_9822550, 2_9826791, 2_9848641, 2_9848917, 2_9892387, 2_9999491, 2_10005167, 2_10024195, 2_10050892, 2_10084933, 2_10086434, 2_10088553, 2_10130519, 2_10147686, 2_10216672, 2_10232026, 2_10353111, 2_10358412, 2_10451705, 2_10468056, 2_10477387, 2_10483929, 2_10495206, 2_10523025, 2_10543902, 2_10563975, 2_10587329, 2_10622338, 2_10754292, 2_10754608, 2_10789138, 2_10847589, 2_10855281, 2_10926437, 2_10937301, 2_10937540, 2_10937841, 2_11022524, 2_11080839, 2_11088649, 2_11184680, 2_11184730, 2_11193048, 2_11218995, 2_11251027, 2_11310667, 2_11349107, 2_11384015, 2_11533416, 2_11655459, 2_11713524, 2_11768261, 2_11772738, 2_11793498, 2_11798387, 2_11815655, 2_11841901, 2_11843262, 2_11849870, 2_11925053, 2_11979587, 2_11990306, 2_12069446, 2_12096467, 2_12110315, 2_12110596, 2_12130209, 2_12141647, 2_12148403, 2_12168910, 2_12187121, 2_12204699, 2_12217072, 2_12244978, 2_12256530, 2_12282218, 2_12338247, 2_12341044, 2_12370130, 2_12374368, 2_12400092, 2_12425339, 2_12439668, 2_12452694, 2_12455104, 2_12492004, 2_12531274, 2_12536202, 2_12546098, 2_12557844, 2_12578535, 2_12581982, 2_12613791, 2_12618237, 2_12708941, 2_12746188, 2_12764679, 2_12783627, 2_12862399, 2_12866333, 2_12878766, 2_12955518, 2_12992148, 2_12992670, 2_12992897, 2_12999001, 2_13016632, 2_13028502, 2_13033025, 2_13059413, 2_13070331, 2_13096879, 2_13114726, 2_13121248, 2_13143353, 2_13162080, 2_13212118, 2_13213831, 2_13217674, 2_13268172, 2_13270151, 2_13270530, 2_13319629, 2_13322837, 2_13327140, 2_13349266, 2_13351743, 2_13361080, 2_13392428, 2_13406684, 2_13413655, 2_13416923, 2_13472946, 2_13482533, 2_13493905, 2_13495278, 2_13497861, 2_13538487, 2_13552772, 2_13553188, 2_13565048, 2_13566936, 2_13586190, 2_13597403, 2_13605770, 2_13620950, 2_13637133, 2_13715602, 2_13730018, 2_13748356, 2_13749554, 2_13787529, 2_13787695, 2_13799050, 2_13799278, 2_13804237, 2_13813731, 2_13832137, 2_13833217, 2_13834050, 2_13876752, 2_13907203, 2_13908065, 2_13943722, 2_13965236, 2_14019166, 2_14048732, 2_14050058, 2_14056348, 2_14061521, 2_14096095, 2_14107651, 2_14166071, 2_14195434, 2_14213118, 2_14214001, 2_14226594, 2_14293559, 2_14306917, 2_14308885, 2_14314811, 2_14323189, 2_14338272, 2_14341605, 2_14347348, 2_14352487, 2_14434241, 2_14443090, 2_14449358, 2_14471662, 2_14551824, 2_14569511, 2_14599003, 2_14622872, 2_14622674, 2_14629573, 2_14631286, 2_14654029, 2_14709074, 2_14725374, 2_14738518, 2_14756516, 2_14803376, 2_14808159, 2_14846910, 2_14854866, 2_14886209, 2_14902446, 2_14906076, 2_14910516, 2_14944238, 2_14947186, 2_14947374, 2_14956467, 2_14960027, 2_15011568, 2_15011785, 2_15060693, 2_15099465, 2_15112574, 2_15131842, 2_15139357, 2_15150980, 2_15151265, 2_15172805, 2_15213707, 2_15242588, 2_15295207, 2_15313997, 2_15345961, 2_15373605, 2_15430930, 2_15526772, 2_15551471, 2_15554038, 2_15560536, 2_15634247, 2_15648974, 2_15706604, 2_15751632, 2_15775229, 2_15836475, 2_15842918, 2_15855118, 2_15869787, 2_15895156, 2_15902361, 2_15903570, 2_15910616, 2_15913110, 2_15915335, 2_15940636, 2_15959502, 2_15963920, 2_15979922, 2_16049219, 2_16080767, 2_16107920, 2_16142129, 2_16205752, 2_16314248, 2_16328258, 2_16416580, 2_16438038, 2_16450698, 2_16457294, 2_16460570, 2_16472225, 2_16494793, 2_16502527, 2_16507815, 2_16515821, 2_16516680, 2_16544230, 2_16544673, 2_16545737, 2_16559708, 2_16602964, 2_16606674, 2_16612486, 2_16615926, 2_16657602, 2_16666760, 2_16697615, 2_16732933, 2_16740396, 2_16749253, 2_16804154, 2_16861828, 2_16862255, 2_16867768, 2_16870301, 2_16871621, 2_16873771, 2_16886785, 2_16911727, 2_16935756, 2_16947613, 2_16961739, 2_17000098, 2_17045032, 2_17084970, 2_17110330, 2_17231528, 2_17236559, 2_17244465, 2_17249751, 2_17308937, 2_17376180, 2_17379448, 2_17438723, 2_17534907, 2_17535353, 2_17535602, 2_17540272, 2_17541342, 2_17552551, 2_17576688, 2_17597067, 2_17598920, 2_17607947, 2_17637338, 2_17657806, 2_17723625, 2_17765167, 2_17783437, 2_17874583, 2_17894505, 2_17914818, 2_17916865, 2_17988757, 2_18033104, 2_18049334, 2_18060282, 2_18100575, 2_18113192, 2_18147701, 2_18151997, 2_18215245, 2_18231961, 2_18296218, 2_18344539, 2_18345576, 2_18346589, 2_18351983, 2_18354334, 2_18357313, 2_18370696, 2_18375870, 2_18384738, 2_18398438, 2_18399445, 2_18413075, 2_18418015, 2_18458196, 2_18462376, 2_18465061, 2_18470048, 2_18509711, 2_18514499, 2_18534518, 2_18539345, 2_18565092, 2_18567355, 2_18567667, 2_18577562, 2_18582253, 2_18582700, 2_18590528, 2_18631950, 2_18639814, 2_18646454, 2_18690244, 2_18720400, 2_18743444, 2_18773357, 2_18774790, 2_18797590, 2_18803821, 2_18804665, 2_18810094, 2_18822940, 2_18872333, 2_18907691, 2_18939806, 2_18964754, 2_18989101, 2_19015568, 2_19060310, 2_19076111, 2_19093858, 2_19094480, 2_19094777, 2_19143503, 2_19180647, 2_19200050, 2_19201604, 2_19201690, 2_19206808, 2_19227366, 2_19229301, 2_19231158, 2_19242338, 2_19244695, 2_19297124, 2_19306267, 2_19333904, 2_19337791, 2_19450461, 2_19458635, 2_19469442, 2_19480497, 2_19535031, 2_19598568, 2_19642573, 2_19695473, 2_19748287, 2_19761830, 2_19773864, 2_19803438, 2_19831872, 2_19843363, 2_19878839, 2_19885664, 2_19919719, 2_19939325, 2_19948540, 2_19949132, 2_19960147, 2_20002418, 2_20082807, 2_20100959, 2_20127120, 2_20134821, 2_20147004, 2_20157266, 2_20162563, 2_20172620, 2_20202399, 2_20217848, 2_20221142, 2_20230692, 2_20266077, 2_20272282, 2_20274960, 2_20279909, 2_20299145, 2_20301943, 2_20308582, 2_20316217, 2_20321161, 2_20328937, 2_20330478, 2_20338275, 2_20343762, 2_20355846, 2_20357824, 2_20367055, 2_20385806, 2_20447429, 2_20457998, 2_20460978, 2_20468434, 2_20468643, 2_20476406, 2_20480975, 2_20510051, 2_20537236, 2_20540157, 2_20553864, 2_20554201, 2_20565051, 2_20565207, 2_20580950, 2_20591523, 2_20592257, 2_20622736, 2_20634370, 2_20642976, 2_20655637, 2_20672032, 2_20721732, 2_20724136, 2_20759474, 2_20759516, 2_20760165, 2_20760239, 2_20775120, 2_20780756, 2_20787978, 2_20805859, 2_20818181, 2_20826849, 2_20826930, 2_20832044, 2_20842231, 2_20842923, 2_20864071, 2_20870173, 2_20917745, 2_20918807, 2_20920979, 2_20927583, 2_20927831, 2_20973532, 2_20997983, 2_21002459, 2_21031640, 2_21076928, 2_21080651, 2_21093991, 2_21101711, 2_21155289, 2_21161115, 2_21163286, 2_21165406, 2_21166242, 2_21175679, 2_21182052, 2_21187424, 2_21192305, 2_21221333, 2_21229614, 2_21233798, 2_21287430, 2_21289328, 2_21292044, 2_21292743, 2_21300204, 2_21303614, 2_21325012, 2_21340808, 2_21348141, 2_21350354, 2_21367534, 2_21373201, 2_21383596, 2_21399682, 2_21425055, 2_21512836, 2_21524909, 2_21539231, 2_21539743, 2_21546194, 2_21570524, 2_21591760, 2_21605146, 2_21608806, 2_21619125, 2_21627551, 2_21634016, 2_21663258, 2_21714261, 2_21736948, 2_21738429, 2_21749368, 2_21799983, 2_21809011, 2_21809257, 2_21809701, 2_21848680, 2_21865764, 2_21882515, 2_21885760, 2_21889165, 2_21916859, 2_21973673, 2_21995236, 2_22013008, 2_22014368, 2_22030254, 2_22031379, 2_22037131, 2_22039457, 2_22044707, 2_22075773, 2_22076744, 2_22080363, 2_22081096, 2_22083352, 2_22086416, 2_22092618, 2_22095530, 2_22096924, 2_22106324, 2_22110332, 2_22112097, 2_22130332, 2_22142747, 2_22147208, 2_22147911, 2_22158516, 2_22183454, 2_22210115, 2_22239559, 2_22259466, 2_22270316, 2_22272673, 2_22280569, 2_22307132, 2_22307422, 2_22316408, 2_22316965, 2_22338200, 2_22349546, 2_22364195, 2_22367924, 2_22374194, 2_22408508, 2_22412240, 2_22413389, 2_22420624, 2_22462380, 2_22486707, 2_22499370, 2_22516864, 2_22531936, 2_22542411, 2_22561807, 2_22577420, 2_22586297, 2_22591500, 2_22591599, 2_22613156, 2_22650858, 2_22687210, 2_22723367, 2_22737907, 2_22744937, 2_22766209, 2_22766505, 2_22805961, 2_22835188, 2_22857770, 2_22867825, 2_22962879, 2_22969091, 2_22974256, 2_23000077, 2_23003457, 2_23017250, 2_23053480, 2_23057217, 2_23057586, 2_23107877, 2_23113303, 2_23118814, 2_23119705, 2_23119980, 2_23126347, 2_23175209, 2_23176326, 2_23209474, 2_23211174, 2_23212323, 2_23217579, 2_23222606, 2_23224684, 2_23232558, 2_23239821, 2_23252676, 2_23295811, 2_23311235, 2_23328110, 2_23336978, 2_23337283, 2_23337733, 2_23344737, 2_23351131, 2_23360938, 2_23399744, 2_23400417, 2_23402703, 2_23407654, 2_23411477, 2_23413588, 2_23428073, 2_23428139, 2_23440219, 2_23442478, 2_23454567, 2_23459251, 2_23471501, 2_23501632, 2_23505223, 2_23515655, 2_23579838, 2_23593489, 2_23609196, 2_23617255, 2_23626688, 2_23641234, 2_23644729, 2_23663678, 2_23702632, 2_23731693, 2_23754158, 2_23758265, 2_23764461, 2_23780257, 2_23800240, 2_23802692, 2_23820669, 2_23833954, 3_6168, 3_11393, 3_72666, 3_198118, 3_221314, 3_226745, 3_242181, 3_272914, 3_322307, 3_324522, 3_375605, 3_534125, 3_584650, 3_679544, 3_703784, 3_724519, 3_730975, 3_813463, 3_852085, 3_859490, 3_864676, 3_867141, 3_925379, 3_929748, 3_1039391, 3_1059230, 3_1103156, 3_1109199, 3_1118440, 3_1132569, 3_1320887, 3_1353399, 3_1382298, 3_1392515, 3_1409288, 3_1474458, 3_1482068, 3_1533869, 3_1571696, 3_1735453, 3_1833740, 3_1867540, 3_1880578, 3_1888396, 3_1896298, 3_1911367, 3_1941226, 3_1944929, 3_2014282, 3_2060785, 3_2062567, 3_2090379, 3_2095125, 3_2136364, 3_2140475, 3_2256202, 3_2264184, 3_2269398, 3_2275689, 3_2350322, 3_2398499, 3_2401494, 3_2453688, 3_2472018, 3_2480632, 3_2531952, 3_2544448, 3_2548942, 3_2566693, 3_2604277, 3_2606959, 3_2663243, 3_2693360, 3_2698074, 3_2698392, 3_2700018, 3_2702853, 3_2731508, 3_2743491, 3_2901044, 3_2912086, 3_2932940, 3_2937490, 3_2937739, 3_2953029, 3_2955722, 3_2972144, 3_2994404, 3_3031945, 3_3054594, 3_3068196, 3_3122970, 3_3123195, 3_3149182, 3_3172902, 3_3181118, 3_3198038, 3_3199670, 3_3239823, 3_3241009, 3_3263477, 3_3286516, 3_3302703, 3_3330394, 3_3334929, 3_3342518, 3_3344190, 3_3365269, 3_3370037, 3_3374130, 3_3385774, 3_3396335, 3_3396728, 3_3400139, 3_3401301, 3_3410258, 3_3445587, 3_3453386, 3_3453979, 3_3517912, 3_3538352, 3_3568936, 3_3589215, 3_3594059, 3_3597968, 3_3607369, 3_3609825, 3_3615916, 3_3620842, 3_3623158, 3_3630364, 3_3638485, 3_3648936, 3_3680931, 3_3696073, 3_3744319, 3_3757805, 3_3760702, 3_3802387, 3_3812220, 3_3817372, 3_3878197, 3_3905988, 3_3911155, 3_3934007, 3_3993307, 3_4003306, 3_4036474, 3_4061766, 3_4068110, 3_4072856, 3_4105064, 3_4106471, 3_4130607, 3_4133470, 3_4139082, 3_4263523, 3_4272257, 3_4321029, 3_4337480, 3_4359876, 3_4390875, 3_4391254, 3_4426636, 3_4428519, 3_4497220, 3_4507553, 3_4508549, 3_4514953, 3_4548023, 3_4550179, 3_4622505, 3_4648153, 3_4651523, 3_4651794, 3_4656381, 3_4672763, 3_4699944, 3_4710541, 3_4714288, 3_4724835, 3_4801966, 3_4832620, 3_4835219, 3_4889905, 3_4899894, 3_4900681, 3_4904916, 3_4905044, 3_4921103, 3_4939810, 3_4942934, 3_4965568, 3_4978718, 3_4989445, 3_5004551, 3_5009731, 3_5017642, 3_5068752, 3_5128800, 3_5142532, 3_5171185, 3_5204322, 3_5243255, 3_5243798, 3_5260948, 3_5272082, 3_5274996, 3_5332830, 3_5373071, 3_5378005, 3_5383546, 3_5386812, 3_5405543, 3_5407594, 3_5416814, 3_5426802, 3_5429440, 3_5452052, 3_5490855, 3_5495081, 3_5505878, 3_5524779, 3_5540276, 3_5576890, 3_5580932, 3_5591143, 3_5592848, 3_5596592, 3_5596890, 3_5598946, 3_5599202, 3_5628240, 3_5659653, 3_5697575, 3_5699509, 3_5711009, 3_5768761, 3_5769216, 3_5775070, 3_5784119, 3_5795531, 3_5807280, 3_5816035, 3_5867876, 3_5881102, 3_5891544, 3_5900296, 3_5901985, 3_5906435, 3_5912575, 3_5923290, 3_5925372, 3_5935187, 3_5943759, 3_5959368, 3_5967438, 3_5968826, 3_5978532, 3_5981933, 3_5992877, 3_6001525, 3_6006002, 3_6065606, 3_6066525, 3_6077229, 3_6106480, 3_6110514, 3_6149995, 3_6153479, 3_6156364, 3_6185029, 3_6194547, 3_6226555, 3_6232424, 3_6238328, 3_6259580, 3_6260857, 3_6323510, 3_6336322, 3_6340741, 3_6400587, 3_6428160, 3_6449470, 3_6512686, 3_6547531, 3_6555951, 3_6560389, 3_6611298, 3_6616405, 3_6622823, 3_6676926, 3_6682453, 3_6692778, 3_6733953, 3_6745004, 3_6755162, 3_6792370, 3_6821985, 3_6847598, 3_6860814, 3_6878337, 3_6886892, 3_6904607, 3_6914706, 3_6955766, 3_7080485, 3_7101549, 3_7101800, 3_7113075, 3_7123662, 3_7159050, 3_7207542, 3_7249686, 3_7251726, 3_7257914, 3_7279057, 3_7285311, 3_7303133, 3_7304953, 3_7325725, 3_7342471, 3_7396391, 3_7398562, 3_7418007, 3_7422878, 3_7435394, 3_7457708, 3_7530013, 3_7555031, 3_7556515, 3_7574205, 3_7582900, 3_7587828, 3_7618659, 3_7642502, 3_7694325, 3_7706414, 3_7720679, 3_7724900, 3_7761815, 3_7764135, 3_7788793, 3_7821254, 3_7832523, 3_7854004, 3_7876319, 3_7897877, 3_7977226, 3_7984726, 3_8021349, 3_8044882, 3_8046426, 3_8088029, 3_8111067, 3_8129050, 3_8145572, 3_8159126, 3_8166149, 3_8170339, 3_8248916, 3_8250119, 3_8275273, 3_8278942, 3_8287571, 3_8302541, 3_8436171, 3_8490789, 3_8509407, 3_8515669, 3_8590578, 3_8598963, 3_8618799, 3_8625624, 3_8638214, 3_8641507, 3_8696939, 3_8708947, 3_8752281, 3_8766114, 3_8797634, 3_8797888, 3_8908668, 3_9014390, 3_9070876, 3_9148837, 3_9149362, 3_9150607, 3_9206408, 3_9223958, 3_9243539, 3_9245842, 3_9264679, 3_9358362, 3_9359640, 3_9371318, 3_9374084, 3_9386226, 3_9390031, 3_9394715, 3_9397746, 3_9474284, 3_9507854, 3_9579826, 3_9590700, 3_9614375, 3_9623636, 3_9625201, 3_9630428, 3_9645940, 3_9652313, 3_9705499, 3_9712783, 3_9717025, 3_9725009, 3_9740952, 3_9784528, 3_9812428, 3_9832181, 3_9851429, 3_9858503, 3_9862689, 3_9934582, 3_10013774, 3_10151674, 3_10171833, 3_10254223, 3_10316270, 3_10341610, 3_10352947, 3_10366313, 3_10396848, 3_10440486, 3_10505614, 3_10511480, 3_10524548, 3_10526314, 3_10533471, 3_10576133, 3_10582016, 3_10587372, 3_10630831, 3_10669767, 3_10697745, 3_10706620, 3_10755373, 3_10777820, 3_10813271, 3_10838376, 3_10855605, 3_10892610, 3_10967713, 3_10968699, 3_10986329, 3_11005834, 3_11024086, 3_11064313, 3_11070862, 3_11153489, 3_11264911, 3_11292683, 3_11369091, 3_11394766, 3_11400013, 3_11421229, 3_11437862, 3_11439757, 3_11452697, 3_11548156, 3_11567294, 3_11569837, 3_11634618, 3_11641770, 3_11645248, 3_11676615, 3_11687640, 3_11693874, 3_11713738, 3_11751993, 3_11766231, 3_11776541, 3_11784371, 3_11821685, 3_11826336, 3_11911875, 3_11914240, 3_11914371, 3_11936085, 3_11965602, 3_11982112, 3_12005153, 3_12013157, 3_12013572, 3_12023994, 3_12086095, 3_12101797, 3_12122703, 3_12152479, 3_12159502, 3_12191576, 3_12237054, 3_12240792, 3_12267077, 3_12280656, 3_12301460, 3_12335569, 3_12341725, 3_12396003, 3_12508370, 3_12590899, 3_12597059, 3_12598929, 3_12609788, 3_12657813, 3_12675429, 3_12704470, 3_12719143, 3_12780142, 3_12837296, 3_12838125, 3_12861645, 3_12935285, 3_13004063, 3_13006880, 3_13025278, 3_13044253, 3_13062541, 3_13076082, 3_13079625, 3_13083926, 3_13088341, 3_13090859, 3_13093326, 3_13108160, 3_13182441, 3_13196544, 3_13212004, 3_13291518, 3_13302699, 3_13305592, 3_13311387, 3_13422848, 3_13432599, 3_13451956, 3_13452675, 3_13454089, 3_13463887, 3_13489686, 3_13490754, 3_13521841, 3_13528126, 3_13531142, 3_13531261, 3_13592166, 3_13596677, 3_13596992, 3_13605878, 3_13616084, 3_13766459, 3_13770864, 3_13803327, 3_13811048, 3_13813361, 3_13836560, 3_13861879, 3_13869789, 3_13872924, 3_13913123, 3_13915465, 3_13947438, 3_13977501, 3_14008207, 3_14025741, 3_14040449, 3_14126338, 3_14181122, 3_14205929, 3_14213669, 3_14253153, 3_14319182, 3_14368383, 3_14373838, 3_14383463, 3_14385343, 3_14448198, 3_14451404, 3_14477074, 3_14511404, 3_14557301, 3_14565567, 3_14603693, 3_14669050, 3_14673745, 3_14702875, 3_14713006, 3_14794605, 3_14806951, 3_14832650, 3_14844218, 3_14897393, 3_14948179, 3_14959012, 3_14996091, 3_15017262, 3_15038469, 3_15063138, 3_15088452, 3_15153361, 3_15158884, 3_15220992, 3_15222567, 3_15226784, 3_15278996, 3_15320772, 3_15333887, 3_15345095, 3_15364007, 3_15427361, 3_15468322, 3_15492284, 3_15594828, 3_15660310, 3_15666672, 3_15714428, 3_15717681, 3_15753326, 3_15832318, 3_15875637, 3_15918454, 3_15922843, 3_15968409, 3_15968996, 3_16008059, 3_16013921, 3_16017788, 3_16029824, 3_16030806, 3_16061995, 3_16126803, 3_16129497, 3_16133197, 3_16136128, 3_16141704, 3_16143387, 3_16214977, 3_16271342, 3_16301588, 3_16378001, 3_16390219, 3_16571388, 3_16574219, 3_16627513, 3_16650904, 3_16660012, 3_16664521, 3_16676063, 3_16723469, 3_16732383, 3_16762233, 3_16773411, 3_16774375, 3_16786533, 3_16794002, 3_16816983, 3_16826636, 3_16832681, 3_16837805, 3_16851923, 3_16855677, 3_16858384, 3_16943268, 3_16947010, 3_16952427, 3_16958534, 3_16966337, 3_16967545, 3_16969448, 3_16974735, 3_17032221, 3_17064491, 3_17065560, 3_17078360, 3_17088691, 3_17090880, 3_17091324, 3_17095924, 3_17100767, 3_17123809, 3_17154145, 3_17162306, 3_17171464, 3_17188189, 3_17188265, 3_17299081, 3_17312217, 3_17330972, 3_17332381, 3_17403408, 3_17413444, 3_17456750, 3_17459745, 3_17476679, 3_17491289, 3_17497496, 3_17526395, 3_17584804, 3_17611442, 3_17629289, 3_17687475, 3_17715027, 3_17749923, 3_17750947, 3_17765804, 3_17779181, 3_17785922, 3_17793614, 3_17805330, 3_17837436, 3_17839007, 3_17870721, 3_17898603, 3_17944963, 3_17979555, 3_18002808, 3_18032966, 3_18033505, 3_18045417, 3_18056760, 3_18081455, 3_18167870, 3_18178994, 3_18186215, 3_18205272, 3_18272622, 3_18279335, 3_18285142, 3_18316671, 3_18327809, 3_18346105, 3_18414344, 3_18431358, 3_18440098, 3_18440304, 3_18444622, 3_18451222, 3_18472847, 3_18474011, 3_18514234, 3_18547814, 3_18547932, 3_18553962, 3_18555500, 3_18571572, 3_18572166, 3_18574990, 3_18575277, 3_18597385, 3_18619566, 3_18622422, 3_18626998, 3_18629621, 3_18642056, 3_18654464, 3_18739291, 3_18747719, 3_18758306, 3_18759413, 3_18776055, 3_18782020, 3_18788795, 3_18798320, 3_18802686, 3_18841175, 3_18864355, 3_18865532, 3_18926230, 3_18936595, 3_18936967, 3_18947182, 3_18956223, 3_18962624, 3_18964733, 3_18970386, 3_18992086, 3_19018247, 3_19018457, 3_19024212, 3_19028033, 3_19035222, 3_19087915, 3_19096464, 3_19098998, 3_19109102, 3_19119584, 3_19120731, 3_19140207, 3_19142961, 3_19175231, 3_19241304, 3_19274809, 3_19287574, 3_19291092, 3_19298865, 3_19330723, 3_19334879, 3_19353058, 3_19359868, 3_19385524, 3_19386302, 3_19414433, 3_19494910, 3_19506147, 3_19521520, 3_19523225, 3_19531276, 3_19545873, 3_19546411, 3_19547789, 3_19557394, 3_19563257, 3_19576114, 3_19577260, 3_19589993, 3_19610543, 3_19614825, 3_19615135, 3_19626125, 3_19689956, 3_19715113, 3_19720875, 3_19721117, 3_19722519, 3_19768097, 3_19771053, 3_19774535, 3_19792574, 3_19812039, 3_19821388, 3_19869665, 3_19881333, 3_19881628, 3_19931762, 3_19942487, 3_19965044, 3_19989754, 3_20002434, 3_20027597, 3_20082771, 3_20151655, 3_20165756, 3_20180589, 3_20182558, 3_20200361, 3_20200701, 3_20209020, 3_20251343, 3_20260087, 3_20305862, 3_20313339, 3_20355685, 3_20358253, 3_20410449, 3_20410753, 3_20442712, 3_20443691, 3_20522196, 3_20525230, 3_20526126, 3_20531986, 3_20537957, 3_20555832, 3_20559887, 3_20619886, 3_20634147, 3_20639681, 3_20648374, 3_20689335, 3_20835069, 3_20858514, 3_20858971, 3_20871452, 3_20917062, 3_20930412, 3_20963910, 3_20990953, 3_20997938, 3_21021450, 3_21048872, 3_21098547, 3_21108581, 3_21120821, 3_21138536, 3_21144241, 3_21238833, 3_21300822, 3_21307868, 3_21317192, 3_21342681, 3_21351137, 3_21359010, 3_21367618, 3_21367893, 3_21368181, 3_21375362, 3_21405332, 3_21429293, 3_21458645, 3_21477306, 3_21484495, 3_21505170, 3_21517886, 3_21528036, 3_21546142, 3_21579981, 3_21596102, 3_21630081, 3_21651850, 3_21707281, 3_21707671, 3_21818584, 3_21838872, 3_21846348, 3_21849462, 3_21852287, 3_21896903, 3_21986372, 3_22022032, 3_22022094, 3_22040222, 3_22047081, 3_22109745, 3_22110267, 3_22111086, 3_22117169, 3_22123166, 3_22131103, 3_22142712, 3_22148022, 3_22151097, 3_22157494, 3_22157833, 3_22193924, 3_22198640, 3_22200828, 3_22202185, 3_22202648, 3_22206422, 3_22207792, 3_22222155, 3_22224857, 3_22278692, 3_22293495, 3_22296357, 3_22301567, 3_22304258, 3_22344469, 3_22353675, 3_22358487, 3_22374610, 3_22382994, 3_22384979, 3_22390810, 3_22395892, 3_22398094, 3_22399588, 3_22401794, 3_22402145, 3_22408923, 3_22416964, 3_22423619, 3_22429710, 3_22433578, 3_22483603, 3_22496798, 3_22507854, 3_22509723, 3_22524213, 3_22524476, 3_22529943, 3_22530165, 3_22532890, 3_22540316, 3_22580343, 3_22593812, 3_22612136, 3_22635734, 3_22643996, 3_22672747, 3_22702252, 3_22720355, 3_22726129, 3_22729572, 3_22745379, 3_22751729, 3_22769621, 3_22776737, 3_22789165, 3_22789609, 3_22805943, 3_22807201, 3_22807537, 3_22808119, 3_22809801, 3_22811749, 3_22847972, 3_22868093, 3_22898747, 3_22899448, 3_22908871, 3_22914652, 3_22932623, 3_22935832, 3_22940972, 3_22954716, 3_22969420, 3_22969702, 3_22970024, 3_22970136, 3_22972029, 3_23006773, 3_23010037, 3_23013902, 3_23021856, 3_23037487, 3_23040055, 3_23050651, 3_23116507, 3_23129560, 3_23178671, 3_23180773, 3_23199548, 3_23205796, 3_23211297, 3_23223939, 3_23226693, 3_23227314, 3_23234836, 3_23250750, 3_23260531, 3_23260933, 3_23269286, 3_23281764, 3_23282866, 3_23295326, 3_23296137, 3_23308844, 3_23319194, 3_23354390, 3_23366595, 3_23367715, 3_23385965, 3_23391562, 3_23393145, 3_23467368, 3_23473763, 3_23486375, 3_23488048, 3_23518637, 3_23564499, 3_23565096, 3_23581023, 3_23592624, 3_23652868, 3_23657851, 3_23663486, 3_23667640, 3_23669065, 3_23674024, 3_23691724, 3_23694563, 3_23701076, 3_23743834, 3_23746953, 3_23796822, 3_23799257, 3_23804477, 3_23805240, 3_23843083, 3_23866001, 3_23879434, 3_23894082, 3_23906819, 3_23911340, 3_23914741, 3_23924438, 3_23937364, 3_23946602, 3_23981181, 3_24008479, 3_24032223, 3_24040365, 3_24052157, 3_24061906, 3_24062240, 3_24075638, 3_24090299, 3_24129219, 4_14944, 4_15425, 4_23346, 4_42720, 4_48135, 4_48742, 4_54172, 4_57419, 4_61730, 4_66019, 4_78956, 4_79667, 4_79728, 4_83848, 4_84258, 4_98774, 4_115533, 4_173497, 4_178526, 4_180448, 4_202176, 4_210383, 4_246685, 4_246820, 4_286801, 4_289617, 4_297943, 4_309355, 4_314777, 4_316104, 4_335161, 4_335588, 4_341493, 4_361558, 4_366989, 4_375637, 4_391817, 4_403096, 4_435221, 4_435624, 4_441478, 4_445637, 4_453128, 4_518203, 4_523074, 4_556459, 4_564703, 4_564887, 4_573054, 4_577000, 4_584353, 4_585817, 4_593459, 4_611335, 4_639163, 4_656936, 4_658560, 4_665936, 4_666278, 4_672218, 4_678263, 4_700579, 4_700799, 4_701373, 4_707983, 4_708248, 4_708451, 4_717034, 4_730419, 4_732298, 4_734432, 4_744753, 4_761203, 4_764985, 4_815171, 4_815670, 4_857482, 4_868117, 4_869545, 4_904286, 4_954361, 4_957982, 4_958708, 4_970565, 4_973797, 4_983514, 4_997395, 4_1001216, 4_1003759, 4_1004291, 4_1075185, 4_1080040, 4_1080207, 4_1090328, 4_1092378, 4_1121174, 4_1124589, 4_1130017, 4_1133212, 4_1159316, 4_1175801, 4_1178344, 4_1178640, 4_1183689, 4_1186539, 4_1187939, 4_1190261, 4_1200410, 4_1203291, 4_1210052, 4_1217022, 4_1237381, 4_1239575, 4_1253727, 4_1261339, 4_1270295, 4_1280685, 4_1298425, 4_1322109, 4_1326044, 4_1353361, 4_1362312, 4_1362854, 4_1382446, 4_1382759, 4_1390901, 4_1486346, 4_1488930, 4_1507983, 4_1510572, 4_1520447, 4_1543246, 4_1546125, 4_1558634, 4_1569913, 4_1588564, 4_1590058, 4_1592741, 4_1647274, 4_1649876, 4_1679154, 4_1728717, 4_1730438, 4_1738238, 4_1738348, 4_1760685, 4_1767347, 4_1826696, 4_1873308, 4_1875213, 4_1892811, 4_1902493, 4_1925444, 4_1929096, 4_1943015, 4_1963162, 4_2019190, 4_2029336, 4_2041113, 4_2044738, 4_2049160, 4_2055574, 4_2060032, 4_2067376, 4_2074229, 4_2075665, 4_2086284, 4_2101241, 4_2105396, 4_2105922, 4_2119844, 4_2121825, 4_2147958, 4_2161282, 4_2166811, 4_2178027, 4_2205863, 4_2209101, 4_2211997, 4_2230523, 4_2252704, 4_2256431, 4_2303493, 4_2311043, 4_2315237, 4_2316282, 4_2362207, 4_2382375, 4_2393079, 4_2395789, 4_2398819, 4_2413957, 4_2479188, 4_2496183, 4_2496788, 4_2505308, 4_2542086, 4_2551171, 4_2562058, 4_2562314, 4_2564446, 4_2575737, 4_2577482, 4_2595374, 4_2599535, 4_2630328, 4_2643660, 4_2647583, 4_2659762, 4_2696883, 4_2730410, 4_2751218, 4_2768895, 4_2795990, 4_2805157, 4_2807944, 4_2821283, 4_2835541, 4_2840902, 4_2846115, 4_2928163, 4_2928404, 4_2944639, 4_2968187, 4_2978260, 4_2988683, 4_2988761, 4_2989928, 4_3029568, 4_3034589, 4_3036563, 4_3044962, 4_3045396, 4_3059195, 4_3115931, 4_3119492, 4_3127759, 4_3136034, 4_3140638, 4_3144888, 4_3156053, 4_3161051, 4_3173143, 4_3184403, 4_3194181, 4_3194494, 4_3231128, 4_3232302, 4_3236835, 4_3246512, 4_3247242, 4_3254841, 4_3255400, 4_3293383, 4_3294629, 4_3302260, 4_3315971, 4_3322365, 4_3329298, 4_3333286, 4_3365888, 4_3371857, 4_3385687, 4_3387365, 4_3398791, 4_3414318, 4_3424591, 4_3464081, 4_3467601, 4_3494366, 4_3507110, 4_3518248, 4_3565067, 4_3566144, 4_3570877, 4_3579415, 4_3594118, 4_3595159, 4_3596358, 4_3603797, 4_3608595, 4_3613879, 4_3615238, 4_3644866, 4_3652825, 4_3659280, 4_3659540, 4_3698047, 4_3702073, 4_3711991, 4_3720789, 4_3742692, 4_3751702, 4_3759554, 4_3762533, 4_3771754, 4_3790571, 4_3802225, 4_3828222, 4_3873953, 4_3931498, 4_3939341, 4_3953177, 4_3963180, 4_3974604, 4_4006428, 4_4094576, 4_4149812, 4_4152701, 4_4153126, 4_4159514, 4_4163544, 4_4174359, 4_4192002, 4_4214472, 4_4232492, 4_4237140, 4_4247000, 4_4248914, 4_4252873, 4_4252932, 4_4258573, 4_4264682, 4_4288509, 4_4305102, 4_4307645, 4_4321722, 4_4417037, 4_4421959, 4_4425483, 4_4426854, 4_4430585, 4_4440482, 4_4469764, 4_4503854, 4_4529053, 4_4536847, 4_4543117, 4_4554975, 4_4566430, 4_4566721, 4_4567985, 4_4574525, 4_4580891, 4_4586945, 4_4589848, 4_4590342, 4_4592909, 4_4636782, 4_4645814, 4_4647379, 4_4663359, 4_4685123, 4_4723016, 4_4733654, 4_4734601, 4_4737006, 4_4757401, 4_4770205, 4_4801294, 4_4806723, 4_4820616, 4_4821864, 4_4831765, 4_4835581, 4_4838019, 4_4839456, 4_4850722, 4_4891561, 4_4912782, 4_4944168, 4_4948332, 4_4948620, 4_4949946, 4_4950362, 4_4957881, 4_4975209, 4_4977887, 4_5003460, 4_5003771, 4_5006927, 4_5032031, 4_5057192, 4_5057498, 4_5132547, 4_5155869, 4_5175222, 4_5203344, 4_5209373, 4_5224697, 4_5283337, 4_5285774, 4_5288347, 4_5307523, 4_5316800, 4_5321905, 4_5326769, 4_5330660, 4_5331229, 4_5331311, 4_5346387, 4_5349583, 4_5375640, 4_5377013, 4_5384140, 4_5388121, 4_5389460, 4_5436994, 4_5447524, 4_5459133, 4_5473854, 4_5506157, 4_5511286, 4_5537699, 4_5558536, 4_5600514, 4_5605057, 4_5627195, 4_5636261, 4_5645387, 4_5662596, 4_5670199, 4_5674836, 4_5690169, 4_5709866, 4_5725737, 4_5739161, 4_5751196, 4_5754499, 4_5767206, 4_5780550, 4_5782625, 4_5800629, 4_5821208, 4_5821265, 4_5822300, 4_5823284, 4_5825120, 4_5828205, 4_5858114, 4_5858403, 4_5874351, 4_5900247, 4_5933782, 4_5949165, 4_5962107, 4_5969662, 4_5979299, 4_6066573, 4_6099532, 4_6106717, 4_6116222, 4_6174462, 4_6218084, 4_6226807, 4_6241541, 4_6249937, 4_6251117, 4_6254193, 4_6254429, 4_6315683, 4_6418351, 4_6454818, 4_6470578, 4_6473128, 4_6513850, 4_6518450, 4_6527093, 4_6555429, 4_6570808, 4_6573612, 4_6664571, 4_6676014, 4_6700192, 4_6703395, 4_6710840, 4_6711179, 4_6724611, 4_6748123, 4_6794339, 4_6807965, 4_6821241, 4_6840139, 4_6858822, 4_6865555, 4_6890465, 4_6947770, 4_7020304, 4_7020536, 4_7034977, 4_7041075, 4_7061365, 4_7073589, 4_7079244, 4_7112114, 4_7121579, 4_7132497, 4_7135145, 4_7155911, 4_7180744, 4_7214171, 4_7227493, 4_7250227, 4_7250325, 4_7266811, 4_7267401, 4_7293741, 4_7299464, 4_7308058, 4_7397136, 4_7420412, 4_7449231, 4_7457872, 4_7465320, 4_7579214, 4_7590202, 4_7630980, 4_7666851, 4_7668183, 4_7675958, 4_7681071, 4_7683188, 4_7698187, 4_7747194, 4_7753888, 4_7756770, 4_7760075, 4_7777289, 4_7785748, 4_7786919, 4_7828368, 4_7837330, 4_7847902, 4_7851424, 4_7875716, 4_7921435, 4_7922646, 4_7941360, 4_7947752, 4_7976489, 4_7998492, 4_8039329, 4_8075337, 4_8077152, 4_8115555, 4_8210828, 4_8211426, 4_8227173, 4_8251251, 4_8380368, 4_8391644, 4_8402220, 4_8437107, 4_8489806, 4_8503964, 4_8525893, 4_8527295, 4_8529199, 4_8569580, 4_8572350, 4_8601818, 4_8608802, 4_8618978, 4_8621233, 4_8631084, 4_8687175, 4_8716701, 4_8733737, 4_8822622, 4_8840981, 4_8856087, 4_8864300, 4_8880058, 4_8880412, 4_8910716, 4_8946844, 4_8957217, 4_9004856, 4_9010144, 4_9116373, 4_9118352, 4_9119932, 4_9125460, 4_9137076, 4_9196017, 4_9255121, 4_9266282, 4_9362276, 4_9382541, 4_9443049, 4_9484708, 4_9487654, 4_9558199, 4_9562326, 4_9573009, 4_9700380, 4_9718327, 4_9730847, 4_9745597, 4_9771324, 4_9804382, 4_9805524, 4_9881761, 4_9914590, 4_9927289, 4_9973751, 4_10018750, 4_10018965, 4_10022140, 4_10033939, 4_10063081, 4_10068804, 4_10084782, 4_10090101, 4_10128381, 4_10145283, 4_10229285, 4_10230699, 4_10260473, 4_10260740, 4_10270790, 4_10342280, 4_10354154, 4_10360666, 4_10371454, 4_10394981, 4_10402368, 4_10487172, 4_10503821, 4_10510651, 4_10527263, 4_10537679, 4_10683703, 4_10719499, 4_10732645, 4_10738547, 4_10744708, 4_10759429, 4_10759996, 4_10773584, 4_10798978, 4_10816492, 4_10834123, 4_10860786, 4_10880409, 4_10931053, 4_10937734, 4_10977020, 4_10994272, 4_10998041, 4_11012917, 4_11055668, 4_11059603, 4_11109800, 4_11140084, 4_11148484, 4_11173598, 4_11174247, 4_11240677, 4_11248868, 4_11287126, 4_11287383, 4_11339622, 4_11356180, 4_11371190, 4_11371722, 4_11394538, 4_11403845, 4_11406120, 4_11442878, 4_11466158, 4_11548995, 4_11549193, 4_11556283, 4_11608099, 4_11637703, 4_11663159, 4_11674290, 4_11702344, 4_11784326, 4_11822282, 4_11882136, 4_11894799, 4_11903575, 4_11922198, 4_11928842, 4_11972857, 4_12019419, 4_12033245, 4_12063851, 4_12083189, 4_12132238, 4_12150017, 4_12190326, 4_12198068, 4_12198235, 4_12227523, 4_12229358, 4_12232068, 4_12235264, 4_12247354, 4_12271385, 4_12282719, 4_12309771, 4_12323921, 4_12332564, 4_12492852, 4_12520836, 4_12522294, 4_12529835, 4_12539352, 4_12553209, 4_12561223, 4_12597379, 4_12657384, 4_12661332, 4_12663024, 4_12702243, 4_12708804, 4_12713362, 4_12714657, 4_12716089, 4_12719101, 4_12754696, 4_12786937, 4_12861638, 4_12864803, 4_12871446, 4_12891371, 4_12906673, 4_12915346, 4_12929527, 4_12936262, 4_12969544, 4_12991267, 4_13007534, 4_13033167, 4_13038864, 4_13052040, 4_13094327, 4_13094406, 4_13167397, 4_13181095, 4_13186847, 4_13201443, 4_13218862, 4_13242287, 4_13290700, 4_13300473, 4_13313324, 4_13323243, 4_13328178, 4_13338397, 4_13351438, 4_13370248, 4_13403696, 4_13443125, 4_13443344, 4_13458418, 4_13460089, 4_13474809, 4_13495833, 4_13496655, 4_13496931, 4_13564460, 4_13606990, 4_13610790, 4_13612508, 4_13633307, 4_13647132, 4_13653765, 4_13677120, 4_13677339, 4_13704413, 4_13706626, 4_13733680, 4_13739422, 4_13747693, 4_13782302, 4_13789422, 4_13801381, 4_13846368, 4_13871758, 4_13894933, 4_13895506, 4_13914195, 4_13917402, 4_13936142, 4_13937588, 4_13949216, 4_13982944, 4_14003024, 4_14032132, 4_14078959, 4_14197337, 4_14205027, 4_14215454, 4_14216653, 4_14223905, 4_14240845, 4_14244532, 4_14277558, 4_14291721, 4_14295316, 4_14297234, 4_14311911, 4_14315665, 4_14337473, 4_14397442, 4_14406816, 4_14426164, 4_14426401, 4_14437023, 4_14448922, 4_14459734, 4_14468185, 4_14499909, 4_14522693, 4_14533571, 4_14541280, 4_14592554, 4_14612556, 4_14627663, 4_14634352, 4_14638062, 4_14643765, 4_14659824, 4_14667587, 4_14683890, 4_14727892, 4_14751707, 4_14761643, 4_14830789, 4_14850266, 4_14850843, 4_14871940, 4_14902300, 4_14999727, 4_15002735, 4_15003851, 4_15008910, 4_15011312, 4_15015801, 4_15020298, 4_15031716, 4_15039376, 4_15109899, 4_15110443, 4_15136484, 4_15200030, 4_15223010, 4_15234515, 4_15244903, 4_15273400, 4_15340548, 4_15342430, 4_15373173, 4_15443316, 4_15452523, 4_15455906, 4_15575233, 4_15603736, 4_15734483, 4_15784036, 4_15786407, 4_15809092, 4_15815584, 4_15823650, 4_15825592, 4_15835219, 4_15836564, 4_15842711, 4_15887614, 4_15889401, 4_15899754, 4_15911080, 4_15914720, 4_15974450, 4_16023114, 4_16030567, 4_16034920, 4_16037910, 4_16059542, 4_16069380, 4_16070148, 4_16112875, 4_16138732, 4_16139187, 4_16159302, 4_16175803, 4_16180021, 4_16198822, 4_16242172, 4_16250308, 4_16329706, 4_16336330, 4_16376821, 4_16377584, 4_16377893, 4_16405389, 4_16412558, 4_16412783, 4_16449531, 4_16458049, 4_16551117, 4_16588253, 4_16623760, 4_16656046, 4_16682428, 4_16694550, 4_16713357, 4_16729910, 4_16777458, 4_16788006, 4_16842450, 4_16843158, 4_16859376, 4_16871183, 4_16878313, 4_16878691, 4_16880785, 4_16930057, 4_16939319, 4_16944374, 4_16951106, 4_16963623, 4_16967603, 4_16970462, 4_16999171, 4_16999729, 4_17008180, 4_17085262, 4_17089789, 4_17097290, 4_17101580, 4_17118158, 4_17140458, 4_17160412, 4_17161401, 4_17186494, 4_17191026, 4_17212356, 4_17230878, 4_17249745, 4_17279477, 4_17280710, 4_17292669, 4_17311695, 4_17326687, 4_17332063, 4_17342569, 4_17359449, 4_17442130, 4_17481637, 4_17485540, 4_17493962, 4_17517318, 4_17580752, 4_17583907, 4_17588214, 4_17625410, 4_17633799, 4_17634176, 4_17659931, 4_17664080, 4_17667004, 4_17723132, 4_17728973, 4_17740963, 4_17857255, 4_17880986, 4_17889938, 4_17917917, 4_17922476, 4_17979584, 4_17991616, 4_18063364, 4_18073382, 4_18084401, 4_18122509, 4_18170777, 4_18180896, 4_18317955, 4_18319331, 4_18325812, 4_18355734, 4_18367937, 4_18368496, 4_18373119, 4_18373510, 4_18417450, 4_18432904, 4_18437807, 4_18444088, 4_18454395, 4_18558429, 4_18563456, 4_18563868, 4_18591258, 4_18619927, 4_18640953, 4_18660906, 4_18684203, 4_18742968, 4_18759532, 4_18797920, 4_18821215, 4_18848769, 4_18873343, 4_18949886, 4_18952219, 4_18993896, 4_19010022, 4_19012532, 4_19036271, 4_19039645, 4_19049894, 4_19084942, 4_19155486, 4_19229868, 4_19230043, 4_19235231, 4_19237360, 4_19304043, 4_19332922, 4_19352459, 4_19386777, 4_19424191, 4_19446652, 4_19468797, 4_19567068, 4_19609287, 4_19610516, 4_19643025, 4_19656631, 4_19699519, 4_19708637, 4_19712873, 4_19723264, 4_19753622, 4_19838202, 4_19843280, 4_19844173, 4_19870525, 4_19874366, 4_19907329, 4_19916849, 4_19943727, 4_19968409, 4_20007858, 4_20049011, 4_20066643, 4_20112241, 4_20226417, 4_20438718, 4_20441944, 4_20449694, 4_20504228, 4_20505575, 4_20522388, 4_20750066, 4_20834851, 4_20835346, 4_20844697, 4_20858647, 4_20867682, 4_20875185, 4_20915541, 4_20933053, 4_20965755, 4_20982676, 4_20998326, 4_21010520, 4_21022599, 4_21052655, 4_21053978, 4_21062173, 4_21070338, 4_21072062, 4_21079973, 4_21104165, 4_21123754, 4_21135984, 4_21140130, 4_21152385, 4_21157229, 4_21207126, 4_21218270, 4_21219686, 4_21226978, 4_21228504, 4_21229049, 4_21331433, 4_21332413, 4_21340963, 4_21359377, 4_21409805, 4_21442502, 4_21453602, 4_21471793, 4_21472974, 4_21505676, 4_21521868, 4_21523752, 4_21554591, 4_21567539, 4_21572180, 4_21602655, 4_21618119, 4_21626112, 4_21632481, 4_21632768, 4_21653347, 4_21657653, 4_21687725, 4_21696114, 4_21701726, 4_21712606, 4_21713408, 4_21734745, 4_21736442, 4_21789663, 4_21798264, 4_21801499, 4_21811910, 4_21816719, 4_21820567, 4_21822913, 4_21826670, 4_21835255, 4_21835341, 4_21839996, 4_21848739, 4_21852340, 4_21852727, 4_21855640, 4_21869063, 4_21882875, 4_21886901, 4_21887545, 4_21895905, 4_21901741, 4_21909514, 4_21917832, 4_21918614, 4_21953407, 4_21953684, 4_21956306, 4_21979034, 4_21993907, 4_22021817, 4_22073161, 4_22085089, 4_22095721, 4_22119810, 4_22141716, 4_22146060, 4_22158763, 4_22159239, 4_22159924, 4_22178738, 4_22216455, 4_22251299, 4_22292151, 4_22309295, 4_22341008, 4_22387862, 4_22396510, 4_22429579, 4_22433842, 4_22439899, 4_22442761, 4_22451606, 4_22470539, 4_22481621, 4_22481859, 4_22484497, 4_22497811, 4_22561029, 4_22596155, 4_22640198, 4_22664535, 4_22686827, 4_22689198, 4_22692264, 4_22696469, 4_22704533, 4_22727940, 4_22808432, 4_22816790, 4_22941266, 4_22947596, 4_22964657, 4_22968368, 4_22985957, 4_23005418, 4_23010241, 4_23026876, 4_23050732, 4_23053498, 4_23076242, 4_23141615, 4_23149528, 4_23149842, 4_23154399, 4_23154489, 4_23164316, 4_23171867, 4_23206330, 4_23221102, 4_23235274, 4_23237458, 4_23260210, 4_23263663, 4_23273536, 4_23293940, 4_23321107, 4_23385874, 4_23431854, 4_23489691, 4_23548556, 4_23620155, 4_23629322, 4_23632486, 4_23645802, 4_23648033, 4_23653988, 4_23659284, 4_23676163, 4_23699497, 4_23702749, 4_23720993, 4_23745773, 4_23762347, 4_23778919, 4_23791137, 4_23809704, 4_23819518, 4_23839646, 4_23853885, 4_23858547, 4_23870842, 4_23913124, 4_23935223, 4_23953406, 4_23996964, 4_24021188, 4_24021485, 4_24063195, 4_24090885, 4_24096627, 4_24096907, 4_24102024, 4_24136814, 4_24156682, 4_24259554, 4_24268180, 4_24279915, 4_24294787, 4_24302920, 4_24303151, 4_24349633, 4_24362292, 4_24365508, 4_24367714, 4_24368202, 4_24379993, 4_24383329, 4_24383726, 4_24391176, 4_24493097, 4_24522383, 4_24543845, 4_24569439, 4_24570652, 4_24571294, 4_24576734, 4_24578279, 4_24582000, 4_24597026, 4_24646127, 4_24651698, 4_24653493, 4_24662944, 4_24678334, 4_24817982, 4_24825267, 4_24827671, 4_24833985, 4_24841776, 4_24922846, 4_24955294, 4_25004638, 4_25013022, 4_25070980, 4_25081448, 4_25109443, 4_25127865, 4_25132097, 4_25133535, 4_25152030, 4_25155773, 4_25167187, 4_25210982, 4_25229890, 4_25254883, 4_25268997, 4_25282641, 4_25318338, 4_25332293, 4_25346585, 4_25407066, 4_25420538, 4_25441179, 4_25459465, 4_25465626, 4_25483691, 4_25489034, 4_25491072, 4_25502104, 4_25502912, 4_25503757, 4_25524731, 4_25556577, 4_25604703, 4_25629360, 4_25669147, 4_25700979, 4_25705928, 4_25736070, 4_25749902, 4_25750624, 4_25759204, 4_25767368, 4_25770289, 4_25770613, 4_25816432, 4_25890554, 4_26111669, 4_26118618, 4_26132884, 4_26143704, 4_26153589, 4_26165793, 4_26194211, 4_26197965, 4_26213637, 4_26398157, 4_26433551, 4_26481553, 4_26540190, 4_26606123, 4_26749440, 4_26864144, 4_26876077, 4_27042404, 4_27042689, 4_27063781, 4_27096538, 4_27133449, 4_27201469, 4_27239321, 4_27269282, 4_27290710, 4_27344226, 4_27376009, 4_27384934, 4_27452504, 4_27465280, 4_27466966, 4_27477559, 4_27539596, 4_27565872, 4_27566676, 4_27593687, 4_27627226, 4_27633919, 4_27636866, 4_27642741, 4_27645133, 4_27667174, 4_27673962, 4_27691755, 4_27712709, 4_27723142, 4_27782135, 4_27830628, 4_27833104, 4_27846937, 4_27867283, 4_27907284, 4_27913199, 4_27951139, 4_28002466, 4_28053964, 4_28057717, 4_28091922, 4_28139221, 4_28156741, 4_28207108, 4_28212229, 4_28290789, 4_28301504, 4_28302614, 4_28315757, 4_28316709, 4_28345475, 4_28346485, 4_28357279, 4_28382196, 4_28453611, 4_28466267, 4_28471869, 4_28495296, 4_28647601, 4_28676981, 4_28700303, 4_28715424, 4_28716764, 4_28737322, 4_28792563, 4_28801488, 4_28801706, 4_28870389, 4_28875899, 4_28949954, 4_28968315, 4_29061732, 4_29125801, 4_29130317, 4_29130898, 4_29187169, 4_29235828, 4_29267676, 4_29277139, 4_29277559, 4_29321165, 4_29347775, 4_29430011, 4_29438659, 4_29493033, 4_29534978, 4_29590184, 4_29602916, 4_29671526, 4_29696930, 4_29722345, 4_29729841, 4_29735283, 4_29748422, 4_29750150, 4_29751555, 4_29766840, 4_29779853, 4_29780585, 4_29789475, 4_29794192, 4_29810463, 4_29825203, 4_29831014, 4_29850727, 4_29861085, 4_29884186, 4_29886033, 4_29904130, 4_29911624, 4_29933643, 4_29942645, 4_30046972, 4_30052883, 4_30069970, 4_30083342, 4_30108024, 4_30139999, 4_30157217, 4_30161165, 4_30163232, 4_30258469, 4_30292151, 4_30300894, 4_30304073, 4_30316054, 4_30317571, 4_30326575, 4_30336585, 4_30343622, 4_30415649, 4_30436816, 4_30470002, 4_30472493, 4_30487369, 4_30552266, 4_30555117, 4_30558422, 4_30561676, 4_30567749, 4_30571455, 4_30601417, 4_30602091, 4_30605334, 4_30606998, 4_30627293, 4_30632078, 4_30636522, 4_30663009, 4_30667741, 4_30671409, 4_30674820, 4_30677840, 4_30715243, 4_30723079, 4_30731192, 4_30755085, 4_30762424, 4_30769912, 4_30794127, 4_30806096, 4_30829525, 4_30829587, 4_30831637, 4_30843357, 4_30863102, 4_30903783, 5_13220, 5_16880, 5_31086, 5_43224, 5_91481, 5_103915, 5_105190, 5_117572, 5_121871, 5_145128, 5_175750, 5_177429, 5_182946, 5_209822, 5_213120, 5_214594, 5_217607, 5_228888, 5_229437, 5_247685, 5_253697, 5_255346, 5_265662, 5_269833, 5_270300, 5_277990, 5_289136, 5_294710, 5_328098, 5_330004, 5_332378, 5_337005, 5_339128, 5_340352, 5_342988, 5_351477, 5_353309, 5_359338, 5_360157, 5_442341, 5_445895, 5_452325, 5_459892, 5_464106, 5_467702, 5_488175, 5_516302, 5_516590, 5_531215, 5_533294, 5_534575, 5_536283, 5_543217, 5_548194, 5_591439, 5_596948, 5_601812, 5_622510, 5_626035, 5_635347, 5_639858, 5_667263, 5_667527, 5_674125, 5_682258, 5_686320, 5_694463, 5_702636, 5_739223, 5_763143, 5_778178, 5_778299, 5_784616, 5_793813, 5_798021, 5_810124, 5_810432, 5_812992, 5_838742, 5_839500, 5_861749, 5_869145, 5_892288, 5_923238, 5_931503, 5_939757, 5_940019, 5_940421, 5_963462, 5_964440, 5_965042, 5_1060437, 5_1102090, 5_1112437, 5_1122425, 5_1129089, 5_1136739, 5_1172649, 5_1177894, 5_1193611, 5_1197682, 5_1230134, 5_1230875, 5_1243765, 5_1244500, 5_1276551, 5_1290565, 5_1346704, 5_1348689, 5_1360880, 5_1364886, 5_1372635, 5_1376533, 5_1388874, 5_1394063, 5_1409703, 5_1409958, 5_1410577, 5_1410648, 5_1413084, 5_1422857, 5_1424762, 5_1430845, 5_1448271, 5_1459102, 5_1462987, 5_1491918, 5_1510354, 5_1514484, 5_1541557, 5_1548106, 5_1550648, 5_1551067, 5_1554723, 5_1565561, 5_1567497, 5_1598025, 5_1613781, 5_1652113, 5_1652706, 5_1685067, 5_1687039, 5_1687353, 5_1713915, 5_1718812, 5_1719816, 5_1720189, 5_1721034, 5_1721448, 5_1724092, 5_1744909, 5_1753359, 5_1772772, 5_1772983, 5_1773458, 5_1796757, 5_1797389, 5_1805727, 5_1806000, 5_1806375, 5_1807997, 5_1809650, 5_1817058, 5_1845206, 5_1847342, 5_1847503, 5_1860340, 5_1878974, 5_1884093, 5_1908612, 5_1948342, 5_1950098, 5_1950544, 5_1996342, 5_2041148, 5_2079907, 5_2109219, 5_2111650, 5_2136056, 5_2157201, 5_2161655, 5_2173017, 5_2186430, 5_2190394, 5_2192625, 5_2209190, 5_2235078, 5_2240108, 5_2258896, 5_2268828, 5_2275236, 5_2285982, 5_2286298, 5_2299219, 5_2302037, 5_2319288, 5_2350877, 5_2367380, 5_2395409, 5_2411826, 5_2418940, 5_2431841, 5_2434455, 5_2454592, 5_2463591, 5_2485497, 5_2498803, 5_2578869, 5_2601468, 5_2616212, 5_2631577, 5_2646613, 5_2657687, 5_2670348, 5_2685323, 5_2685927, 5_2692001, 5_2692834, 5_2695793, 5_2701476, 5_2723539, 5_2724037, 5_2731217, 5_2743094, 5_2805071, 5_2822296, 5_2835088, 5_2878664, 5_2885139, 5_2919360, 5_2924073, 5_2930003, 5_2953218, 5_2959265, 5_2960134, 5_2962242, 5_2976202, 5_3020329, 5_3022830, 5_3038542, 5_3046211, 5_3049396, 5_3049641, 5_3050347, 5_3070037, 5_3119710, 5_3121679, 5_3128890, 5_3143303, 5_3155704, 5_3195289, 5_3197462, 5_3268231, 5_3280403, 5_3286096, 5_3309698, 5_3313141, 5_3324494, 5_3325766, 5_3350912, 5_3366861, 5_3372119, 5_3404418, 5_3410843, 5_3429197, 5_3430820, 5_3435173, 5_3450475, 5_3484417, 5_3485515, 5_3500960, 5_3517953, 5_3558131, 5_3577217, 5_3604491, 5_3605963, 5_3656940, 5_3662500, 5_3671195, 5_3688481, 5_3717178, 5_3718545, 5_3742680, 5_3748001, 5_3783028, 5_3809010, 5_3809516, 5_3851144, 5_3900051, 5_3902323, 5_3922833, 5_3931006, 5_3965750, 5_3987185, 5_4006730, 5_4048410, 5_4059068, 5_4079406, 5_4176560, 5_4226042, 5_4230027, 5_4255140, 5_4270240, 5_4273117, 5_4290051, 5_4306615, 5_4344773, 5_4367258, 5_4387819, 5_4402455, 5_4441463, 5_4444231, 5_4457509, 5_4465251, 5_4469697, 5_4518275, 5_4586784, 5_4616479, 5_4648237, 5_4649483, 5_4650534, 5_4673549, 5_4694023, 5_4731734, 5_4738405, 5_4780735, 5_4815060, 5_4818218, 5_4846663, 5_4851202, 5_4858709, 5_4872135, 5_4907482, 5_4956183, 5_4983546, 5_4983758, 5_4985574, 5_4988151, 5_4989112, 5_5002605, 5_5009432, 5_5039866, 5_5061891, 5_5081546, 5_5103699, 5_5104053, 5_5124047, 5_5149937, 5_5153152, 5_5156538, 5_5171567, 5_5183545, 5_5215429, 5_5226270, 5_5231047, 5_5243907, 5_5247615, 5_5258318, 5_5263432, 5_5266774, 5_5284543, 5_5285886, 5_5291060, 5_5296321, 5_5315542, 5_5324678, 5_5326289, 5_5331953, 5_5336544, 5_5347184, 5_5374151, 5_5387645, 5_5400864, 5_5441617, 5_5444379, 5_5464658, 5_5485566, 5_5492428, 5_5499365, 5_5512975, 5_5514142, 5_5515441, 5_5515735, 5_5519077, 5_5519475, 5_5558751, 5_5563574, 5_5572815, 5_5583011, 5_5585341, 5_5707360, 5_5749038, 5_5760730, 5_5765868, 5_5787009, 5_5800454, 5_5820885, 5_5822824, 5_5826102, 5_5906955, 5_5970496, 5_5975851, 5_5995797, 5_6006209, 5_6015774, 5_6034735, 5_6065257, 5_6071990, 5_6072790, 5_6118307, 5_6163528, 5_6176717, 5_6179997, 5_6248495, 5_6297751, 5_6305768, 5_6365996, 5_6366444, 5_6437833, 5_6452662, 5_6519832, 5_6520323, 5_6541165, 5_6577012, 5_6589377, 5_6603918, 5_6609746, 5_6614664, 5_6618288, 5_6639795, 5_6655128, 5_6678357, 5_6701252, 5_6719223, 5_6725397, 5_6733592, 5_6886727, 5_7040619, 5_7066787, 5_7103095, 5_7166563, 5_7203407, 5_7213687, 5_7329515, 5_7353745, 5_7353842, 5_7383053, 5_7445118, 5_7461151, 5_7463873, 5_7557666, 5_7618278, 5_7620924, 5_7669888, 5_7674020, 5_7683745, 5_7685754, 5_7710876, 5_7729112, 5_7775278, 5_7791790, 5_7858701, 5_7879032, 5_7900966, 5_7926537, 5_7937305, 5_7937811, 5_7968060, 5_7970045, 5_7970628, 5_7981064, 5_7993124, 5_8013690, 5_8020239, 5_8020628, 5_8021285, 5_8021412, 5_8093237, 5_8095690, 5_8105093, 5_8118321, 5_8191549, 5_8199239, 5_8209453, 5_8217679, 5_8227720, 5_8238968, 5_8243623, 5_8250412, 5_8257383, 5_8290893, 5_8334640, 5_8337222, 5_8349274, 5_8356250, 5_8361206, 5_8361721, 5_8392835, 5_8393823, 5_8396585, 5_8458769, 5_8497926, 5_8499283, 5_8614814, 5_8640654, 5_8648789, 5_8657633, 5_8686072, 5_8708649, 5_8708872, 5_8709640, 5_8735050, 5_8747935, 5_8756853, 5_8823005, 5_8825436, 5_8846832, 5_8847308, 5_8884412, 5_8884440, 5_8908055, 5_8914305, 5_8917108, 5_8990467, 5_8993433, 5_9011079, 5_9020509, 5_9117097, 5_9152294, 5_9159182, 5_9162064, 5_9171035, 5_9264207, 5_9283623, 5_9296468, 5_9486806, 5_9503440, 5_9560945, 5_9575899, 5_9604663, 5_9619442, 5_9622191, 5_9663047, 5_9663114, 5_9666910, 5_9762614, 5_9886565, 5_9931201, 5_9947333, 5_9987731, 5_10021816, 5_10059240, 5_10069079, 5_10078582, 5_10079185, 5_10117044, 5_10127877, 5_10145513, 5_10185107, 5_10201580, 5_10230421, 5_10232762, 5_10233022, 5_10247405, 5_10289623, 5_10343491, 5_10426204, 5_10439918, 5_10441167, 5_10441426, 5_10471057, 5_10482105, 5_10491977, 5_10498287, 5_10501852, 5_10556694, 5_10609998, 5_10615765, 5_10705136, 5_10717210, 5_10717709, 5_10737971, 5_10758947, 5_10831600, 5_10841218, 5_10841435, 5_10851946, 5_10858111, 5_10858671, 5_10891104, 5_10904666, 5_10937943, 5_10958086, 5_10997891, 5_11026063, 5_11027955, 5_11064169, 5_11081402, 5_11090748, 5_11117983, 5_11141493, 5_11206088, 5_11206405, 5_11210628, 5_11252601, 5_11333670, 5_11359805, 5_11387182, 5_11470195, 5_11530078, 5_11541637, 5_11541810, 5_11561985, 5_11566941, 5_11592487, 5_11675012, 5_11686024, 5_11832622, 5_11850001, 5_11874269, 5_11899272, 5_11905194, 5_11929084, 5_11934690, 5_12251017, 5_12387888, 5_12395054, 5_12426725, 5_12472377, 5_12499549, 5_12511667, 5_12587612, 5_12587936, 5_12601920, 5_12719374, 5_12770093, 5_12837963, 5_12843804, 5_12894083, 5_12932293, 5_13064054, 5_13117885, 5_13240598, 5_13270682, 5_13284578, 5_13309645, 5_13341594, 5_13421678, 5_13454186, 5_13461400, 5_13462676, 5_13481800, 5_13513686, 5_13560317, 5_13562819, 5_13592585, 5_13648049, 5_13657274, 5_13689973, 5_13725724, 5_13734477, 5_13772621, 5_13845823, 5_13879948, 5_13938344, 5_13962369, 5_13978125, 5_13988376, 5_13998219, 5_14087032, 5_14150719, 5_14159236, 5_14221843, 5_14288427, 5_14300334, 5_14524113, 5_14602247, 5_14652021, 5_14652422, 5_14756410, 5_14813175, 5_14864967, 5_14943799, 5_14977446, 5_15058070, 5_15079022, 5_15089222, 5_15317671, 5_15370743, 5_15429811, 5_15430399, 5_15495244, 5_15502370, 5_15505734, 5_15624381, 5_15649453, 5_15674784, 5_15778138, 5_15781954, 5_15801681, 5_16127290, 5_16231093, 5_16345292, 5_16348487, 5_16353953, 5_16408845, 5_16410068, 5_16430194, 5_16488402, 5_16495338, 5_16736443, 5_16752474, 5_16798856, 5_16799190, 5_16822583, 5_16839530, 5_16847513, 5_16849553, 5_16851053, 5_16904283, 5_16936620, 5_17032421, 5_17057969, 5_17060426, 5_17086044, 5_17089139, 5_17093939, 5_17122441, 5_17126711, 5_17255261, 5_17335424, 5_17392410, 5_17493360, 5_17590138, 5_17593329, 5_17594677, 5_17612540, 5_17618531, 5_17627029, 5_17636013, 5_17644373, 5_17693173, 5_17714680, 5_17733372, 5_17740927, 5_17769645, 5_17777170, 5_17783743, 5_17784055, 5_17915602, 5_18120076, 5_18143560, 5_18183951, 5_18256143, 5_18271389, 5_18335478, 5_18342528, 5_18359242, 5_18360427, 5_18397936, 5_18414213, 5_18416751, 5_18433009, 5_18454992, 5_18468373, 5_18487744, 5_18547324, 5_18565006, 5_18569460, 5_18657207, 5_18670364, 5_18679037, 5_18720550, 5_18753779, 5_18760944, 5_18765964, 5_18795043, 5_18827576, 5_18874758, 5_18879954, 5_18929973, 5_18931990, 5_18939304, 5_18959087, 5_18960417, 5_18967618, 5_19017716, 5_19035521, 5_19057527, 5_19214610, 5_19220099, 5_19221900, 5_19224824, 5_19273611, 5_19297368, 5_19308936, 5_19477731, 5_19482032, 5_19484325, 5_19505713, 5_19535911, 5_19540135, 5_19569903, 5_19618113, 5_19621703, 5_19643417, 5_19653136, 5_19653853, 5_19654161, 5_19661174, 5_19663857, 5_19679724, 5_19686905, 5_19701070, 5_19730607, 5_19752943, 5_19754256, 5_19795812, 5_19893243, 5_19902541, 5_19906010, 5_19913562, 5_19915087, 5_19962656, 5_19966361, 5_19979128, 5_19987661, 5_20014054, 5_20094687, 5_20095844, 5_20105419, 5_20132190, 5_20149964, 5_20166052, 5_20211882, 5_20225011, 5_20293870, 5_20306231, 5_20367771, 5_20439871, 5_20455319, 5_20465928, 5_20498724, 5_20515636, 5_20560216, 5_20563129, 5_20574766, 5_20598132, 5_20600965, 5_20608028, 5_20642443, 5_20676432, 5_20703830, 5_20704134, 5_20714056, 5_20729653, 5_20730939, 5_20736428, 5_20744635, 5_20755706, 5_20791966, 5_20794806, 5_20803400, 5_20805987, 5_20858425, 5_20868646, 5_20882867, 5_20892792, 5_20935251, 5_20940056, 5_20956409, 5_21004622, 5_21076613, 5_21201885, 5_21231602, 5_21266958, 5_21279161, 5_21305461, 5_21331111, 5_21332445, 5_21358643, 5_21393349, 5_21395004, 5_21430951, 5_21433649, 5_21451664, 5_21504500, 5_21505460, 5_21506873, 5_21514413, 5_21540653, 5_21546397, 5_21566624, 5_21566930, 5_21573688, 5_21584009, 5_21585980, 5_21587699, 5_21622254, 5_21654389, 5_21685969, 5_21692288, 5_21715285, 5_21721829, 5_21727442, 5_21765509, 5_21794322, 5_21857007, 5_21879187, 5_21917920, 5_21970905, 5_21994170, 5_22019238, 5_22051699, 5_22052106, 5_22065120, 5_22085515, 5_22140841, 5_22154013, 5_22161719, 5_22171029, 5_22176295, 5_22201007, 5_22221736, 5_22222036, 5_22222841, 5_22227040, 5_22244311, 5_22245647, 5_22256852, 5_22257429, 5_22278996, 5_22279083, 5_22332798, 5_22334599, 5_22366230, 5_22385047, 5_22403304, 5_22413082, 5_22421798, 5_22448102, 5_22493035, 5_22500300, 5_22559687, 5_22568181, 5_22578123, 5_22599056, 5_22649472, 5_22653950, 5_22680223, 5_22686100, 5_22710582, 5_22737312, 5_22741936, 5_22751341, 5_22763568, 5_22777410, 5_22813268, 5_22839627, 5_22861117, 5_22863504, 5_22900976, 5_22928821, 5_22931145, 5_22933568, 5_22970626, 5_22977256, 5_22986447, 5_22990896, 5_23005846, 5_23056180, 5_23062015, 5_23076888, 5_23096375, 5_23101099, 5_23141729, 5_23143565, 5_23164141, 5_23173054, 5_23194102, 5_23198288, 5_23251865, 5_23254301, 5_23260242, 5_23345582, 5_23371615, 5_23379765, 5_23386566, 5_23405964, 5_23412654, 5_23414395, 5_23427464, 5_23457827, 5_23512266, 5_23572225, 5_23581731, 5_23599744, 5_23615470, 5_23635397, 5_23677464, 5_23694165, 5_23704000, 5_23715999, 5_23719172, 5_23761023, 5_23848879, 5_23851194, 5_23879610, 5_23919850, 5_23932647, 5_23939773, 5_23945659, 5_23971332, 5_23990363, 5_23996421, 5_24003755, 5_24020150, 5_24047542, 5_24054024, 5_24057388, 5_24098870, 5_24141037, 5_24142927, 5_24146730, 5_24162556, 5_24267317, 5_24326021, 5_24372744, 5_24379047, 5_24386169, 5_24402264, 5_24408090, 5_24450004, 5_24455967, 5_24460811, 5_24466465, 5_24521332, 5_24539388, 5_24543370, 5_24596714, 5_24598687, 5_24609237, 5_24622981, 5_24628232, 5_24634990, 5_24646805, 5_24656323, 5_24670836, 5_24686736, 5_24686868, 5_24691232, 5_24720344, 5_24729692, 5_24732788, 5_24740851, 5_24782881, 5_24816181, 5_24854676, 5_24860012, 5_24860656, 5_24864843, 5_24870206, 5_24900998, 5_24931268, 5_24943738, 5_25048163, 5_25095357, 5_25095893, 5_25105794, 5_25170587, 5_25210066, 5_25232023, 5_25234076, 5_25240702, 5_25294808, 5_25331118, 5_25341992, 5_25375866, 5_25417716, 5_25510694, 5_25529955, 5_25557240, 5_25601806, 5_25626179, 5_25651905, 5_25698818, 5_25715055, 5_25783850, 5_25820419, 5_25824566, 5_25839961, 5_25874758, 5_26008982, 5_26023293, 5_26064399, 5_26325959, 5_26353977, 5_26371954, 5_26389353, 5_26394835, 5_26533059, 5_26598831, 5_26612271, 5_26619061, 5_26630667, 5_26666136, 5_26671754, 5_26686028, 5_26768483, 5_26821132, 5_26823755, 5_26863584, 5_26886190, 5_26919682, 5_26950729, 5_26959456, 5_26960082, 5_26961635, 5_26981253, 5_27003764, 5_27014011, 5_27019018, 5_27022583, 5_27089476, 5_27097530, 5_27130031, 5_27134094, 5_27236905, 5_27321847, 5_27333756, 5_27379374, 5_27379835, 5_27428146, 5_27528646, 5_27539772, 5_27548698, 5_27548774, 5_27549391, 5_27596406, 5_27615623, 5_27615844, 5_27632118, 5_27647773, 5_27655226, 5_27658944, 5_27680163, 5_27732652, 5_27732814, 5_27734837, 5_27766256, 5_27767646, 5_27927873, 5_27963029, 5_27976140, 5_27981436, 5_27982118, 5_27983497, 5_28021829, 5_28051569, 5_28126100, 5_28139509, 5_28152166, 5_28177798, 5_28189496, 5_28234091, 5_28304846, 5_28326902, 5_28430661, 5_28450314, 5_28459733, 5_28467255, 5_28518768, 5_28547976, 5_28580241, 5_28604560, 5_28614308, 5_28634829, 5_28664864, 5_28672730, 5_28696482, 5_28738206, 5_28788435, 5_28817829, 5_28840007, 5_28841878, 5_28846596, 5_28865179, 5_28866653, 5_28869213, 5_28880861, 5_28889502, 5_28917446, 5_28923652, 5_28927644, 5_29001418, 5_29007207, 5_29020632, 5_29043377, 5_29075951, 5_29133534, 5_29193167, 5_29205653, 5_29212154, 5_29214047, 5_29226029, 5_29308278, 5_29310230, 5_29310571, 5_29324845, 5_29350035, 5_29357076, 6_680, 6_7022, 6_27558, 6_54001, 6_54556, 6_74421, 6_74755, 6_92567, 6_95840, 6_101958, 6_117977, 6_118699, 6_134134, 6_134421, 6_145780, 6_151115, 6_163846, 6_165445, 6_197574, 6_199211, 6_208889, 6_209249, 6_229634, 6_230280, 6_233423, 6_241515, 6_253864, 6_256774, 6_282130, 6_286125, 6_296897, 6_313511, 6_323743, 6_326469, 6_329948, 6_330503, 6_344622, 6_362257, 6_364270, 6_374093, 6_409531, 6_427067, 6_432257, 6_432532, 6_451181, 6_454757, 6_500278, 6_502321, 6_524619, 6_527572, 6_548308, 6_558728, 6_570854, 6_581370, 6_611224, 6_611332, 6_622787, 6_627887, 6_631843, 6_632155, 6_635093, 6_653854, 6_669013, 6_689934, 6_721381, 6_746874, 6_765011, 6_772919, 6_775040, 6_795653, 6_803404, 6_819472, 6_831737, 6_839563, 6_848110, 6_853066, 6_907576, 6_917767, 6_983696, 6_998112, 6_998616, 6_1001168, 6_1004432, 6_1010491, 6_1033370, 6_1056505, 6_1056804, 6_1066731, 6_1069054, 6_1097624, 6_1124802, 6_1152042, 6_1229781, 6_1232645, 6_1235786, 6_1305967, 6_1307484, 6_1325638, 6_1375646, 6_1378823, 6_1383063, 6_1396122, 6_1399318, 6_1413512, 6_1430606, 6_1470047, 6_1471271, 6_1481152, 6_1505421, 6_1521831, 6_1527662, 6_1530610, 6_1536749, 6_1554539, 6_1575481, 6_1600734, 6_1604114, 6_1624534, 6_1638604, 6_1682786, 6_1686317, 6_1693393, 6_1693463, 6_1708599, 6_1713073, 6_1721837, 6_1726018, 6_1757484, 6_1759294, 6_1766203, 6_1781352, 6_1794797, 6_1805104, 6_1828132, 6_1915958, 6_1918256, 6_1972322, 6_2033784, 6_2050791, 6_2054215, 6_2067969, 6_2073970, 6_2091643, 6_2118203, 6_2130567, 6_2131132, 6_2145757, 6_2177421, 6_2194096, 6_2212959, 6_2219954, 6_2228366, 6_2228482, 6_2244837, 6_2249458, 6_2253956, 6_2257267, 6_2263677, 6_2278407, 6_2300178, 6_2309710, 6_2330410, 6_2353136, 6_2391844, 6_2453038, 6_2453187, 6_2507839, 6_2507911, 6_2519328, 6_2545960, 6_2562563, 6_2571826, 6_2610571, 6_2634729, 6_2635690, 6_2661425, 6_2665253, 6_2682200, 6_2684307, 6_2684520, 6_2708935, 6_2742425, 6_2779943, 6_2781029, 6_2788314, 6_2791674, 6_2794532, 6_2797296, 6_2821231, 6_2832596, 6_2842668, 6_2870233, 6_2908412, 6_2941091, 6_2957146, 6_2961224, 6_2972296, 6_3003010, 6_3006720, 6_3021032, 6_3022962, 6_3071444, 6_3105189, 6_3116721, 6_3120250, 6_3120618, 6_3161365, 6_3168401, 6_3177274, 6_3205375, 6_3208284, 6_3215903, 6_3228252, 6_3256567, 6_3263398, 6_3309707, 6_3339599, 6_3347213, 6_3358076, 6_3371965, 6_3385189, 6_3388523, 6_3464916, 6_3484634, 6_3491191, 6_3503399, 6_3535021, 6_3553977, 6_3560559, 6_3597150, 6_3598280, 6_3601459, 6_3601710, 6_3607443, 6_3607697, 6_3612153, 6_3625087, 6_3629143, 6_3633967, 6_3644310, 6_3645078, 6_3647480, 6_3677568, 6_3683394, 6_3691217, 6_3731573, 6_3750216, 6_3782329, 6_3792087, 6_3799596, 6_3804254, 6_3807811, 6_3818601, 6_3864676, 6_3880866, 6_3886394, 6_3899749, 6_3913905, 6_3915653, 6_3928765, 6_3937247, 6_3938124, 6_3941832, 6_3944184, 6_3944614, 6_3990019, 6_4005817, 6_4006096, 6_4013956, 6_4032669, 6_4035056, 6_4036134, 6_4044406, 6_4057583, 6_4057692, 6_4059468, 6_4091737, 6_4102317, 6_4104849, 6_4115503, 6_4124680, 6_4154500, 6_4160128, 6_4165162, 6_4180222, 6_4182392, 6_4193430, 6_4217404, 6_4257471, 6_4265596, 6_4271848, 6_4277760, 6_4283113, 6_4287842, 6_4304515, 6_4323307, 6_4326158, 6_4355337, 6_4416846, 6_4429771, 6_4431021, 6_4451405, 6_4470078, 6_4483965, 6_4505062, 6_4514662, 6_4520066, 6_4521386, 6_4577111, 6_4580328, 6_4583097, 6_4617672, 6_4654435, 6_4659173, 6_4664714, 6_4665023, 6_4680505, 6_4680761, 6_4684858, 6_4693552, 6_4695799, 6_4696683, 6_4708908, 6_4721916, 6_4740193, 6_4780006, 6_4804636, 6_4847266, 6_4859240, 6_4860488, 6_4864284, 6_4879909, 6_4898004, 6_4904403, 6_4936036, 6_4953601, 6_4978233, 6_4985769, 6_4996017, 6_5005570, 6_5027179, 6_5045148, 6_5070708, 6_5072438, 6_5075781, 6_5079751, 6_5081214, 6_5086499, 6_5090692, 6_5114006, 6_5115434, 6_5167153, 6_5171826, 6_5183646, 6_5183991, 6_5186523, 6_5190720, 6_5195134, 6_5248527, 6_5273501, 6_5289742, 6_5332132, 6_5407001, 6_5407066, 6_5407359, 6_5408797, 6_5417170, 6_5457858, 6_5466286, 6_5469695, 6_5488198, 6_5499804, 6_5504020, 6_5506306, 6_5506784, 6_5507137, 6_5555781, 6_5562368, 6_5562521, 6_5566737, 6_5589156, 6_5612550, 6_5613848, 6_5614135, 6_5622286, 6_5623097, 6_5641737, 6_5664538, 6_5728985, 6_5730261, 6_5773524, 6_5815663, 6_5816875, 6_5842747, 6_5905119, 6_5907768, 6_5925665, 6_5935458, 6_5941533, 6_5964467, 6_5999583, 6_6000476, 6_6027617, 6_6057848, 6_6061198, 6_6079450, 6_6092434, 6_6162383, 6_6172792, 6_6207940, 6_6217141, 6_6232801, 6_6242159, 6_6244309, 6_6245190, 6_6251513, 6_6254269, 6_6311860, 6_6316470, 6_6348564, 6_6392732, 6_6415260, 6_6419739, 6_6428677, 6_6435624, 6_6437215, 6_6437825, 6_6450841, 6_6469694, 6_6494073, 6_6498975, 6_6499202, 6_6507838, 6_6549231, 6_6556479, 6_6558025, 6_6558305, 6_6562001, 6_6570628, 6_6592573, 6_6600123, 6_6615262, 6_6620832, 6_6622553, 6_6670605, 6_6702162, 6_6728473, 6_6761879, 6_6772818, 6_6781503, 6_6809976, 6_6821234, 6_6821684, 6_6828812, 6_6857901, 6_6869652, 6_6889876, 6_6893730, 6_6899440, 6_6899525, 6_6910215, 6_6912422, 6_6912746, 6_6918027, 6_6946591, 6_6955559, 6_6959345, 6_6964825, 6_6984650, 6_6993557, 6_6993911, 6_7055757, 6_7057844, 6_7062507, 6_7065707, 6_7069588, 6_7071183, 6_7095647, 6_7095957, 6_7190660, 6_7213750, 6_7223699, 6_7233576, 6_7269042, 6_7270050, 6_7278728, 6_7291695, 6_7307954, 6_7339054, 6_7364422, 6_7405199, 6_7471956, 6_7488924, 6_7548032, 6_7559958, 6_7582209, 6_7667492, 6_7697361, 6_7707309, 6_7710002, 6_7749009, 6_7749283, 6_7774008, 6_7779553, 6_7802312, 6_7826443, 6_7864473, 6_7917129, 6_7923626, 6_7929630, 6_7934382, 6_7956927, 6_7970542, 6_7970811, 6_7991766, 6_8029082, 6_8068025, 6_8068581, 6_8068866, 6_8076858, 6_8089532, 6_8120502, 6_8126638, 6_8150269, 6_8150571, 6_8195442, 6_8218600, 6_8226633, 6_8232280, 6_8246205, 6_8314112, 6_8387518, 6_8416629, 6_8418717, 6_8436927, 6_8505998, 6_8511115, 6_8512591, 6_8515345, 6_8567039, 6_8587765, 6_8642638, 6_8676121, 6_8691984, 6_8693315, 6_8720068, 6_8782953, 6_8818102, 6_8852287, 6_8862020, 6_8862866, 6_8862939, 6_8873926, 6_8877070, 6_8878353, 6_8897514, 6_8900528, 6_8923643, 6_8925945, 6_8929873, 6_8937590, 6_8950981, 6_8969636, 6_9015806, 6_9063312, 6_9067405, 6_9073810, 6_9082077, 6_9084246, 6_9093110, 6_9107249, 6_9111378, 6_9119220, 6_9192859, 6_9206816, 6_9225527, 6_9226421, 6_9237501, 6_9263612, 6_9264107, 6_9283016, 6_9350366, 6_9363739, 6_9376007, 6_9391324, 6_9394396, 6_9406319, 6_9468504, 6_9499502, 6_9511885, 6_9514712, 6_9559469, 6_9596417, 6_9610630, 6_9652272, 6_9666732, 6_9693214, 6_9707471, 6_9798869, 6_9855951, 6_9880245, 6_9990946, 6_10002120, 6_10037238, 6_10048538, 6_10048801, 6_10082295, 6_10114948, 6_10118809, 6_10120957, 6_10130431, 6_10137711, 6_10142375, 6_10162698, 6_10223150, 6_10225768, 6_10225927, 6_10243496, 6_10255402, 6_10309083, 6_10309363, 6_10313668, 6_10332572, 6_10337842, 6_10403032, 6_10403599, 6_10405093, 6_10413762, 6_10433146, 6_10440657, 6_10471481, 6_10581110, 6_10616383, 6_10627765, 6_10633684, 6_10633936, 6_10692654, 6_10723297, 6_10761142, 6_10785582, 6_10786007, 6_10924295, 6_10941812, 6_10947568, 6_10965012, 6_11024393, 6_11047593, 6_11086324, 6_11129152, 6_11133433, 6_11149791, 6_11169718, 6_11182463, 6_11207821, 6_11216723, 6_11220626, 6_11236513, 6_11330680, 6_11365328, 6_11366964, 6_11399715, 6_11436080, 6_11494955, 6_11587359, 6_11673290, 6_11684884, 6_11719650, 6_11819346, 6_11846219, 6_11891202, 6_11913602, 6_11913944, 6_11917028, 6_11942910, 6_12036174, 6_12038039, 6_12046112, 6_12120436, 6_12120720, 6_12139473, 6_12361368, 6_12362190, 6_12367195, 6_12412235, 6_12420703, 6_12465061, 6_12474143, 6_12478388, 6_12509495, 6_12512166, 6_12514180, 6_12516297, 6_12559657, 6_12599691, 6_12625267, 6_12628768, 6_12635116, 6_12658704, 6_12666826, 6_12678857, 6_12744948, 6_12751636, 6_12780257, 6_12823282, 6_12824993, 6_12831761, 6_12845169, 6_12850190, 6_12853690, 6_12861592, 6_12872723, 6_12872813, 6_12873591, 6_12878723, 6_12897351, 6_12906033, 6_12910916, 6_12921032, 6_12931862, 6_12937728, 6_12956136, 6_12956189, 6_12981063, 6_12985664, 6_13011415, 6_13014066, 6_13014962, 6_13024684, 6_13046286, 6_13054233, 6_13058681, 6_13089831, 6_13102640, 6_13140285, 6_13161991, 6_13219715, 6_13255063, 6_13308323, 6_13342169, 6_13387886, 6_13413378, 6_13415529, 6_13419005, 6_13423851, 6_13482039, 6_13531016, 6_13554164, 6_13641573, 6_13662789, 6_13665055, 6_13668471, 6_13669785, 6_13701928, 6_13709080, 6_13734385, 6_13809416, 6_13837547, 6_13845112, 6_13845554, 6_13854717, 6_13862546, 6_13878460, 6_13887389, 6_13888274, 6_13888368, 6_13962369, 6_13978488, 6_14061443, 6_14114186, 6_14176044, 6_14199267, 6_14214465, 6_14218796, 6_14262249, 6_14318134, 6_14382661, 6_14395522, 6_14449329, 6_14451112, 6_14504996, 6_14507073, 6_14519643, 6_14523925, 6_14528312, 6_14534029, 6_14549487, 6_14567667, 6_14589466, 6_14599874, 6_14607080, 6_14620833, 6_14653199, 6_14665382, 6_14727642, 6_14745057, 6_14746339, 6_14772695, 6_14777914, 6_14784254, 6_14808284, 6_14843223, 6_14870606, 6_14875913, 6_14937712, 6_14947751, 6_14977126, 6_15009939, 6_15030790, 6_15038595, 6_15041161, 6_15052017, 6_15090699, 6_15106215, 6_15122989, 6_15308745, 6_15308840, 6_15423545, 6_15429187, 6_15465000, 6_15501256, 6_15507429, 6_15509585, 6_15532245, 6_15546015, 6_15547775, 6_15565303, 6_15622530, 6_15668662, 6_15713858, 6_15721459, 6_15876929, 6_15886154, 6_15907440, 6_15923778, 6_15924044, 6_15938768, 6_15973696, 6_15988011, 6_16005886, 6_16006408, 6_16035736, 6_16115436, 6_16123282, 6_16124289, 6_16128595, 6_16168906, 6_16183622, 6_16202980, 6_16254353, 6_16283359, 6_16294464, 6_16301506, 6_16322908, 6_16333298, 6_16340412, 6_16374995, 6_16375850, 6_16391145, 6_16433350, 6_16443800, 6_16495539, 6_16510747, 6_16539115, 6_16595276, 6_16595678, 6_16595979, 6_16618353, 6_16623065, 6_16627448, 6_16649383, 6_16665416, 6_16666698, 6_16703987, 6_16706006, 6_16718398, 6_16737625, 6_16771896, 6_16788878, 6_16802134, 6_16804384, 6_16852394, 6_16876500, 6_16878809, 6_16880100, 6_16880238, 6_16939935, 6_16948925, 6_16967921, 6_16982287, 6_16984461, 6_17005026, 6_17014750, 6_17117200, 6_17124521, 6_17144577, 6_17145089, 6_17148278, 6_17162159, 6_17177507, 6_17244197, 6_17326702, 6_17332846, 6_17345380, 6_17437861, 6_17441449, 6_17450844, 6_17528683, 6_17541943, 6_17571038, 6_17577156, 6_17586258, 6_17587632, 6_17599847, 6_17609494, 6_17618286, 6_17659547, 6_17668318, 6_17687512, 6_17696276, 6_17736052, 6_17739671, 6_17786253, 6_17814352, 6_17875579, 6_17885484, 6_17898053, 6_17898890, 6_17918078, 6_17922583, 6_18043595, 6_18054358, 6_18077492, 6_18135010, 6_18177977, 6_18226508, 6_18235419, 6_18238647, 6_18264470, 6_18339799, 6_18358603, 6_18372699, 6_18435054, 6_18441688, 6_18452236, 6_18464954, 6_18486179, 6_18527514, 6_18543760, 6_18561119, 6_18566150, 6_18566395, 6_18576657, 6_18602916, 6_18630215, 6_18666833, 6_18702297, 6_18702961, 6_18717222, 6_18718935, 6_18735745, 6_18745319, 6_18779512, 6_18782424, 6_18844592, 6_18855509, 6_18928501, 6_18931929, 6_18977208, 6_18977902, 6_19096841, 6_19110812, 6_19112423, 6_19118898, 6_19132738, 6_19145610, 6_19165758, 6_19176330, 6_19187266, 6_19222788, 6_19227625, 6_19246517, 6_19301751, 6_19306991, 6_19313573, 6_19317776, 6_19329519, 6_19365474, 6_19393102, 6_19393372, 6_19394860, 6_19396906, 6_19397619, 6_19443058, 6_19464444, 6_19470224, 6_19497907, 6_19504208, 6_19505759, 6_19508713, 6_19563490, 6_19567089, 6_19571371, 6_19577585, 6_19578810, 6_19641522, 6_19643558, 6_19645381, 6_19692090, 6_19705602, 6_19715056, 6_19722017, 6_19726603, 6_19736985, 6_19752891, 6_19781527, 6_19828599, 6_19846717, 6_19850602, 6_19851173, 6_19853640, 6_19926392, 6_19930830, 6_19970660, 6_19977515, 6_19993093, 6_20006420, 6_20006760, 6_20007512, 6_20009259, 6_20011385, 6_20028051, 6_20036588, 6_20053751, 6_20054906, 6_20056560, 6_20066024, 6_20070220, 6_20071344, 6_20077495, 6_20078919, 6_20079752, 6_20080858, 6_20082611, 6_20083630, 6_20087398, 6_20112017, 6_20135641, 6_20141062, 6_20188589, 6_20199873, 6_20201587, 6_20205073, 6_20227834, 6_20237201, 6_20239308, 6_20256812, 6_20258215, 6_20263826, 6_20265532, 6_20282649, 6_20310658, 6_20319899, 6_20334312, 6_20338165, 6_20369613, 6_20379795, 6_20386860, 6_20387195, 6_20391139, 6_20392461, 6_20408675, 6_20413060, 6_20442334, 6_20450121, 6_20451258, 6_20458439, 6_20480257, 6_20485571, 6_20505967, 6_20524557, 6_20528242, 6_20551510, 6_20564006, 6_20638168, 6_20649745, 6_20653984, 6_20692181, 6_20692256, 6_20710340, 6_20725127, 6_20769040, 6_20782306, 6_20782883, 6_20786109, 6_20788467, 6_20803465, 6_20804006, 6_20822396, 6_20822791, 6_20828331, 6_20830025, 6_20845158, 6_20873118, 6_20907636, 6_20931442, 6_20966429, 6_20982650, 6_20992775, 6_20998035, 6_21003221, 6_21016165, 6_21036003, 6_21041015, 6_21045975, 6_21055207, 6_21056849, 6_21066220, 6_21087126, 6_21101325, 6_21106960, 6_21109712, 6_21201109, 6_21221559, 6_21227724, 6_21233031, 6_21244212, 6_21250949, 6_21262155, 6_21266725, 6_21270015, 6_21272207, 6_21297844, 6_21351196, 6_21352792, 6_21376166, 6_21395565, 6_21407338, 6_21420087, 6_21462293, 6_21463991, 6_21478751, 6_21493136, 6_21505129, 6_21594574, 6_21687007, 6_21710556, 6_21716774, 6_21719148, 6_21722023, 6_21722238, 6_21725878, 6_21727311, 6_21744548, 6_21747540, 6_21773129, 6_21781961, 6_21812941, 6_21822845, 6_21834118, 6_21866875, 6_21878330, 6_21894396, 6_21929755, 6_21969674, 6_22014076, 6_22032249, 6_22036815, 6_22112535, 6_22128446, 6_22151927, 6_22178826, 6_22184325, 6_22222938, 6_22254417, 6_22366443, 6_22420658, 6_22446396, 6_22463815, 6_22560903, 6_22566194, 6_22570890, 6_22621673, 6_22658807, 6_22712471, 6_22728460, 6_22740695, 6_22741635, 6_22746715, 6_22818000, 6_22847973, 6_22880882, 6_22891745, 6_22916400, 6_23026062, 6_23026380, 6_23037304, 6_23042861, 6_23070111, 6_23093745, 6_23129816, 6_23181180, 6_23210882, 6_23245046, 6_23259337, 6_23271803, 6_23272374, 6_23337357, 6_23357632, 6_23391220, 6_23400723, 6_23488704, 6_23529006, 6_23622308, 6_23637666, 6_23641970, 6_23650713, 6_23704420, 6_23709695, 6_23712603, 6_23758798, 6_23758918, 6_23763713, 6_23782562, 6_23823441, 6_23824762, 6_23828347, 6_23828622, 6_23907949, 6_23913694, 6_23952469, 6_24003160, 6_24051601, 6_24079885, 6_24083388, 6_24107833, 6_24119663, 6_24122471, 6_24241609, 6_24295740, 6_24295809, 6_24331243, 6_24339394, 6_24387706, 6_24395786, 6_24451119, 6_24498390, 6_24553893, 6_24561302, 6_24600712, 6_24645527, 6_24702715, 6_24719932, 6_24722341, 6_24756525, 6_24816827, 6_24845641, 6_24872824, 6_24874603, 6_24928878, 6_24958128, 6_24994313, 6_25013902, 6_25023750, 6_25037092, 6_25039024, 6_25081054, 6_25114586, 6_25120814, 6_25179868, 6_25200878, 6_25206597, 6_25261156, 6_25261479, 6_25277805, 6_25299315, 6_25315198, 6_25318656, 6_25325115, 6_25338901, 6_25342323, 6_25355326, 6_25372478, 6_25375552, 6_25392309, 6_25408691, 6_25411531, 6_25443277, 6_25452996, 6_25468920, 6_25469233, 6_25470372, 6_25496055, 6_25517395, 6_25520008, 6_25549606, 6_25549968, 6_25554170, 6_25565957, 6_25567612, 6_25593135, 6_25630139, 6_25638325, 6_25640011, 6_25674300, 6_25687098, 6_25774222, 6_25792518, 6_25793290, 6_25819687, 6_25833257, 6_25841893, 6_25849943, 6_25865115, 6_25868001, 6_25894201, 6_25898114, 6_25909727, 6_25910021, 6_25931933, 6_25978010, 6_25982787, 6_25987370, 6_25993469, 6_25995790, 6_26000056, 6_26005006, 6_26007581, 6_26011520, 6_26058466, 6_26072196, 6_26076987, 6_26087807, 6_26092208, 6_26098253, 6_26102768, 6_26106886, 6_26111439, 6_26129547, 6_26133354, 6_26151076, 6_26152765, 6_26157760, 6_26198522, 6_26209945, 6_26215124, 6_26225037, 6_26229269, 6_26231897, 6_26239053, 6_26271329, 6_26276706, 6_26281091, 6_26287067, 6_26301714, 6_26318282, 6_26356199, 6_26369121, 6_26441054, 6_26482082, 6_26508456, 6_26509501, 6_26523132, 6_26531997, 6_26579913, 6_26605180, 6_26606738, 6_26612381, 6_26633347, 6_26637114, 6_26641811, 6_26643004, 6_26644033, 6_26648562, 6_26687741, 6_26699853, 6_26716297, 6_26768435, 6_26775754, 6_26783072, 6_26834604, 6_26838492, 6_26895636, 6_26944302, 6_26945553, 6_26946290, 6_26947947, 6_26950503, 6_26961722, 6_26997288, 6_26997782, 6_27057880, 6_27134606, 6_27146203, 6_27191580, 6_27285194, 6_27287200, 6_27290962, 6_27391349, 6_27407373, 7_2337, 7_8401, 7_9328, 7_13543, 7_20176, 7_26153, 7_27391, 7_34695, 7_60813, 7_84289, 7_85035, 7_86412, 7_86636, 7_89845, 7_93820, 7_94097, 7_116849, 7_151971, 7_167691, 7_182012, 7_186848, 7_237294, 7_245090, 7_246845, 7_268220, 7_268511, 7_278976, 7_284223, 7_290261, 7_307525, 7_324285, 7_324644, 7_399964, 7_497040, 7_503239, 7_505440, 7_505916, 7_516526, 7_521266, 7_530608, 7_603458, 7_630391, 7_637875, 7_638760, 7_651053, 7_662943, 7_664871, 7_669394, 7_672734, 7_733709, 7_751062, 7_770711, 7_777512, 7_787618, 7_789175, 7_795436, 7_802155, 7_816038, 7_819050, 7_836070, 7_836553, 7_837977, 7_838432, 7_856503, 7_861420, 7_903521, 7_924400, 7_931717, 7_944768, 7_983580, 7_988442, 7_1013063, 7_1014356, 7_1030760, 7_1059318, 7_1092837, 7_1108702, 7_1149209, 7_1161318, 7_1173810, 7_1237585, 7_1263302, 7_1269803, 7_1277246, 7_1277799, 7_1279344, 7_1283862, 7_1298196, 7_1323197, 7_1340796, 7_1355512, 7_1424083, 7_1432990, 7_1453605, 7_1458310, 7_1458650, 7_1474664, 7_1493206, 7_1536032, 7_1560262, 7_1563192, 7_1582377, 7_1596984, 7_1654629, 7_1655475, 7_1660283, 7_1660940, 7_1664801, 7_1718212, 7_1738080, 7_1775459, 7_1780267, 7_1794274, 7_1804823, 7_1808266, 7_1827723, 7_1831995, 7_1842368, 7_1921783, 7_1922834, 7_1934862, 7_1994605, 7_2002988, 7_2037135, 7_2037413, 7_2042472, 7_2045792, 7_2127764, 7_2137788, 7_2142005, 7_2147876, 7_2150256, 7_2166179, 7_2166417, 7_2178533, 7_2237699, 7_2255750, 7_2280934, 7_2281299, 7_2296421, 7_2299245, 7_2335072, 7_2335343, 7_2346490, 7_2394937, 7_2412159, 7_2430661, 7_2467200, 7_2475369, 7_2508123, 7_2521760, 7_2537511, 7_2542956, 7_2550810, 7_2551043, 7_2560436, 7_2568476, 7_2573764, 7_2622853, 7_2681527, 7_2726394, 7_2750435, 7_2755166, 7_2779889, 7_2789512, 7_2794400, 7_2809803, 7_2815372, 7_2859310, 7_2868682, 7_2874103, 7_2914025, 7_2924607, 7_2982356, 7_2984706, 7_3006437, 7_3013879, 7_3042454, 7_3094754, 7_3139737, 7_3144037, 7_3146522, 7_3164320, 7_3176488, 7_3181497, 7_3199771, 7_3199994, 7_3212160, 7_3219195, 7_3234740, 7_3284071, 7_3301476, 7_3306034, 7_3306782, 7_3323515, 7_3352121, 7_3356747, 7_3357353, 7_3367002, 7_3381877, 7_3390913, 7_3395274, 7_3397006, 7_3402637, 7_3409193, 7_3409464, 7_3412103, 7_3435644, 7_3454228, 7_3459363, 7_3497751, 7_3528981, 7_3530780, 7_3532115, 7_3532849, 7_3539455, 7_3549261, 7_3554717, 7_3575117, 7_3604697, 7_3609924, 7_3614244, 7_3622167, 7_3626600, 7_3630693, 7_3646987, 7_3693150, 7_3715729, 7_3748577, 7_3796518, 7_3796914, 7_3814022, 7_3828575, 7_3874064, 7_3874759, 7_3906177, 7_3925939, 7_3938639, 7_3939991, 7_3951876, 7_3951903, 7_3963772, 7_3998145, 7_4013064, 7_4017961, 7_4032622, 7_4060896, 7_4079889, 7_4085902, 7_4103466, 7_4115972, 7_4127666, 7_4128862, 7_4184766, 7_4188535, 7_4208030, 7_4211398, 7_4221059, 7_4252513, 7_4280408, 7_4299269, 7_4303987, 7_4304916, 7_4326667, 7_4340263, 7_4342719, 7_4345295, 7_4362596, 7_4364676, 7_4371250, 7_4379075, 7_4379310, 7_4403315, 7_4408291, 7_4429754, 7_4433063, 7_4433533, 7_4447959, 7_4477297, 7_4527825, 7_4528116, 7_4534791, 7_4554573, 7_4584583, 7_4601495, 7_4627189, 7_4644956, 7_4701700, 7_4715170, 7_4734241, 7_4880402, 7_4904307, 7_4978318, 7_4986539, 7_4990061, 7_4990546, 7_5011707, 7_5022412, 7_5024977, 7_5057500, 7_5075852, 7_5096871, 7_5102140, 7_5115598, 7_5137284, 7_5153679, 7_5162489, 7_5192844, 7_5239110, 7_5245709, 7_5252201, 7_5258218, 7_5285077, 7_5323951, 7_5325752, 7_5356358, 7_5374288, 7_5395904, 7_5421031, 7_5468809, 7_5474418, 7_5487470, 7_5512528, 7_5540789, 7_5587660, 7_5608974, 7_5618762, 7_5643478, 7_5659897, 7_5673356, 7_5716596, 7_5762774, 7_5762846, 7_5791680, 7_5852731, 7_5856946, 7_5899983, 7_5922706, 7_5923580, 7_5942196, 7_5942527, 7_5957442, 7_5967591, 7_6005766, 7_6019870, 7_6019954, 7_6032341, 7_6032545, 7_6071073, 7_6126514, 7_6145372, 7_6213693, 7_6217932, 7_6226413, 7_6254184, 7_6266233, 7_6275775, 7_6303719, 7_6341508, 7_6362102, 7_6364342, 7_6385071, 7_6405489, 7_6416279, 7_6431139, 7_6501710, 7_6523915, 7_6523982, 7_6536566, 7_6549812, 7_6575390, 7_6665202, 7_6676079, 7_6676209, 7_6701471, 7_6705774, 7_6718234, 7_6752452, 7_6766862, 7_6767558, 7_6768779, 7_6826989, 7_6867435, 7_6900520, 7_6902579, 7_6908791, 7_6949735, 7_6965921, 7_7016868, 7_7017210, 7_7036056, 7_7046067, 7_7060182, 7_7073360, 7_7073840, 7_7117095, 7_7125078, 7_7162827, 7_7202909, 7_7227141, 7_7341369, 7_7391193, 7_7406286, 7_7435193, 7_7445536, 7_7483166, 7_7484396, 7_7490856, 7_7513864, 7_7619389, 7_7640760, 7_7646150, 7_7687210, 7_7697667, 7_7801941, 7_7811261, 7_7888204, 7_7902052, 7_7912194, 7_7926225, 7_7937895, 7_7957124, 7_7964564, 7_7995772, 7_7999802, 7_8020511, 7_8047331, 7_8061660, 7_8097768, 7_8150179, 7_8154717, 7_8207555, 7_8231855, 7_8245065, 7_8245406, 7_8247489, 7_8252999, 7_8281517, 7_8306613, 7_8311076, 7_8313098, 7_8324949, 7_8336854, 7_8348191, 7_8362886, 7_8385591, 7_8390686, 7_8491332, 7_8495161, 7_8497883, 7_8515742, 7_8516428, 7_8580401, 7_8612225, 7_8614529, 7_8621712, 7_8623673, 7_8633337, 7_8679355, 7_8689604, 7_8693242, 7_8694008, 7_8703904, 7_8707120, 7_8711383, 7_8810414, 7_8819234, 7_8849145, 7_8959596, 7_8984316, 7_8991307, 7_8999834, 7_9001517, 7_9073306, 7_9158167, 7_9165963, 7_9205330, 7_9227552, 7_9230590, 7_9243020, 7_9282571, 7_9285977, 7_9286472, 7_9297510, 7_9305751, 7_9356019, 7_9357470, 7_9360079, 7_9390592, 7_9391777, 7_9418030, 7_9463194, 7_9536728, 7_9537092, 7_9557906, 7_9614017, 7_9615169, 7_9618035, 7_9628888, 7_9659762, 7_9662498, 7_9673608, 7_9691738, 7_9702916, 7_9705985, 7_9728351, 7_9733686, 7_9743443, 7_9819885, 7_9821679, 7_9831730, 7_9894773, 7_9913604, 7_9980122, 7_10059611, 7_10091009, 7_10130162, 7_10164364, 7_10198433, 7_10252098, 7_10283127, 7_10287393, 7_10355089, 7_10362253, 7_10445451, 7_10466981, 7_10512107, 7_10522176, 7_10555405, 7_10606530, 7_10655001, 7_10680530, 7_10731424, 7_10738008, 7_10762619, 7_10827256, 7_10836337, 7_10876163, 7_10921806, 7_10955926, 7_10956308, 7_10958004, 7_10961512, 7_11005282, 7_11103838, 7_11189723, 7_11202100, 7_11242166, 7_11251999, 7_11277821, 7_11281320, 7_11320525, 7_11358728, 7_11369946, 7_11378526, 7_11393419, 7_11424780, 7_11428122, 7_11434166, 7_11458079, 7_11546668, 7_11548289, 7_11555337, 7_11570345, 7_11590709, 7_11600122, 7_11602745, 7_11606077, 7_11640633, 7_11641903, 7_11642422, 7_11643232, 7_11646249, 7_11651739, 7_11728324, 7_11740553, 7_11842294, 7_11932226, 7_11981039, 7_11996966, 7_12010322, 7_12016881, 7_12099746, 7_12196773, 7_12202663, 7_12209902, 7_12225157, 7_12229478, 7_12250637, 7_12257088, 7_12265548, 7_12274042, 7_12309499, 7_12309844, 7_12317072, 7_12411343, 7_12439765, 7_12463494, 7_12471828, 7_12508645, 7_12515148, 7_12553048, 7_12577585, 7_12600553, 7_12652928, 7_12656064, 7_12679521, 7_12679782, 7_12707622, 7_12707884, 7_12770239, 7_12792391, 7_12803532, 7_12815792, 7_12881180, 7_13062432, 7_13080687, 7_13145534, 7_13182343, 7_13254669, 7_13301298, 7_13340062, 7_13368648, 7_13385763, 7_13395202, 7_13478147, 7_13492225, 7_13578821, 7_13581128, 7_13581808, 7_13594490, 7_13653349, 7_13669308, 7_13677020, 7_13683832, 7_13734406, 7_13740998, 7_13742391, 7_13796333, 7_13801573, 7_13826449, 7_13832653, 7_13868930, 7_13870371, 7_13886755, 7_13924532, 7_13947509, 7_13983386, 7_13984890, 7_14006360, 7_14029325, 7_14053559, 7_14054393, 7_14090207, 7_14097895, 7_14115688, 7_14120007, 7_14126641, 7_14126795, 7_14133068, 7_14149829, 7_14157361, 7_14193463, 7_14240568, 7_14248190, 7_14339281, 7_14340440, 7_14361656, 7_14376386, 7_14408790, 7_14409264, 7_14426126, 7_14466677, 7_14469699, 7_14516849, 7_14521290, 7_14544245, 7_14560570, 7_14563892, 7_14572984, 7_14573521, 7_14578177, 7_14583591, 7_14587147, 7_14604427, 7_14618364, 7_14631583, 7_14640438, 7_14643246, 7_14721443, 7_14735812, 7_14743966, 7_14747191, 7_14750822, 7_14779534, 7_14807434, 7_14821303, 7_14828283, 7_14830126, 7_14883578, 7_14883673, 7_14908556, 7_14949298, 7_14963945, 7_15002528, 7_15019710, 7_15024581, 7_15036946, 7_15037495, 7_15054666, 7_15077394, 7_15081329, 7_15084815, 7_15097713, 7_15146509, 7_15157000, 7_15162769, 7_15180172, 7_15180658, 7_15189579, 7_15190306, 7_15190516, 7_15208042, 7_15223678, 7_15237116, 7_15263902, 7_15269713, 7_15278094, 7_15283609, 7_15288960, 7_15299726, 7_15315235, 7_15315431, 7_15451115, 7_15452136, 7_15509082, 7_15541643, 7_15567348, 7_15569857, 7_15578852, 7_15579072, 7_15580202, 7_15580459, 7_15580805, 7_15586599, 7_15590432, 7_15613184, 7_15613431, 7_15640072, 7_15652198, 7_15653745, 7_15675631, 7_15692177, 7_15760266, 7_15765691, 7_15781945, 7_15785111, 7_15797019, 7_15822554, 7_15822800, 7_15832649, 7_15855680, 7_15864142, 7_15867482, 7_15874197, 7_15886172, 7_15938180, 7_15944241, 7_15963474, 7_15963663, 7_15966824, 7_15991340, 7_16007443, 7_16007640, 7_16032820, 7_16058971, 7_16072358, 7_16116769, 7_16145738, 7_16151704, 7_16192619, 7_16208114, 7_16235453, 7_16266184, 7_16271981, 7_16281590, 7_16344241, 7_16347431, 7_16440732, 7_16446164, 7_16446428, 7_16449701, 7_16558743, 7_16616608, 7_16619358, 7_16636810, 7_16639294, 7_16663289, 7_16731041, 7_16785235, 7_16814319, 7_16824163, 7_16836233, 7_16847538, 7_16858734, 7_16908599, 7_16923603, 7_16924188, 7_17001209, 7_17035577, 7_17036512, 7_17051281, 7_17080631, 7_17103306, 7_17153018, 7_17199787, 7_17272187, 7_17273395, 7_17294966, 7_17307544, 7_17353025, 7_17355252, 7_17377075, 7_17390671, 7_17398531, 7_17398962, 7_17461510, 7_17488783, 7_17492128, 7_17493873, 7_17531071, 7_17534952, 7_17552559, 7_17558287, 7_17595116, 7_17656253, 7_17666330, 7_17673877, 7_17695509, 7_17767155, 7_17797048, 7_17801504, 7_17810353, 7_17812085, 7_17814344, 7_17836928, 7_17863151, 7_17863251, 7_17871063, 7_17965650, 7_18055805, 7_18071311, 7_18114802, 7_18129767, 7_18157649, 7_18242606, 7_18311532, 7_18327930, 7_18344789, 7_18346891, 7_18352651, 7_18356260, 7_18381345, 7_18385746, 7_18400813, 7_18401394, 7_18408542, 7_18409780, 7_18435569, 7_18534423, 7_18539838, 7_18654726, 7_18665352, 7_18796998, 7_18811865, 7_18831692, 7_18833135, 7_18833340, 7_18917984, 7_18952830, 7_18987881, 7_19034383, 7_19074859, 7_19150894, 7_19210630, 7_19234723, 7_19240234, 7_19308273, 7_19336000, 7_19348102, 7_19356435, 7_19360673, 7_19374515, 7_19414455, 7_19434146, 7_19437010, 7_19457058, 7_19506274, 7_19506360, 7_19565942, 7_19667777, 7_19690879, 7_19753713, 7_19772456, 7_19788824, 7_19829802, 7_19885662, 7_19986770, 7_20008448, 7_20013477, 7_20054262, 7_20074819, 7_20078465, 7_20089526, 7_20135538, 7_20137107, 7_20143769, 7_20175011, 7_20197398, 7_20214061, 7_20222898, 7_20234479, 7_20244684, 7_20278026, 7_20278245, 7_20289945, 7_20304481, 7_20342598, 7_20392876, 7_20401867, 7_20421503, 7_20467752, 7_20478732, 7_20485360, 7_20494318, 7_20522682, 7_20525564, 7_20545434, 7_20557000, 7_20595892, 7_20601082, 7_20626792, 7_20692892, 7_20699094, 7_20751013, 7_20772206, 7_20794448, 7_20798990, 7_20817799, 7_20864800, 7_20909246, 7_20951178, 7_20951378, 7_20979107, 7_21022459, 7_21046193, 7_21065691, 7_21103123, 7_21114179, 7_21118484, 7_21122454, 7_21138474, 7_21150739, 7_21190429, 7_21208629, 7_21263272, 7_21267649, 7_21287064, 7_21287700, 7_21297237, 7_21305192, 7_21305261, 7_21306625, 7_21316292, 7_21326747, 7_21366805, 7_21367186, 7_21369708, 7_21374810, 7_21404420, 7_21404821, 7_21441126, 7_21528895, 7_21535188, 7_21583196, 7_21625430, 7_21630659, 7_21641121, 7_21694300, 7_21749867, 7_21750216, 7_21754674, 7_21759430, 7_21761559, 7_21775774, 7_21782821, 7_21804958, 7_21818652, 7_21827772, 7_21837778, 7_21848171, 7_21857365, 7_21862601, 7_21874763, 7_21888324, 7_21894341, 7_21899636, 7_21910687, 7_21912888, 7_21928565, 7_21982349, 7_21991543, 7_21994079, 7_21994465, 7_21995524, 7_21997107, 7_22057720, 7_22058093, 7_22077769, 7_22103049, 7_22123654, 7_22137554, 7_22203784, 7_22204214, 7_22224253, 7_22257129, 7_22295742, 7_22303782, 7_22307960, 7_22309383, 7_22322729, 7_22344678, 7_22350839, 7_22360789, 7_22362225, 7_22365321, 7_22373138, 7_22411211, 7_22443064, 7_22454274, 7_22504997, 7_22529573, 7_22559889, 7_22606211, 7_22626315, 7_22689520, 7_22697515, 7_22732601, 7_22743806, 7_22752453, 7_22857941, 7_22863436, 7_22876519, 7_22893823, 7_22907342, 7_22930348, 7_22959509, 7_22977211, 7_23048655, 7_23053432, 7_23067985, 7_23072930, 7_23075945, 7_23110002, 7_23145241, 7_23171984, 7_23185272, 7_23239703, 7_23252625, 7_23273343, 7_23287304, 7_23299644, 7_23347209, 7_23440520, 7_23469158, 7_23497321, 7_23497581, 7_23500851, 7_23509573, 7_23550121, 7_23553958, 7_23568331, 7_23577214, 7_23582766, 7_23582826, 7_23611377, 7_23633266, 7_23655103, 7_23678963, 7_23679357, 7_23723101, 7_23740909, 7_23751782, 7_23755813, 7_23756731, 7_23766070, 7_23777998, 7_23798552, 7_23823867, 7_23832762, 7_23875308, 7_23893617, 7_23898367, 7_23901556, 7_23912735, 7_23924778, 7_23931854, 7_23940720, 7_24005304, 7_24010234, 7_24075298, 7_24090971, 7_24109816, 7_24120993, 7_24132443, 7_24160820, 7_24161447, 7_24167325, 7_24172609, 7_24196401, 7_24200632, 7_24294159, 7_24298452, 7_24305486, 7_24333460, 7_24335145, 7_24353235, 7_24397783, 7_24409594, 7_24409738, 7_24410843, 7_24411179, 7_24411439, 7_24421209, 7_24446991, 7_24459512, 7_24491470, 7_24517873, 7_24530446, 7_24536179, 7_24560131, 7_24570661, 7_24594507, 7_24606272, 7_24626951, 7_24627784, 7_24632349, 7_24632958, 7_24756613, 7_24782382, 7_24782663, 7_24790455, 7_24802780, 7_24811577, 7_24834119, 7_24853494, 7_24910573, 7_24916126, 7_24947594, 7_24963266, 7_25012604, 7_25022492, 7_25054446, 7_25176065, 7_25180554, 7_25278782, 7_25302075, 7_25304154, 7_25307976, 7_25346408, 7_25369878, 7_25461266, 7_25461560, 7_25477850, 7_25555986, 7_25604060, 7_25636273, 7_25638748, 7_25646044, 7_25678997, 7_25748553, 7_25769448, 7_25775651, 7_25824843, 7_25895311, 7_25902158, 7_26011371, 7_26045035, 7_26086542, 7_26086762, 7_26089711, 7_26134669, 7_26184503, 7_26306839, 7_26322665, 7_26470843, 7_26527982, 7_26545180, 7_26574083, 7_26594336, 7_26627374, 7_26640679, 7_26665731, 7_26709219, 7_26725285, 7_26781081, 7_26790175, 8_16991, 8_18836, 8_38586, 8_40454, 8_47257, 8_89337, 8_97501, 8_112742, 8_115275, 8_170934, 8_180412, 8_208666, 8_219311, 8_227783, 8_235516, 8_236057, 8_247447, 8_264416, 8_286419, 8_289751, 8_298116, 8_306549, 8_321229, 8_328626, 8_341388, 8_357019, 8_373113, 8_386399, 8_386819, 8_387596, 8_392571, 8_393687, 8_400779, 8_402832, 8_435180, 8_443778, 8_443928, 8_444864, 8_482496, 8_499920, 8_499977, 8_500436, 8_508324, 8_511008, 8_520634, 8_537540, 8_542533, 8_551148, 8_584739, 8_604190, 8_604670, 8_624649, 8_632530, 8_647805, 8_650681, 8_654210, 8_656935, 8_667072, 8_668930, 8_688911, 8_692005, 8_705954, 8_706533, 8_726496, 8_752309, 8_770297, 8_824349, 8_830269, 8_830627, 8_835253, 8_859894, 8_866229, 8_879727, 8_880534, 8_956167, 8_957003, 8_965219, 8_1004793, 8_1022606, 8_1029869, 8_1038247, 8_1089618, 8_1137527, 8_1155237, 8_1170496, 8_1184192, 8_1259307, 8_1275682, 8_1305129, 8_1336923, 8_1346126, 8_1388259, 8_1388743, 8_1440332, 8_1454574, 8_1465813, 8_1487169, 8_1511860, 8_1518072, 8_1520473, 8_1523930, 8_1524260, 8_1528657, 8_1544751, 8_1550652, 8_1564088, 8_1573667, 8_1607322, 8_1608162, 8_1608807, 8_1747690, 8_1749502, 8_1797367, 8_1815819, 8_1921846, 8_1922083, 8_1931041, 8_1936793, 8_1952319, 8_1953618, 8_1984283, 8_1997071, 8_2007029, 8_2008672, 8_2011858, 8_2035069, 8_2052607, 8_2061084, 8_2069054, 8_2073652, 8_2096056, 8_2119401, 8_2136003, 8_2184853, 8_2203085, 8_2261833, 8_2290255, 8_2295208, 8_2322670, 8_2329832, 8_2357158, 8_2388718, 8_2389198, 8_2393387, 8_2401182, 8_2403927, 8_2404707, 8_2410301, 8_2426391, 8_2446142, 8_2468815, 8_2474071, 8_2495536, 8_2498652, 8_2517976, 8_2539218, 8_2564982, 8_2583609, 8_2595501, 8_2603615, 8_2616858, 8_2639208, 8_2651808, 8_2665619, 8_2670989, 8_2735408, 8_2772734, 8_2775132, 8_2775558, 8_2780184, 8_2806519, 8_2863367, 8_2907359, 8_2912278, 8_2946405, 8_2954353, 8_2981313, 8_2992118, 8_2992261, 8_2992551, 8_3000328, 8_3004674, 8_3006023, 8_3076637, 8_3084128, 8_3087577, 8_3093730, 8_3097189, 8_3128714, 8_3142098, 8_3150660, 8_3152476, 8_3155756, 8_3170172, 8_3180182, 8_3187907, 8_3190788, 8_3210385, 8_3221997, 8_3231262, 8_3277866, 8_3281565, 8_3288840, 8_3291840, 8_3293328, 8_3298839, 8_3351329, 8_3388229, 8_3407221, 8_3410680, 8_3420832, 8_3432772, 8_3433302, 8_3464984, 8_3471197, 8_3488592, 8_3503496, 8_3508290, 8_3510840, 8_3517481, 8_3521321, 8_3540497, 8_3552511, 8_3585423, 8_3589034, 8_3608283, 8_3645991, 8_3648117, 8_3658522, 8_3668413, 8_3736858, 8_3788587, 8_3797886, 8_3799560, 8_3808441, 8_3838442, 8_3861227, 8_3867000, 8_3874840, 8_3894169, 8_3894393, 8_3899505, 8_3921834, 8_3967318, 8_3973217, 8_3976047, 8_3999518, 8_4012112, 8_4015446, 8_4051013, 8_4052487, 8_4052715, 8_4067418, 8_4082887, 8_4091636, 8_4091875, 8_4108203, 8_4114628, 8_4135597, 8_4183469, 8_4266879, 8_4292152, 8_4295245, 8_4302538, 8_4307904, 8_4313829, 8_4318372, 8_4356435, 8_4357397, 8_4381973, 8_4384791, 8_4394715, 8_4432085, 8_4433136, 8_4469170, 8_4472141, 8_4495645, 8_4522862, 8_4537511, 8_4541462, 8_4558297, 8_4569635, 8_4592386, 8_4614407, 8_4657509, 8_4673440, 8_4705462, 8_4718492, 8_4725216, 8_4749623, 8_4753221, 8_4775710, 8_4776308, 8_4829063, 8_4847390, 8_4857800, 8_4859568, 8_4866751, 8_4869747, 8_4901812, 8_4906893, 8_4911918, 8_4913705, 8_4923151, 8_4934007, 8_4937844, 8_4953003, 8_4957300, 8_4965990, 8_4999775, 8_5002311, 8_5012464, 8_5014890, 8_5015204, 8_5033757, 8_5084135, 8_5092409, 8_5092482, 8_5106652, 8_5106879, 8_5113577, 8_5116935, 8_5159158, 8_5161282, 8_5183941, 8_5216190, 8_5260057, 8_5270981, 8_5279830, 8_5298966, 8_5318275, 8_5325557, 8_5340305, 8_5364237, 8_5370913, 8_5436746, 8_5467319, 8_5544530, 8_5608903, 8_5610947, 8_5614181, 8_5621371, 8_5623881, 8_5625992, 8_5644400, 8_5645708, 8_5662252, 8_5671731, 8_5682504, 8_5772961, 8_5789315, 8_5789538, 8_5817095, 8_5822569, 8_5828449, 8_5843208, 8_5843254, 8_5843553, 8_5847426, 8_5869293, 8_5869474, 8_5904375, 8_5939822, 8_5974197, 8_5976073, 8_5978437, 8_5988327, 8_6001684, 8_6002608, 8_6004539, 8_6005712, 8_6009719, 8_6019192, 8_6025973, 8_6054579, 8_6075515, 8_6079250, 8_6082834, 8_6095651, 8_6104122, 8_6112385, 8_6161404, 8_6170432, 8_6175908, 8_6186588, 8_6201542, 8_6263855, 8_6333062, 8_6344268, 8_6347116, 8_6374278, 8_6393460, 8_6398512, 8_6407508, 8_6454508, 8_6467364, 8_6480309, 8_6480560, 8_6538985, 8_6567043, 8_6589321, 8_6624093, 8_6678104, 8_6678456, 8_6682170, 8_6787385, 8_6795623, 8_6804017, 8_6873865, 8_6876101, 8_6878578, 8_6892985, 8_6902811, 8_6914485, 8_6915901, 8_6925054, 8_6938006, 8_6953406, 8_6996624, 8_7007052, 8_7064369, 8_7104355, 8_7107816, 8_7114223, 8_7126922, 8_7127999, 8_7128691, 8_7133937, 8_7166184, 8_7184446, 8_7240089, 8_7311586, 8_7315791, 8_7316237, 8_7320266, 8_7325458, 8_7347327, 8_7347761, 8_7383413, 8_7384362, 8_7393248, 8_7405160, 8_7436212, 8_7482835, 8_7501965, 8_7514853, 8_7524341, 8_7536401, 8_7541973, 8_7624482, 8_7629023, 8_7659504, 8_7701253, 8_7724199, 8_7730751, 8_7807478, 8_7817315, 8_7917155, 8_7977456, 8_8038257, 8_8075775, 8_8104576, 8_8127595, 8_8141690, 8_8151216, 8_8160914, 8_8167149, 8_8220486, 8_8240908, 8_8279016, 8_8292475, 8_8326826, 8_8428209, 8_8453513, 8_8479571, 8_8498947, 8_8511743, 8_8547082, 8_8554035, 8_8646096, 8_8701534, 8_8767007, 8_8779229, 8_8791962, 8_8802117, 8_8805021, 8_8825081, 8_8864501, 8_8939570, 8_8986546, 8_8994726, 8_9030529, 8_9042320, 8_9125300, 8_9131275, 8_9133307, 8_9134032, 8_9142768, 8_9229732, 8_9239397, 8_9252918, 8_9273096, 8_9274623, 8_9277956, 8_9289904, 8_9292185, 8_9325858, 8_9330673, 8_9340640, 8_9371149, 8_9372420, 8_9393460, 8_9451796, 8_9486653, 8_9490723, 8_9523393, 8_9540065, 8_9588954, 8_9624145, 8_9649824, 8_9659774, 8_9682691, 8_9684564, 8_9758551, 8_9782355, 8_9929496, 8_10007556, 8_10012267, 8_10033284, 8_10068792, 8_10108083, 8_10121724, 8_10122207, 8_10125278, 8_10136924, 8_10247094, 8_10262747, 8_10311142, 8_10373456, 8_10407484, 8_10410672, 8_10420540, 8_10528971, 8_10583726, 8_10588714, 8_10679139, 8_10707917, 8_10738194, 8_10761370, 8_10783068, 8_10861886, 8_10896947, 8_10901268, 8_10950079, 8_10959623, 8_10960936, 8_10972457, 8_11024144, 8_11144719, 8_11153907, 8_11169433, 8_11196622, 8_11202390, 8_11204360, 8_11224287, 8_11238426, 8_11269333, 8_11293259, 8_11297078, 8_11314453, 8_11388679, 8_11393117, 8_11405951, 8_11419214, 8_11456045, 8_11460915, 8_11539739, 8_11581179, 8_11600950, 8_11668860, 8_11790612, 8_11792622, 8_11814476, 8_11816033, 8_11859777, 8_11875191, 8_11938734, 8_11988557, 8_12029366, 8_12036188, 8_12114363, 8_12140632, 8_12209827, 8_12232000, 8_12262753, 8_12271005, 8_12302555, 8_12312703, 8_12312967, 8_12313383, 8_12512552, 8_12545700, 8_12546990, 8_12571775, 8_12630849, 8_12643181, 8_12713307, 8_12718918, 8_12728785, 8_12729150, 8_12749011, 8_12771411, 8_12771890, 8_12773597, 8_12798665, 8_12807073, 8_12822534, 8_12862537, 8_12875943, 8_12893833, 8_12936447, 8_12957765, 8_12971258, 8_12976162, 8_12980407, 8_13015482, 8_13033382, 8_13056598, 8_13070003, 8_13078768, 8_13112240, 8_13113884, 8_13114317, 8_13126129, 8_13129698, 8_13132050, 8_13190342, 8_13200653, 8_13208943, 8_13213626, 8_13265254, 8_13290231, 8_13351744, 8_13508505, 8_13511323, 8_13535218, 8_13538267, 8_13565733, 8_13575064, 8_13606035, 8_13623731, 8_13628447, 8_13651875, 8_13716167, 8_13717310, 8_13750968, 8_13751213, 8_13777378, 8_13799927, 8_13905085, 8_13922418, 8_13937138, 8_13980851, 8_14001185, 8_14019781, 8_14035892, 8_14040390, 8_14041212, 8_14070263, 8_14073001, 8_14104980, 8_14113253, 8_14118761, 8_14147092, 8_14188093, 8_14292847, 8_14373372, 8_14374044, 8_14378120, 8_14411393, 8_14430554, 8_14441193, 8_14485842, 8_14497180, 8_14586582, 8_14592834, 8_14597073, 8_14598816, 8_14621618, 8_14630640, 8_14642167, 8_14679155, 8_14706829, 8_14748695, 8_14755619, 8_14758983, 8_14785024, 8_14835475, 8_14851937, 8_14868404, 8_14978889, 8_14985796, 8_14999190, 8_15009682, 8_15025337, 8_15062760, 8_15084089, 8_15163242, 8_15163546, 8_15184128, 8_15257025, 8_15264313, 8_15271041, 8_15286806, 8_15291372, 8_15385122, 8_15396458, 8_15487914, 8_15488657, 8_15514920, 8_15630146, 8_15698223, 8_15730249, 8_15752637, 8_15867134, 8_15870967, 8_16012443, 8_16031421, 8_16067978, 8_16073859, 8_16104020, 8_16104710, 8_16113833, 8_16146795, 8_16237066, 8_16245679, 8_16307127, 8_16321758, 8_16370022, 8_16379925, 8_16416794, 8_16418751, 8_16427990, 8_16441064, 8_16441509, 8_16451088, 8_16489421, 8_16493851, 8_16498857, 8_16502872, 8_16520773, 8_16527508, 8_16570437, 8_16580106, 8_16584659, 8_16587822, 8_16615180, 8_16616037, 8_16622471, 8_16630160, 8_16646301, 8_16654149, 8_16655159, 8_16660769, 8_16661609, 8_16669456, 8_16676268, 8_16686822, 8_16687637, 8_16691097, 8_16709098, 8_16714964, 8_16720623, 8_16752590, 8_16754379, 8_16764914, 8_16772666, 8_16781173, 8_16783465, 8_16801123, 8_16812502, 8_16817234, 8_16817566, 8_16832218, 8_16842984, 8_16869451, 8_16872855, 8_16892750, 8_16900035, 8_16950965, 8_16970176, 8_16972338, 8_16973694, 8_16976686, 8_17013782, 8_17032972, 8_17035013, 8_17089105, 8_17126578, 8_17152159, 8_17153398, 8_17208105, 8_17252144, 8_17327070, 8_17341534, 8_17343125, 8_17379477, 8_17385484, 8_17388116, 8_17404157, 8_17405735, 8_17445174, 8_17538424, 8_17556188, 8_17558925, 8_17561977, 8_17562400, 8_17564974, 8_17579415, 8_17596654, 8_17596971, 8_17632746, 8_17651149, 8_17684781, 8_17692936, 8_17693404, 8_17696886, 8_17706518, 8_17707561, 8_17708416, 8_17708844, 8_17718827, 8_17724317, 8_17727540, 8_17765341, 8_17775126, 8_17788891, 8_17811759, 8_17815424, 8_17815624, 8_17851508, 8_17857280, 8_17858835, 8_17868875, 8_17872679, 8_17887032, 8_17901892, 8_17920583, 8_17953078, 8_17985700, 8_18081524, 8_18087118, 8_18093948, 8_18094900, 8_18101174, 8_18121289, 8_18126640, 8_18133624, 8_18133754, 8_18156267, 8_18207606, 8_18219547, 8_18294459, 8_18310670, 8_18324336, 8_18327619, 8_18335663, 8_18359702, 8_18394638, 8_18398006, 8_18399663, 8_18403524, 8_18404068, 8_18404452, 8_18413652, 8_18417406, 8_18426903, 8_18439212, 8_18440028, 8_18444943, 8_18454365, 8_18455954, 8_18459856, 8_18471217, 8_18481147, 8_18499686, 8_18518901, 8_18524398, 8_18529487, 8_18535328, 8_18535740, 8_18548682, 8_18571751, 8_18608356, 8_18614057, 8_18614409, 8_18621523, 8_18638980, 8_18643165, 8_18661143, 8_18667128, 8_18667991, 8_18711295, 8_18713991, 8_18739143, 8_18741529, 8_18751215, 8_18776323, 8_18781292, 8_18799600, 8_18823604, 8_18869928, 8_18951257, 8_18968784, 8_18987946, 8_19073153, 8_19077791, 8_19078287, 8_19082939, 8_19084468, 8_19113142, 8_19114685, 8_19115729, 8_19128129, 8_19157030, 8_19160449, 8_19161922, 8_19165518, 8_19174372, 8_19183603, 8_19188109, 8_19199725, 8_19210262, 8_19226132, 8_19235119, 8_19235227, 8_19242670, 8_19263611, 8_19267557, 8_19281585, 8_19282583, 8_19305991, 8_19318216, 8_19320444, 8_19325292, 8_19330811, 8_19337202, 8_19345353, 8_19357916, 8_19376190, 8_19376398, 8_19381001, 8_19400678, 8_19434908, 8_19437948, 8_19454809, 8_19505462, 8_19601358, 8_19630301, 8_19670795, 8_19671037, 8_19672474, 8_19674659, 8_19675929, 8_19684851, 8_19706612, 8_19819288, 8_19956256, 8_19982218, 8_20006444, 8_20074839, 8_20088413, 8_20127817, 8_20128536, 8_20146515, 8_20147830, 8_20152618, 8_20185470, 8_20202005, 8_20252547, 8_20301055, 8_20330445, 8_20330757, 8_20345353, 8_20362753, 8_20364405, 8_20364717, 8_20376533, 8_20381726, 8_20384333, 8_20396415, 8_20396796, 8_20397851, 8_20410744, 8_20455121, 8_20476067, 8_20480825, 8_20495255, 8_20497394, 8_20532232, 8_20532864, 8_20538220, 8_20546223, 8_20633294, 8_20657787, 8_20675420, 8_20717993, 8_20730248, 8_20792623, 8_20817097, 8_20842380, 8_20881673, 8_20890647, 8_20922152, 8_20922522, 8_20925615, 8_20942814, 8_21003850, 8_21012706, 8_21013009, 8_21052874, 8_21054639, 8_21059521, 8_21118959, 8_21119635, 8_21157625, 8_21187248, 8_21189022, 8_21206064, 8_21207508, 8_21216112, 8_21225728, 8_21263160, 8_21268927, 8_21298918, 8_21339227, 8_21363563, 8_21433386, 8_21463057, 8_21491923, 8_21497583, 8_21501067, 8_21501323, 8_21511750, 8_21548588, 8_21570430, 8_21580603, 8_21689284, 8_21694151, 8_21731947, 8_21744398, 8_21787337, 8_21816273, 8_21862336, 8_21971837, 8_21987645, 8_21991522, 8_22002792, 8_22009733, 8_22009925, 8_22017445, 8_22020789, 8_22099286, 8_22108533, 8_22147362, 8_22181319, 8_22303270, 8_22426888, 8_22464454, 8_22512866, 8_22570905, 8_22585039, 8_22590543, 8_22608307, 8_22647004, 8_22668012, 8_22675194, 8_22781711, 8_22789006, 8_22792110, 8_22820472, 8_22821013, 8_22856195, 8_22871505, 8_22897848, 8_22917724, 8_22929548, 8_22934360, 8_22949542, 8_22966281, 8_22967459, 8_23023853, 8_23123450, 8_23142858, 8_23154823, 8_23155091, 8_23155131, 8_23178455, 8_23191357, 8_23192926, 8_23204532, 8_23258544, 8_23275310, 8_23281262, 8_23281768, 8_23304383, 8_23308639, 8_23309791, 8_23339869, 8_23352541, 8_23372586, 8_23380693, 8_23385822, 8_23482646, 8_23515311, 8_23523943, 8_23540748, 8_23635853, 8_23645466, 8_23651460, 8_23690221, 8_23751711, 8_23810924, 8_23834154, 8_23839029, 8_23902600, 8_23932705, 8_23944924, 8_23953341, 8_23956718, 8_23968642, 8_23991635, 8_24005981, 8_24009853, 8_24011961, 8_24024011, 8_24035986, 8_24048904, 8_24050427, 8_24052555, 8_24069963, 8_24091601, 8_24094501, 8_24097356, 8_24103403, 8_24159793, 8_24160053, 8_24163611, 8_24171381, 8_24176482, 8_24228525, 8_24231617, 8_24250955, 8_24251455, 8_24277712, 8_24334735, 8_24346653, 8_24382237, 8_24438595, 8_24449735, 8_24458624, 8_24459874, 8_24515402, 8_24582852, 8_24590562, 8_24639003, 8_24642058, 8_24692698, 8_24705012, 8_24853216, 8_24880391, 8_24892181, 8_24969225, 8_24977560, 8_24978233, 8_24982120, 8_24984651, 8_25064379, 8_25102541, 8_25102965, 8_25195456, 8_25221068, 8_25261716, 8_25271136, 8_25308814, 8_25326088, 8_25328484, 8_25346239, 8_25352964, 8_25357236, 8_25457647, 8_25472686, 8_25531499, 8_25603844, 8_25646058, 8_25668792, 8_25692264, 8_25698395, 8_25709754, 8_25722110, 9_22123, 9_26461, 9_26581, 9_31754, 9_49324, 9_62423, 9_71869, 9_77181, 9_78491, 9_90926, 9_91261, 9_94032, 9_110870, 9_126461, 9_134308, 9_149664, 9_185518, 9_200890, 9_209392, 9_224443, 9_254568, 9_291918, 9_294699, 9_297818, 9_313360, 9_327065, 9_338683, 9_341514, 9_363461, 9_367291, 9_379989, 9_388167, 9_388826, 9_420281, 9_439130, 9_442104, 9_451641, 9_472104, 9_474690, 9_475399, 9_478057, 9_484678, 9_487302, 9_559369, 9_571874, 9_572688, 9_578814, 9_597115, 9_661351, 9_675364, 9_675484, 9_689763, 9_694290, 9_703639, 9_712266, 9_716274, 9_719870, 9_721031, 9_737190, 9_739001, 9_816518, 9_826823, 9_831953, 9_838477, 9_853737, 9_855553, 9_856957, 9_868533, 9_871487, 9_900335, 9_914340, 9_993913, 9_999242, 9_1005784, 9_1011926, 9_1021635, 9_1048196, 9_1048705, 9_1057346, 9_1073802, 9_1079046, 9_1098363, 9_1101149, 9_1115989, 9_1126264, 9_1126810, 9_1137233, 9_1149264, 9_1150366, 9_1171822, 9_1191440, 9_1198563, 9_1204843, 9_1229019, 9_1230422, 9_1239966, 9_1283319, 9_1292930, 9_1321595, 9_1335339, 9_1350862, 9_1356433, 9_1379525, 9_1381345, 9_1414239, 9_1423195, 9_1427688, 9_1429363, 9_1461217, 9_1477390, 9_1482655, 9_1484757, 9_1519090, 9_1521917, 9_1525659, 9_1529809, 9_1537823, 9_1562633, 9_1582163, 9_1595956, 9_1618990, 9_1629823, 9_1649194, 9_1674490, 9_1681979, 9_1687964, 9_1688677, 9_1767802, 9_1777790, 9_1784020, 9_1795938, 9_1801729, 9_1803394, 9_1874743, 9_1886833, 9_1899649, 9_1914852, 9_1924016, 9_1946020, 9_1962302, 9_2014519, 9_2016024, 9_2030283, 9_2031231, 9_2032139, 9_2032793, 9_2056241, 9_2137423, 9_2140383, 9_2152977, 9_2170780, 9_2197579, 9_2200939, 9_2220933, 9_2239976, 9_2257820, 9_2280211, 9_2309082, 9_2321623, 9_2326801, 9_2347267, 9_2354427, 9_2355156, 9_2384397, 9_2423879, 9_2443264, 9_2445077, 9_2483507, 9_2499774, 9_2533016, 9_2543668, 9_2568805, 9_2578442, 9_2583845, 9_2592544, 9_2598754, 9_2613819, 9_2618961, 9_2638671, 9_2641523, 9_2664896, 9_2685157, 9_2695764, 9_2696431, 9_2698412, 9_2702853, 9_2712030, 9_2725479, 9_2747665, 9_2755742, 9_2760671, 9_2764235, 9_2764450, 9_2771856, 9_2775594, 9_2802387, 9_2835744, 9_2838920, 9_2840079, 9_2844500, 9_2847012, 9_2852176, 9_2921639, 9_2929528, 9_2942415, 9_2948023, 9_2995859, 9_2998436, 9_3009727, 9_3032695, 9_3040322, 9_3098270, 9_3098631, 9_3120526, 9_3146913, 9_3179983, 9_3222295, 9_3224189, 9_3238731, 9_3241721, 9_3300957, 9_3301634, 9_3302201, 9_3318809, 9_3351615, 9_3439102, 9_3474446, 9_3490412, 9_3505555, 9_3543310, 9_3570156, 9_3570823, 9_3597381, 9_3597815, 9_3643626, 9_3651516, 9_3712023, 9_3744214, 9_3747433, 9_3819273, 9_3846341, 9_3897671, 9_3920266, 9_3948690, 9_3981735, 9_4021417, 9_4023671, 9_4029874, 9_4036094, 9_4082316, 9_4115079, 9_4129119, 9_4148519, 9_4203361, 9_4211562, 9_4248689, 9_4256130, 9_4257306, 9_4258699, 9_4259135, 9_4259449, 9_4273858, 9_4280211, 9_4295200, 9_4304583, 9_4313105, 9_4317203, 9_4329591, 9_4330933, 9_4478428, 9_4508804, 9_4509802, 9_4555010, 9_4566969, 9_4605649, 9_4678883, 9_4837306, 9_4837598, 9_4846614, 9_4866767, 9_4902248, 9_4910266, 9_4939068, 9_4967841, 9_4984091, 9_4995703, 9_5001780, 9_5019890, 9_5031113, 9_5156426, 9_5196364, 9_5205729, 9_5220378, 9_5332936, 9_5401225, 9_5494020, 9_5508450, 9_5514017, 9_5683078, 9_5684752, 9_5801948, 9_5834112, 9_5866746, 9_5940012, 9_5982540, 9_5993561, 9_6074771, 9_6142937, 9_6205776, 9_6318404, 9_6444010, 9_6457136, 9_6507673, 9_6529021, 9_6531972, 9_6534079, 9_6537428, 9_6546613, 9_6605900, 9_6616775, 9_6631414, 9_6645438, 9_6655143, 9_6679274, 9_6704917, 9_6810498, 9_6815389, 9_6819730, 9_6821254, 9_6828076, 9_6833952, 9_6866123, 9_6933747, 9_6951230, 9_6986464, 9_6986548, 9_7023001, 9_7046927, 9_7049668, 9_7069060, 9_7092453, 9_7108957, 9_7128489, 9_7130640, 9_7131274, 9_7158531, 9_7173439, 9_7202406, 9_7221273, 9_7238219, 9_7249472, 9_7249849, 9_7266435, 9_7282250, 9_7294940, 9_7500311, 9_7561629, 9_7682434, 9_7691562, 9_7712815, 9_7713779, 9_7718789, 9_7719084, 9_7728408, 9_7739386, 9_7744125, 9_7747870, 9_7765913, 9_7790927, 9_7792310, 9_7793895, 9_7798920, 9_7819548, 9_7821889, 9_7829791, 9_7842135, 9_7850245, 9_7887420, 9_7888223, 9_7894724, 9_7904279, 9_7911322, 9_7924719, 9_7927401, 9_7927804, 9_7928567, 9_7944909, 9_7956779, 9_7964323, 9_7976441, 9_7976547, 9_8048171, 9_8113393, 9_8149329, 9_8163777, 9_8228010, 9_8243781, 9_8262641, 9_8265854, 9_8325467, 9_8325713, 9_8349950, 9_8352243, 9_8368128, 9_8382640, 9_8394826, 9_8422193, 9_8436005, 9_8464080, 9_8519272, 9_8538133, 9_8607656, 9_8623708, 9_8639398, 9_8642018, 9_8643482, 9_8698255, 9_8721471, 9_8828038, 9_8840085, 9_8853059, 9_8864181, 9_8918037, 9_8947008, 9_9181374, 9_9235182, 9_9252306, 9_9265931, 9_9335998, 9_9341303, 9_9359096, 9_9360285, 9_9384950, 9_9408009, 9_9414345, 9_9442937, 9_9464882, 9_9467806, 9_9490893, 9_9492156, 9_9565431, 9_9601504, 9_9608313, 9_9610350, 9_9652687, 9_9659262, 9_9661256, 9_9671927, 9_9715747, 9_9733729, 9_9772165, 9_9772552, 9_9810856, 9_9818320, 9_9902798, 9_9961877, 9_10040944, 9_10064764, 9_10081164, 9_10085063, 9_10162091, 9_10203107, 9_10324581, 9_10357597, 9_10359323, 9_10398049, 9_10459607, 9_10572833, 9_10589168, 9_10590071, 9_10619147, 9_10642956, 9_10649053, 9_10651084, 9_10676616, 9_10722149, 9_10729403, 9_10785152, 9_10947898, 9_10965920, 9_10967682, 9_11090112, 9_11095690, 9_11298127, 9_11300084, 9_11335604, 9_11340120, 9_11341850, 9_11347724, 9_11351279, 9_11359342, 9_11378462, 9_11385380, 9_11401450, 9_11410145, 9_11412945, 9_11421763, 9_11458000, 9_11466460, 9_11468089, 9_11469956, 9_11478948, 9_11479400, 9_11480753, 9_11481639, 9_11548887, 9_11614106, 9_11623778, 9_11725058, 9_11752683, 9_11771671, 9_11781855, 9_11846514, 9_11846791, 9_11863183, 9_11882248, 9_11884826, 9_11913451, 9_11960188, 9_11966811, 9_12032271, 9_12091287, 9_12093162, 9_12116200, 9_12120110, 9_12195131, 9_12208167, 9_12296285, 9_12331203, 9_12333781, 9_12373418, 9_12384570, 9_12468809, 9_12469206, 9_12534447, 9_12668610, 9_12678618, 9_12678709, 9_12693892, 9_12706903, 9_12733254, 9_12738423, 9_12774059, 9_12789335, 9_12801783, 9_12822475, 9_12850507, 9_12853087, 9_12859201, 9_12886217, 9_13035672, 9_13041513, 9_13072321, 9_13100055, 9_13104017, 9_13181970, 9_13195553, 9_13197517, 9_13225880, 9_13271452, 9_13332669, 9_13346136, 9_13365511, 9_13387935, 9_13496647, 9_13518903, 9_13681447, 9_13709000, 9_13747900, 9_13818003, 9_13842755, 9_13849067, 9_13927945, 9_13966266, 9_13973237, 9_14012095, 9_14021844, 9_14028608, 9_14083012, 9_14141964, 9_14200788, 9_14245685, 9_14303008, 9_14341724, 9_14501373, 9_14551869, 9_14685699, 9_14734750, 9_14770604, 9_14784579, 9_14798710, 9_14824728, 9_14824981, 9_14836402, 9_14838323, 9_14899195, 9_15020596, 9_15073648, 9_15138715, 9_15280083, 9_15668294, 9_15671991, 9_15730735, 9_15733231, 9_15746454, 9_15826178, 9_15847565, 9_15855677, 9_15859020, 9_15860412, 9_15911331, 9_15941657, 9_15955805, 9_15972781, 9_15978698, 9_16003669, 9_16012680, 9_16028650, 9_16034063, 9_16043189, 9_16050588, 9_16078021, 9_16112373, 9_16153702, 9_16160849, 9_16200695, 9_16210283, 9_16243682, 9_16300703, 9_16456693, 9_16462599, 9_16476577, 9_16483990, 9_16497942, 9_16518877, 9_16628417, 9_16628788, 9_16677711, 9_16692816, 9_16829557, 9_16845580, 9_16949704, 9_16953533, 9_16956335, 9_16965108, 9_16967450, 9_17026639, 9_17097174, 9_17113355, 9_17133493, 9_17197641, 9_17229302, 9_17243334, 9_17253565, 9_17308522, 9_17309420, 9_17324366, 9_17324659, 9_17325107, 9_17330628, 9_17332353, 9_17334721, 9_17352289, 9_17364989, 9_17368735, 9_17600093, 9_17600651, 9_17641803, 9_17646088, 9_17784843, 9_17828130, 9_17836977, 9_17844218, 9_17852852, 9_17854487, 9_17861442, 9_17904269, 9_17904931, 9_17926645, 9_17933515, 9_17935250, 9_17998623, 9_18018332, 9_18026816, 9_18032390, 9_18048465, 9_18065187, 9_18080896, 9_18082131, 9_18170797, 9_18174867, 9_18247273, 9_18270434, 9_18334993, 9_18339360, 9_18340726, 9_18391348, 9_18403740, 9_18439441, 9_18474033, 9_18474342, 9_18517512, 9_18529008, 9_18532656, 9_18562188, 9_18565804, 9_18566290, 9_18567781, 9_18614367, 9_18636114, 9_18650764, 9_18660235, 9_18667686, 9_18724087, 9_18724983, 9_18725878, 9_18745645, 9_18746004, 9_18747615, 9_18816130, 9_18861529, 9_18864596, 9_18924101, 9_18967965, 9_18973357, 9_18985135, 9_18986928, 9_18991655, 9_19011409, 9_19017652, 9_19043596, 9_19075739, 9_19091097, 9_19091208, 9_19130526, 9_19153055, 9_19200509, 9_19211779, 9_19214993, 9_19218736, 9_19280598, 9_19296749, 9_19340405, 9_19366263, 9_19376547, 9_19439297, 9_19454606, 9_19484388, 9_19490089, 9_19497945, 9_19506897, 9_19521328, 9_19523349, 9_19524604, 9_19533021, 9_19544641, 9_19608805, 9_19642453, 9_19657726, 9_19658167, 9_19701280, 9_19704246, 9_19704560, 9_19841713, 9_19857939, 9_19864909, 9_19870821, 9_19886068, 9_19918811, 9_19939324, 9_19946994, 9_19963690, 9_19970824, 9_19985383, 9_19993093, 9_20049697, 9_20053923, 9_20059935, 9_20060388, 9_20061742, 9_20072032, 9_20086909, 9_20117693, 9_20119337, 9_20130860, 9_20133333, 9_20137694, 9_20180722, 9_20191727, 9_20271604, 9_20276663, 9_20283961, 9_20298237, 9_20304834, 9_20307949, 9_20309933, 9_20326511, 9_20331534, 9_20333698, 9_20334154, 9_20343795, 9_20388752, 9_20418859, 9_20432609, 9_20434459, 9_20434624, 9_20435214, 9_20458723, 9_20477276, 9_20505086, 9_20519643, 9_20535626, 9_20587783, 9_20602873, 9_20616243, 9_20619722, 9_20628835, 9_20635829, 9_20669161, 9_20681068, 9_20734346, 9_20744968, 9_20757062, 9_20779184, 9_20796390, 9_20809311, 9_20866583, 9_20882154, 9_20883232, 9_20883517, 9_20892040, 9_20928739, 9_20993671, 9_21044389, 9_21064005, 9_21066292, 9_21103856, 9_21121607, 9_21130223, 9_21147082, 9_21155712, 9_21172386, 9_21178791, 9_21181846, 9_21196326, 9_21229853, 9_21230848, 9_21259579, 9_21260224, 9_21325707, 9_21334773, 9_21367248, 9_21375784, 9_21383009, 9_21455420, 9_21486284, 9_21490536, 9_21492603, 9_21497366, 9_21508331, 9_21524848, 9_21537386, 9_21552214, 9_21558229, 9_21591435, 9_21596551, 9_21605249, 9_21623820, 9_21628203, 9_21648392, 9_21694062, 9_21699776, 9_21709149, 9_21746527, 9_21778507, 9_21802333, 9_21839661, 9_21870229, 9_21902956, 9_21909707, 9_21915421, 9_21935867, 9_21951163, 9_21951434, 9_21955087, 9_21963176, 9_21978538, 9_21992729, 9_22009474, 9_22011337, 9_22040882, 9_22042467, 9_22051146, 9_22087504, 9_22101145, 9_22134634, 9_22144483, 9_22150596, 9_22155515, 9_22156887, 9_22169078, 9_22172046, 9_22174141, 9_22183573, 9_22200898, 9_22210568, 9_22218089, 9_22229088, 9_22248256, 9_22268890, 9_22284447, 9_22299028, 9_22317468, 9_22318675, 9_22325558, 9_22374943, 9_22387588, 9_22392236, 9_22395426, 9_22403680, 9_22408806, 9_22415981, 9_22424670, 9_22426450, 9_22429741, 9_22432105, 9_22432360, 9_22486885, 9_22498791, 9_22500526, 9_22505616, 9_22539358, 9_22545668, 9_22545862, 9_22572257, 9_22620104, 9_22624953, 9_22626559, 9_22628095, 9_22629223, 9_22629464, 9_22636571, 9_22637324, 9_22642889, 9_22665101, 9_22668743, 9_22668932, 9_22676372, 9_22683624, 9_22701304, 9_22704333, 9_22710720, 9_22748204, 9_22785188, 9_22792086, 9_22792339, 9_22814147, 9_22830599, 9_22836981, 9_22839170, 9_22862323, 9_22884844, 9_22885106, 9_22909989, 9_22917101, 9_22928723, 9_22935595, 9_22937522, 9_22947782, 9_22947930, 9_22952248, 9_22988586, 9_22998152, 9_23011031, 9_23039222, 9_23055386, 9_23079040, 9_23091383, 9_23133533, 9_23152017, 9_23155873, 9_23174849, 9_23176122, 9_23196653, 9_23197131, 9_23205586, 9_23253153, 9_23262392, 9_23269756, 9_23283529, 9_23285673, 9_23325076, 9_23356915, 9_23374516, 9_23391422, 9_23396690, 9_23403229, 9_23410538, 9_23441597, 9_23473365, 9_23512746, 9_23517601, 9_23517853, 9_23529141, 9_23532490, 9_23534257, 9_23540969, 9_23546786, 9_23556306, 9_23557035, 9_23566045, 9_23588837, 9_23606556, 9_23607142, 9_23615580, 9_23631619, 9_23636803, 9_23679912, 9_23698895, 9_23751220, 9_23754671, 9_23756544, 9_23762140, 9_23802210, 9_23825342, 9_23873563, 9_23875350, 9_23878087, 9_23895249, 9_23962032, 9_23974362, 9_23974681, 9_23988332, 9_24002692, 9_24067953, 9_24092110, 9_24094463, 9_24099286, 9_24104748, 9_24118026, 9_24118544, 9_24120050, 9_24140661, 9_24144440, 9_24167290, 9_24202694, 9_24202864, 9_24203183, 9_24210707, 9_24254909, 9_24263397, 9_24270809, 9_24274900, 9_24298563, 9_24304116, 9_24320841, 9_24331276, 9_24332673, 9_24333062, 9_24343262, 9_24343712, 9_24344954, 9_24384902, 9_24398599, 9_24416900, 9_24426583, 9_24448752, 9_24465462, 9_24493694, 9_24533028, 9_24540677, 9_24571782, 9_24586495, 9_24589977, 9_24599509, 9_24613263, 9_24624180, 9_24678838, 9_24743324, 9_24743869, 9_24795211, 9_24822379, 9_24835220, 9_24841787, 9_24847508, 9_24847780, 9_24874677, 9_24890251, 9_24897018, 9_24915640, 9_24997556, 9_25015713, 9_25017125, 9_25033884, 9_25079176, 9_25093394, 9_25097786, 9_25098052, 9_25108190, 9_25120977, 9_25132481, 9_25160136, 9_25160668, 9_25160892, 9_25161579, 9_25161814, 9_25175814, 9_25215340, 9_25215539, 10_765, 10_83828, 10_86722, 10_86997, 10_98093, 10_107228, 10_255403, 10_307045, 10_309143, 10_310876, 10_334131, 10_379839, 10_380194, 10_393245, 10_404349, 10_453562, 10_453774, 10_455782, 10_509160, 10_549508, 10_684127, 10_766798, 10_768282, 10_786700, 10_790184, 10_826079, 10_849835, 10_865300, 10_871393, 10_888333, 10_911416, 10_932718, 10_939421, 10_974928, 10_981802, 10_1046679, 10_1077461, 10_1138217, 10_1143581, 10_1159174, 10_1280944, 10_1338555, 10_1352983, 10_1353694, 10_1353746, 10_1401593, 10_1401669, 10_1402936, 10_1418426, 10_1430127, 10_1439018, 10_1453449, 10_1482231, 10_1483075, 10_1486363, 10_1506430, 10_1530481, 10_1532585, 10_1540016, 10_1543516, 10_1548487, 10_1556139, 10_1585628, 10_1590177, 10_1604103, 10_1607075, 10_1661903, 10_1668828, 10_1684246, 10_1714080, 10_1715588, 10_1720455, 10_1728179, 10_1778317, 10_1804217, 10_1854465, 10_1865364, 10_1890557, 10_1908326, 10_1919454, 10_1939591, 10_1950069, 10_1952779, 10_1953253, 10_2002427, 10_2015603, 10_2017950, 10_2033770, 10_2063694, 10_2099481, 10_2107745, 10_2113643, 10_2124102, 10_2149557, 10_2165578, 10_2193040, 10_2200020, 10_2220222, 10_2262412, 10_2265550, 10_2266051, 10_2276555, 10_2298338, 10_2300367, 10_2323678, 10_2335557, 10_2336153, 10_2340593, 10_2344003, 10_2353184, 10_2355024, 10_2359413, 10_2370481, 10_2420762, 10_2452618, 10_2501714, 10_2506919, 10_2516663, 10_2548517, 10_2573634, 10_2589319, 10_2609207, 10_2624636, 10_2652773, 10_2679206, 10_2684617, 10_2692043, 10_2697122, 10_2721177, 10_2730267, 10_2738932, 10_2795799, 10_2796669, 10_2812486, 10_2816874, 10_2840631, 10_2855345, 10_2857518, 10_2871117, 10_2929949, 10_2940435, 10_2943536, 10_2945142, 10_2947138, 10_2954437, 10_2972011, 10_2978226, 10_2985849, 10_3074096, 10_3080617, 10_3096999, 10_3116820, 10_3146422, 10_3185187, 10_3187802, 10_3264728, 10_3297560, 10_3301948, 10_3321757, 10_3341823, 10_3357617, 10_3366974, 10_3379332, 10_3391012, 10_3396606, 10_3396711, 10_3403864, 10_3428332, 10_3510149, 10_3510752, 10_3531106, 10_3580865, 10_3765273, 10_3823882, 10_3845881, 10_3881425, 10_3937302, 10_3982145, 10_4001951, 10_4037552, 10_4114589, 10_4176828, 10_4210951, 10_4222453, 10_4237392, 10_4262935, 10_4289796, 10_4301009, 10_4332580, 10_4379479, 10_4538262, 10_4539130, 10_4583759, 10_4584212, 10_4592552, 10_4598970, 10_4599265, 10_4601388, 10_4637985, 10_4665319, 10_4686122, 10_4733831, 10_4749145, 10_4760544, 10_4763492, 10_4763737, 10_4767264, 10_4786781, 10_4817184, 10_4822531, 10_4899174, 10_4906262, 10_4915360, 10_4930230, 10_5062224, 10_5077499, 10_5182472, 10_5190924, 10_5223216, 10_5223455, 10_5230421, 10_5241708, 10_5290668, 10_5338552, 10_5396072, 10_5414132, 10_5415700, 10_5473781, 10_5503797, 10_5518924, 10_5561874, 10_5563912, 10_5567522, 10_5604148, 10_5605527, 10_5605495, 10_5637335, 10_5637482, 10_5669617, 10_5669657, 10_5698922, 10_5710229, 10_5734473, 10_5755806, 10_5757755, 10_5789465, 10_5802605, 10_5803060, 10_5826701, 10_5853879, 10_5869157, 10_5933514, 10_6001800, 10_6055622, 10_6090065, 10_6092974, 10_6227033, 10_6227374, 10_6243794, 10_6258493, 10_6271002, 10_6286710, 10_6358700, 10_6365034, 10_6467378, 10_6475960, 10_6499660, 10_6512565, 10_6522598, 10_6528350, 10_6532767, 10_6545489, 10_6633508, 10_6651723, 10_6680518, 10_6726570, 10_6821362, 10_6828866, 10_6843166, 10_6851864, 10_6856555, 10_6869506, 10_6894621, 10_6933580, 10_7125957, 10_7162602, 10_7167552, 10_7168095, 10_7168298, 10_7168975, 10_7176438, 10_7176651, 10_7204214, 10_7257945, 10_7279399, 10_7295253, 10_7313111, 10_7313870, 10_7319733, 10_7324717, 10_7336313, 10_7340571, 10_7389909, 10_7406463, 10_7457121, 10_7560576, 10_7578337, 10_7578766, 10_7586138, 10_7587935, 10_7590048, 10_7590760, 10_7603575, 10_7661171, 10_7668820, 10_7670481, 10_7673462, 10_7706289, 10_7716275, 10_7744309, 10_7746193, 10_7752436, 10_7757634, 10_7765632, 10_7767734, 10_7774237, 10_7781861, 10_7805630, 10_7807340, 10_7822622, 10_7836026, 10_7840565, 10_7876515, 10_7899752, 10_7913795, 10_7954307, 10_7954562, 10_7973638, 10_7987835, 10_8000358, 10_8041558, 10_8045501, 10_8054373, 10_8155183, 10_8159128, 10_8161871, 10_8162754, 10_8193098, 10_8197852, 10_8214217, 10_8223499, 10_8223974, 10_8250052, 10_8255546, 10_8267851, 10_8268918, 10_8289089, 10_8316279, 10_8351185, 10_8351878, 10_8363605, 10_8370398, 10_8372871, 10_8391776, 10_8412063, 10_8441616, 10_8452020, 10_8469310, 10_8485617, 10_8528264, 10_8532738, 10_8559517, 10_8567113, 10_8584828, 10_8596310, 10_8613740, 10_8646891, 10_8649273, 10_8698884, 10_8702655, 10_8716304, 10_8724168, 10_8763359, 10_8799946, 10_8833661, 10_8852919, 10_8906061, 10_8996948, 10_9006186, 10_9010381, 10_9028636, 10_9030482, 10_9049388, 10_9076495, 10_9113426, 10_9124888, 10_9139044, 10_9163613, 10_9182931, 10_9199711, 10_9236564, 10_9250255, 10_9276630, 10_9334719, 10_9347922, 10_9388070, 10_9407335, 10_9425826, 10_9445940, 10_9464251, 10_9483706, 10_9498164, 10_9510220, 10_9538043, 10_9543209, 10_9569017, 10_9569914, 10_9581058, 10_9581388, 10_9591490, 10_9664917, 10_9666539, 10_9714178, 10_9739936, 10_9743781, 10_9744033, 10_9785028, 10_9797220, 10_9825619, 10_9854484, 10_9856168, 10_9866395, 10_9880237, 10_9911875, 10_9917573, 10_9931145, 10_9931239, 10_9987543, 10_9988132, 10_10003721, 10_10006828, 10_10009726, 10_10016383, 10_10114434, 10_10126196, 10_10134948, 10_10146415, 10_10222430, 10_10227840, 10_10236438, 10_10251622, 10_10256289, 10_10261016, 10_10273635, 10_10284027, 10_10358156, 10_10364245, 10_10382197, 10_10395277, 10_10397337, 10_10397538, 10_10417963, 10_10418265, 10_10471183, 10_10503491, 10_10527531, 10_10557990, 10_10559636, 10_10567792, 10_10581470, 10_10616822, 10_10623304, 10_10634413, 10_10642979, 10_10672930, 10_10682641, 10_10693816, 10_10697424, 10_10698246, 10_10721802, 10_10725370, 10_10781428, 10_10799451, 10_10805118, 10_10824120, 10_10857255, 10_10860298, 10_10888724, 10_10922028, 10_10971128, 10_11021447, 10_11090320, 10_11090450, 10_11111056, 10_11126980, 10_11140152, 10_11140640, 10_11142736, 10_11191720, 10_11206830, 10_11248892, 10_11280538, 10_11327388, 10_11373415, 10_11411116, 10_11411239, 10_11427230, 10_11476533, 10_11516503, 10_11528398, 10_11564656, 10_11593765, 10_11595286, 10_11607072, 10_11608202, 10_11624609, 10_11633682, 10_11684906, 10_11726918, 10_11799131, 10_11824129, 10_11865729, 10_11875494, 10_11875772, 10_11893051, 10_11926533, 10_11950710, 10_11965435, 10_11974860, 10_12007327, 10_12015770, 10_12027472, 10_12028023, 10_12089110, 10_12103795, 10_12108097, 10_12109294, 10_12113889, 10_12148736, 10_12177805, 10_12202819, 10_12406153, 10_12415216, 10_12458287, 10_12498530, 10_12500818, 10_12514665, 10_12624501, 10_12653652, 10_12691391, 10_12696761, 10_12727028, 10_12740078, 10_12763006, 10_12792913, 10_12810176, 10_12822382, 10_12823234, 10_12834464, 10_12859085, 10_12890233, 10_12897294, 10_12921280, 10_12924610, 10_12940355, 10_12988424, 10_13031132, 10_13039852, 10_13040934, 10_13053793, 10_13139111, 10_13152988, 10_13201635, 10_13207754, 10_13298304, 10_13307002, 10_13428830, 10_13463509, 10_13503993, 10_13716429, 10_13749805, 10_13758961, 10_13821820, 10_13822622, 10_13822696, 10_13836061, 10_13851343, 10_13859264, 10_13889987, 10_13893075, 10_13923126, 10_13956909, 10_14007270, 10_14029171, 10_14035255, 10_14038749, 10_14050932, 10_14052630, 10_14086721, 10_14188110, 10_14205845, 10_14209740, 10_14220193, 10_14234244, 10_14268103, 10_14272348, 10_14273299, 10_14329332, 10_14398276, 10_14406327, 10_14422503, 10_14490812, 10_14496625, 10_14653263, 10_14677015, 10_14735592, 10_14737470, 10_14790359, 10_14816150, 10_14841413, 10_14882902, 10_14911426, 10_14983804, 10_15005603, 10_15019563, 10_15118259, 10_15129431, 10_15154571, 10_15205846, 10_15281046, 10_15294142, 10_15324682, 10_15401938, 10_15408467, 10_15460674, 10_15486878, 10_15511674, 10_15512018, 10_15512609, 10_15523171, 10_15527631, 10_15550327, 10_15578474, 10_15584653, 10_15629279, 10_15640876, 10_15662173, 10_15690118, 10_15690375, 10_15691669, 10_15697442, 10_15700608, 10_15702709, 10_15718382, 10_15745735, 10_15783292, 10_15790446, 10_15803824, 10_15804104, 10_15823375, 10_15860576, 10_15888783, 10_15936249, 10_16002127, 10_16008159, 10_16017477, 10_16025704, 10_16026142, 10_16029155, 10_16034096, 10_16034360, 10_16043831, 10_16045764, 10_16051248, 10_16068084, 10_16097787, 10_16104273, 10_16111665, 10_16119882, 10_16126641, 10_16159002, 10_16164319, 10_16175276, 10_16221645, 10_16255236, 10_16280394, 10_16335163, 10_16372828, 10_16397442, 10_16397770, 10_16463282, 10_16469951, 10_16470977, 10_16484953, 10_16554769, 10_16576995, 10_16610373, 10_16615501, 10_16687838, 10_16693742, 10_16715138, 10_16751697, 10_16781193, 10_16795195, 10_16829796, 10_16836864, 10_16845987, 10_17001722, 10_17033030, 10_17059365, 10_17151103, 10_17151219, 10_17188620, 10_17188868, 10_17225043, 10_17243924, 10_17265083, 10_17269511, 10_17270151, 10_17270552, 10_17294381, 10_17294512, 10_17305994, 10_17306099, 10_17316533, 10_17323899, 10_17348773, 10_17366734, 10_17373832, 10_17376107, 10_17410057, 10_17435390, 10_17453857, 10_17489302, 10_17549358, 10_17579394, 10_17589691, 10_17604823, 10_17630837, 10_17642486, 10_17648704, 10_17668158, 10_17690294, 10_17692829, 10_17700506, 10_17706340, 10_17710182, 10_17711529, 10_17715512, 10_17732005, 10_17751224, 10_17757308, 10_17759512, 10_17761927, 10_17764889, 10_17773674, 10_17785792, 10_17789215, 10_17816367, 10_17817052, 10_17823704, 10_17828686, 10_17835906, 10_17850858, 10_17854395, 10_17873962, 10_17909525, 10_17988545, 10_17996534, 10_18004176, 10_18026658, 10_18034025, 10_18055456, 10_18061907, 10_18083698, 10_18153379, 10_18156807, 10_18162337, 10_18196667, 10_18197292, 10_18208189, 10_18208470, 10_18238088, 10_18241673, 10_18270366, 10_18277079, 10_18283442, 10_18317230, 10_18340777, 10_18342376, 10_18351084, 10_18367790, 10_18406752, 10_18416464, 10_18427763, 10_18447748, 10_18456804, 10_18472439, 10_18478085, 10_18509272, 10_18515576, 10_18523417, 10_18532079, 10_18532533, 10_18581716, 10_18583550, 10_18586510, 10_18609365, 10_18621675, 10_18625190, 10_18627678, 10_18639743, 10_18700063, 10_18710802, 10_18739192, 10_18748947, 10_18749242, 10_18757360, 10_18777848, 10_18781208, 10_18788301, 10_18821903, 10_18824088, 10_18825674, 10_18856275, 10_18856551, 10_18857148, 10_18857978, 10_18859895, 10_18892965, 10_18932191, 10_18933028, 10_18944404, 10_18945221, 10_18972476, 10_18993241, 10_19021336, 10_19023205, 10_19024961, 10_19045111, 10_19073432, 10_19073849, 10_19090640, 10_19099878, 10_19112930, 10_19139464, 10_19155645, 10_19166955, 10_19183097, 10_19187834, 10_19188407, 10_19224149, 10_19257594, 10_19260484, 10_19262322, 10_19285889, 10_19304465, 10_19307990, 10_19322477, 10_19326478, 10_19332200, 10_19336760, 10_19338059, 10_19358376, 10_19373335, 10_19375240, 10_19379845, 10_19415567, 10_19442747, 10_19449132, 10_19452106, 10_19452430, 10_19480641, 10_19529100, 10_19531073, 10_19532406, 10_19557384, 10_19563237, 10_19563896, 10_19568910, 10_19594778, 10_19622486, 10_19638155, 10_19706405, 10_19706944, 10_19716662, 10_19730450, 10_19743298, 10_19750960, 10_19763343, 10_19785611, 10_19788917, 10_19797653, 10_19817902, 10_19912417, 10_19920792, 10_19928799, 10_19941051, 10_19941812, 10_19975129, 10_19978049, 10_20028158, 10_20077952, 10_20088228, 10_20090577, 10_20092975, 10_20108557, 10_20138548, 10_20150119, 10_20154026, 10_20169888, 10_20176802, 10_20184114, 10_20227487, 10_20230945, 10_20283137, 10_20295059, 10_20308634, 10_20330796, 10_20344492, 10_20348559, 10_20361370, 10_20367987, 10_20392517, 10_20393931, 10_20443529, 10_20505077, 10_20512841, 10_20576983, 10_20580487, 10_20658992, 10_20661081, 10_20661879, 10_20667300, 10_20696600, 10_20700964, 10_20703068, 10_20715148, 10_20788674, 10_20801462, 10_20819650, 10_20835848, 10_20852436, 10_20861304, 10_20868987, 10_20875304, 10_20878734, 10_20896721, 10_20983380, 10_20989378, 10_21000872, 10_21018680, 10_21036897, 10_21041444, 10_21063644, 10_21074456, 10_21075155, 10_21075943, 10_21077174, 10_21104878, 10_21132667, 10_21175514, 10_21189107, 10_21203981, 10_21205163, 10_21206277, 10_21309974, 10_21321539, 10_21332720, 10_21334561, 10_21386508, 10_21441998, 10_21468917, 10_21508851, 10_21529991, 10_21540650, 10_21558350, 10_21651681, 10_21659509, 10_21665893, 10_21668439, 10_21690318, 10_21701865, 10_21712283, 10_21746100, 10_21756478, 10_21783410, 10_21786019, 10_21791204, 10_21853622, 10_21854166, 10_21860091, 10_21861688, 10_21897363, 10_21905075, 10_21909609, 10_21915774, 10_21918438, 10_21943696, 10_22004085, 10_22037399, 10_22041754, 10_22044321, 10_22048487, 10_22081663, 10_22084569, 10_22086373, 10_22095430, 10_22098715, 10_22114734, 10_22131841, 10_22141838, 10_22148051, 10_22163089, 10_22169974, 10_22170313, 10_22176848, 10_22213148, 10_22214003, 10_22261868, 10_22274432, 10_22293367, 10_22296581, 10_22317577, 10_22338252, 10_22379596, 10_22380131, 10_22384804, 10_22384959, 10_22402543, 10_22413560, 10_22440659, 10_22458423, 10_22464569, 10_22486207, 10_22490806, 10_22513109, 10_22525434, 10_22553421, 10_22553712, 10_22559209, 10_22566358, 10_22592797, 10_22620349, 10_22620935, 10_22622865, 10_22627740, 10_22651855, 10_22660627, 10_22670339, 10_22681946, 10_22695732, 10_22702541, 10_22706488, 10_22718683, 10_22720650, 10_22725978, 10_22770378, 10_22776630, 10_22777431, 10_22802722, 10_22825959, 10_22826459, 10_22847369, 10_22885848, 10_22926096, 10_22930958, 10_22963899, 10_22994529, 10_23022329, 10_23032448, 10_23039573, 10_23056419, 10_23056742, 10_23057476, 10_23057738, 10_23065276, 10_23092900, 10_23096055, 10_23129954, 10_23165949, 10_23188969, 10_23192935, 10_23283515, 10_23313875, 10_23320882, 10_23341154, 10_23379867, 10_23397601, 10_23408922, 10_23429619, 10_23431078, 10_23443363, 10_23465208, 10_23466566, 10_23468876, 10_23474851, 10_23476243, 10_23483971, 10_23499127, 10_23508471, 10_23532622, 10_23544581, 10_23550011, 10_23596351, 10_23603012, 10_23616350, 10_23616624, 10_23627651, 10_23633536, 10_23639261, 10_23659063, 10_23660134, 10_23673926, 10_23713788, 10_23732365, 10_23743765, 10_23797374, 10_23806542, 10_23856593, 10_23902179, 10_23936254, 10_23945403, 10_23959332, 10_23973944, 10_23976195, 10_23978427, 10_23995664, 10_24009131, 10_24015086, 10_24015442, 10_24035261, 10_24053732, 10_24073515, 10_24095955, 10_24109801, 10_24119445, 10_24123827, 10_24138661, 10_24162686, 10_24207497, 10_24212076, 10_24214592, 10_24216468, 10_24223083, 10_24227656, 10_24230614, 10_24241277, 10_24261918, 10_24264517, 10_24266563, 10_24283445, 10_24297899, 10_24310755, 10_24328355, 10_24338541, 10_24342504, 10_24370552, 10_24370813, 10_24372068, 10_24383232, 10_24387875, 10_24420523, 10_24433258, 10_24442902, 10_24444607, 10_24464451, 10_24483230, 10_24491441, 10_24496588, 10_24521361, 10_24547172, 10_24566321, 10_24574359, 10_24581751, 10_24589852, 10_24591474, 10_24622639, 10_24689930, 10_24693921, 10_24720975, 10_24769308, 10_24777905, 10_24787202, 10_24798535, 10_24808870, 10_24823950, 10_24834267, 10_24835889, 10_24853087, 10_24855120, 10_24856651, 10_24897245, 10_24921780, 10_24921825, 10_24935800, 10_24951849, 10_24954227, 10_24962492, 10_24973419, 10_25020658, 10_25027885, 10_25051380, 10_25066626, 10_25070702, 10_25076147, 10_25076699, 10_25076957, 10_25102873, 10_25126978, 10_25129124, 10_25139075, 10_25140900, 10_25143044, 10_25150997, 10_25153047, 10_25158789, 10_25170899, 10_25184852, 10_25205994, 11_14766, 11_42129, 11_56095, 11_56422, 11_135071, 11_148565, 11_178948, 11_195246, 11_206477, 11_206969, 11_293410, 11_337549, 11_343955, 11_346214, 11_346803, 11_421446, 11_424098, 11_432344, 11_436527, 11_449465, 11_450029, 11_463152, 11_469033, 11_485408, 11_513115, 11_517453, 11_519561, 11_533833, 11_545629, 11_570032, 11_578269, 11_656422, 11_661728, 11_694706, 11_700275, 11_719175, 11_725557, 11_732754, 11_733001, 11_733648, 11_746915, 11_771236, 11_783125, 11_828948, 11_836332, 11_873868, 11_914931, 11_946145, 11_1007720, 11_1022850, 11_1040938, 11_1049600, 11_1109366, 11_1111882, 11_1120709, 11_1165409, 11_1186335, 11_1212447, 11_1250161, 11_1284744, 11_1330416, 11_1350278, 11_1368607, 11_1413313, 11_1448213, 11_1480064, 11_1481901, 11_1553475, 11_1574241, 11_1616966, 11_1806326, 11_1835292, 11_1838764, 11_1845021, 11_2000393, 11_2006037, 11_2057200, 11_2108855, 11_2149833, 11_2165071, 11_2181192, 11_2196158, 11_2231681, 11_2254025, 11_2292002, 11_2311889, 11_2317512, 11_2363404, 11_2371488, 11_2376515, 11_2421607, 11_2427771, 11_2500708, 11_2547025, 11_2554350, 11_2566246, 11_2615330, 11_2617428, 11_2631061, 11_2652944, 11_2663416, 11_2664671, 11_2681209, 11_2801566, 11_2812818, 11_2821610, 11_2824863, 11_2831716, 11_2861133, 11_2861906, 11_2889103, 11_2914924, 11_2954083, 11_2956296, 11_3007089, 11_3036769, 11_3037060, 11_3074797, 11_3116754, 11_3197802, 11_3231301, 11_3266322, 11_3300741, 11_3371726, 11_3373779, 11_3378423, 11_3394484, 11_3403857, 11_3444076, 11_3517135, 11_3523165, 11_3523920, 11_3547594, 11_3555978, 11_3562892, 11_3643250, 11_3648210, 11_3667464, 11_3705866, 11_3710300, 11_3716056, 11_3725768, 11_3816442, 11_3860489, 11_3870973, 11_3882420, 11_3884690, 11_3907927, 11_3910907, 11_3917426, 11_3941191, 11_3963050, 11_3969822, 11_3975747, 11_3975957, 11_4016390, 11_4018392, 11_4050657, 11_4082840, 11_4111233, 11_4119895, 11_4140399, 11_4142894, 11_4153177, 11_4170864, 11_4200736, 11_4214151, 11_4217185, 11_4235920, 11_4291835, 11_4299993, 11_4331041, 11_4338475, 11_4411077, 11_4422388, 11_4426253, 11_4514058, 11_4517445, 11_4522199, 11_4569950, 11_4574880, 11_4602702, 11_4625206, 11_4669409, 11_4670710, 11_4728498, 11_4731575, 11_4733971, 11_4771275, 11_4776826, 11_4794609, 11_4795029, 11_4837490, 11_4840811, 11_4847502, 11_4847811, 11_4856460, 11_4859972, 11_4882972, 11_4918643, 11_4955335, 11_4970243, 11_4976301, 11_5036733, 11_5039885, 11_5045250, 11_5053049, 11_5064930, 11_5066823, 11_5075479, 11_5090139, 11_5122922, 11_5124669, 11_5135992, 11_5137123, 11_5149116, 11_5151429, 11_5151610, 11_5182633, 11_5189518, 11_5212322, 11_5223324, 11_5236035, 11_5241816, 11_5247829, 11_5268105, 11_5280841, 11_5321187, 11_5328719, 11_5378328, 11_5384991, 11_5387974, 11_5421013, 11_5447607, 11_5449392, 11_5452979, 11_5457947, 11_5462010, 11_5471764, 11_5502236, 11_5515902, 11_5544436, 11_5578976, 11_5634275, 11_5647587, 11_5652903, 11_5677341, 11_5686701, 11_5688767, 11_5717358, 11_5724838, 11_5750544, 11_5768200, 11_5773040, 11_5803556, 11_5811892, 11_5834087, 11_5857736, 11_5858069, 11_5862297, 11_5865065, 11_5866631, 11_5912991, 11_5945342, 11_5957429, 11_5972323, 11_5974289, 11_5975252, 11_5993615, 11_5995842, 11_6001422, 11_6073228, 11_6109304, 11_6123881, 11_6124234, 11_6126940, 11_6138289, 11_6188883, 11_6199448, 11_6204189, 11_6214242, 11_6226598, 11_6227625, 11_6236045, 11_6279943, 11_6282412, 11_6297027, 11_6318461, 11_6320352, 11_6329543, 11_6334690, 11_6358730, 11_6359522, 11_6399853, 11_6402306, 11_6416772, 11_6417856, 11_6422339, 11_6482274, 11_6498250, 11_6498442, 11_6498587, 11_6521633, 11_6526204, 11_6537859, 11_6560545, 11_6564878, 11_6577590, 11_6586755, 11_6588725, 11_6589236, 11_6603254, 11_6623327, 11_6629154, 11_6649831, 11_6668615, 11_6668649, 11_6691286, 11_6696593, 11_6699197, 11_6703837, 11_6706177, 11_6706707, 11_6720230, 11_6724191, 11_6737920, 11_6745520, 11_6767868, 11_6776468, 11_6779013, 11_6785980, 11_6839808, 11_6853155, 11_6860832, 11_6861640, 11_6861991, 11_6876099, 11_6951325, 11_6953709, 11_6991677, 11_7020823, 11_7027402, 11_7029362, 11_7061923, 11_7094343, 11_7109950, 11_7115336, 11_7130917, 11_7148586, 11_7200044, 11_7218650, 11_7229348, 11_7253539, 11_7270292, 11_7283476, 11_7287219, 11_7313163, 11_7349018, 11_7358682, 11_7416613, 11_7440169, 11_7447626, 11_7462577, 11_7532342, 11_7546412, 11_7609421, 11_7609947, 11_7650880, 11_7651221, 11_7699292, 11_7726134, 11_7737976, 11_7791295, 11_7792690, 11_7794596, 11_7809813, 11_7820376, 11_7878246, 11_7880275, 11_7907381, 11_7913868, 11_7937154, 11_7974802, 11_8022432, 11_8125300, 11_8169285, 11_8230357, 11_8242123, 11_8254935, 11_8266766, 11_8296857, 11_8339602, 11_8341271, 11_8357623, 11_8362982, 11_8370210, 11_8382798, 11_8386922, 11_8456652, 11_8471857, 11_8491878, 11_8506072, 11_8528353, 11_8536687, 11_8542406, 11_8559527, 11_8561151, 11_8586765, 11_8596658, 11_8600932, 11_8605391, 11_8644810, 11_8663305, 11_8669056, 11_8683497, 11_8698360, 11_8702369, 11_8703639, 11_8773489, 11_8797721, 11_8824070, 11_8921931, 11_9004808, 11_9011571, 11_9105681, 11_9114935, 11_9122467, 11_9123097, 11_9125502, 11_9136601, 11_9138396, 11_9143191, 11_9158106, 11_9173243, 11_9175718, 11_9188170, 11_9230677, 11_9270129, 11_9297851, 11_9316874, 11_9319178, 11_9324311, 11_9365210, 11_9369387, 11_9370007, 11_9374462, 11_9379997, 11_9392121, 11_9392551, 11_9393008, 11_9477553, 11_9479349, 11_9497374, 11_9499527, 11_9568047, 11_9582861, 11_9625540, 11_9710692, 11_9725373, 11_9726357, 11_9736006, 11_9770189, 11_9781196, 11_9802345, 11_9806256, 11_9807773, 11_9834573, 11_9839050, 11_9845350, 11_9848555, 11_9907897, 11_9909232, 11_9910889, 11_9945394, 11_9979019, 11_10005897, 11_10006739, 11_10007598, 11_10044501, 11_10089307, 11_10122756, 11_10124383, 11_10174275, 11_10217977, 11_10218182, 11_10246622, 11_10249851, 11_10267293, 11_10291923, 11_10319531, 11_10330271, 11_10331015, 11_10364341, 11_10382872, 11_10452986, 11_10453982, 11_10472123, 11_10518008, 11_10545886, 11_10554479, 11_10555318, 11_10694612, 11_10745247, 11_10753195, 11_10765918, 11_10814362, 11_10845026, 11_10859159, 11_10861711, 11_10897695, 11_10945396, 11_10953556, 11_10958699, 11_10958739, 11_10992945, 11_11004212, 11_11004480, 11_11027757, 11_11040588, 11_11100531, 11_11103637, 11_11103996, 11_11148544, 11_11157994, 11_11168194, 11_11181346, 11_11182553, 11_11220862, 11_11240054, 11_11263682, 11_11274337, 11_11277690, 11_11287524, 11_11291102, 11_11291673, 11_11294284, 11_11296846, 11_11300118, 11_11300325, 11_11304269, 11_11309073, 11_11315121, 11_11329646, 11_11417999, 11_11421735, 11_11421875, 11_11434849, 11_11439882, 11_11471181, 11_11495159, 11_11548587, 11_11584365, 11_11589351, 11_11600375, 11_11628245, 11_11632501, 11_11733397, 11_11734896, 11_11768879, 11_11794921, 11_11828680, 11_11841647, 11_11942104, 11_11946299, 11_11947787, 11_11948691, 11_11992775, 11_12028350, 11_12041506, 11_12100282, 11_12133471, 11_12153827, 11_12162302, 11_12245769, 11_12254590, 11_12279017, 11_12301878, 11_12302444, 11_12358765, 11_12385068, 11_12390657, 11_12411726, 11_12435841, 11_12469799, 11_12568386, 11_12584351, 11_12589567, 11_12595134, 11_12651133, 11_12659494, 11_12701439, 11_12750226, 11_12847825, 11_12909899, 11_12935607, 11_12953634, 11_12959479, 11_13021181, 11_13069293, 11_13075089, 11_13078639, 11_13085731, 11_13090468, 11_13102750, 11_13104802, 11_13121874, 11_13154482, 11_13168955, 11_13182430, 11_13223429, 11_13235002, 11_13325644, 11_13390609, 11_13395634, 11_13444588, 11_13454337, 11_13460167, 11_13465421, 11_13509099, 11_13515363, 11_13523951, 11_13579952, 11_13597942, 11_13627999, 11_13669719, 11_13691307, 11_13733691, 11_13738072, 11_13764532, 11_13792183, 11_13812048, 11_13834765, 11_13885699, 11_13886178, 11_13932085, 11_13934173, 11_14032013, 11_14064396, 11_14085388, 11_14106428, 11_14127740, 11_14190674, 11_14206071, 11_14237095, 11_14254717, 11_14262991, 11_14280367, 11_14310557, 11_14352601, 11_14352920, 11_14370985, 11_14385561, 11_14390473, 11_14473351, 11_14509531, 11_14580914, 11_14620727, 11_14627332, 11_14644143, 11_14652104, 11_14663139, 11_14704345, 11_14789342, 11_14815713, 11_14820327, 11_14853641, 11_14869373, 11_14874706, 11_14887914, 11_14910049, 11_14921937, 11_15014353, 11_15015148, 11_15048434, 11_15061276, 11_15162828, 11_15167597, 11_15176341, 11_15249791, 11_15272616, 11_15319847, 11_15344082, 11_15344495, 11_15378836, 11_15409260, 11_15441829, 11_15526367, 11_15542848, 11_15562066, 11_15654681, 11_15687493, 11_15736024, 11_15796955, 11_15797366, 11_15817720, 11_15842146, 11_15905155, 11_15914709, 11_15947398, 11_16006562, 11_16032418, 11_16041866, 11_16045427, 11_16112591, 11_16112863, 11_16131998, 11_16141384, 11_16196571, 11_16221596, 11_16236134, 11_16250057, 11_16272037, 11_16279717, 11_16365345, 11_16379123, 11_16391166, 11_16450831, 11_16470055, 11_16470812, 11_16488325, 11_16549291, 11_16559289, 11_16577604, 11_16579488, 11_16591076, 11_16634044, 11_16634871, 11_16664593, 11_16671786, 11_16676427, 11_16676509, 11_16704183, 11_16735196, 11_16754550, 11_16792512, 11_16819033, 11_16820481, 11_16833590, 11_16837422, 11_16837990, 11_16877498, 11_16882063, 11_16889786, 11_16897060, 11_16926160, 11_16938624, 11_16970681, 11_16975595, 11_16993150, 11_16997128, 11_16997172, 11_17034088, 11_17084059, 11_17130466, 11_17151886, 11_17152048, 11_17155362, 11_17163882, 11_17223827, 11_17226602, 11_17252219, 11_17268168, 11_17273415, 11_17284434, 11_17295022, 11_17310588, 11_17316227, 11_17326856, 11_17348856, 11_17358768, 11_17440556, 11_17460881, 11_17485540, 11_17489240, 11_17500116, 11_17500228, 11_17522132, 11_17592238, 11_17598492, 11_17612943, 11_17694735, 11_17695227, 11_17700405, 11_17705095, 11_17718588, 11_17720623, 11_17737458, 11_17744916, 11_17772886, 11_17774579, 11_17801547, 11_17801707, 11_17804329, 11_17843823, 11_17847801, 11_17853182, 11_17861929, 11_17862888, 11_17866680, 11_17867086, 11_17917707, 11_17939471, 11_17949290, 11_17963138, 11_17971414, 11_17977179, 11_17983078, 11_17989001, 11_17996245, 11_18017873, 11_18056595, 11_18060768, 11_18071074, 11_18071390, 11_18077490, 11_18079501, 11_18084410, 11_18105787, 11_18112502, 11_18124036, 11_18149107, 11_18165572, 11_18168396, 11_18213783, 11_18238348, 11_18273549, 11_18275743, 11_18305488, 11_18341398, 11_18378828, 11_18411301, 11_18413343, 11_18477296, 11_18486228, 11_18519096, 11_18529250, 11_18546862, 11_18547306, 11_18607623, 11_18631504, 11_18633289, 11_18636026, 11_18642222, 11_18703861, 11_18767096, 11_18784889, 11_18794428, 11_18802163, 11_18836588, 11_18838176, 11_18863560, 11_18865520, 11_18891747, 11_18896180, 11_18902437, 11_18921673, 11_18925682, 11_18931160, 11_18952148, 11_18994388, 11_19017260, 11_19023627, 11_19060552, 11_19062772, 11_19069418, 11_19074315, 11_19075198, 11_19087223, 11_19090748, 11_19106959, 11_19111293, 11_19128344, 11_19148974, 11_19176685, 11_19178544, 11_19185999, 11_19188382, 11_19204630, 11_19208856, 11_19214103, 11_19230542, 11_19272156, 11_19330397, 11_19417729, 11_19431678, 11_19462367, 11_19488509, 11_19511643, 11_19512012, 11_19516682, 11_19526529, 11_19554580, 11_19590271, 11_19599229, 11_19599286, 11_19630282, 11_19654528, 11_19658389, 11_19659770, 11_19659942, 11_19715406, 11_19726662, 11_19743441, 11_19744596, 11_19757356, 11_19762350, 11_19773353, 11_19777791, 11_19791438, 11_19803886, 11_19810403, 11_19838254, 11_19844461, 11_19849685, 11_19872014, 11_19882710, 11_19898290, 11_19916112, 11_19944620, 11_19978000, 11_19979256, 11_19990478, 11_20012904, 11_20014203, 11_20014815, 11_20051335, 11_20054708, 11_20072676, 11_20086961, 11_20093048, 11_20093617, 11_20095005, 11_20098546, 11_20105647, 11_20109290, 11_20115269, 11_20116268, 11_20131273, 11_20132898, 11_20140033, 11_20143616, 11_20151038, 11_20155367, 11_20171864, 11_20188848, 11_20193693, 11_20208191, 11_20227518, 11_20316170, 11_20325625, 11_20330609, 11_20343881, 11_20357365, 11_20359863, 11_20370078, 11_20452066, 11_20511179, 11_20515450, 11_20518942, 11_20522118, 11_20531531, 11_20554583, 11_20554926, 11_20555817, 11_20564493, 11_20574924, 11_20578785, 11_20599096, 11_20602127, 11_20607875, 11_20618222, 11_20620386, 11_20628001, 11_20636943, 11_20692430, 11_20699135, 11_20702986, 11_20703677, 11_20754995, 11_20760275, 11_20773152, 11_20790976, 11_20799128, 11_20809789, 11_20833453, 11_20836611, 11_20858564, 11_20882451, 11_20901298, 11_20915121, 11_20948001, 11_20970098, 11_20975040, 11_20982598, 11_20990625, 11_21000029, 11_21001857, 11_21007803, 11_21009632, 11_21021158, 11_21025672, 11_21035112, 11_21060572, 11_21062629, 11_21077058, 11_21078093, 11_21101596, 11_21119546, 11_21119850, 11_21127399, 11_21155625, 11_21155857, 11_21157798, 11_21198396, 11_21201374, 11_21201925, 11_21211884, 11_21216166, 11_21232368, 11_21234968, 11_21235157, 11_21240944, 11_21244316, 11_21270867, 11_21272433, 11_21274214, 11_21274298, 11_21276573, 11_21280244, 11_21280404, 11_21291159, 11_21305608, 11_21311152, 11_21313812, 11_21344274, 11_21358911, 11_21363490, 11_21370878, 11_21380086, 11_21396325, 11_21398260, 11_21409625, 11_21419822, 11_21423602, 11_21439632, 11_21441689, 11_21445784, 11_21450347, 11_21465230, 11_21467126, 11_21479899, 11_21541913, 11_21554511, 11_21563891, 11_21567559, 11_21577399, 11_21578535, 11_21579617, 11_21642599, 11_21644786, 11_21647515, 11_21669421, 11_21680206, 11_21709203, 11_21712225, 11_21722600, 11_21728912, 11_21742142, 11_21761357, 11_21772023, 11_21785804, 11_21806664, 11_21820286, 11_21860562, 11_21862778, 11_21881357, 11_21893597, 11_21894033, 11_21907885, 11_21944667, 11_21946794, 11_21953992, 11_21972149, 11_21972488, 11_21987147, 11_22012913, 11_22016472, 11_22024657, 11_22024933, 11_22037886, 11_22050967, 11_22070464, 11_22077305, 11_22089265, 11_22093138, 11_22184319, 11_22194650, 11_22225608, 11_22238302, 11_22256158, 11_22264518, 11_22335255, 11_22338477, 11_22343965, 11_22350131, 11_22369008, 11_22422412, 11_22426459, 11_22480084, 11_22482632, 11_22503399, 11_22504176, 11_22506615, 11_22506908, 11_22534970, 11_22579356, 11_22580447, 11_22589186, 11_22591454, 11_22594274, 11_22600991, 11_22603069, 11_22615570, 11_22640256, 11_22656764, 11_22669470, 11_22672866, 11_22673172, 11_22731886, 11_22735233, 11_22736118, 11_22739988, 11_22744289, 11_22801410, 11_22803434, 11_22835474, 11_22859482, 11_22891801, 11_22903399, 11_22904768, 11_22905059, 11_22912716, 11_22928414, 11_22963753, 11_22966909, 11_22986443, 11_23015712, 11_23031961, 11_23044155, 11_23046865, 11_23066496, 11_23066868, 11_23072706, 11_23104644, 11_23114217, 11_23131449, 11_23138241, 11_23144875, 11_23188063, 11_23208315, 11_23212616, 11_23212798, 11_23225926, 11_23236400, 11_23237428, 11_23258309, 11_23306731, 11_23314155, 11_23320119, 11_23336752, 11_23336847, 11_23371505, 11_23392666, 11_23395168, 11_23425493, 11_23425736, 11_23427539, 11_23440644, 11_23452880, 11_23457530, 11_23460996, 11_23469574, 11_23483702, 11_23487932, 11_23564358, 11_23579736, 11_23581365, 11_23582298, 11_23584590, 11_23597594, 11_23600322, 11_23606940, 11_23630891, 11_23638127, 11_23650831, 11_23660609, 11_23669314, 11_23723282, 11_23726008, 11_23752102, 11_23767145, 11_23793958, 11_23820819, 11_23835437, 11_23836612, 11_23846869, 11_23852266, 11_23858394, 11_23863713, 11_23880150, 11_23884938, 11_23891418, 11_23893002, 11_23895653, 11_23905413, 11_23908420, 11_23916707, 11_23937247, 11_23965160, 11_23971229, 11_23973619, 11_23980968, 11_23995295, 11_23999571, 11_24118667, 11_24132312, 11_24172583, 11_24201439, 11_24201998, 11_24210555, 11_24216953, 11_24227520, 11_24239373, 11_24241457, 11_24269968, 11_24276727, 11_24277412, 11_24282795, 11_24283321, 11_24283606, 11_24284097, 11_24292667, 11_24295585, 11_24297241, 11_24308980, 11_24309674, 11_24320986, 11_24328377, 11_24329528, 11_24345390, 11_24373615, 11_24387530, 11_24393074, 11_24416786, 11_24417879, 11_24428938, 11_24430335, 11_24433836, 11_24435291, 11_24441641, 11_24444096, 11_24447276, 11_24448518, 11_24462947, 11_24466952, 11_24483691, 11_24483991, 11_24508432, 11_24519553, 11_24527240, 11_24542834, 11_24545434, 11_24548478, 11_24595098, 11_24603558, 11_24635925, 11_24647280, 11_24652660, 11_24679140, 11_24707683, 11_24711606, 11_24716899, 11_24717209, 11_24717747, 11_24740205, 11_24791168, 11_24792104, 11_24795743, 11_24797447, 11_24815926, 11_24818351, 11_24822877, 11_24884971, 11_24886106, 11_24895818, 11_24901156, 11_24914121, 11_24941585, 11_24959211, 11_24965631, 11_24974579, 11_24990217, 11_24990739, 11_25059267, 11_25062634, 11_25076782, 11_25087958, 12_27732, 12_59844, 12_107367, 12_114489, 12_116086, 12_119854, 12_119972, 12_164909, 12_165609, 12_166250, 12_203545, 12_207866, 12_215088, 12_215776, 12_218377, 12_226760, 12_235845, 12_236150, 12_261602, 12_263702, 12_272086, 12_277738, 12_294588, 12_328664, 12_333865, 12_343242, 12_343701, 12_353379, 12_360853, 12_361887, 12_389056, 12_391764, 12_425102, 12_465309, 12_466923, 12_473448, 12_553750, 12_554159, 12_554436, 12_607335, 12_608565, 12_608629, 12_635385, 12_643770, 12_660647, 12_663889, 12_673852, 12_673908, 12_678821, 12_680423, 12_696438, 12_724256, 12_775070, 12_777912, 12_816450, 12_819517, 12_854008, 12_854495, 12_855817, 12_856240, 12_856635, 12_876329, 12_887959, 12_897733, 12_916031, 12_963307, 12_973459, 12_973513, 12_977933, 12_1006015, 12_1010537, 12_1041358, 12_1046046, 12_1064115, 12_1075382, 12_1107485, 12_1145651, 12_1236806, 12_1253790, 12_1256174, 12_1256577, 12_1256645, 12_1288228, 12_1326359, 12_1334500, 12_1352636, 12_1363553, 12_1367774, 12_1369643, 12_1382092, 12_1412132, 12_1419691, 12_1421945, 12_1425871, 12_1426123, 12_1444173, 12_1449851, 12_1451138, 12_1451460, 12_1493496, 12_1498041, 12_1518392, 12_1548626, 12_1556701, 12_1557888, 12_1561770, 12_1583616, 12_1586346, 12_1593858, 12_1645668, 12_1647408, 12_1668343, 12_1681716, 12_1698258, 12_1698693, 12_1751354, 12_1751485, 12_1761642, 12_1763406, 12_1786654, 12_1818326, 12_1873000, 12_1876314, 12_1908479, 12_1949656, 12_1984688, 12_1995940, 12_2017821, 12_2031817, 12_2035334, 12_2040149, 12_2053309, 12_2056158, 12_2070465, 12_2084196, 12_2108236, 12_2127549, 12_2148290, 12_2156233, 12_2159857, 12_2164009, 12_2176101, 12_2177460, 12_2194381, 12_2283663, 12_2294376, 12_2309010, 12_2311916, 12_2328557, 12_2330297, 12_2398409, 12_2413896, 12_2422261, 12_2433984, 12_2438658, 12_2458784, 12_2467838, 12_2482374, 12_2501264, 12_2517345, 12_2558255, 12_2574148, 12_2576994, 12_2595886, 12_2613941, 12_2631143, 12_2641053, 12_2643752, 12_2793457, 12_2844906, 12_2848900, 12_2853653, 12_2858161, 12_2860788, 12_2881561, 12_2884520, 12_2884782, 12_2907835, 12_3000092, 12_3024534, 12_3090446, 12_3173019, 12_3227311, 12_3245729, 12_3256158, 12_3270102, 12_3291027, 12_3294456, 12_3308280, 12_3340690, 12_3351478, 12_3367700, 12_3383371, 12_3396164, 12_3444542, 12_3454361, 12_3481807, 12_3514028, 12_3543460, 12_3546171, 12_3550717, 12_3563393, 12_3564221, 12_3565729, 12_3577768, 12_3600521, 12_3618596, 12_3618923, 12_3619297, 12_3630072, 12_3663090, 12_3672756, 12_3685802, 12_3713908, 12_3719463, 12_3721180, 12_3724145, 12_3732961, 12_3745172, 12_3747634, 12_3797573, 12_3806647, 12_3839296, 12_3930099, 12_3932024, 12_4016954, 12_4036637, 12_4038888, 12_4039619, 12_4052405, 12_4057909, 12_4062763, 12_4096353, 12_4106957, 12_4116290, 12_4123852, 12_4143876, 12_4144384, 12_4151027, 12_4174452, 12_4177464, 12_4179657, 12_4191471, 12_4191673, 12_4203943, 12_4227159, 12_4227339, 12_4235994, 12_4249561, 12_4279425, 12_4288194, 12_4288771, 12_4291211, 12_4297963, 12_4351693, 12_4352528, 12_4380626, 12_4385585, 12_4387184, 12_4393047, 12_4395539, 12_4413968, 12_4428961, 12_4460442, 12_4466099, 12_4495489, 12_4503210, 12_4531782, 12_4535013, 12_4536585, 12_4551260, 12_4554061, 12_4558901, 12_4617152, 12_4629681, 12_4652185, 12_4669930, 12_4681503, 12_4730097, 12_4736525, 12_4736662, 12_4769937, 12_4778725, 12_4782603, 12_4793857, 12_4804889, 12_4805279, 12_4820886, 12_4830203, 12_4830559, 12_4833543, 12_4835806, 12_4871186, 12_4872345, 12_4912942, 12_4929053, 12_4933540, 12_4948725, 12_4952143, 12_4963270, 12_4977544, 12_4985215, 12_5002061, 12_5012298, 12_5017286, 12_5018479, 12_5022874, 12_5049292, 12_5049704, 12_5055733, 12_5059010, 12_5103062, 12_5110848, 12_5134027, 12_5137043, 12_5138895, 12_5171308, 12_5176645, 12_5186904, 12_5189568, 12_5233230, 12_5265219, 12_5265432, 12_5288440, 12_5298316, 12_5333051, 12_5343774, 12_5350149, 12_5365435, 12_5440432, 12_5463585, 12_5523350, 12_5567221, 12_5569285, 12_5587230, 12_5589029, 12_5596230, 12_5638088, 12_5638386, 12_5658953, 12_5666413, 12_5668900, 12_5692289, 12_5693252, 12_5697421, 12_5702966, 12_5754008, 12_5771183, 12_5780760, 12_5787676, 12_5791766, 12_5812775, 12_5822863, 12_5825767, 12_5836658, 12_5840004, 12_5860983, 12_5869830, 12_5889139, 12_5919604, 12_5924167, 12_5964066, 12_5976527, 12_6003853, 12_6004168, 12_6006324, 12_6024727, 12_6047094, 12_6087721, 12_6094392, 12_6157142, 12_6160928, 12_6161266, 12_6172528, 12_6176598, 12_6191481, 12_6245705, 12_6256291, 12_6269704, 12_6290873, 12_6313158, 12_6313618, 12_6318493, 12_6318899, 12_6328241, 12_6343447, 12_6354169, 12_6355527, 12_6399188, 12_6414261, 12_6432935, 12_6435109, 12_6436841, 12_6443632, 12_6454507, 12_6456123, 12_6490774, 12_6498084, 12_6528499, 12_6549073, 12_6567832, 12_6608201, 12_6609086, 12_6620605, 12_6622886, 12_6623192, 12_6624427, 12_6636136, 12_6647193, 12_6650615, 12_6676967, 12_6690262, 12_6698100, 12_6711373, 12_6714654, 12_6766459, 12_6784302, 12_6786598, 12_6811469, 12_6839251, 12_6904850, 12_6906221, 12_6956786, 12_6990046, 12_7007692, 12_7013864, 12_7034024, 12_7053380, 12_7054531, 12_7106443, 12_7108570, 12_7138687, 12_7154953, 12_7155431, 12_7160688, 12_7161732, 12_7180770, 12_7203637, 12_7208909, 12_7211037, 12_7219204, 12_7230489, 12_7232898, 12_7237510, 12_7242783, 12_7245800, 12_7253942, 12_7355087, 12_7371486, 12_7390309, 12_7394297, 12_7410338, 12_7415524, 12_7443120, 12_7444163, 12_7479815, 12_7494032, 12_7524390, 12_7616850, 12_7638325, 12_7688118, 12_7710129, 12_7748033, 12_7756941, 12_7787519, 12_7792172, 12_7801683, 12_7903409, 12_7915838, 12_7924456, 12_7949068, 12_7957731, 12_7980046, 12_8001590, 12_8004676, 12_8025086, 12_8027577, 12_8034405, 12_8058533, 12_8112546, 12_8142299, 12_8157343, 12_8175690, 12_8182205, 12_8210519, 12_8220878, 12_8225388, 12_8242512, 12_8300817, 12_8310650, 12_8367332, 12_8410608, 12_8450061, 12_8453388, 12_8468380, 12_8471104, 12_8538362, 12_8580542, 12_8614478, 12_8638412, 12_8650846, 12_8656914, 12_8674256, 12_8688977, 12_8715145, 12_8736973, 12_8741305, 12_8742938, 12_8743516, 12_8823094, 12_8869057, 12_8882273, 12_8882800, 12_8892790, 12_8900218, 12_8966357, 12_9030058, 12_9071739, 12_9145923, 12_9147179, 12_9196657, 12_9197254, 12_9202275, 12_9247710, 12_9250741, 12_9278832, 12_9340945, 12_9397846, 12_9446630, 12_9447503, 12_9452545, 12_9581596, 12_9613913, 12_9658477, 12_9787149, 12_9862645, 12_9914684, 12_9959084, 12_10001716, 12_10014850, 12_10021740, 12_10055165, 12_10068766, 12_10107280, 12_10117265, 12_10158628, 12_10160116, 12_10198408, 12_10242327, 12_10295239, 12_10344769, 12_10435477, 12_10452904, 12_10532284, 12_10554732, 12_10612411, 12_10615488, 12_10662178, 12_10686275, 12_10690946, 12_10697757, 12_10704448, 12_10753261, 12_10769117, 12_10769529, 12_10831462, 12_10847220, 12_10885680, 12_10904379, 12_10908277, 12_10916329, 12_10949568, 12_11032056, 12_11045785, 12_11061195, 12_11061465, 12_11140904, 12_11160025, 12_11163284, 12_11253416, 12_11269506, 12_11270005, 12_11270891, 12_11283614, 12_11284893, 12_11288644, 12_11433435, 12_11461093, 12_11470591, 12_11528123, 12_11570724, 12_11593301, 12_11611570, 12_11613384, 12_11658897, 12_11659584, 12_11668479, 12_11733989, 12_11748129, 12_11807300, 12_11809919, 12_11914080, 12_11921393, 12_11921524, 12_11927470, 12_11932704, 12_11943085, 12_11956546, 12_11961718, 12_11996592, 12_12010627, 12_12016904, 12_12057413, 12_12073247, 12_12094529, 12_12100197, 12_12161002, 12_12162376, 12_12162716, 12_12168849, 12_12169116, 12_12179575, 12_12182016, 12_12203469, 12_12204191, 12_12217691, 12_12245250, 12_12259592, 12_12259753, 12_12260019, 12_12280471, 12_12291957, 12_12298529, 12_12307339, 12_12335072, 12_12364189, 12_12378798, 12_12404096, 12_12438022, 12_12456968, 12_12484641, 12_12513207, 12_12528247, 12_12544883, 12_12568018, 12_12581433, 12_12626988, 12_12633832, 12_12646326, 12_12705350, 12_12714141, 12_12763162, 12_12795767, 12_12832258, 12_12833308, 12_12837164, 12_12873309, 12_12993652, 12_12995913, 12_13000994, 12_13002939, 12_13006264, 12_13008404, 12_13012165, 12_13015907, 12_13046053, 12_13050441, 12_13199767, 12_13203075, 12_13205987, 12_13257493, 12_13283049, 12_13305417, 12_13376966, 12_13397271, 12_13426623, 12_13457593, 12_13460123, 12_13497995, 12_13522954, 12_13524522, 12_13607597, 12_13614870, 12_13621955, 12_13687256, 12_13697100, 12_13700857, 12_13740923, 12_13807679, 12_13815080, 12_13835070, 12_13843626, 12_13861135, 12_13863583, 12_13864753, 12_13866686, 12_13874371, 12_13874843, 12_13881387, 12_13887157, 12_13887580, 12_13887649, 12_13887938, 12_13911172, 12_13920492, 12_13947169, 12_13947489, 12_13952845, 12_14019965, 12_14026340, 12_14054227, 12_14114792, 12_14119541, 12_14120356, 12_14134601, 12_14137663, 12_14146222, 12_14175189, 12_14207315, 12_14219298, 12_14231523, 12_14258183, 12_14273609, 12_14328154, 12_14333261, 12_14354750, 12_14355061, 12_14356980, 12_14380095, 12_14413503, 12_14482956, 12_14576737, 12_14578752, 12_14597183, 12_14619286, 12_14747912, 12_14771629, 12_14782373, 12_14820945, 12_14878727, 12_14889196, 12_14903727, 12_14942764, 12_14995227, 12_15035946, 12_15039438, 12_15041639, 12_15047186, 12_15073738, 12_15085725, 12_15096310, 12_15101234, 12_15157555, 12_15194634, 12_15202715, 12_15203401, 12_15204998, 12_15210750, 12_15232032, 12_15234757, 12_15271002, 12_15305741, 12_15325997, 12_15345576, 12_15374238, 12_15417692, 12_15432135, 12_15445143, 12_15458405, 12_15466882, 12_15544147, 12_15549766, 12_15559332, 12_15564635, 12_15584282, 12_15587669, 12_15610852, 12_15611961, 12_15682720, 12_15702237, 12_15736279, 12_15796849, 12_15847580, 12_15887104, 12_15887297, 12_15888102, 12_15914178, 12_16000560, 12_16045009, 12_16105325, 12_16119040, 12_16175077, 12_16186279, 12_16252401, 12_16309944, 12_16320545, 12_16326806, 12_16343552, 12_16356512, 12_16386194, 12_16386643, 12_16437927, 12_16439662, 12_16452080, 12_16458081, 12_16468244, 12_16468486, 12_16514835, 12_16523843, 12_16524929, 12_16583382, 12_16593883, 12_16611727, 12_16632856, 12_16645955, 12_16650835, 12_16695713, 12_16713023, 12_16718639, 12_16749482, 12_16808059, 12_16835074, 12_16843658, 12_16846951, 12_16847485, 12_16849785, 12_16927689, 12_16940600, 12_16996958, 12_17050027, 12_17082279, 12_17088902, 12_17107891, 12_17108032, 12_17120384, 12_17121068, 12_17125209, 12_17158615, 12_17177092, 12_17241120, 12_17248139, 12_17264849, 12_17267152, 12_17267364, 12_17277923, 12_17290485, 12_17290869, 12_17298205, 12_17339748, 12_17345921, 12_17355775, 12_17395722, 12_17432113, 12_17432918, 12_17440938, 12_17441990, 12_17443851, 12_17462408, 12_17469626, 12_17472545, 12_17484484, 12_17488929, 12_17506033, 12_17531528, 12_17569373, 12_17581338, 12_17593176, 12_17622909, 12_17647286, 12_17660298, 12_17697353, 12_17698584, 12_17701398, 12_17707596, 12_17710034, 12_17716658, 12_17723643, 12_17725939, 12_17733475, 12_17743893, 12_17762316, 12_17777021, 12_17806003, 12_17811661, 12_17832525, 12_17841267, 12_17850694, 12_17890395, 12_17914779, 12_17989425, 12_18062344, 12_18128397, 12_18130163, 12_18140251, 12_18141099, 12_18151262, 12_18184485, 12_18190890, 12_18207381, 12_18214143, 12_18290239, 12_18353277, 12_18353471, 12_18353775, 12_18364138, 12_18395439, 12_18426470, 12_18434522, 12_18438999, 12_18439419, 12_18460823, 12_18470868, 12_18471014, 12_18471976, 12_18510278, 12_18526628, 12_18563430, 12_18578714, 12_18589474, 12_18659269, 12_18717022, 12_18724858, 12_18729734, 12_18757283, 12_18763576, 12_18821353, 12_18824062, 12_18834130, 12_18879217, 12_18888252, 12_18897890, 12_18904801, 12_18906025, 12_18933191, 12_18968038, 12_18969967, 12_18990015, 12_19002112, 12_19007255, 12_19018641, 12_19057973, 12_19088368, 12_19122143, 12_19162904, 12_19238174, 12_19482553, 12_19489106, 12_19492031, 12_19503215, 12_19541135, 12_19572565, 12_19593661, 12_19616751, 12_19623050, 12_19661952, 12_19669878, 12_19697695, 12_19709029, 12_19758343, 12_19769768, 12_19804107, 12_19812005, 12_19818562, 12_19820391, 12_19824216, 12_19851457, 12_19868757, 12_19885737, 12_19924463, 12_19941774, 12_19943811, 12_19984756, 12_19993620, 12_20017050, 12_20020081, 12_20038906, 12_20039353, 12_20068279, 12_20094500, 12_20107891, 12_20114236, 12_20144119, 12_20145727, 12_20153305, 12_20228619, 12_20230513, 12_20234879, 12_20246498, 12_20269284, 12_20274673, 12_20283053, 12_20317243, 12_20325079, 12_20328516, 12_20338116, 12_20343665, 12_20369175, 12_20379758, 12_20385732, 12_20394031, 12_20395552, 12_20426236, 12_20427463, 12_20452798, 12_20461528, 12_20461843, 12_20559612, 12_20569154, 12_20574048, 12_20595357, 12_20596791, 12_20625238, 12_20642477, 12_20701239, 12_20702945, 12_20706088, 12_20742284, 12_20812035, 12_20816708, 12_20831013, 12_20832271, 12_20840021, 12_20846378, 12_20881368, 12_20884906, 12_20898632, 12_20917571, 12_20917964, 12_20956277, 12_20975795, 12_20979875, 12_20999422, 12_21079269, 12_21080702, 12_21087284, 12_21107185, 12_21124698, 12_21126204, 12_21128756, 12_21135602, 12_21181377, 12_21184348, 12_21184989, 12_21189929, 12_21222243, 12_21224413, 12_21228551, 12_21261790, 12_21271685, 12_21374321, 12_21374436, 12_21403586, 12_21420440, 12_21420703, 12_21453377, 12_21466019, 12_21483094, 12_21483223, 12_21518348, 12_21518920, 12_21521977, 12_21596840, 12_21694010, 12_21697207, 12_21731421, 12_21786362, 12_21791328, 12_21815779, 12_21838941, 12_21847810, 12_21858221, 12_21909151, 12_21909425, 12_21922400, 12_21953199, 12_21960347, 12_21978554, 12_21989649, 12_21990124, 12_21998458, 12_22011193, 12_22027279, 12_22031959, 12_22034435, 12_22077489, 12_22131416, 12_22134424, 12_22140749, 12_22151632, 12_22169267, 12_22200935, 12_22214244, 12_22215782, 12_22234748, 12_22248177, 12_22261713, 12_22270161, 12_22271115, 12_22287059, 12_22308635, 12_22349877, 12_22352682, 12_22355823, 12_22386496, 12_22397269, 12_22417509, 12_22459023, 12_22464211, 12_22469821, 12_22494982, 12_22533430, 12_22563773, 12_22579037, 12_22588481, 12_22591146, 12_22618126, 12_22628748, 12_22629297, 12_22695631, 12_22698622, 12_22723151, 12_22783564, 12_22843968, 12_22850779, 12_22863944, 12_22886523, 12_22907903, 12_22921862, 12_22928834, 12_22954389, 12_23019800, 12_23047602, 12_23061802, 12_23125166, 12_23134598, 12_23195384, 12_23203264, 12_23215320, 12_23249843, 12_23254324, 12_23320073, 12_23334127, 12_23356100, 12_23367873, 12_23401815, 12_23433822, 12_23494766, 12_23529181, 12_23542750, 12_23566781, 12_23572877, 12_23608552, 12_23648267, 12_23652685, 12_23667023, 12_23720533, 12_23783531, 12_23786580, 12_23805086, 12_23854704, 12_23863186, 12_23863788, 12_23868072, 12_24153487, 12_24196962, 12_24224916, 12_24257245, 12_24277040, 12_24283248, 12_24292952, 12_24311996, 12_24352055, 12_24462264, 12_24568479, 12_24571388, 12_24586656, 12_24591551, 12_24602187, 12_24620546, 12_24689145, 12_24707530, 12_24720463, 12_24729786, 12_24750867, 12_24760038, 13_5290, 13_60535, 13_148091, 13_153619, 13_183771, 13_186273, 13_204594, 13_216397, 13_222242, 13_251272, 13_252422, 13_271744, 13_271886, 13_275617, 13_283253, 13_286790, 13_287023, 13_288730, 13_314211, 13_322812, 13_323542, 13_352857, 13_365980, 13_369516, 13_391054, 13_391441, 13_396981, 13_428883, 13_463422, 13_465845, 13_470279, 13_476294, 13_478991, 13_479544, 13_507018, 13_539428, 13_550153, 13_553991, 13_554511, 13_576973, 13_578278, 13_584842, 13_598543, 13_601885, 13_603964, 13_616998, 13_617760, 13_637939, 13_640023, 13_641493, 13_669472, 13_685426, 13_688925, 13_703146, 13_704068, 13_705267, 13_723499, 13_735827, 13_751362, 13_753670, 13_756170, 13_777936, 13_806407, 13_820236, 13_864474, 13_869348, 13_875392, 13_880582, 13_881047, 13_923808, 13_931320, 13_957466, 13_957773, 13_962718, 13_963239, 13_972866, 13_987219, 13_1011391, 13_1037675, 13_1053988, 13_1101142, 13_1104821, 13_1117395, 13_1181409, 13_1189663, 13_1200618, 13_1203614, 13_1253096, 13_1266655, 13_1283340, 13_1284282, 13_1295654, 13_1312821, 13_1331993, 13_1332085, 13_1335766, 13_1339243, 13_1360314, 13_1382922, 13_1392258, 13_1411063, 13_1414777, 13_1452368, 13_1466110, 13_1466848, 13_1476248, 13_1477622, 13_1484698, 13_1485158, 13_1487360, 13_1502085, 13_1510742, 13_1515434, 13_1524846, 13_1533524, 13_1535633, 13_1544490, 13_1561627, 13_1571606, 13_1582403, 13_1585197, 13_1588236, 13_1623751, 13_1653394, 13_1664227, 13_1673315, 13_1680185, 13_1697616, 13_1720179, 13_1729813, 13_1769973, 13_1770308, 13_1784509, 13_1823374, 13_1824039, 13_1835641, 13_1926433, 13_1928050, 13_1928106, 13_1981392, 13_1989613, 13_1995357, 13_1995612, 13_2003946, 13_2006367, 13_2007784, 13_2008681, 13_2018482, 13_2029153, 13_2034606, 13_2041626, 13_2056352, 13_2060815, 13_2065269, 13_2065534, 13_2074489, 13_2076574, 13_2077601, 13_2077841, 13_2085616, 13_2093165, 13_2115011, 13_2131741, 13_2133107, 13_2139258, 13_2155709, 13_2178793, 13_2188038, 13_2190167, 13_2191123, 13_2193518, 13_2194353, 13_2201688, 13_2201899, 13_2203744, 13_2220869, 13_2221594, 13_2232000, 13_2232238, 13_2237642, 13_2247878, 13_2254480, 13_2259319, 13_2260848, 13_2268913, 13_2274438, 13_2285340, 13_2288857, 13_2290834, 13_2308111, 13_2352174, 13_2365921, 13_2380585, 13_2381062, 13_2422243, 13_2436560, 13_2441781, 13_2444644, 13_2460327, 13_2471693, 13_2477952, 13_2485700, 13_2495463, 13_2498850, 13_2550224, 13_2577154, 13_2607384, 13_2642864, 13_2651599, 13_2654391, 13_2673986, 13_2687435, 13_2693146, 13_2740192, 13_2756008, 13_2756314, 13_2759269, 13_2759890, 13_2781243, 13_2791859, 13_2804277, 13_2807194, 13_2816159, 13_2840711, 13_2841591, 13_2873761, 13_2956438, 13_2956680, 13_2971588, 13_2980176, 13_2986993, 13_3016871, 13_3032769, 13_3040191, 13_3064819, 13_3065508, 13_3068976, 13_3069889, 13_3071436, 13_3079377, 13_3081988, 13_3121678, 13_3121775, 13_3155143, 13_3158359, 13_3161747, 13_3195489, 13_3242976, 13_3245534, 13_3248767, 13_3255624, 13_3302367, 13_3307164, 13_3308015, 13_3406216, 13_3432212, 13_3439296, 13_3455962, 13_3491651, 13_3500661, 13_3524572, 13_3531285, 13_3547206, 13_3557665, 13_3641878, 13_3665867, 13_3715643, 13_3716429, 13_3721056, 13_3773113, 13_3774026, 13_3815830, 13_3858906, 13_3889871, 13_3919794, 13_3927814, 13_3931217, 13_3940757, 13_3941503, 13_3951068, 13_3967927, 13_4015714, 13_4021867, 13_4047629, 13_4066369, 13_4077837, 13_4121676, 13_4134558, 13_4137931, 13_4142885, 13_4152467, 13_4198767, 13_4247352, 13_4255390, 13_4312654, 13_4312845, 13_4323634, 13_4338688, 13_4359491, 13_4371150, 13_4388839, 13_4401642, 13_4402191, 13_4411136, 13_4452549, 13_4465996, 13_4506253, 13_4521416, 13_4536427, 13_4543675, 13_4555797, 13_4556013, 13_4560905, 13_4563678, 13_4592805, 13_4601526, 13_4628509, 13_4648351, 13_4653330, 13_4654425, 13_4664484, 13_4672232, 13_4677679, 13_4684973, 13_4707794, 13_4739092, 13_4743810, 13_4747061, 13_4753030, 13_4784341, 13_4808778, 13_4870995, 13_4881865, 13_4892554, 13_4909829, 13_4923704, 13_4942763, 13_4943701, 13_5000141, 13_5007400, 13_5023361, 13_5095632, 13_5128853, 13_5136204, 13_5149037, 13_5150694, 13_5188125, 13_5194979, 13_5195363, 13_5196572, 13_5204720, 13_5220025, 13_5307213, 13_5315563, 13_5367226, 13_5391284, 13_5410084, 13_5416304, 13_5441753, 13_5445175, 13_5445328, 13_5475358, 13_5478045, 13_5485025, 13_5496657, 13_5543825, 13_5558589, 13_5811862, 13_5870325, 13_5889267, 13_5946677, 13_5948225, 13_5954716, 13_5988543, 13_6019544, 13_6047502, 13_6121423, 13_6213613, 13_6258908, 13_6273368, 13_6290146, 13_6309812, 13_6315911, 13_6326010, 13_6327742, 13_6349379, 13_6432518, 13_6569243, 13_6595454, 13_6659223, 13_6695497, 13_6713265, 13_6727355, 13_6760623, 13_6783542, 13_6786969, 13_6793402, 13_6831319, 13_6833051, 13_6860547, 13_6873683, 13_6876733, 13_6896911, 13_7048180, 13_7061710, 13_7064078, 13_7073396, 13_7094009, 13_7095944, 13_7104103, 13_7129469, 13_7182787, 13_7293013, 13_7297812, 13_7298066, 13_7324924, 13_7333624, 13_7337945, 13_7353206, 13_7377745, 13_7396127, 13_7494481, 13_7518062, 13_7525728, 13_7532882, 13_7609033, 13_7676419, 13_7705307, 13_7724215, 13_7799284, 13_7887658, 13_7889369, 13_7945186, 13_8037937, 13_8048465, 13_8066158, 13_8409682, 13_8410217, 13_8412940, 13_8445844, 13_8471921, 13_8489666, 13_8505377, 13_8510443, 13_8544136, 13_8553049, 13_8558660, 13_8583877, 13_8630284, 13_8683198, 13_8697264, 13_8702438, 13_8765104, 13_8799777, 13_8818772, 13_8828873, 13_8847895, 13_8865051, 13_8897514, 13_8909000, 13_8946276, 13_8987605, 13_9035916, 13_9045093, 13_9105419, 13_9106238, 13_9112016, 13_9143232, 13_9150624, 13_9152464, 13_9244381, 13_9269360, 13_9283135, 13_9285625, 13_9349609, 13_9365158, 13_9383595, 13_9395774, 13_9407857, 13_9440520, 13_9469891, 13_9492663, 13_9518244, 13_9525537, 13_9525640, 13_9568023, 13_9595911, 13_9620304, 13_9634735, 13_9635087, 13_9675162, 13_9756225, 13_9767138, 13_9769197, 13_9814731, 13_9872078, 13_9872330, 13_9907756, 13_9928040, 13_9951902, 13_9964770, 13_10000063, 13_10003841, 13_10030651, 13_10051241, 13_10066081, 13_10124423, 13_10166310, 13_10185024, 13_10213971, 13_10215648, 13_10315123, 13_10319256, 13_10366325, 13_10380765, 13_10382404, 13_10382901, 13_10385516, 13_10408853, 13_10452974, 13_10464345, 13_10528836, 13_10559050, 13_10663306, 13_10701429, 13_10711662, 13_10713975, 13_10739535, 13_10761204, 13_10813821, 13_11081133, 13_11121975, 13_11195193, 13_11230087, 13_11285456, 13_11285828, 13_11307548, 13_11336808, 13_11404716, 13_11408816, 13_11409461, 13_11512107, 13_11516597, 13_11681469, 13_11684072, 13_11704163, 13_11717140, 13_11723765, 13_11743821, 13_11745514, 13_11755207, 13_11765539, 13_11773971, 13_11829068, 13_11830282, 13_11883648, 13_11885682, 13_11887065, 13_11888107, 13_11911138, 13_11948785, 13_11966767, 13_11973710, 13_11994982, 13_11995641, 13_12023714, 13_12051067, 13_12088314, 13_12126268, 13_12153805, 13_12172312, 13_12180621, 13_12338270, 13_12341470, 13_12356133, 13_12633064, 13_12643139, 13_12648756, 13_12726701, 13_12729331, 13_12806748, 13_12811039, 13_12821827, 13_12833531, 13_12837721, 13_12841063, 13_12851240, 13_12852999, 13_12915548, 13_12922471, 13_12971176, 13_12985252, 13_13012015, 13_13029365, 13_13031361, 13_13055185, 13_13062601, 13_13065749, 13_13112651, 13_13133985, 13_13176574, 13_13285498, 13_13339037, 13_13356343, 13_13402545, 13_13404057, 13_13447171, 13_13448242, 13_13465596, 13_13498120, 13_13524991, 13_13527734, 13_13550195, 13_13550555, 13_13556879, 13_13626219, 13_13690363, 13_13725989, 13_13744864, 13_13747019, 13_13767709, 13_13819210, 13_13842257, 13_13869143, 13_13871898, 13_13874420, 13_13953200, 13_13955620, 13_13980513, 13_13986050, 13_14009786, 13_14017311, 13_14105337, 13_14105831, 13_14117454, 13_14122640, 13_14142988, 13_14174681, 13_14202837, 13_14205807, 13_14228362, 13_14240785, 13_14245813, 13_14251974, 13_14268984, 13_14278653, 13_14282794, 13_14283055, 13_14321237, 13_14351495, 13_14410782, 13_14449528, 13_14494181, 13_14507968, 13_14575831, 13_14589643, 13_14646232, 13_14673467, 13_14695952, 13_14700160, 13_14719859, 13_14721640, 13_14735694, 13_14740503, 13_14749352, 13_14749445, 13_14754024, 13_14758072, 13_14779446, 13_14793106, 13_14853499, 13_14864965, 13_14887739, 13_14888965, 13_14927946, 13_14959060, 13_14982128, 13_14984589, 13_15008208, 13_15017417, 13_15019497, 13_15028836, 13_15032793, 13_15056979, 13_15160095, 13_15160350, 13_15182284, 13_15193388, 13_15206122, 13_15207670, 13_15251172, 13_15258344, 13_15282196, 13_15293111, 13_15301190, 13_15301311, 13_15360546, 13_15364916, 13_15367981, 13_15397056, 13_15464634, 13_15485959, 13_15504450, 13_15505511, 13_15506661, 13_15522342, 13_15543247, 13_15546082, 13_15576430, 13_15585400, 13_15592180, 13_15612601, 13_15655985, 13_15666565, 13_15682571, 13_15728448, 13_15756217, 13_15757042, 13_15765755, 13_15794200, 13_15816087, 13_15949510, 13_16045301, 13_16058547, 13_16072546, 13_16160423, 13_16203571, 13_16278270, 13_16279848, 13_16280004, 13_16310255, 13_16314963, 13_16315077, 13_16322639, 13_16322928, 13_16332965, 13_16360655, 13_16377640, 13_16414203, 13_16416342, 13_16484025, 13_16524204, 13_16543673, 13_16551346, 13_16616940, 13_16655473, 13_16658546, 13_16670472, 13_16730154, 13_16738801, 13_16739434, 13_16817872, 13_16841548, 13_16860531, 13_16905394, 13_16905918, 13_16926263, 13_16961787, 13_17039911, 13_17043410, 13_17075286, 13_17076268, 13_17089150, 13_17100059, 13_17107037, 13_17107293, 13_17123051, 13_17142100, 13_17143486, 13_17147534, 13_17197781, 13_17205838, 13_17207702, 13_17247258, 13_17254356, 13_17263331, 13_17265994, 13_17276816, 13_17336137, 13_17346696, 13_17350154, 13_17356451, 13_17382990, 13_17418731, 13_17444119, 13_17454849, 13_17456085, 13_17458483, 13_17460505, 13_17467333, 13_17490285, 13_17493293, 13_17503191, 13_17513749, 13_17516092, 13_17519515, 13_17525548, 13_17526493, 13_17539976, 13_17547337, 13_17568928, 13_17607390, 13_17614278, 13_17615347, 13_17632644, 13_17642372, 13_17644419, 13_17672986, 13_17674330, 13_17680111, 13_17680175, 13_17686021, 13_17697064, 13_17701068, 13_17709024, 13_17727372, 13_17730481, 13_17743207, 13_17743436, 13_17781265, 13_17798538, 13_17815708, 13_17828904, 13_17842602, 13_17865572, 13_17896123, 13_17900999, 13_17919491, 13_17943127, 13_17965116, 13_17975030, 13_17975165, 13_17979247, 13_18004787, 13_18012875, 13_18013237, 13_18020823, 13_18023841, 13_18025445, 13_18071322, 13_18073842, 13_18089567, 13_18100648, 13_18122530, 13_18136411, 13_18138854, 13_18167267, 13_18171493, 13_18177396, 13_18200968, 13_18204664, 13_18216403, 13_18220127, 13_18239413, 13_18240308, 13_18265518, 13_18282182, 13_18309805, 13_18321085, 13_18322163, 13_18324097, 13_18357063, 13_18373359, 13_18380424, 13_18407013, 13_18420199, 13_18429431, 13_18447785, 13_18502597, 13_18508306, 13_18532769, 13_18559260, 13_18583954, 13_18608815, 13_18615317, 13_18616279, 13_18621174, 13_18636991, 13_18648397, 13_18653275, 13_18653454, 13_18677454, 13_18711394, 13_18719671, 13_18733401, 13_18777188, 13_18822649, 13_18841674, 13_18849967, 13_18869277, 13_18880164, 13_18925677, 13_18937089, 13_18941741, 13_18943122, 13_18943684, 13_18944294, 13_18949495, 13_18963555, 13_18966730, 13_18989527, 13_19002443, 13_19059589, 13_19063374, 13_19066390, 13_19091329, 13_19105504, 13_19108856, 13_19137295, 13_19214240, 13_19218601, 13_19229266, 13_19240973, 13_19279175, 13_19293994, 13_19294903, 13_19321745, 13_19339258, 13_19340920, 13_19347888, 13_19352495, 13_19378810, 13_19397598, 13_19405278, 13_19429748, 13_19480609, 13_19481264, 13_19495393, 13_19510400, 13_19538770, 13_19541025, 13_19542571, 13_19546808, 13_19547289, 13_19559103, 13_19559191, 13_19590058, 13_19595311, 13_19601542, 13_19605234, 13_19606221, 13_19613316, 13_19617261, 13_19665144, 13_19692970, 13_19710349, 13_19727336, 13_19732676, 13_19784218, 13_19802006, 13_19826069, 13_19875036, 13_19917996, 13_19929333, 13_19956299, 13_19957673, 13_19970553, 13_19985983, 13_19988496, 13_20003964, 13_20010661, 13_20012547, 13_20020334, 13_20071445, 13_20084972, 13_20117869, 13_20118150, 13_20118925, 13_20127356, 13_20225780, 13_20445126, 13_20450152, 13_20629043, 13_20698314, 13_20701025, 13_20794653, 13_20820806, 13_20863000, 13_20881807, 13_20897464, 13_21065827, 13_21082104, 13_21319262, 13_21343487, 13_21373450, 13_21379016, 13_21553050, 13_21572681, 13_21583645, 13_21640365, 13_21749752, 13_21819985, 13_21831480, 13_21836098, 13_21859393, 13_21873921, 13_21973291, 13_22025003, 13_22036688, 13_22046734, 13_22117266, 13_22157320, 13_22157677, 13_22187046, 13_22198864, 13_22242703, 13_22255347, 13_22257410, 13_22284301, 13_22287294, 13_22310794, 13_22356034, 13_22360180, 13_22368557, 13_22388028, 13_22416996, 13_22710895, 13_22729761, 13_22749381, 13_22749468, 13_22771666, 13_22791737, 13_22792021, 13_22910500, 13_22928444, 13_22940640, 13_22976193, 13_23024601, 13_23034808, 13_23091438, 13_23095982, 13_23097360, 13_23122936, 13_23251515, 13_23255512, 13_23289597, 13_23308978, 13_23426144, 13_23496890, 13_23499153, 13_23501355, 13_23560102, 13_23596598, 13_23702254, 13_23749552, 13_23891552, 13_23976029, 13_24010054, 13_24307702, 13_24416287, 13_24439654, 14_26477, 14_77591, 14_132953, 14_318004, 14_326865, 14_327371, 14_348829, 14_433469, 14_460512, 14_603374, 14_605270, 14_632696, 14_632990, 14_710513, 14_724994, 14_959812, 14_1031384, 14_1037800, 14_1061571, 14_1138991, 14_1145131, 14_1250979, 14_1267738, 14_1270229, 14_1287088, 14_1313552, 14_1339497, 14_1343999, 14_1364412, 14_1393990, 14_1404649, 14_1410118, 14_1431850, 14_1480724, 14_1487010, 14_1490850, 14_1508862, 14_1510168, 14_1522280, 14_1525797, 14_1544769, 14_1600772, 14_1611020, 14_1626063, 14_1650848, 14_1699119, 14_1704935, 14_1781663, 14_1786851, 14_1794220, 14_1802116, 14_1807242, 14_1807443, 14_1830576, 14_1830889, 14_1839372, 14_1983908, 14_2004478, 14_2013212, 14_2037900, 14_2072982, 14_2095905, 14_2157900, 14_2199928, 14_2201624, 14_2223379, 14_2306824, 14_2341229, 14_2394456, 14_2410184, 14_2460667, 14_2473114, 14_2517239, 14_2519181, 14_2529303, 14_2531984, 14_2533273, 14_2536328, 14_2537239, 14_2567634, 14_2592218, 14_2597524, 14_2602045, 14_2604634, 14_2619822, 14_2758698, 14_2777894, 14_2816649, 14_2839930, 14_2884069, 14_2908266, 14_2924479, 14_2952659, 14_3074557, 14_3090529, 14_3252200, 14_3269214, 14_3270868, 14_3286035, 14_3310101, 14_3347609, 14_3414483, 14_3507757, 14_3519344, 14_3569586, 14_3591775, 14_3599451, 14_3685073, 14_3757587, 14_3759940, 14_3760479, 14_3761026, 14_3979060, 14_4073479, 14_4080554, 14_4141705, 14_4151381, 14_4207310, 14_4238799, 14_4313852, 14_4389946, 14_4391043, 14_4406476, 14_4436113, 14_4480211, 14_4504220, 14_4511881, 14_4528232, 14_4590741, 14_4600864, 14_4613168, 14_4636443, 14_4659150, 14_4675508, 14_4676822, 14_4699829, 14_4811157, 14_4836247, 14_4840943, 14_4866724, 14_4917174, 14_4952046, 14_4968741, 14_4994322, 14_5020228, 14_5055760, 14_5056965, 14_5085295, 14_5100037, 14_5141024, 14_5156473, 14_5199815, 14_5251381, 14_5287182, 14_5288428, 14_5359778, 14_5369277, 14_5399056, 14_5410054, 14_5458448, 14_5464197, 14_5466807, 14_5470664, 14_5498702, 14_5503637, 14_5528230, 14_5573575, 14_5641969, 14_5647482, 14_5648925, 14_5682639, 14_5684463, 14_5693428, 14_5757686, 14_5763871, 14_5779106, 14_5884599, 14_5892423, 14_5906303, 14_5942482, 14_5975291, 14_5977201, 14_5992686, 14_6030666, 14_6093104, 14_6093564, 14_6094595, 14_6094953, 14_6096826, 14_6098251, 14_6108252, 14_6114419, 14_6125689, 14_6140997, 14_6142087, 14_6153618, 14_6166971, 14_6218415, 14_6253535, 14_6253606, 14_6283894, 14_6285033, 14_6377454, 14_6383528, 14_6437876, 14_6445182, 14_6503282, 14_6521583, 14_6548896, 14_6556532, 14_6573361, 14_6577531, 14_6581578, 14_6601095, 14_6675903, 14_6722244, 14_6758547, 14_6761725, 14_6765335, 14_6828343, 14_6852764, 14_6856846, 14_6857236, 14_6873584, 14_6878183, 14_6878749, 14_6882427, 14_6885791, 14_6894742, 14_6923605, 14_6928641, 14_7021408, 14_7043446, 14_7047464, 14_7049365, 14_7054760, 14_7055336, 14_7056215, 14_7095131, 14_7121535, 14_7125385, 14_7140688, 14_7163635, 14_7165644, 14_7191800, 14_7209811, 14_7209939, 14_7212574, 14_7232040, 14_7301568, 14_7386034, 14_7397321, 14_7492592, 14_7560497, 14_7573128, 14_7599621, 14_7615816, 14_7644191, 14_7668600, 14_7746957, 14_7759600, 14_7776318, 14_7777755, 14_7794906, 14_7831165, 14_7834121, 14_7845490, 14_7916600, 14_7938414, 14_7959002, 14_7960000, 14_8005508, 14_8103186, 14_8124207, 14_8127549, 14_8130948, 14_8145011, 14_8148953, 14_8200919, 14_8224208, 14_8239127, 14_8244087, 14_8283719, 14_8294593, 14_8315613, 14_8364396, 14_8380491, 14_8409733, 14_8482681, 14_8532499, 14_8662722, 14_8663233, 14_8706667, 14_8719560, 14_8720921, 14_8738028, 14_8765780, 14_8784351, 14_8819558, 14_8838679, 14_8868306, 14_8869017, 14_8919144, 14_8924904, 14_8941903, 14_8979713, 14_9036632, 14_9062283, 14_9104245, 14_9114425, 14_9126179, 14_9159949, 14_9220210, 14_9249390, 14_9268068, 14_9274490, 14_9295929, 14_9320166, 14_9334124, 14_9336945, 14_9350072, 14_9358363, 14_9377729, 14_9386089, 14_9392286, 14_9399908, 14_9446811, 14_9452165, 14_9494977, 14_9501477, 14_9518316, 14_9523380, 14_9548418, 14_9548715, 14_9588083, 14_9626432, 14_9712848, 14_9732979, 14_9916675, 14_9935003, 14_9974328, 14_10068689, 14_10170865, 14_10171925, 14_10177811, 14_10211262, 14_10219474, 14_10255388, 14_10263792, 14_10265744, 14_10266921, 14_10297703, 14_10318632, 14_10340213, 14_10391746, 14_10397999, 14_10404411, 14_10411575, 14_10450746, 14_10461185, 14_10471706, 14_10477024, 14_10483571, 14_10518201, 14_10536251, 14_10538193, 14_10565070, 14_10565971, 14_10588590, 14_10591684, 14_10633231, 14_10649837, 14_10659854, 14_10704698, 14_10705210, 14_10707231, 14_10711706, 14_10719955, 14_10756390, 14_10780016, 14_10805283, 14_10822987, 14_10827929, 14_10828662, 14_10867969, 14_10917982, 14_10935128, 14_10982360, 14_11009774, 14_11112627, 14_11118828, 14_11134524, 14_11167071, 14_11176751, 14_11180027, 14_11222885, 14_11240121, 14_11267870, 14_11293224, 14_11354352, 14_11363505, 14_11366111, 14_11440673, 14_11445684, 14_11518926, 14_11524638, 14_11528289, 14_11539326, 14_11615239, 14_11636711, 14_11693691, 14_11697610, 14_11721829, 14_11759125, 14_11760361, 14_11764159, 14_11774629, 14_11774937, 14_11775731, 14_11777149, 14_11868069, 14_11893684, 14_11928423, 14_11947883, 14_12106253, 14_12115112, 14_12115256, 14_12133016, 14_12176258, 14_12207323, 14_12230716, 14_12234181, 14_12283600, 14_12304948, 14_12328705, 14_12334885, 14_12366965, 14_12416044, 14_12418276, 14_12429520, 14_12429951, 14_12431408, 14_12434225, 14_12453795, 14_12466456, 14_12469406, 14_12478760, 14_12499631, 14_12565048, 14_12573853, 14_12597579, 14_12617886, 14_12628512, 14_12629030, 14_12629881, 14_12676683, 14_12707022, 14_12754364, 14_12764594, 14_12789840, 14_12810261, 14_12896671, 14_12907440, 14_12909215, 14_12969144, 14_13001225, 14_13024951, 14_13044166, 14_13115463, 14_13141424, 14_13162174, 14_13162507, 14_13183619, 14_13252135, 14_13254815, 14_13305755, 14_13367926, 14_13415425, 14_13520783, 14_13533743, 14_13564538, 14_13576007, 14_13583725, 14_13586870, 14_13617487, 14_13666788, 14_13676918, 14_13710498, 14_13724474, 14_13777140, 14_13807055, 14_13835200, 14_13883611, 14_13915966, 14_13925391, 14_13951985, 14_13971134, 14_14005630, 14_14006100, 14_14006714, 14_14026190, 14_14077541, 14_14094031, 14_14095965, 14_14225844, 14_14226215, 14_14299486, 14_14302633, 14_14311687, 14_14350502, 14_14374279, 14_14379096, 14_14389048, 14_14395385, 14_14402723, 14_14441292, 14_14468110, 14_14483414, 14_14496432, 14_14554743, 14_14589940, 14_14656625, 14_14723553, 14_14739732, 14_14746453, 14_14807577, 14_14823383, 14_14888473, 14_14951345, 14_14954757, 14_14966412, 14_14981805, 14_14982484, 14_14993605, 14_15000852, 14_15002780, 14_15008807, 14_15012204, 14_15049482, 14_15066025, 14_15105520, 14_15107852, 14_15129589, 14_15153174, 14_15165258, 14_15184202, 14_15192978, 14_15200134, 14_15216798, 14_15217531, 14_15268306, 14_15275676, 14_15335886, 14_15339564, 14_15352989, 14_15379193, 14_15395376, 14_15402622, 14_15418570, 14_15420526, 14_15441051, 14_15443410, 14_15443887, 14_15448789, 14_15455652, 14_15457007, 14_15459970, 14_15532669, 14_15538451, 14_15559344, 14_15579810, 14_15601000, 14_15625214, 14_15662978, 14_15664103, 14_15667466, 14_15711280, 14_15711573, 14_15720373, 14_15750801, 14_15757884, 14_15810007, 14_15853547, 14_15855500, 14_15855955, 14_15868765, 14_15902159, 14_15902342, 14_15945453, 14_15951999, 14_15988352, 14_15990005, 14_15990565, 14_16017581, 14_16036196, 14_16050522, 14_16050618, 14_16053080, 14_16063053, 14_16092414, 14_16109221, 14_16127726, 14_16148540, 14_16156849, 14_16169828, 14_16175007, 14_16190417, 14_16202648, 14_16208304, 14_16211639, 14_16213826, 14_16236637, 14_16250979, 14_16278374, 14_16306597, 14_16313607, 14_16320913, 14_16341096, 14_16359105, 14_16381554, 14_16418413, 14_16431471, 14_16444581, 14_16482370, 14_16488993, 14_16493369, 14_16496999, 14_16497458, 14_16565547, 14_16573966, 14_16583469, 14_16602943, 14_16617112, 14_16617444, 14_16623863, 14_16664863, 14_16722314, 14_16729251, 14_16729473, 14_16756408, 14_16815766, 14_16830626, 14_16841352, 14_16846302, 14_16904079, 14_16930403, 14_16930446, 14_17012148, 14_17013861, 14_17035648, 14_17071230, 14_17073810, 14_17084474, 14_17095287, 14_17095983, 14_17107645, 14_17123946, 14_17192213, 14_17200339, 14_17210553, 14_17244714, 14_17244801, 14_17249707, 14_17250242, 14_17298451, 14_17304735, 14_17306378, 14_17325808, 14_17345916, 14_17353116, 14_17355611, 14_17378045, 14_17402989, 14_17423917, 14_17442249, 14_17445623, 14_17450818, 14_17463602, 14_17467970, 14_17491074, 14_17503816, 14_17511224, 14_17526660, 14_17533878, 14_17543523, 14_17561918, 14_17562950, 14_17581810, 14_17621497, 14_17642682, 14_17658861, 14_17663789, 14_17664189, 14_17682561, 14_17731146, 14_17734516, 14_17746140, 14_17759561, 14_17760367, 14_17794603, 14_17803299, 14_17822709, 14_17845683, 14_17851632, 14_17869816, 14_17872580, 14_17883285, 14_17891266, 14_17907307, 14_17912751, 14_17917062, 14_17930709, 14_17948716, 14_17962618, 14_17971242, 14_17984358, 14_18008466, 14_18017880, 14_18021025, 14_18053017, 14_18090530, 14_18127816, 14_18152005, 14_18165786, 14_18174400, 14_18197351, 14_18206468, 14_18222683, 14_18255118, 14_18269641, 14_18278758, 14_18288492, 14_18317324, 14_18318762, 14_18331456, 14_18356293, 14_18366426, 14_18374522, 14_18423134, 14_18456051, 14_18456187, 14_18512127, 14_18512418, 14_18520186, 14_18521350, 14_18527813, 14_18542091, 14_18596421, 14_18596997, 14_18597425, 14_18598074, 14_18607296, 14_18612594, 14_18643336, 14_18657840, 14_18658712, 14_18706891, 14_18716001, 14_18716450, 14_18721405, 14_18750235, 14_18784646, 14_18790759, 14_18810963, 14_18815062, 14_18831689, 14_18857430, 14_18885642, 14_18901035, 14_18929084, 14_18971778, 14_18973599, 14_18985292, 14_18999904, 14_19012169, 14_19039490, 14_19054880, 14_19064195, 14_19066248, 14_19073664, 14_19074770, 14_19089931, 14_19100379, 14_19108414, 14_19146096, 14_19153228, 14_19187365, 14_19249198, 14_19262403, 14_19295280, 14_19302976, 14_19360577, 14_19361684, 14_19366134, 14_19383256, 14_19409287, 14_19423436, 14_19457398, 14_19462172, 14_19471760, 14_19475351, 14_19485260, 14_19490924, 14_19491138, 14_19493289, 14_19493673, 14_19575152, 14_19592528, 14_19607864, 14_19610632, 14_19634741, 14_19647387, 14_19647510, 14_19648113, 14_19658285, 14_19659815, 14_19674625, 14_19676712, 14_19676984, 14_19680043, 14_19694226, 14_19702375, 14_19750520, 14_19799892, 14_19801281, 14_19816005, 14_19825162, 14_19834230, 14_19836226, 14_19842696, 14_19847661, 14_19963957, 14_20045850, 14_20052268, 14_20055145, 14_20062402, 14_20065627, 14_20073648, 14_20086249, 14_20095442, 14_20098769, 14_20108183, 14_20122510, 14_20145200, 14_20178784, 14_20181481, 14_20188900, 14_20200378, 14_20209172, 14_20252462, 14_20274037, 14_20281408, 14_20290726, 14_20308701, 14_20317580, 14_20348349, 14_20375311, 14_20378628, 14_20383099, 14_20390979, 14_20401479, 14_20401698, 14_20521257, 14_20525340, 14_20545414, 14_20548256, 14_20557189, 14_20561859, 14_20570161, 14_20572245, 14_20586734, 14_20590801, 14_20594085, 14_20601612, 14_20623837, 14_20631209, 14_20632213, 14_20634989, 14_20654877, 14_20683424, 14_20716034, 14_20718439, 14_20730918, 14_20740462, 14_20754374, 14_20755842, 14_20758099, 14_20760902, 14_20765458, 14_20768228, 14_20801458, 14_20826366, 14_20837809, 14_20848098, 14_20848607, 14_20868809, 14_20871103, 14_20877011, 14_20879368, 14_20890659, 14_20897292, 14_20899483, 14_20936297, 14_20943796, 14_20951328, 14_20953525, 14_20966160, 14_21010366, 14_21012222, 14_21014215, 14_21014601, 14_21014748, 14_21036781, 14_21044224, 14_21052549, 14_21056566, 14_21082179, 14_21087850, 14_21089628, 14_21116573, 14_21132047, 14_21150377, 14_21159175, 14_21159527, 14_21163001, 14_21163240, 14_21165250, 14_21170750, 14_21186481, 14_21190928, 14_21192380, 14_21211863, 14_21231502, 14_21235434, 14_21240273, 14_21247350, 14_21254811, 14_21257170, 14_21264118, 14_21285589, 14_21289530, 14_21297181, 14_21309250, 14_21334391, 14_21364022, 14_21367702, 14_21370104, 14_21385910, 14_21391014, 14_21399690, 14_21407314, 14_21409593, 14_21424093, 14_21424279, 14_21425629, 14_21435596, 14_21439333, 14_21441290, 14_21442726, 14_21446705, 14_21466028, 14_21468310, 14_21486217, 14_21495354, 14_21517785, 14_21544196, 14_21544526, 14_21544630, 14_21574817, 14_21579375, 14_21593755, 14_21602431, 14_21608225, 14_21611130, 14_21613707, 14_21621193, 14_21625220, 14_21688697, 14_21689128, 14_21692111, 14_21749333, 14_21754362, 14_21754889, 14_21756766, 14_21764043, 14_21765542, 14_21770613, 14_21809914, 14_21813078, 14_21814029, 14_21814125, 14_21816909, 14_21825811, 14_21854169, 14_21855088, 14_21864437, 14_21873581, 14_21924137, 14_21964723, 14_21969935, 14_21976804, 14_21983996, 14_21987726, 14_21995927, 14_22011092, 14_22054877, 14_22055462, 14_22096886, 14_22109972, 14_22113468, 14_22122177, 14_22158961, 14_22174350, 14_22176768, 14_22179097, 14_22180806, 14_22190287, 14_22195084, 14_22212191, 14_22216443, 14_22218567, 14_22258092, 14_22271344, 14_22282910, 14_22323751, 14_22324448, 14_22324886, 14_22332124, 14_22376763, 14_22401819, 14_22420910, 14_22445712, 14_22448213, 14_22454749, 14_22459497, 14_22491251, 14_22520824, 14_22617836, 14_22619405, 14_22621661, 14_22630349, 14_22641755, 14_22673246, 14_22690334, 14_22696480, 14_22713469, 14_22716326, 14_22716957, 15_11333, 15_48165, 15_185535, 15_235624, 15_323571, 15_366416, 15_400698, 15_421456, 15_437290, 15_454937, 15_475788, 15_476178, 15_476716, 15_479060, 15_496266, 15_509707, 15_510206, 15_515726, 15_584002, 15_591907, 15_669202, 15_676527, 15_676756, 15_717511, 15_757779, 15_778327, 15_856318, 15_860800, 15_866126, 15_875130, 15_900475, 15_902647, 15_932836, 15_959335, 15_963219, 15_983586, 15_1007597, 15_1039322, 15_1124560, 15_1137222, 15_1185413, 15_1255538, 15_1375743, 15_1377037, 15_1401298, 15_1418714, 15_1616169, 15_1632042, 15_1641991, 15_1747901, 15_1818459, 15_1867324, 15_2019065, 15_2019970, 15_2022118, 15_2046430, 15_2052657, 15_2060283, 15_2065712, 15_2073913, 15_2099095, 15_2139383, 15_2175293, 15_2189945, 15_2201669, 15_2206834, 15_2282484, 15_2295846, 15_2371184, 15_2388003, 15_2511366, 15_2520908, 15_2606840, 15_2686231, 15_2732613, 15_2749883, 15_2756157, 15_2776802, 15_2786989, 15_2793132, 15_2807926, 15_2816477, 15_2820290, 15_2821351, 15_2829627, 15_2832796, 15_2834862, 15_2843395, 15_2844244, 15_2872168, 15_2890692, 15_2921707, 15_2954796, 15_2956012, 15_3045790, 15_3106755, 15_3106975, 15_3200573, 15_3201219, 15_3228751, 15_3237392, 15_3261844, 15_3269007, 15_3290660, 15_3318938, 15_3329890, 15_3412383, 15_3412637, 15_3451299, 15_3467368, 15_3557042, 15_3567024, 15_3600035, 15_3626543, 15_3724575, 15_3796788, 15_3836225, 15_3840728, 15_3988496, 15_3994608, 15_4010586, 15_4016284, 15_4035682, 15_4086286, 15_4089810, 15_4147286, 15_4199879, 15_4246041, 15_4274811, 15_4284324, 15_4303779, 15_4304281, 15_4306224, 15_4316555, 15_4325926, 15_4378371, 15_4392601, 15_4401552, 15_4461509, 15_4501205, 15_4518395, 15_4534967, 15_4553501, 15_4563788, 15_4583518, 15_4592378, 15_4595831, 15_4626247, 15_4633093, 15_4643285, 15_4792742, 15_4814342, 15_4819339, 15_4835922, 15_4837559, 15_4849198, 15_4862502, 15_4862918, 15_4871601, 15_4884892, 15_4894088, 15_4951258, 15_4955793, 15_5027279, 15_5029713, 15_5042926, 15_5072782, 15_5121281, 15_5126920, 15_5153630, 15_5197633, 15_5218785, 15_5290714, 15_5301421, 15_5312063, 15_5322625, 15_5327977, 15_5334716, 15_5347989, 15_5356401, 15_5385978, 15_5427052, 15_5434894, 15_5434917, 15_5439434, 15_5442689, 15_5452429, 15_5454263, 15_5469206, 15_5470181, 15_5487978, 15_5508524, 15_5545572, 15_5557304, 15_5567788, 15_5572131, 15_5625241, 15_5625414, 15_5636300, 15_5641536, 15_5807322, 15_5807718, 15_5821872, 15_5838029, 15_5852567, 15_5854634, 15_5862058, 15_5866858, 15_5925413, 15_5927926, 15_5928555, 15_5946457, 15_6024269, 15_6040070, 15_6080959, 15_6107080, 15_6107763, 15_6109950, 15_6112291, 15_6113239, 15_6119024, 15_6125844, 15_6134255, 15_6134458, 15_6156031, 15_6198295, 15_6206925, 15_6214231, 15_6237906, 15_6256276, 15_6263862, 15_6264029, 15_6274766, 15_6306609, 15_6332201, 15_6339308, 15_6343145, 15_6343417, 15_6352691, 15_6355716, 15_6371835, 15_6372654, 15_6383300, 15_6386739, 15_6388827, 15_6403773, 15_6440057, 15_6443903, 15_6463163, 15_6473887, 15_6479708, 15_6481418, 15_6491011, 15_6521611, 15_6549048, 15_6608829, 15_6711871, 15_6742483, 15_6759997, 15_6778251, 15_6795019, 15_6798193, 15_6802986, 15_6805217, 15_6816989, 15_6837319, 15_6855252, 15_6866760, 15_6934711, 15_6988722, 15_7044150, 15_7044539, 15_7111698, 15_7193002, 15_7217444, 15_7238934, 15_7266740, 15_7271858, 15_7277268, 15_7277983, 15_7280293, 15_7328007, 15_7339644, 15_7365349, 15_7369078, 15_7374012, 15_7390118, 15_7392506, 15_7412670, 15_7414653, 15_7419916, 15_7420062, 15_7483398, 15_7527475, 15_7548393, 15_7585215, 15_7602437, 15_7741543, 15_7761035, 15_7764293, 15_7767902, 15_7768258, 15_7826877, 15_7910530, 15_7924805, 15_8011889, 15_8013864, 15_8044093, 15_8071526, 15_8079459, 15_8087622, 15_8094145, 15_8123350, 15_8133254, 15_8162991, 15_8267735, 15_8276162, 15_8310163, 15_8334124, 15_8426673, 15_8426953, 15_8427583, 15_8491611, 15_8547377, 15_8583804, 15_8596184, 15_8602159, 15_8652762, 15_8661378, 15_8687858, 15_8699963, 15_8712748, 15_8717383, 15_8754558, 15_8754806, 15_8786906, 15_8818677, 15_8822530, 15_8825643, 15_8826144, 15_8835015, 15_8863552, 15_8907359, 15_8920099, 15_8988420, 15_9014443, 15_9021290, 15_9039308, 15_9078325, 15_9201487, 15_9226141, 15_9248778, 15_9279463, 15_9305443, 15_9312209, 15_9320613, 15_9334522, 15_9346260, 15_9452444, 15_9483532, 15_9512509, 15_9525910, 15_9543453, 15_9554897, 15_9635668, 15_9647649, 15_9673591, 15_9673658, 15_9673850, 15_9678910, 15_9683372, 15_9724559, 15_9740584, 15_9750195, 15_9765413, 15_9802324, 15_9823193, 15_9837744, 15_9842882, 15_9849272, 15_9862436, 15_9914457, 15_9925983, 15_9953044, 15_9961094, 15_9992862, 15_10001049, 15_10004375, 15_10104452, 15_10119758, 15_10120728, 15_10152686, 15_10253019, 15_10290583, 15_10300143, 15_10308375, 15_10308774, 15_10379256, 15_10384808, 15_10502637, 15_10608417, 15_10632075, 15_10650129, 15_10659893, 15_10665646, 15_10668154, 15_10742180, 15_10816040, 15_10881068, 15_10893683, 15_10893834, 15_10919114, 15_10935528, 15_10945207, 15_10949558, 15_11001846, 15_11015959, 15_11023723, 15_11026738, 15_11033791, 15_11034622, 15_11040288, 15_11065335, 15_11065734, 15_11067551, 15_11109199, 15_11112081, 15_11114844, 15_11118290, 15_11148920, 15_11151571, 15_11174677, 15_11183938, 15_11185052, 15_11203210, 15_11216937, 15_11239323, 15_11241339, 15_11262005, 15_11285675, 15_11296106, 15_11297794, 15_11308990, 15_11313503, 15_11323686, 15_11341950, 15_11360197, 15_11430551, 15_11433656, 15_11434980, 15_11448773, 15_11451754, 15_11457505, 15_11474310, 15_11481340, 15_11558679, 15_11584174, 15_11586051, 15_11593150, 15_11612867, 15_11669009, 15_11669865, 15_11705578, 15_11713598, 15_11727454, 15_11727757, 15_11745159, 15_11777542, 15_11816500, 15_11842566, 15_11871751, 15_11883946, 15_11890665, 15_11939634, 15_11964467, 15_11966859, 15_11969288, 15_11970293, 15_11974851, 15_12007184, 15_12057786, 15_12071517, 15_12089635, 15_12122462, 15_12125294, 15_12133596, 15_12192925, 15_12240477, 15_12242163, 15_12339883, 15_12368407, 15_12412562, 15_12429170, 15_12429614, 15_12471683, 15_12480437, 15_12493773, 15_12499245, 15_12516880, 15_12529585, 15_12558866, 15_12566450, 15_12574083, 15_12582184, 15_12594320, 15_12614985, 15_12644687, 15_12655550, 15_12656692, 15_12665401, 15_12673460, 15_12717355, 15_12732633, 15_12775599, 15_12796621, 15_12798975, 15_12816839, 15_12833018, 15_12854356, 15_12877153, 15_12981666, 15_12987186, 15_13083775, 15_13091558, 15_13100517, 15_13138667, 15_13146915, 15_13165716, 15_13167866, 15_13168567, 15_13177061, 15_13186060, 15_13197015, 15_13208616, 15_13237549, 15_13246913, 15_13296137, 15_13340071, 15_13352099, 15_13357749, 15_13358069, 15_13375191, 15_13419546, 15_13454758, 15_13489328, 15_13525886, 15_13526231, 15_13528970, 15_13569559, 15_13588494, 15_13589464, 15_13608577, 15_13649383, 15_13649600, 15_13665266, 15_13720582, 15_13739070, 15_13754981, 15_13804202, 15_13829496, 15_13837090, 15_13844359, 15_13853464, 15_13869844, 15_13916203, 15_13937599, 15_13942726, 15_13960785, 15_14032058, 15_14051991, 15_14096193, 15_14162555, 15_14198799, 15_14255917, 15_14257085, 15_14257986, 15_14262959, 15_14273065, 15_14300683, 15_14302734, 15_14306732, 15_14325907, 15_14330165, 15_14386048, 15_14477899, 15_14564992, 15_14600256, 15_14618138, 15_14629249, 15_14655126, 15_14657830, 15_14734235, 15_14741766, 15_14763266, 15_14763927, 15_14765313, 15_14798492, 15_14823528, 15_14824088, 15_14839103, 15_14842538, 15_14859428, 15_14882448, 15_14905088, 15_14941776, 15_14949196, 15_14960665, 15_15088447, 15_15094408, 15_15094657, 15_15116453, 15_15138524, 15_15185862, 15_15263182, 15_15271962, 15_15286819, 15_15321138, 15_15334422, 15_15350275, 15_15379037, 15_15386322, 15_15397607, 15_15403405, 15_15408878, 15_15423521, 15_15429486, 15_15462449, 15_15462833, 15_15497293, 15_15497411, 15_15508941, 15_15536637, 15_15542734, 15_15614651, 15_15659079, 15_15691010, 15_15692626, 15_15709697, 15_15710227, 15_15713024, 15_15723303, 15_15734438, 15_15734521, 15_15747717, 15_15772272, 15_15786433, 15_15809722, 15_15853762, 15_15876240, 15_15891177, 15_15901178, 15_15913280, 15_15918361, 15_15970522, 15_15979977, 15_15985177, 15_15990774, 15_15995271, 15_16012932, 15_16057950, 15_16067260, 15_16067644, 15_16069906, 15_16085675, 15_16099538, 15_16100108, 15_16117779, 15_16119013, 15_16122563, 15_16133667, 15_16136200, 15_16187148, 15_16208932, 15_16210617, 15_16217049, 15_16219287, 15_16220242, 15_16222383, 15_16228207, 15_16235764, 15_16312886, 15_16318030, 15_16324040, 15_16369622, 15_16373415, 15_16376330, 15_16383497, 15_16421410, 15_16424617, 15_16430525, 15_16430819, 15_16465158, 15_16517334, 15_16520410, 15_16525657, 15_16531283, 15_16548576, 15_16551093, 15_16552194, 15_16554373, 15_16556260, 15_16558144, 15_16594829, 15_16605713, 15_16606627, 15_16613716, 15_16616684, 15_16621138, 15_16633829, 15_16643296, 15_16644894, 15_16649702, 15_16653870, 15_16689842, 15_16690914, 15_16693361, 15_16693915, 15_16706355, 15_16720936, 15_16727729, 15_16731012, 15_16762545, 15_16768878, 15_16800428, 15_16820857, 15_16851902, 15_16869545, 15_16898312, 15_16902813, 15_16920806, 15_16928235, 15_16932748, 15_16955117, 15_17000654, 15_17012396, 15_17055624, 15_17075412, 15_17078722, 15_17094236, 15_17120761, 15_17132757, 15_17147660, 15_17158228, 15_17204467, 15_17233416, 15_17258343, 15_17278635, 15_17291667, 15_17308037, 15_17317345, 15_17345102, 15_17347413, 15_17348073, 15_17349443, 15_17368978, 15_17369746, 15_17377856, 15_17390674, 15_17442395, 15_17446773, 15_17497251, 15_17500689, 15_17503247, 15_17580071, 15_17582784, 15_17590390, 15_17592338, 15_17607188, 15_17617341, 15_17626497, 15_17643657, 15_17661947, 15_17663166, 15_17664045, 15_17680211, 15_17699710, 15_17705339, 15_17706316, 15_17712872, 15_17730509, 15_17732640, 15_17733476, 15_17736552, 15_17800447, 15_17807330, 15_17817613, 15_17817800, 15_17819231, 15_17841052, 15_17844044, 15_17846051, 15_17852988, 15_17866610, 15_17892145, 15_17892763, 15_17895055, 15_17897324, 15_18002376, 15_18007995, 15_18016940, 15_18019573, 15_18025841, 15_18080175, 15_18082460, 15_18093315, 15_18104749, 15_18113173, 15_18119402, 15_18123031, 15_18127512, 15_18132350, 15_18170289, 15_18170652, 15_18170837, 15_18172569, 15_18176672, 15_18194945, 15_18212733, 15_18224714, 15_18244290, 15_18257936, 15_18273865, 15_18305988, 15_18306017, 15_18312064, 15_18315244, 15_18328554, 15_18366023, 15_18375427, 15_18414445, 15_18447090, 15_18461747, 15_18479122, 15_18510419, 15_18526976, 15_18551507, 15_18582926, 15_18591909, 15_18618096, 15_18625542, 15_18631209, 15_18632779, 15_18639081, 15_18639486, 15_18647588, 15_18653408, 15_18666475, 15_18670296, 15_18680325, 15_18692334, 15_18692717, 15_18695210, 15_18729838, 15_18772811, 15_18787899, 15_18789723, 15_18808916, 15_18835229, 15_18863581, 15_18892191, 15_18922242, 15_18923284, 15_18930098, 15_18964827, 15_18966536, 15_18979065, 15_18992222, 15_19023499, 15_19061880, 15_19066178, 15_19067556, 15_19135178, 15_19148349, 15_19151633, 15_19170677, 15_19183277, 15_19185490, 15_19217040, 15_19246241, 15_19246457, 15_19298679, 15_19348658, 15_19351819, 15_19354690, 15_19364329, 15_19366582, 15_19368184, 15_19387194, 15_19444715, 15_19452536, 15_19460664, 15_19479500, 15_19490975, 15_19498154, 15_19529626, 15_19546646, 15_19550193, 15_19550271, 15_19556057, 15_19572945, 15_19585166, 15_19588426, 15_19599171, 15_19599529, 15_19624319, 15_19631572, 15_19631777, 15_19644660, 15_19660787, 15_19661259, 15_19662680, 15_19683744, 15_19710379, 15_19739401, 15_19745534, 15_19767746, 15_19786990, 15_19788874, 15_19789220, 15_19792088, 15_19794175, 15_19805499, 15_19818622, 15_19831262, 15_19876989, 15_19877507, 15_19898749, 15_19900613, 15_19900986, 15_19902546, 15_19945073, 15_19994155, 15_20008024, 15_20009727, 15_20034044, 15_20068964, 15_20083159, 15_20093274, 15_20094612, 15_20115303, 15_20116018, 15_20123171, 15_20128818, 15_20140230, 15_20164033, 15_20184653, 15_20184983, 15_20186192, 15_20187129, 15_20243254, 15_20248639, 15_20267943, 15_20269987, 15_20276411, 15_20319387, 15_20331558, 15_20341555, 15_20345278, 15_20349901, 15_20350950, 15_20360626, 15_20416730, 15_20419311, 15_20420002, 15_20454137, 15_20457072, 15_20462423, 15_20470942, 15_20472863, 15_20496960, 15_20506792, 15_20515366, 15_20529196, 15_20529600, 15_20530879, 15_20533076, 15_20588811, 15_20646742, 15_20648495, 15_20665006, 15_20668847, 15_20701257, 15_20706688, 15_20733764, 15_20760423, 15_20765054, 15_20771227, 15_20808854, 15_20821106, 15_20838572, 15_20839185, 15_20880073, 15_20892134, 15_20899418, 15_20938269, 15_20938482, 15_20948214, 15_20957287, 15_20963712, 15_21003020, 15_21005106, 15_21006073, 15_21006346, 15_21012083, 15_21013683, 15_21023076, 15_21023390, 15_21030328, 15_21046602, 15_21064002, 15_21073750, 15_21097213, 15_21108953, 15_21111413, 15_21118511, 15_21120847, 15_21125551, 15_21133169, 15_21168069, 15_21188197, 15_21204034, 15_21209508, 15_21222849, 15_21224999, 15_21231538, 15_21238276, 15_21257663, 15_21273507, 15_21293997, 15_21341985, 15_21343081, 15_21363306, 15_21376699, 15_21411027, 15_21434418, 15_21472599, 15_21475648, 15_21486085, 15_21486615, 15_21486641, 15_21494707, 15_21519353, 15_21523871, 15_21529951, 15_21540620, 15_21546932, 15_21578086, 15_21594087, 15_21616094, 15_21627292, 15_21639512, 15_21657701, 15_21666279, 15_21667764, 15_21698316, 15_21704825, 15_21721706, 15_21750612, 15_21758513, 15_21760450, 15_21772893, 16_9309, 16_64996, 16_68141, 16_68987, 16_74186, 16_77122, 16_79381, 16_79451, 16_80205, 16_80407, 16_107187, 16_110761, 16_117013, 16_126818, 16_146344, 16_166176, 16_171528, 16_192036, 16_208545, 16_218867, 16_225706, 16_227623, 16_230056, 16_253932, 16_283961, 16_288307, 16_292083, 16_333178, 16_336843, 16_373930, 16_378447, 16_400155, 16_406326, 16_419186, 16_420120, 16_451549, 16_454669, 16_454747, 16_466855, 16_467497, 16_476225, 16_479627, 16_495583, 16_498592, 16_503434, 16_504147, 16_510159, 16_510720, 16_511488, 16_511891, 16_524094, 16_536294, 16_545644, 16_557167, 16_566459, 16_576934, 16_584906, 16_597479, 16_619376, 16_684469, 16_710585, 16_714638, 16_715116, 16_719491, 16_728130, 16_735568, 16_746955, 16_750549, 16_750866, 16_762306, 16_784114, 16_785114, 16_793762, 16_819317, 16_819683, 16_829023, 16_829832, 16_840951, 16_844711, 16_871418, 16_885192, 16_885778, 16_892436, 16_922871, 16_936514, 16_956812, 16_958181, 16_964737, 16_983523, 16_983961, 16_1001708, 16_1038722, 16_1076096, 16_1076316, 16_1081606, 16_1085028, 16_1121477, 16_1121900, 16_1153269, 16_1168835, 16_1180242, 16_1188392, 16_1190485, 16_1190692, 16_1190812, 16_1192617, 16_1200573, 16_1206596, 16_1211566, 16_1220502, 16_1228845, 16_1230675, 16_1231019, 16_1233926, 16_1237725, 16_1250206, 16_1275445, 16_1279070, 16_1292835, 16_1326672, 16_1341694, 16_1378264, 16_1430693, 16_1462889, 16_1468450, 16_1479557, 16_1504260, 16_1508656, 16_1523354, 16_1526595, 16_1535037, 16_1580271, 16_1601726, 16_1611708, 16_1613644, 16_1634658, 16_1638466, 16_1654217, 16_1664410, 16_1680416, 16_1713027, 16_1719673, 16_1720631, 16_1726990, 16_1740518, 16_1752672, 16_1763262, 16_1766450, 16_1788047, 16_1791967, 16_1793626, 16_1794088, 16_1803941, 16_1804055, 16_1810822, 16_1811398, 16_1811760, 16_1814208, 16_1815600, 16_1836271, 16_1843283, 16_1844935, 16_1845737, 16_1849230, 16_1875695, 16_1881818, 16_1891634, 16_1969882, 16_1972206, 16_2019550, 16_2025520, 16_2049263, 16_2065220, 16_2088132, 16_2089730, 16_2181968, 16_2205907, 16_2207862, 16_2219673, 16_2221946, 16_2222666, 16_2238295, 16_2239530, 16_2252294, 16_2254518, 16_2255810, 16_2263650, 16_2263787, 16_2267279, 16_2269768, 16_2281591, 16_2284948, 16_2297989, 16_2303237, 16_2326218, 16_2373992, 16_2421435, 16_2427022, 16_2428554, 16_2429184, 16_2431271, 16_2433948, 16_2434167, 16_2435367, 16_2443302, 16_2450516, 16_2456605, 16_2486977, 16_2494168, 16_2495710, 16_2495779, 16_2514740, 16_2523373, 16_2527668, 16_2532234, 16_2539426, 16_2566173, 16_2572398, 16_2592822, 16_2601659, 16_2612648, 16_2613730, 16_2622054, 16_2622411, 16_2624388, 16_2655635, 16_2673782, 16_2676777, 16_2682240, 16_2687867, 16_2700528, 16_2713591, 16_2722379, 16_2765978, 16_2777255, 16_2777663, 16_2788364, 16_2789070, 16_2797843, 16_2801398, 16_2803891, 16_2823364, 16_2827125, 16_2838762, 16_2850223, 16_2861576, 16_2882532, 16_2884793, 16_2894988, 16_2895328, 16_2945902, 16_2987350, 16_2995767, 16_3011078, 16_3054417, 16_3064508, 16_3068450, 16_3072444, 16_3085994, 16_3107166, 16_3112788, 16_3131164, 16_3131631, 16_3134738, 16_3138680, 16_3162715, 16_3177399, 16_3180894, 16_3210015, 16_3222549, 16_3235130, 16_3239561, 16_3258989, 16_3266987, 16_3267585, 16_3282748, 16_3306119, 16_3323969, 16_3329778, 16_3338573, 16_3353910, 16_3358993, 16_3359210, 16_3366929, 16_3384902, 16_3393501, 16_3415989, 16_3449118, 16_3450079, 16_3459527, 16_3468941, 16_3470624, 16_3479310, 16_3501106, 16_3528139, 16_3548321, 16_3573759, 16_3575994, 16_3614832, 16_3618088, 16_3624051, 16_3703715, 16_3800535, 16_3808256, 16_3848718, 16_3855909, 16_3864329, 16_3942123, 16_3954160, 16_3969332, 16_3969791, 16_3975687, 16_3992064, 16_4004820, 16_4008602, 16_4012705, 16_4029439, 16_4073436, 16_4116614, 16_4202823, 16_4206084, 16_4213764, 16_4214138, 16_4222364, 16_4283961, 16_4302973, 16_4303490, 16_4315311, 16_4349557, 16_4351217, 16_4355002, 16_4393456, 16_4407698, 16_4412464, 16_4457199, 16_4472677, 16_4486144, 16_4495879, 16_4625409, 16_4647717, 16_4662365, 16_4707192, 16_4712528, 16_4714802, 16_4732994, 16_4733465, 16_4739896, 16_4749973, 16_4772465, 16_4778802, 16_4824374, 16_4827748, 16_4828434, 16_4867652, 16_4877493, 16_4881956, 16_4884180, 16_4913516, 16_4967706, 16_4988053, 16_5015361, 16_5056723, 16_5084441, 16_5179048, 16_5198703, 16_5210394, 16_5243304, 16_5249659, 16_5272426, 16_5273454, 16_5273713, 16_5284193, 16_5315592, 16_5340791, 16_5355857, 16_5365101, 16_5371111, 16_5388414, 16_5394630, 16_5421638, 16_5425071, 16_5440803, 16_5444279, 16_5449059, 16_5458537, 16_5469190, 16_5521722, 16_5537670, 16_5543499, 16_5555326, 16_5559651, 16_5572775, 16_5584737, 16_5587534, 16_5590035, 16_5593124, 16_5600096, 16_5610305, 16_5616039, 16_5620397, 16_5665302, 16_5681568, 16_5694055, 16_5694313, 16_5699234, 16_5701475, 16_5702220, 16_5744931, 16_5748721, 16_5786362, 16_5798541, 16_5803113, 16_5805322, 16_5814931, 16_5817783, 16_5842595, 16_5842712, 16_5856644, 16_5860916, 16_5875949, 16_5907309, 16_5907578, 16_5916392, 16_5919873, 16_5928368, 16_5941052, 16_5943920, 16_5980499, 16_6027205, 16_6027806, 16_6029431, 16_6062986, 16_6100781, 16_6107280, 16_6122146, 16_6134761, 16_6164291, 16_6246340, 16_6246748, 16_6285744, 16_6381399, 16_6393839, 16_6408808, 16_6434187, 16_6436347, 16_6438469, 16_6457665, 16_6466389, 16_6495057, 16_6508976, 16_6527377, 16_6587045, 16_6590226, 16_6599519, 16_6605526, 16_6624558, 16_6639162, 16_6649556, 16_6687960, 16_6693480, 16_6730344, 16_6738877, 16_6761615, 16_6814685, 16_6816882, 16_6859138, 16_6865804, 16_6887149, 16_6896350, 16_6902685, 16_6950553, 16_6957561, 16_7066621, 16_7072340, 16_7141999, 16_7171210, 16_7185855, 16_7201340, 16_7246997, 16_7262222, 16_7290844, 16_7298426, 16_7298646, 16_7298959, 16_7305925, 16_7314350, 16_7330290, 16_7348022, 16_7353064, 16_7393387, 16_7480395, 16_7501910, 16_7501990, 16_7506883, 16_7508594, 16_7540818, 16_7547312, 16_7597940, 16_7600820, 16_7608537, 16_7642991, 16_7678575, 16_7710137, 16_7757704, 16_7769544, 16_7775899, 16_7819747, 16_7820653, 16_7826585, 16_7828374, 16_7828926, 16_7834004, 16_7888074, 16_7923685, 16_7946844, 16_7962885, 16_8023296, 16_8027163, 16_8040164, 16_8122253, 16_8138700, 16_8152426, 16_8172821, 16_8173331, 16_8176828, 16_8238275, 16_8266520, 16_8278439, 16_8287686, 16_8303126, 16_8319007, 16_8357927, 16_8388909, 16_8396896, 16_8417125, 16_8421255, 16_8448090, 16_8455533, 16_8504562, 16_8522191, 16_8529509, 16_8530117, 16_8530343, 16_8548393, 16_8589466, 16_8598513, 16_8681185, 16_8701594, 16_8706927, 16_8707347, 16_8710055, 16_8725999, 16_8734340, 16_8759046, 16_8789455, 16_8789750, 16_8791632, 16_8792767, 16_8846753, 16_8861229, 16_8861692, 16_8863172, 16_8876802, 16_8892136, 16_8908491, 16_8909308, 16_8952820, 16_8967431, 16_9008404, 16_9021041, 16_9064936, 16_9126181, 16_9135048, 16_9156525, 16_9172735, 16_9215582, 16_9244729, 16_9246570, 16_9259453, 16_9266699, 16_9266742, 16_9275797, 16_9278063, 16_9325635, 16_9386709, 16_9389963, 16_9525015, 16_9554606, 16_9555375, 16_9557100, 16_9591755, 16_9636271, 16_9636418, 16_9640027, 16_9649109, 16_9689874, 16_9719268, 16_9726799, 16_9733114, 16_9817609, 16_9829852, 16_9870701, 16_9881839, 16_9892532, 16_9900832, 16_9910370, 16_9948004, 16_10049038, 16_10059288, 16_10108450, 16_10157091, 16_10220097, 16_10235179, 16_10349965, 16_10364176, 16_10432262, 16_10580420, 16_10688153, 16_10710688, 16_10711724, 16_10779695, 16_10792297, 16_10815448, 16_10866461, 16_10885769, 16_10923902, 16_10949866, 16_10969478, 16_10977445, 16_10990219, 16_10998196, 16_11013519, 16_11034334, 16_11042195, 16_11042552, 16_11043901, 16_11051316, 16_11076946, 16_11097002, 16_11109959, 16_11129732, 16_11151393, 16_11173236, 16_11217304, 16_11230257, 16_11241941, 16_11286594, 16_11341894, 16_11381508, 16_11407558, 16_11407765, 16_11411522, 16_11413168, 16_11421323, 16_11448076, 16_11495935, 16_11508705, 16_11515535, 16_11532358, 16_11532701, 16_11591180, 16_11595850, 16_11627995, 16_11661277, 16_11677693, 16_11707402, 16_11711424, 16_11713795, 16_11717196, 16_11719080, 16_11811825, 16_11826595, 16_11844587, 16_11859798, 16_11879206, 16_11894012, 16_11910854, 16_11964044, 16_12013603, 16_12015925, 16_12018854, 16_12046960, 16_12059342, 16_12061610, 16_12069390, 16_12090278, 16_12156657, 16_12163848, 16_12166846, 16_12184339, 16_12184476, 16_12231954, 16_12267870, 16_12271625, 16_12303864, 16_12304100, 16_12315462, 16_12330078, 16_12365294, 16_12392724, 16_12435780, 16_12472635, 16_12487779, 16_12532842, 16_12577216, 16_12577643, 16_12578858, 16_12583058, 16_12609683, 16_12668057, 16_12683983, 16_12712365, 16_12728646, 16_12735947, 16_12741579, 16_12747851, 16_12808062, 16_12811313, 16_12846963, 16_12858063, 16_12881607, 16_12938642, 16_13009945, 16_13016271, 16_13040652, 16_13041548, 16_13050643, 16_13051120, 16_13060262, 16_13075132, 16_13078204, 16_13103722, 16_13126267, 16_13138158, 16_13157151, 16_13159419, 16_13193784, 16_13195202, 16_13238325, 16_13303095, 16_13389541, 16_13421586, 16_13436458, 16_13474663, 16_13475097, 16_13483406, 16_13489097, 16_13501984, 16_13516017, 16_13559562, 16_13608681, 16_13640777, 16_13641018, 16_13641345, 16_13642749, 16_13658907, 16_13683752, 16_13711049, 16_13733629, 16_13745875, 16_13756598, 16_13805456, 16_13820917, 16_13822649, 16_13826074, 16_13879350, 16_13894678, 16_13948145, 16_13956668, 16_13979543, 16_13985610, 16_13991675, 16_13995999, 16_14004966, 16_14005270, 16_14008086, 16_14010111, 16_14030566, 16_14030974, 16_14033433, 16_14047073, 16_14047437, 16_14052894, 16_14113039, 16_14158814, 16_14192837, 16_14196173, 16_14212245, 16_14215874, 16_14237259, 16_14263778, 16_14300870, 16_14306841, 16_14320573, 16_14337092, 16_14342581, 16_14352763, 16_14373137, 16_14387214, 16_14395722, 16_14396677, 16_14436610, 16_14440772, 16_14442640, 16_14454891, 16_14466566, 16_14511474, 16_14535623, 16_14554292, 16_14561231, 16_14625389, 16_14626372, 16_14638997, 16_14642863, 16_14646792, 16_14647695, 16_14678724, 16_14706904, 16_14717952, 16_14733938, 16_14760315, 16_14767675, 16_14790295, 16_14866787, 16_14951048, 16_14952507, 16_14953642, 16_15008217, 16_15058824, 16_15069958, 16_15083609, 16_15087575, 16_15095075, 16_15128638, 16_15131959, 16_15203585, 16_15233331, 16_15252319, 16_15265463, 16_15356192, 16_15458637, 16_15521838, 16_15584194, 16_15597850, 16_15631677, 16_15661166, 16_15664513, 16_15693988, 16_15723868, 16_15735696, 16_15756397, 16_15822453, 16_15832005, 16_15843863, 16_15861529, 16_15871525, 16_15906814, 16_15928062, 16_15937763, 16_15940277, 16_15958654, 16_15961003, 16_15986721, 16_16028016, 16_16035828, 16_16043319, 16_16067781, 16_16069530, 16_16107765, 16_16110690, 16_16119528, 16_16140519, 16_16152507, 16_16160027, 16_16162434, 16_16171919, 16_16201822, 16_16245651, 16_16254814, 16_16262325, 16_16278752, 16_16331826, 16_16342973, 16_16476100, 16_16523155, 16_16529179, 16_16533534, 16_16533689, 16_16541484, 16_16544469, 16_16579071, 16_16604705, 16_16611452, 16_16614300, 16_16616751, 16_16617478, 16_16637819, 16_16645258, 16_16660870, 16_16660987, 16_16688233, 16_16690117, 16_16727071, 16_16757684, 16_16771359, 16_16801396, 16_16835634, 16_16841790, 16_16841971, 16_16894428, 16_16894903, 16_16905247, 16_16918334, 16_16961099, 16_16967780, 16_16976084, 16_17031551, 16_17102103, 16_17118203, 16_17129677, 16_17188021, 16_17194705, 16_17220411, 16_17281086, 16_17310736, 16_17330763, 16_17381765, 16_17384982, 16_17407799, 16_17408134, 16_17480742, 16_17480882, 16_17488566, 16_17528376, 16_17564158, 16_17596453, 16_17602042, 16_17682144, 16_17698756, 16_17714023, 16_17812882, 16_17818177, 16_17835768, 16_17864116, 16_17886603, 16_17911923, 16_17941214, 16_17945769, 16_18007944, 16_18052785, 16_18063275, 16_18065582, 16_18090593, 16_18162054, 16_18162484, 16_18242217, 16_18257104, 16_18276525, 16_18292234, 16_18414857, 16_18432588, 16_18455746, 16_18456951, 16_18457515, 16_18457819, 16_18532030, 16_18553093, 16_18560519, 16_18568128, 16_18568357, 16_18581970, 16_18592026, 16_18599880, 16_18662156, 16_18676557, 16_18688954, 16_18708080, 16_18712157, 16_18729059, 16_18748149, 16_18749145, 16_18763177, 16_18765248, 16_18766691, 16_18853524, 16_18921071, 16_19111413, 16_19171549, 16_19199749, 16_19224230, 16_19227062, 16_19254534, 16_19264199, 16_19333444, 16_19360962, 16_19407243, 16_19411303, 16_19437554, 16_19461756, 16_19472919, 16_19498759, 16_19500024, 16_19501624, 16_19506036, 16_19507941, 16_19511931, 16_19513781, 16_19592953, 16_19613960, 16_19617614, 16_19621202, 16_19668972, 16_19687427, 16_19695029, 16_19749742, 16_19756362, 16_19757720, 16_19800106, 16_19802589, 16_19807649, 16_19813571, 16_19815155, 16_19824717, 16_19837150, 16_19840003, 16_19845536, 16_19845857, 16_19853437, 16_19884890, 16_19897334, 16_19943844, 16_19948472, 16_19954938, 16_19959650, 16_20004993, 16_20005434, 16_20012678, 16_20021425, 16_20021906, 16_20029160, 16_20049045, 16_20090094, 16_20090335, 16_20104112, 16_20115480, 16_20133544, 16_20139737, 16_20146751, 16_20185531, 16_20190564, 16_20194584, 16_20199639, 16_20214467, 16_20236247, 16_20244353, 16_20253653, 16_20264497, 16_20275218, 16_20301108, 16_20309020, 16_20361057, 16_20362452, 16_20370456, 16_20384564, 16_20393836, 16_20405376, 16_20436446, 16_20466495, 16_20466574, 16_20472753, 16_20474384, 16_20493266, 16_20497315, 16_20501275, 16_20504666, 16_20507126, 16_20574267, 16_20581150, 16_20597615, 16_20597699, 16_20609303, 16_20612053, 16_20631050, 16_20636304, 16_20654347, 16_20654735, 16_20659903, 16_20690721, 16_20696122, 16_20699523, 16_20729356, 16_20734279, 16_20740956, 16_20760511, 16_20785315, 16_20789616, 16_20794495, 16_20796882, 16_20804757, 16_20808882, 16_20853403, 16_20867557, 16_20872498, 16_20874275, 16_20875101, 16_20884586, 16_20905425, 16_20950169, 16_20966558, 16_20969292, 16_20979889, 16_20982747, 16_20989239, 16_21005589, 16_21007333, 16_21012338, 16_21034273, 16_21082172, 16_21084378, 16_21094229, 16_21110215, 16_21114710, 16_21119361, 16_21125638, 16_21155290, 16_21166529, 16_21170967, 16_21175883, 16_21183461, 16_21196820, 16_21203809, 16_21233870, 16_21253591, 16_21282953, 16_21289653, 16_21310531, 16_21310771, 16_21322388, 16_21338001, 16_21346889, 16_21353544, 16_21409452, 16_21452027, 17_39698, 17_49378, 17_69643, 17_84164, 17_86087, 17_109818, 17_127909, 17_128629, 17_132042, 17_165408, 17_168195, 17_174778, 17_182666, 17_190172, 17_210874, 17_239367, 17_264395, 17_286536, 17_293693, 17_330918, 17_340961, 17_349190, 17_367637, 17_367948, 17_370387, 17_392314, 17_395439, 17_395711, 17_406796, 17_432430, 17_443575, 17_465128, 17_465410, 17_487628, 17_500806, 17_517622, 17_529707, 17_529777, 17_531447, 17_546872, 17_547778, 17_551636, 17_553616, 17_560022, 17_636308, 17_665939, 17_680663, 17_716471, 17_780225, 17_801650, 17_871849, 17_873198, 17_877581, 17_878225, 17_879261, 17_888334, 17_902789, 17_907830, 17_912956, 17_918107, 17_926309, 17_956197, 17_962688, 17_965072, 17_968836, 17_969340, 17_979656, 17_991538, 17_995190, 17_995534, 17_1002085, 17_1013328, 17_1016031, 17_1020019, 17_1034577, 17_1039038, 17_1041071, 17_1049365, 17_1058452, 17_1074667, 17_1081923, 17_1131930, 17_1138770, 17_1148986, 17_1168144, 17_1168350, 17_1234467, 17_1252581, 17_1282325, 17_1295440, 17_1295694, 17_1306225, 17_1357569, 17_1379733, 17_1380269, 17_1388136, 17_1392946, 17_1395763, 17_1435286, 17_1464953, 17_1516339, 17_1532032, 17_1541030, 17_1542074, 17_1551299, 17_1562270, 17_1566290, 17_1608137, 17_1610455, 17_1652842, 17_1658940, 17_1683854, 17_1687846, 17_1704187, 17_1724087, 17_1728208, 17_1737137, 17_1768640, 17_1772468, 17_1787919, 17_1788463, 17_1789863, 17_1797697, 17_1860376, 17_1865382, 17_1865927, 17_1874411, 17_1888103, 17_1900057, 17_1927100, 17_1928609, 17_1931601, 17_1960838, 17_1969874, 17_1980115, 17_1991067, 17_2012284, 17_2021542, 17_2032912, 17_2036881, 17_2052557, 17_2062507, 17_2095695, 17_2106849, 17_2109834, 17_2112258, 17_2114880, 17_2119007, 17_2133536, 17_2166911, 17_2188350, 17_2221107, 17_2221998, 17_2223625, 17_2228254, 17_2233275, 17_2272059, 17_2274897, 17_2285620, 17_2298893, 17_2302251, 17_2314249, 17_2328515, 17_2339150, 17_2381971, 17_2397538, 17_2405257, 17_2479396, 17_2481960, 17_2483653, 17_2537996, 17_2556919, 17_2566613, 17_2616333, 17_2634615, 17_2637719, 17_2656389, 17_2662259, 17_2669398, 17_2709115, 17_2757780, 17_2764528, 17_2769610, 17_2772096, 17_2772338, 17_2776651, 17_2784501, 17_2793208, 17_2797804, 17_2799229, 17_2812150, 17_2816959, 17_2823719, 17_2835030, 17_2869414, 17_2872386, 17_2919994, 17_2927988, 17_2937010, 17_2969396, 17_2996038, 17_3011116, 17_3014613, 17_3024634, 17_3036503, 17_3052452, 17_3052542, 17_3071405, 17_3092721, 17_3117093, 17_3127773, 17_3141557, 17_3152957, 17_3164383, 17_3209397, 17_3237520, 17_3237842, 17_3288829, 17_3301532, 17_3319558, 17_3320252, 17_3350520, 17_3351347, 17_3366179, 17_3391237, 17_3394839, 17_3400036, 17_3400560, 17_3406685, 17_3434291, 17_3499317, 17_3500601, 17_3502327, 17_3517157, 17_3517414, 17_3545753, 17_3590339, 17_3596095, 17_3597180, 17_3617667, 17_3629791, 17_3668112, 17_3691505, 17_3704269, 17_3717563, 17_3723950, 17_3731695, 17_3735302, 17_3737868, 17_3761354, 17_3762742, 17_3803337, 17_3809174, 17_3827022, 17_3833240, 17_3837775, 17_3845966, 17_3873711, 17_3884209, 17_3896633, 17_3940079, 17_3951663, 17_3953812, 17_3954432, 17_3962616, 17_3971245, 17_3972043, 17_3983658, 17_3991990, 17_4013110, 17_4035318, 17_4039481, 17_4062456, 17_4070554, 17_4105606, 17_4137082, 17_4137355, 17_4154051, 17_4164399, 17_4199545, 17_4204466, 17_4204698, 17_4206218, 17_4206682, 17_4212001, 17_4213537, 17_4216603, 17_4227434, 17_4247402, 17_4265401, 17_4267244, 17_4267735, 17_4294024, 17_4296507, 17_4303187, 17_4349451, 17_4412416, 17_4413991, 17_4428059, 17_4441113, 17_4452462, 17_4468218, 17_4489812, 17_4491970, 17_4496009, 17_4528203, 17_4539012, 17_4544431, 17_4548651, 17_4553486, 17_4571089, 17_4633939, 17_4634417, 17_4650322, 17_4669892, 17_4719375, 17_4759067, 17_4789127, 17_4790076, 17_4794857, 17_4812767, 17_4841263, 17_4853644, 17_4859038, 17_4871961, 17_4917003, 17_4917676, 17_4931095, 17_4945548, 17_4949264, 17_5011667, 17_5016699, 17_5022730, 17_5033610, 17_5078918, 17_5101869, 17_5119032, 17_5178260, 17_5182209, 17_5187921, 17_5189286, 17_5282472, 17_5335156, 17_5343226, 17_5361813, 17_5374090, 17_5387333, 17_5415245, 17_5432883, 17_5446023, 17_5466114, 17_5470639, 17_5537743, 17_5554596, 17_5554988, 17_5563778, 17_5574675, 17_5584768, 17_5584969, 17_5593537, 17_5599379, 17_5606206, 17_5606675, 17_5629719, 17_5636668, 17_5686173, 17_5730274, 17_5753015, 17_5809597, 17_5857301, 17_5862103, 17_5904233, 17_5909223, 17_5922362, 17_5929259, 17_5969792, 17_6005491, 17_6005747, 17_6018939, 17_6021578, 17_6022106, 17_6023734, 17_6023891, 17_6034608, 17_6066970, 17_6068915, 17_6126900, 17_6135482, 17_6145841, 17_6153422, 17_6175286, 17_6187029, 17_6201873, 17_6215676, 17_6231806, 17_6232644, 17_6244391, 17_6259216, 17_6262880, 17_6290128, 17_6306845, 17_6314452, 17_6373315, 17_6388268, 17_6402628, 17_6406645, 17_6430986, 17_6441873, 17_6497280, 17_6539511, 17_6543155, 17_6572768, 17_6575023, 17_6590642, 17_6613534, 17_6670095, 17_6689949, 17_6693464, 17_6721573, 17_6735693, 17_6763085, 17_6788152, 17_6819405, 17_6819696, 17_6833327, 17_6858448, 17_6878380, 17_6885342, 17_6937413, 17_6963472, 17_6979475, 17_7032752, 17_7036430, 17_7069204, 17_7074268, 17_7076553, 17_7137082, 17_7142887, 17_7165651, 17_7189972, 17_7195274, 17_7213396, 17_7283953, 17_7286268, 17_7322477, 17_7332088, 17_7354156, 17_7363202, 17_7371791, 17_7393307, 17_7428960, 17_7482814, 17_7486604, 17_7487240, 17_7504470, 17_7511087, 17_7529742, 17_7550657, 17_7553774, 17_7554230, 17_7569029, 17_7582563, 17_7639225, 17_7670413, 17_7694829, 17_7695097, 17_7795551, 17_7842701, 17_7844074, 17_7854474, 17_7858760, 17_7865380, 17_7867127, 17_7904123, 17_7980270, 17_8016036, 17_8045463, 17_8088554, 17_8091229, 17_8165613, 17_8211786, 17_8245039, 17_8308966, 17_8329301, 17_8376904, 17_8445100, 17_8447963, 17_8470866, 17_8474919, 17_8488147, 17_8516138, 17_8528478, 17_8531552, 17_8533236, 17_8549635, 17_8556928, 17_8630727, 17_8654252, 17_8673449, 17_8712216, 17_8724972, 17_8799118, 17_8842403, 17_8843257, 17_8858083, 17_8889990, 17_8890494, 17_8931866, 17_9027267, 17_9044869, 17_9046655, 17_9056601, 17_9058785, 17_9062537, 17_9111554, 17_9112172, 17_9113244, 17_9140165, 17_9141112, 17_9156631, 17_9159733, 17_9168679, 17_9225536, 17_9226822, 17_9244328, 17_9258987, 17_9273017, 17_9323274, 17_9424427, 17_9424743, 17_9431837, 17_9440188, 17_9455812, 17_9464355, 17_9475849, 17_9476032, 17_9476485, 17_9478949, 17_9495737, 17_9538927, 17_9539688, 17_9605445, 17_9633168, 17_9668117, 17_9744650, 17_9801291, 17_9828121, 17_9871646, 17_9880683, 17_9886256, 17_9936379, 17_10008289, 17_10014410, 17_10016221, 17_10095166, 17_10098534, 17_10118058, 17_10118232, 17_10119824, 17_10120248, 17_10122544, 17_10123517, 17_10137881, 17_10144604, 17_10149306, 17_10175749, 17_10200429, 17_10208625, 17_10229035, 17_10245542, 17_10261471, 17_10264033, 17_10272995, 17_10283059, 17_10300237, 17_10301798, 17_10368918, 17_10377589, 17_10420914, 17_10492607, 17_10493144, 17_10519767, 17_10520203, 17_10642233, 17_10658712, 17_10671392, 17_10688656, 17_10694716, 17_10729686, 17_10730959, 17_10734124, 17_10742897, 17_10765549, 17_10786396, 17_10849306, 17_10852597, 17_10860344, 17_10860732, 17_10869048, 17_10875044, 17_10890182, 17_10917973, 17_10937474, 17_10943762, 17_10949038, 17_10951892, 17_10952628, 17_10966711, 17_11004641, 17_11082365, 17_11097600, 17_11115250, 17_11120815, 17_11133302, 17_11148102, 17_11205535, 17_11206399, 17_11208850, 17_11276626, 17_11276944, 17_11281947, 17_11299367, 17_11310919, 17_11319415, 17_11319951, 17_11324128, 17_11328348, 17_11338261, 17_11349523, 17_11351202, 17_11398710, 17_11408003, 17_11423227, 17_11438128, 17_11446334, 17_11475270, 17_11479072, 17_11482150, 17_11496607, 17_11504273, 17_11508505, 17_11509728, 17_11516572, 17_11545591, 17_11566454, 17_11593537, 17_11604138, 17_11605919, 17_11629193, 17_11677821, 17_11708877, 17_11725201, 17_11726844, 17_11728357, 17_11733857, 17_11737921, 17_11745745, 17_11750236, 17_11761085, 17_11765291, 17_11768465, 17_11789465, 17_11793626, 17_11850478, 17_11852305, 17_11854078, 17_11855662, 17_11861927, 17_11867350, 17_11871226, 17_11873511, 17_11874037, 17_11912659, 17_11920669, 17_12018913, 17_12020581, 17_12062770, 17_12134703, 17_12141688, 17_12175462, 17_12176664, 17_12186575, 17_12219677, 17_12222372, 17_12226414, 17_12230706, 17_12231902, 17_12249081, 17_12281248, 17_12291830, 17_12329372, 17_12343426, 17_12395179, 17_12409029, 17_12424463, 17_12432709, 17_12433081, 17_12454462, 17_12457414, 17_12459452, 17_12472289, 17_12475859, 17_12492609, 17_12493677, 17_12518238, 17_12522190, 17_12526935, 17_12544494, 17_12551316, 17_12551644, 17_12553664, 17_12564572, 17_12594613, 17_12603925, 17_12607878, 17_12617812, 17_12659882, 17_12674176, 17_12675536, 17_12681548, 17_12684681, 17_12690874, 17_12693870, 17_12694812, 17_12709327, 17_12709757, 17_12723285, 17_12736832, 17_12773916, 17_12777543, 17_12786835, 17_12788612, 17_12789604, 17_12804620, 17_12867524, 17_12872878, 17_12888096, 17_12889412, 17_12895024, 17_12898868, 17_12911676, 17_12914631, 17_12929775, 17_12929845, 17_12938041, 17_12970503, 17_12980531, 17_13002840, 17_13017074, 17_13027838, 17_13032146, 17_13045763, 17_13095363, 17_13135351, 17_13151746, 17_13151787, 17_13200317, 17_13289917, 17_13303857, 17_13318002, 17_13330799, 17_13337066, 17_13357349, 17_13374552, 17_13380833, 17_13392268, 17_13402489, 17_13409312, 17_13411624, 17_13422319, 17_13425340, 17_13425574, 17_13437901, 17_13438472, 17_13442095, 17_13455970, 17_13461676, 17_13461966, 17_13501600, 17_13502517, 17_13524089, 17_13543968, 17_13551840, 17_13569118, 17_13574305, 17_13581872, 17_13597406, 17_13619760, 17_13665518, 17_13689968, 17_13699160, 17_13728470, 17_13759581, 17_13762722, 17_13781988, 17_13791913, 17_13807514, 17_13850760, 17_13856598, 17_13876988, 17_13879185, 17_13883423, 17_13883642, 17_13883988, 17_13895170, 17_13898606, 17_13904923, 17_13922841, 17_13924034, 17_13924869, 17_13932091, 17_13940519, 17_13940843, 17_13953512, 17_13967767, 17_13976951, 17_13977012, 17_13978851, 17_14007072, 17_14011099, 17_14039872, 17_14040517, 17_14052495, 17_14057024, 17_14081858, 17_14082104, 17_14096739, 17_14114209, 17_14116479, 17_14143118, 17_14151252, 17_14158807, 17_14160820, 17_14168691, 17_14198343, 17_14217899, 17_14223985, 17_14237834, 17_14243082, 17_14359544, 17_14365130, 17_14450370, 17_14466365, 17_14469353, 17_14473098, 17_14475535, 17_14476766, 17_14501244, 17_14509966, 17_14514291, 17_14517465, 17_14520997, 17_14538457, 17_14547550, 17_14563842, 17_14564062, 17_14599977, 17_14605405, 17_14649942, 17_14650273, 17_14692032, 17_14692165, 17_14696264, 17_14716066, 17_14791003, 17_14815266, 17_14815606, 17_14934362, 17_14935923, 17_14938625, 17_14959060, 17_14997972, 17_15054089, 17_15072540, 17_15108256, 17_15119993, 17_15174475, 17_15174637, 17_15181004, 17_15212330, 17_15234540, 17_15249894, 17_15261072, 17_15264363, 17_15286025, 17_15316773, 17_15355710, 17_15389273, 17_15419069, 17_15423410, 17_15450012, 17_15457480, 17_15459194, 17_15468506, 17_15526876, 17_15536108, 17_15580937, 17_15584586, 17_15585193, 17_15654023, 17_15684790, 17_15702385, 17_15718247, 17_15745917, 17_15754163, 17_15768360, 17_15781341, 17_15788615, 17_15790028, 17_15798562, 17_15816569, 17_15829159, 17_15834158, 17_15893695, 17_15896740, 17_15898827, 17_16010494, 17_16050135, 17_16051706, 17_16084446, 17_16092228, 17_16102546, 17_16156628, 17_16212189, 17_16245360, 17_16271920, 17_16325946, 17_16357776, 17_16358039, 17_16373701, 17_16388070, 17_16389570, 17_16415741, 17_16417505, 17_16467607, 17_16471037, 17_16471178, 17_16471226, 17_16490088, 17_16524732, 17_16526095, 17_16547023, 17_16589935, 17_16620745, 17_16629764, 17_16643381, 17_16665758, 17_16669797, 17_16691669, 17_16707763, 17_16748419, 17_16784048, 17_16801709, 17_16843879, 17_16894666, 17_16997621, 17_17015238, 17_17017597, 17_17023387, 17_17033917, 17_17071570, 17_17080836, 17_17093197, 17_17116362, 17_17152481, 17_17167290, 17_17187516, 17_17191560, 17_17206424, 17_17219395, 17_17228391, 17_17234345, 17_17252370, 17_17257975, 17_17275604, 17_17275819, 17_17314887, 17_17320306, 17_17339549, 17_17341581, 17_17341889, 17_17380553, 17_17396154, 17_17398224, 17_17444690, 17_17448905, 17_17485626, 17_17486377, 17_17489431, 17_17497778, 17_17500828, 17_17501178, 17_17504290, 17_17508499, 17_17511139, 17_17570327, 17_17573963, 17_17580625, 17_17586653, 17_17588302, 17_17596521, 17_17602592, 17_17631020, 17_17631535, 17_17645544, 17_17651662, 17_17688860, 17_17689792, 17_17723415, 17_17731664, 17_17757009, 17_17807487, 17_17875290, 17_17875551, 17_17881350, 17_17903515, 17_17910601, 17_17920937, 17_17934831, 17_17939480, 17_17951021, 17_17951300, 17_17980690, 17_17982923, 17_17989848, 17_18081884, 17_18082176, 17_18116538, 17_18246821, 17_18282348, 17_18302067, 17_18335878, 17_18377795, 17_18408352, 17_18409458, 17_18494407, 17_18496057, 17_18544765, 17_18583252, 17_18649138, 17_18721280, 17_18739479, 17_18773244, 17_18838419, 17_18860551, 17_18955163, 17_19008605, 17_19060089, 17_19136371, 17_19140486, 17_19172570, 17_19220489, 17_19245690, 17_19350483, 17_19353646, 17_19368499, 17_19410213, 17_19412970, 17_19421579, 17_19422310, 17_19469781, 17_19524403, 17_19538069, 17_19554959, 17_19574663, 17_19633316, 17_19718123, 17_19803146, 17_19967749, 17_19981040, 17_19981219, 17_19982485, 17_19982772, 17_19991478, 17_20002550, 17_20038686, 17_20137731, 17_20205037, 17_20222771, 17_20264151, 17_20368608, 17_20390203, 17_20427257, 17_20466648, 17_20490458, 17_20567310, 17_20593560, 17_20645538, 17_20648187, 17_20686109, 17_20744756, 17_20753096, 17_20783479, 17_20787665, 17_20816995, 17_20866186, 17_20876689, 17_20888086, 17_20897213, 17_20898444, 17_20903054, 17_20923604, 17_20955271, 17_20962276, 17_20963427, 17_20974805, 17_21035278, 17_21036650, 17_21098670, 17_21113274, 17_21147630, 17_21204447, 17_21227883, 17_21241338, 17_21266336, 17_21335660, 17_21340134, 17_21348182, 17_21372484, 17_21395669, 17_21396423, 18_1113, 18_131806, 18_161285, 18_191683, 18_194860, 18_200042, 18_217313, 18_235011, 18_235340, 18_295970, 18_320518, 18_407382, 18_410446, 18_428460, 18_469849, 18_476203, 18_480890, 18_510809, 18_668953, 18_686519, 18_706186, 18_751061, 18_751283, 18_767845, 18_813103, 18_813487, 18_902795, 18_910303, 18_959291, 18_963051, 18_974551, 18_1023910, 18_1066303, 18_1093430, 18_1105660, 18_1114725, 18_1115195, 18_1144453, 18_1145374, 18_1171254, 18_1216294, 18_1270573, 18_1280630, 18_1332699, 18_1333222, 18_1353242, 18_1353802, 18_1376319, 18_1384461, 18_1435207, 18_1437122, 18_1484019, 18_1546208, 18_1559160, 18_1594788, 18_1623032, 18_1628806, 18_1644275, 18_1647597, 18_1661077, 18_1674857, 18_1676609, 18_1676650, 18_1686330, 18_1687777, 18_1695343, 18_1738521, 18_1792737, 18_1895237, 18_1909129, 18_1972867, 18_1991776, 18_2142806, 18_2187709, 18_2200153, 18_2204594, 18_2206074, 18_2206771, 18_2208868, 18_2209688, 18_2214626, 18_2238409, 18_2281307, 18_2418558, 18_2445370, 18_2459018, 18_2477606, 18_2490601, 18_2521220, 18_2639725, 18_2650698, 18_2654605, 18_2657663, 18_2694248, 18_2701775, 18_2752222, 18_2753443, 18_2852150, 18_2873504, 18_2892360, 18_2914340, 18_2962175, 18_3072662, 18_3104274, 18_3104438, 18_3145928, 18_3148516, 18_3176696, 18_3179511, 18_3270520, 18_3271022, 18_3282125, 18_3364165, 18_3403401, 18_3453029, 18_3488039, 18_3554246, 18_3596808, 18_3617566, 18_3628914, 18_3631376, 18_3700295, 18_3710565, 18_3713387, 18_3772668, 18_3850771, 18_3854804, 18_3879164, 18_3885706, 18_3897014, 18_3917675, 18_4021369, 18_4057758, 18_4098104, 18_4105724, 18_4123681, 18_4173529, 18_4188750, 18_4207764, 18_4252280, 18_4265809, 18_4382552, 18_4396364, 18_4400397, 18_4426158, 18_4469422, 18_4489198, 18_4658774, 18_4676616, 18_4710027, 18_4750567, 18_4867804, 18_4900439, 18_4954326, 18_4955804, 18_5021017, 18_5021224, 18_5021683, 18_5025464, 18_5043501, 18_5120696, 18_5120964, 18_5133002, 18_5163328, 18_5187001, 18_5187265, 18_5242452, 18_5270859, 18_5289542, 18_5335127, 18_5340947, 18_5353662, 18_5371361, 18_5375187, 18_5379927, 18_5391060, 18_5421585, 18_5429092, 18_5562541, 18_5571140, 18_5600237, 18_5633530, 18_5666657, 18_5688536, 18_5695256, 18_5723376, 18_5728988, 18_5730911, 18_5810958, 18_5844310, 18_5855917, 18_5890635, 18_5896267, 18_5901883, 18_5959034, 18_5961191, 18_5968084, 18_5982303, 18_6023372, 18_6024628, 18_6026399, 18_6035677, 18_6058910, 18_6080088, 18_6088626, 18_6108270, 18_6126848, 18_6166197, 18_6220530, 18_6245562, 18_6248214, 18_6253324, 18_6257051, 18_6265169, 18_6290370, 18_6296485, 18_6301644, 18_6320715, 18_6383732, 18_6385211, 18_6459003, 18_6459225, 18_6469866, 18_6482605, 18_6511010, 18_6526426, 18_6526787, 18_6549661, 18_6558522, 18_6597808, 18_6598434, 18_6655594, 18_6657225, 18_6699199, 18_6711707, 18_6712540, 18_6723938, 18_6728414, 18_6755420, 18_6851409, 18_6899071, 18_6905166, 18_6949934, 18_6965529, 18_6966601, 18_6981407, 18_6990763, 18_7038929, 18_7046496, 18_7068367, 18_7083234, 18_7083548, 18_7087492, 18_7198293, 18_7199917, 18_7208476, 18_7220211, 18_7221246, 18_7226765, 18_7237422, 18_7295720, 18_7301305, 18_7326733, 18_7351904, 18_7417986, 18_7436795, 18_7444713, 18_7491479, 18_7513346, 18_7576221, 18_7582303, 18_7591438, 18_7618439, 18_7618639, 18_7625449, 18_7644343, 18_7644910, 18_7650955, 18_7711451, 18_7724192, 18_7731844, 18_7755554, 18_7819422, 18_7831933, 18_7840782, 18_7865505, 18_7881835, 18_7923040, 18_7945398, 18_7975139, 18_8000249, 18_8002134, 18_8005884, 18_8011322, 18_8021748, 18_8023965, 18_8026673, 18_8031399, 18_8054452, 18_8110362, 18_8145499, 18_8165008, 18_8174640, 18_8196291, 18_8230115, 18_8258435, 18_8264167, 18_8289695, 18_8290120, 18_8294558, 18_8316282, 18_8369968, 18_8370673, 18_8372962, 18_8393824, 18_8394799, 18_8395269, 18_8396560, 18_8398101, 18_8484788, 18_8528224, 18_8532821, 18_8558422, 18_8568910, 18_8570665, 18_8582650, 18_8584583, 18_8587170, 18_8589288, 18_8591096, 18_8596670, 18_8643197, 18_8654581, 18_8661787, 18_8669211, 18_8684222, 18_8690996, 18_8693564, 18_8728668, 18_8730290, 18_8731699, 18_8748871, 18_8760665, 18_8771245, 18_8831454, 18_8836217, 18_8854490, 18_8868469, 18_8873218, 18_8880671, 18_8903548, 18_8912740, 18_8927909, 18_8935386, 18_8967295, 18_8983197, 18_8992485, 18_9017177, 18_9023852, 18_9028794, 18_9033333, 18_9043574, 18_9045967, 18_9050199, 18_9052726, 18_9066576, 18_9086288, 18_9097240, 18_9110330, 18_9125834, 18_9151631, 18_9166683, 18_9174929, 18_9207711, 18_9210977, 18_9211530, 18_9219497, 18_9234095, 18_9272968, 18_9285336, 18_9298814, 18_9331480, 18_9350636, 18_9360920, 18_9378859, 18_9384577, 18_9388751, 18_9398812, 18_9431024, 18_9431343, 18_9431939, 18_9437157, 18_9450048, 18_9457663, 18_9510986, 18_9512328, 18_9563366, 18_9585557, 18_9588180, 18_9606651, 18_9619207, 18_9635932, 18_9640393, 18_9652910, 18_9655695, 18_9658161, 18_9669406, 18_9734292, 18_9775708, 18_9785455, 18_9790906, 18_9795679, 18_9796007, 18_9808690, 18_9831599, 18_9837710, 18_9839837, 18_9870500, 18_9907391, 18_9928640, 18_10000694, 18_10056309, 18_10061770, 18_10077818, 18_10117120, 18_10183420, 18_10214201, 18_10226597, 18_10261734, 18_10262387, 18_10270596, 18_10323386, 18_10334806, 18_10347567, 18_10392263, 18_10469802, 18_10504831, 18_10530974, 18_10546173, 18_10675226, 18_10703825, 18_10713638, 18_10717089, 18_10725333, 18_10731423, 18_10735263, 18_10738197, 18_10742872, 18_10748591, 18_10809752, 18_10819770, 18_10826107, 18_10837197, 18_10877706, 18_10900499, 18_10910280, 18_10917989, 18_10949840, 18_10954125, 18_10987069, 18_11003638, 18_11011314, 18_11027410, 18_11043841, 18_11078597, 18_11085888, 18_11105755, 18_11114498, 18_11163897, 18_11169381, 18_11188512, 18_11229443, 18_11235145, 18_11237040, 18_11299773, 18_11304188, 18_11306662, 18_11320710, 18_11322318, 18_11322723, 18_11326077, 18_11326642, 18_11334392, 18_11359232, 18_11364856, 18_11369310, 18_11397017, 18_11415755, 18_11421817, 18_11431288, 18_11487235, 18_11511699, 18_11522059, 18_11566150, 18_11630766, 18_11636197, 18_11637968, 18_11673895, 18_11711342, 18_11782596, 18_11905574, 18_11958363, 18_11992048, 18_11992323, 18_12015282, 18_12025902, 18_12068392, 18_12135202, 18_12139488, 18_12184204, 18_12205541, 18_12211190, 18_12248529, 18_12306629, 18_12359236, 18_12385539, 18_12386386, 18_12397247, 18_12401875, 18_12412037, 18_12461214, 18_12469323, 18_12471882, 18_12494910, 18_12499581, 18_12528073, 18_12542734, 18_12592632, 18_12598382, 18_12598647, 18_12641446, 18_12644361, 18_12670219, 18_12681721, 18_12703289, 18_12770526, 18_12829260, 18_12883507, 18_12899764, 18_12903104, 18_12928276, 18_12933284, 18_12935935, 18_12975117, 18_12985874, 18_13002347, 18_13020051, 18_13049268, 18_13106281, 18_13136759, 18_13140533, 18_13148642, 18_13154642, 18_13158780, 18_13176416, 18_13176568, 18_13188139, 18_13188372, 18_13195927, 18_13203249, 18_13254995, 18_13278657, 18_13287952, 18_13290779, 18_13295881, 18_13297519, 18_13314409, 18_13349314, 18_13351820, 18_13407005, 18_13420691, 18_13421006, 18_13482970, 18_13502755, 18_13522821, 18_13533437, 18_13579561, 18_13632083, 18_13640043, 18_13650047, 18_13653178, 18_13679112, 18_13695566, 18_13756738, 18_13771893, 18_13776617, 18_13829949, 18_13832763, 18_13861834, 18_13888590, 18_13904722, 18_13911342, 18_13918117, 18_13942616, 18_13951661, 18_13967728, 18_14016223, 18_14031701, 18_14040129, 18_14088741, 18_14094347, 18_14210269, 18_14215824, 18_14242263, 18_14308597, 18_14316964, 18_14327551, 18_14336721, 18_14351190, 18_14375011, 18_14412436, 18_14416701, 18_14447277, 18_14472847, 18_14501147, 18_14535083, 18_14551741, 18_14595798, 18_14633100, 18_14632980, 18_14641830, 18_14693863, 18_14695741, 18_14698452, 18_14710053, 18_14727193, 18_14741793, 18_14752514, 18_14755098, 18_14775584, 18_14783507, 18_14786812, 18_14790567, 18_14795469, 18_14830744, 18_14854966, 18_14855165, 18_14855823, 18_14874286, 18_14894856, 18_14906608, 18_14914976, 18_14921745, 18_14921881, 18_14924699, 18_14960698, 18_14961315, 18_14989567, 18_14999976, 18_15000645, 18_15003903, 18_15023436, 18_15029551, 18_15076459, 18_15121102, 18_15134485, 18_15136817, 18_15169054, 18_15273557, 18_15295494, 18_15305640, 18_15341447, 18_15344469, 18_15345250, 18_15345819, 18_15370160, 18_15382986, 18_15393695, 18_15397992, 18_15415288, 18_15439670, 18_15453297, 18_15453519, 18_15458330, 18_15463726, 18_15493635, 18_15503457, 18_15532982, 18_15536877, 18_15541956, 18_15556163, 18_15590113, 18_15605504, 18_15617858, 18_15628075, 18_15633463, 18_15638699, 18_15648084, 18_15650834, 18_15658575, 18_15665445, 18_15667642, 18_15706125, 18_15708876, 18_15718605, 18_15725098, 18_15764346, 18_15764562, 18_15772668, 18_15774914, 18_15780885, 18_15782059, 18_15795162, 18_15833326, 18_15940836, 18_15947227, 18_15947464, 18_15951621, 18_15957507, 18_15961386, 18_15998510, 18_16005899, 18_16038138, 18_16069052, 18_16069530, 18_16073859, 18_16074271, 18_16076956, 18_16083895, 18_16085156, 18_16117164, 18_16122313, 18_16197090, 18_16207220, 18_16228876, 18_16237896, 18_16255781, 18_16256171, 18_16321988, 18_16357990, 18_16372167, 18_16423739, 18_16449810, 18_16458525, 18_16484110, 18_16488961, 18_16492523, 18_16493910, 18_16502082, 18_16526585, 18_16536025, 18_16547418, 18_16548018, 18_16574579, 18_16631202, 18_16635855, 18_16639208, 18_16642988, 18_16677589, 18_16678989, 18_16736187, 18_16737003, 18_16739090, 18_16761280, 18_16770212, 18_16772266, 18_16772885, 18_16776295, 18_16777973, 18_16855823, 18_16876237, 18_16884919, 18_16887924, 18_16914433, 18_16924399, 18_16930297, 18_16984857, 18_16985541, 18_17006419, 18_17007496, 18_17012728, 18_17041524, 18_17054446, 18_17062395, 18_17089428, 18_17100575, 18_17103924, 18_17109278, 18_17170529, 18_17173636, 18_17176294, 18_17185159, 18_17192003, 18_17193271, 18_17246804, 18_17276039, 18_17281349, 18_17336044, 18_17346640, 18_17346994, 18_17375760, 18_17395574, 18_17412210, 18_17412558, 18_17425525, 18_17458081, 18_17469453, 18_17470251, 18_17489437, 18_17517555, 18_17519776, 18_17547229, 18_17576366, 18_17577513, 18_17578748, 18_17615938, 18_17628459, 18_17632186, 18_17634276, 18_17661200, 18_17715825, 18_17781612, 18_17787608, 18_17794502, 18_17832951, 18_17836560, 18_17841342, 18_17852024, 18_17867541, 18_17894583, 18_17901022, 18_17904955, 18_17906027, 18_17932502, 18_17932621, 18_17935647, 18_17936596, 18_17938357, 18_17941776, 18_17961585, 18_17974872, 18_17977850, 18_17987184, 18_17990551, 18_17991940, 18_18002209, 18_18007204, 18_18025250, 18_18039326, 18_18046641, 18_18058692, 18_18107315, 18_18111035, 18_18113355, 18_18182772, 18_18191482, 18_18210763, 18_18215454, 18_18255391, 18_18256847, 18_18257659, 18_18262719, 18_18312051, 18_18320749, 18_18327710, 18_18332679, 18_18352987, 18_18367350, 18_18368884, 18_18377967, 18_18386857, 18_18386956, 18_18387847, 18_18403033, 18_18404112, 18_18407365, 18_18408099, 18_18414110, 18_18425008, 18_18425122, 18_18425622, 18_18428604, 18_18431559, 18_18448527, 18_18453021, 18_18468970, 18_18491305, 18_18504840, 18_18505005, 18_18505512, 18_18517828, 18_18525268, 18_18543794, 18_18544051, 18_18544909, 18_18564133, 18_18592168, 18_18598678, 18_18601681, 18_18602047, 18_18602954, 18_18622499, 18_18622849, 18_18626689, 18_18652907, 18_18654180, 18_18697358, 18_18710365, 18_18744721, 18_18763190, 18_18768343, 18_18768857, 18_18772238, 18_18782274, 18_18802848, 18_18828727, 18_18835223, 18_18836056, 18_18861579, 18_18869866, 18_18877764, 18_18898682, 18_18925752, 18_18927436, 18_18927827, 18_18939414, 18_18943085, 18_18949100, 18_18967338, 18_18971881, 18_18977480, 18_18985276, 18_19009097, 18_19019697, 18_19050192, 18_19062633, 18_19066660, 18_19073640, 18_19081215, 18_19083988, 18_19116862, 18_19117726, 18_19136693, 18_19144862, 18_19151376, 18_19184511, 18_19211659, 18_19224493, 18_19234313, 18_19258655, 18_19265572, 18_19282658, 18_19362713, 18_19377712, 18_19390113, 18_19390556, 18_19444707, 18_19447856, 18_19448492, 18_19452776, 18_19495044, 18_19501341, 18_19501612, 18_19507902, 18_19528422, 18_19530985, 18_19533615, 18_19536209, 18_19549988, 18_19560026, 18_19569894, 18_19570467, 18_19580915, 18_19581421, 18_19589866, 18_19592238, 18_19602055, 18_19603326, 18_19605144, 18_19624350, 18_19627214, 18_19627424, 18_19658060, 18_19659472, 18_19659764, 18_19668970, 18_19670230, 18_19675896, 18_19687902, 18_19719135, 18_19719906, 18_19737092, 18_19737850, 18_19763656, 18_19775521, 18_19779385, 18_19782779, 18_19804727, 18_19807425, 18_19817712, 18_19824790, 18_19836050, 18_19860224, 18_19892636, 18_19925870, 18_19927014, 18_19941311, 18_19951066, 18_19953675, 18_19962543, 18_19971965, 18_19974550, 18_19992959, 18_19995094, 18_20007424, 18_20027571, 18_20032910, 18_20049247, 18_20051736, 18_20053801, 18_20057930, 18_20061283, 18_20070198, 18_20079074, 18_20093053, 18_20103991, 18_20111624, 18_20114075, 18_20119053, 18_20121623, 18_20131100, 18_20147377, 18_20149323, 18_20160040, 18_20160898, 18_20161260, 18_20165289, 18_20168394, 18_20171813, 18_20172063, 18_20203527, 18_20206778, 18_20209009, 18_20209340, 18_20224098, 18_20249035, 18_20253832, 18_20282892, 18_20284045, 18_20286306, 18_20301260, 18_20304589, 18_20304945, 18_20306550, 18_20313865, 18_20320819, 18_20327405, 18_20330554, 18_20333449, 18_20344444, 18_20348019, 18_20351014, 18_20355908, 18_20357338, 18_20362275, 18_20362797, 18_20365464, 18_20372731, 18_20399942, 18_20402574, 18_20434651, 18_20469457, 18_20518129, 18_20532959, 18_20536913, 18_20538524, 18_20546171, 18_20546368, 18_20552033, 18_20556156, 18_20564260, 18_20579757, 18_20582746, 18_20606599, 18_20677994, 18_20679037, 18_20685583, 18_20689876, 18_20691466, 18_20700492, 18_20725253, 18_20736106, 18_20769982, 18_20776531, 18_20850578, 18_20853274, 18_20869648, 18_20872784, 18_20876846, 18_20902317, 18_20934467, 18_20963174, 18_20966220, 18_20989180, 18_20990411, 18_21008686, 18_21034062, 18_21039254, 18_21059764, 18_21071740, 18_21090214, 18_21116797, 18_21155574, 18_21164039, 18_21172265, 18_21183258, 18_21209740, 18_21234859, 18_21235911, 18_21268247, 18_21269892, 18_21282735, 18_21290416, 18_21307287, 18_21333477, 18_21335271, 19_674, 19_8665, 19_46664, 19_72372, 19_84290, 19_97546, 19_117773, 19_170822, 19_261544, 19_273718, 19_357279, 19_392557, 19_443293, 19_452199, 19_463022, 19_670528, 19_713264, 19_723155, 19_853053, 19_1011086, 19_1086461, 19_1109197, 19_1119866, 19_1161946, 19_1198123, 19_1233138, 19_1272543, 19_1296200, 19_1298218, 19_1304804, 19_1305375, 19_1311312, 19_1315234, 19_1319411, 19_1341997, 19_1361409, 19_1386529, 19_1402853, 19_1429542, 19_1434544, 19_1456807, 19_1475833, 19_1490191, 19_1518213, 19_1565980, 19_1566998, 19_1580933, 19_1591145, 19_1623026, 19_1630342, 19_1651504, 19_1674536, 19_1698961, 19_1896924, 19_1903754, 19_1924870, 19_1933068, 19_1943371, 19_1964259, 19_1986432, 19_1996193, 19_2075414, 19_2121846, 19_2215199, 19_2366799, 19_2373020, 19_2391961, 19_2407724, 19_2481365, 19_2494805, 19_2523964, 19_2547022, 19_2733119, 19_2771877, 19_2787004, 19_2804169, 19_2820305, 19_2823220, 19_2833499, 19_2872229, 19_2922357, 19_2930614, 19_2939321, 19_2950972, 19_2993350, 19_3002309, 19_3005583, 19_3059314, 19_3088466, 19_3089894, 19_3105990, 19_3117276, 19_3122000, 19_3140144, 19_3142025, 19_3161508, 19_3170562, 19_3184152, 19_3212094, 19_3253610, 19_3267052, 19_3272789, 19_3283937, 19_3288730, 19_3323038, 19_3352507, 19_3371622, 19_3388053, 19_3392722, 19_3428680, 19_3442935, 19_3443155, 19_3450575, 19_3485491, 19_3485599, 19_3489033, 19_3492533, 19_3497653, 19_3503759, 19_3519972, 19_3561078, 19_3582069, 19_3695530, 19_3704453, 19_3707076, 19_3735949, 19_3743105, 19_3787790, 19_3811013, 19_3827985, 19_3830464, 19_3856337, 19_3883295, 19_3942029, 19_3970358, 19_3970635, 19_3973788, 19_4025569, 19_4056765, 19_4061044, 19_4075955, 19_4108614, 19_4108948, 19_4110973, 19_4119704, 19_4137312, 19_4144005, 19_4150151, 19_4165974, 19_4176985, 19_4210235, 19_4210556, 19_4223965, 19_4227927, 19_4240784, 19_4252730, 19_4271720, 19_4282149, 19_4299124, 19_4310143, 19_4363044, 19_4364442, 19_4403043, 19_4404567, 19_4419773, 19_4427047, 19_4453004, 19_4504180, 19_4507217, 19_4507644, 19_4512826, 19_4539051, 19_4573613, 19_4598735, 19_4608952, 19_4610321, 19_4690849, 19_4713509, 19_4725329, 19_4795568, 19_4799371, 19_4805532, 19_4805876, 19_4823937, 19_4857184, 19_4899909, 19_4925963, 19_4934929, 19_4936415, 19_4942227, 19_4950923, 19_4987816, 19_5003884, 19_5006297, 19_5007053, 19_5019175, 19_5020048, 19_5056164, 19_5072000, 19_5191449, 19_5212274, 19_5221102, 19_5223578, 19_5243326, 19_5250401, 19_5255826, 19_5260412, 19_5262250, 19_5262833, 19_5267681, 19_5292040, 19_5298302, 19_5306396, 19_5306753, 19_5308261, 19_5310356, 19_5320467, 19_5326675, 19_5333658, 19_5333981, 19_5336931, 19_5337129, 19_5356773, 19_5365250, 19_5367306, 19_5375101, 19_5397209, 19_5422738, 19_5428494, 19_5487663, 19_5489659, 19_5490869, 19_5499108, 19_5514897, 19_5517961, 19_5530509, 19_5535038, 19_5539396, 19_5582217, 19_5585229, 19_5645843, 19_5650959, 19_5659329, 19_5670406, 19_5670943, 19_5678621, 19_5683800, 19_5684544, 19_5745025, 19_5763017, 19_5773831, 19_5785507, 19_5791063, 19_5819218, 19_5855324, 19_5857364, 19_5878043, 19_5899436, 19_5901460, 19_5947840, 19_6041582, 19_6059451, 19_6090653, 19_6144304, 19_6175966, 19_6211844, 19_6224695, 19_6225592, 19_6282845, 19_6286871, 19_6300712, 19_6306543, 19_6332531, 19_6334808, 19_6347210, 19_6359815, 19_6372214, 19_6409943, 19_6412205, 19_6418444, 19_6420901, 19_6429411, 19_6443991, 19_6452747, 19_6467475, 19_6498653, 19_6503439, 19_6558211, 19_6584632, 19_6588622, 19_6602198, 19_6626116, 19_6626249, 19_6632250, 19_6635727, 19_6638103, 19_6642226, 19_6666276, 19_6670302, 19_6681027, 19_6682379, 19_6683230, 19_6702656, 19_6709395, 19_6720834, 19_6721197, 19_6734170, 19_6738394, 19_6738630, 19_6747880, 19_6754772, 19_6756023, 19_6756247, 19_6761402, 19_6769693, 19_6777150, 19_6803806, 19_6819323, 19_6821108, 19_6856434, 19_6896236, 19_6900873, 19_6905288, 19_6950575, 19_6959637, 19_6960883, 19_6963354, 19_7066530, 19_7088798, 19_7100364, 19_7116983, 19_7158719, 19_7174871, 19_7176242, 19_7186103, 19_7276711, 19_7278872, 19_7280016, 19_7300901, 19_7312139, 19_7346543, 19_7386392, 19_7388033, 19_7388218, 19_7412777, 19_7419311, 19_7422467, 19_7432624, 19_7448881, 19_7449770, 19_7486024, 19_7495723, 19_7554853, 19_7565770, 19_7571683, 19_7618654, 19_7726453, 19_7786119, 19_7801897, 19_7812441, 19_7842035, 19_7843743, 19_7864150, 19_7900524, 19_7906659, 19_7907019, 19_7935879, 19_7945209, 19_7968145, 19_7968564, 19_7971303, 19_7983687, 19_8003246, 19_8007720, 19_8027593, 19_8036388, 19_8047031, 19_8066215, 19_8076816, 19_8100243, 19_8162484, 19_8179742, 19_8188093, 19_8191421, 19_8191612, 19_8211241, 19_8211715, 19_8254782, 19_8255267, 19_8272672, 19_8286706, 19_8287017, 19_8333204, 19_8411041, 19_8428061, 19_8442784, 19_8453527, 19_8497152, 19_8545755, 19_8548007, 19_8550099, 19_8550770, 19_8574226, 19_8631961, 19_8687890, 19_8702822, 19_8771644, 19_8791706, 19_8816672, 19_8828425, 19_8844538, 19_8862420, 19_8867627, 19_8901750, 19_8913716, 19_9082663, 19_9090406, 19_9111456, 19_9154660, 19_9304542, 19_9353317, 19_9356383, 19_9364613, 19_9388704, 19_9400223, 19_9402912, 19_9434190, 19_9436540, 19_9455642, 19_9544696, 19_9590965, 19_9602968, 19_9610433, 19_9631290, 19_9659228, 19_9667096, 19_9669393, 19_9678708, 19_9717532, 19_9720314, 19_9729999, 19_9731345, 19_9785803, 19_9786493, 19_9861635, 19_9879143, 19_9902496, 19_9924765, 19_9985181, 19_9987030, 19_10001243, 19_10006740, 19_10009095, 19_10012219, 19_10013902, 19_10040183, 19_10064051, 19_10101267, 19_10101360, 19_10108998, 19_10128794, 19_10147329, 19_10296191, 19_10299359, 19_10323053, 19_10327537, 19_10350184, 19_10374566, 19_10398271, 19_10399586, 19_10400569, 19_10418185, 19_10429519, 19_10439420, 19_10445341, 19_10446602, 19_10486031, 19_10491318, 19_10493031, 19_10502805, 19_10505965, 19_10543025, 19_10566503, 19_10588033, 19_10600072, 19_10610008, 19_10616610, 19_10623650, 19_10677414, 19_10696774, 19_10701073, 19_10732967, 19_10734231, 19_10754588, 19_10848266, 19_10907882, 19_10909540, 19_10996173, 19_11052658, 19_11064855, 19_11083313, 19_11083911, 19_11102567, 19_11108687, 19_11111605, 19_11127128, 19_11131680, 19_11150631, 19_11181746, 19_11225758, 19_11241994, 19_11266793, 19_11335997, 19_11341936, 19_11353865, 19_11383527, 19_11400908, 19_11415348, 19_11497292, 19_11500040, 19_11514029, 19_11521639, 19_11534548, 19_11536547, 19_11544413, 19_11610771, 19_11611164, 19_11613992, 19_11619480, 19_11644633, 19_11647935, 19_11649172, 19_11653440, 19_11666281, 19_11714677, 19_11732578, 19_11782182, 19_11799593, 19_11841163, 19_11862177, 19_11903781, 19_11955803, 19_11973885, 19_11994909, 19_12008032, 19_12067949, 19_12080123, 19_12083546, 19_12083958, 19_12109931, 19_12128349, 19_12130186, 19_12167119, 19_12205512, 19_12249580, 19_12315712, 19_12320556, 19_12365582, 19_12393390, 19_12468779, 19_12469503, 19_12501416, 19_12542731, 19_12554085, 19_12571253, 19_12581724, 19_12587694, 19_12593570, 19_12604300, 19_12614050, 19_12633827, 19_12648748, 19_12667319, 19_12678126, 19_12691438, 19_12698106, 19_12711074, 19_12749934, 19_12753902, 19_12757669, 19_12770228, 19_12789901, 19_12796916, 19_12832730, 19_12839009, 19_12843612, 19_12848672, 19_12850758, 19_12875524, 19_12881312, 19_12890020, 19_12897431, 19_12901271, 19_12905806, 19_12930569, 19_12937409, 19_12940335, 19_12944857, 19_12959298, 19_13014328, 19_13023547, 19_13060605, 19_13081333, 19_13087353, 19_13119422, 19_13127168, 19_13161335, 19_13171657, 19_13182801, 19_13182841, 19_13185770, 19_13197990, 19_13198505, 19_13210941, 19_13221262, 19_13221396, 19_13227104, 19_13257675, 19_13260298, 19_13292960, 19_13309863, 19_13346615, 19_13362908, 19_13380789, 19_13397706, 19_13467309, 19_13486159, 19_13569694, 19_13650850, 19_13669985, 19_13700777, 19_13705798, 19_13712441, 19_13784212, 19_13792543, 19_13799115, 19_13834120, 19_13842806, 19_13852170, 19_13853416, 19_13854650, 19_13876554, 19_13886022, 19_13889232, 19_13895503, 19_13933119, 19_13986961, 19_13994246, 19_14009846, 19_14041129, 19_14054913, 19_14064671, 19_14090223, 19_14097032, 19_14121651, 19_14154219, 19_14161205, 19_14162057, 19_14179783, 19_14182241, 19_14182689, 19_14183522, 19_14188452, 19_14190841, 19_14194150, 19_14207390, 19_14246998, 19_14251864, 19_14252291, 19_14255854, 19_14292689, 19_14318482, 19_14335707, 19_14354208, 19_14410884, 19_14419608, 19_14423644, 19_14427576, 19_14429362, 19_14434180, 19_14434376, 19_14439595, 19_14466767, 19_14469467, 19_14484658, 19_14484820, 19_14487293, 19_14487541, 19_14488668, 19_14492045, 19_14492293, 19_14494859, 19_14502563, 19_14503794, 19_14506358, 19_14506829, 19_14528310, 19_14532511, 19_14541776, 19_14546788, 19_14575085, 19_14617379, 19_14623395, 19_14653365, 19_14663902, 19_14669479, 19_14697909, 19_14704406, 19_14731731, 19_14784589, 19_14787989, 19_14822180, 19_14831812, 19_14842308, 19_14878432, 19_14889858, 19_14942200, 19_14948965, 19_14953047, 19_14953658, 19_14960158, 19_14971461, 19_14972772, 19_14985946, 19_15030528, 19_15032931, 19_15039904, 19_15054531, 19_15064325, 19_15094131, 19_15140820, 19_15142953, 19_15202295, 19_15212196, 19_15223049, 19_15226567, 19_15229839, 19_15232505, 19_15244115, 19_15250477, 19_15250774, 19_15263095, 19_15273813, 19_15309726, 19_15313633, 19_15314575, 19_15346291, 19_15374184, 19_15390201, 19_15408712, 19_15410964, 19_15413987, 19_15427896, 19_15428206, 19_15455813, 19_15456722, 19_15457222, 19_15458748, 19_15462361, 19_15471931, 19_15480262, 19_15539365, 19_15539754, 19_15545218, 19_15573151, 19_15589666, 19_15614528, 19_15633348, 19_15655668, 19_15703414, 19_15708731, 19_15731381, 19_15773683, 19_15780755, 19_15786567, 19_15794939, 19_15812566, 19_15841748, 19_15849620, 19_15926204, 19_15943038, 19_15974209, 19_15975126, 19_15993510, 19_15995534, 19_16032029, 19_16039334, 19_16056998, 19_16057121, 19_16087171, 19_16104350, 19_16156953, 19_16169042, 19_16173532, 19_16211230, 19_16213690, 19_16219294, 19_16239779, 19_16245302, 19_16258538, 19_16283453, 19_16285262, 19_16300807, 19_16303243, 19_16304330, 19_16320485, 19_16355236, 19_16358980, 19_16361946, 19_16403368, 19_16412921, 19_16421122, 19_16436742, 19_16446251, 19_16464698, 19_16507899, 19_16508831, 19_16517286, 19_16525695, 19_16555554, 19_16555708, 19_16563303, 19_16574991, 19_16578623, 19_16583424, 19_16590565, 19_16595402, 19_16624229, 19_16626404, 19_16627799, 19_16646575, 19_16663278, 19_16677690, 19_16686575, 19_16692189, 19_16693268, 19_16739890, 19_16744613, 19_16757085, 19_16759353, 19_16782134, 19_16806034, 19_16810062, 19_16845479, 19_16865639, 19_16886495, 19_16886998, 19_16888056, 19_16891700, 19_16905519, 19_16909717, 19_16924564, 19_16956559, 19_16964417, 19_16967865, 19_16984895, 19_17001605, 19_17013855, 19_17016981, 19_17023199, 19_17039088, 19_17050406, 19_17065799, 19_17105571, 19_17111856, 19_17114803, 19_17115729, 19_17116194, 19_17116664, 19_17126119, 19_17127263, 19_17169283, 19_17195936, 19_17214824, 19_17237188, 19_17311694, 19_17346161, 19_17353937, 19_17387346, 19_17397958, 19_17399261, 19_17399685, 19_17463718, 19_17491602, 19_17515012, 19_17518173, 19_17532865, 19_17548969, 19_17555467, 19_17556462, 19_17561331, 19_17562893, 19_17582868, 19_17592712, 19_17593701, 19_17607683, 19_17698300, 19_17700752, 19_17715000, 19_17734353, 19_17737165, 19_17745675, 19_17747118, 19_17752744, 19_17756096, 19_17762460, 19_17762618, 19_17770623, 19_17808341, 19_17845358, 19_17850739, 19_17856115, 19_17856387, 19_17865189, 19_17865496, 19_17873090, 19_17877268, 19_17885512, 19_17945101, 19_17945322, 19_17982196, 19_17988287, 19_17989988, 19_18027720, 19_18041567, 19_18060432, 19_18071867, 19_18134341, 19_18135540, 19_18143221, 19_18163635, 19_18184107, 19_18203250, 19_18223077, 19_18229506, 19_18229816, 19_18235442, 19_18246721, 19_18246940, 19_18247338, 19_18247845, 19_18261738, 19_18266926, 19_18273390, 19_18385473, 19_18392158, 19_18402271, 19_18407680, 19_18411298, 19_18412794, 19_18416778, 19_18431481, 19_18462330, 19_18490608, 19_18504668, 19_18509675, 19_18516959, 19_18522062, 19_18522410, 19_18529260, 19_18532922, 19_18549023, 19_18559462, 19_18559812, 19_18580649, 19_18581206, 19_18582202, 19_18583044, 19_18583328, 19_18591761, 19_18593591, 19_18621018, 19_18622205, 19_18624851, 19_18626722, 19_18665591, 19_18666561, 19_18716298, 19_18743239, 19_18758593, 19_18779676, 19_18779884, 19_18781106, 19_18785152, 19_18804195, 19_18806433, 19_18807994, 19_18815071, 19_18834426, 19_18842927, 19_18867692, 19_18881499, 19_18887508, 19_18892166, 19_18908174, 19_18923764, 19_18936377, 19_18966887, 19_18970366, 19_19004533, 19_19007464, 19_19050346, 19_19076072, 19_19081346, 19_19119896, 19_19123232, 19_19125306, 19_19130035, 19_19133210, 19_19143868, 19_19203815, 19_19217312, 19_19239881, 19_19244116, 19_19256974, 19_19287905, 19_19299234, 19_19341512, 19_19342238, 19_19346685, 19_19349703, 19_19351755, 19_19375237, 19_19387476, 19_19412540, 19_19421223, 19_19467592, 19_19481594, 19_19487136, 19_19503951, 19_19506195, 19_19512903, 19_19531582, 19_19541378, 19_19561572, 19_19567735, 19_19574850, 19_19602704, 19_19616920, 19_19618540, 19_19641031, 19_19642234, 19_19654114, 19_19682356, 19_19683408, 19_19691774, 19_19698411, 19_19699395, 19_19711368, 19_19731516, 19_19733036, 19_19739109, 19_19740733, 19_19746154, 19_19762737, 19_19765169, 19_19766445, 19_19771754, 19_19777340, 19_19784787, 19_19786971, 19_19788177, 19_19829954, 19_19852563, 19_19853878, 19_19859439, 19_19913093, 19_19916121, 19_19921830, 19_19926812, 19_19927164, 19_19934031, 19_19946111, 19_19971374, 19_19973994, 19_19996484, 19_20006531, 19_20008361, 19_20017098, 19_20025032, 19_20029817, 19_20079129, 19_20094276, 19_20097132, 19_20101150, 19_20134574, 19_20167814, 19_20203616, 19_20239864, 19_20243059, 20_72050, 20_73680, 20_110172, 20_327834, 20_358268, 20_360482, 20_387162, 20_392109, 20_395577, 20_415215, 20_416572, 20_485286, 20_524498, 20_528918, 20_536135, 20_553402, 20_589718, 20_618597, 20_630992, 20_663503, 20_678116, 20_721383, 20_727314, 20_727810, 20_760076, 20_764834, 20_780282, 20_796872, 20_821913, 20_825154, 20_879383, 20_911470, 20_935910, 20_976208, 20_1021240, 20_1046045, 20_1112663, 20_1217354, 20_1272051, 20_1482677, 20_1491712, 20_1493291, 20_1503534, 20_1506514, 20_1518364, 20_1558122, 20_1585878, 20_1604686, 20_1623992, 20_1635517, 20_1648361, 20_1672039, 20_1672335, 20_1676858, 20_1679453, 20_1689430, 20_1698777, 20_1716753, 20_1730336, 20_1832814, 20_1840380, 20_1869643, 20_1895803, 20_1897728, 20_1927449, 20_1955659, 20_1959351, 20_1961166, 20_2048571, 20_2102188, 20_2107575, 20_2119141, 20_2119660, 20_2130954, 20_2132560, 20_2200917, 20_2220440, 20_2237298, 20_2248810, 20_2251168, 20_2300478, 20_2303214, 20_2305906, 20_2313802, 20_2313925, 20_2509599, 20_2512125, 20_2592668, 20_2627739, 20_2680342, 20_2688499, 20_2773720, 20_2828126, 20_2859766, 20_2916116, 20_2924956, 20_2944082, 20_2958998, 20_2990031, 20_3002779, 20_3010620, 20_3023448, 20_3078706, 20_3078972, 20_3079594, 20_3128787, 20_3152107, 20_3168966, 20_3192748, 20_3193221, 20_3215649, 20_3237043, 20_3281131, 20_3307454, 20_3310308, 20_3313619, 20_3319194, 20_3325906, 20_3331031, 20_3341703, 20_3353817, 20_3354199, 20_3356836, 20_3361241, 20_3372584, 20_3379771, 20_3395234, 20_3424007, 20_3439011, 20_3451512, 20_3468552, 20_3479754, 20_3480839, 20_3506847, 20_3511846, 20_3518045, 20_3525380, 20_3530807, 20_3548199, 20_3562833, 20_3574750, 20_3623845, 20_3638893, 20_3640453, 20_3642263, 20_3679919, 20_3699294, 20_3715841, 20_3722097, 20_3784584, 20_3788338, 20_3792397, 20_3794028, 20_3811297, 20_3812897, 20_3829518, 20_3831742, 20_3868390, 20_3881186, 20_3896458, 20_3904717, 20_3962874, 20_3974942, 20_3995120, 20_4001032, 20_4008084, 20_4015192, 20_4061123, 20_4062266, 20_4063309, 20_4127971, 20_4138519, 20_4151526, 20_4174872, 20_4175542, 20_4200423, 20_4209512, 20_4226697, 20_4231877, 20_4246331, 20_4295543, 20_4310885, 20_4329826, 20_4377601, 20_4387482, 20_4390933, 20_4397845, 20_4448249, 20_4451928, 20_4465107, 20_4493405, 20_4507051, 20_4512149, 20_4518832, 20_4546586, 20_4591839, 20_4621474, 20_4659214, 20_4659447, 20_4661619, 20_4664064, 20_4669359, 20_4689351, 20_4702120, 20_4707772, 20_4712888, 20_4730103, 20_4750345, 20_4754221, 20_4768886, 20_4769118, 20_4785180, 20_4834451, 20_4838143, 20_4885983, 20_4893833, 20_4908303, 20_4908510, 20_4914982, 20_4918963, 20_4933065, 20_4946551, 20_4960623, 20_4967314, 20_5005423, 20_5045381, 20_5061865, 20_5068443, 20_5108086, 20_5124752, 20_5134649, 20_5134834, 20_5146305, 20_5154424, 20_5163273, 20_5168647, 20_5169853, 20_5171757, 20_5176825, 20_5177347, 20_5177674, 20_5194406, 20_5194830, 20_5199185, 20_5205484, 20_5212105, 20_5221971, 20_5224950, 20_5236267, 20_5249904, 20_5257857, 20_5289018, 20_5335585, 20_5338969, 20_5348083, 20_5370862, 20_5421731, 20_5422105, 20_5424302, 20_5424886, 20_5429750, 20_5432127, 20_5433451, 20_5468629, 20_5474723, 20_5492668, 20_5497458, 20_5549788, 20_5562562, 20_5563711, 20_5568226, 20_5577675, 20_5593002, 20_5639097, 20_5671869, 20_5674302, 20_5682626, 20_5691609, 20_5692031, 20_5701998, 20_5724071, 20_5741930, 20_5748967, 20_5793925, 20_5799680, 20_5822994, 20_5831984, 20_5856067, 20_5900081, 20_5914669, 20_5937945, 20_5946751, 20_5969549, 20_5999456, 20_6001728, 20_6050676, 20_6053213, 20_6055789, 20_6094216, 20_6122563, 20_6131455, 20_6154592, 20_6181280, 20_6186007, 20_6193844, 20_6248093, 20_6267047, 20_6296477, 20_6312433, 20_6346472, 20_6387242, 20_6396998, 20_6397317, 20_6426300, 20_6436688, 20_6447511, 20_6461177, 20_6488771, 20_6543936, 20_6553626, 20_6554082, 20_6564245, 20_6581948, 20_6583004, 20_6586506, 20_6622411, 20_6638326, 20_6644332, 20_6647479, 20_6651351, 20_6657719, 20_6684740, 20_6761296, 20_6784146, 20_6865923, 20_6870490, 20_6888898, 20_6912336, 20_6913910, 20_6934494, 20_6934731, 20_6949460, 20_6975240, 20_6990637, 20_7025646, 20_7047492, 20_7078372, 20_7092415, 20_7110512, 20_7136731, 20_7172902, 20_7240959, 20_7267152, 20_7306167, 20_7355541, 20_7364282, 20_7366648, 20_7433679, 20_7472712, 20_7490513, 20_7520536, 20_7548703, 20_7584109, 20_7589960, 20_7633023, 20_7678549, 20_7685826, 20_7713951, 20_7761063, 20_7815096, 20_7817723, 20_7851205, 20_7866549, 20_7896185, 20_7902101, 20_7909385, 20_7940084, 20_7953506, 20_7964856, 20_8028917, 20_8048066, 20_8063286, 20_8071309, 20_8090450, 20_8094044, 20_8171745, 20_8296917, 20_8308713, 20_8310254, 20_8328082, 20_8445949, 20_8447503, 20_8523033, 20_8528128, 20_8535075, 20_8539668, 20_8540219, 20_8540430, 20_8560188, 20_8566500, 20_8587317, 20_8642240, 20_8642356, 20_8662939, 20_8691292, 20_8706005, 20_8726042, 20_8784582, 20_8785026, 20_8790407, 20_8817155, 20_8832089, 20_8933779, 20_8991807, 20_8997778, 20_9006599, 20_9016241, 20_9057696, 20_9064169, 20_9078903, 20_9083915, 20_9091816, 20_9218438, 20_9249338, 20_9278114, 20_9278174, 20_9285049, 20_9301615, 20_9391018, 20_9396067, 20_9409112, 20_9436323, 20_9436608, 20_9461686, 20_9465982, 20_9467439, 20_9514437, 20_9543308, 20_9571627, 20_9584792, 20_9592101, 20_9609151, 20_9660862, 20_9706606, 20_9710060, 20_9744401, 20_9795904, 20_9812328, 20_9862688, 20_9882585, 20_9889807, 20_9894649, 20_9897669, 20_9909069, 20_9913267, 20_9928446, 20_9952954, 20_9960271, 20_10019270, 20_10063922, 20_10125643, 20_10150179, 20_10201301, 20_10228622, 20_10233859, 20_10310679, 20_10339950, 20_10426645, 20_10436324, 20_10483983, 20_10499697, 20_10674596, 20_10687309, 20_10778277, 20_10789669, 20_10846514, 20_10884131, 20_10896718, 20_10910155, 20_10910383, 20_10923202, 20_10960897, 20_10965118, 20_10966638, 20_10989333, 20_11006113, 20_11012941, 20_11019277, 20_11045475, 20_11058397, 20_11115701, 20_11119369, 20_11193000, 20_11193417, 20_11276529, 20_11278432, 20_11358403, 20_11443792, 20_11456834, 20_11459765, 20_11463090, 20_11467996, 20_11475180, 20_11475447, 20_11559071, 20_11576536, 20_11607376, 20_11671352, 20_11678531, 20_11681419, 20_11693240, 20_11702259, 20_11720352, 20_11745970, 20_11748914, 20_11749345, 20_11762768, 20_11804618, 20_11808995, 20_11815588, 20_11825437, 20_11827099, 20_11865837, 20_11875645, 20_11938811, 20_11947400, 20_11975361, 20_11983980, 20_12005145, 20_12087451, 20_12176414, 20_12202940, 20_12260563, 20_12273110, 20_12283113, 20_12344331, 20_12366345, 20_12382527, 20_12384315, 20_12427006, 20_12436953, 20_12456278, 20_12479753, 20_12501655, 20_12502083, 20_12510719, 20_12513651, 20_12515329, 20_12555375, 20_12556778, 20_12556924, 20_12570604, 20_12717581, 20_12740297, 20_12745730, 20_12752987, 20_12767006, 20_12804330, 20_12804411, 20_12823276, 20_12846310, 20_12846525, 20_12854745, 20_12861042, 20_12873753, 20_12890397, 20_12934144, 20_12941006, 20_12942273, 20_12963784, 20_12978644, 20_12982025, 20_13007647, 20_13025968, 20_13054327, 20_13064965, 20_13097819, 20_13132559, 20_13135553, 20_13138813, 20_13201047, 20_13208878, 20_13214010, 20_13221272, 20_13229262, 20_13251660, 20_13254472, 20_13267849, 20_13268143, 20_13276818, 20_13277322, 20_13291284, 20_13298057, 20_13369974, 20_13370352, 20_13375691, 20_13383628, 20_13394689, 20_13405727, 20_13446765, 20_13459745, 20_13512973, 20_13522032, 20_13534666, 20_13540177, 20_13564495, 20_13567010, 20_13576723, 20_13586200, 20_13605972, 20_13650206, 20_13658787, 20_13658981, 20_13666369, 20_13671466, 20_13673043, 20_13694022, 20_13721501, 20_13756801, 20_13763001, 20_13763110, 20_13763779, 20_13786058, 20_13793766, 20_13902803, 20_13906746, 20_13933232, 20_13953208, 20_13956695, 20_13957060, 20_13968343, 20_13968804, 20_14001928, 20_14003821, 20_14012851, 20_14018262, 20_14043584, 20_14072482, 20_14088976, 20_14100867, 20_14150977, 20_14165951, 20_14170220, 20_14190151, 20_14199260, 20_14203177, 20_14214586, 20_14216606, 20_14223319, 20_14295912, 20_14318848, 20_14341023, 20_14361343, 20_14366865, 20_14383088, 20_14385894, 20_14420838, 20_14442431, 20_14452534, 20_14483698, 20_14499819, 20_14517572, 20_14518696, 20_14524539, 20_14551940, 20_14575842, 20_14585125, 20_14629120, 20_14645587, 20_14740161, 20_14745978, 20_14753953, 20_14782298, 20_14818100, 20_14841744, 20_14860980, 20_14862257, 20_14868031, 20_14868266, 20_14878934, 20_14893066, 20_14913886, 20_14916300, 20_14931581, 20_14961702, 20_15005960, 20_15041064, 20_15051856, 20_15098241, 20_15098665, 20_15100169, 20_15104083, 20_15120259, 20_15120551, 20_15161298, 20_15199868, 20_15211416, 20_15220018, 20_15220284, 20_15221887, 20_15231019, 20_15246406, 20_15296938, 20_15324089, 20_15400003, 20_15400122, 20_15400455, 20_15405856, 20_15407490, 20_15407836, 20_15431005, 20_15464304, 20_15489191, 20_15493815, 20_15494041, 20_15498177, 20_15504503, 20_15505018, 20_15521650, 20_15526555, 20_15548355, 20_15558633, 20_15571939, 20_15605017, 20_15626866, 20_15667498, 20_15670783, 20_15672229, 20_15686312, 20_15687327, 20_15689924, 20_15710218, 20_15724094, 20_15729267, 20_15733154, 20_15754147, 20_15754976, 20_15781438, 20_15811947, 20_15823378, 20_15823564, 20_15833739, 20_15841010, 20_15852137, 20_15864596, 20_15888954, 20_15901369, 20_15901951, 20_15918705, 20_15919295, 20_15920827, 20_15965590, 20_15981405, 20_15993545, 20_16020241, 20_16039422, 20_16044316, 20_16055199, 20_16083237, 20_16083444, 20_16100981, 20_16135934, 20_16161790, 20_16162684, 20_16165214, 20_16186377, 20_16216191, 20_16216832, 20_16224730, 20_16242890, 20_16251680, 20_16255063, 20_16287910, 20_16307276, 20_16312499, 20_16315886, 20_16317958, 20_16354607, 20_16372728, 20_16378539, 20_16378836, 20_16385338, 20_16391432, 20_16396955, 20_16414001, 20_16426327, 20_16428003, 20_16428360, 20_16432619, 20_16441462, 20_16454886, 20_16460154, 20_16461500, 20_16471779, 20_16479087, 20_16502274, 20_16503829, 20_16507523, 20_16515786, 20_16536440, 20_16575348, 20_16584529, 20_16591881, 20_16621621, 20_16621976, 20_16632464, 20_16633317, 20_16635234, 20_16641381, 20_16647277, 20_16658802, 20_16660082, 20_16662223, 20_16683339, 20_16693049, 20_16714443, 20_16764311, 20_16770312, 20_16770886, 20_16776361, 20_16821691, 20_16838163, 20_16860896, 20_16860966, 20_16888135, 20_16913367, 20_16922408, 20_16955726, 20_16981726, 20_17039130, 20_17039459, 20_17064196, 20_17075174, 20_17076764, 20_17084931, 20_17086378, 20_17119956, 20_17160125, 20_17168609, 20_17218661, 20_17223424, 20_17233331, 20_17285043, 20_17285951, 20_17317934, 20_17318431, 20_17325340, 20_17330575, 20_17331512, 20_17340069, 20_17351194, 20_17422248, 20_17442692, 20_17455031, 20_17472204, 20_17484256, 20_17505631, 20_17508860, 20_17511205, 20_17528062, 20_17532986, 20_17543851, 20_17560363, 20_17562380, 20_17568494, 20_17575145, 20_17576763, 20_17596536, 20_17612752, 20_17656350, 20_17657111, 20_17677530, 20_17708547, 20_17726120, 20_17785031, 20_17787272, 20_17795388, 20_17836727, 20_17863960, 20_17901929, 20_17934021, 20_17942283, 20_17950886, 20_17962964, 20_17971999, 20_17980541, 20_17986843, 20_17987075, 20_18000548, 20_18003433, 20_18013250, 20_18032888, 20_18052546, 20_18075186, 20_18149800, 20_18168489, 20_18168982, 20_18169206, 20_18182141, 20_18194408, 20_18206569, 20_18218981, 20_18246342, 20_18256709, 20_18276965, 20_18281382, 20_18292514, 20_18294611, 20_18304911, 20_18323140, 20_18357301, 20_18360292, 20_18372766, 20_18451134, 20_18461327, 20_18463321, 20_18493089, 20_18524331, 20_18528709, 20_18572355, 20_18586305, 20_18586638, 20_18598775, 20_18618361, 20_18620331, 20_18622828, 20_18623905, 20_18661275, 20_18677662, 20_18677908, 20_18678809, 20_18711592, 20_18722641, 20_18760292, 20_18768081, 20_18775217, 20_18787670, 20_18806077, 20_18810871, 20_18833675, 20_18834047, 20_18870499, 20_18872149, 20_18896758, 20_18954474, 20_18980021, 20_18984273, 20_19014992, 20_19026660, 20_19033138, 20_19038258, 20_19041250, 20_19058692, 20_19066180, 20_19085841, 20_19090021, 20_19096933, 20_19098864, 20_19108884, 20_19109930, 20_19118513, 20_19136734, 20_19145767, 20_19155096, 20_19158308, 20_19161178, 20_19161537, 20_19192894, 20_19199884, 20_19207778, 20_19222285, 20_19248562, 20_19310143, 20_19331343, 20_19348030, 20_19354097, 20_19366337, 20_19391442, 20_19431974, 20_19456379, 20_19459160, 20_19483687, 20_19491242, 20_19550545, 20_19553024, 20_19565817, 20_19573683, 20_19586556, 20_19596473, 20_19608128, 20_19619449, 20_19643696, 20_19649189, 20_19649453, 20_19650799, 20_19659287, 20_19696889, 20_19705567, 20_19709207, 20_19725100, 20_19769023, 20_19769280, 20_19783193, 20_19798457, 20_19802014, 20_19802877, 20_19828706, 20_19859667, 20_19874687, 20_19880841, 20_19886731, 20_19887109, 20_19891890, 20_19898444, 20_19899554, 21_44925, 21_105346, 21_113797, 21_173473, 21_194470, 21_194906, 21_195356, 21_315997, 21_333229, 21_346696, 21_350759, 21_566020, 21_574157, 21_597909, 21_607987, 21_659954, 21_669188, 21_863922, 21_865801, 21_866522, 21_919108, 21_983369, 21_1027717, 21_1065332, 21_1068270, 21_1083461, 21_1085402, 21_1096465, 21_1110900, 21_1113400, 21_1154438, 21_1159057, 21_1203984, 21_1225448, 21_1237863, 21_1238358, 21_1290933, 21_1330811, 21_1336397, 21_1337157, 21_1337352, 21_1344230, 21_1367645, 21_1404663, 21_1444633, 21_1492291, 21_1514964, 21_1525652, 21_1537425, 21_1547775, 21_1643654, 21_1645391, 21_1652626, 21_1655388, 21_1658534, 21_1666844, 21_1670550, 21_1711192, 21_1720330, 21_1744804, 21_1763023, 21_1770638, 21_1788163, 21_1828188, 21_1844257, 21_1867936, 21_1873811, 21_1877254, 21_1892130, 21_1940414, 21_1946474, 21_1957969, 21_1993635, 21_2014761, 21_2015423, 21_2015660, 21_2025417, 21_2049974, 21_2063013, 21_2063361, 21_2093536, 21_2101184, 21_2131818, 21_2138437, 21_2141609, 21_2169022, 21_2175075, 21_2192564, 21_2197567, 21_2197835, 21_2199428, 21_2205050, 21_2211313, 21_2234042, 21_2235002, 21_2256439, 21_2262542, 21_2265518, 21_2266261, 21_2279704, 21_2297130, 21_2343009, 21_2345624, 21_2351630, 21_2366178, 21_2381158, 21_2383826, 21_2384005, 21_2424987, 21_2431161, 21_2503581, 21_2518570, 21_2526691, 21_2538787, 21_2540659, 21_2550504, 21_2550950, 21_2552915, 21_2603741, 21_2606882, 21_2614753, 21_2620568, 21_2635311, 21_2673272, 21_2683114, 21_2683912, 21_2694690, 21_2722520, 21_2738970, 21_2769735, 21_2810701, 21_2811252, 21_2814674, 21_2834127, 21_2860842, 21_2868110, 21_2880181, 21_2892081, 21_2893751, 21_2935876, 21_2967911, 21_2970199, 21_2994521, 21_2998082, 21_3002959, 21_3024191, 21_3042988, 21_3043329, 21_3066249, 21_3079230, 21_3114654, 21_3117827, 21_3139703, 21_3169947, 21_3180473, 21_3227529, 21_3246085, 21_3250264, 21_3271933, 21_3293093, 21_3294993, 21_3413316, 21_3434789, 21_3441718, 21_3450864, 21_3462154, 21_3471387, 21_3473388, 21_3486917, 21_3489375, 21_3527803, 21_3543387, 21_3570586, 21_3579960, 21_3611282, 21_3617683, 21_3627252, 21_3634890, 21_3639585, 21_3641160, 21_3647863, 21_3677130, 21_3708279, 21_3717672, 21_3735348, 21_3738178, 21_3749001, 21_3770436, 21_3783013, 21_3830967, 21_3849407, 21_3865408, 21_3880806, 21_3903990, 21_3907101, 21_3933160, 21_3934707, 21_3939641, 21_3983641, 21_3992882, 21_3999386, 21_4030211, 21_4038925, 21_4073540, 21_4080060, 21_4080069, 21_4089540, 21_4091488, 21_4091707, 21_4092310, 21_4137578, 21_4146854, 21_4154355, 21_4168086, 21_4211091, 21_4221346, 21_4263612, 21_4324691, 21_4344237, 21_4413926, 21_4475531, 21_4502656, 21_4521433, 21_4547175, 21_4552854, 21_4574063, 21_4652372, 21_4655320, 21_4666574, 21_4666945, 21_4713857, 21_4727917, 21_4735935, 21_4771374, 21_4807428, 21_4824939, 21_4831558, 21_4844496, 21_4846465, 21_4846909, 21_4860217, 21_4871706, 21_4891527, 21_4900376, 21_4913871, 21_4927660, 21_4930800, 21_4940489, 21_4975248, 21_4994501, 21_5013278, 21_5016486, 21_5032716, 21_5038447, 21_5051308, 21_5116402, 21_5118815, 21_5134576, 21_5138907, 21_5142439, 21_5144622, 21_5196795, 21_5224461, 21_5244621, 21_5288327, 21_5342889, 21_5440714, 21_5444809, 21_5579621, 21_5665492, 21_5672679, 21_5689087, 21_5694168, 21_5714862, 21_5717127, 21_5734905, 21_5777034, 21_5808159, 21_5832547, 21_5941260, 21_5976909, 21_6024419, 21_6024796, 21_6198083, 21_6198860, 21_6253179, 21_6316526, 21_6356318, 21_6356469, 21_6412422, 21_6417762, 21_6423458, 21_6440523, 21_6465449, 21_6554275, 21_6589704, 21_6772458, 21_6795510, 21_6808061, 21_6839076, 21_6889902, 21_6904687, 21_6978647, 21_6979438, 21_7014452, 21_7056526, 21_7057509, 21_7060209, 21_7067948, 21_7099832, 21_7101526, 21_7115141, 21_7124205, 21_7127790, 21_7139265, 21_7155447, 21_7181423, 21_7217382, 21_7228941, 21_7234644, 21_7242071, 21_7244312, 21_7254019, 21_7256277, 21_7285687, 21_7287716, 21_7294698, 21_7301346, 21_7308499, 21_7323466, 21_7326299, 21_7329063, 21_7350046, 21_7385105, 21_7386207, 21_7394478, 21_7394666, 21_7414867, 21_7421086, 21_7435733, 21_7449744, 21_7459993, 21_7477971, 21_7481137, 21_7496381, 21_7508366, 21_7517252, 21_7519994, 21_7526465, 21_7531051, 21_7534006, 21_7544786, 21_7558140, 21_7573944, 21_7586471, 21_7614062, 21_7637081, 21_7664006, 21_7681854, 21_7696267, 21_7723521, 21_7725302, 21_7877474, 21_7885939, 21_7984990, 21_8015029, 21_8044748, 21_8082713, 21_8117649, 21_8283125, 21_8283362, 21_8314964, 21_8335767, 21_8337679, 21_8365319, 21_8399007, 21_8425223, 21_8451519, 21_8507986, 21_8525053, 21_8525314, 21_8562743, 21_8564102, 21_8589165, 21_8589634, 21_8604204, 21_8613283, 21_8617096, 21_8621211, 21_8622491, 21_8633850, 21_8634040, 21_8659094, 21_8671913, 21_8691024, 21_8692685, 21_8696948, 21_8700051, 21_8702487, 21_8705592, 21_8705958, 21_8707985, 21_8804206, 21_8828041, 21_8829787, 21_8855406, 21_8908967, 21_8909680, 21_8920976, 21_8933449, 21_8950398, 21_8973145, 21_8991272, 21_9000115, 21_9025115, 21_9044340, 21_9052906, 21_9095317, 21_9109782, 21_9126604, 21_9168275, 21_9168525, 21_9179593, 21_9301004, 21_9311152, 21_9312175, 21_9349268, 21_9370608, 21_9464515, 21_9484241, 21_9506637, 21_9510547, 21_9524237, 21_9555384, 21_9558000, 21_9559961, 21_9565704, 21_9591563, 21_9615455, 21_9634504, 21_9637586, 21_9659251, 21_9680751, 21_9687705, 21_9705669, 21_9707614, 21_9721016, 21_9726674, 21_9808178, 21_9819843, 21_9822275, 21_9832884, 21_9833375, 21_9856426, 21_9860185, 21_9860509, 21_9889437, 21_9923988, 21_9942882, 21_9972868, 21_9984799, 21_9997154, 21_10003868, 21_10018108, 21_10038495, 21_10039006, 21_10050278, 21_10061724, 21_10078071, 21_10079971, 21_10088162, 21_10110790, 21_10116759, 21_10118526, 21_10139642, 21_10184664, 21_10220699, 21_10224830, 21_10228111, 21_10231317, 21_10245981, 21_10252345, 21_10288533, 21_10350880, 21_10359672, 21_10385039, 21_10400980, 21_10460208, 21_10474856, 21_10477880, 21_10507659, 21_10531665, 21_10559963, 21_10599195, 21_10599455, 21_10603124, 21_10614093, 21_10628671, 21_10628969, 21_10648275, 21_10654908, 21_10662715, 21_10675663, 21_10711772, 21_10780059, 21_10801969, 21_10813312, 21_10832280, 21_10843499, 21_10863287, 21_10864657, 21_10878394, 21_10890566, 21_10912387, 21_10931684, 21_10935389, 21_10968511, 21_11000496, 21_11016066, 21_11021589, 21_11023453, 21_11042995, 21_11045865, 21_11053914, 21_11059317, 21_11109791, 21_11123130, 21_11127455, 21_11146517, 21_11169801, 21_11195778, 21_11211238, 21_11212771, 21_11221253, 21_11231064, 21_11238028, 21_11245289, 21_11250784, 21_11251035, 21_11260234, 21_11282696, 21_11313765, 21_11323826, 21_11330232, 21_11338910, 21_11353812, 21_11361847, 21_11371632, 21_11371977, 21_11372077, 21_11380532, 21_11393603, 21_11397273, 21_11398170, 21_11422105, 21_11434504, 21_11446436, 21_11481925, 21_11495880, 21_11531858, 21_11565021, 21_11577388, 21_11606204, 21_11634847, 21_11643085, 21_11657202, 21_11668699, 21_11692560, 21_11694338, 21_11700303, 21_11701926, 21_11714913, 21_11726617, 21_11773378, 21_11786697, 21_11797806, 21_11829226, 21_11878967, 21_11880503, 21_11887834, 21_11894866, 21_11901984, 21_11909129, 21_11924191, 21_11925588, 21_11936447, 21_11940200, 21_11958152, 21_11963286, 21_11964065, 21_11983393, 21_11986033, 21_12030338, 21_12051701, 21_12053393, 21_12063688, 21_12077910, 21_12079236, 21_12079675, 21_12086590, 21_12122005, 21_12134229, 21_12158058, 21_12179605, 21_12184405, 21_12189647, 21_12190361, 21_12193965, 21_12196803, 21_12231847, 21_12265779, 21_12274277, 21_12279305, 21_12282791, 21_12294454, 21_12311550, 21_12357972, 21_12360909, 21_12377704, 21_12380985, 21_12382341, 21_12391121, 21_12392985, 21_12398905, 21_12404674, 21_12412343, 21_12427605, 21_12428088, 21_12432459, 21_12470505, 21_12480429, 21_12488355, 21_12504950, 21_12518635, 21_12535412, 21_12544027, 21_12548837, 21_12550492, 21_12559005, 21_12569940, 21_12574608, 21_12622475, 21_12622820, 21_12630667, 21_12646628, 21_12662442, 21_12679654, 21_12721268, 21_12722540, 21_12736705, 21_12777976, 21_12808314, 21_12840198, 21_12841145, 21_12848341, 21_12862374, 21_12872818, 21_12882075, 21_12882468, 21_12890091, 21_12898979, 21_12914737, 21_12930263, 21_12940326, 21_12974349, 21_12977679, 21_12997803, 21_13001294, 21_13024326, 21_13034024, 21_13053524, 21_13063071, 21_13082798, 21_13095732, 21_13097317, 21_13143662, 21_13170000, 21_13191659, 21_13193043, 21_13193493, 21_13200379, 21_13238337, 21_13242098, 21_13247061, 21_13259532, 21_13283324, 21_13313360, 21_13316313, 21_13319485, 21_13320646, 21_13324110, 21_13330295, 21_13336681, 21_13349421, 21_13365392, 21_13366252, 21_13379389, 21_13437609, 21_13450965, 21_13462152, 21_13464271, 21_13505969, 21_13517011, 21_13526861, 21_13550080, 21_13573461, 21_13576011, 21_13622757, 21_13623053, 21_13636242, 21_13638633, 21_13659532, 21_13661906, 21_13662266, 21_13760202, 21_13784423, 21_13785216, 21_13892205, 21_13981252, 21_13986430, 21_13994178, 21_14028363, 21_14029826, 21_14032127, 21_14063648, 21_14073705, 21_14076091, 21_14078142, 21_14086644, 21_14113327, 21_14117166, 21_14132745, 21_14143096, 21_14145708, 21_14157533, 21_14171603, 21_14189258, 21_14195474, 21_14197069, 21_14206018, 21_14207423, 21_14208094, 21_14236415, 21_14237539, 21_14245856, 21_14262981, 21_14266646, 21_14267026, 21_14274018, 21_14284546, 21_14306913, 21_14331273, 21_14331691, 21_14341297, 21_14342883, 21_14349423, 21_14397802, 21_14399094, 21_14414970, 21_14470930, 21_14533320, 21_14535343, 21_14550979, 21_14551182, 21_14574012, 21_14605882, 21_14607645, 21_14614516, 21_14650054, 21_14651965, 21_14652312, 21_14658053, 21_14671362, 21_14671914, 21_14675452, 21_14683732, 21_14694810, 21_14711468, 21_14736082, 21_14737503, 21_14753675, 21_14759997, 21_14767485, 21_14789972, 21_14791549, 21_14807964, 21_14821769, 21_14840937, 21_14841684, 21_14853145, 21_14874319, 21_14887635, 21_14897668, 21_14897843, 21_14903202, 21_14903596, 21_14905907, 21_14955369, 21_14961226, 21_14961524, 21_14977690, 21_14990219, 21_14993884, 21_15005018, 21_15006446, 21_15042274, 21_15064364, 21_15064878, 21_15070055, 21_15072425, 21_15096819, 21_15110080, 21_15113378, 21_15140045, 21_15160296, 21_15169740, 21_15231535, 21_15233084, 21_15256450, 21_15319059, 21_15351424, 21_15389221, 21_15394155, 21_15401402, 21_15425907, 21_15447743, 21_15456025, 21_15546351, 21_15559578, 21_15573740, 21_15575732, 21_15576034, 21_15588317, 21_15597095, 21_15598405, 21_15605272, 21_15622282, 21_15623844, 21_15635674, 21_15640225, 21_15688175, 21_15703293, 21_15710642, 21_15761217, 21_15768938, 21_15792390, 21_15800502, 21_15801443, 21_15807637, 21_15816921, 21_15827795, 21_15829717, 21_15873268, 22_35242, 22_38903, 22_76735, 22_76919, 22_115207, 22_126045, 22_126868, 22_141195, 22_149068, 22_160817, 22_165282, 22_177734, 22_210121, 22_215265, 22_222737, 22_224009, 22_259152, 22_263543, 22_293930, 22_349578, 22_373681, 22_404919, 22_408787, 22_419753, 22_428755, 22_454656, 22_464516, 22_468572, 22_483232, 22_499180, 22_502574, 22_507144, 22_517701, 22_519230, 22_548705, 22_581169, 22_594297, 22_596786, 22_609918, 22_623451, 22_648282, 22_650435, 22_664910, 22_673596, 22_686521, 22_693795, 22_694548, 22_702208, 22_708612, 22_729019, 22_744213, 22_745683, 22_746076, 22_749114, 22_790082, 22_795317, 22_822317, 22_827338, 22_869766, 22_870035, 22_878946, 22_884480, 22_885008, 22_935860, 22_945977, 22_983258, 22_1022202, 22_1022676, 22_1053945, 22_1066850, 22_1082207, 22_1091548, 22_1092233, 22_1104464, 22_1117515, 22_1154426, 22_1159763, 22_1185804, 22_1187980, 22_1189234, 22_1189995, 22_1191087, 22_1213066, 22_1221273, 22_1230696, 22_1238242, 22_1241871, 22_1244010, 22_1251509, 22_1256086, 22_1300738, 22_1306442, 22_1310785, 22_1321587, 22_1342086, 22_1345111, 22_1414403, 22_1420853, 22_1421280, 22_1425704, 22_1426481, 22_1430271, 22_1433330, 22_1460482, 22_1467281, 22_1468454, 22_1469770, 22_1494677, 22_1534551, 22_1553068, 22_1563322, 22_1568608, 22_1592783, 22_1600000, 22_1607748, 22_1609440, 22_1627138, 22_1656499, 22_1685549, 22_1688091, 22_1691701, 22_1694314, 22_1717335, 22_1722815, 22_1774427, 22_1795709, 22_1817834, 22_1836393, 22_1839486, 22_1842288, 22_1858018, 22_1886198, 22_1886735, 22_1897723, 22_1901693, 22_1947907, 22_1952201, 22_1952648, 22_1968847, 22_1969219, 22_1984409, 22_1988409, 22_1995896, 22_2018285, 22_2028868, 22_2032721, 22_2057052, 22_2068329, 22_2091733, 22_2122974, 22_2127108, 22_2134748, 22_2142506, 22_2156956, 22_2173055, 22_2175411, 22_2186937, 22_2189468, 22_2211488, 22_2236844, 22_2241539, 22_2241651, 22_2242223, 22_2249830, 22_2262240, 22_2263279, 22_2279612, 22_2285457, 22_2292232, 22_2294977, 22_2296382, 22_2297205, 22_2299076, 22_2309298, 22_2320998, 22_2329773, 22_2335319, 22_2343534, 22_2344289, 22_2355258, 22_2369497, 22_2375031, 22_2380352, 22_2401090, 22_2405824, 22_2412089, 22_2412752, 22_2467601, 22_2505380, 22_2512417, 22_2512835, 22_2519920, 22_2520209, 22_2569121, 22_2586156, 22_2591098, 22_2602393, 22_2613951, 22_2614298, 22_2659227, 22_2684897, 22_2747288, 22_2772767, 22_2781978, 22_2821881, 22_2829230, 22_2833972, 22_2849097, 22_2851202, 22_2857582, 22_2861756, 22_2874661, 22_2887142, 22_2893382, 22_2901408, 22_2904698, 22_2905090, 22_2908981, 22_2959002, 22_2976170, 22_2986499, 22_2990878, 22_3006429, 22_3043036, 22_3051544, 22_3070163, 22_3073870, 22_3078513, 22_3082868, 22_3133197, 22_3137154, 22_3137524, 22_3151217, 22_3151925, 22_3161036, 22_3161217, 22_3163628, 22_3173041, 22_3220129, 22_3222469, 22_3242423, 22_3259417, 22_3262838, 22_3288603, 22_3298015, 22_3321670, 22_3331550, 22_3332151, 22_3343493, 22_3350704, 22_3368022, 22_3370669, 22_3385877, 22_3389132, 22_3391860, 22_3397660, 22_3414854, 22_3424131, 22_3433283, 22_3435197, 22_3441090, 22_3443294, 22_3445293, 22_3448383, 22_3452310, 22_3464590, 22_3491399, 22_3492475, 22_3513324, 22_3518562, 22_3550304, 22_3551819, 22_3560960, 22_3580805, 22_3581247, 22_3607905, 22_3614825, 22_3644537, 22_3649639, 22_3667785, 22_3671053, 22_3690850, 22_3699304, 22_3739433, 22_3742240, 22_3782360, 22_3803297, 22_3824333, 22_3830228, 22_3836158, 22_3848667, 22_3867223, 22_3872503, 22_3909236, 22_3955469, 22_3984955, 22_3992439, 22_4002560, 22_4021702, 22_4071633, 22_4149116, 22_4161703, 22_4166631, 22_4181525, 22_4202056, 22_4246801, 22_4248926, 22_4288608, 22_4294763, 22_4319814, 22_4337674, 22_4351469, 22_4378578, 22_4380918, 22_4413623, 22_4413878, 22_4414103, 22_4456264, 22_4459705, 22_4482316, 22_4498564, 22_4509117, 22_4516407, 22_4524345, 22_4524581, 22_4575828, 22_4583851, 22_4589371, 22_4627628, 22_4666266, 22_4666442, 22_4673524, 22_4683418, 22_4699428, 22_4703034, 22_4735801, 22_4754487, 22_4759747, 22_4858790, 22_4884538, 22_4887512, 22_4910769, 22_4915824, 22_4956802, 22_4958974, 22_4980010, 22_5012508, 22_5028887, 22_5080138, 22_5105334, 22_5114697, 22_5126436, 22_5136437, 22_5146230, 22_5147258, 22_5156468, 22_5163239, 22_5173309, 22_5182006, 22_5189469, 22_5223551, 22_5237672, 22_5237999, 22_5241464, 22_5257059, 22_5280249, 22_5360245, 22_5362650, 22_5369793, 22_5379010, 22_5383257, 22_5384423, 22_5384710, 22_5385462, 22_5387481, 22_5387691, 22_5415480, 22_5441754, 22_5442524, 22_5442961, 22_5461828, 22_5482730, 22_5495150, 22_5530389, 22_5548499, 22_5549363, 22_5569153, 22_5572014, 22_5576499, 22_5613713, 22_5624793, 22_5650985, 22_5655032, 22_5657377, 22_5661658, 22_5686566, 22_5698387, 22_5751522, 22_5782797, 22_5785169, 22_5797445, 22_5798708, 22_5816630, 22_5819118, 22_5828161, 22_5828451, 22_5844672, 22_5845434, 22_5860163, 22_5882079, 22_5883351, 22_5909923, 22_5930169, 22_5932739, 22_5950332, 22_5959726, 22_5963952, 22_5965566, 22_5967114, 22_5978904, 22_5994815, 22_6000179, 22_6004932, 22_6010285, 22_6027062, 22_6062797, 22_6065239, 22_6066376, 22_6096204, 22_6116068, 22_6140103, 22_6144560, 22_6169779, 22_6177181, 22_6184346, 22_6187642, 22_6195517, 22_6202912, 22_6215297, 22_6218355, 22_6221480, 22_6235272, 22_6235552, 22_6242732, 22_6255586, 22_6255841, 22_6265708, 22_6276458, 22_6309909, 22_6376555, 22_6400010, 22_6400137, 22_6422587, 22_6431478, 22_6439947, 22_6441770, 22_6457791, 22_6464927, 22_6483287, 22_6531439, 22_6533128, 22_6562027, 22_6584425, 22_6820576, 22_6828415, 22_6844739, 22_6856166, 22_6863686, 22_6925945, 22_7033859, 22_7105794, 22_7117145, 22_7253082, 22_7271285, 22_7346115, 22_7399209, 22_7433950, 22_7504047, 22_7543547, 22_7551426, 22_7636031, 22_7677343, 22_7685965, 22_7687332, 22_7718815, 22_7731990, 22_7767196, 22_7775047, 22_7796318, 22_7812149, 22_7845191, 22_7845629, 22_7870190, 22_7886979, 22_7900116, 22_7900411, 22_7906419, 22_7947970, 22_7984815, 22_8000709, 22_8023842, 22_8117758, 22_8130563, 22_8182981, 22_8183229, 22_8185733, 22_8187726, 22_8224585, 22_8229125, 22_8298514, 22_8314234, 22_8327591, 22_8327981, 22_8330748, 22_8333563, 22_8338344, 22_8352776, 22_8416176, 22_8438713, 22_8571836, 22_8586541, 22_8632691, 22_8634537, 22_8650540, 22_8658323, 22_8660419, 22_8668180, 22_8725319, 22_8797915, 22_8877433, 22_8877785, 22_8879937, 22_8905751, 22_8906863, 22_8936689, 22_8952529, 22_8959988, 22_8975672, 22_8995480, 22_9006803, 22_9009187, 22_9024424, 22_9028999, 22_9079949, 22_9133230, 22_9209429, 22_9210460, 22_9236320, 22_9244170, 22_9244417, 22_9250869, 22_9262459, 22_9298940, 22_9299371, 22_9310411, 22_9316774, 22_9326718, 22_9355548, 22_9363570, 22_9397100, 22_9403956, 22_9404025, 22_9406558, 22_9428312, 22_9465941, 22_9482920, 22_9491378, 22_9523085, 22_9527624, 22_9527997, 22_9539810, 22_9556078, 22_9559201, 22_9573562, 22_9596251, 22_9598407, 22_9648858, 22_9653820, 22_9661379, 22_9712071, 22_9719370, 22_9747217, 22_9753150, 22_9777010, 22_9777522, 22_9785302, 22_9842874, 22_9874880, 22_9875660, 22_9895927, 22_9943404, 22_9967944, 22_9974993, 22_10000341, 22_10028449, 22_10064525, 22_10072585, 22_10081358, 22_10089367, 22_10125525, 22_10173451, 22_10234695, 22_10250562, 22_10270476, 22_10283784, 22_10321403, 22_10342093, 22_10372859, 22_10382682, 22_10384238, 22_10388268, 22_10398962, 22_10411330, 22_10412717, 22_10422518, 22_10426849, 22_10461680, 22_10501172, 22_10502170, 22_10540183, 22_10568229, 22_10619630, 22_10682376, 22_10701464, 22_10721852, 22_10736367, 22_10794796, 22_10796109, 22_10851347, 22_10860919, 22_10879790, 22_10901931, 22_10918711, 22_10943477, 22_10959385, 22_10965148, 22_10970469, 22_10981095, 22_10989551, 22_11007607, 22_11011666, 22_11038362, 22_11067018, 22_11083112, 22_11116247, 22_11119861, 22_11143274, 22_11151316, 22_11207263, 22_11261197, 22_11262726, 22_11277147, 22_11281307, 22_11293268, 22_11325666, 22_11342087, 22_11393782, 22_11404589, 22_11427085, 22_11428642, 22_11501216, 22_11585825, 22_11599576, 22_11611201, 22_11611666, 22_11649433, 22_11656627, 22_11706618, 22_11711647, 22_11744536, 22_11746983, 22_11757477, 22_11769230, 22_11782325, 22_11788465, 22_11801248, 22_11825712, 22_11825803, 22_11871219, 22_11878189, 22_11900862, 22_11917163, 22_11922083, 22_11966804, 22_12152381, 22_12274255, 22_12297183, 22_12344256, 22_12373101, 22_12546276, 22_12555080, 22_12563584, 22_12584329, 22_12823206, 22_12846807, 22_12855679, 22_12859528, 22_12989221, 22_13025575, 22_13031203, 22_13034852, 22_13051743, 22_13132606, 22_13238494, 22_13239841, 22_13245409, 22_13247298, 22_13271682, 22_13277106, 22_13280294, 22_13335073, 22_13357852, 22_13360056, 22_13385758, 22_13405667, 22_13409395, 22_13409688, 22_13410148, 22_13453426, 22_13500227, 22_13576788, 22_13583688, 22_13654124, 22_13662362, 22_13671376, 22_13739284, 22_13756183, 22_13767568, 22_13775214, 22_13777515, 22_13837260, 22_13879690, 22_13908346, 22_13940943, 22_13944163, 22_13949387, 22_13962290, 22_13979192, 22_14072831, 22_14092367, 22_14135147, 22_14189782, 22_14202142, 22_14212436, 22_14243795, 22_14245627, 22_14274595, 22_14366995, 22_14448295, 22_14465979, 22_14466786, 22_14539501, 22_14630195, 22_14635952, 22_14645234, 22_14754076, 22_14802376, 22_14835396

Pop
[truncated: 4,623,569 more chars]
