# Supplementary material for: Genomic Signatures After Five Generations of Intensive Selective Breeding: Runs of Homozygosity and Genetic Diversity in Representative Domestic and Wild Populations of Turbot (Scophthalmus maximus)
Source: Front Genet. 2020 Apr 3;11:296. doi: 10.3389/fgene.2020.00296 (PMC7169425; doi:10.3389/fgene.2020.00296)
Supplement: Supplementary file 7 [file Data_Sheet_1.PDF]

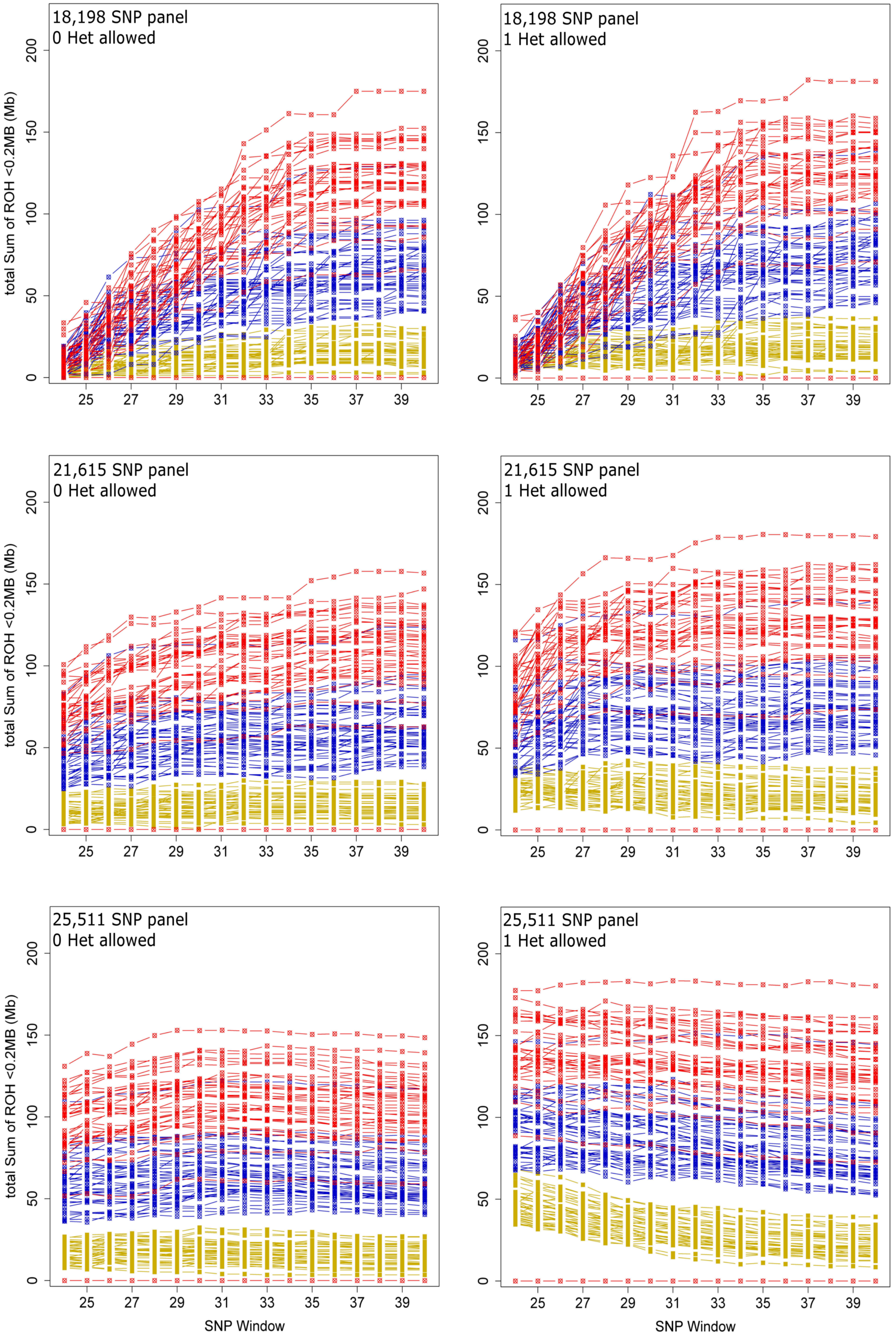

Supplementary Figure 1. Total sum of ROH in domesticated turbot. Three SNP panels (18,198 SNP, 21,615 SNP and 25,511 SNP) were tested, with different window sizes (24-40 SNP) and none or 1 heterozygous SNP (Het) allowed per window. Families were classified according to their parent's kinship coefficients (θIBD): red (θIBD >0.0937), blue (0.0076 < θIBD < 0.0937) and yellow (0 < θIBD < 0.0076).
