# Supplementary material for: Genomic Signatures After Five Generations of Intensive Selective Breeding: Runs of Homozygosity and Genetic Diversity in Representative Domestic and Wild Populations of Turbot (Scophthalmus maximus)
Source: Front Genet. 2020 Apr 3;11:296. doi: 10.3389/fgene.2020.00296 (PMC7169425; doi:10.3389/fgene.2020.00296)
Supplement: Supplementary file 8 [file Data_Sheet_2.PDF]

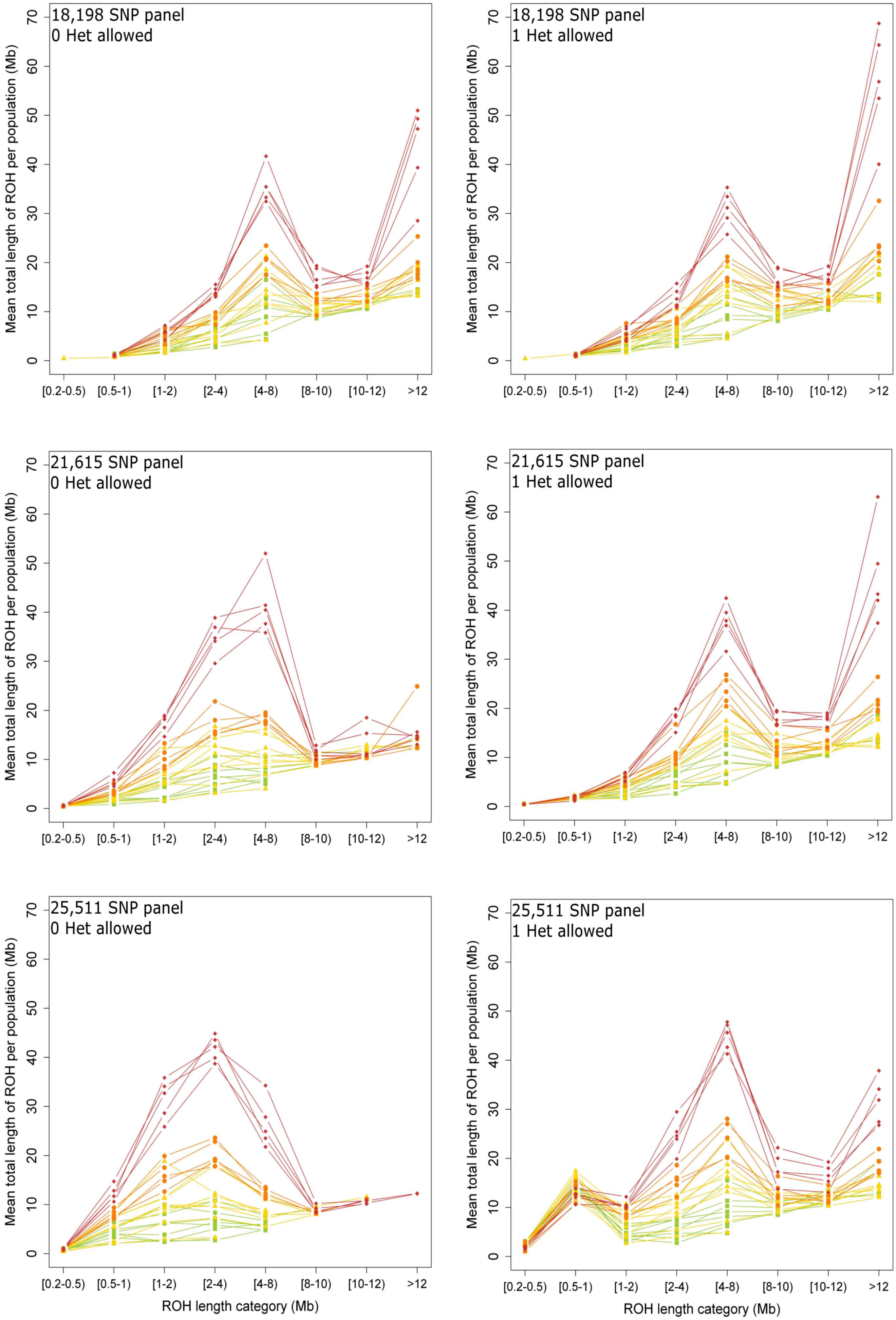

Supplementary Figure 2. Mean sum of ROH in different length categories. Three SNP panels (18,198 SNP, 21,615 SNP and 25,511 SNP) were tested and none or 1 heterozygous SNP (Het) allowed per window. Families were classified according to their parents kinship coefficients (θIBD): unrelated (green: $0 < \theta IBD < 0.0076$), second cousin (yellow-circle: $0.0076 < \theta IBD < 0.038$), first cousin (yellow-triangle: $0.038 < \theta IBD < 0.0937$), half-sibling (orange: $0.0937 < \theta IBD < 0.1872$) and full-sibling (red: $\theta IBD > 0.18$).
