# Supplementary material for: Genomic Signatures After Five Generations of Intensive Selective Breeding: Runs of Homozygosity and Genetic Diversity in Representative Domestic and Wild Populations of Turbot (Scophthalmus maximus)
Source: Front Genet. 2020 Apr 3;11:296. doi: 10.3389/fgene.2020.00296 (PMC7169425; doi:10.3389/fgene.2020.00296)
Supplement: Supplementary file 9 [file Data_Sheet_3.PDF]

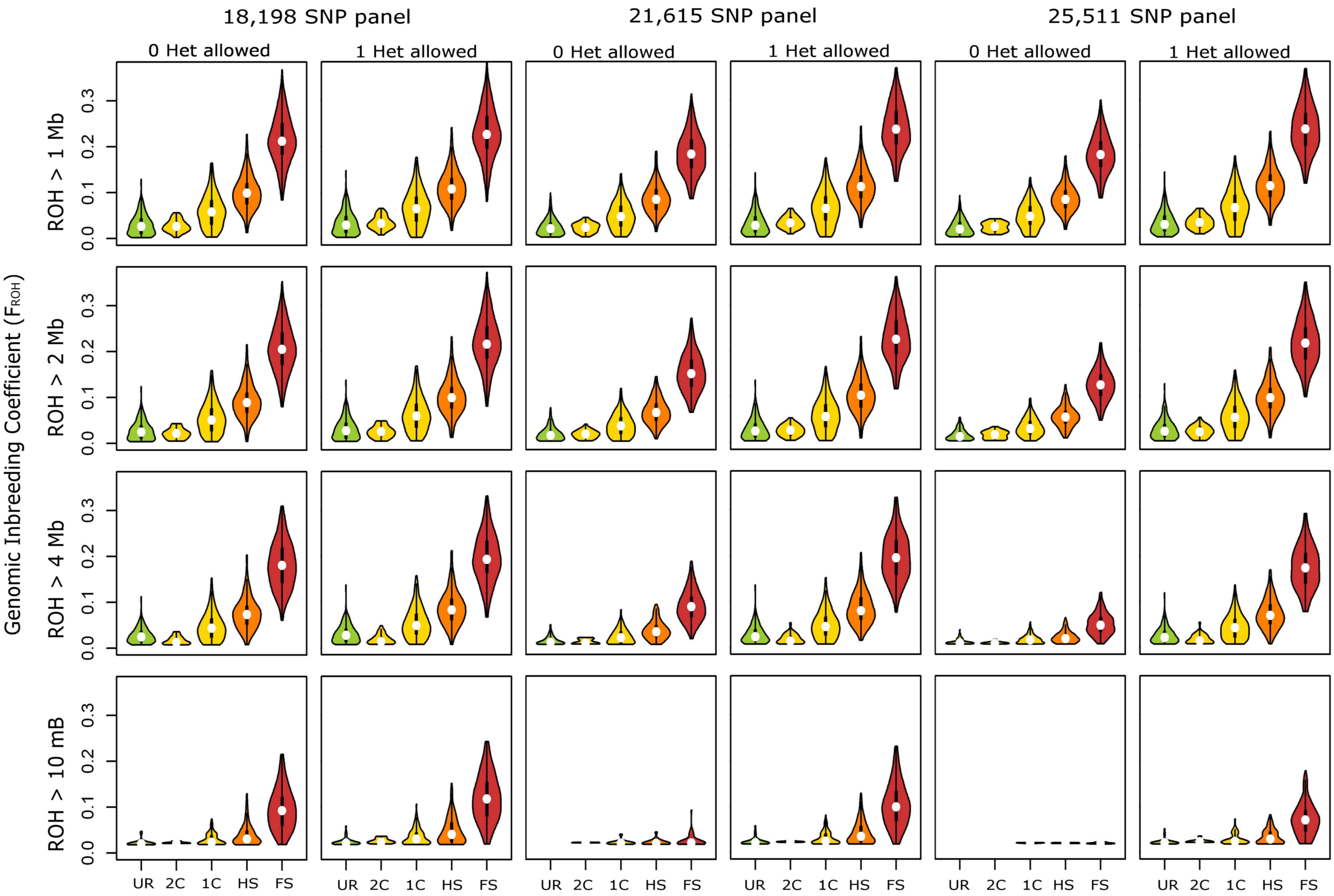

Supplementary Figure 3. Distribution of ROH by different size cut-offs and kinship categories. Three SNP panels were tested and none or 1 heterozygous SNP (Het) allowed for each kinship coefficient θIBD category. Unrelated (UR; green: 0 < θIBD <0.0076), second cousin (2C; yellow: 0.0076< θIBD <0.038), first cousin (1C; yellow: 0.038 < θIBD < 0.0937), half-sibling (HS; orange: 0.0937 < θIBD < 0.1872) and full-sibling (FS; red: θIBD > 0.1872).
