# Supplementary material for: Genomic Signatures After Five Generations of Intensive Selective Breeding: Runs of Homozygosity and Genetic Diversity in Representative Domestic and Wild Populations of Turbot (Scophthalmus maximus)
Source: Front Genet. 2020 Apr 3;11:296. doi: 10.3389/fgene.2020.00296 (PMC7169425; doi:10.3389/fgene.2020.00296)
Supplement: Supplementary file 10 [file Data_Sheet_4.PDF]

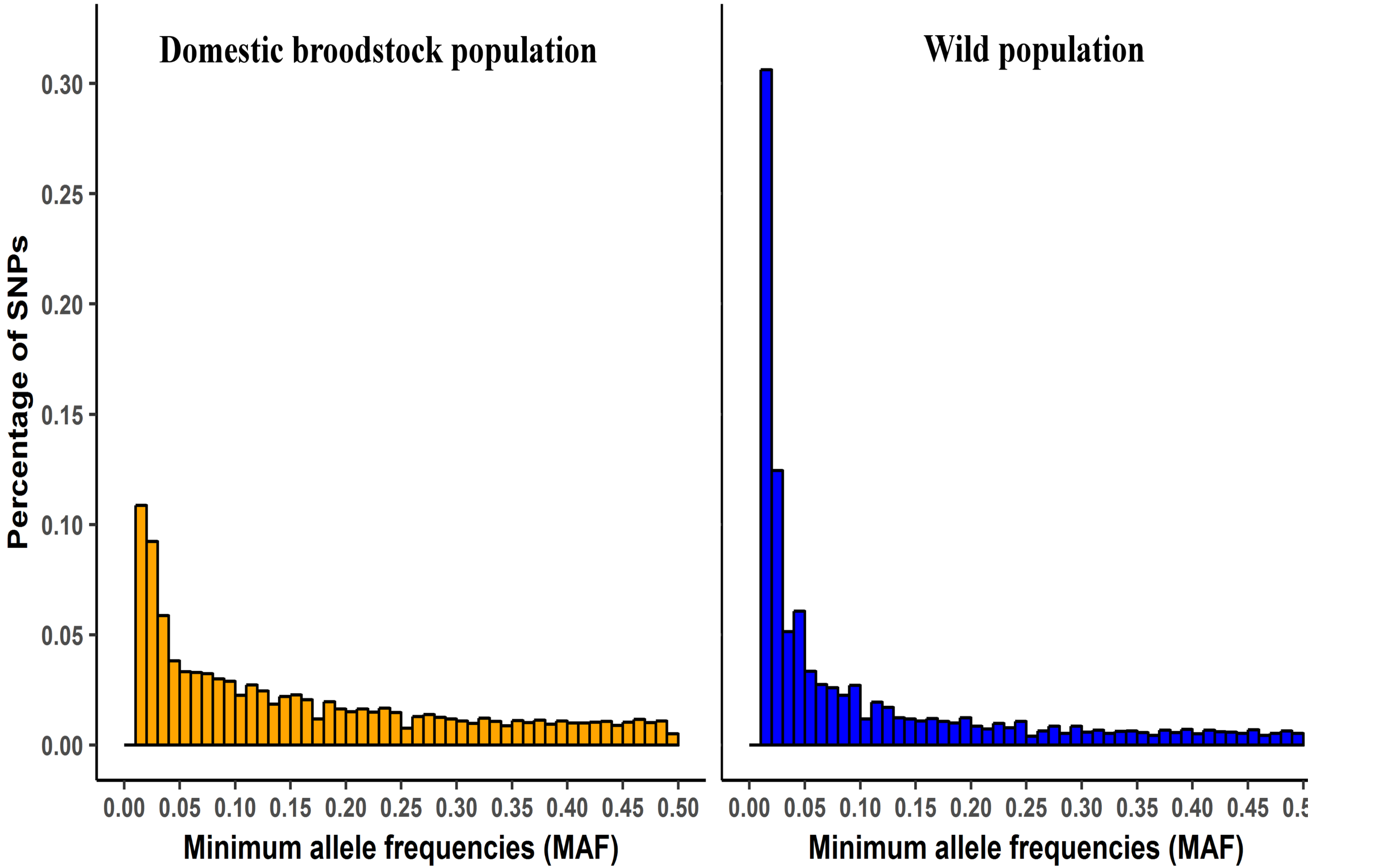

Supplementary Figure 4. Distribution of the Minimum Allele Frequency per SNP (MAF) in the broodstock and wild populations.
