# Supplementary material for: Genomic Signatures After Five Generations of Intensive Selective Breeding: Runs of Homozygosity and Genetic Diversity in Representative Domestic and Wild Populations of Turbot (Scophthalmus maximus)
Source: Front Genet. 2020 Apr 3;11:296. doi: 10.3389/fgene.2020.00296 (PMC7169425; doi:10.3389/fgene.2020.00296)
Supplement: Supplementary file 11 [file Data_Sheet_5.PDF]

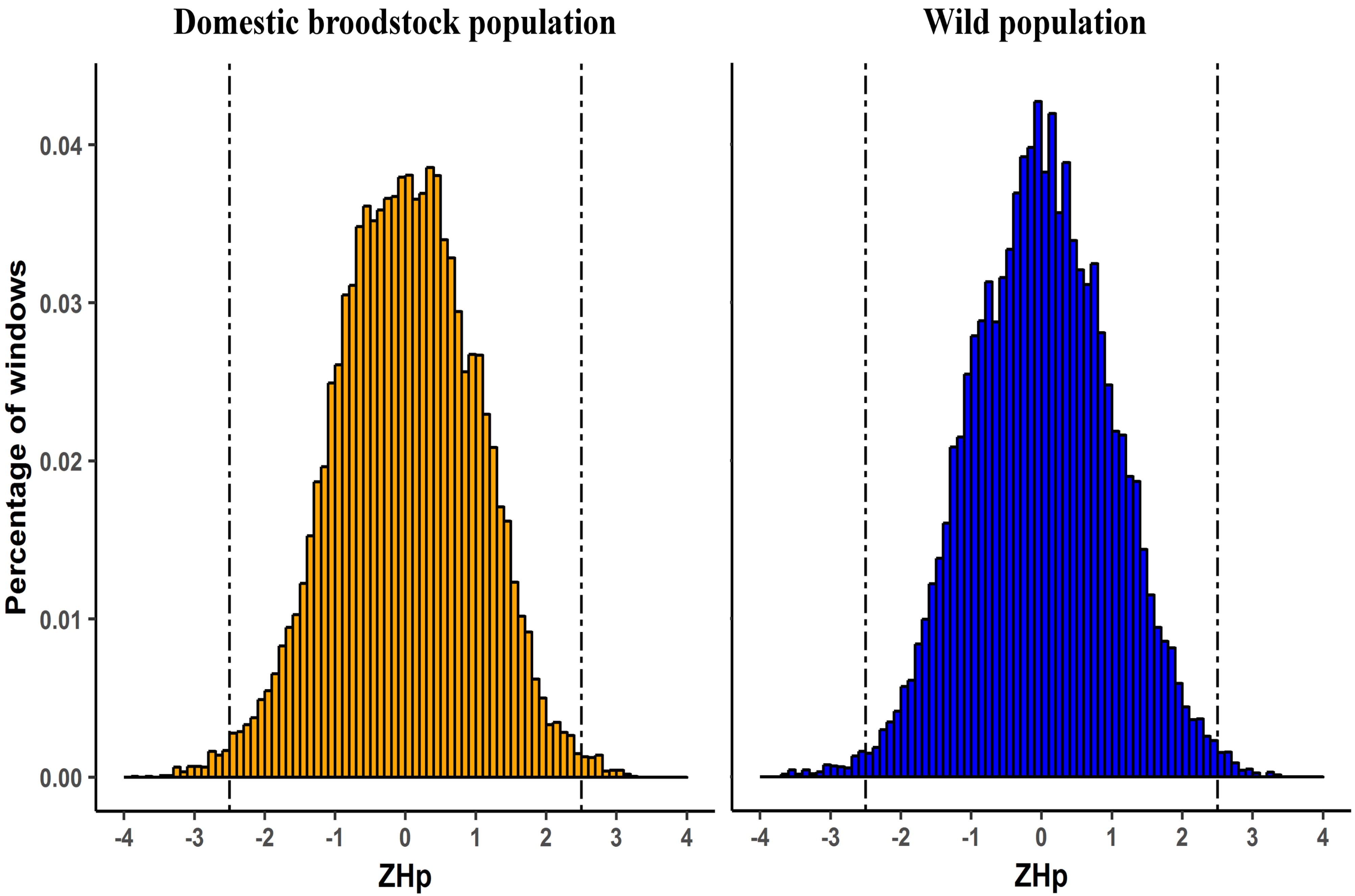

Supplementary Figure 5. Distribution of Z-transformed pooled heterozygosity (ZHp) per 37-SNP window in the broodstock and wild populations.
