# Supplementary material for: Genomic Signatures After Five Generations of Intensive Selective Breeding: Runs of Homozygosity and Genetic Diversity in Representative Domestic and Wild Populations of Turbot (Scophthalmus maximus)
Source: Front Genet. 2020 Apr 3;11:296. doi: 10.3389/fgene.2020.00296 (PMC7169425; doi:10.3389/fgene.2020.00296)
Supplement: Supplementary file 12 [file Data_Sheet_6.PDF]

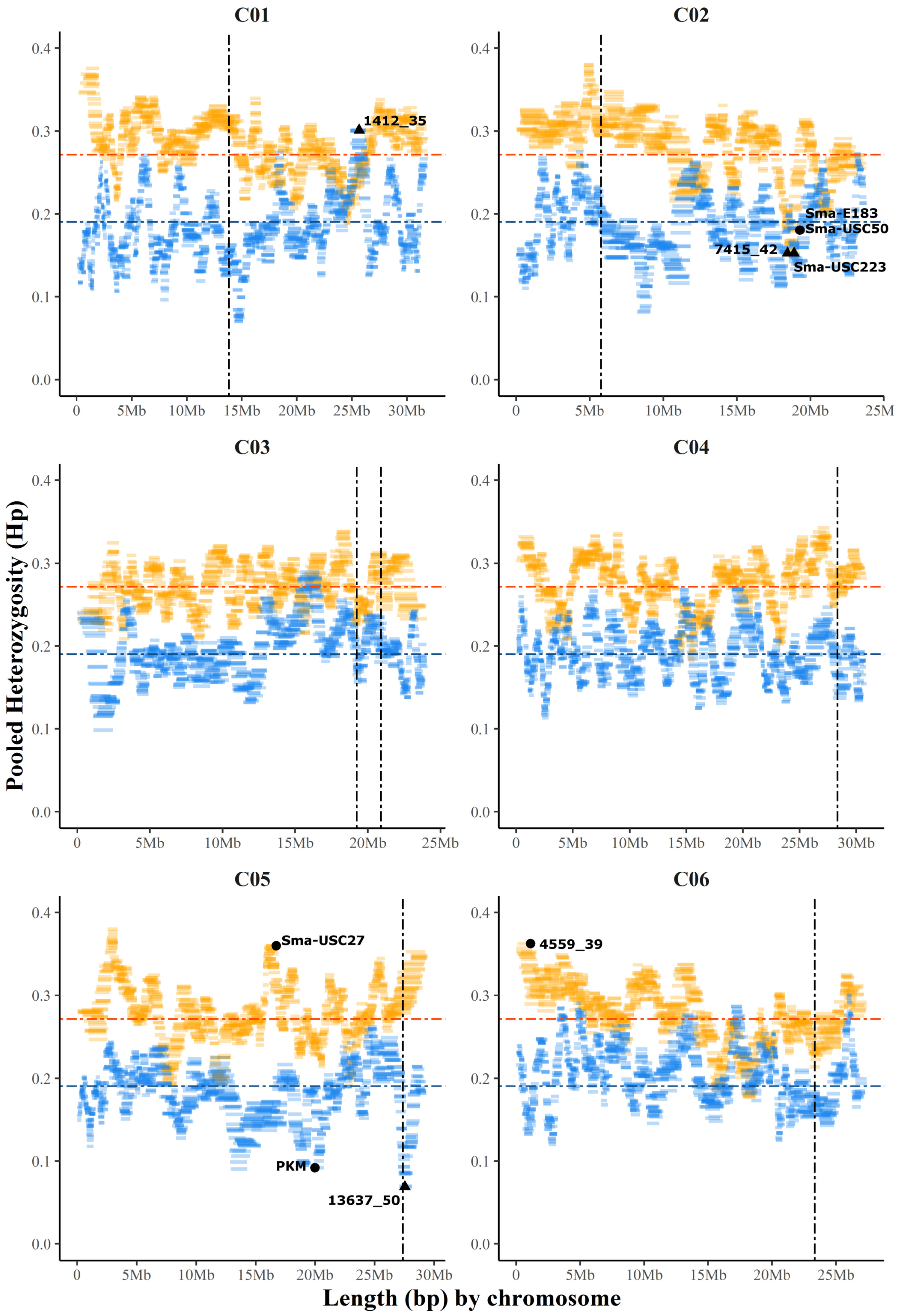

Supplementary Figure 6. Genome-wide distribution of pooled heterozygosity (Hp) for both populations. Hp estimates were obtained using 37-SNP sliding windows across each chromosome of broodstock (orange segments) and wild (blue segments) turbot populations. Different SNP panels were used for each population. Averages for broodstock and wild windows were represented as dotted horizontal lines (orange and blue, respectively). Relevant markers included inside suggestive low and/or high genetic diversity regions had their positions graphed as black dots; those included in strict low and/or high genetic diversity are graphed as black triangles. Dotted vertical lines represent centromeric regions.

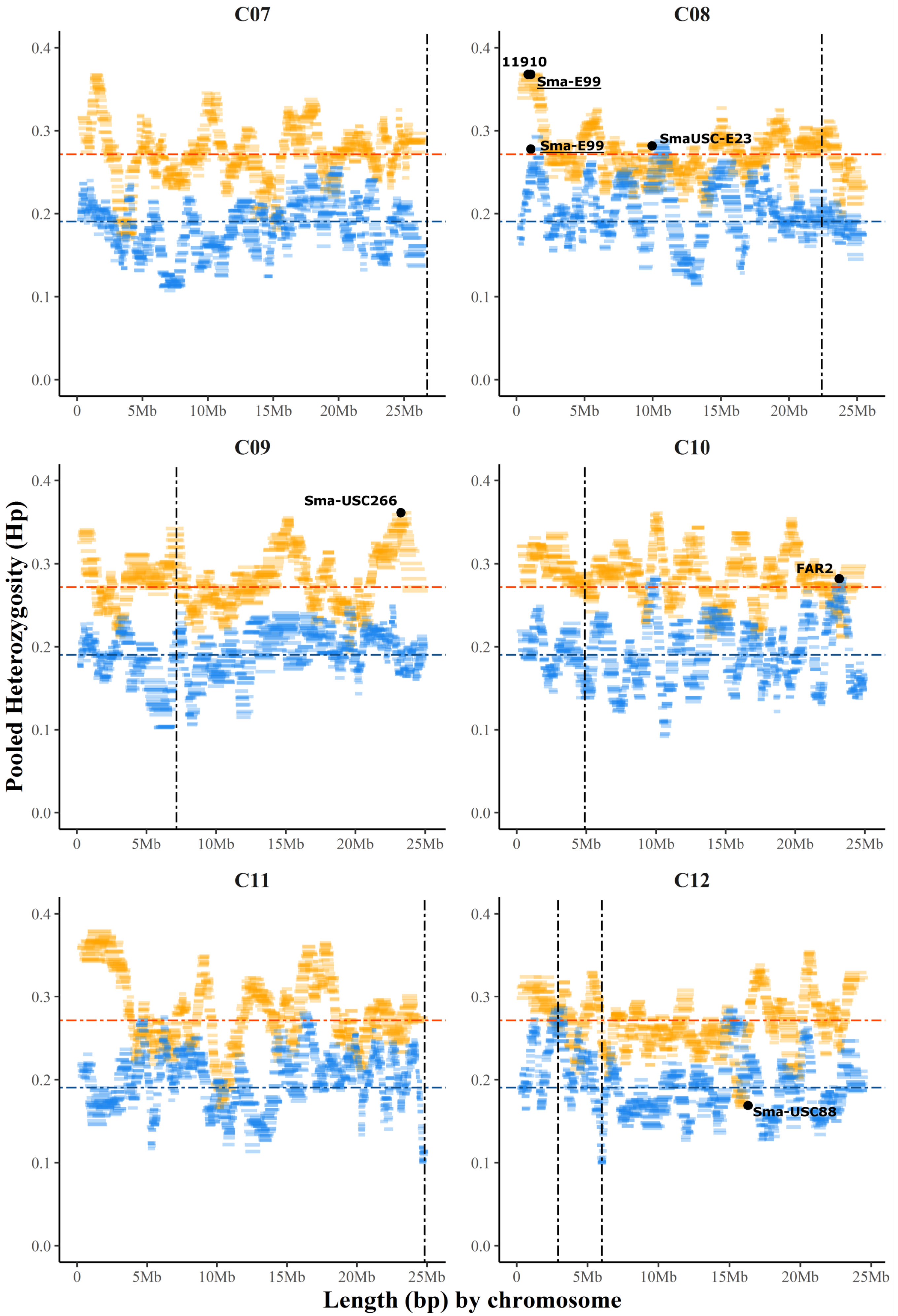

Supplementary Figure 6. Genome-wide distribution of pooled heterozygosity (Hp) for both populations. Hp estimates were obtained using 37-SNP sliding windows across each chromosome of broodstock (orange segments) and wild (blue segments) turbot populations. Different SNP panels were used for each population. Averages for broodstock and wild windows were represented as dotted horizontal lines (orange and blue, respectively). Relevant markers included inside suggestive low and/or high genetic diversity regions had their positions graphed as black dots; those included in strict low and/or high genetic diversity are graphed as black triangles. Dotted vertical lines represent centromeric regions.

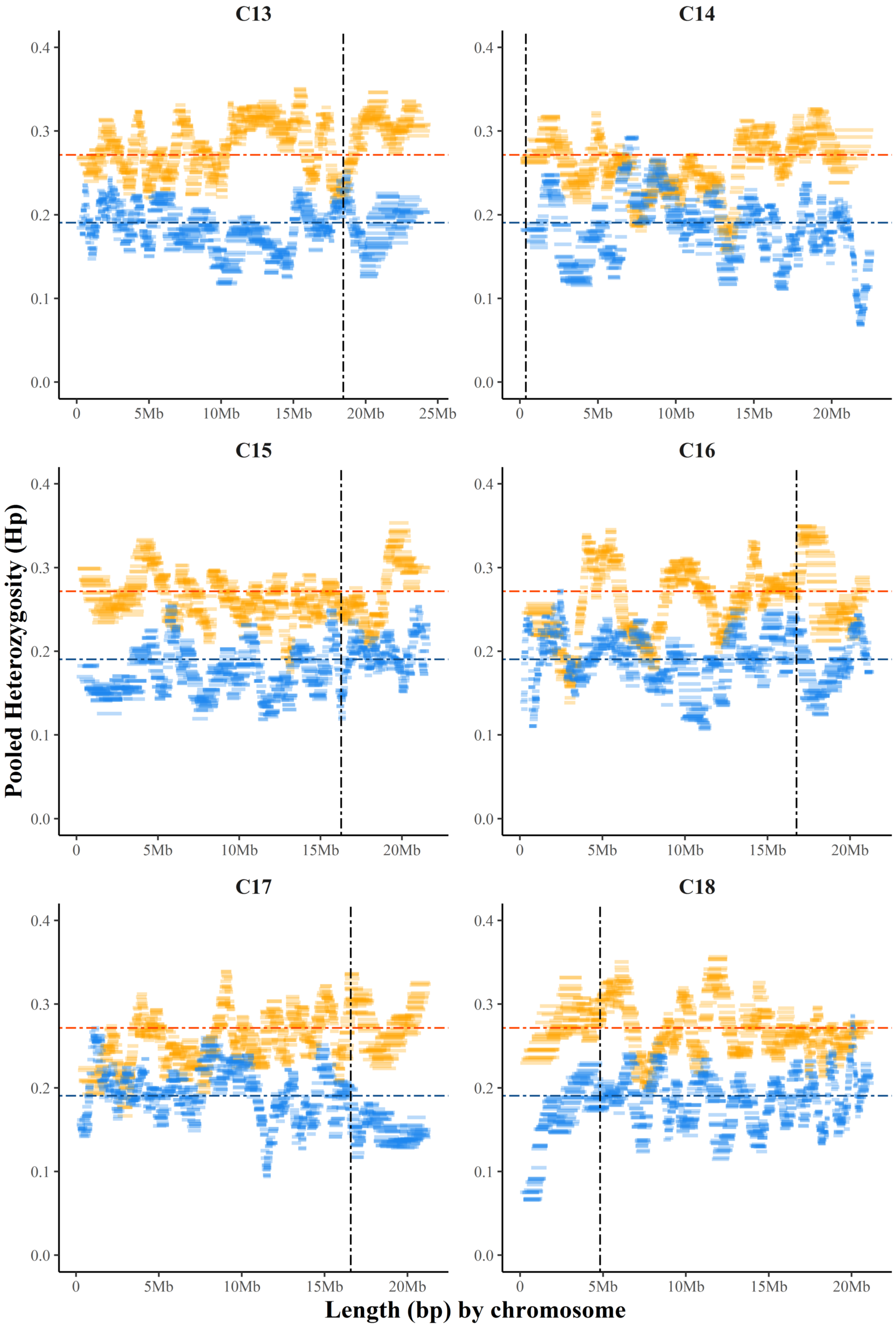

Supplementary Figure 6. Genome-wide distribution of pooled heterozygosity (Hp) for both populations. Hp estimates were obtained using 37-SNP sliding windows across each chromosome of broodstock (orange segments) and wild (blue segments) turbot populations. Different SNP panels were used for each population. Averages for broodstock and wild windows were represented as dotted horizontal lines (orange and blue, respectively). Relevant markers included inside suggestive low and/or high genetic diversity regions had their positions graphed as black dots; those included in strict low and/or high genetic diversity are graphed as black triangles. Dotted vertical lines represent centromeric regions.

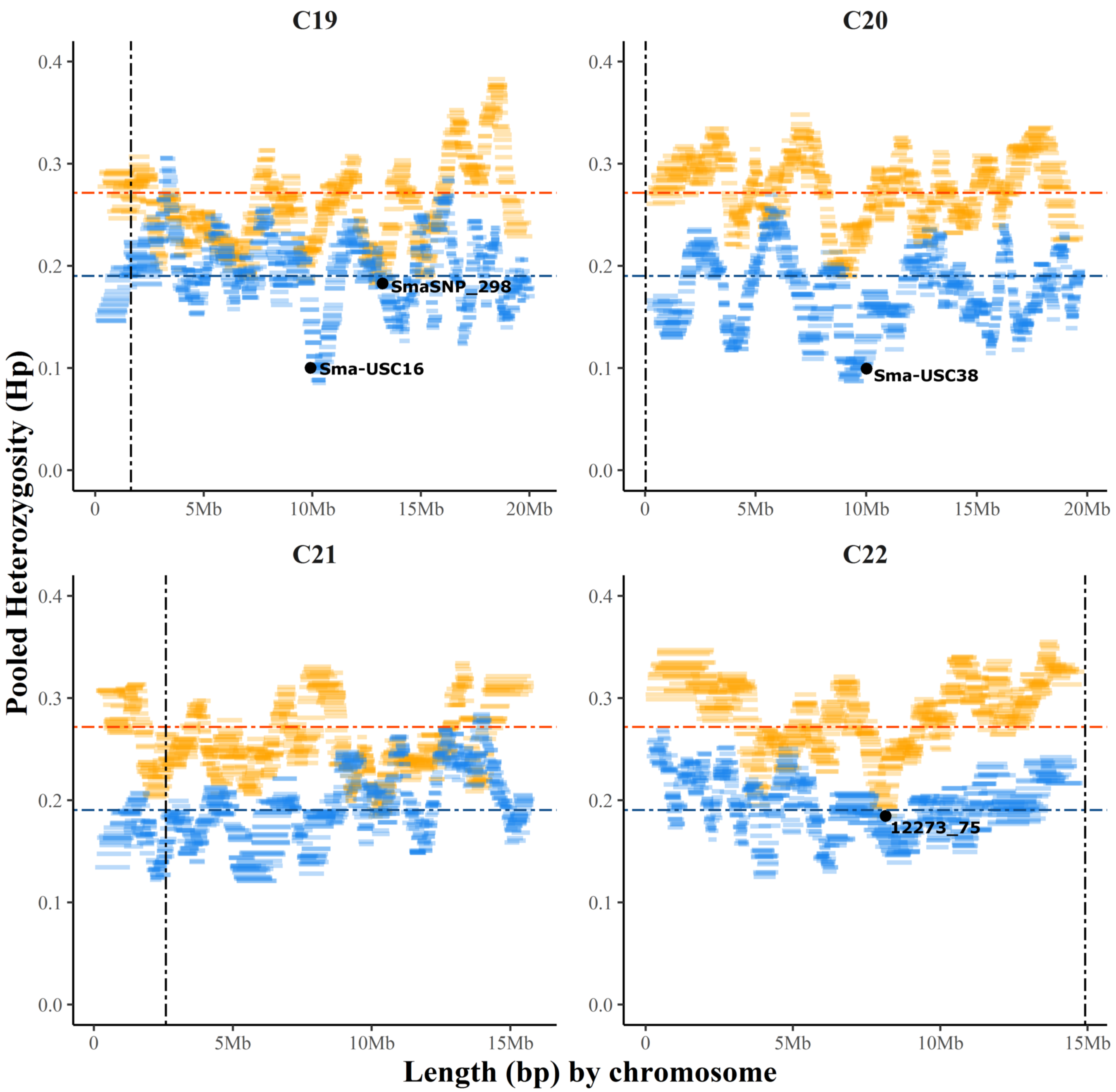

Supplementary Figure 6. Genome-wide distribution of pooled heterozygosity (Hp) for both populations. Hp estimates were obtained using 37-SNP sliding windows across each chromosome of broodstock (orange segments) and wild (blue segments) turbot populatio ns. Different SNP panels were used for each population. Averages for broodstock and wild windows were represented as dotted horizontal line s (orange and blue, respectively). Relevant markers included inside suggestive low and/or high genetic diversity regions had their positions gra phed as black dots; those included in strict low and/or high genetic diversity are graphed as black triangles. Dotted vertical lines represent centr omeric regions.
