# Supplementary material for: Genomic Signatures After Five Generations of Intensive Selective Breeding: Runs of Homozygosity and Genetic Diversity in Representative Domestic and Wild Populations of Turbot (Scophthalmus maximus)
Source: Front Genet. 2020 Apr 3;11:296. doi: 10.3389/fgene.2020.00296 (PMC7169425; doi:10.3389/fgene.2020.00296)
Supplement: Supplementary file 13 [file Data_Sheet_7.PDF]

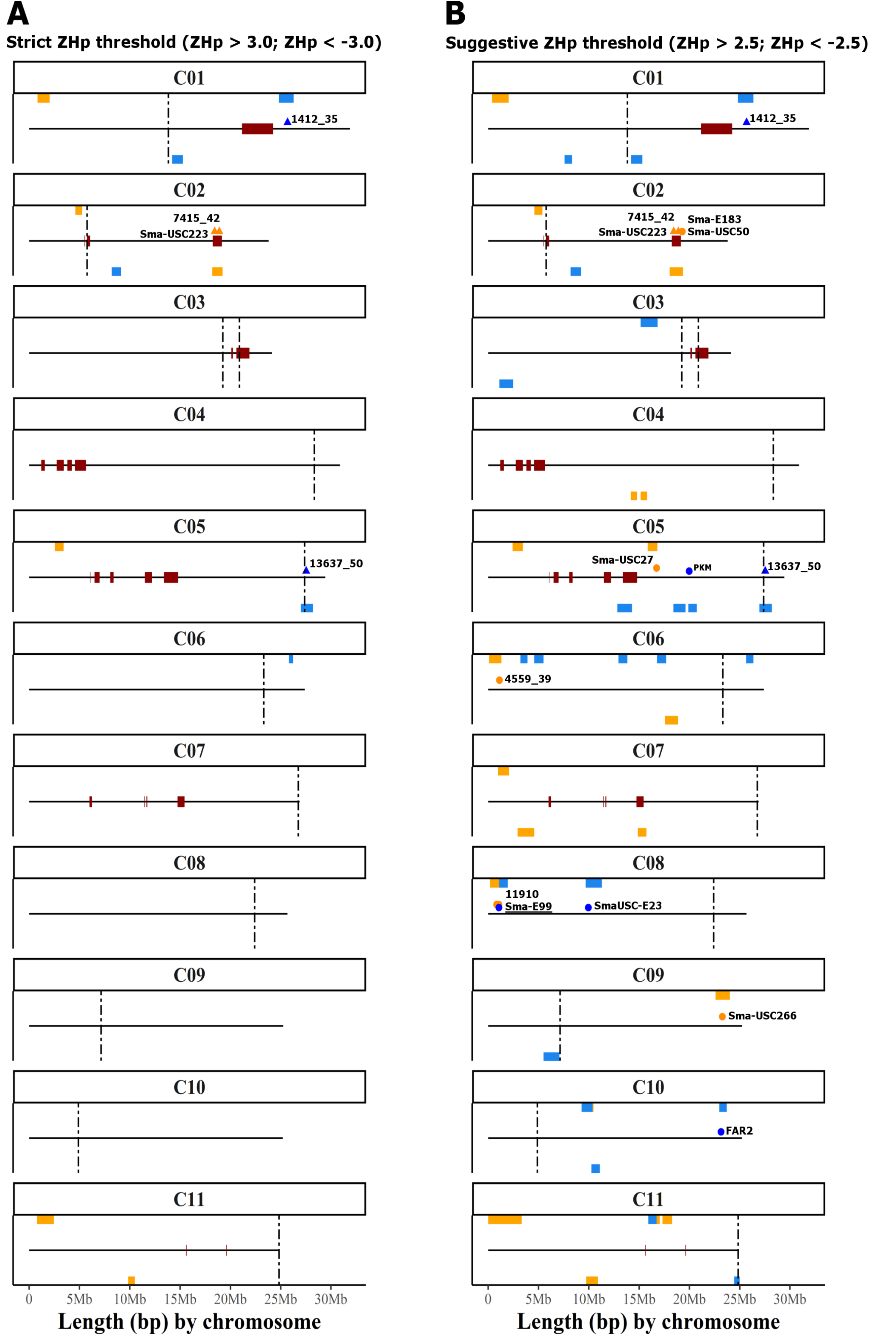


**Supplementary Figure 7. Genome-wide distribution of ROHi and low-/high-GD windows in the broodstock population and low-/high-GD windows in the wild population, according to both strict (A) and suggestive (B) ZHp thresholds.** Different SNP panels were used for each population. Broodstock ROHi are shown as red segments; high-/low-genetic diversity (GD) regions are drawn as orange segments for broodstock and blue segments for wild; in both cases, high-GD regions are represented above the horizontal black line, while low-GD windows are located below. Relevant suggestive markers included inside low-GD and/or high-GD regions in broodstock (orange) and wild (blue) had their positions graphed.

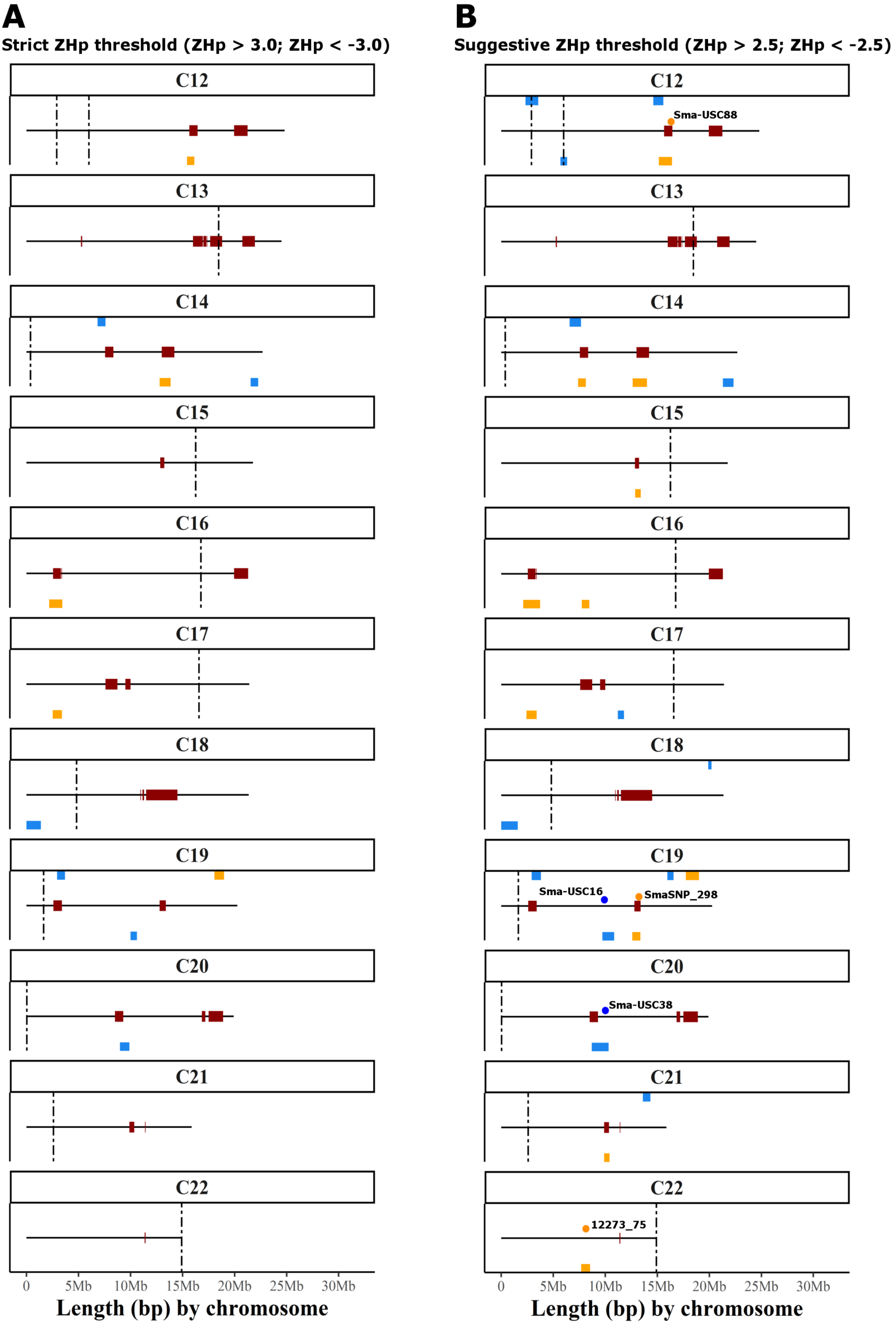


**Supplementary Figure 7. Genome-wide distribution of ROHi and low-/high-GD windows in the broodstock population and low-/high-GD windows in the wild population, according to both strict (A) and suggestive (B) ZHp thresholds.** Different SNP panels were used for each population. Broodstock ROHi are shown as red segments; high-/low-genetic diversity (GD) regions are drawn as orange segments for broodstock and blue segments for wild; in both cases, high-GD regions are represented above the horizontal black line, while low-GD windows are located below. Relevant suggestive markers included inside low-GD and/or high-GD regions in broodstock (orange) and wild (blue) had their positions graphed.
